# Psiguajadials A–K: Unusual *Psidium* Meroterpenoids as Phosphodiesterase-4 Inhibitors from the Leaves of *Psidium guajava*

**DOI:** 10.1038/s41598-017-01028-4

**Published:** 2017-04-21

**Authors:** Gui-Hua Tang, Zhen Dong, Yan-Qiong Guo, Zhong-Bin Cheng, Chu-Jun Zhou, Sheng Yin

**Affiliations:** School of Pharmaceutical Sciences, Sun Yat-sen University, Guangzhou, 510006 People’s Republic of China

## Abstract

Bioassay-guided fractionation of the ethanolic extract of the leaves of *Psidium guajava* led to the isolation of 11 new *Psidium* meroterpenoids, psiguajadials A–K (**1**–**11**), along with 17 known ones (**12**–**28**). Their structures and absolute configurations were elucidated by spectroscopic methods and comparison of experimental and calculated ECD. Compounds **1** and **2** represent two unprecedented skeletons of 3,5-diformyl-benzyl phloroglucinol-coupled sesquiterpenoid, while **3** is the first example of *Psidium* meroterpenoids coupling *via* an oxepane ring. Putative biosynthetic pathways towards **1** and **2** are proposed. Compounds **1**–**13** and **16**–**26** exhibited moderate inhibitory activities against phosphodiesterase-4 (PDE4), a drug target for asthma and chronic obstructive pulmonary disease, with IC_50_ values in the range of 1.34–7.26 *μ*M.

## Introduction

Phloroglucinol-coupled mono- or sesquiterpenoids are a group of structurally diverse meroterpenoids mainly occurring in species of the genera *Eucalyptus, Psidium*, *Rhodomyrtus*, *Myrtus* (Myrtaceae), and *Hypericum* (Guttiferae)^1–17^. Biosynthetically, they are proposed to be adducts of phloroglucinol derivatives and terpenoids *via* hetero-Diels-Alder or carbocation-induced stepwise reactions^6, 10^. So far more than 100 phloroglucinol-coupled terpenoids have been reported^11, 18^, and their intriguing structures and important biological activities have attracted broad interest from both natural products and synthetic chemists over the last half century^5, 19–24^. For instance, psiguadials A, C, and D^9, 10^, hyperjapones A–E^16^, and hyperjaponols A and C^17^ with novel skeletons and appealing pharmacological activities (e.g., antitumor and antiviral properties) have been synthesized by several groups^25, 26^. *Psidium* meroterpenoids are a subgroup of this compound class, which are exclusively reported from the species *Psidium guajava*. Structurally, they are characterized by the presences of 3,5-diformyl-benzyl phloroglucinol moiety and a dihydropyran ring junction. Recently, a number of excellent articles on the separation or biomimetic total synthesis of *Psidium* meroterpenoids have increased the interest in the study of this type of compounds^8, 25, 27^.

Phosphodiesterase-4 (PDE4), which specifically catalyzes the hydrolysis of the second messenger cyclic adenosine monophosphate (cAMP), is a therapeutic target of high interest for central nervous system (CNS), inflammatory, and respiratory diseases^28^. Although a number of structurally diverse PDE4 inhibitors have been developed over the last decade, roflumilast is the only one that successfully launched on the market to treat asthma and chronic obstructive pulmonary disease^29^. However, the wide clinical use of roflumilast has been limited by the dose-limiting side effects, such as diarrhea, nausea, headaches, and weight loss. Therefore, the discovery of novel PDE4 inhibitors with stronger potency and less side-effects continues unabated.

In our continuing search for novel PDE4 inhibitors from Chinese herbs^30–32^, a fraction of the 95% aqueous ethanol extract of *Psidium guajava* exhibited moderate inhibitory effect on PDE4 (inhibitory rate >50% at 10 *μ*g/mL). Subsequent chemical investigation of this fraction led to the isolation of 28 *Psidium* meroterpenoids including 11 new ones (**1**–**11**). Bioassay verified that most of these meroterpenoids were responsible for the activity of the crude fraction. Herein, we report the isolation, structure elucidation, putative biosynthetic origin, and PDE4 inhibitory activities of these metabolites.

## Results and Discussion

**Figure 1:**
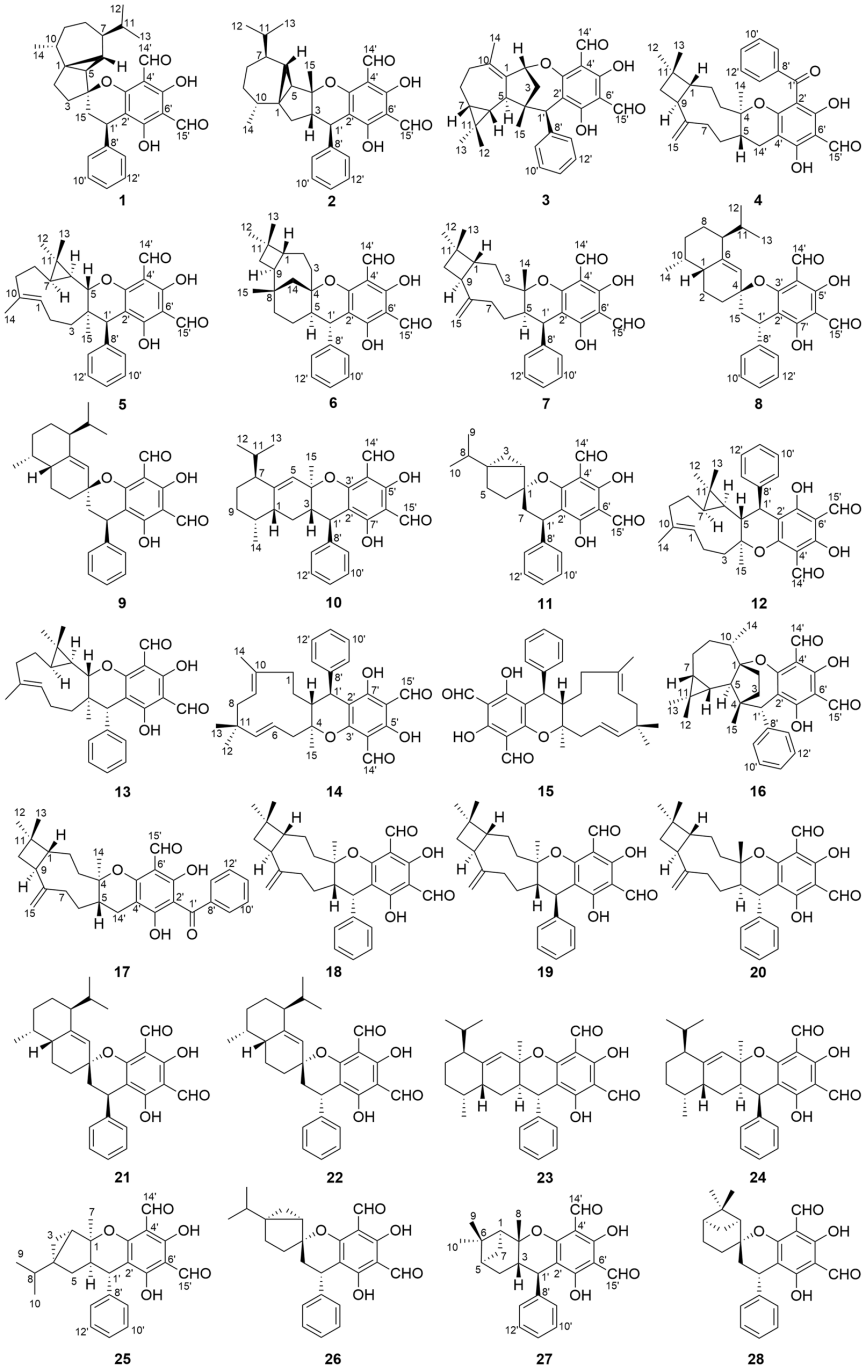
Structures of compounds **1**–**28**.

Leaves of *P. guajava* (6.0 kg) was extracted with 95% EtOH at room temperature (rt) to give a residue, which was suspended in H_2_O and partitioned with EtOAc and *n*-BuOH, respectively. Subsequent purification of the EtOAc fraction with various column chromatographic methods afforded compounds **1**–**28** (Fig. 1).

Psiguajadial A (**1**), a colourless oil, had a molecular formula of C_30_H_34_O_5_ as determined by the HR-ESI-MS ion at *m/z* 473.2304 [M – H]^−^ (calcd 473.2333), corresponding to 14 degrees of unsaturation (DOUs). The IR absorption bands revealed the presence of OH (3448 cm^−1^) and conjugated carbonyl (1633 cm^−1^) groups. The ^1^H NMR data (Table 1) displayed signals for three secondary methyl groups [*δ*_H_ 1.00 (3H, d, *J* = 6.3 Hz), 0.89 (3H, d, *J* = 6.6 Hz), and 0.85 (3H, d, *J* = 6.6 Hz)], two formyl groups [*δ*_H_ 10.13 and 10.12 (each 1H, s)], two chelated phenolic hydroxyls [*δ*_H_ 13.55 and 13.19 (each 1H, s)], a monosubstituted benzene ring [*δ*_H_ 7.14–7.28 (5H, m)], and a series of aliphatic multiplets. The ^13^C NMR spectrum, associated with DEPT experiments, resolved 30 carbon resonances attributable to two conjugated aldehydes (*δ*_C_ 192.2 and 191.6), a monosubstituted phenyl [*δ*_C_ 144.6, 128.6 (×2), 126.7 (×2), and 126.3], a hexasubstituted phenyl, three sp^3^ methyls, five sp^3^ methylenes, six sp^3^ methines, and two sp^3^ quaternary carbons (one oxygenated). The collective information suggested that **1** was a *Psidium* meroterpenoid possessing the basic structural features of a cubebane sesquiterpenoid unit^33^ and a 3,5-diformyl-benzyl phloroglucinol substructure^6^.

**Table 1:** ^1^H NMR (400 Hz) data of compounds **1**–**4** in CDCl_3_ (*δ* in ppm, *J* in Hz).

Position	1	2	3	4
1	—	—	—	1.66, m
2	*α* 1.82, m; *β* 1.59, m	*β* 1.96, dd (12.8, 7.9); *α* 1.77, t (12.0)	5.50, d (4.7)	0.92, m
3	*β* 1.77, m; *α* 1.60, m	2.28, t (9.3)	a 2.45, d (14.5); b 1.54, dd (14.5, 4.0)	*β* 1.60, m; *α* 0.90, m
5	1.13, d (2.9)	1.19, br. s	2.27, m	1.82, m
6	1.04, m	0.64, br. s	0.77, t (10.3)	*β* 1.57, m; *α* 1.49, m
7	0.84, m	1.00, m	0.57, m	*α* 2.36, m; *β* 2.03, m
8	*α* 1.40, m; *β* 0.81, m	*α* 1.42, m; *β* 0.83, m	*β* 1.67, m; *α* 1.54, m	—
9	*β* 1.65, m; *α* 0.53, dddd (12.1, 12.1, 12.1, 1.9)	*β* 1.61, m; *α* 0.51, q (12.4)	*α* 2.26, m; *β* 2.16, m	2.27, m
10	1.71, m	1.70, m	—	*α* 1.61, m; *β* 1.56, m
11	1.52, m	1.56, m	—	—
12	0.89, d (6.6)	0.93, d (6.6)	*β* 1.02, s	*α* 0.87, s
13	0.85, d (6.6)	0.90, d (6.7)	*α* 1.15, s	*β* 0.93, s
14	1.00, d (6.3)	0.75, d (6.2)	1.72, s	1.00, s
15	*α* 2.27, dd (14.1, 7.2); *β* 1.99, dd (14.1, 10.4)	1.08, s	1.08, s	4.83, br.s; 4.80, br.s
1′	4.17, dd (10.4, 7.2)	4.20, br. s	4.39, br. s	—﻿
9′/13′	7.14, m	7.09, d (7.5)	7.27, m	7.40, m
10′/12′	7.28, m	7.28, t (7.5)	7.24, m	7.39, m
11′	7.21, m	7.20, t (7.5)	7.19, t (6.7)	7.47, m
14′	10.12, s	10.18, s	10.22, s	*α* 2.61, dd (16.6, 5.4); *β* 2.00, dd (16.6, 12.1)
15′	10.13, s	10.16, s	10.10, s	10.23, s
5′-OH	13.55, s	13.61, s	13.53, s	13.23, s
7′-OH	13.19, s	13.23, s	13.44, s	13.71, s

**Figure 2:**
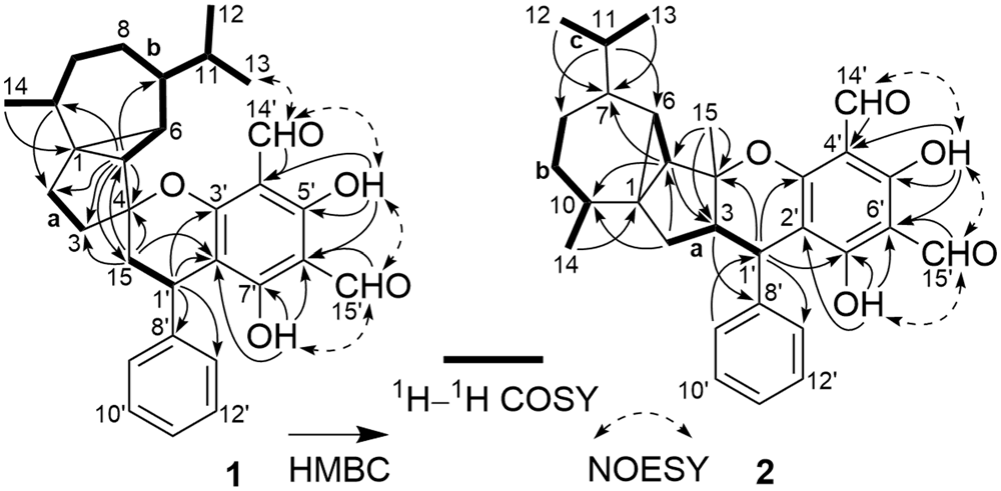
Selected ^1^H–^1^H COSY, HMBC, and NOESY correlations of **1** and **2**.

Detailed 2D NMR analysis (^1^H–^1^H COSY, HSQC, HMBC, and NOESY) further confirmed the presence of these moieties. In particular, two structural fragments **a** (C-2–C-3) and **b** (from C-5 to C-14) were first established by the ^1^H–^1^H COSY correlations (Fig. 2). The connectivities of **a**, **b**, the methylene (C-15), and two sp^3^ quaternary carbons (C-4 and C-5) were achieved by the HMBC correlations of H_3_-14/C-1, H-10/C-2, H-5/C-2, C-3, C-4, C-10, and C-15, and H_2_-15/C-3, C-4, and C-5 (Fig. 2), which led to the identification of the cubebane moiety. The HMBC correlations from two phenolic hydroxyls (5′-OH and 7′-OH) and two formyl protons (H-14′ and H-15′) to C-5′, C-7′, C-4′, and C-6′ of a benzene ring, respectively, together with the NOE correlations of H-15′/5′-OH and 7′-OH, and H-14′/5′-OH outlined a 3,5-diformyl phloroglucinol fragment. The aforementioned fragment and a mono-substituted benzene ring were connected to C-1′ by the HMBC correlations of H-1′/C-2′, C-3′, C-7′, C-8′, C-9′, and C-13′ to generate a 3,5-diformyl-benzyl phloroglucinol moiety. The connection between cubebane and 3,5-diformylbenzyl phloroglucinol moieties *via* C-1′–C-15 bond was identified by ^1^H–^1^H COSY correlations of H-1′/H-15. As 13 of the 14 DOUs were accounted for by two aldehydes, two benzene rings, and three rings of cubebane, the remaining DOU required the presence of an additional ring in **1**. Although no direct HMBC correlations were available to construct the additional ring, the still “loose ends” of an oxygenated quaternary carbon (*δ*_C_ 89.8, C-4) and a downfield-shifted aromatic carbon (*δ*_C_ 165.8, C-3′) suggested that an oxygen bridge was located between C-4 and C-3′ to form a dihydropyran ring. This was further supported by the NOE correlation between H-13 (12) and H-14′. Thus, a planar structure of **1** was proposed as depicted in Fig. 2, which was fully consistent with its molecular composition. Compound **1** represents a new skeleton of sesquiterpenoid-based *Psidium* meroterpenoid with the presence of cubebane moiety in this compound class for the first time.

**Figure 3:**
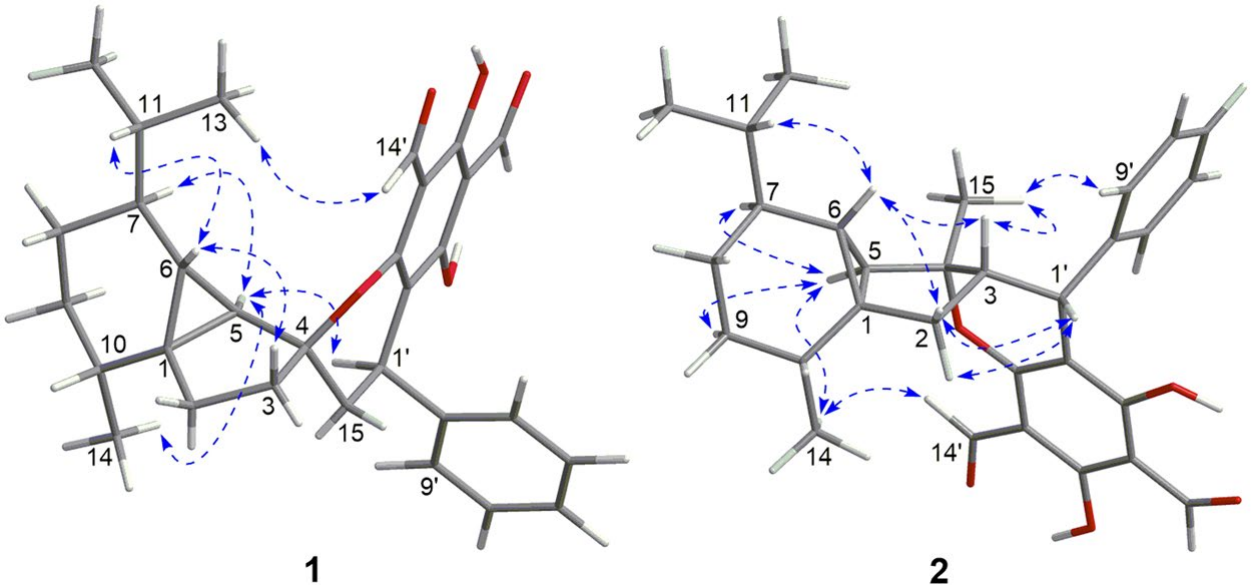
Selected NOESY correlations of **1** and **2**.

The relative configuration of **1** was established on the basis of NOESY experiments (recorded in both CDCl_3_ and pyridine-*d*_5_) and ^1^H−^1^H coupling constant analysis. The NOE correlations of H-5/H-7, H-9*α*, and H_3_-14 indicated that these protons or functional groups were cofacial and were arbitrarily assigned *α*-orientations (Fig. 3). Accordingly, the NOE correlations of H-6/H-3*β* and H-11 designated H-6 to be *β*-oriented. The *trans*-relationship of H-5 and H-6 on the cyclopropane ring was further supported by the small coupling constant (*J* = 2.9 Hz) between them^33^. Finally, the *β*-orientation of C-4–*O*–C-3′ bond at the spirocenter and the α-orientation of H-1′ were assigned by NOE correlations of H-5/H-1′ and H_3_-13(12)/H-14′, respectively.

**Figure 4:**
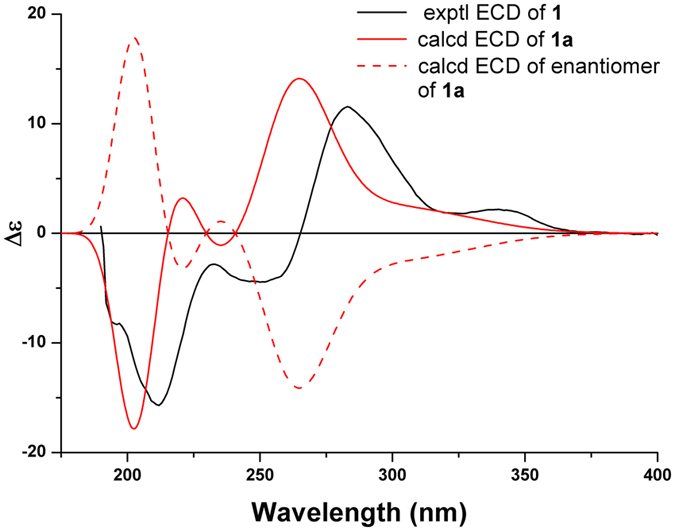
Experimental ECD spectrum of **1** and TDDFT calculated ECD spectra for **1a** (1*R*, 4*R*, 5*R*, 6*R*, 7*S*, 10*R*, 1′*R*) and enantiomer of **1a**.

To determine the absolute configuration of **1**, the experimental ECD spectrum of **1** was compared with those calculated for its isomers by the TDDFT method. In Fig. 4, the experimental ECD spectrum of **1** showed sequential positive, positive, negative, and negative Cotton effects at 340, 283, 250, and 212 nm, respectively, which matched the calculated ECD curve for **1a**, the isomer with the 1*R*, 4*R*, 5*R*, 6*R*, 7*S*, 10*R*, 1′*R* absolute configuration, indicating that **1** possessed the same absolute configuration as **1a**.

Psiguajadial B (**2**), a colourless oil, exhibited a molecular formula of C_30_H_34_O_5_ as determined by HR-ESI-MS at *m*/*z* 475.2475 [M + H]^+^ (calcd 475.2479), indicating that it was an isomer of **1**. The 1D NMR spectra of **2** (Tables 1 and 2) exhibited most of the structural features found in **1**, especially those for a cubebane moiety and a 3,5-diformylbenzyl phloroglucinol unit, indicating that **2** is comprised of the same coupling units as **1**, with differences being due to the coupling patterns. Detailed 2D NMR analysis (^1^H–^1^H COSY, HMBC, and NOESY) (Fig. 2) further confirmed this fact. The benzyl methine (CH-1′) of the 3,5-diformylbenzyl phloroglucinol moeity was first established by HMBC correlations of H-1′/C-9′ (13′), C-3′, and C-7′ and H-9′(13′)/C-1′. Then a methine from the cubebane moiety which showed the ^1^H–^1^H COSY correlation with a methylene (CH_2_-2) and the HMBC correlation with a tertiary methyl (CH_3_-15) was assigned as CH-3. The cubebane and phloroglucinol moieties were connected by ^1^H–^1^H COSY correlation of H-1′/H-3, revealing a C-3–C-1′ coupling pattern in **2**. The presence of the oxygen bridge between C-4 and C-3′ was determined using the same way as described for **1**. Therefore, the planar structure of **2** was established as shown in Fig. 2, which represents another new skeleton of cubebane-based *Psidium* meroterpenoid with a C-1′–C-3 linkage rather than the C-1′–C-15 linkage in **1**.

**Table 2:** ^13^C NMR (100 Hz) data of compounds **1**–**4** in CDCl_3_ (*δ* in ppm).

Position	1	2	3	4
1	34. 6, C	32.9, C	138.2, C	53.2, CH
2	28.4, CH_2_	38.7, CH_2_	85.5, CH	22.0, CH_2_
3	33.5, CH_2_	44.3, CH	40.1, CH_2_	37.5, CH_2_
4	89.8, C	90.6, C	45.1, C	84.6, C
5	33.4, CH	40.8, CH	47.3, CH	33.2, CH
6	23.4, CH	27.0, CH	32.9, CH	33.5, CH_2_
7	44.3, CH	44.6, CH	25.1, CH	35.0, CH_2_
8	27.1, CH_2_	26.8, CH_2_	21.4, CH_2_	152.0, C
9	31.6, CH_2_	31.3, CH_2_	36.6, CH_2_	41.5, CH
10	30.5, CH	29.5, CH	136.0, C	36.4, CH_2_
11	33.6, CH	33.6, CH	18.8, C	33.7, C
12	19.6, CH_3_	19.9, CH_3_	28.2, CH_3_	22.1, CH_3_
13	20.2, CH_3_	20.0, CH_3_	15.9, CH_3_	30.2, CH_3_
14	19.0, CH_3_	18.8, CH_3_	22.1, CH_3_	20.4, CH_3_
15	42.2, CH_2_	23.8, CH_3_	22.9, CH_3_	110.3, CH_2_
1′	35.1, CH	35.2, CH	53.7, CH	200.3, C
2′	103.5, C	99.9, C	113.8, C	103.7, C
3′	165.8, C	163.4, C	167.4, C	162.2, C
4′	104.2, C	103.8, C	107.6, C	100.9, C
5′	168.5, C	168.7, C	167.8, C	167.3, C
6′	104.2, C	103.7, C	105.2, C	104.2, C
7′	169.8, C	169.7, C	170.0, C	168.9, C
8′	144.6, C	142.3, C	140.7, C	142.5, C
9′/13′	126.7, CH	127.3, CH	130.5, CH	127.7, CH
10′/12′	128.6, CH	128.5, CH	127.7, CH	126.7, CH
11′	126.3, CH	126.5, CH	126.4, CH	130.2, CH
14′	192.2, CH	192.1, CH	193.6, CH	24.3, CH_2_
15′	191.6, CH	191.4, CH	191.9, CH	192.1, CH

**Figure 5:**
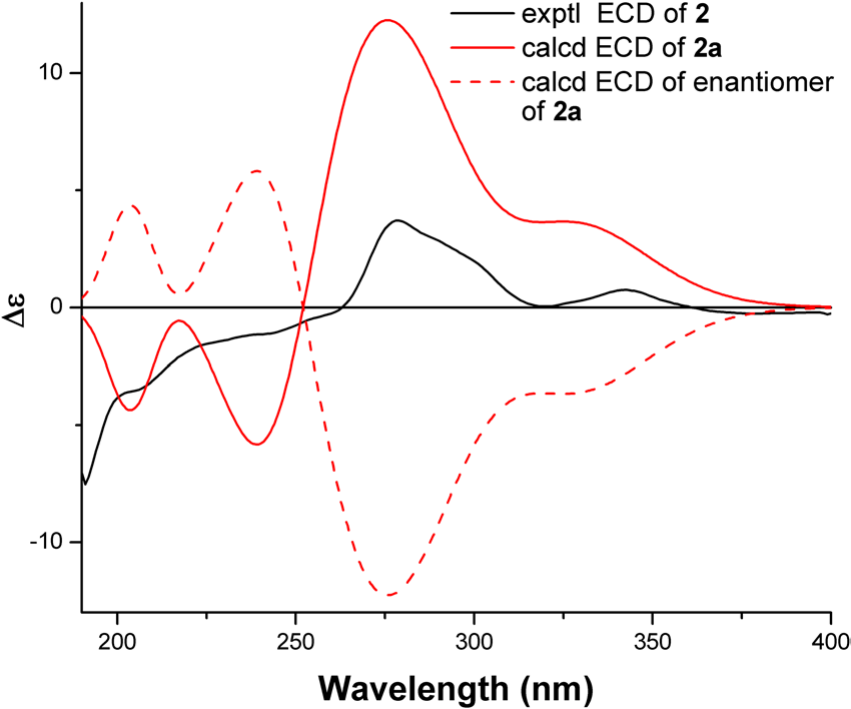
Experimental ECD spectrum of **2** and TDDFT calculated ECD spectra for **2a** (1*R*, 3*S*, 4*R*, 5*R*, 6*R*, 7*S*, 10*R*, 1′*R*) and enantiomer of **2a**.

The relative configuration of **2** was established by NOESY analysis. The key NOE correlations (Fig. 3) of H-5/H-7, H-9*α*, and H_3_-14 suggested that H-5, H-7, H-9*α*, and the H_3_-14 were cofacial and were arbitrarily assigned the *α*-orientation. Thus, the *β*-orientation of H-3, H-6, CH_3_-15, and the phenyl group were established by the NOE correlations of H-6/H-11, H-3, and H-2*β*, and H_3_-15/H-3 and H-9′ (13′). The small coupling constant (br. s) between H-5 and H-6 further confirmed the *trans*-relationship of these protons on the cyclopropane ring^33^. The absolute configuration of **2** was deduced to be 1*R*, 3*S*, 4*R*, 5*R*, 6*R*, 7*S*, 10*R*, 1′*R* by comparison of its experimental ECD spectrum with those of the calculated for the isomers (Fig. 5).

**Figure 6:**
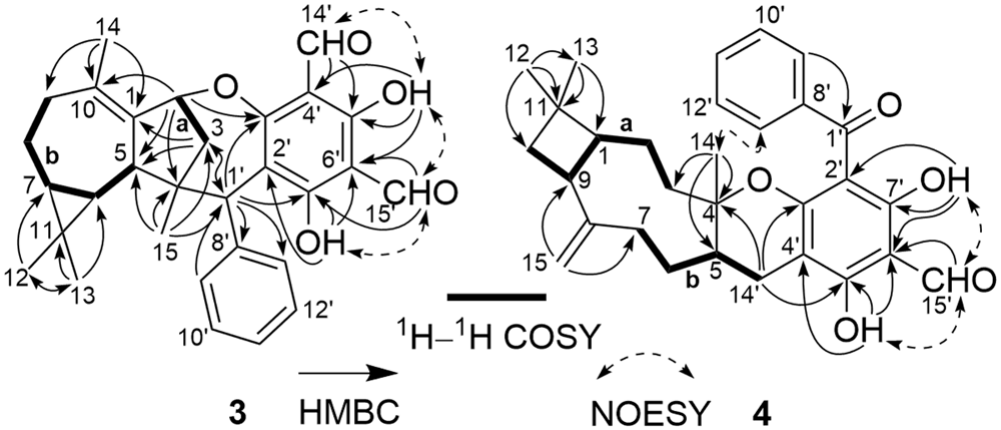
Selected ^1^H–^1^H COSY, HMBC, and NOESY correlations of **3** and **4**.

**Figure 7:**
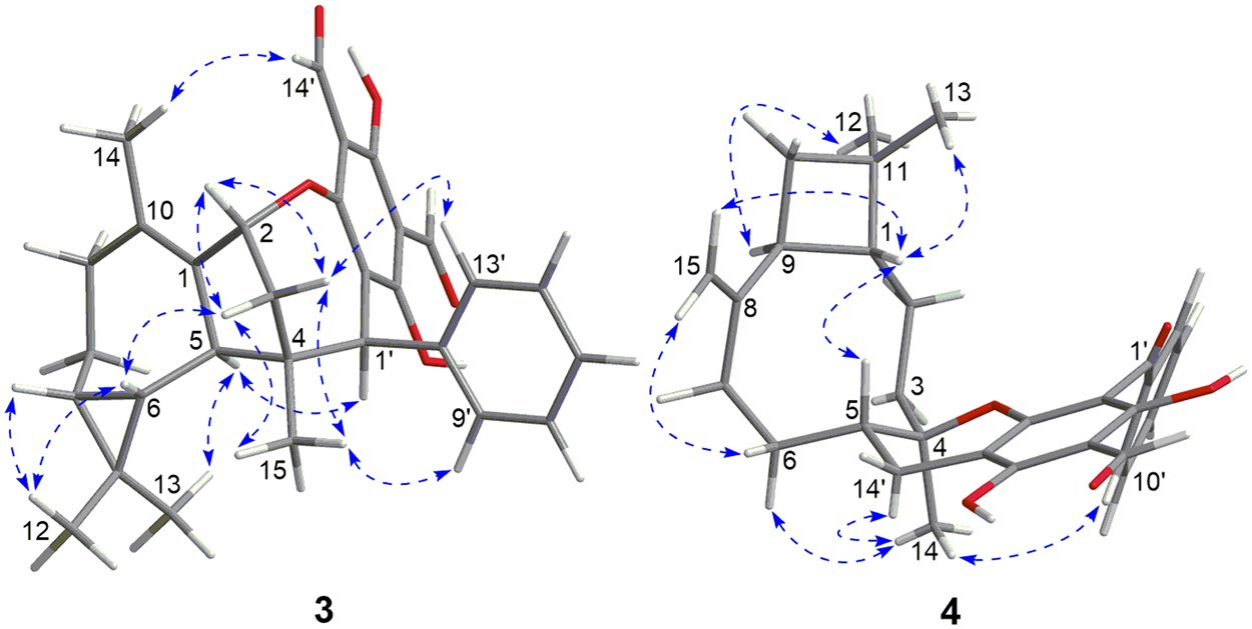
Selected NOESY correlations of **3** and **4**.

**Figure 8:**
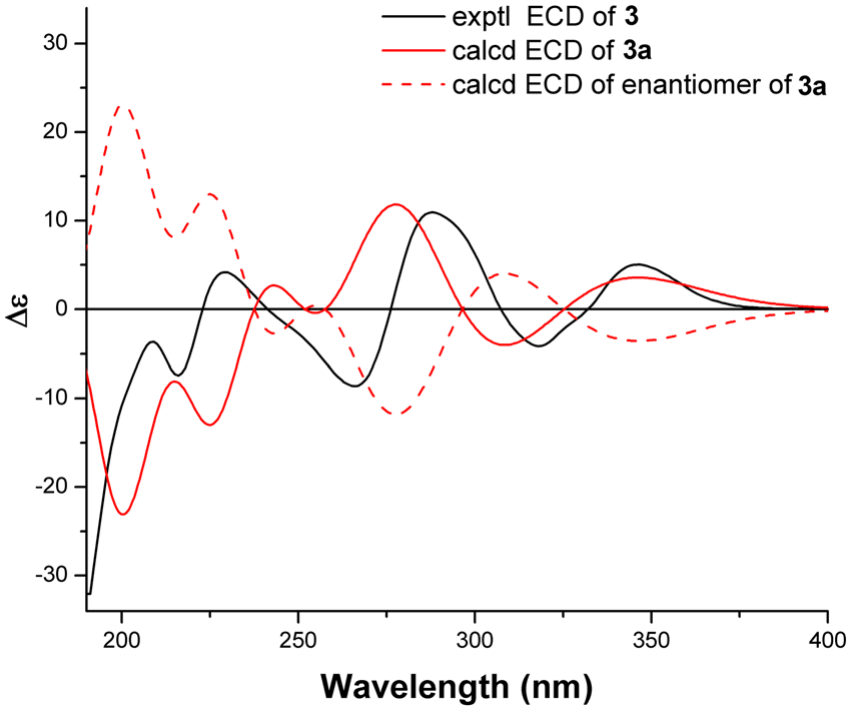
Experimental ECD spectrum of **3** and TDDFT calculated ECD spectra for **3a** (2*R*, 4*S*, 5*R*, 6*R*, 7*R*, 1′*R*) and enantiomer of **3a**.

Psiguajadial C (**3**) was assigned the molecular formula C_30_H_32_O_5_ based on the HR-ESI-MS and NMR data. The 1D NMR spectra of **3** (Tables 1 and 2) exhibited most of the structural features found in psiguadial A (**16**)^9^, a known globulane-based *Psidium* meroterpenoid featuring a rare oxocane ring junction, except that a methylene (*δ*_C_ 36.6, C-2), an oxygenated quaternary carbon (*δ*_C_ 104.1, C-1) and an upfielded methine (*δ*_C_ 39.9, C-10) in **16** were replaced by a oxygenated methine (*δ*_C_ 85.5, C-2) and a tetra-subsitituted double bond (*δ*_C_ 138.2 and 136.0, C-1 and C-10) in **3**. This indicated that the oxygen bridge started from C-1 in **16** was migrated to C-2 with the formation of Δ^1(10)^ in **3**, which was further confirmed by the HMBC correlations (Fig. 6) from H_3_-14 to C-1, C-9, and C-10 and from H-2 to C-3′, C-1, and C-10. The relative configuration of **3** was assigned by the NOE analysis (Fig. 7). The NOE correlations of H-3b/H-2, H-6, and H_3_-15, H-3a/H-2, H_3_-15, and H-13′ (9′), H_3_-12/H-6 and H-7, and H_3_-15/H-9′ (13′) indicated that H-2, H-6, H-7, CH_3_-12, CH_3_-15, the phenyl group, and the C-2–C-3–C-4 bridge were cofacial and were arbitrarily assigned *β*-orientations. Thus, the *α*-orientation of H-5, H_3_-13, and H-1′ was deduced by the NOE correlations of H-5/H_3_-13 and H-1′. The absolute configuration of **3** was established as 2*R*, 4*S*, 5*R*, 6*R*, 7*R*, 1′*R* by comparison of its experimental ECD spectrum with those of the calculated isomers (Fig. 8). Compound **3** represents the first example of *Psidium* meroterpenoid coupling *via* an oxepane ring.

**Figure 9:**
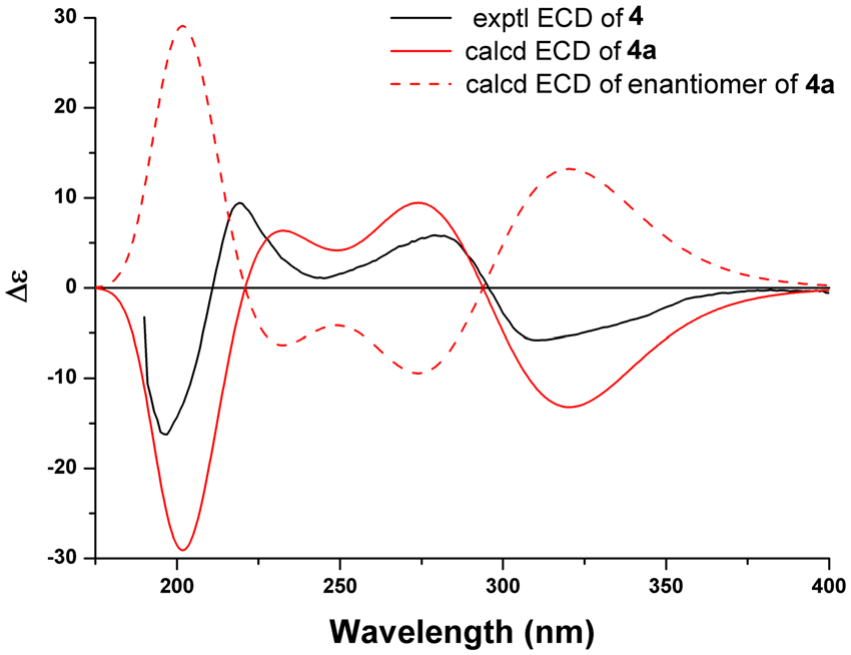
Experimental ECD spectrum of **4** and TDDFT calculated ECD spectra for **4a** (1*R*, 4*R*, 5*S*, 9*S*) and enantiomer of **4a**.

Psiguajadial D (**4**) possessed the molecular formula C_30_H_34_O_5_ as determined by its HR-ESI-MS and 1D NMR data. The ^1^H and ^13^C NMR data of **4** bore a high resemblance to those of guapsidial A (**17**)^11^, with the major differences occurring at C-3′, C-7′, and C-8′ of the benzoyl-phloroglucinol moiety (Δ*δ*_C_ = 2.3, 1.9, and 1.6 ppm, respectively), indicating that **4** possessed a different substitution pattern at the phloroglucinol unit. The locations of 5′-OH, 7′-OH, and 15′-CHO were assigned by HMBC correlations from the corresponding protons to C-5′, C-7′, and C-6′, respectively (Fig. 6). Consequently, the benzoyl group was located at the only remaining position on the phloroglucinol ring (C-2′). This was further supported by the NOE cross-peaks of H-15′/5′-OH and 7′-OH (Fig. 6). The absolute configuration of **4** (1*R*, 4*R*, 5*S*, 9*S*) was determined to be the same as that of **17** by comparison of their ^13^C NMR, NOESY, and ECD spectra, as well as TDDFT method (Fig. 9). *Psidium* terpenoids usually possess a C-1′ coupling site on the 3,5-diformyl-benzyl phloroglucinol part^6–10^. Psiguajadial D (**4**) with the C-14′ coupling site represented the second example of this compound category, while the first example, guapsidial A (**17**), was reported recently from the same species^11^.

**Figure 10:**
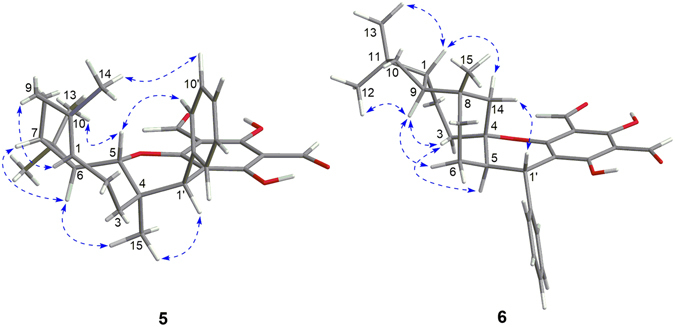
Selected NOESY correlations of **5** and **6**.

Psiguajadial E (**5**) was assigned the molecular formula of C_30_H_33_O_5_ by HR-ESI-MS, suggesting that it was an isomer of psiguadial D (**13**)^10^. The 1D (Tables 3 and 4) and 2D NMR analysis (see Supplementary Fig. S1.1) revealed that **5** possessed the same gross structure as that of **13**, with main differences being observed at C-3 (*δ*_C_ 40.1 in **5**, 35.3 in **13**), C-5 (*δ*_C_ 80.1 in **5**, 85.2 in **13**), C-15 (*δ*_C_ 26.0 in **5**, 18.6 in **13**), and C-1′(*δ*_C_ 48.6 in **5**, 43.9 in **13**), indicating that the configuration of the coupling sites in **5** was varied. In the NOESY spectrum of **5** (Fig. 10), the key cross-peaks of H-9′(13′)/H-5, H-10′(12′)/H_3_-14, and H-1′/H_3_-15 suggested that H-1′ and H_3_-15 were *α*-oriented while H-5 was *β*-oriented. Thus, **5** was assigned as the C-1′ epimer of **13**. This was further confirmed by comparison of the ECD spectra of **5** (see Supplementary S7.57) and **13**, which showed the reversed Cotton effects at 344 and 281 nm, as the Cotton effects arising from 3,5-diformyl-benzyl phloroglucinol moiety (UV *λ*_max_ at 340 and 279 nm) was dominated by the configuration of the chiral carbon C-1′. Thus, the structure of compound **5** was deduced as depicted.

**Table 3:** ^1^H NMR (400 Hz) data of compounds **5**–**10** in CDCl_3_ (*δ* in ppm, *J* in Hz).

Position	5	6	7	8	9	10
1	4.90, dd (12.3, 3.5)	1.75, m	1.57, m	2.36, m	2.35, m	2.21, m
2	*α* 1.55, m; *β* 1.24, m	*α* 1.64, m; *β* 1.31, m	*β* 1.51, m; *α* 1.32, m	a 1.50, m; b 1.34, m	*β* 1.85, m; *α* 1.44, m	*β* 1.53, m; *α* 1.21, m
3	a 2.01, m; b 1.80, m	*α* 1.95, m; *β* 1.80, m	a 2.12, dd (15.7, 10.0); b 1.76, m	1.82, m	*β* 1.79, m; *α* 1.67, m	1.89, m
5	4.45, d (7.1)	2.10, m	2.51, m	5.31, s	5.58, s	5.67, d (1.6)
6	0.93, t (7.8)	*α* 1.91, m; *β* 1.72, m	a 1.89, m; b 1.28, m	—	—	—
7	0.57, m	*β* 1.46, m; *α* 1.38, m	*β* 2.52, m; *α* 2.31, dd (13.6, 9.9)	1.49, m	1.62, m	1.67, m
8	*α* 1.90, m; *β* 1.00, m	—	—	*α* 1.80, m; *β* 1.61, m	*α* 1.70, m; *β* 1.61, m	1.65, m
9	*β* 1.95, m; *α* 1.88, m	2.16, m	2.63, dd (18.1, 9.1)	*β* 1.78, m; *α* 1.30, m	*β* 1.85, m; *α* 1.33, m	*β* 1.67, m; *α* 1.33, m
10	—	*α* 1.51, m; *β* 1.32, m	1.73, m	1.94, m	1.94, m	1.83, m
11	—	—	—	1.81, m	1.81, m	1.74, m
12	*α* 1.17, s	*α* 0.97, s	*α* 0.96, s	0.78, d (6.5)	0.65, d (6.6)	0.75, d (6.1)
13	*β* 1.23, s	*β* 0.99, s	*β* 0.99, s	0.90, d (6.4)	0.89, d (6.9)	0.91, d (6.0)
14	1.08, s	a 1.59, d (12.8); b 1.39, d (12.8)	1.05, s	0.82, d (7.0)	0.85, d (7.2)	0.86, d (6.7)
15	1.13, s	0.84, s	4.89, s 4.81, s	*β* 2.23, dd (14.2, 7.0); *α* 2.13, dd (14.2, 9.4)	*α* 2.32, dd (14.0, 6.9); *β* 2.02, dd (14.0, 9.8)	1.34, s
1′	3.78, s	3.76, d (8.1)	4.34, d (6.1)	4.08, t (8.1)	4.17, t (8.5)	3.49, d (11.6)
9′/13′	7.33, m	7.13, d (7.0)	7.11, d (7.4)	7.15, d (7.3)	7.16, d (7.5)	7.13, d (7.0)
10′/12′	7.25, m	7.26, t (7.0)	7.26, m	7.27, t (7.0)	7.29, t (7.0)	7.28, t (7.0)
11′	7.15, t (7.0)	7.29, t (7.0)	7.22, m	7.20, t (7.0)	7.21, t (7.0)	7.22, t (7.0)
14′	10.19, s	10.09, s	10.10, s	10.04, s	10.04, s	10.04, s
15′	10.07, s	10.08, s	10.15, s	10.13, s	10.13, s	10.08, s
5′-OH	13.74, s	13.48, s	13.63, s	13.53, s	13.51, s	13.54, s
7′-OH	13.05, s	13.11, s	13.20, s	13.18, s	13.18, s	13.07, s

**Table 4:** ^13^C NMR (100 Hz) data of compounds **5**–**10** in CDCl_3_ (*δ* in ppm).

Position	5	6	7	8	9	10
1	127.1, CH	44.3, CH	57.7, CH	36.1, CH	36.5, CH	38.4, CH
2	24.3, CH_2_	20.5, CH_2_	23.2, CH_2_	22.9, CH_2_	22.8, CH_2_	26.1, CH_2_
3	40.1, CH_2_	37.8, CH_2_	42.3, CH_2_	30.6, CH_2_	33.7, CH_2_	45.9, CH
4	40.9, C	85.0, C	86.1, C	78.9, C	78.9, C	80.2, C
5	80.1, CH	43.4, CH	39.0, CH	125.5, CH	122.8, CH	127.8, CH
6	28.5, CH	23.3, CH_2_	27.7, CH_2_	145.5, C	146.9, C	141.9, C
7	32.8, CH	32.6, CH_2_	36.1, CH_2_	51.1, CH	51.2, CH	50.0, CH
8	23.3, CH_2_	33.3, C	154.2, C	22.7, CH_2_	22.7, CH_2_	22.3, CH_2_
9	37.7, CH_2_	38.4, CH	42.6, CH	29.0, CH_2_	29.0, CH_2_	28.7, CH_2_
10	131.3, C	35.5, CH_2_	37.6, CH_2_	34.9, CH	34.9, CH	32.6, CH
11	19.4, C	34.7, C	33.5, C	26.5, CH	26.6, CH	26.6, CH
12	30.5, CH_3_	20.9, CH_3_	22.6, CH_3_	21.4, CH_3_	21.3, CH_3_	21.8, CH_3_
13	19.9, CH_3_	30.6, CH_3_	29.6, CH_3_	21.1, CH_3_	21.2, CH_3_	21.2, CH_3_
14	18.0, CH_3_	41.9, CH_2_	22.9, CH_3_	14.4, CH_3_	14.3, CH_3_	13.9, CH_3_
15	26.0, CH_3_	26.5, CH_3_	110.3, CH_2_	42.3, CH_2_	42.8, CH_2_	19.7, CH_3_
1′	48.6, CH	37.9, CH	35.8, CH	34.3, CH	34.3, CH	41.0, CH
2′	105.7, C	103.2, C	103.7, C	102.8, C	103.3, C	105.3, C
3′	163.0, C	162.8, C	164.2, C	164.5, C	164.7, C	164.1, C
4′	103.6, C	104.0, C	103.8, C	104.5, C	104.6, C	104.5, C
5′	168.6, C	168.4, C	168.75, C	168.6, C	168.5, C	168.5, C
6′	104.0, C	104.0, C	103.6, C	104.0, C	104.1, C	104.2, C
7′	168.8, C	169.6, C	168.83, C	169.7, C	169.7, C	169.7, C
8′	142.9, C	145.0, C	139.2, C	144.3, C	144.6, C	143.3, C
9′/13′	128.6, CH	127.9, CH	130.2, CH	126.9, CH	126.8, CH	127.8, CH
10′/12′	126.9, CH	128.3, CH	127.8, CH	128.4, CH	128.5, CH	128.2, CH
11′	126.7, CH	126.3, CH	126.7, CH	126.1, CH	126.2, CH	126.3, CH
14′	192.7, CH	191.5, CH	192.2, CH	192.3, CH	192.2, CH	192.4, CH
15′	191.4, CH	192.2, CH	192.5, CH	191.5, CH	191.6, CH	191.5, CH

Psiguajadial F (**6**) displayed a *quasi*-molecular ion peak at *m*/*z* 473.2353 [M – H]^−^ (calcd 473.2333) in HR-ESI-MS, in agreement with the molecular formula C_30_H_34_O_5_. The 1D and 2D NMR analysis revealed that **6** shared the same gross structure with psiguadial B^9^, a known caryolane-based *Psidium* monoterpenoid. Thus, the structural differences between the two compounds were attributable to the configurational variations on the coupling sites. The relative configuration of the caryolane moiety in **6** (C-1, C-4, C-8, and C-9) was assigned to be the same as those in psiguadial B by NOESY analysis (Fig. 10) and comparison of their 1D NMR data. Consequently, the NOE correlations of H-3*α*/H-5 and H-9 and H-1′/H-14b indicated that H-5 and H-1′ were *α*- and *β*- oriented in **6**, respectively, which were reversed as in psiguadial B. The absolute configuration of **6** was determined by comparison of its ECD spectrum (see Supplementary S7.58) with that of psiguadial B, in which the reversed Cotton effects at 344 and 284 nm indicated that the configuration of C-1′ in **6** was *S*. Thus, **6** was assigned as depicted.

**Figure 11:**
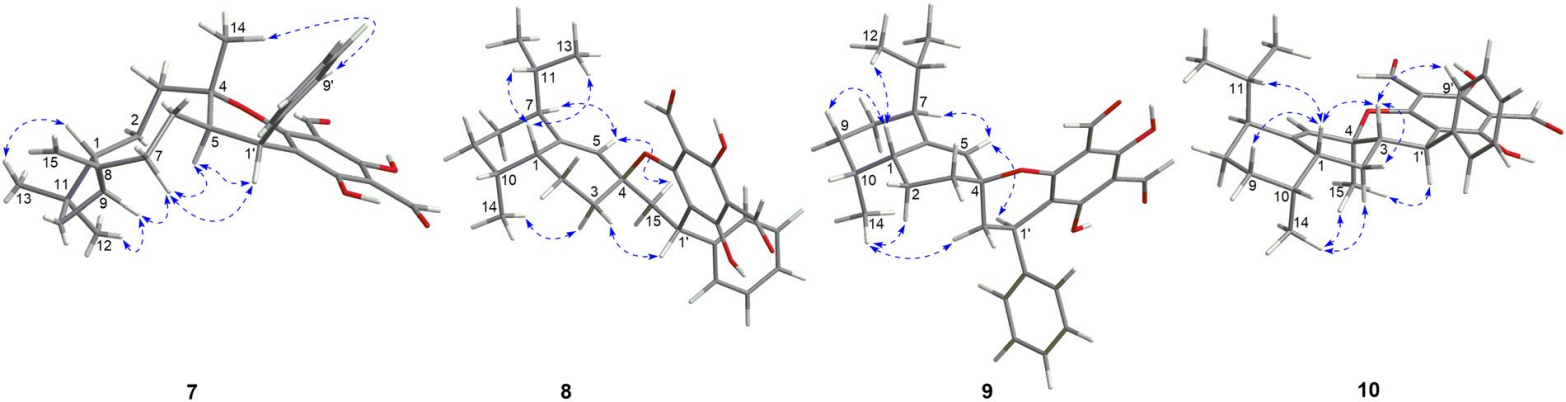
Selected NOESY correlations of **7**–**10**.

Psiguajadial G (**7**) had the molecular formula C_30_H_34_O_5_ as determined by HR-ESI-MS. Its 1D NMR data (Tables 3 and 4) were very similar to those of the synthesized caryophyllane-based meroterpenoid, compound **20** ^27^, which was isolated as a natural product for the first time in the current study and was given a trivial psiguajadial L. The structural difference between **7** and **20** was being due to the configuration of H-1′, which was designated as *α*-orientation in **7** by NOE correlations of H_3_-14/H-9′ (13′) and H-1′/H-5 and H-7*α* (Fig. 11). Thus, psiguajadial G (**7**) was assigned as the C-1′ epimer of **20**. The absolute configurations of psiguajadials G (**7**) and L (**20**) were established by analysis of their ECD spectra (see Supplementary S7.59 and S7.67), in which the Cotton effects around at 345 nm (positive for **7** and negative for **20**) and 284 nm (positive for **7** and negative for **20**) indicated that the C-1′ configuration of **7** and **20** were *R* and *S*, respectively. These assignments were in agreement with the ECD tendencies of those for guajadial (**19**) and psidial A (**18**)^9, 27^, respectively.

Psiguajadials H (**8**) and I (**9**) had the identical molecular formula C_30_H_34_O_5_ on the basis of their HR-ESI-MS data. Analysis of their 1D and 2D NMR data indicated that both compounds had the same gross structure as the known cadinane-based *Pisudium* meroterpenoids guajadials C (**21**) and D (**22**)^34^, a pair of C-1′ epimers also isolated in the current study. The relative configurations of the cadinane moiety (C-1, C-7, and C-10) in **8** and **9** were established as the same as those in **21** and **22** based on the NOESY analysis and comparison of their 1D NMR data. Thus, the remaining two chiral centers (C-4 and C-1′) formed by the spiro ring junction suggested that **8**, **9**, **21**, and **22** were four stereoisomers. Detailed NOESY analysis of **8** and **9** (Fig. 11) indicated that they had the same configuration at the spirocenter (C-4–C-15 bond *α*-oriented) but different configuration at C-1′ (H-1′*β* in **8** and H-1′*α* in **9**). Thus, compounds **8** and **9** were also a pair of C-1′ epimers. The absolute configurations of **8** and **9** were established by analysis of their ECD spectra (see Supplementary S7.60 and S7.61), in which the Cotton effects at 344 nm (negative for **8** and positive for **9**) and 284 nm (negative for **8** and positive for **9**) indicated that the C-1′ configuration of **8** and **9** were *S* and *R*, respectively. These assignments were in agreement with the ECD tendencies of those for **21** and **22**, respectively.

Psiguajadial J (**10**) had the molecular formula C_30_H_34_O_5_ as determined on the basis of HR-ESI-MS and NMR data. The 1D and 2D NMR analysis revealed that **10** shared the same gross structure with those of guajadials E (**23**) and F (**24**)^34^, a pair of C-1′ epimers of cadinane-based *Psidium* meroterpenoid, indicating that they were stereoisomers with configurational variations occurring at the coupling sites (C-1′, C-3, and C-4). The relative configuration of **10** was established by NOESY experiment and analysis of its coupling constant. The NOE correlations of H-1/H-3, H-9*β*, and H-11 and H-3/H-9′ (13′), and H-2*β* indicated that these protons and the benzene ring were cofacial and were arbitrarily assigned as *β*-orientated. Thus, the NOE correlations of H_3_-15/H-1′, H-2*α*, and H_3_-14 (Fig. 11), assigned these protons or methyl group in *α*-orientation. The *trans*-relationship of H-1′ and H-3 were further confirmed by the large coupling constant of H-1′ (*J* = 11.6 Hz). Thus, **10** was established as a C-3 epimer of **24**, with a *trans*-fused dihydropyran ring. The *R*-configuration of C-1′ in **10** was established by the Cotton effects at 346 (positive) and 278 nm (positive) in its ECD spectrum (see Supplementary S7.62), which showed the similar tendency to those of **24**.

**Figure 12:**
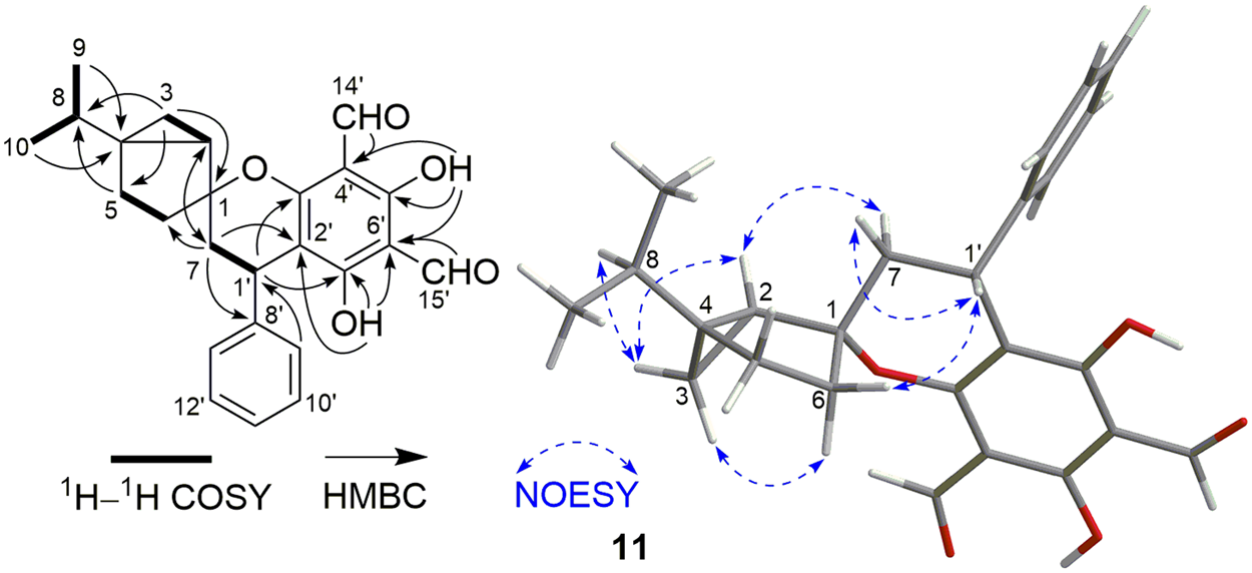
Selected ^1^H–^1^H COSY, HMBC, and NOESY correlations of **11**.

Psiguajadial K (**11**) had a molecular formula of C_25_H_25_O_5_ as determined by its HR-ESI-MS. The 1D (Table 5) and 2D NMR analysis (Fig. 12) revealed that **11** possessed the same gross structure as that of guadial A (**26**)^10^, indicating that they were stereoisomers with configurational variations occurring at the coupling sites. In the NOESY spectrum, the correlations of H-2/H-7*β* and H-1′/H-6*β* and H-7*α* (Fig. 12) indicated that C-1–C-7 bond was *β*-oriented at the spirocenter while H-1′ was *α*-oriented on the pyran ring. Thus, **11** was established as the C-1′ epimer of guadial A. This was further confirmed by the ECD spectrum of **11**, which showed the reversed Cotton effects at 341 (positive) and 278 nm (positive) as compared to those of **26**.

**Table 5:** ^1^H (400 Hz) and ^13^C NMR (100 Hz) data of compound **11** in CDCl_3_ (*δ* in ppm).

Position	**11**	
	*δ*_H_, multi. (*J* in Hz)	*δ*_C_, type
1	—	88.7, C
2	1.06, dd (7.8, 3.5)	30.0, CH
3	*α* 0.81, dd (5.2, 3.5); *β* 0.41, dd (7.8, 5.2)	11.6, CH_2_
4	—	33.6, C
5	1.71, m	25.1, CH_2_
6	*β* 1.84, m; *α* 1.50, m	32.1, CH_2_
7	*β* 2.30, dd (14.3, 7.4); *α* 2.23, ddd (14.3, 7.6, 1.0)	40.5, CH_2_
8	1.21, t (6.9)	32.1, CH
9	0.76, d (6.8)	19.3, CH_3_
10	0.73, d (6.9)	19.3, CH_3_
1′	4.15, t (7.5)	34.3, CH
2′	—	102.6, C
3′	—	165.3, C
4′	—	104.4, C
5′	—	168.5, C
6′	—	104.0, C
7′	—	169.8, C
8′	—	144.2, C
9′/13′	7.18, d (7.0)	127.0, CH
10′/12′	7.28, t (7.0)	128.5, CH
11′	7.20, t (7.0)	126.3, CH
14′	10.13, s	191.6, CH
15′	10.12, s	192.4, CH
5′-OH	13.56, s	
7′-OH	13.19, s

The spectroscopic data of compound **12** was identical to guajavadial C, a new skeleton of bicyclogermacrane-based *Psidium* meroterpenoid recently isolated from the same plant by Qin *et al*.^35^. However, after careful analysis of its NMR data, we found the configuration of guajavadial C was incorrect (see Supplementary Fig. S1.2). The *cis*-relationship of H-5 and H-1′ was assigned by the author only based on the NOE correlation of H-5/H-1′. In fact, on a six-mumbered ring both *cis* and *trans* adjacent protons could generate NOE correlations. In this case, the *trans*-relationship of H_3_-15 and H-5 was firstly assigned by the NOE correlations of H-5/H_3_-13 and H-3*β*, and H-6/H_3_-15 (see Supplementary Fig. S1.2). Then the NOE correlations of H-9′(13′)/H-3*β* further assigned the benzene group in *β*-orientation. Thus, H-1′ was *α*-oriented and in the *trans*-position of H-5. In additon, the congfiguration of Δ^1(10)^ was revised as *E* by the observed NOE correlation of H-1/H-9*β*. The absolute configuration of **12** was established as 4*S*, 5*R*, 6*R*, 7*R*, 1′*S* by comparison of its experimental ECD spectrum with those of calculated for its isomers (see Supplementary Fig. S1.3).

(±)-Guajadial B^8^, a racemic *Psidium* meroterpenoid previously isolated from the same plant, was chirally separated in the current study to afford a pair of enantiomers, (−)-guajadial B (**14**) ([*α*]^26^_D_ −30.0) and (+)-guajadial B (**15**) ([*α*]^26^_D_ +30.0). The absolute configurations of **14** (3*R*, 4*S*, 1′*S*) and **15** (3*S*, 4*R*, 1′*R*) were established by analysis of their ECD spectra (see Supplementary S7.65 and S7.66), in which the Cotton effects at 348 and 290 nm were correlated with the configuration of C-1′ as described for the aforementioned *Psidium* meroterpenoids.

The known compounds psiguadial D (**13**)^10^, psiguadial A (**16**)^9^, guapsidial A (**17**)^11^, psidial A (**18**), guajadial (**19**)^9, 27^, psiguajadial L (**20**)^27^, guajadials C–F (**21–24**)^34^, guajavadial A (**25**)^35^, guadial A (**26**)^10^, guadial B (**27**)^11^, and guadial C (**28**)^11^ were identified by comparison of their NMR (see Supplementary Tables S2.23–S2.7) and optical rotation data with those in the literature. In addition, the absolute configurations of psiguajadial L (**20**) and guajadials C–F (**21–24**) were established for the first time based on ECD analysis (see Supplementary S7.67–S7.71).

**Figure 13:**
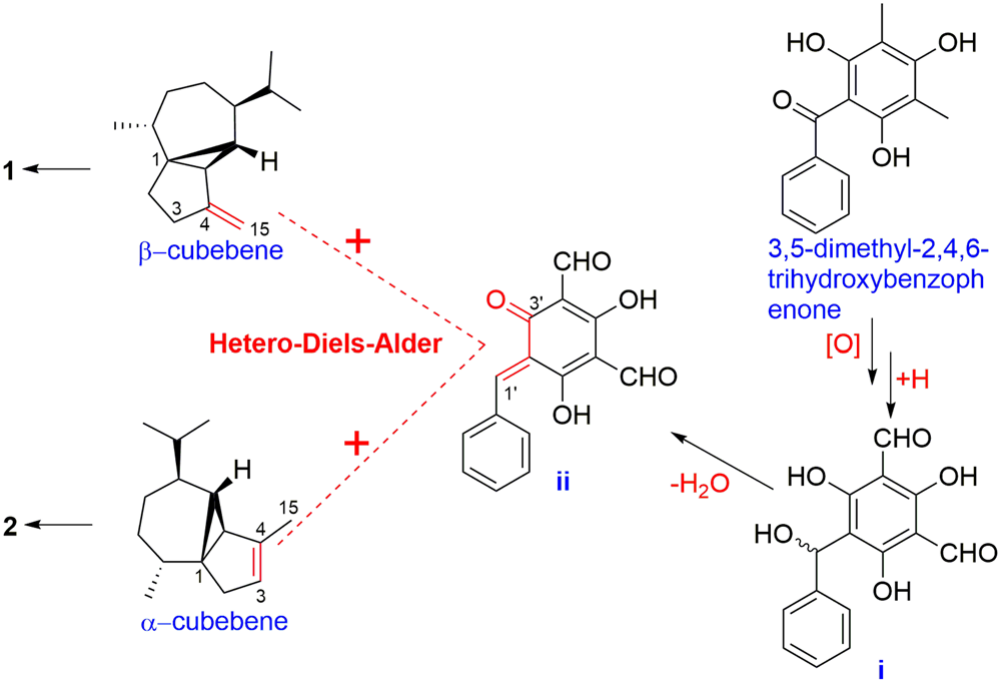
A plausible biosynthetic pathway of **1** and **2**.

The biosynthesis of *Psidium* meroterpenoids was previously hypothesized to occur *via* hetero-Diels-Alder reactions or a series of electrophilic addition reactions between the mono- or sesquiterpenoid precursors and the key intermediate 3,5-dimethyl-2,4,6-trihydroxybenzophenon^6, 10, 11, 36^. The hetero-Diels-Alder pathway was supported by the biomimetic synthesis of (±)-guajadial B, psidial A (**18**), guajadial (**19**), psiguajadial L (**20**), and psiguadial D (**13**)^8, 25, 27^. Thus, the biosynthetic pathways of the new skeleton compounds (**1** and **2**) in this study were proposed based on the hetero-Diels-Alder reaction as shown in Fig. 13. 3,5-Dimethyl-2,4,6-trihydroxybenzophenone, which has been previously isolated from the same plant^10, 37^, is presumed to be the biogenetic precursor of the benzyl phloroglucinol intermediates **i** ^9^. Dehydration of **i** further produced the *O*-quinone methide intermediate **ii**. *β*-Cubebene and *α*-cubebene, which have been reported to be present in the same species^10, 38, 39^, are deemed to be the sesquiterpenoid precursors. Reaction of the terpenoid precursors with **ii** *via* hetero-Diels-Alder reactions would afford **1** and **2**.

Compounds **1**–**28** were tested for their inhibitory activities against PDE4D2. Rolipram, a well-known PDE4 inhibitor, was used as the positive control (IC_50_ 0.62 ± 0.03 *μ*M). Most of compounds (**1**–**13** and **16**–**26**) exhibited moderate inhibitory activities with IC_50_ values in the range of 1.34–7.26 *μ*M (Table 6), which may explain the inhibitory activity of the selected fraction and the anti-inflammatory usage of *P. guajava* in traditional medicine.

**Table 6:** IC_50_ Values of compounds against PDE4D2.

Compound	IC_50_ (*μ*M)	Compound	IC_50_ (*μ*M)	Compound	IC_50_ (*μ*M)
**1**	3.11 ± 0.10	**11**	3.68 ± 0.27	**21**	2.28 ± 0.11
**2**	5.03 ± 0.33	**12**	4.33 ± 0.21	**22**	1.93 ± 0.11
**3**	4.50 ± 0.28	**13**	3.42 ± 0.21	**23**	2.73 ± 0.15
**4**	4.14 ± 0.26	**14**	>50	**24**	2.67 ± 0.17
**5**	3.25 ± 0.20	**15**	>50	**25**	2.01 ± 0.07
**6**	2.63 ± 0.13	**16**	7.26 ± 0.41	**26**	2.70 ± 0.15
**7**	1.34 ± 0.33	**17**	5.61 ± 0.51	**27**	>50
**8**	1.81 ± 0.09	**18**	1.60 ± 0.09	**28**	17.09 ± 0.96
**9**	2.51 ± 0.17	**19**	1.62 ± 0.08	**rolipram** ^**a**^	0.62 ± 0.03
**10**	2.53 ± 0.33	**20**	1.37 ± 0.07		

^a^Positive control.

*Psidium* meroterpenoids are a small group of natural products characterized by a 3,5-diformyl-benzyl phloroglucinol moiety coupled with a mono- or sesquiterpenoid unit. In the current study, bioassay-guided fractionation of the ethanolic extract of the leaves of *Psidium guajava* led to the isolation of 28 *Psidium* meroterpenoids, including 11 new ones (**1**–**11**). Compounds **1** and **2** represent two unprecedented skeletons with the presence of cubebane moiety in this compound class reported for the first time. Compound **3** represents the first example of *Psidium* meroterpenoid coupling *via* an oxepane ring, and **4** was the second example featuring the C-14′ coupling site. Compounds **1**–**13** and **16**–**26** exhibited moderate inhibitory activities against phosphodiesterase-4 (PDE4), with IC_50_ values in the range of 1.34–7.26 *μ*M, suggesting that this compound class represents a new class of PDE4 inhibitors, which may serve as new structural motifs for designing new PDE4 inhibitors. Investigations of their mechanism of action and selectivity *versus* other PDEs are ongoing.

## Methods

### General

Optical rotations were determined on a Perkin-Elmer 341 polarimeter at 20 °C. UV spectra were performed on a Shimadzu UV-2450 spectrophotometer, and ECD spectra were performed on an Applied Photophysics Chirascan spectrometer. Infrared spectra (IR) were measured on a Bruker Tensor 37 infrared spectrophotometer. NMR experiments were carried out on a Bruker AM-400 spectrometer at the temperature thermostatically controlled at 25 °C. Exact mass measurements and molecular formulas were obtained from ESIMS and HR-ESI-MS using a Finnigan LCQ Deca and a Thermo Scientific LTQ Orbitrap XL spectrometers, respectively. Semi-preparative reversed-phase (RP) HPLC was performed with a YMC-pack ODS-A column (10 × 250 mm, S-5 *μ*m) or a Phenomenex Lux cellulose-2 chiral column (10 × 250 mm, 5 *μ*m) under Shimadzu LC-20 AT equipped with a SPD-M20A PDA detector. Column chromatography (CC) was performed on RP-C_18_ silica gel (S-50 *μ*m, 12 nm, YMC Co., Ltd.), MCI gel (CHP20P, 75–150 *μ*m, Mitsubishi Chemical Industries Ltd.), Sephadex LH-20 gel (Amersham Biosciences), and silica gel (300–400 mesh, Qingdao Haiyang Chemical Co., Ltd.). For RP-HPLC and CC, the analytical grade solvents (Guangzhou Chemical Reagents Company, Ltd.) were employed.

### Plant material

*P. guajava* were collected in Guangzhou, Guangdong Province, China, in August 2014. The plant was identified by Dr. You-Kai Xu, Xishuangbanna Tropical Botanical Garden, Chinese Academy of Sciences, and voucher specimens (FSL201408) have been stored at the School of Pharmaceutical Sciences, Sun Yat-sen University.

### Extraction and isolation

Sequential extraction was carried out on 6.0 kg of the air-dried leaves of *P. guajava* with 95% EtOH immersion (10 L × 4) at rt. After evaporating the solvent, the residue (320 g) was suspended in H_2_O (1.5 L) and extracted with EtOAc (1.5 L × 3) and *n*-BuOH (1.5 L × 3), respectively. The EtOAc partition (165 g) was chromatographed over MCI gel CC (MeOH-H_2_O, 10%→100%) to give seven sub-fractions (A–G).

Each fraction was subjected to CC over RP-C_18_ column, silica gel, and Sephadex LH-20 and then further purified by semipreparative HPLC with a YMC-pack ODS-A column or a Phenomenex Lux chiral column to yield pure compounds. Compounds **11** (2 mg), **13** (256 mg), **19** (532 mg), **20** (222 mg), and **23** (433 mg) were obtained from Fr. B. Fr. D gave compounds **14** (23 mg) and **15** (24 mg). Fr. E afforded compounds **4** (14 mg), **5** (11 mg), **7** (14 mg), **8** (49 mg), **10** (17 mg), **17** (6 mg), **21** (113 mg), **22** (17 mg), **24** (72 mg), **26** (1 mg), and **28** (17 mg). Compounds **1** (14 mg), **2** (19 mg), **3** (5 mg), **6** (5 mg), **11** (9 mg), **12** (13 mg), **16** (8 mg), **18** (269 mg), **25** (26 mg), and **27** (8 mg) were obtained from Fr. F. The details on isolation of these compounds are provided in Supplementary S3.1.

*Psiguajadial A (* ***1*** *)*. Colourless oil; [*α*]^26^_D_ + 22 (*c* 0.3, CHCl_3_); UV (MeCN) *λ*_max_ (log *ε*) 280 (4.5) nm; ECD (*c* 1.1 × 10^−4^ M, MeCN); λ_max_ (Δ*ε*) 340 (+2.2), 283 (+11.6), 250 (−4.5), 212 (−15.7) nm; IR (KBr) *ν*_max_ 3448, 2957, 2924, 2851, 1633, 1439, 1302, 1181, 773 cm^−1^; ^1^H and ^13^C NMR data, see Tables 1 and 2; (−) MS-ESI *m/z* 473.3 [M – H]^−^; (−) HR-MS-ESI *m/z* 473.2304 [M – H]^−^ (calcd for C_30_H_33_O_5_, 473.2333).

*Psiguajadial B (* ***2*** *)*. Colourless oil; [*α*]^26^_D_ +7.1 (*c* 0.5, CHCl_3_); UV (MeCN) *λ*_max_ (log *ε*) 197 (4.4), 278 (4.6), 343 (3.6) nm; ECD (*c* 4.2 × 10^−4^ M, MeCN) λ_max_ (Δ*ε*) 342 (+0.8), 279 (+3.7), 191 (−7.5) nm; IR (KBr) *ν*_max_ 3448, 2955, 2925, 2869, 1633, 1443, 1381, 1305, 1179 cm^−1^; ^1^H and ^13^C NMR data, see Tables 1 and 2; (+) MS-ESI *m*/*z* 475.3 [M + H]^+^; (+) HR-MS-ESI *m*/*z* 475.2475 [M + H]^+^ (calcd for C_30_H_35_O_5_, 475.2479).

*Psiguajadial C (* ***3*** *)*. Colourless oil; [*α*]^26^_D_ +76.6 (*c* 0.2, CHCl_3_); UV (MeCN) *λ*_max_ (log *ε*) 198 (4.5), 275 (4.5), 347 (3.6) nm; ECD (*c* 4.2 × 10^−4^ M, MeCN) λ_max_ (Δ*ε*) 346 (+5.1), 318 (−4.2), 288 (+11.0), 266 (−8.6), 229 (+4.2), 216 (−7.4), 191 (−32.1) nm; IR (KBr) *ν*_max_ 3447, 2958, 2922, 2872, 1634, 1602, 1445, 1379, 1306, 1130 cm^−1^; ^1^H and ^13^C NMR data, see Tables 1 and 2; (−) MS-ESI *m*/*z* 471.2 [M – H]^−^; (−) HR-MS-ESI *m*/*z* 471.2191 [M – H]^−^ (calcd for C_30_H_31_O_5_, 471.2177).

*Psiguajadial D (* ***4*** *)*. Colourless oil; [*α*]^26^_D_ −149 (*c* 0.2, CHCl_3_); UV (MeCN) λ_max_ (log *ε*) 199 (4.5), 285 (4.5) nm; ECD (*c* 2.1 × 10^−4^ M, MeCN) λ_max_ (Δ*ε*) 308 (−5.7), 282 (+5.8), 218 (+9.2), 196 (−16.2) nm; IR (KBr) *ν*_max_ 3453, 2950, 2924, 2857, 1630, 1605, 1425, 1317, 1287, 697 cm^−1^; ^1^H and ^13^C NMR data, see Tables 1 and 2; (−) MS-ESI *m/z* 473.3 [M – H]^−^; (−) HR-MS-ESI *m/z* 473.2319 [M – H]^−^ (calcd for C_30_H_33_O_5_, 473.2333).

*Psiguajadial E (* ***5*** *)*. Colourless oil; [*α*]^26^_D_ −105.4 (*c* 0.3, CHCl_3_); UV (MeCN) *λ*_max_ (log *ε*) 199 (4.6), 279 (4.6), 340 (3.7) nm; ECD (*c* 4.2 × 10^−4^ M, MeCN) λ_max_ (Δ*ε*) 344 (+0.7), 304 (+1.8), 281 (+0.9), 265 (−3.6), 221 (−7.1), 190 (−21.3) nm; IR (KBr) *ν*_max_ 3437, 2955, 2922, 2854, 1628, 1439, 1382, 1300, 1267, 1181 cm^−1^; ^1^H and ^13^C NMR data, see Tables 3 and 4; (−) MS-ESI *m*/*z* 473.3 [M – H]^−^; (−) HR-MS-ESI *m*/*z* 473.2297 [M – H]^−^ (calcd for C_30_H_33_O_5_, 473.2333).

*Psiguajadial F (* ***6*** *)*. Colourless oil; [*α*]^26^_D_ −50.3 (*c* 0.2, CHCl_3_); UV (MeCN) *λ*_max_ (log *ε*) 198 (4.4), 278 (4.5), 343 (3.5) nm; ECD (*c* 4.2 × 10^−4^ M, MeCN) λ_max_ (Δ*ε*) 344 (−2.5), 284 (−13.5), 247 (+3.6), 206 (+20.0), 190 (+12.8) nm; IR (KBr) *ν*_max_ 3432, 2955, 2922, 2870, 1634, 1443, 1383, 1306, 1181 cm^−1^; ^1^H and ^13^C NMR data, see Tables 3 and 4; (−) MS-ESI *m*/*z* 473.2 [M − H]^−^; (−) HR-MS-ESI *m*/*z* 473.2353 [M − H]^−^ (calcd for C_30_H_33_O_5_, 473.2333).

*Psiguajadial G (* ***7*** *)*. Colourless oil; [*α*]^26^_D_ −61.7 (*c* 0.2, CHCl_3_); UV (MeCN) *λ*_max_ (log *ε*) 199 (4.6), 278 (4.6), 340 (3.7) nm; ECD (*c* 4.2 × 10^−4^ M, MeCN) λ_max_ (Δ*ε*) 345 (+1.1), 279 (+6.0), 254 (−2.1), 201 (−12.8), 190 (−11.5) nm; IR (KBr) *ν*_max_ 3466, 2952, 2925, 2856, 1630, 1439, 1382, 1302, 1182 cm^−1^; ^1^H and ^13^C NMR data, see Tables 3 and 4; (−) MS-ESI *m*/*z* 473.3 [M – H]^−^; (−) HR-MS-ESI *m*/*z* 473.2296 [M – H]^−^ (calcd for C_30_H_33_O_5_, 473.2333).

*Psiguajadial H (* ***8*** *)*. Colourless oil; [*α*]^26^_D_ −6.3 (*c* 0.4, CHCl_3_); UV (MeCN) *λ*_max_ (log *ε*) 198 (4.5), 278 (4.5), 342 (3.6) nm; ECD (*c* 4.2 × 10^−4^ M, MeCN) λ_max_ (Δ*ε*) 344 (−1.5), 328 (−1.5), 284 (−6.0), 259 (+4.1), 216 (+17.8), 191 (+10.2) nm; IR (KBr) *ν*_max_ 3431, 2956, 2924, 2871, 1635, 1441, 1381, 1303, 1182 cm^−1^; ^1^H and ^13^C NMR data, see Tables 4 and 5; (−) MS-ESI *m*/*z* 473.2 [M – H]^−^; (−) HR-MS-ESI *m*/*z* 473.2351 [M – H]^−^ (calcd for C_30_H_33_O_5_, 473.2333).

*Psiguajadial I (* ***9*** *)*. Colourless oil; [*α*]^26^_D_ −37.8 (*c* 0.4, CHCl_3_); UV (MeCN) *λ*_max_ (log *ε*) 197 (4.5), 278 (4.5), 342 (3.6) nm; ECD (*c* 4.2 × 10^−4^ M, MeCN) λ_max_ (Δ*ε*) 344 (+2.3), 284 (+7.8), 257 (−7.1), 215 (−23.2), 190 (−19.8) nm; IR (KBr) *ν*_max_ 3441, 2956, 2923, 2871, 1634, 1441, 1380, 1304, 1181 cm^−1^; ^1^H and ^13^C NMR data, see Tables 3 and 4; (+) MS-ESI *m*/*z* 475.3 [M + H]^+^, 497.2 [M + Na]^+^; (+) HR-MS-ESI *m*/*z* 475.2479 [M + H]^+^ (calcd for C_30_H_35_O_5_, 475.2479).

*Psiguajadial J (* ***10*** *)*. Colourless oil; [*α*]^26^_D_ +124.1 (*c* 0.6, CHCl_3_); UV (MeCN) *λ*_max_ (log *ε*) 198 (4.5), 278 (4.6), 342 (3.6) nm; ECD (*c* 4.2 × 10^−4^ M, MeCN) λ_max_ (Δ*ε*) 346 (+1.0), 278 (+14.5), 246 (−2.4), 226 (+2.4), 218 (−1.3), 212 (+1.4), 191 (−21.6) nm; IR (KBr) *ν*_max_ 3429, 2957, 2925, 2872, 1364, 1141, 1380, 1304, 1263, 1185, 1155 cm^−1^; ^1^H and ^13^C NMR data, see Tables 3 and 4; (+) MS-ESI *m*/*z* 475.3 [M + H]^+^, 497.2 [M + Na]^+^; (+) HR-MS-ESI *m*/*z* 475.2477 [M + H]^+^ (calcd for C_30_H_35_O_5_, 475.2479).

*Psiguajadial K (* ***11*** *)*. Colourless oil; [*α*]^26^_D_ +147.6 (*c* 0.1, CHCl_3_); UV (MeCN) *λ*_max_ (log *ε*) 230 (4.1), 280 (4.5), 342 (3.5) nm; ECD (*c* 1.2 × 10^−4^ M, MeCN) λ_max_ (Δ*ε*) 341 (+2.1), 278 (+20.6), 250 (−4.0), 230 (+2.3), 213 (−16.4), 190 (+17.6) nm; IR (KBr) *ν*_max_ 3446, 2957, 2924, 2851, 1635, 1559, 1440, 1381, 1302, 1182 cm^−1^; ^1^H and ^13^C NMR data, see Table 5; (−) MS-ESI *m*/*z* 405.4 [M – H]^−^; (−) HR-MS-ESI *m*/*z* 405.1688 [M – H]^−^ (calcd for C_25_H_25_O_5_, 405.1707).

*Guajavadial C (* ***12*** *)*. Colourless oil; [*α*]^26^_D_ −44.7 (*c* 0.4, CHCl_3_); UV (MeCN) *λ*_max_ (log *ε*) 197 (4.5), 275 (4.5), 346 (3.5) nm; ECD (*c* 4.2 × 10^−4^ M, MeCN) λ_max_ (Δ*ε*) 346 (−1.9), 314 (+3.4), 285 (−13.2), 251 (+3.0), 218 (+1.2), 209 (−0.3), 192 (+10.6) nm; IR (KBr) *ν*_max_ 3429, 2956, 2923, 2870, 1638, 1445, 1380, 1305, 1176 cm^−1^; ^1^H and ^13^C NMR data, see Supplementary Table S2.3; (−) MS-ESI *m*/*z* 473.2 [M – H]^−^; (−) HR-MS-ESI *m*/*z* 473.2356 [M – H]^−^ (calcd for C_30_H_33_O_5_, 473.2333).

*(−)-Guajadial B (* ***14*** *)*. [*α*]^26^_D_ −30.0 (*c* 0.1, CHCl_3_); ECD (*c* 5.5 × 10^−4^ M, MeCN) λ_max_ (Δ*ε*) 348 (−2.6), 334 (−0.5), 290 (−24.3), 260 (+7.2), 228 (−7.0), 216 (+25.6), 197 (+31.3) nm.

*(+)-Guajadial B (* ***15*** *)*. [*α*]^26^_D_ +30.0 (*c* 0.1, CHCl_3_); ECD (*c* 3.6 × 10^−4^ M, MeCN) λ_max_ (Δ*ε*) 348 (+3.7), 334 (+0.4), 289 (+35.1), 261 (−10.5), 228 (+12.2), 217 (−33.5), 190 (+25.6) nm.

*Psiguajadial N (* ***20*** *)*. ECD (*c* 4.6 × 10^−4^ M, MeCN) λ_max_ (Δ*ε*) 347 (−1.3), 284 (−19.7), 255 (+4.4), 228 (−3.0), 202 (+46.2) nm.

*Guajadial C (* ***21*** *)*. ECD (*c* 4.6 × 10^−4^ M, MeCN) λ_max_ (Δ*ε*) 345 (+1.4), 280 (+11.9), 252 (−2.3), 218 (−10.5), 207 (+27), 191 (−12.0) nm.

*Guajadial D (* ***22*** *)*. ECD (*c* 4.4 × 10^−4^ M, MeCN) λ_max_ (Δ*ε*) 340 (−1.1), 297 (−1.4), 261 (+3.9), 214 (+16.9) nm.

*Guajadial E (* ***23*** *)*. ECD (*c* 4.2 × 10^−4^ M, MeCN) λ_max_ (Δ*ε*) 344 (−3.3), 285 (−15.0), 259 (+5.8), 211 (+27.3), 191 (−9.0) nm.

*Guajadial F (* ***24*** *)*. ECD (*c* 4.9 × 10^−4^ M, MeCN) λ_max_ (Δ*ε*) 349 (+1.2), 277 (+25.0), 247 (−3.5), 215 (+20.6), 192 (−43.0) nm.

*Guajavadial A (* ***25*** *)*. Colourless oil; [*α*]^26^_D_ −33 (*c* 0.2, CHCl_3_); UV (MeCN) λ_max_ (log *ε*) 280 (4.3) nm; ECD (*c* 1.1 × 10^−4^ M, MeCN) λ_max_ (Δ*ε*) 340 (−2.4), 284 (−9.8), 246 (+1.7), 191 (+31.2) nm; IR (KBr) *ν*_max_ 3447, 2957, 2923, 2851, 1635, 1442, 1307, 1219, 772 cm^−1^; ^1^H and ^13^C NMR data, see Supplementary Table S2.6; (−) MS-ESI *m/z* 405.3 [M – H]^−^; (−) HR-MS-ESI *m/z* 405.1688 [M – H]^−^ (calcd for C_25_H_25_O_5_, 405.1707).

### ECD Calculations

For details of the ECD calculation for compounds **1**–**4**, see Supplementary S4.

### PDE4D Inhibitory Screening Assays

The protocols for expression, purification, and enzymatic assays of PDE4D2 were similar to those we described previously^31^. More details about the experimental procedures are provided in Supplementary S3.

## Electronic supplementary material

Supplementary Information

## References

[1] Kozuka M (1982). The granulation-inhibiting principles from *Eucalyptus globulus* Labill. II. The structures of euglobal Ia1, Ia2, Ib, Ic, IIa, IIb and IIc. Chem. Pharm. Bull..

[2] Nishizawa M (1992). Macrocarpals: HIV-reverse transcriptase inhibitors of *Eucalyptus globulus*. Tetrahedron Lett..

[3] Osawa K, Yasuda H, Morita H, Takeya K, Itokawa H (1996). Macrocarpals H, I, and J from the leaves of *Eucalyptus globulus*. J. Nat. Prod..

[4] Yin S (2007). Eucalyptals A–C with a new skeleton isolated from *Eucalyptus globulus*. Org. Lett..

[5] Yang S-P (2012). Potent HGF/c-Met axis inhibitors from *Eucalyptus globulus*: the coupling of phloroglucinol and sesquiterpenoid is essential for the activity. J. Med. Chem..

[6] Yang X-L, Hsieh K-L, Liu J-K (2007). Guajadial:  an unusual meroterpenoid from guava leaves *Psidium guajava*. Org. Lett..

[7] Fu H-Z, Luo Y-M, Li C-J, Yang J-Z, Zhang D-M (2010). Psidials A–C, three unusual meroterpenoids from the leaves of *Psidium guajava* L. Org. Lett..

[8] Gao Y (2012). Isolation and biomimetic synthesis of (±)-guajadial B, a novel meroterpenoid from *Psidium guajava*. Org. Lett..

[9] Shao M (2010). Psiguadials A and B, two novel meroterpenoids with unusual skeletons from the leaves of *Psidium guajava*. Org. Lett..

[10] Shao M (2012). Guadial A and psiguadials C and D, three unusual meroterpenoids from *Psidium guajava*. Org. Lett..

[11] Jian Y-Q (2015). Guapsidial A and guadials B and C: three new meroterpenoids with unusual skeletons from the leaves of *Psidium guajava*. Chem. Eur. J..

[12] Liu HX (2016). Isolation and biomimetic total synthesis of tomentodiones A–B, terpenoid-conjugated phloroglucinols from the leaves of *Rhodomyrtus tomentosa*. RSC Adv..

[13] Zhang Y-L (2016). Rhodomyrtials A and B, two meroterpenoids with a triketone-sesquiterpene-triketone skeleton from *Rhodomyrtus tomentosa*: structural elucidation and biomimetic synthesis. Org. Lett..

[14] Liu C (2016). Meroterpenoids with new skeletons from *Myrtus communis* and structure revision of myrtucommulone K. Org. Lett..

[15] Hu LZ (2016). (±)-Japonicols A–D, acylphloroglucinol-based meroterpenoid enantiomers with anti-KSHV activities from *Hypericum japonicum*. J. Nat. Prod..

[16] Hu LZ (2016). Filicinic acid based meroterpenoids with anti-Epstein-Barr virus activities from *Hypericum japonicum*. Org. Lett..

[17] Yang X-W, Li Y-P, Su J, Ma W-G, Xu G (2016). Hyperjapones A–E, terpenoid polymethylated acylphloroglucinols from *Hypericum japonicum*. Org. Lett..

[18] Singh IP, Bharate SB (2006). Phloroglucinol compounds of natural origin. Nat. Prod. Rep..

[19] Xu R, Snyder JK, Nakanishi K (1984). Robustadials A and B from *Eucalyptus robusta*. J. Am. Chem. Soc..

[20] Lal K, Zarate EA, Youngs WJ, Salomon RG (1986). Total synthesis necessitates revision of the structure of robustadials. J. Am. Chem. Soc..

[21] Salomon RG, Lal K, Mazza SM, Zarate EA, Youngs WJ (1988). The total synthesis of robustadial A and B dimethyl ethers. J. Am. Chem. Soc..

[22] Koser S, Hoffmann HMR, Williams DJ (1993). Stereoselective synthesis of precursors of naturally occurring robustadials A and B. J. Org. Chem..

[23] Majewski M, Irvine NM, Bantle GW (1994). Stereoselective synthesis of dimethylrobustadials. J. Org. Chem..

[24] Tanaka T (1998). Total synthesis of (−)-macrocarpal C. Stereoselective coupling reaction with a novel hexasubstituted benzene Cr(CO)_3_ complex as a biomimetic chiral benzyl cation equivalent. J. Org. Chem..

[25] Tran DN, Cramer N (2014). Biomimetic synthesis of (+)-ledene, (+)-viridiflorol, (−)-palustrol, (+)-spathulenol, and psiguadial A, C, and D via the platform terpene (+)-bicyclogermacrene. Chem. Eur. J..

[26] Lam HC, Spence JTJ, George JH (2016). Biomimetic total synthesis of hyperjapones A–E and hyperjaponols A and C. Angew. Chem. Int. Ed..

[27] Lawrence AL (2010). A short biomimetic synthesis of the meroterpenoids guajadial and psidial A. Org. Lett..

[28] Burgin AB (2010). Design of phosphodiesterase 4D (PDE4D) allosteric modulators for enhancing cognition with improved safety. Nat. Biotechnol..

[29] Fabbri LM, Beghe B, Yasothan U, Kirkpatrick P (2010). Roflumilast. Nat. Rev. Drug Discovery.

[30] Cheng Z-B (2014). Prostaglandin derivatives: nonaromatic phosphodiesterase-4 inhibitors from the soft coral *Sarcophyton ehrenbergi*. J. Nat. Prod..

[31] Lin T-T (2014). Prenylated coumarins: natural phosphodiesterase-4 inhibitors from *Toddalia asiatica*. J. Nat. Prod..

[32] Liu X (2014). Selaginpulvilins A–D, new phosphodiesterase-4 inhibitors with an unprecedented skeleton from *Selaginella pulvinata*. Org. Lett..

[33] Fuerstner A, Hannen P (2006). Platinum- and gold-catalyzed rearrangement reactions of propargyl acetates: total syntheses of (−)-*α*-cubebene, (−)-cubebol, sesquicarene and related terpenes. Chem. Eur. J..

[34] Gao Y (2013). Guajadials C–F, four unusual meroterpenoids from *Psidium guajava*. Nat. Prod. Bioprospect..

[35] Qin XJ (2016). Cytotoxic meroterpenoids with rare skeletons from *Psidium guajava* cultivated in temperate zone. Sci. Rep..

[36] Li CJ, Ma J, Sun H, Zhang D, Zhang DM (2016). Guajavadimer A, a dimeric caryophyllene-derived meroterpenoid with a new carbon skeleton from the leaves of *Psidium guajava*. Org. Lett..

[37] Shu J, Chou G, Wang Z (2010). Two new benzophenone glycosides from the fruit of *Psidium guajava* L. Fitoterapia.

[38] Thuaytong W, Anprung P (2011). Bioactive compounds and prebiotic activity in Thailand-grown red and white guava fruit (*Psidium guajava* L.). Food Sci. Technol. Int..

[39] Khadhri A, El Mokni R, Almeida C, Nogueira JMF, Araújo MEM (2014). Chemical composition of essential oil of *Psidium guajava* L. growing in Tunisia. Ind. Crop. Prod..

