# Supplementary material for: Psiguajadials A–K: Unusual Psidium Meroterpenoids as Phosphodiesterase-4 Inhibitors from the Leaves of Psidium guajava
Source: Sci Rep. 2017 Apr 21;7:1047. doi: 10.1038/s41598-017-01028-4 (PMC5430738; doi:10.1038/s41598-017-01028-4)

Supplementary Information

Psiguajadials A–K: Unusual *Psidium* Meroterpenoids as Phosphodiesterase-4 Inhibitors from the Leaves of *Psidium guajava*

Gui-Hua Tang, Zhen Dong, Yan-Qiong Guo, Zhong-Bin Cheng, Chu-Jun Zhou, and Sheng Yin*[a]

Contents:

S1. Figures.

Figure S1.1. Selected ^{1}H–^{1}H COSY, HMBC, and NOESY correlations of compounds **5–10**.

Figure S1.2. The original and revised structures of guajavadial C and key NOE correlations of guajavadial C (**12**).

Figure S1.3. (A) Experimental ECD spectrum of **12** and TDDFT calculated ECD spectra for **12a** (4*S*, 5*R*, 6*R*, 7*R*, 1′*S*) and enantiomer of **12a**. (B) Experimental ECD spectrum of **25** and TDDFT calculated ECD spectra for **25a** (1*S*, 2*S*, 4*S*, 6*R*, 1′*S*) and enantiomer of **25a**.

S2. Tables.

Table S2.1. ^{1}H NMR (400 Hz) and ^{13}C NMR (100 Hz) data of **1** and **2** in pyridine-d_5 (δ in ppm).

Table S2.2. ^{1}H NMR (400 Hz) and ^{13}C NMR (100 Hz) data of **4** in pyridine-d_5 (δ in ppm).

Table S2.3. ^{1}H NMR (400 Hz) and ^{13}C NMR (100 Hz) data of **12** in $CDCl_3$ and pridine-d_5 (δ in ppm).

Table S2.4. ^{13}C NMR (100 Hz) data of known compounds **13–19** in $CDCl_3$ (δ in ppm).

Table S2.5. ^{13}C NMR (100 Hz) data of known compounds **20–24** in $CDCl_3$ (δ in ppm).

Table S2.6. ^{1}H NMR (400 Hz) and ^{13}C NMR (100 Hz) data of known compound **25** in $CDCl_3$ (δ in ppm).

Table S2.7. ^{13}C NMR (100 Hz) data of known compounds **26–28** in $CDCl_3$ (δ in ppm).

S3. Experimental section   Extraction and isolation and PDE4D assay

[a] Dr. G.-H. Tang,[+] Z. Dong,[+] Y.-Q. Guo, Z.-B. Cheng, C.-J. Zhou, Prof. Dr. S. Yin
School of Pharmaceutical Sciences, Sun Yat-sen University, Guangzhou 510006, People's Republic of China
Fax: (+86) 20-3994-3090
E-mail: yinsh2@mail.sysu.edu.cn
[+] These authors contributed equally to this work.

S3.1. Extraction and isolation.

Scheme S3.1. Flow chart for the isolation of chemical constituents from *Psidium guajava*

S3.2. Expression and purification of PDE4D2 protein.

S3.3. Enzymatic assay.

S4. Experimental section ECD calculations

S4.1. ECD calculations of compounds **1–4**, **12**, and **25**.

S4.2. ECD simulation.

S4.3. Lowest energy conformers.

S4.4. Energy analysis table.

S4.5. ECD data of compounds **1–4**, **12**, and **25**.

S5. 1D and 2D NMR spectra of new compounds 1–11 (in $CDCl_3$).

**S5.1.** ^{1}H NMR spectrum of compound **1**	**S5.2.** DEPT spectra of compound **1**
**S5.3.** HSQC spectrum of compound **1**	**S5.4.** ^{1}H–^{1}H COSY spectrum of compound **1**
**S5.5.** HMBC spectrum of compound **1**	**S5.6.** NOESY spectrum of compound **1**

**S5.7.** ^{1}H NMR spectrum of compound **2**	**S5.8.** DEPT spectra of compound **2**
**S5.9.** HSQC spectrum of compound **2**	**S5.10.** ^{1}H–^{1}H COSY spectrum of compound **2**
**S5.11.** HMBC spectrum of compound **2**	**S5.12.** NOESY spectrum of compound **2**

**S5.13.** ^{1}H NMR spectrum of compound **3**	**S5.14.** DEPT spectra of compound **3**
**S5.15.** HSQC spectrum of compound **3**	**S5.16.** ^{1}H–^{1}H COSY spectrum of compound **3**
**S5.17.** HMBC spectrum of compound **3**	**S5.18.** NOESY spectrum of compound **3**

**S5.19.** ^{1}H NMR spectrum of compound **4**	**S5.20.** DEPT spectra of compound **4**
**S5.21.** HSQC spectrum of compound **4**	**S5.22.** ^{1}H–^{1}H COSY spectrum of compound **4**
**S5.23.** HMBC spectrum of compound **4**	**S5.24.** NOESY spectrum of compound **4**

**S5.25.** ^{1}H NMR spectrum of compound **5**	**S5.26.** DEPT spectra of compound **5**
**S5.27.** HSQC spectrum of compound **5**	**S5.28.** ^{1}H–^{1}H COSY spectrum of compound **5**
**S5.29.** HMBC spectrum of compound **5**	**S5.30.** NOESY spectrum of compound **5**

**S5.31.** ^{1}H NMR spectrum of compound **6**	**S5.32.** DEPT spectra of compound **6**
**S5.33.** HSQC spectrum of compound **6**	**S5.34.** ^{1}H–^{1}H COSY spectrum of compound **6**
**S5.35.** HMBC spectrum of compound **6**	**S5.36.** NOESY spectrum of compound **6**

**S5.37.** ^{1}H NMR spectrum of compound **7**	**S5.38.** DEPT spectra of compound **7**

S5.39. HSQC spectrum of compound **7**
S5.40. ^{1}H–^{1}H COSY spectrum of compound **7**
S5.41. HMBC spectrum of compound **7**
S5.42. NOESY spectrum of compound **7**

S5.43. ^{1}H NMR spectrum of compound **8**
S5.44. DEPT spectra of compound **8**
S5.45. HSQC spectrum of compound **8**
S5.46. ^{1}H–^{1}H COSY spectrum of compound **8**
S5.47. HMBC spectrum of compound **8**
S5.48. NOESY spectrum of compound **8**

S5.49. ^{1}H NMR spectrum of compound **9**
S5.50. DEPT spectra of compound **9**
S5.51. HSQC spectrum of compound **9**
S5.52. ^{1}H–^{1}H COSY spectrum of compound **9**
S5.53. HMBC spectrum of compound **9**
S5.54. NOESY spectrum of compound **9**

S5.55. ^{1}H NMR spectrum of compound **10**
S5.56. DEPT spectra of compound **10**
S5.57. HSQC spectrum of compound **10**
S5.58. ^{1}H–^{1}H COSY spectrum of compound **10**
S5.59. HMBC spectrum of compound **10**
S5.60. NOESY spectrum of compound **10**

S5.61. ^{1}H NMR spectrum of compound **11**
S5.62. DEPT spectra of compound **11**
S5.63. HSQC spectrum of compound **11**
S5.64. ^{1}H–^{1}H COSY spectrum of compound **11**
S5.65. HMBC spectrum of compound **11**
S5.66. NOESY spectrum of compound **11**

S6. 1D and 2D NMR spectra of new compounds 1, 2, and 4 (in pyridine-d_5).

S6.1. ^{1}H NMR spectrum of compound **1**
S6.2. DEPT spectra of compound **1**
S6.3. HSQC spectrum of compound **1**
S6.4. ^{1}H–^{1}H COSY spectrum of compound **1**
S6.5. HMBC spectrum of compound **1**
S6.6. NOESY spectrum of compound **1**

S6.7. ^{1}H NMR spectrum of compound **2**
S6.8. DEPT spectra of compound **2**
S6.9. HSQC spectrum of compound **2**
S6.10. ^{1}H–^{1}H COSY spectrum of compound **2**
S6.11. HMBC spectrum of compound **2**
S6.12. NOESY spectrum of compound **2**

S6.13. ^{1}H NMR spectrum of compound **4**
S6.14. DEPT spectra of compound **4**
S6.15. HSQC spectrum of compound **4**
S6.16. ^{1}H–^{1}H COSY spectrum of compound **4**
S6.17. HMBC spectrum of compound **4**
S6.18. NOESY spectrum of compound **4**

S7. MS, HRMS, IR, UV, and ECD spectra of compounds 1–12 and 25, and ECD spectra of compounds 12, 14, 15, and 20-25.

S7.1. ESIMS data of compound **1**
S7.2. HRESIMS data of compound **1**
S7.3. ESIMS data of compound **2**
S7.4. HRESIMS data of compound **2**
S7.5. ESIMS data of compound **3**
S7.6. HRESIMS data of compound **3**
S7.7. ESIMS data of compound **4**
S7.8. HRESIMS data of compound **4**
S7.9. ESIMS data of compound **5**
S7.10. HRESIMS data of compound **5**
S7.11. ESIMS data of compound **6**
S7.12. HRESIMS data of compound **6**

S7.13. ESIMS data of compound **7** **S7.14.** HRESIMS data of compound **7**
S7.15. ESIMS data of compound **8** **S7.16.** HRESIMS data of compound **8**
S7.17. ESIMS data of compound **9** **S7.18.** HRESIMS data of compound **9**
S7.19. ESIMS data of compound **10** **S7.20.** HRESIMS data of compound **10**
S7.21. ESIMS data of compound **11** **S7.22.** HRESIMS data of compound **11**
S7.23. ESIMS data of compound **12** **S7.24.** HRESIMS data of compound **12**
S7.25. ESIMS data of compound **25** **S7.26.** HRESIMS data of compound **25**

S7.27. IR (KBr disc) spectrum of compound **1** **S7.28.** IR (KBr disc) spectrum of compound **2**
S7.29. IR (KBr disc) spectrum of compound **3** **S7.30.** IR (KBr disc) spectrum of compound **4**
S7.31. IR (KBr disc) spectrum of compound **5** **S7.32.** IR (KBr disc) spectrum of compound **6**
S7.33. IR (KBr disc) spectrum of compound **7** **S7.34.** IR (KBr disc) spectrum of compound **8**
S7.35. IR (KBr disc) spectrum of compound **9** **S7.36.** IR (KBr disc) spectrum of compound **10**
S7.37. IR (KBr disc) spectrum of compound **11** **S7.38.** IR (KBr disc) spectrum of compound **12**
S7.39. IR (KBr disc) spectrum of compound **25**

S7.40. UV spectrum of compound **1** **S7.41.** UV spectrum of compound **2**
S7.42. UV spectrum of compound **3** **S7.43.** UV spectrum of compound **4**
S7.44. UV spectrum of compound **5** **S7.45.** UV spectrum of compound **6**
S7.46. UV spectrum of compound **7** **S7.47.** UV spectrum of compound **8**
S7.48. UV spectrum of compound **9** **S7.49.** UV spectrum of compound **10**
S7.50. UV spectrum of compound **11** **S7.51.** UV spectrum of compound **12**
S7.52. UV spectrum of compound **25**

S7.53. ECD spectrum of compound **1** **S7.54.** ECD spectrum of compound **2**
S7.55. ECD spectrum of compound **3** **S7.56.** ECD spectrum of compound **4**
S7.57. ECD spectrum of compound **5** **S7.58.** ECD spectrum of compound **6**
S7.59. ECD spectrum of compound **7** **S7.60.** ECD spectrum of compound **8**
S7.61. ECD spectrum of compound **9** **S7.62.** ECD spectrum of compound **10**
S7.63. ECD spectrum of compound **11** **S7.64.** ECD spectrum of compound **12**
S7.65. ECD spectrum of compound **14** **S7.66.** ECD spectrum of compound **15**
S7.67. ECD spectrum of compound **20** **S7.68.** ECD spectrum of compound **21**
S7.69. ECD spectrum of compound **22** **S7.70.** ECD spectrum of compound **23**
S7.71. ECD spectrum of compound **24** **S7.72.** ECD spectrum of compound **25**

S8. 1D NMR spectra of known compounds 12 (in $CDCl_3$ and pyridine-d_5) and 13–28 (in $CDCl_3$)

In $CDCl_3$
S8.1. ^{1}H NMR spectrum of compound **12** **S8.2.** DEPT spectra of compound **12**
S8.3. HSQC spectrum of compound **12** **S8.4.** ^{1}H–^{1}H COSY spectrum of compound **12**
S8.5. HMBC spectrum of compound **12** **S8.6.** NOESY spectrum of compound **12**

In pyridine-*d*5

**S8.7.** ^{1}H NMR spectrum of compound **12**	**S8.8.** DEPT spectra of compound **12**
**S8.9.** HSQC spectrum of compound **12**	**S8.10.** ^{1}H–^{1}H COSY spectrum of compound **12**
**S8.11.** HMBC spectrum of compound **12**	**S8.12.** NOESY spectrum of compound **12**

**S8.13.** ^{1}H NMR spectrum of compound **13**	**S8.14.** DEPT spectra of compound **13**
**S8.15.** ^{1}H NMR spectrum of compounds **14** and **15**	**S8.16.** ^{13}C NMR spectrum of compounds **14** and **15**
**S8.17.** ^{1}H NMR spectrum of compound **16**	**S8.18.** DEPT spectra of compound **16**
**S8.19.** ^{1}H NMR spectrum of compound **17**	**S8.20.** DEPT spectra of compound **17**
**S8.21.** ^{1}H NMR spectrum of compound **18**	**S8.22.** DEPT spectra of compound **18**
**S8.23.** ^{1}H NMR spectrum of compound **19**	**S8.24.** DEPT spectra of compound **19**
**S8.25.** ^{1}H NMR spectrum of compound **20**	**S8.26.** DEPT spectra of compound **20**
**S8.27.** ^{1}H NMR spectrum of compound **21**	**S8.28.** DEPT spectra of compound **21**
**S8.29.** ^{1}H NMR spectrum of compound **22**	**S8.30.** DEPT spectra of compound **22**
**S8.31.** ^{1}H NMR spectrum of compound **23**	**S8.32.** DEPT spectra of compound **23**
**S8.33.** ^{1}H NMR spectrum of compound **24**	**S8.34.** DEPT spectra of compound **24**

**S8.35.** ^{1}H NMR spectrum of compound **25**	**S8.36.** DEPT spectra of compound **25**
**S8.37.** HSQC spectrum of compound **25**	**S8.38.** ^{1}H–^{1}H COSY spectrum of compound **25**
**S8.39.** HMBC spectrum of compound **25**	**S8.40.** NOESY spectrum of compound **25**

**S8.41.** ^{1}H NMR spectrum of compound **26**	**S8.42.** DEPT spectra of compound **26**
**S8.43.** ^{1}H NMR spectrum of compound **27**	**S8.44.** DEPT spectra of compound **27**
**S8.45.** ^{1}H NMR spectrum of compound **28**	**S8.46.** DEPT spectra of compound **28**

S1. Figures

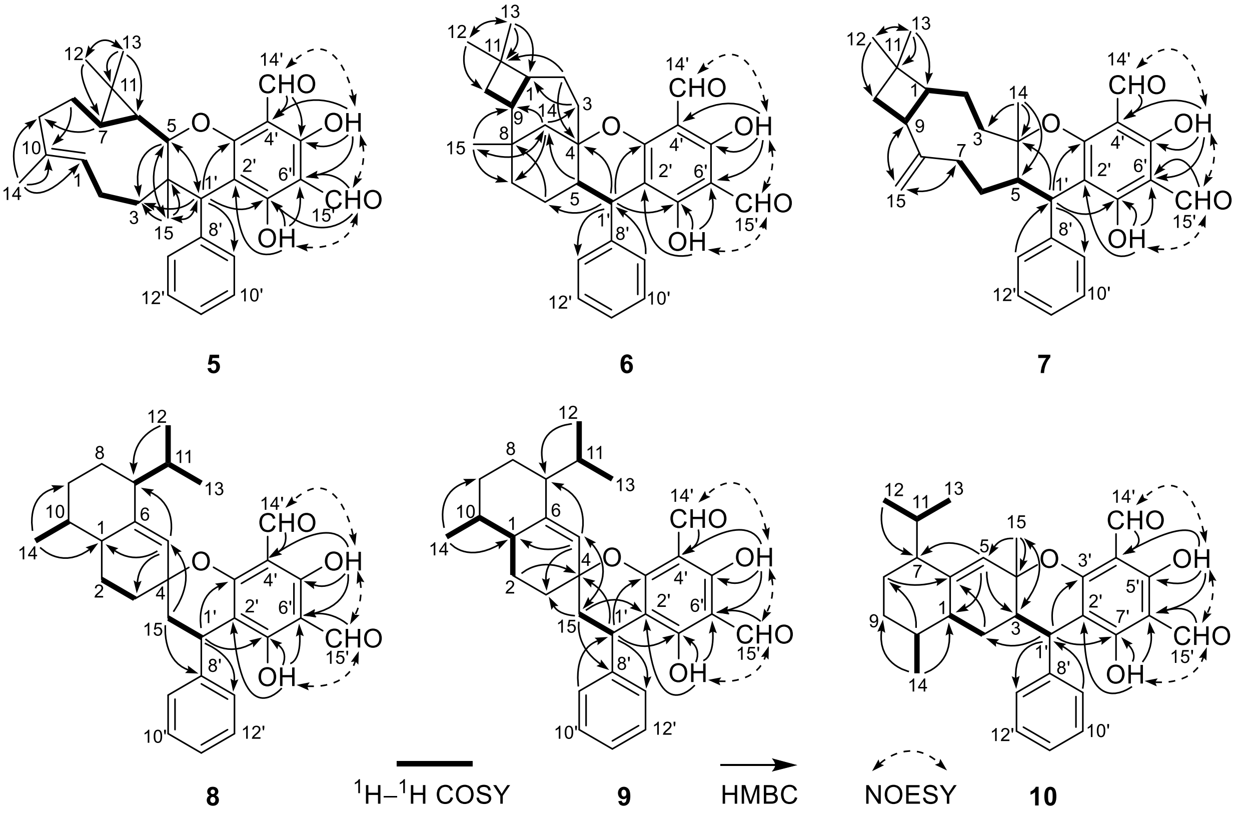

Figure S1.1. Selected ^{1}H–^{1}H COSY, HMBC, and NOESY correlations of compounds **5–10**.

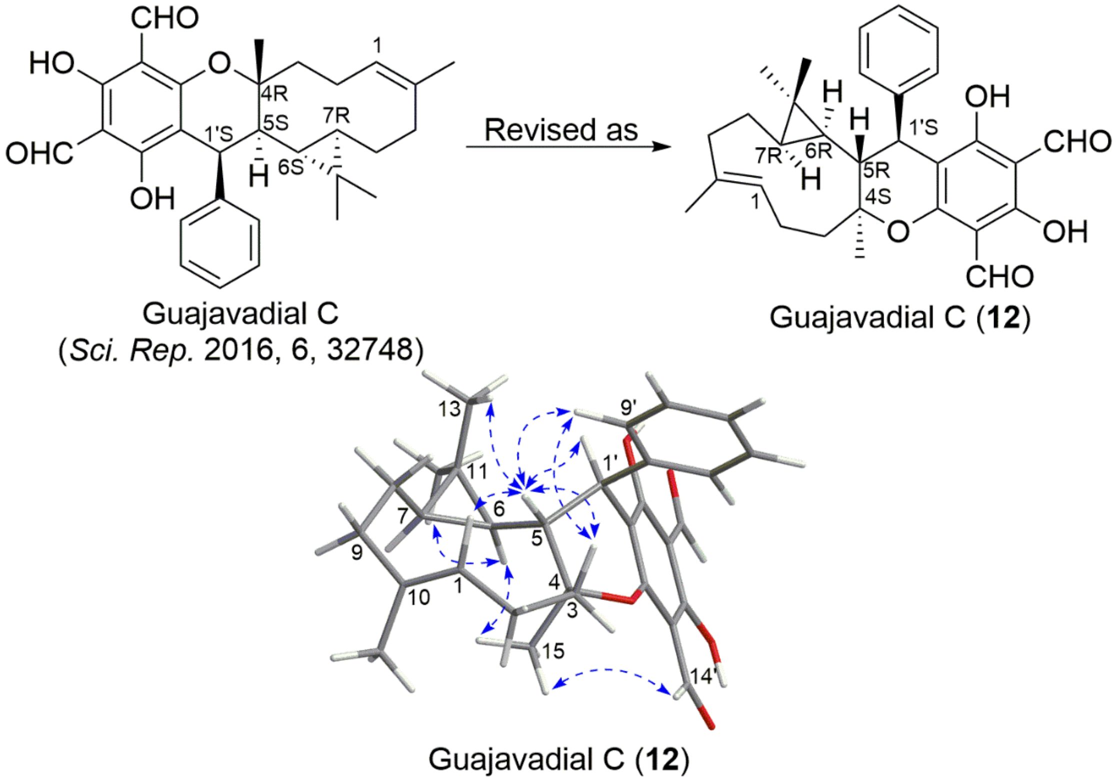

Figure S1.2. The original and revised structures of guajavadial C and key NOE correlations of guajavadial C (**12**).

A

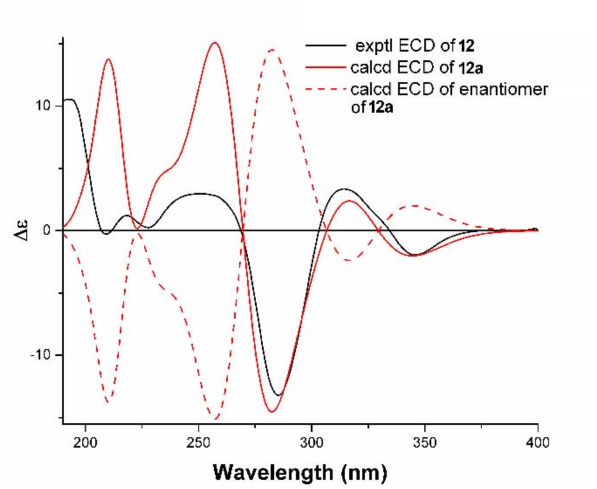

B

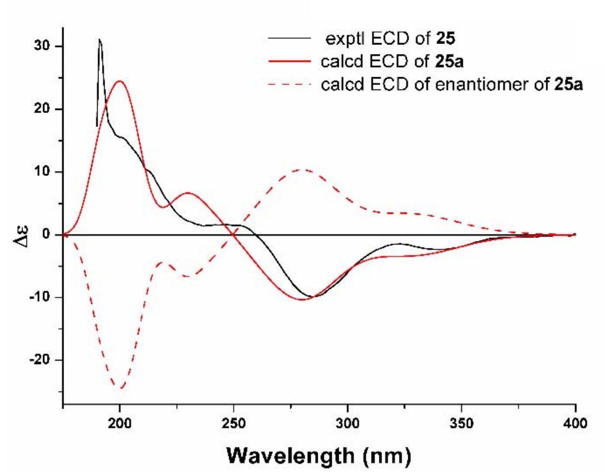

Figure S1.3. (A) Experimental ECD spectrum of **12** and TDDFT calculated ECD spectra for **12a** (4*S*, 5*R*, 6*R*, 7*R*, 1′*S*) and enantiomer of **12a**. (B) Experimental ECD spectrum of **25** and TDDFT calculated ECD spectra for **25a** (1*S*, 2*S*, 4*S*, 6*R*, 1′*S*) and enantiomer of **25a**.

S2. Tables

Table S2.1. ^{1}H NMR (400 Hz) and ^{13}C NMR (100 Hz) data of **1** and **2** in pyridine-d_5 (δ in ppm).

Position	**1**		**2**	
	δ_H, multi. (J in Hz)	δ_C, type	δ_H, multi. (J in Hz)	δ_C, type
1	-	34.9, C	-	34.3, C
2	β 1.55, m	28.7, CH_2	β 2.02, dd (12.8, 7.8)	39.5, CH_2
	α 1.83, m		α 1.83, t (12.1)	
3	β 1.83, m	33.7, CH_2	2.58, t (9.2)	45.1, CH
	α 1.59, m			
4	-	90.1,	-	91.7, C
5	1.34, d (2.7)	33.7, CH	1.24, br.s	41.5, CH
6	1.12, m	23.6, CH	0.81, br.s	27.6, CH
7	0.79, m	44.5, CH	0.98, m	45.3, CH
8	α 1.28, m	27.6, CH_2	α 1.36, m	27.6, CH_2
	β 0.79, m		β 0.83, m	
9	β 1.56, m	31.7, CH_2	β 1.57, m	32.0, CH_2
	α 0.54, dddd (12.1, 12.1, 12.1, 2.0)		α 0.54, q (12.5)	
10	1.64, m	30.8, CH	1.68, m	30.3, CH
11	1.48, m	33.9, CH	1.53, m	33.6, CH
12	0.91, d (6.4)	19.8, CH_3	0.92, d (6.9)	20.5, CH_3
13	0.85, d (6.6)	20.5, CH_3	0.90, d (6.8)	20.6, CH_3
14	1.03, d (6.2)	19.0, CH_3	0.80, d (6.5)	19.4, CH_3
15	α 2.23, dd (14.0, 7.2)	42.4, CH_2	1.21, s	24.5, CH_3
	β 1.98, dd (14.0, 10.3)			
1′	4.44, dd (10.3, 7.2)	35.5, CH	4.46, br.s	36.2, CH
2′	-	104.1, C	-	101.2, C
3′	-	166.1, C	-	164.3, C
4′	-	104.7, C	-	104.8, C
5′	-	168.6, C	-	169.3, C
6′	-	104.6, C	-	104.7, C
7′	-	169.9, C	-	170.4, C
8′	-	145.7, C	-	143.6, C
9′/13′	7.37, m	127.4, CH	7.31, m	128.5, CH
10′/12′	7.43, m	129.0, CH	7.38, t (7.6)	129.4, CH
11′	7.32, m	126.6, CH	7.31, m	127.3, CH
14′	10.38, s	192.9, CH	10.33, s	192.2, CH
15′	10.21, s	191.7, CH	10.42, s	193.2, CH

Table S2.2. ^{1}H NMR (400 Hz) and ^{13}C NMR (100 Hz) data of **4** in pyridine-d_5 (δ in ppm).

Position	**4**	
	δ_H, multi. (J in Hz)	δ_C, type
1	1.84, m	53.7, CH
2	α 1.20, m	22.3, CH_2
	β 0.97, m	
3	β 1.70, m	37.8, CH_2
	α 1.18, m	
4	-	84.2, C
5	1.97, m	33.7, CH
6	β 1.53, m	33.6, CH_2
	α 1.40, m	
7	α 2.35, m	35.3, CH_2
	β 2.02, m	
8	-	152.6, C
9	2.30, m	41.6, CH
10	α 1.70, m	36.6, CH_2
	β 1.55, m	
11	-	33.7, C
12	0.93, s	30.2, CH_3
13	0.87, s	22.1, CH_3
14	1.00, s	20.6, CH_3
15	a 5.00, br.s	110.5, CH_2
	b 4.94, br.s	
1′	-	197.8, C
2′	-	106.7, C
3′	-	161.4, C
4′	-	101.4, C
5′	-	165.4, C
6′	-	105.6, C
7′	-	164.6, C
8′	-	141.5, C
9′/13′	7.81, m	128.3, CH
10′/12′	7.45, m	128.5, CH
11′	7.52, m	131.8, CH
14′	α 2.72, dd (16.5, 5.4)	25.0, CH_2
	β 2.13, dd (16.5, 12.1)	
15′	10.53, s	192.7

Table S2.3. ^{1}H NMR (400 Hz) and ^{13}C NMR (100 Hz) data of **12** in $CDCl_3$ and pridine-d_5 (δ in ppm).

Position	**12**[a]		**12**[b]	
	δ_H, multi. (J in Hz)	δ_C, type	δ_H, multi. (J in Hz)	δ_C, type
1	5.19, d (11.4)	123.5, CH	5.19, d (12.0)	124.4, CH
2	α 2.33, m<br>β 1.99, m	23.6, CH_2	α 2.28, m<br>β 1.88, m	24.3, CH_2
3	α 1.83, m<br>β 1.57, m	43.2, CH_2	α 1.89, m<br>β 1.78, m	44.1, CH_2
4	-	91.7, C	-	92.5, C
5	2.71, d (7.9)	49.2, CH	2.85, d (10.0)	49.9, CH
6	0.38, m	29.3, CH	0.58, t (9.3)	30.2, CH
7	0.38, m	28.6, CH	0.40, t (9.8)	29.3, CH
8	α 1.90, m<br>β 1.36, m	25.4, CH_2	α 1.73, m<br>β 1.35, m	26.1, CH_2
9	2.45, d (14.8)<br>1.63, m	34.9, CH_2	β 2.40, m<br>α 1.57, m	35.6, CH_2
10	-	133.9, C	-	134.4, C
11	-	17.2, C	-	17.8, C
12	α 0.97, s	30.1, CH_3	1.10, s	30.6, CH_3
13	β 1.28, s	17.2, CH_3	1.35, s	17.7, CH_3
14	1.69, s	20.9, CH_3	1.65, s	21.3, CH_3
15	1.22, s	24.5, CH_3	1.22, s	24.8, CH_3
1′	4.53, br. s	38.1, CH	4.78, br.s	39.2, CH
2′	-	108.4, C	-	109.3, C
3′	-	165.8, C	-	168.0, C
4′	-	106.6, C	-	105.9, C
5′	-	168.1, C	-	168.5, C
6′	-	105.0, C	-	107.8, C
7′	-	167.6, C	-	166.6, C
8′	-	141.9, C	-	143.2, C
9′/13′	7.11, d (7.6)	127.5, CH	7.31, m	128.6, CH
10′/12′	7.25, t (7.6)	128.4, CH	7.45, m	129.4, CH
11′	7.18, t (7.6)	126.2, CH	7.31, m	127.2, CH
14′	10.04, s	192.0, CH	10.35, s	192.9, CH
15′	10.25, s	192.2, CH	10.25, s	192.9, CH
5′-OH	13.21, s			
7′-OH	13.01, s			

[a] In $CDCl_3$; [b] In pridine-d_5.

Table S2.4. ^{13}C NMR (100 Hz) data of known compounds **13–19** in $CDCl_3$ (δ in ppm).

Position	**13**	**14/15**	**16**	**17**	**18**	**19**
1	127.5, CH	37.4, CH_2	104.1, C	53.3, CH	59.3, CH	53.3, CH
2	23.3, CH_2	30.8, CH_2	36.6, CH_2	22.5, CH_2	24.4, CH_2	22.2, CH_2
3	35.3, CH_2	43.5, CH	37.9, CH_2	37.9, CH_2	47.8, CH_2	37.1, CH_2
4	41.3, C	85.2, C	47.8, C	84.3, C	88.0, C	84.3, C
5	85.2, CH	42.5, CH_2	48.7, CH	33.6, CH	39.7, CH	43.4, CH
6	26.9, CH	119.3, CH	26.3, CH	33.8, CH	23.9, CH_2	30.4, CH_2
7	31.5, CH	143.2, C	23.9, CH	35.3, CH_2	35.5, CH_2	35.4, CH_2
8	22.4, CH_2	41.4, CH_2	20.3, CH_2	151.9, C	150.7, C	150.7, C
9	38.1, CH_2	123.1, CH	30.3, CH_2	41.8, CH	42.2, CH	41.3, CH
10	130.7, C	136.3, C	39.9, CH	36.4, CH_2	36.1, CH_2	36.7, CH_2
11	19.8, C	38.6, C	19.2, C	33.8, C	34.7, C	33.8, C
12	30.4, CH_3	29.8, CH_3	28.4, CH_3	30.2, CH_3	21.7, CH_3	22.0, CH_3
13	19.3, CH_3	23.9, CH_3	14.3, CH_3	22.2, CH_3	29.7, CH_3	30.4, CH_3
14	17.4, CH_3	16.7, CH_3	17.4, CH_3	21.2, CH_3	22.2, CH_3	21.2, CH_3
15	18.6, CH_3	19.9, CH_3	24.9, CH_3	110.5, CH	111.2, CH_2	110.2, CH_2
1′	43.9, CH	44.7, CH	53.2, CH	199.6, C	35.0, CH	43.4, CH
2′	105.2, C	105.8, C	114.2, C	102.8, C	107.5, C	105.5, C
3′	166.3, C	163.3, C	166.7, C	169.4, C	164.1, C	163.4, C
4′	104.4, C	104.3, C	108.2, C	101.3, C	104.7, C	104.2, C
5′	168.2, C	168.3, C	168.2, C	162.1, C	167.5, C	168.3, C
6′	104.2, C	104.3, C	105.0, C	104.1, C	104.2, C	104.3, C
7′	170.5, C	169.3, C	170.0, C	166.8, C	168.4, C	169.5, C
8′	140.3, C	144.7, C	140.9, C	140.8, C	138.6, C	143.7, C
9′/13′	127.7/127.4, CH	128.3, CH	130.3, CH	128.1, CH	129.9, CH	128.7, CH
10′/12′	130.2/127.4, CH	128.3, CH	127.6, CH	127.7, CH	128.0, CH	127.9, CH
11′	126.2, CH	126.5, CH	126.4, CH	131.5, CH	126.9, CH	126.2, CH
14′	192.1, CH	192.1, CH	193.7, CH	24.7, CH_2	192.0, CH	192.0, CH
15′	191.5, CH	191.6, CH	191.8, CH	191.7, CH	191.6, CH	191.5, CH

Table S2.5. ^{13}C NMR (100 Hz) data of known compounds **20–24** in $CDCl_3$ (δ in ppm).

Position	**20**	**21**	**22**	**23**	**24**
1	57.4, CH	37.7, CH	37.3, CH	34.2, CH	35.2, CH
2	22.4, CH_2	22.0, CH_2	22.2, CH_2	27.2, CH_2	26.0, CH_2
3	38.3, CH_2	30.9, CH_2	34.3, CH_2	44.5, CH_2	38.2, CH
4	84.7, C	78.0, C	77.9, C	80.1, C	78.0, C
5	43.8, CH	125.5, CH	123.0, CH	126.8, CH	125.8, CH
6	33.6, CH_2	147.8, C	148.2, C	144.6, C	147.2, C
7	37.2, CH_2	50.6, CH	51.2, CH	50.9, CH	51.0, CH
8	154.9, C	22.1, CH_2	22.0, CH_2	22.6, CH_2	22.9, CH_2
9	42.2, CH	28.7, CH_2	28.7, CH_2	29.1, CH_2	28.8, CH_2
10	38.7, CH_2	33.1, CH	33.5, CH	33.8, CH	36.7, CH
11	33.2, C	27.0, CH	26.9, CH	26.5, CH	26.3, CH
12	22.4, CH_3	21.5, CH_3	21.6, CH_3	21.1, CH_3	21.4, CH_3
13	29.5, CH_3	21.2, CH_3	21.3, CH_3	21.1, CH_3	21.0, CH_3
14	20.1, CH_3	14.3, CH_3	14.3, CH_3	14.3, CH_3	14.8, CH_3
15	109.6, CH_2	43.7, CH_2	43.2, CH_2	28.1, CH_2	24.4, CH_3
1′	44.3, CH	34.6, CH	34.1, CH	38.6, CH	38.3, CH
2′	105.0, C	102.8, C	103.0, C	104.4, C	100.6, C
3′	164.6, C	164.5, C	164.7, C	163.4, C	164.2, C
4′	104.1, C	104.6, C	104.6, C	104.0, C	104.5, C
5′	169.38, C	168.6, C	168.6, C	168.2, C	168.5, C
6′	104.2, C	104.1, C	104.0, C	104.0, C	104.2, C
7′	169.40, C	169.8, C	169.7, C	169.7, C	170.3, C
8′	144.1, C	144.6, C	144.5, C	144.4, C	140.7, C
9′/13′	128.3, CH	126.2, CH	126.8, CH	127.6, CH	126.9/127.7, CH
10′/12′	128.1, CH	128.5, CH	128.5, CH	128.3, CH	128.4/127.7, CH
11′	126.4, CH	126.2, CH	126.2, CH	126.2, CH	126.2, CH
14′	192.2, CH	192.4, CH	192.3, CH	191.5, CH	192.4, CH
15′	191.5, CH	191.5, CH	191.6, CH	192.3, CH	191.5, CH

Table S2.6. ^{1}H NMR (400 Hz) and ^{13}C NMR (100 Hz) data of known compound **25** in $CDCl_3$ (δ in ppm).

Position	**25**	
	δ_H, multi. (J in Hz)	δ_C, type
1	-	89.8, C
2	1.39, dd (8.4, 3.6)	35.3, CH
3	β 0.58, dd (8.4, 5.4)	15.0, CH_2
	α 0.49, dd (5.4, 3.6)	
4	-	32.6, C
5	α 2.03, dd (12.5, 7.5)	34.3, CH_2
	β 1.59, dd (12.5, 12.5)	
6	2.23, m	44.1, CH
7	1.04, s	23.5, CH_3
8	1.29, m	32.0, CH
9	0.88, d (6.8)	19.9, CH_3
10	0.81, d (6.8)	19.5, CH_3
1′	4.19, brs	35.2, CH
2′	-	99.6, C
3′	-	163.3, C
4′	-	103.8, C
5′	-	168.7, C
6′	-	103.8, C
7′	-	169.9, C
8′	-	142.5, C
9′/13′	7.07, m	127.3, CH
10′/12′	7.26, m	128.5, CH
11′	7.19, m	126.5, CH
14′	10.17, s	192.1, CH
15′	10.18, s	191.5, CH
5′-OH	13.63, s	
7′-OH	13.24, s	

Table S2.7. ^{13}C NMR (100 Hz) data of known compounds **26–28** in $CDCl_3$ (δ in ppm).

Position	**26**	**27**	**28**	Position	**26**	**27**	**28**
1	88.7, C	56.3, C	47.6, C	1′	35.0, CH	39.1, CH	35.1, CH
2	28.2, CH	88.9, C	86.2, C	2′	103.5, C	101.5, C	103.0, C
3	12.1, CH_2	41.2, CH	29.9, CH_2	3′	165.7, C	166.4, C	164.6, C
4	34.5, C	36.5, CH_2	24.6, CH_2	4′	104.2, C	103.7, C	104.5, C
5	24.3, CH_2	40.6, C	40.6, C	5′	168.5, C	168.8, C	169.5, C
6	33.4, CH_2	40.2, C	38.3, C	6′	104.2, C	104.0, C	104.1, C
7	42.2, CH_2	27.6, CH_2	26.3, CH_2	7′	169.8, C	169.4, C	168.6, C
8	32.5, CH	29.2, CH_3	43.7, CH_2	8′	144.6, C	142.6, C	144.4, C
9	19.5, CH_3	22.8, CH_3	23.1, CH_3	9′/13′	126.7, CH	127.4, CH	126.8, CH
10	19.6, CH_3	27.9, CH_3	27.5, CH_3	10′/12′	128.6, CH	128.6, CH	128.5, CH
				11′	126.3, CH	126.7, CH	126.3, CH
				14′	191.6, CH	191.6, CH	191.6, CH
				15′	192.4, CH	192.1, CH	192.2, CH

S3. Experimental section Extraction and isolation and PDE4D assay

S3.1. Extraction and isolation

See Scheme S3.1.

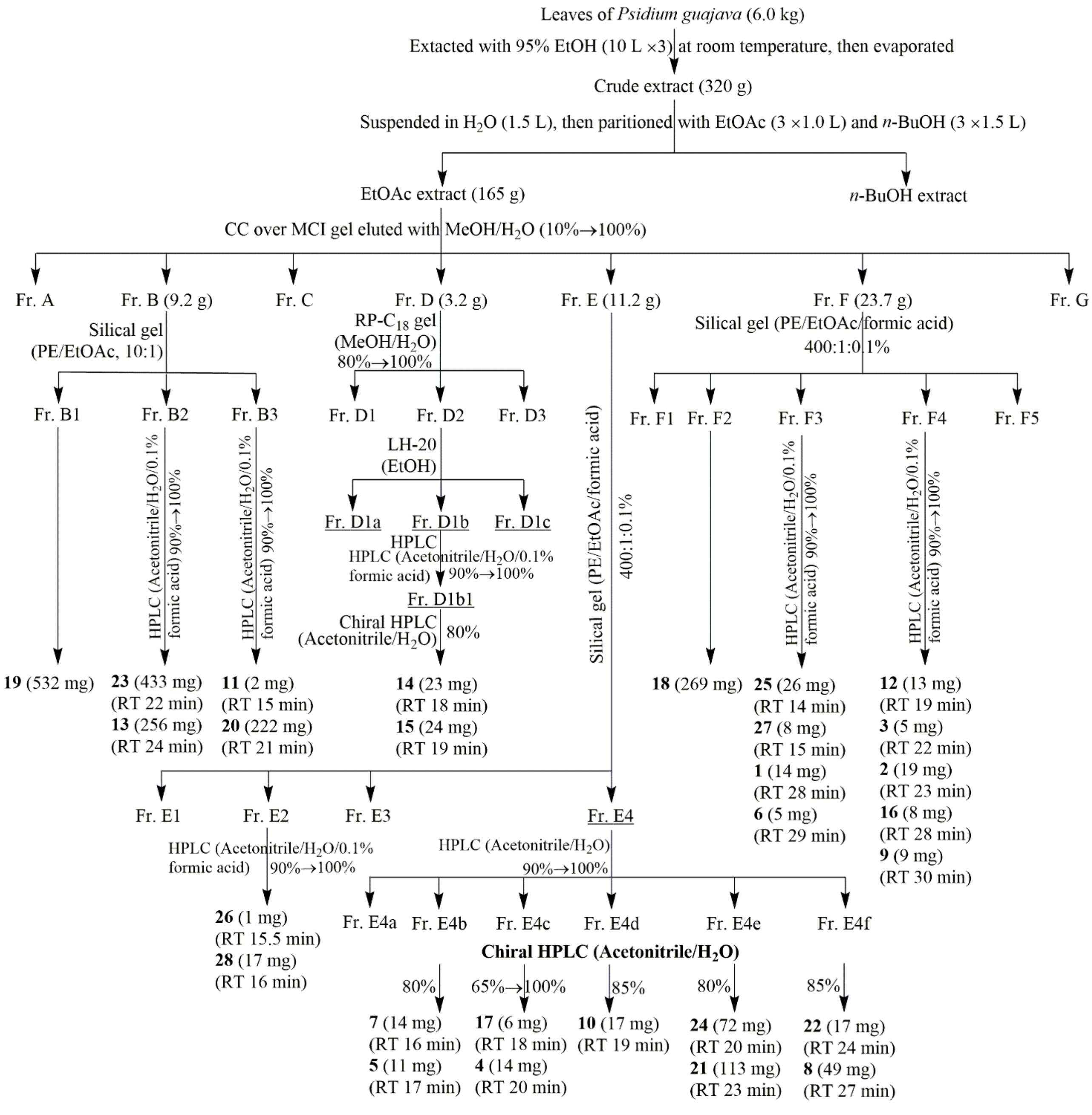

Scheme S3.1. Flow chart for the isolation of chemical constituents from *Psidium guajava*

S3.2. Expression and purification of PDE4D2 protein

The cDNAs for expression of human PDE4D2 (catalytic domain, residues 86–413) were subcloned into the expression vector pET15b. All these resultant plasmids were transformed into E. coli strain BL21 (Codonplus) for over expression. The *E. coli* cells carrying these plasmids were grown in LB medium at 37 °C to OD_{600} = 0.7, and then 0.1 mM isopropyl

β-D-thiogalactopyranoside was added for further growth at 16 °C for 20–40 h. The recombinant protein was purified by Ni-NTA column (Qiagen). The purity of PDE4D2 protein was greater than 95% as shown by SDS–PAGE. A typical batch of purification yielded over 50 mg of PDE4D2 (catalytic domain) from 1 L cell culture.

S3.3. Enzymatic assay.

The enzymatic activities of PDE4D catalytic domain and the inhibition of PDE4D by extracted compounds were assayed by using ^{3}H-cAMP as substrates (20000–30000 cpm/assay) and the reactions were occured in mixture containing 50 mM Tris/HCl (pH 7.5), 10 mM $MgCl_2$, 0.5 mM DTT at room temperature (25 °C) for 15 min. The reactions were terminated by addition of 0.2 M $ZnSO_4$ and $Ba(OH)_2$. The reaction product ^{3}H-AMP was precipitated out, while unreacted ^{3}H-cAMP remained in the supernatant. The mechanism was illustrated as shown below (Scheme S3.2). Radioactivity in the supernatant was measured in 2.5 mL Ultima Gold liquid scintillation cocktails (PerkinElmer) by a PerkinElmer 2910 liquid scintillation counter. Each measurement was repeated at least three times. The IC_{50} values were calculated by nonlinear regression. As a reference compound, rolipram purchased from Sigma was measured its IC_{50} value before other assays.

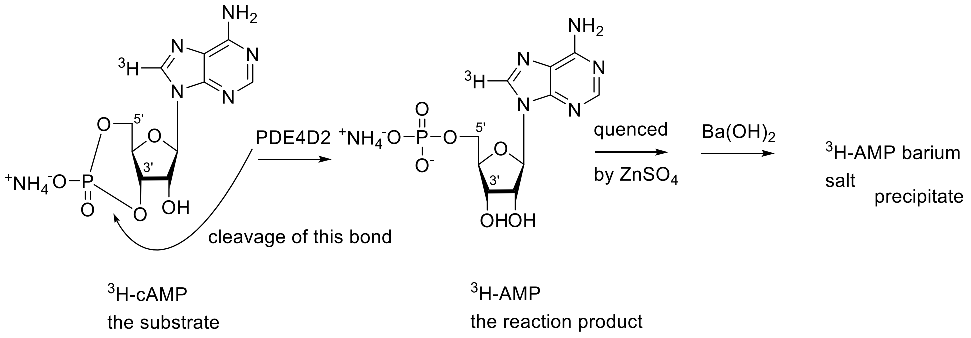

Scheme S3.2. The mechanism of enzymatic assay

S4. Experimental section ECD calculations

S4.1. ECD calculations of compounds 1–4, 12, and 25.

The absolute configurations of **1**–**4**, **12**, and **25**. were determined by quantum chemical TDDFT calculations of their theoretical ECD spectra. Firstly, conformational searching were carried out via Monte Carlo searching using molecular mechanism with MMFF94 force field in the Spartan 08 program.[1] The results showed only one lowest energy conformers for compound **3** (Figure S4.3), two lowest energy conformers for compound **12** (Figure S4.5), three lowest energy conformers for compounds **1** (Figure S4.1), **2** (Figure S4.2), **25** (Figure S4.6), and four lowest energy conformers (relative energy within 2.0 Kcal/mol) for compound **4** (Figure S4.4), respectively. Subsequently, the conformers were reoptimized using DFT at the B3LYP/6-31+G(d) level in vacuum with the Gaussian 09 program.[2] The B3LYP/6-31+G(d) harmonic vibrational frequencies were further calculated to confirm their stability. The energies, oscillator strengths, and rotational strengths of the first 60 electronic excitations were calculated using the TDDFT methodology at the B3LYP/6-311++G(2d,2p) level in vacuum. The ECD spectra were simulated by the overlapping Gaussian function[3] in which velocity rotatory strengths of the first 36 exited states for **1**, the first 35

exited states for **2**, 60 exited states for **3**, the first 55 exited states for **4**, the first 45 exited states for **12**, and the first 45 exited states for **25** were adopted, respectively. To get the final ECD spectra, the simulated spectra of the lowest energy conformers for each compound were averaged according to the Boltzmann distribution theory and their relative Gibbs free energy (G).

References:

1. *Spartan 04*; Wavefunction Inc.:Irvine, CA.

2. *Gaussian 09*, Revision A.1, Frisch, M. J.; Trucks, G. W.; Schlegel, H. B.; Scuseria, G. E.; Robb, M. A.; Cheeseman, J. R.; Scalmani, G.; Barone, V.; Mennucci, B.; Petersson, G. A.; Nakatsuji, H.; Caricato, M.; Li, X.; Hratchian, H. P.; Izmaylov, A. F.; Bloino, J.; Zheng, G.; Sonnenberg, J. L.; Hada, M.; Ehara, M.; Toyota, K.; Fukuda, R.; Hasegawa, J.; Ishida, M.; Nakajima, T.; Honda, Y.; Kitao, O.; Nakai, H.; Vreven, T.; Montgomery, Jr., J. A.; Peralta, J. E.; Ogliaro, F.; Bearpark, M.; Heyd, J. J.; Brothers, E.; Kudin, K. N.; Staroverov, V. N.; Kobayashi, R.; Normand, J.; Raghavachari, K.; Rendell, A.; Burant, J. C.; Iyengar, S. S.; Tomasi, J.; Cossi, M.; Rega, N.; Millam, J. M.; Klene, M.; Knox, J. E.; Cross, J. B.; Bakken, V.; Adamo, C.; Jaramillo, J.; Gomperts, R.; Stratmann, R. E.; Yazyev, O.; Austin, A. J.; Cammi, R.; Pomelli, C.; Ochterski, J. W.; Martin, R. L.; Morokuma, K.; Zakrzewski, V. G.; Voth, G. A.; Salvador, P.; Dannenberg, J. J.; Dapprich, S.; Daniels, A. D.; Farkas, Ö.; Foresman, J. B.; Ortiz, J. V.; Cioslowski, J.; Fox, D. J. Gaussian, Inc., Wallingford CT, 2009.

3. Stephens, P. J.; Harada, N. ECD cotton effect approximated by the Gaussian curve and other methods. *Chirality* **2010**, *22*, 229–233.

S4.2. ECD simulation:

ECD spectrum of each conformation is simulated according to the overlapping Gaussian functions expressed as:

$$\Delta\varepsilon(E)=\frac{1}{2.296\times10^{-39}\sqrt{\pi}\sigma}\sum_{i}^{A}\Delta E_i R_i e^{[-(E-\Delta Ei)^2/\sigma^2]}$$

Where σ is half the bandwidth at 1/e peak height and expressed in energy units. The parameters ΔE_i and R_i are the excitation energies and rotational strengths for the transition *i*, respectively.

The above function is converted to Δε, λ (wavelength) correlations as:

$$\Delta\varepsilon(\lambda)=\frac{1}{2.296\times10^{-39}\sqrt{\pi}\sigma}\sum_{i}^{A}\Delta E_i R_i e^{[-(1240/\lambda-\Delta Ei)^2/\sigma^2]}$$

and then simulation were accomplished by using the Excel 2003 and the Origin 7.0 software.

To get the final spectra, all the simulated spectra of conformations of each compound were averaged according to their energy and the Boltzmann distribution theory expressed as:

$$\frac{N_i^*}{N}=\frac{g_i e^{-\varepsilon_i/k_BT}}{\sum g_i e^{-\varepsilon_i/k_BT}}$$

S4.3. Lowest energy conformers:

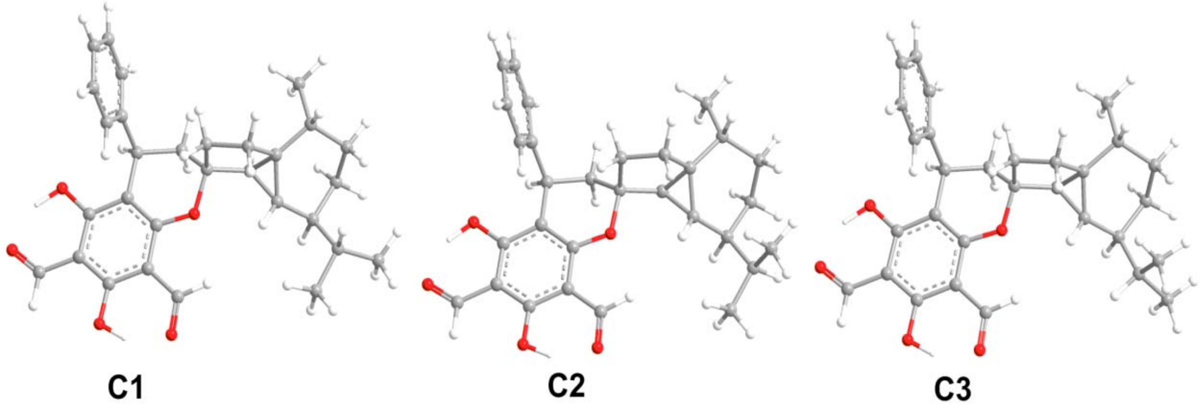

Figure S4.1. B3LYP/6-31+G(d) optimized lowest energy 3D conformers of **1**.

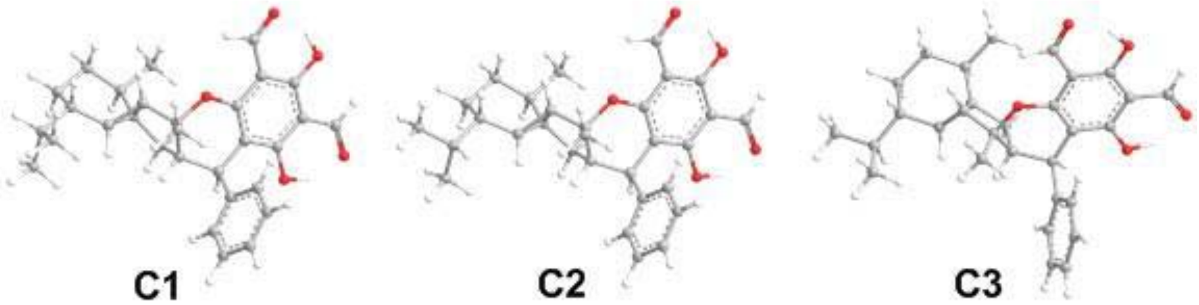

Figure S4.2. B3LYP/6-31G(d) optimized lowest energy 3D conformers of **2**.

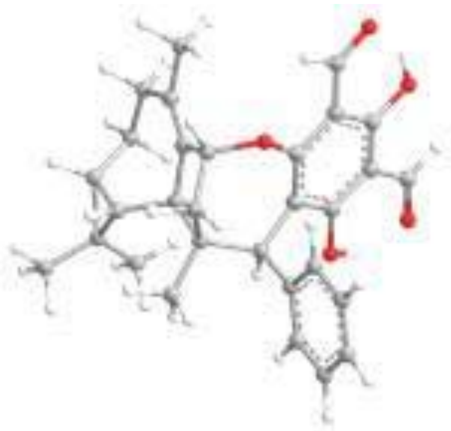

Figure S4.3. B3LYP/6-31G(d) optimized lowest energy 3D conformers of **3**.

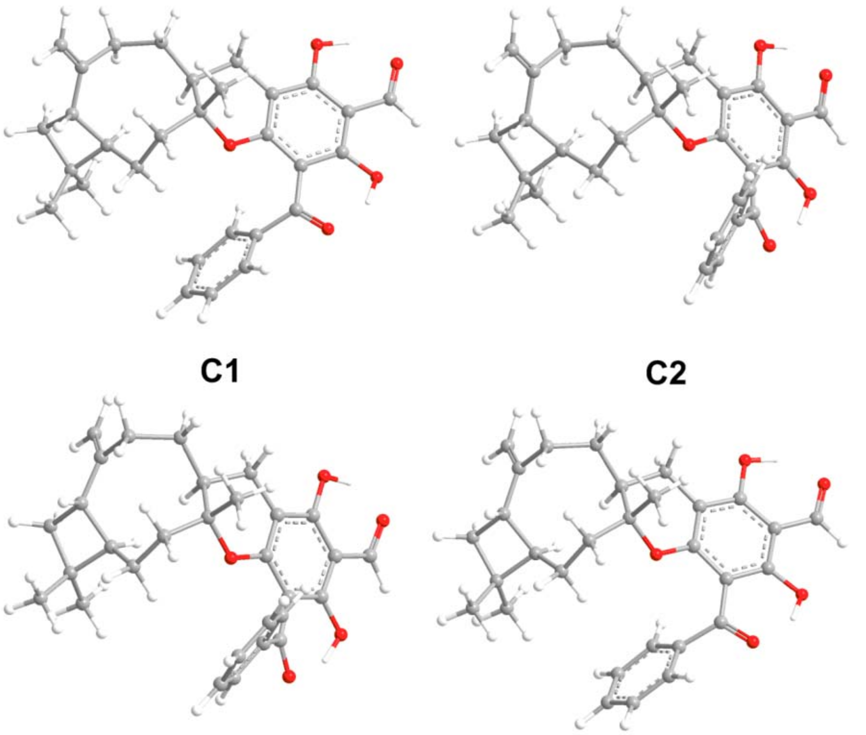

C1 C2 C3 C4

Figure S4.4. B3LYP/6-31+G(d) optimized lowest energy 3D conformers of **4**.

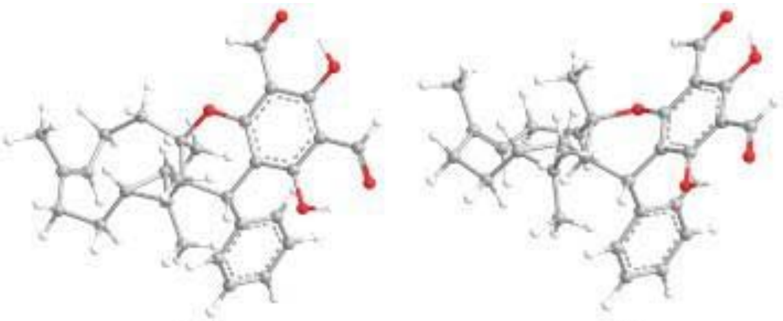

C1 C2

Figure S4.5. B3LYP/6-31G(d) optimized lowest energy 3D conformers of **12**.

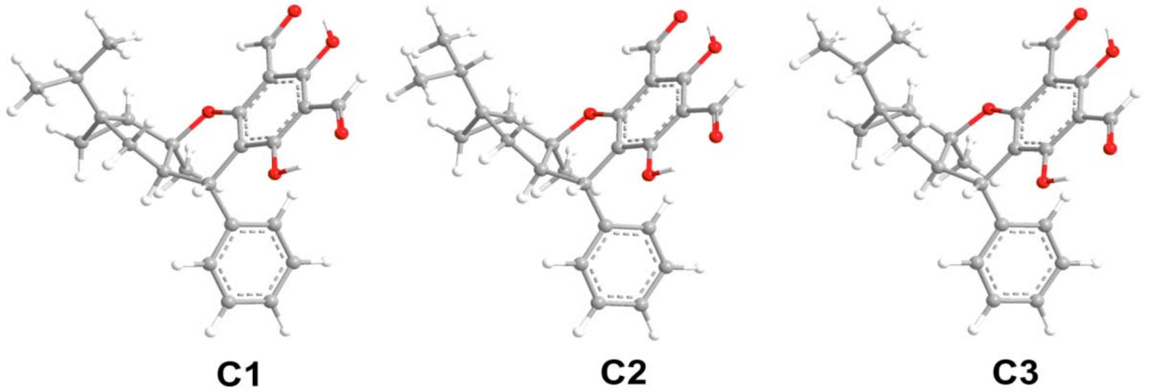

C1 C2 C3

Figure S4.6. B3LYP/6-31+G(d) optimized lowest energy 3D conformers of **25**.

S4.4. Energy analysis table:

conf.	Gibbs free energy (298.15 K)		
	G (Hartree)	ΔG (Kcal/mol)	Boltzmann Distribution
**1C1**	-1539.311911	0	0.773
**1C2**	-1539.310026	1.18285635	0.105
**1C3**	-1539.310169	1.09312242	0.122
**2C1**	-1539.274754	0.17884035	0.312919
**2C2**	-1539.274593	0.27986946	0.263839
**2C3**	-1539.275039	0	0.423242
**3C1**	-1539.293615	1.91829807	0.028
**3C2**	-1539.295626	0.65637546	0.232
**3C3**	-1539.296672	0	0.702
**3C4**	-1539.293931	1.72000491	0.038
**12C1**	-1539.224038	1.24121478	0.109486
**12C2**	-1539.226016	0	0.890514
**25C1**	-1344.076050	0	0.747
**25C2**	-1344.074930	0.70281120	0.228
**25C3**	-1344.072846	2.01054204	0.025

S4.5. ECD data of compounds 1–4, 12, and 25:

S4.5.1. ECD data table for **1**:

State	**1C1**		**1C2**		**1C3**	
	Excitation energies(eV)	Rotatory Strengths*	Excitation energies(eV)	Rotatory Strengths*	Excitation energies(eV)	Rotatory Strengths*
1	3.7657	2.6069	3.7662	2.4982	3.7659	2.7025
2	3.994	0.0355	3.9934	0.1792	3.9943	0.6171
3	4.0007	1.8407	4.0004	2.5432	4.0004	0.9353
4	4.0848	2.2091	4.0856	1.5961	4.0845	2.4714
5	4.2589	3.0268	4.259	3.1091	4.2592	2.7045
6	4.39	-1.1249	4.3903	-0.9252	4.3902	2.3408
7	4.505	1.1076	4.5054	1.1163	4.5051	1.1894
8	4.5431	1.7906	4.5435	1.7734	4.5422	1.8455
9	4.6647	25.6476	4.6649	28.5728	4.6649	22.5965
10	4.7637	4.3732	4.7644	4.447	4.7626	4.1244
11	4.7934	4.3666	4.7937	4.3949	4.7907	4.9753
12	4.8282	2.9011	4.8308	3.2061	4.8303	2.5585
13	4.8525	-1.5893	4.8616	-1.5055	4.8576	-1.4707
14	5.0048	-0.0852	4.9605	-0.1453	4.9488	-0.1057
15	5.1009	0.1692	5.1134	0.2006	5.1079	0.1235

16	5.1864	-2.2546	5.1864	-2.2086	5.1867	-2.2502
17	5.2482	0.7198	5.218	-0.0464	5.2054	-0.0548
18	5.263	-0.6878	5.2479	0.0304	5.2487	0.0438
19	5.4207	-6.4684	5.4199	-6.3894	5.4202	-6.2939
20	5.5107	4.8886	5.5083	5.1337	5.5134	5.2035
21	5.5639	-2.6854	5.5519	-2.3475	5.5496	-1.8893
22	5.5965	-0.4364	5.5962	-0.4238	5.5961	-0.419
23	5.6708	4.6385	5.6684	4.736	5.6712	4.4639
24	5.7288	0.4213	5.7272	0.4609	5.7265	0.76
25	5.7437	16.7559	5.7424	17.8264	5.7424	17.9597
26	5.7769	0.8404	5.7884	-0.1051	5.776	0.4239
27	5.8047	-7.3723	5.8004	-6.2479	5.7981	-7.9783
28	5.8456	-2.4997	5.834	-2.8055	5.8276	-3.3549
29	5.8703	2.904	5.87	1.0686	5.8764	2.3786
30	5.93	-2.8097	5.9383	-7.2512	5.9209	-7.3804
31	5.9991	-19.3507	5.993	-9.2158	5.9927	-7.2807
32	6.0068	7.1824	5.9995	7.2043	5.9992	5.3546
33	6.0119	-0.3825	6.0202	0.2099	6.0085	-0.2206
34	6.0902	0.2306	6.0907	-0.5988	6.0718	-1.8844
35	6.0986	0.9088	6.1033	1.4874	6.095	2.5677
36	6.1506	-35.9717	6.1205	-0.0682	6.1158	-0.3536
37	6.1623	27.826	6.1393	-24.621	6.1386	-8.2845
38	6.1787	13.3298	6.1475	1.9055	6.1418	-6.3883
39	6.2023	4.6546	6.1825	31.873	6.1848	23.2773
40	6.2036	-7.1557	6.2023	3.9668	6.202	3.2164
41	6.2223	14.2865	6.2245	-29.5487	6.2141	-34.3854
42	6.246	1.5852	6.2329	26.1762	6.2273	34.4892
43	6.2478	7.4182	6.2592	8.9484	6.2546	0.0764
44	6.2809	1.4371	6.2671	3.0337	6.2634	1.2411
45	6.2841	-10.1258	6.2703	5.4319	6.2652	12.1452
46	6.2928	24.3738	6.2868	12.3615	6.278	13.6856
47	6.2964	-14.5832	6.2951	-23.6604	6.2937	-18.648
48	6.3092	-10.4307	6.3054	-10.423	6.301	0.722
49	6.3252	-5.1725	6.3262	-2.6434	6.3107	-13.3402
50	6.3471	1.7018	6.3271	-8.9863	6.3233	-21.2953
51	6.3621	-50.8927	6.3509	8.1767	6.353	-0.1714
52	6.3912	-11.4571	6.3643	-56.929	6.3654	4.0687
53	6.3938	-7.1824	6.3742	20.2418	6.369	-26.1102
54	6.399	-4.5061	6.382	-13.7924	6.3787	-8.4597
55	6.4032	-9.5103	6.3878	-1.7741	6.393	-3.8241
56	6.4155	3.225	6.4061	5.6555	6.4026	-13.8212
57	6.4272	-12.699	6.4119	-20.3395	6.4064	-16.5066
58	6.4386	0.2079	6.4132	-5.5549	6.4297	7.9013

59	6.4459	-1.6264	6.4364	2.2743	6.4327	1.516
60	6.4524	-1.5111	6.4433	-2.6655	6.4415	3.5435

* R(velocity) 10**-40 erg-esu-cm

S4.5.2. ECD data table for **2**:

State	**2C1**		**2C2**		**2C3**	
	Excitation energies(eV)	Rotatory Strengths*	Excitation energies(eV)	Rotatory Strengths*	Excitation energies(eV)	Rotatory Strengths*
1	3.7682	12.2091	3.7682	12.2824	3.7687	12.0863
2	4.0191	-7.0231	4.0191	-7.0368	4.0193	-6.4463
3	4.0267	-2.2111	4.0267	-2.2188	4.0267	-2.295
4	4.0743	10.2419	4.0744	10.2565	4.0749	9.8527
5	4.3076	5.8546	4.3081	5.7658	4.3073	5.9286
6	4.4081	16.9438	4.4087	16.2292	4.4089	16.1192
7	4.5577	-0.4359	4.5582	-0.469	4.5572	-0.3574
8	4.5818	2.747	4.5825	2.6448	4.5817	2.7059
9	4.6755	16.4967	4.676	15.4658	4.6762	15.9008
10	4.8029	0.9987	4.8034	0.9804	4.8025	1.0265
11	4.8122	3.4334	4.8125	3.6721	4.8121	3.2008
12	4.8388	6.4961	4.84	6.3649	4.8386	6.901
13	4.8643	-12.9407	4.8645	-12.1923	4.864	-12.4626
14	5.0908	-0.5902	5.0937	-0.6939	5.0912	-0.7184
15	5.1587	-7.6928	5.1576	-8.7896	5.1671	-7.3089
16	5.1736	-5.6063	5.1736	-5.6436	5.1746	-6.2128
17	5.2563	2.4903	5.2559	2.5258	5.2562	2.5469
18	5.385	-3.4012	5.3845	-2.86	5.386	-4.0026
19	5.4013	1.8917	5.3991	1.7526	5.4081	2.5089
20	5.5902	-2.1264	5.5882	-2.8279	5.5953	-1.2402
21	5.5962	-0.5765	5.5958	0.0972	5.601	-1.8767
22	5.6092	-0.7321	5.6092	-0.7624	5.6103	-1.0002
23	5.7178	-1.3952	5.7175	-1.5225	5.7186	-1.3763
24	5.7419	1.6183	5.7428	1.634	5.7444	1.6063
25	5.7805	-6.1515	5.7768	-6.4437	5.7778	-6.0609
26	5.7995	14.2051	5.7967	13.4864	5.7947	14.3272
27	5.843	3.0208	5.8221	2.9207	5.8403	3.3169
28	5.8753	-0.5935	5.8714	-0.8782	5.8773	0.4153
29	5.881	-4.9282	5.886	-6.2352	5.8955	-6.1388
30	5.894	-2.7553	5.9031	-0.7513	5.9198	-0.959
31	5.9552	3.3466	5.9494	2.3583	5.9649	1.8234
32	6.0319	-7.9299	6.0321	-7.7892	6.0315	-7.1488
33	6.0527	-2.0165	6.0519	-1.5139	6.0595	-2.3613
34	6.081	-1.0532	6.0604	0.5654	6.0719	-2.2687

35	6.0846	0.444	6.0701	-1.8197	6.079	0.313
36	6.1414	0.9232	6.1524	-52.5612	6.1633	-71.3868
37	6.1663	-69.6034	6.1618	-0.2453	6.1708	2.0932
38	6.1839	-31.7543	6.1793	-36.1192	6.1794	11.6605
39	6.1942	34.9059	6.1895	31.3111	6.1911	6.1837
40	6.2126	-0.0704	6.2131	0.1568	6.2105	-1.1085
41	6.2239	-10.6351	6.2188	-11.5134	6.2329	-2.8428
42	6.2372	3.2	6.2372	-0.3731	6.2368	-14.37
43	6.2499	0.6965	6.2449	9.6536	6.2477	0.1644
44	6.2558	24.563	6.2554	-4.5234	6.2714	22.991
45	6.2707	18.811	6.2727	54.6061	6.2849	38.8086
46	6.2788	11.3897	6.2816	-1.4215	6.2959	3.926
47	6.3267	-18.1788	6.3258	-7.0291	6.3288	-25.3513
48	6.3396	-11.4565	6.3315	-38.2764	6.3451	-16.483
49	6.349	-7.1045	6.3413	-12.2255	6.3574	-4.2088
50	6.3576	-0.5807	6.3594	-0.5616	6.3597	-15.9917
51	6.3764	-8.4659	6.3838	-13.2072	6.3788	-11.4791
52	6.384	-0.5617	6.3878	0.1273	6.3846	-0.8832
53	6.4016	6.2861	6.3951	13.6356	6.3968	8.7769
54	6.4162	-66.8091	6.4212	-72.2245	6.4194	-65.6179
55	6.4452	6.6768	6.4502	9.7811	6.4539	4.6525
56	6.4524	5.8011	6.4565	8.7028	6.464	17.2542
57	6.4705	9.8889	6.465	0.0785	6.4755	19.5788
58	6.4744	-7.2217	6.4729	2.2973	6.4843	-24.05
59	6.4745	-1.6405	6.4779	-10.183	6.4869	-4.9237
60	6.482	-0.7893	6.4837	10.8887	6.4938	2.4122

* R(velocity) 10**-40 erg-esu-cm

S4.5.3. ECD data table for **3**:

**3**								
State	Excitation energies(eV)	Rotatory Strengths*	State	Excitation energies(eV)	Rotatory Strengths*	State	Excitation energies(eV)	Rotatory Strengths*
1	3.6352	13.8825	21	5.2777	1.2336	41	5.966	-6.0923
2	3.9103	7.115	22	5.307	7.3795	42	5.975	1.9398
3	3.9652	-18.0959	23	5.4808	-8.3398	43	5.9956	-1.8026
4	3.9863	1.4883	24	5.4912	-38.2721	44	6.0121	-3.2144
5	4.0197	1.4	25	5.5391	-4.0497	45	6.027	2.3937
6	4.15	-8.9806	26	5.5569	2.0044	46	6.0663	1.4009
7	4.255	-9.1311	27	5.6054	5.6991	47	6.0719	-0.6789
8	4.3061	-10.0501	28	5.6142	2.738	48	6.1034	25.4888
9	4.3992	49.202	29	5.6691	-3.4294	49	6.1137	-7.4824
10	4.5102	-0.4859	30	5.747	16.9928	50	6.1546	19.4935

11	4.6535	-1.0698	31	5.7642	-17.2934	51	6.1882	0.6444
12	4.6819	18.3442	32	5.7902	10.9682	52	6.197	-23.5966
13	4.7151	-0.1137	33	5.7923	3.5735	53	6.2151	-40.8055
14	4.7584	-9.0928	34	5.8096	8.9036	54	6.2196	-9.4117
15	4.8052	-13.181	35	5.8298	-8.281	55	6.243	1.5352
16	4.8825	-3.211	36	5.8721	0.2391	56	6.2486	-3.4556
17	5.0537	0.4923	37	5.925	16.4278	57	6.2658	-7.991
18	5.1003	2.3006	38	5.9467	-0.8006	58	6.2696	-4.9825
19	5.1475	4.9097	39	5.9525	-13.0017	59	6.2733	9.8106
20	5.1709	9.0422	40	5.9613	-23.9791	60	6.2848	12.4785

* R(velocity) 10**-40 erg-esu-cm

S4.5.4. ECD data table for **4**:

State	**4C1**		**4C2**		**4C3**	
	Excitation energies(eV)	Rotatory Strengths*	Excitation energies(eV)	Rotatory Strengths*	Excitation energies(eV)	Rotatory Strengths*
1	3.5619	-12.0057	3.5933	-5.2855	3.58	-4.8726
2	3.8394	20.1945	3.8857	-5.7483	3.8746	-7.4354
3	3.8864	53.8579	3.9265	-50.5996	3.9272	-51.2718
4	3.9969	83.9328	4.0077	-52.6437	4.0083	-47.7624
5	3.9985	-137.917	4.0238	64.2451	4.0246	53.9166
6	4.2537	-0.6744	4.3031	-1.372	4.3094	-0.3598
7	4.4743	44.9113	4.4789	-30.1913	4.4889	-4.7228
8	4.4818	-59.9465	4.4877	68.9042	4.4944	52.1904
9	4.5617	24.051	4.5607	-15.1041	4.563	-16.3541
10	4.6051	-0.5058	4.6236	1.619	4.6259	0.5885
11	4.7243	4.2313	4.7318	-3.7587	4.7334	-0.2791
12	4.8175	-7.1435	4.8	9.9817	4.8157	8.0777
13	4.9799	4.1856	4.9481	-10.8416	4.9709	-8.9567
14	5.0693	0.4994	5.0933	11.5851	5.0548	9.4207
15	5.1181	-15.0568	5.1451	-1.5347	5.117	0.0326
16	5.1442	5.5988	5.1713	0.9542	5.1892	0.4053
17	5.2427	5.0149	5.3002	-0.503	5.2583	1.5617
18	5.3317	1.0507	5.3443	5.5672	5.3143	3.21
19	5.3488	2.7845	5.3925	-2.9587	5.373	-5.1305
20	5.3846	-17.2199	5.4076	3.3893	5.3833	0.8689
21	5.4122	-4.2615	5.4391	0.4737	5.4329	12.7358
22	5.4666	8.0582	5.5004	4.4002	5.5131	-1.7266
23	5.5385	1.0534	5.5301	9.0392	5.5277	9.2358
24	5.5498	-2.1082	5.5326	-0.3043	5.5773	0.2681
25	5.595	15.8445	5.6278	-1.5497	5.6047	-1.5025
26	5.6471	1.447	5.6721	-4.8368	5.6818	1.4496

27	5.6735	-2.0907	5.7126	-0.7439	5.6909	-3.2183
28	5.7087	-8.0677	5.719	3.1579	5.7144	-4.3812
29	5.7171	28.2665	5.7323	-19.6784	5.7191	1.9845
30	5.7213	1.1973	5.7461	-1.9544	5.7423	-2.8478
31	5.7375	5.2896	5.7491	2.1652	5.7488	-0.7848
32	5.751	-15.159	5.7724	3.5459	5.7513	1.5395
33	5.7808	-7.3915	5.778	-1.9592	5.7623	2.5538
34	5.7926	-14.4793	5.792	28.633	5.7872	4.6404
35	5.8418	-7.1361	5.8274	1.6918	5.8526	-0.5635
36	5.8434	17.3068	5.8316	-1.1943	5.8935	-0.8805
37	5.8679	-23.4812	5.8455	-3.9857	5.9163	-0.309
38	5.8821	11.2308	5.8894	-13.9053	5.9447	-19.2659
39	5.9249	8.4972	5.9597	-17.2969	5.9932	9.9484
40	5.9345	2.9554	5.9699	-0.9599	6.0005	-12.0662
41	5.9701	0.2677	6.0088	-15.2067	6.0067	-0.0008
42	6.0003	1.7801	6.0235	0.5293	6.0273	0.7828
43	6.0025	7.4718	6.0617	1.8908	6.0615	-1.3685
44	6.0831	1.6923	6.0731	-3.7569	6.0742	-4.7429
45	6.1003	1.7721	6.1106	0.1542	6.0927	-12.1016
46	6.1475	7.0615	6.1149	-1.7009	6.1125	-4.7257
47	6.1545	5.6922	6.1187	-2.2028	6.1203	3.9806
48	6.1582	0.4314	6.1348	-2.9361	6.146	-0.6725
49	6.1673	10.7959	6.1434	1.6917	6.1543	-3.5661
50	6.1865	-16.6319	6.176	-9.3202	6.1657	-1.9958
51	6.1956	-0.5618	6.1955	4.481	6.1848	13.2987
52	6.2155	73.533	6.2126	-0.8856	6.1941	-3.0873
53	6.239	39.2097	6.2303	-17.2166	6.2107	-22.0616
54	6.242	-38.3535	6.2461	-17.1971	6.2207	17.8733
55	6.2573	12.1983	6.2761	60.6619	6.2571	-70.244
56	6.2757	-12.1952	6.2884	-23.0631	6.2731	-58.734
57	6.2847	57.9165	6.295	-59.2007	6.2896	-1.9582
58	6.2879	-49.4276	6.3094	-0.9216	6.3091	43.4603
59	6.3055	-6.3244	6.3154	-40.9419	6.3601	7.2797
60	6.3083	-8.6541	6.3379	49.2061	6.3769	-60.7561

* R(velocity) 10**-40 erg-esu-cm

S4.5.5. ECD data table for **4**: (continued)

**4C4**								
State	Excitation energies(eV)	Rotatory Strengths*	State	Excitation energies(eV)	Rotatory Strengths*	State	Excitation energies(eV)	Rotatory Strengths*
1	3.5488	-12.2344	21	5.4247	0.5359	41	6.0099	8.309
2	3.8289	11.3107	22	5.5111	-0.0212	42	6.0126	2.1821
3	3.8875	58.4086	23	5.5425	1.4568	43	6.0661	-1.9947

4	3.9936	47.9284	24	5.5605	-4.994	44	6.0826	-17.9577
5	4.0023	-104.135	25	5.5838	20.9202	45	6.0978	2.7412
6	4.2784	0.1715	26	5.6736	0.3698	46	6.1152	-1.9381
7	4.4747	25.4546	27	5.6815	-0.1907	47	6.1519	1.0877
8	4.4847	-25.1219	28	5.6912	1.3807	48	6.1631	1.7678
9	4.5585	25.7329	29	5.7188	-6.4529	49	6.1654	7.3635
10	4.624	-0.6437	30	5.7293	10.5223	50	6.1904	-18.5183
11	4.7204	3.1607	31	5.7414	-2.0537	51	6.199	-0.473
12	4.8142	-5.8831	32	5.7642	-3.9435	52	6.2174	20.5232
13	4.9837	2.8862	33	5.794	-7.0539	53	6.2279	12.8958
14	5.0531	-0.1973	34	5.8217	-0.7614	54	6.2534	62.6303
15	5.1119	-11.1112	35	5.8633	-4.0037	55	6.2639	-11.6024
16	5.1431	5.8769	36	5.8905	-8.7485	56	6.2918	-0.3937
17	5.218	3.5281	37	5.9237	-0.2875	57	6.3042	7.8994
18	5.3222	1.4728	38	5.9312	18.1355	58	6.3089	-7.1466
19	5.3439	2.7241	39	5.9349	17.3027	59	6.3171	-7.8661
20	5.3807	-5.6758	40	5.9737	-3.2621	60	6.3523	3.5958

* R(velocity) 10**-40 erg-esu-cm

S4.5.6. ECD data table for **12**:

State	**12C1**		**12C2**	
	Excitation energies(eV)	Rotatory Strengths*	Excitation energies(eV)	Rotatory Strengths*
1	3.7659	-5.3352	3.7015	-10.1121
2	4.0063	-3.1167	3.9555	19.8379
3	4.0191	-7.9343	3.9885	7.9197
4	4.0658	-0.2195	4.0299	-6.8052
5	4.0801	0.1655	4.0523	-4.7795
6	4.1922	0.9859	4.1275	-7.7002
7	4.2918	0.0678	4.2878	-0.4488
8	4.4126	7.492	4.4079	-1.8171
9	4.4708	-4.2135	4.4229	1.911
10	4.4974	-3.0661	4.4661	-47.2017
11	4.6749	28.5355	4.6549	1.2978
12	4.754	-6.066	4.6758	9.9104
13	4.7733	-4.6183	4.7088	18.6593
14	4.8195	-3.174	4.7919	-3.8346
15	4.8531	-5.7414	4.8246	15.7648
16	4.9698	0.0605	4.9121	5.3827
17	5.036	-0.3647	4.9463	1.8532
18	5.1759	-0.3123	5.1702	5.5758
19	5.2374	-0.8028	5.1849	-0.2141

20	5.2936	0.531	5.29	-4.1254
21	5.3964	16.1259	5.3828	1.3252
22	5.4219	-0.2583	5.4033	1.8447
23	5.436	1.3008	5.4334	10.4202
24	5.5597	26.8486	5.5006	-1.6493
25	5.5693	3.7474	5.5719	-0.4658
26	5.5878	-54.449	5.6048	-3.4683
27	5.6012	-0.1182	5.649	0.9263
28	5.6827	5.318	5.6965	-61.2663
29	5.6856	-1.0384	5.7118	54.321
30	5.7167	-3.9729	5.7572	-8.8361
31	5.7326	4.4422	5.8008	-2.9471
32	5.7803	-6.6752	5.8139	7.8934
33	5.8031	29.8158	5.8584	17.9337
34	5.8187	-10.682	5.8813	11.9163
35	5.8823	-1.7223	5.9185	9.9515
36	5.9028	8.1426	5.9295	-0.9058
37	5.921	-0.6098	5.9417	0.6643
38	5.9394	1.2592	5.9637	-6.8298
39	5.9885	-0.6033	6.0286	-7.4721
40	6.0025	2.3879	6.0486	3.4873
41	6.0309	-1.7383	6.0567	-21.6021
42	6.0726	-0.7242	6.0697	-1.3196
43	6.0989	7.1326	6.0876	5.7061
44	6.1423	-15.3627	6.1007	22.2003
45	6.1702	-6.04	6.1268	-0.5711
46	6.1867	22.458	6.1691	18.4681
47	6.1977	44.5936	6.1972	-2.3507
48	6.2179	-84.749	6.213	-22.1727
49	6.2246	43.6596	6.2206	-9.8344
50	6.2412	-5.4584	6.2312	-12.9399
51	6.2499	0.6885	6.25	-45.6844
52	6.2563	1.4847	6.2704	3.0366
53	6.2653	5.7556	6.2793	10.1436
54	6.2682	18.2084	6.2831	-1.0561
55	6.2804	-8.3062	6.3013	-24.5811
56	6.2905	3.192	6.3038	0.1342
57	6.2945	-4.8737	6.3069	-18.0495
58	6.2996	30.5399	6.3175	16.9292
59	6.3098	45.6536	6.3223	6.4313
60	6.3164	-20.1048	6.3247	6.9393

* R(velocity) 10**-40 erg-esu-cm

S4.5.7. ECD data table for **25**:

State	**25C1**		**25C2**		**25C3**	
	Excitation energies(eV)	Rotatory Strengths*	Excitation energies(eV)	Rotatory Strengths*	Excitation energies(eV)	Rotatory Strengths*
1	3.7656	-11.0651	3.7667	-10.5207	3.7662	-10.8333
2	3.9958	2.5346	3.9972	4.4101	3.9974	3.5011
3	4.0048	1.7378	4.005	1.9168	4.005	1.9501
4	4.0778	-6.0156	4.0781	-6.9261	4.0782	-6.8116
5	4.3041	-3.8593	4.3059	-4.0842	4.3057	-4.226
6	4.4111	-20.4495	4.414	-21.5504	4.4105	-18.3347
7	4.5446	1.3653	4.5475	1.4278	4.5465	1.4025
8	4.5825	-1.5639	4.5836	-1.4742	4.5844	-1.8165
9	4.677	-7.0676	4.68	1.0783	4.6773	-10.5688
10	4.7921	-4.6769	4.7939	-4.4023	4.7945	-3.045
11	4.7952	-2.5973	4.7969	-2.396	4.7973	-3.8713
12	4.8385	4.1904	4.8383	5.0419	4.8373	4.6827
13	5.0842	0.4213	5.0877	-0.9675	5.065	0.6867
14	5.1658	5.0906	5.1655	5.2399	5.165	5.3153
15	5.2586	-2.5398	5.2576	-2.3987	5.2573	-2.4707
16	5.3449	0.3577	5.3472	-0.5187	5.3251	1.0673
17	5.3844	3.6014	5.3837	3.6894	5.3836	3.4665
18	5.4681	13.3533	5.4826	12.8741	5.4725	13.4554
19	5.6015	1.5563	5.5861	1.1842	5.5994	-0.332
20	5.6021	-0.3054	5.6014	-0.5973	5.6004	1.6439
21	5.6549	-3.9664	5.6602	-4.7101	5.6554	-4.8649
22	5.6902	-2.458	5.7052	0.672	5.704	1.0409
23	5.7153	-0.4419	5.7209	-2.7039	5.7216	-2.8612
24	5.7844	-0.5428	5.7852	-1.3425	5.7847	-1.6152
25	5.8362	0.3229	5.8314	-0.8377	5.8337	-0.3547
26	5.8825	2.1514	5.8688	2.8629	5.8804	2.9747
27	5.8976	0.3047	5.9171	4.5446	5.9212	1.0417
28	5.9177	2.2898	5.9247	-0.5509	5.9288	3.3727
29	5.9541	-4.9553	5.9596	-0.6653	5.9591	-2.1735
30	5.995	1.5877	5.9971	0.5507	5.9945	-0.1009
31	6.0636	7.0705	6.054	6.4608	6.0492	7.2025
32	6.0754	0.7361	6.0726	2.9835	6.0757	3.7406
33	6.1415	8.6259	6.1438	7.8919	6.1415	8.75
34	6.1847	6.2221	6.1871	16.4793	6.1959	30.6377
35	6.2037	9.2727	6.2036	-1.8277	6.2027	-2.09
36	6.2064	58.0991	6.2163	50.3127	6.2138	62.8703
37	6.2182	0.9186	6.2215	9.1837	6.2224	-13.5552

38	6.233	23.553	6.2543	-23.69	6.2308	13.6977
39	6.2612	-70.761	6.2634	-16.2209	6.2504	-54.4002
40	6.2672	-18.305	6.2752	-10.5346	6.2672	-6.2945
41	6.2815	-11.5392	6.2841	-38.5152	6.2786	-42.5186
42	6.31	-3.3019	6.3131	8.0513	6.3109	4.2239
43	6.3199	2.9542	6.3218	-1.914	6.3186	1.6086
44	6.3898	40.2066	6.3801	10.7008	6.3833	11.6908
45	6.4003	23.7517	6.4008	-2.0738	6.3991	0.1046
46	6.4157	1.2548	6.4028	66.0299	6.4054	63.6772
47	6.4364	1.1355	6.4365	1.997	6.4439	9.1439
48	6.4492	46.5035	6.4418	-1.7881	6.4527	12.7199
49	6.4555	0.1645	6.4479	50.8811	6.4534	-0.9474
50	6.4731	2.2221	6.4711	7.2218	6.4589	17.4362
51	6.4783	5.9758	6.4769	-2.9969	6.4706	15.0731
52	6.4818	2.1929	6.4861	-24.9619	6.4845	-13.5886
53	6.4892	-38.3511	6.4918	-10.7943	6.4895	-35.3276
54	6.4972	1.826	6.5102	-9.0086	6.5044	-0.073
55	6.5216	-1.388	6.5254	-2.24	6.5109	4.8398
56	6.5254	4.8279	6.5344	1.6018	6.5212	4.0636
57	6.5508	-5.9722	6.5665	-2.7941	6.5586	-2.5603
58	6.5697	6.0183	6.5748	1.5815	6.5757	3.438
59	6.5782	-13.1707	6.5776	-11.2076	6.5798	-6.2997
60	6.597	-13.5064	6.59	15.7647	6.5921	-21.1571

* R(velocity)   10**-40 erg-esu-cm

S5.1. ^{1}H NMR spectrum of compound **1**

In CDCl3

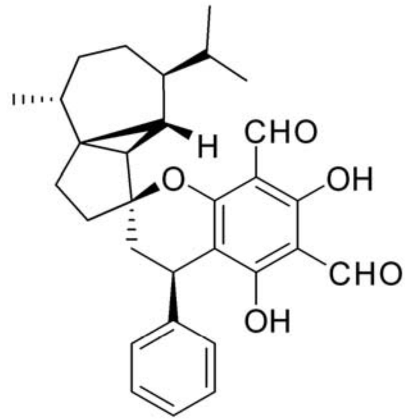

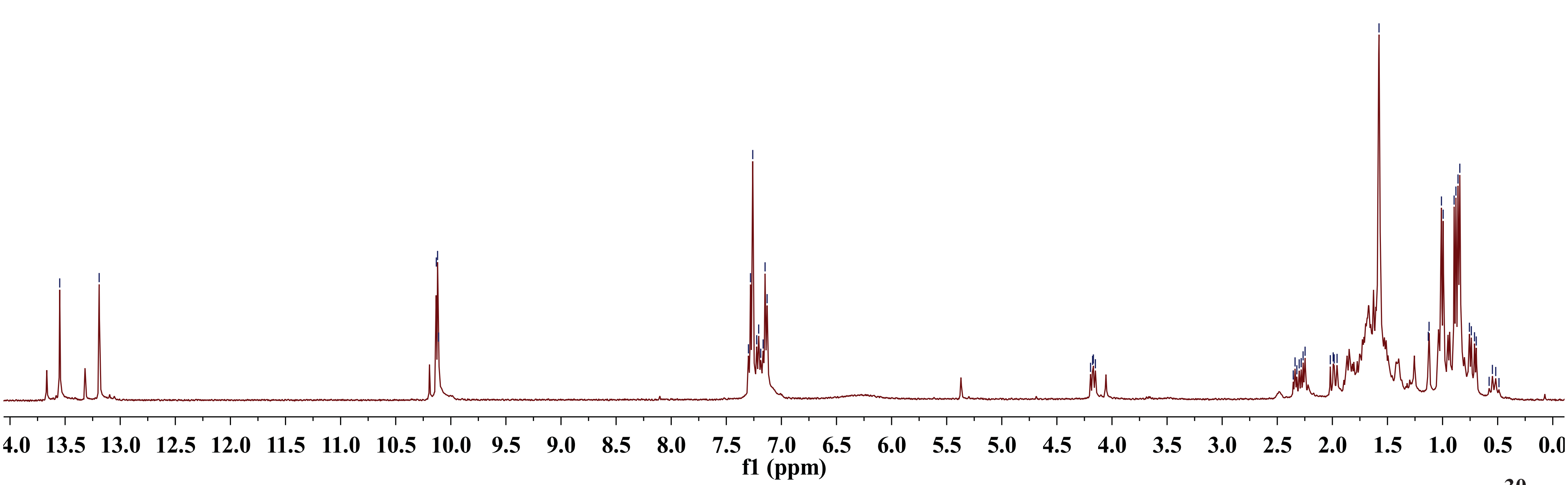

S5.2. DEPT spectra of compound **1**

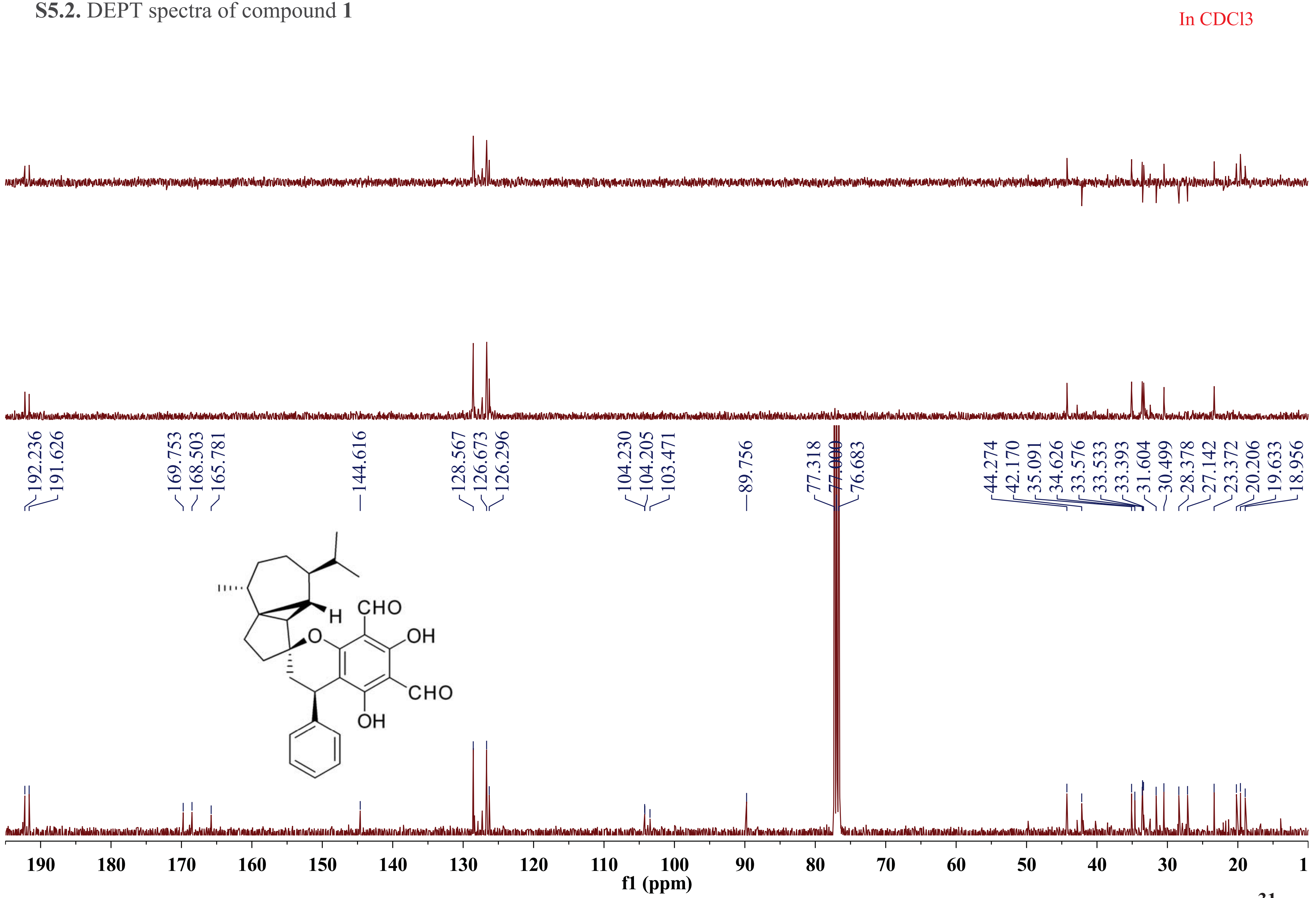

S5.3. HSQC spectrum of compound **1**

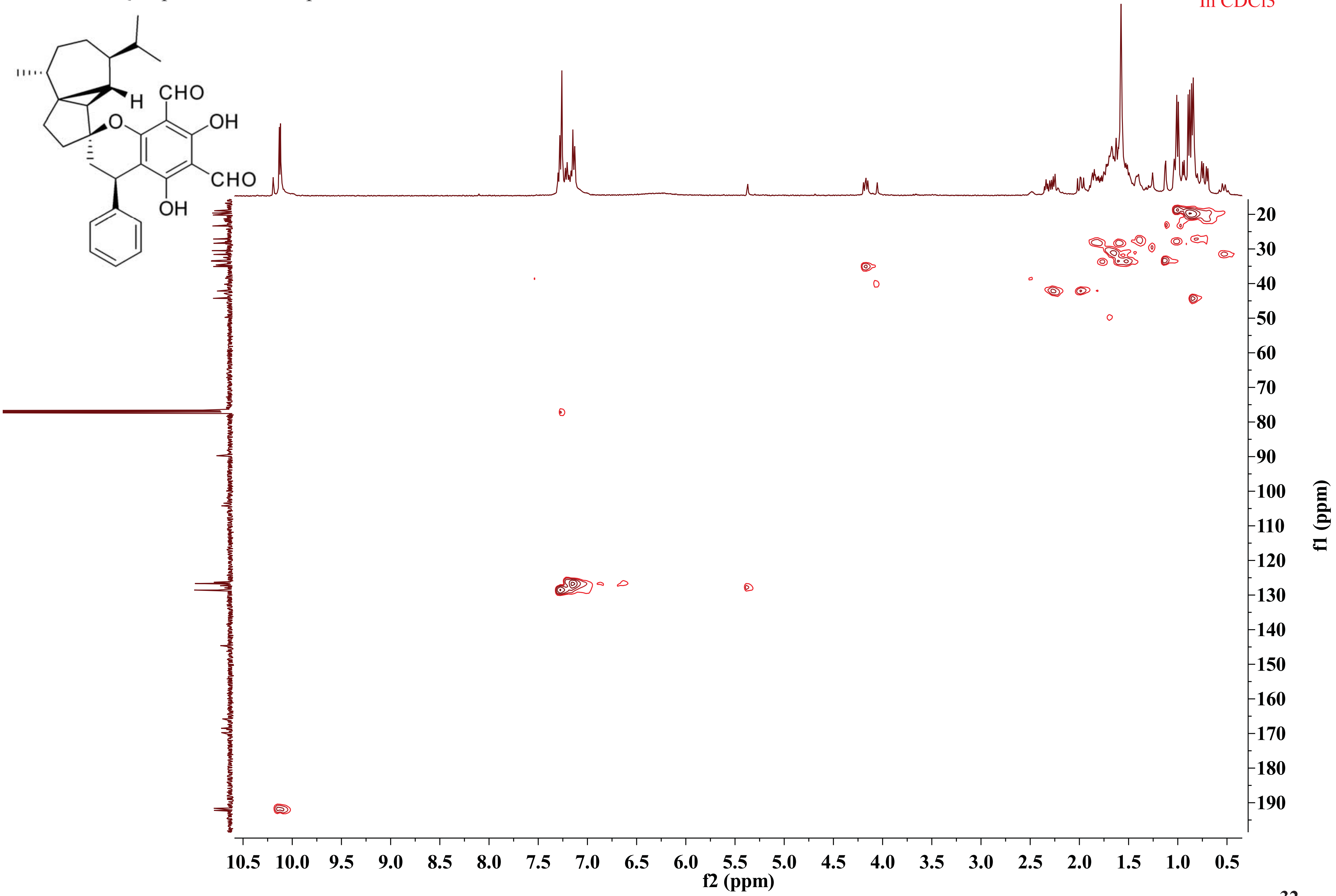

S5.4. ^{1}H-^{1}H COSY spectrum of compound **1**

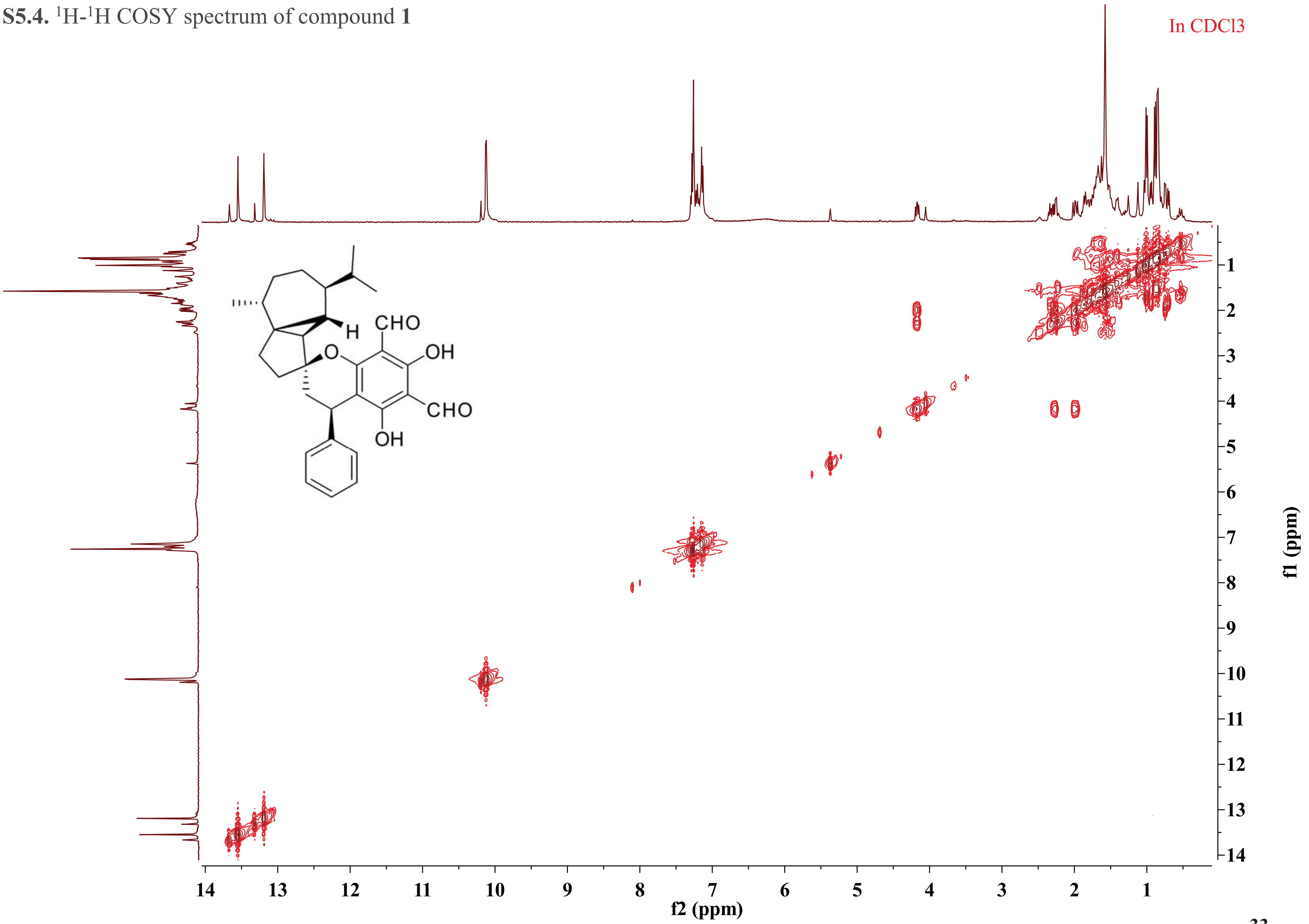

S5.5. HMBC spectrum of compound **1**

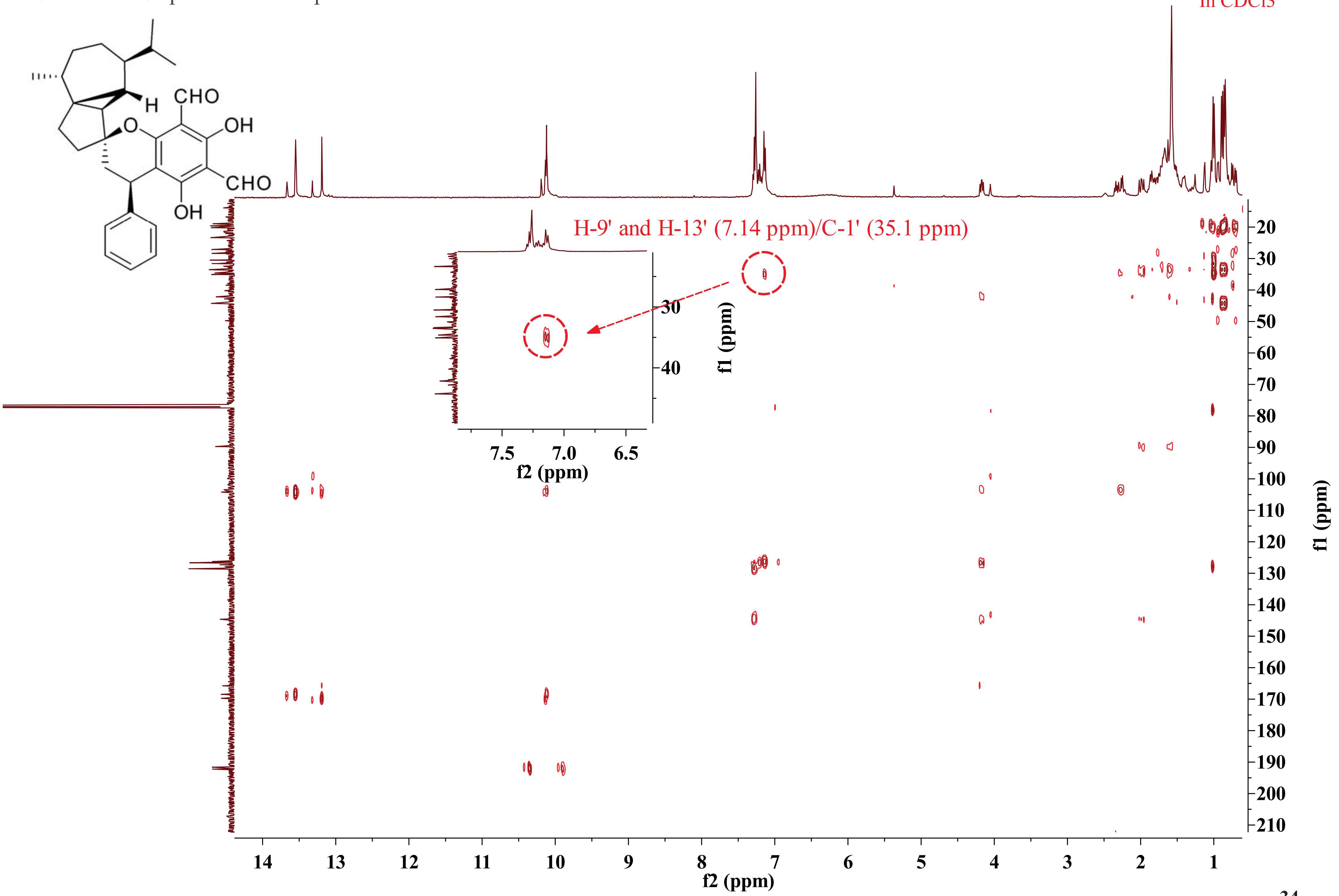

S5.6. NOESY spectrum of compound **1**

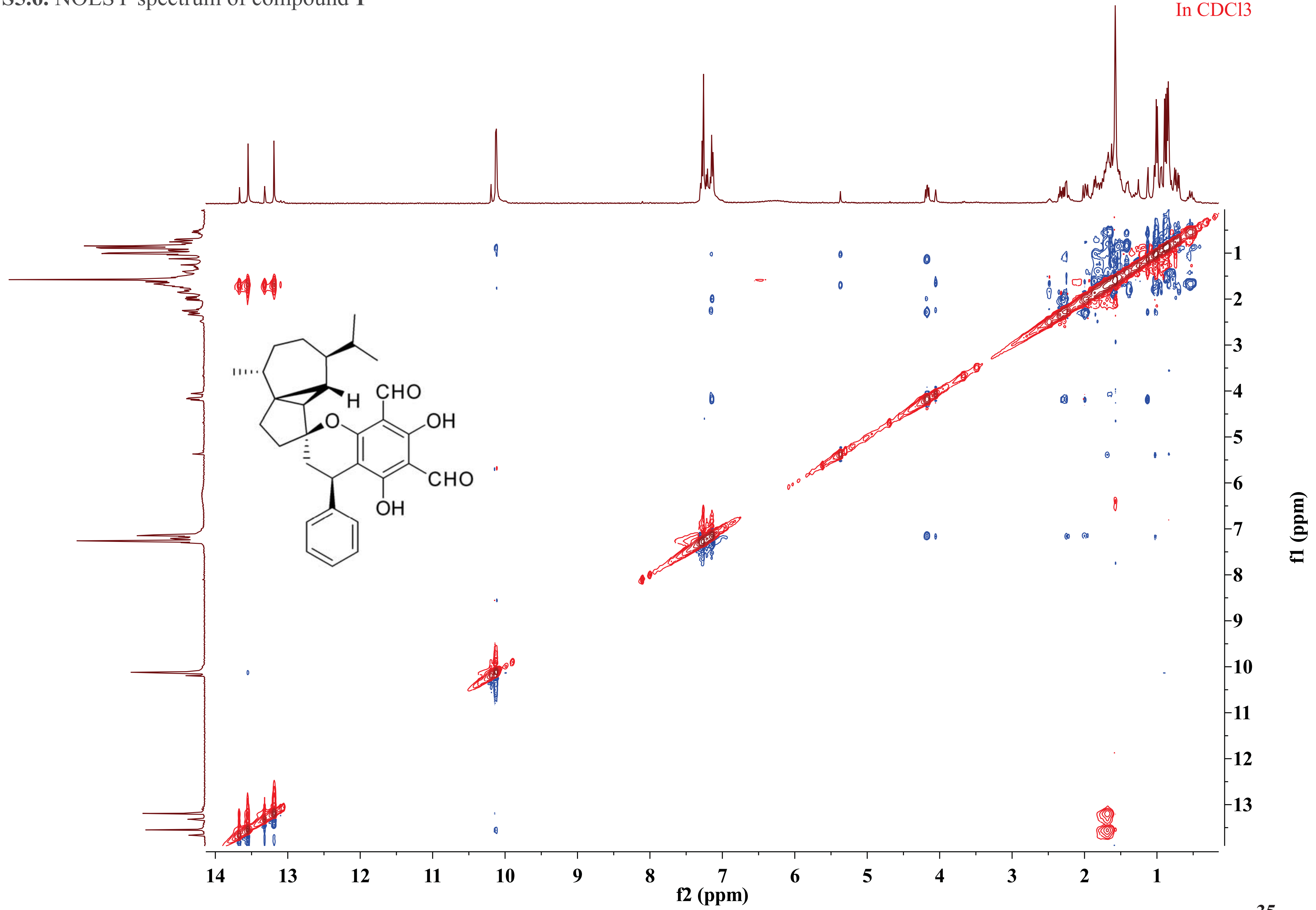

S5.7. ^{1}H NMR spectrum of compound **2**

In CDCl3

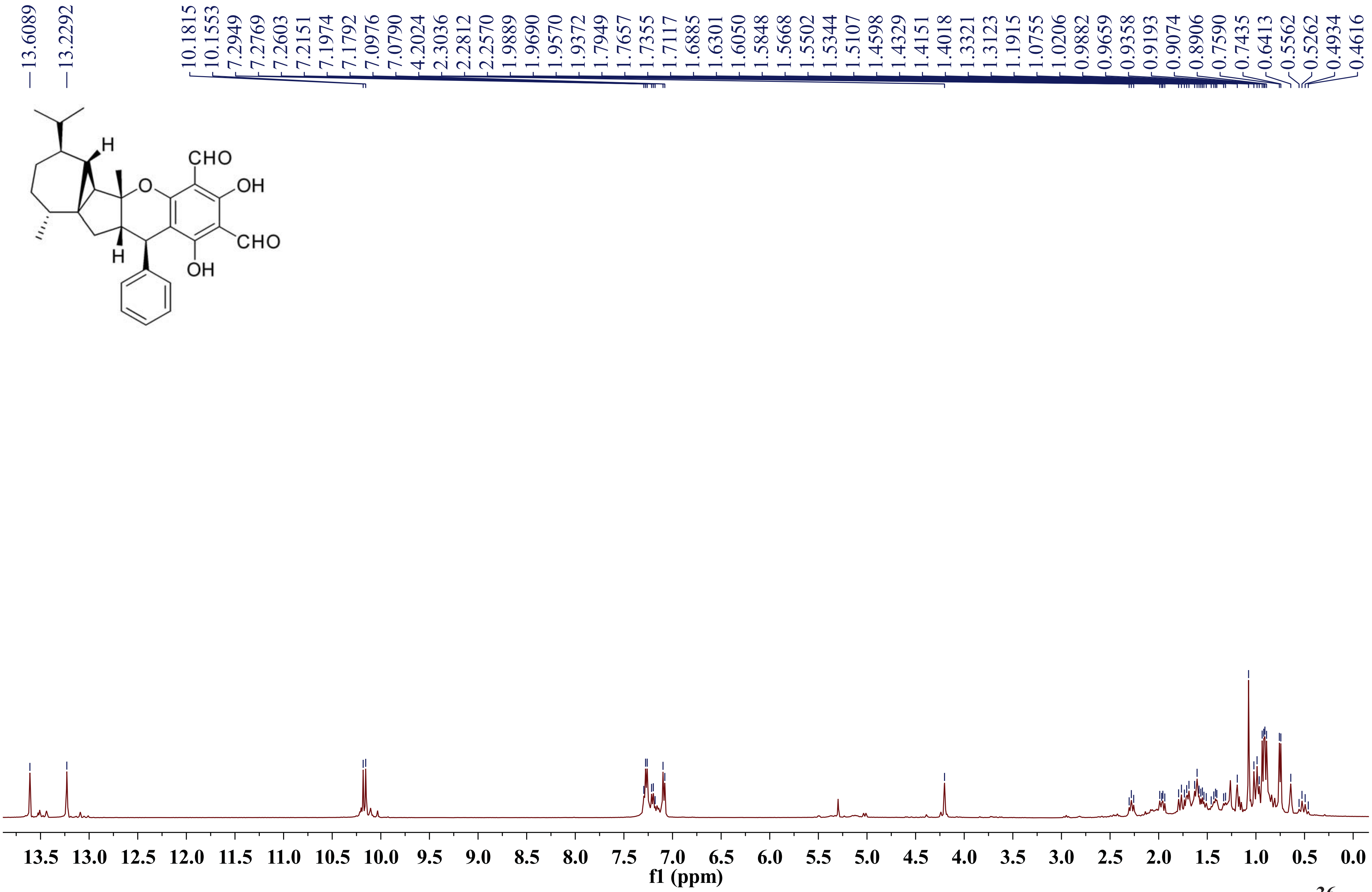

S5.8. DEPT spectra of compound **2**

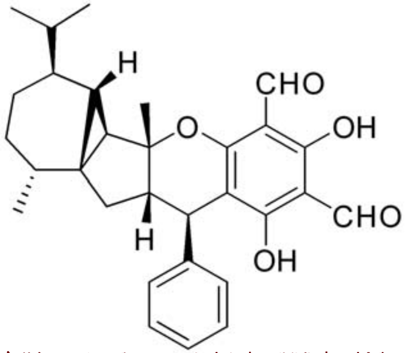

In CDCl3

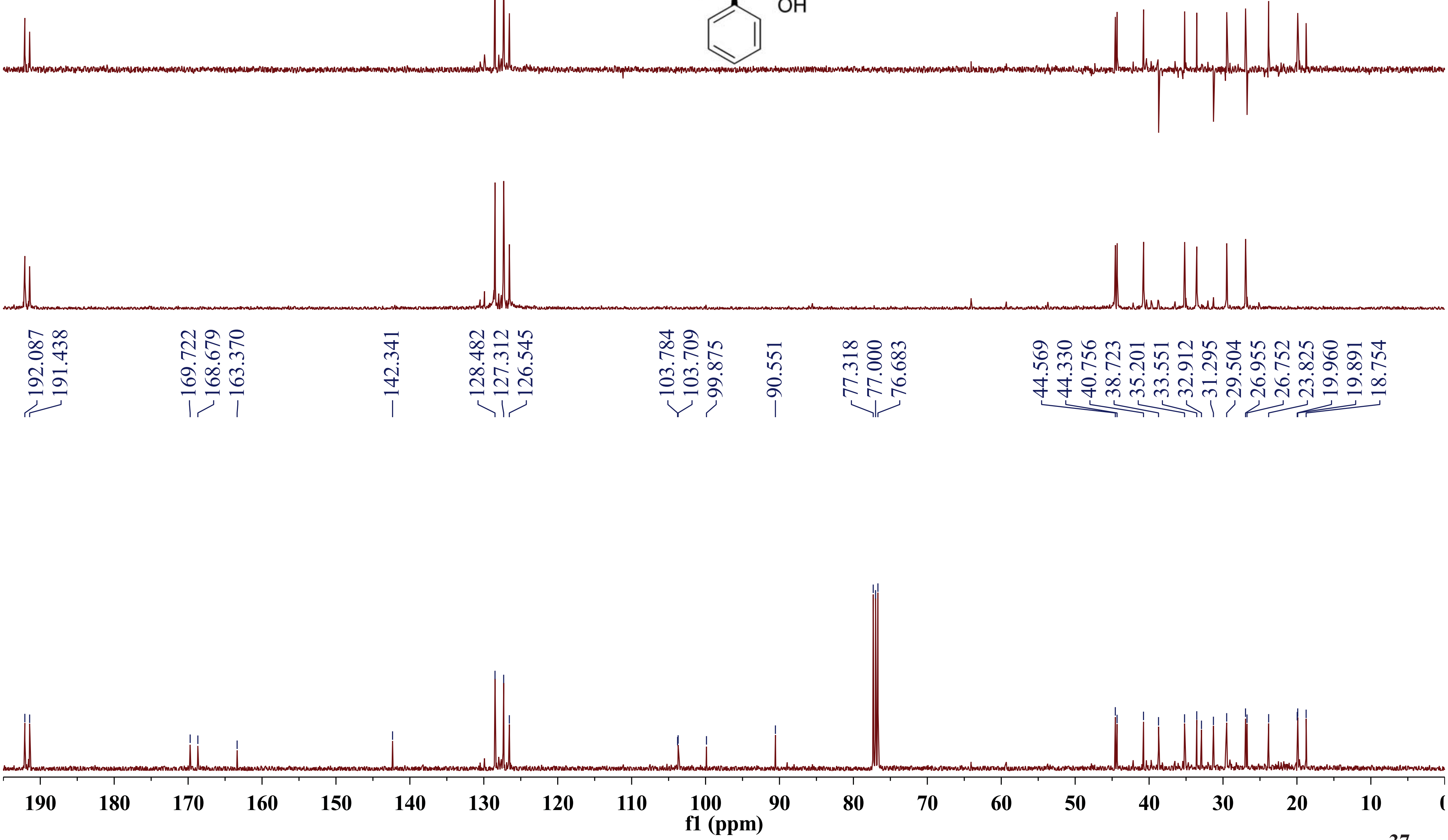

S5.9. HSQC spectrum of compound **2**

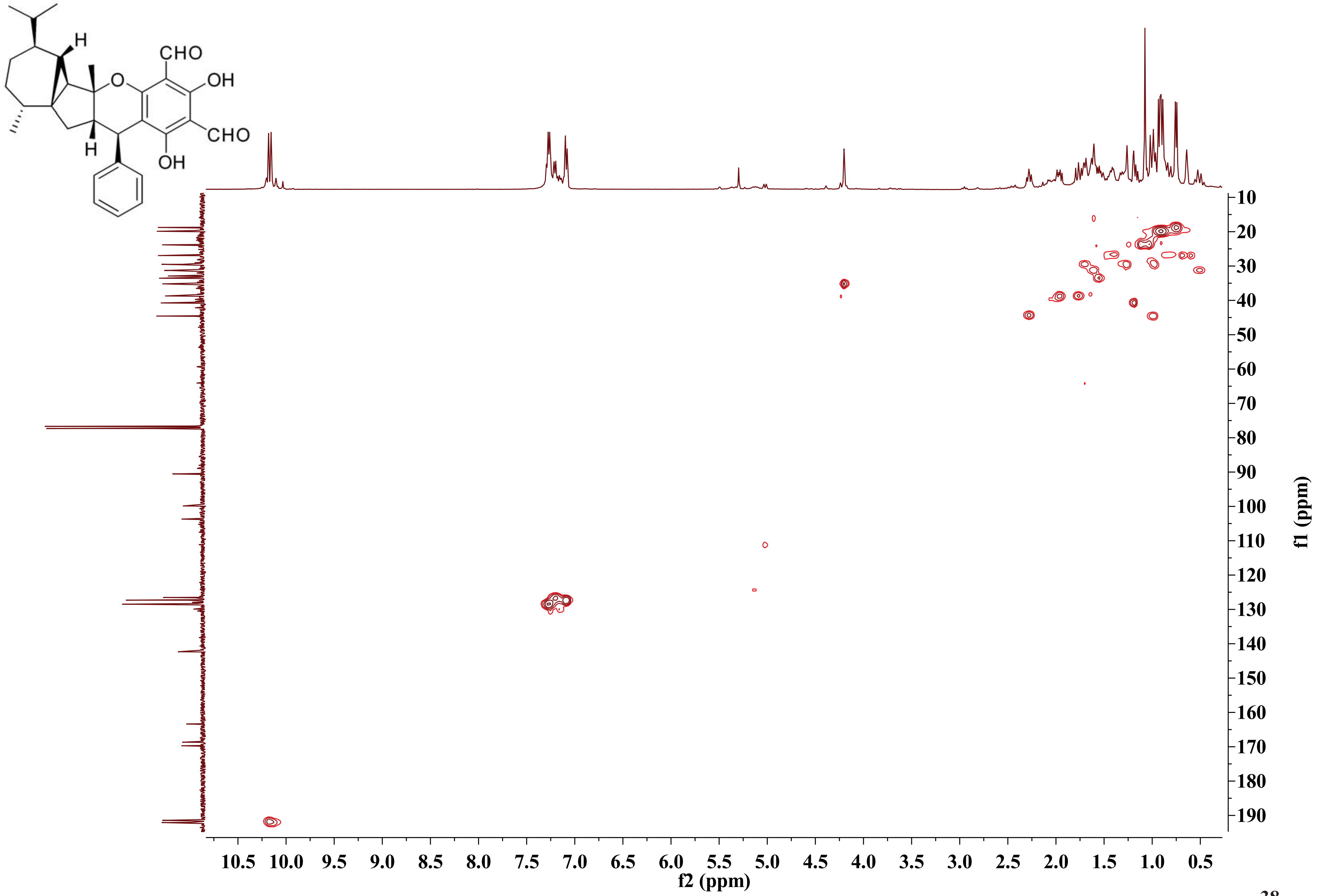

S5.10. ^{1}H-^{1}H COSY spectrum of compound **2**

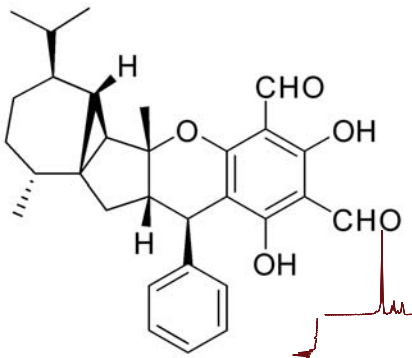

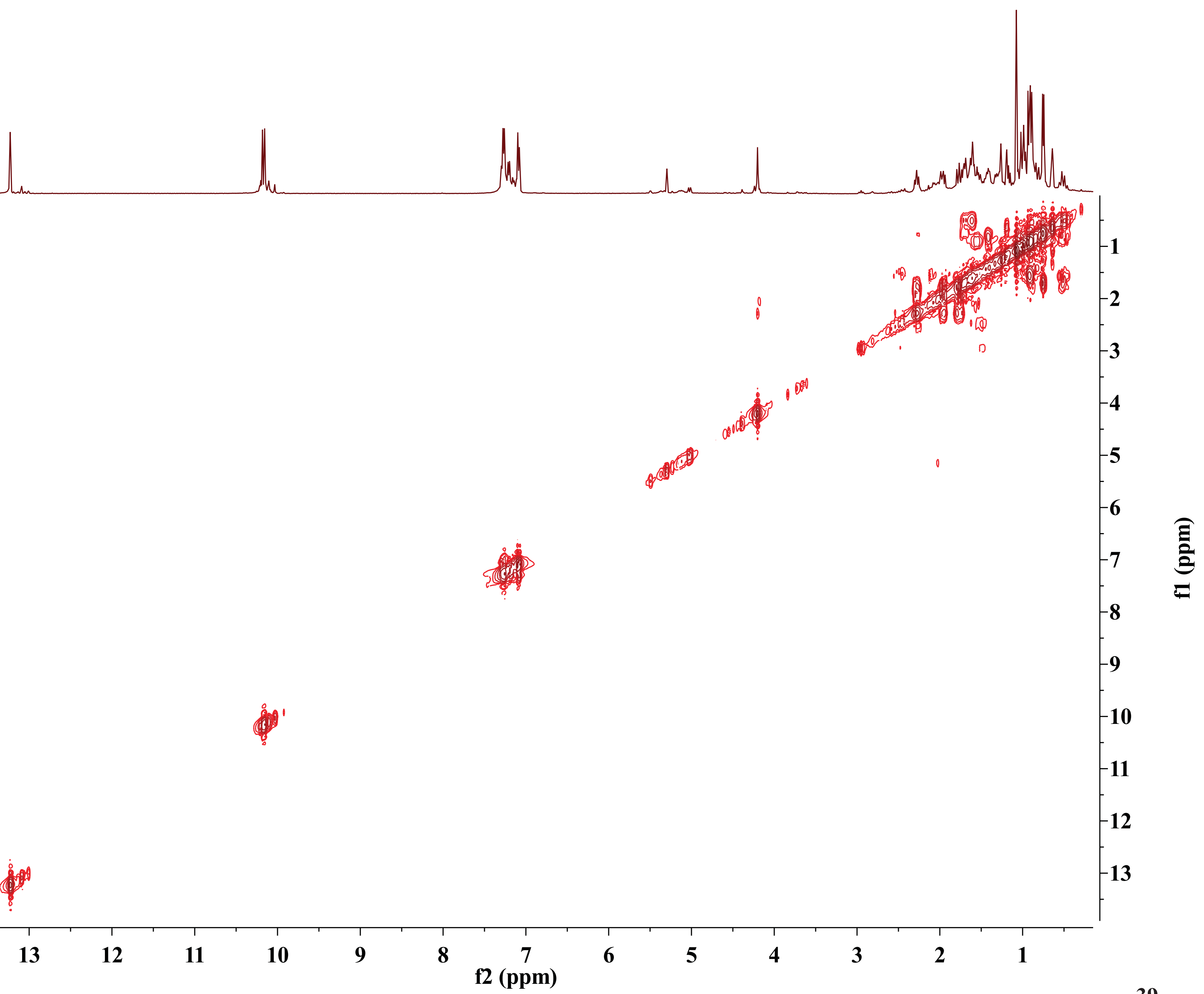

S5.11. HMBC spectrum of compound **2**

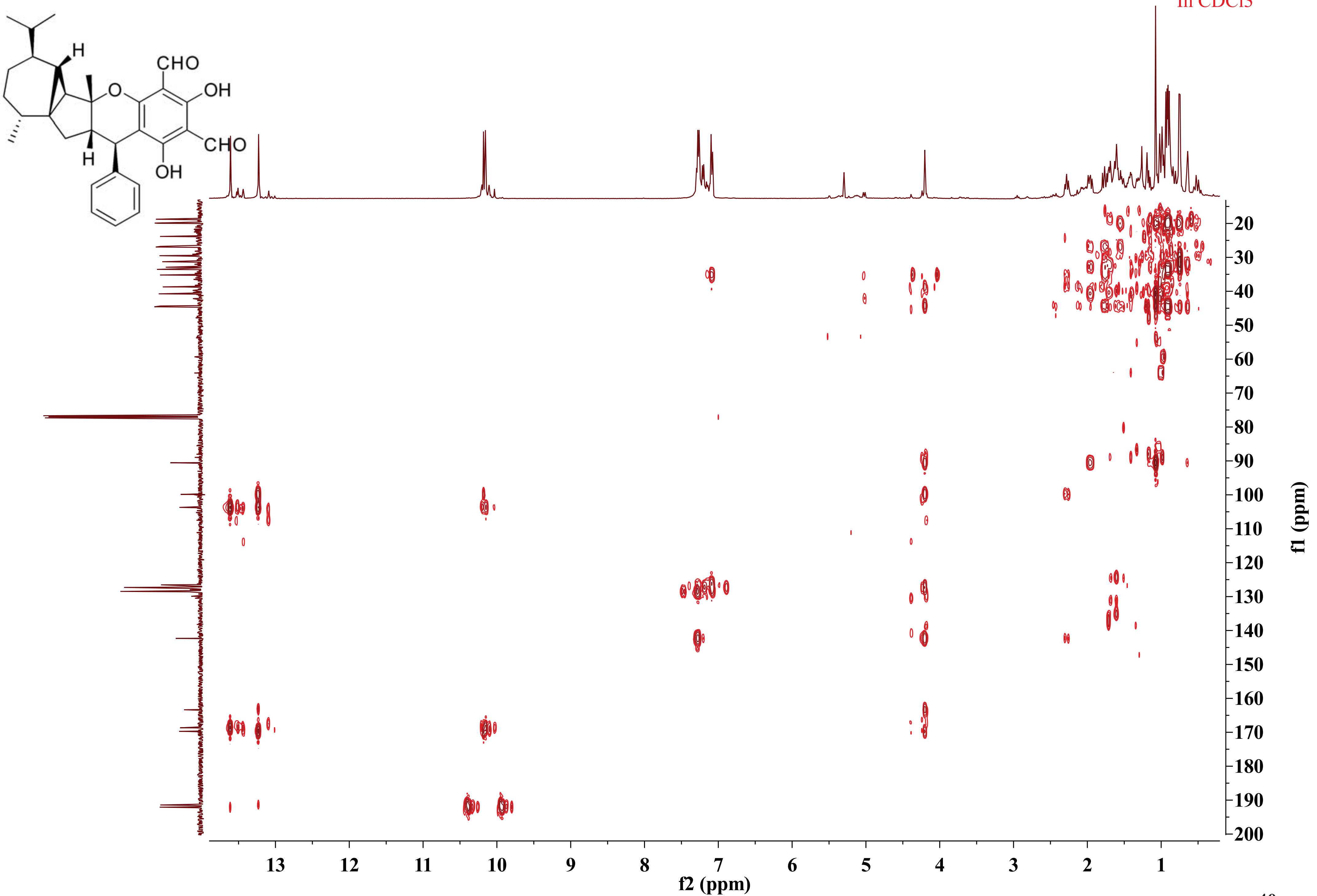

S5.12. NOESY spectrum of compound **2**

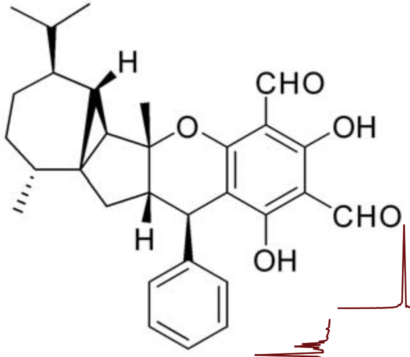

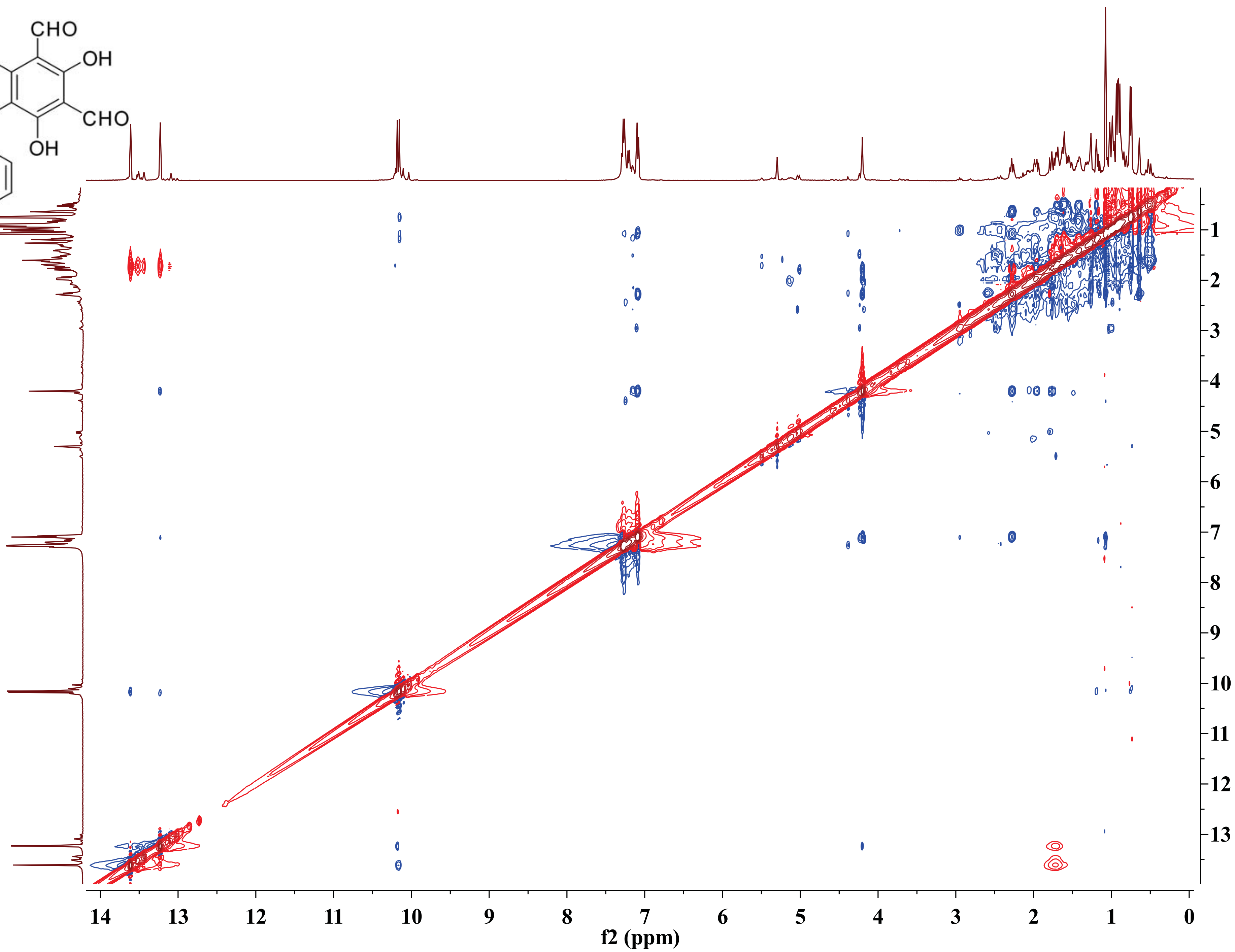

S5.13. ¹H NMR spectrum of compound **3**

In CDCl3

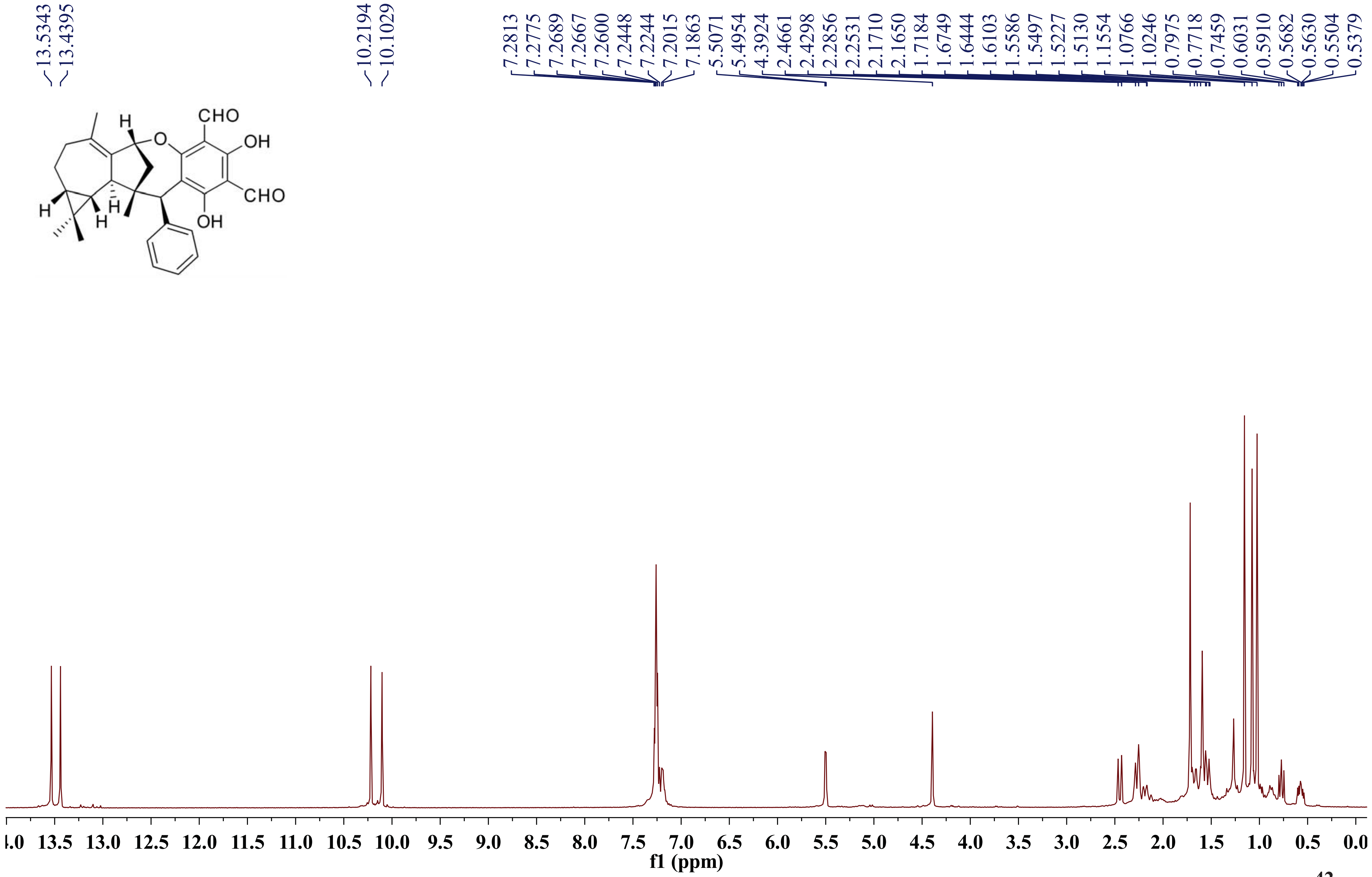

S5.14. DEPT spectra of compound **3**

In CDCl3

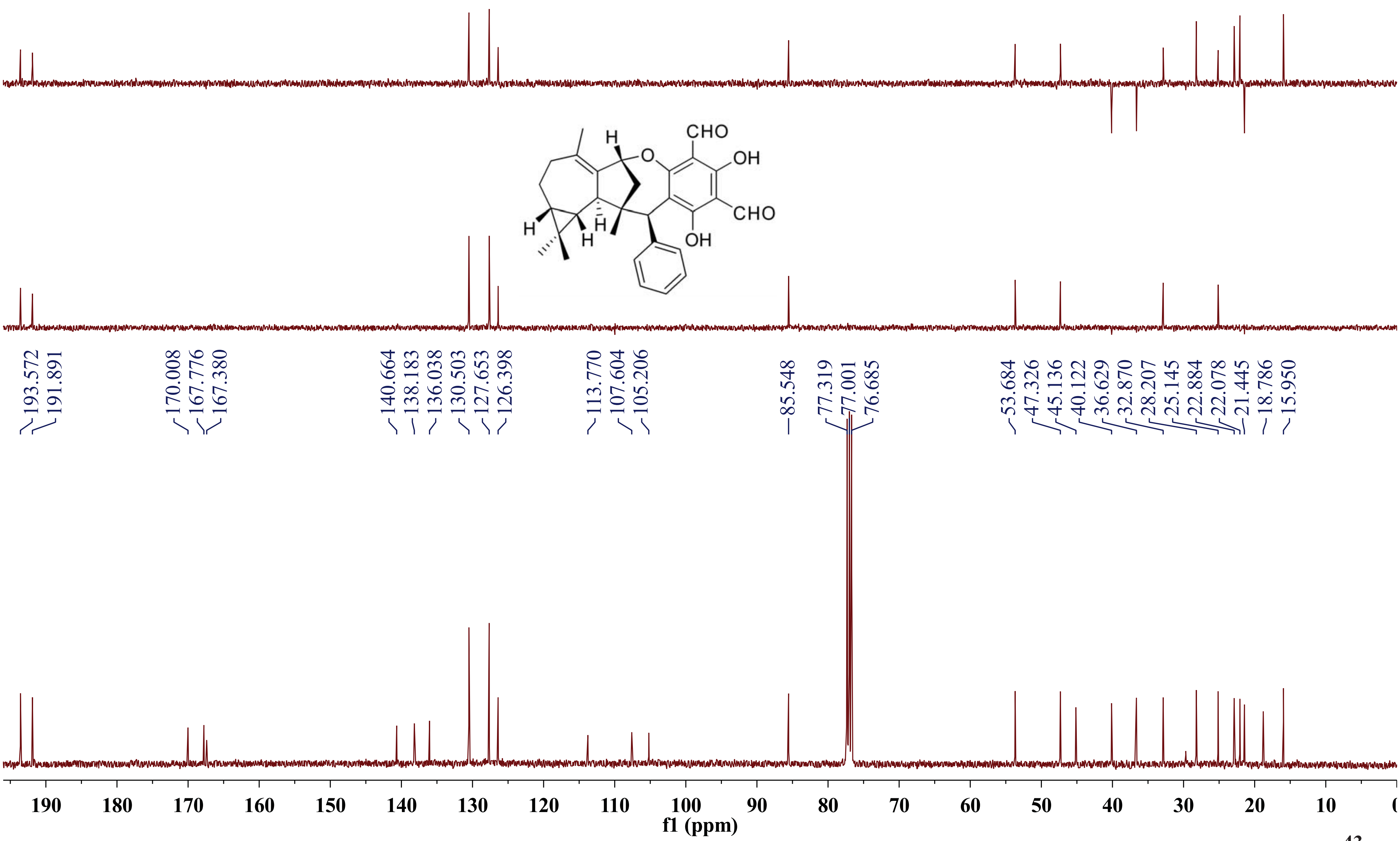

S5.15. HSQC spectrum of compound **3**

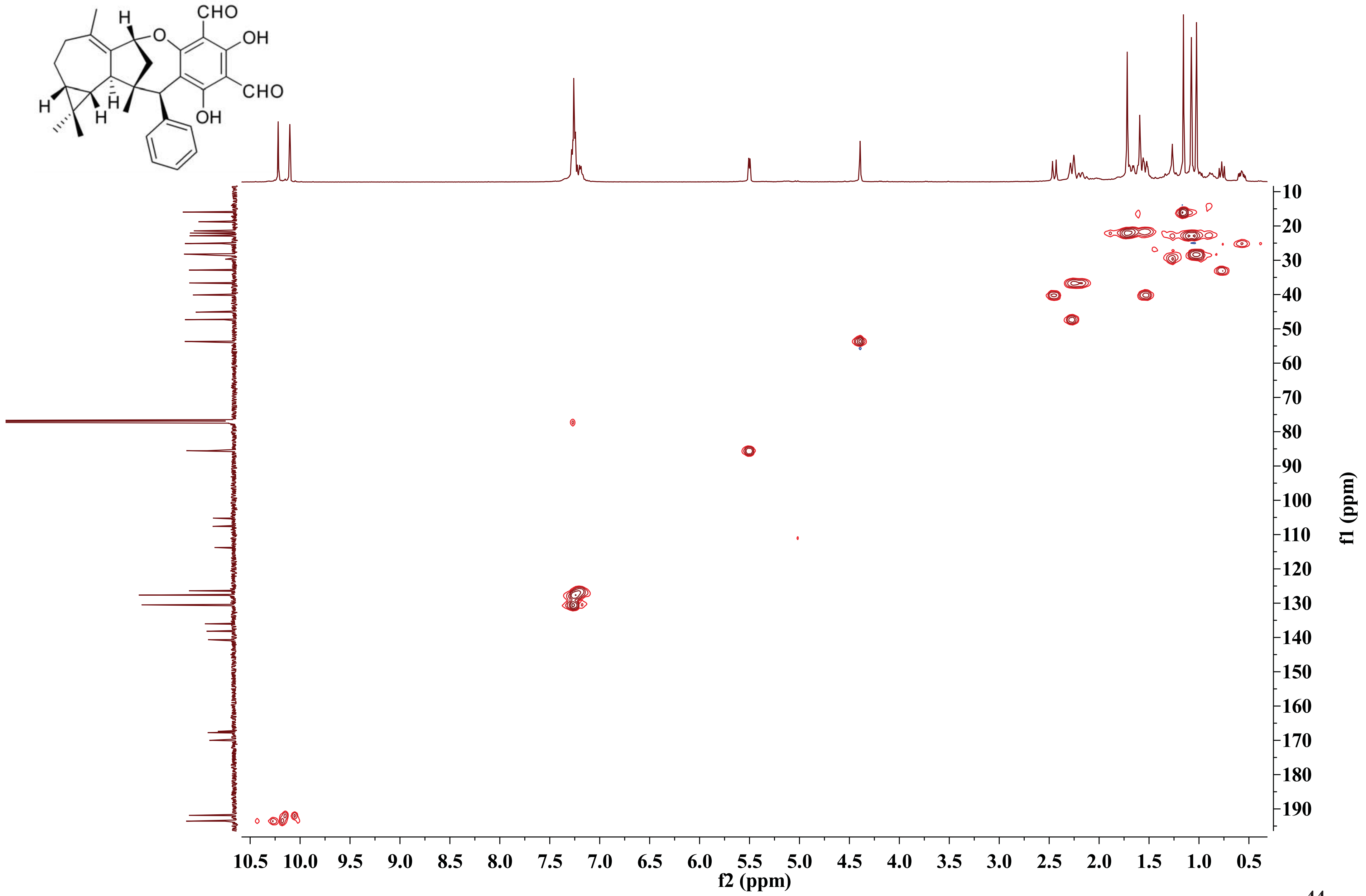

S5.16. ^{1}H-^{1}H COSY spectrum of compound **3**

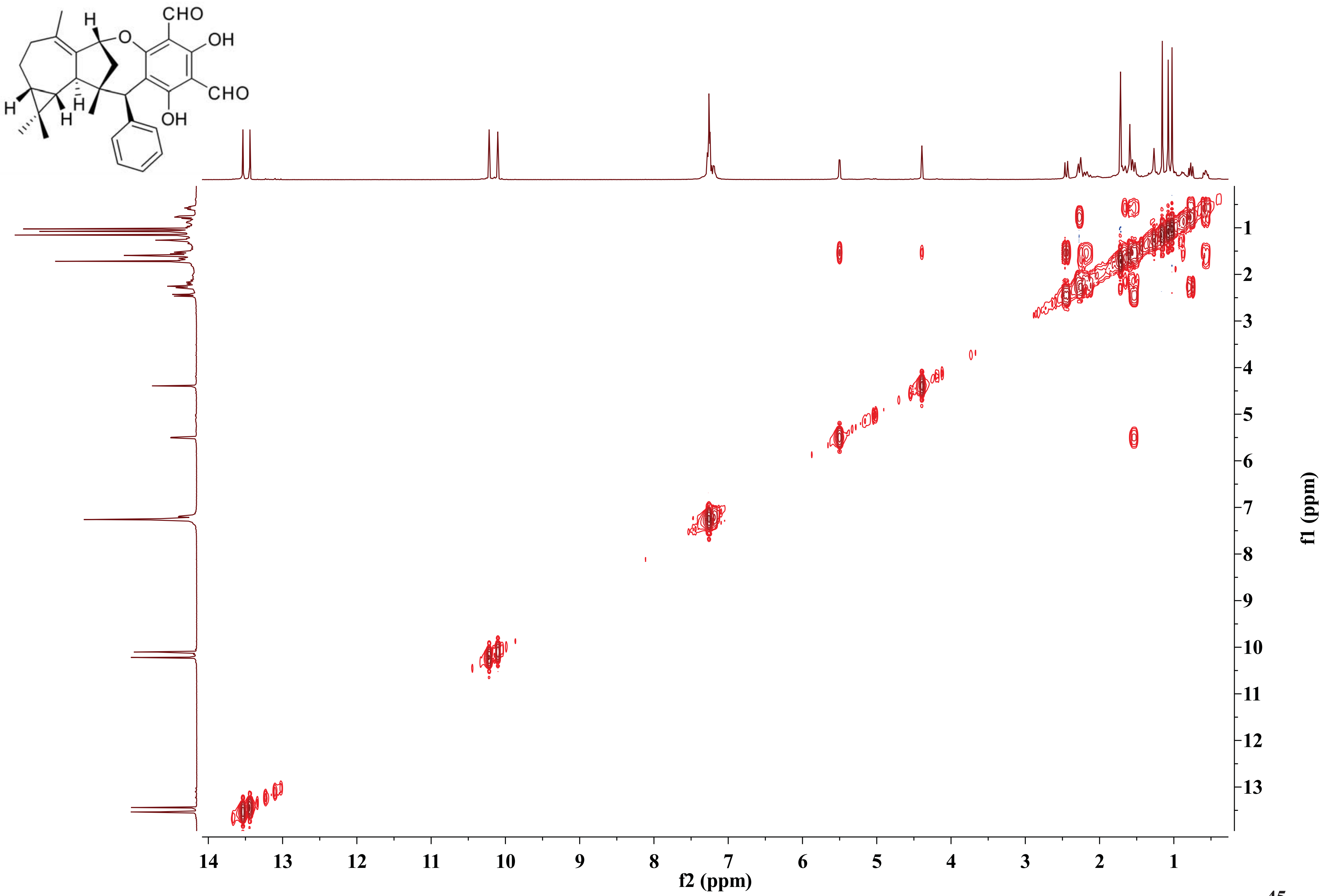

S5.17. HMBC spectrum of compound **3**

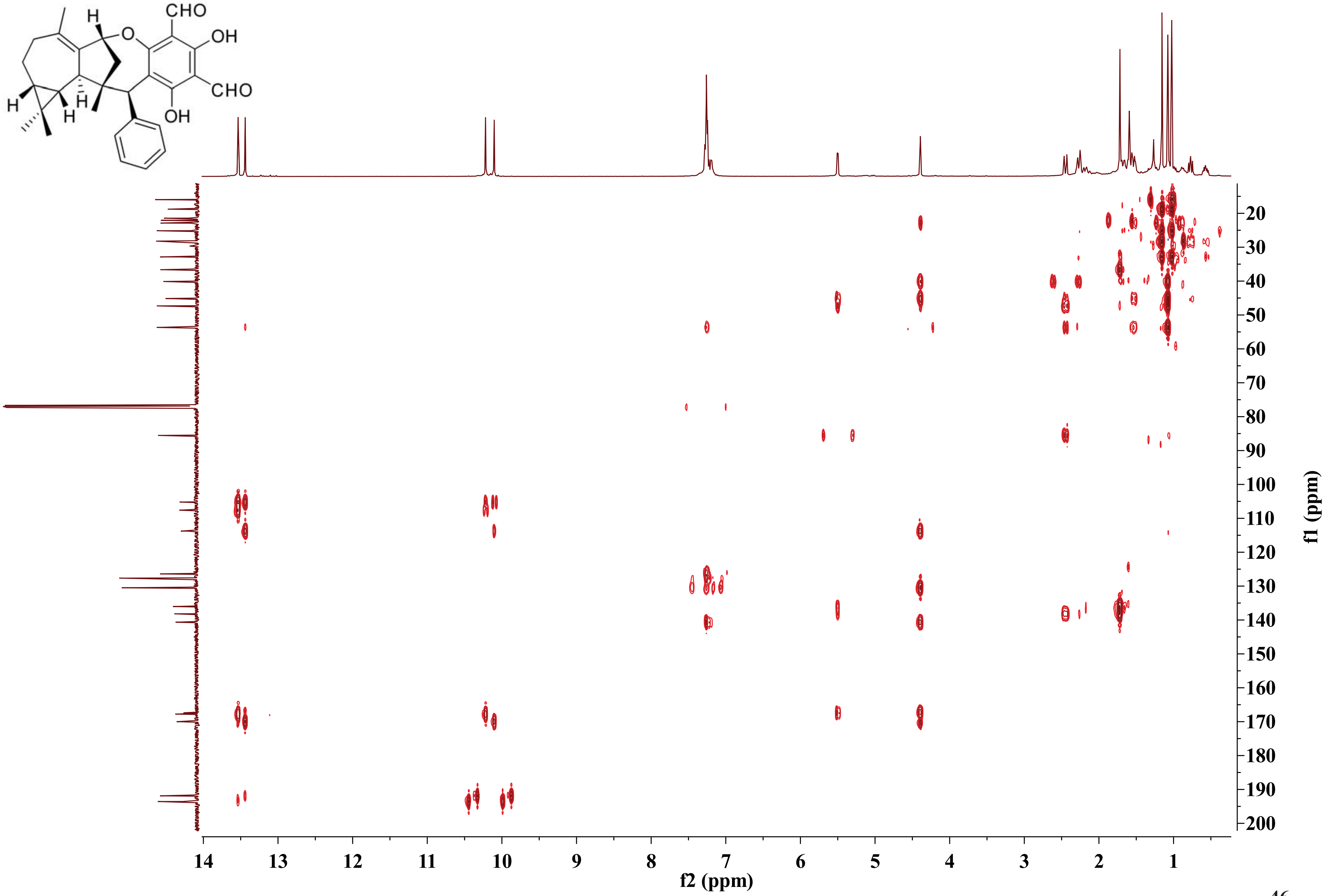

S5.18. NOESY spectrum of compound **3**

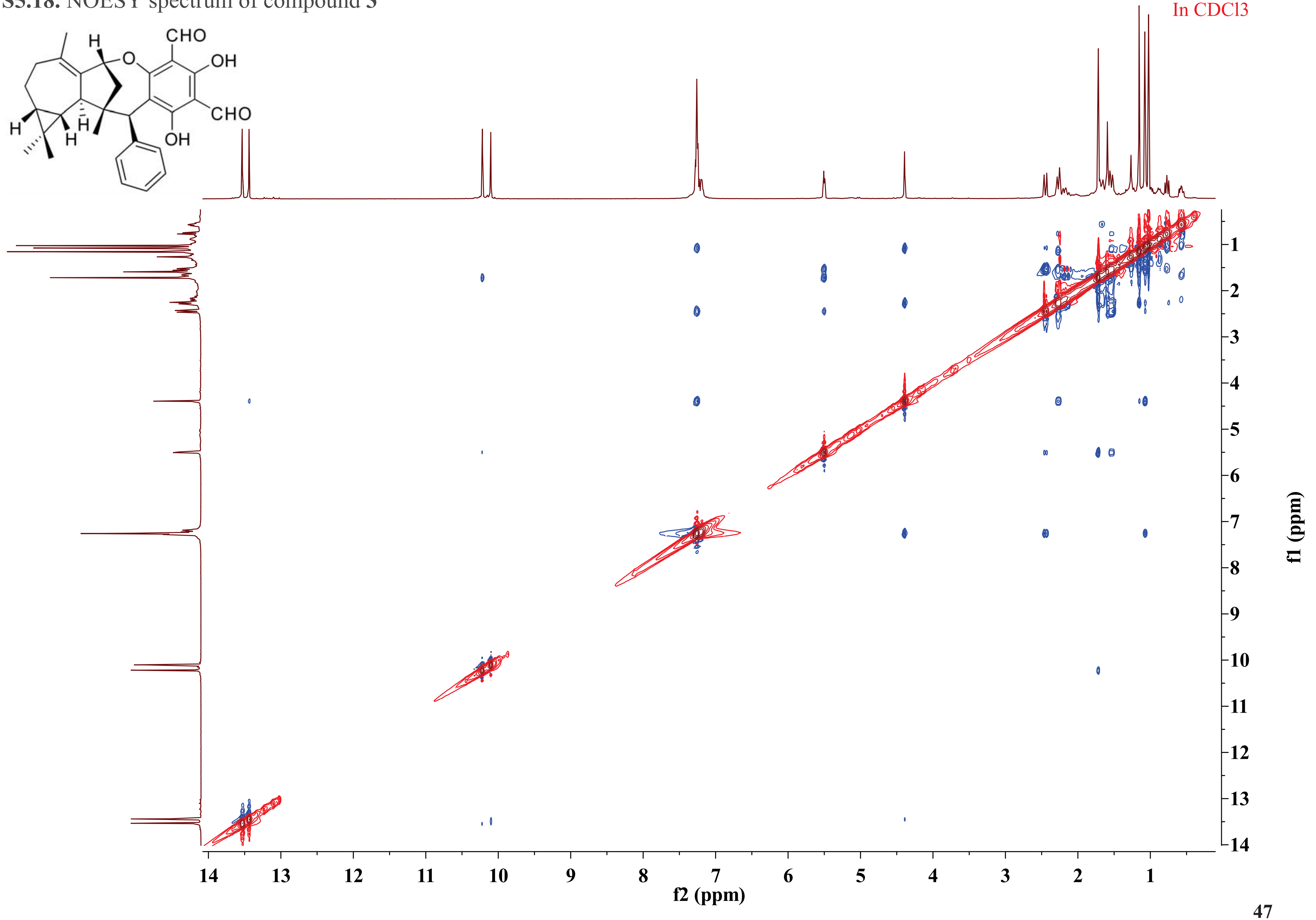

S5.19. ^{1}H NMR spectrum of compound **4**

In CDCl3

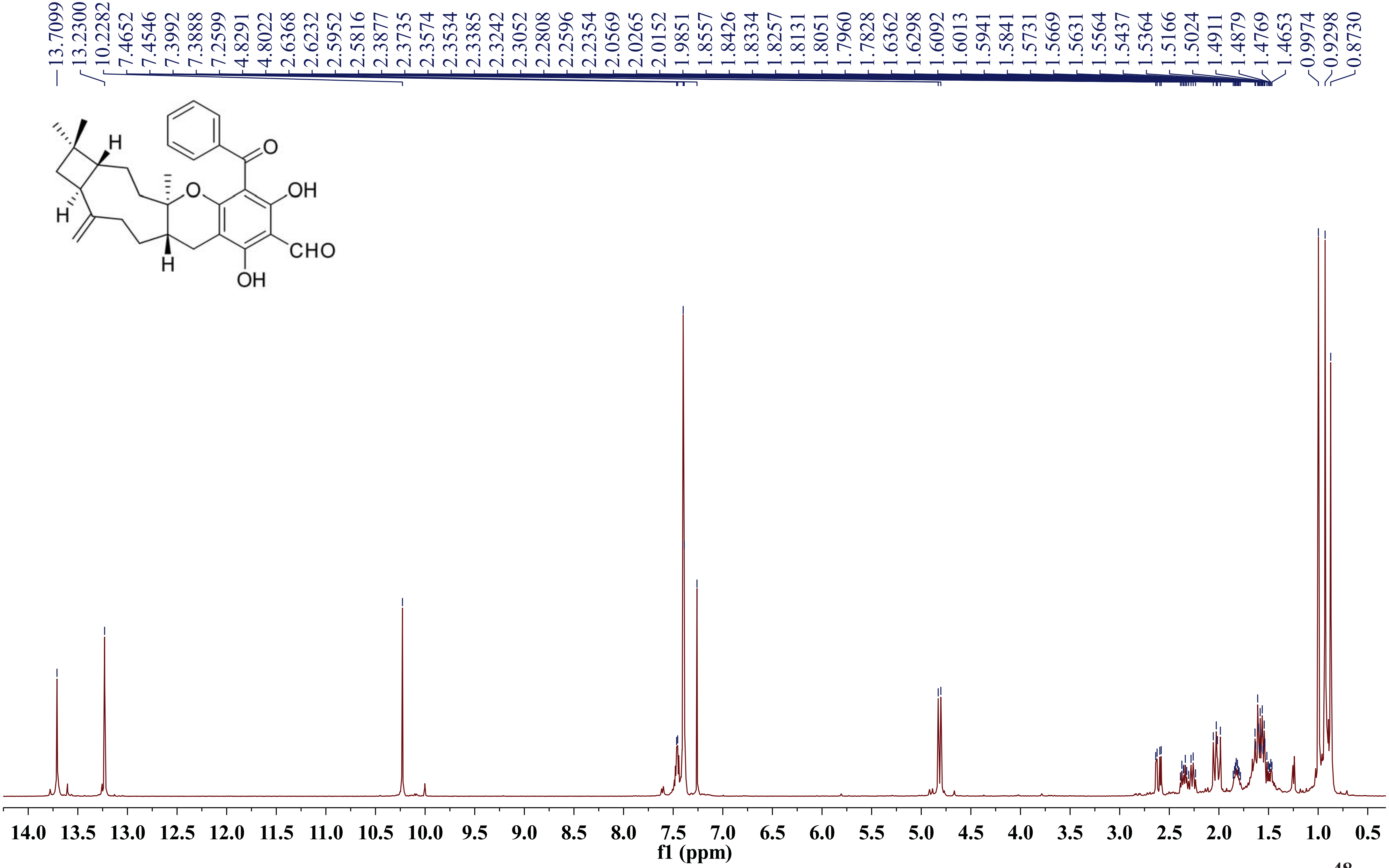

S5.20. DEPT spectra of compound **4**

In CDCl3

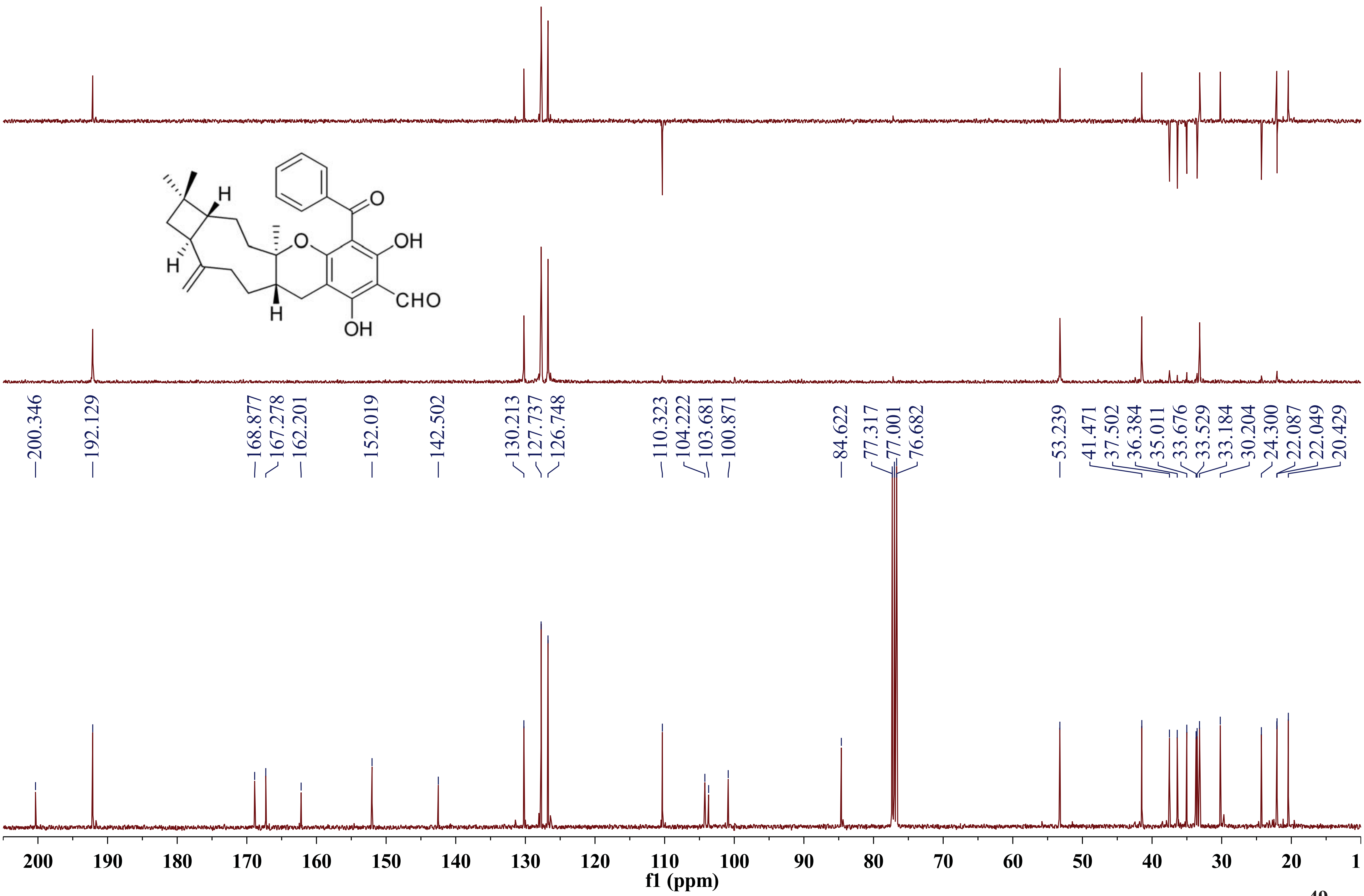

S5.21. HSQC spectrum of compound **4**

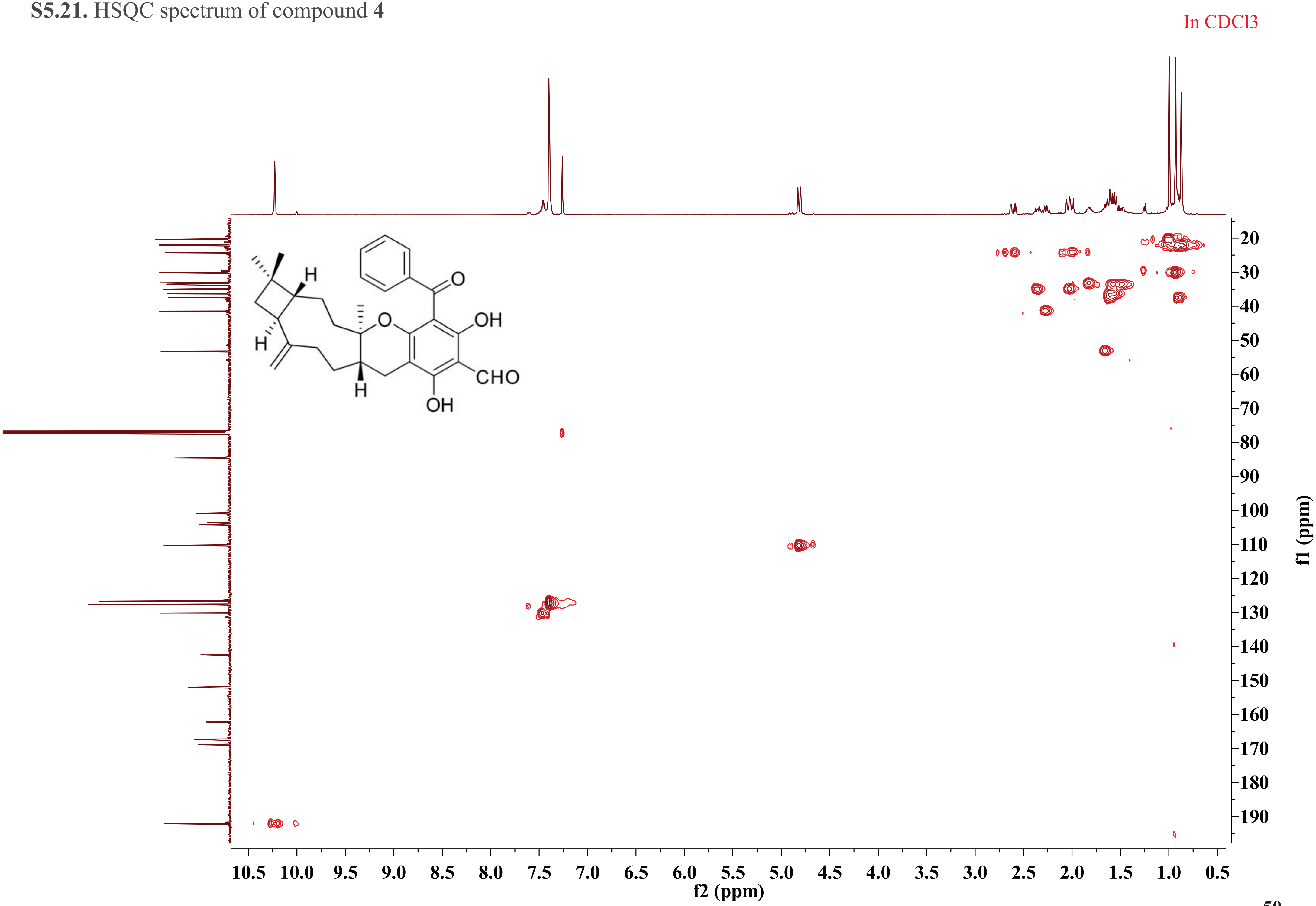

S5.22. ^{1}H-^{1}H COSY spectrum of compound **4**

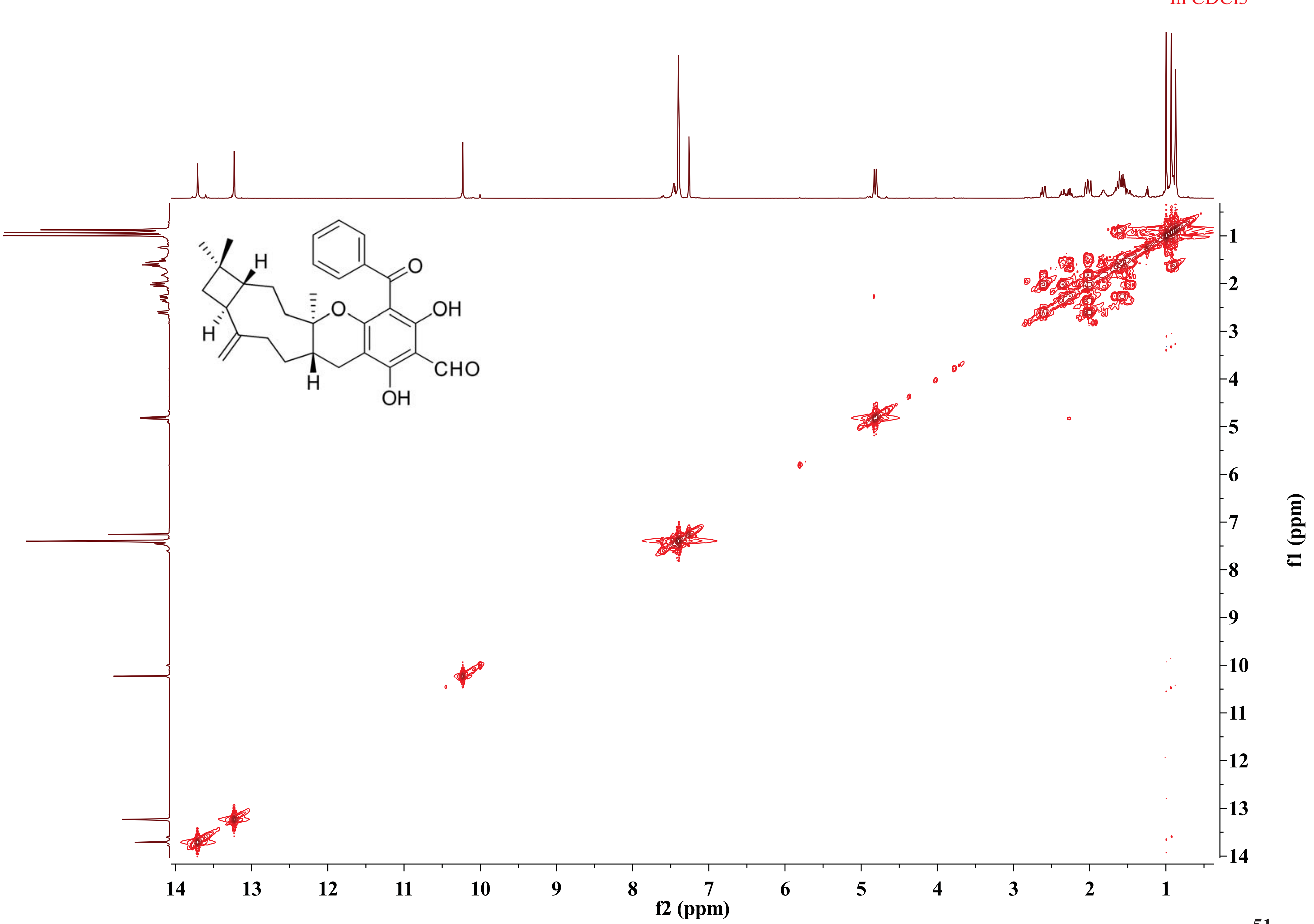

S5.23. HMBC spectrum of compound **4**

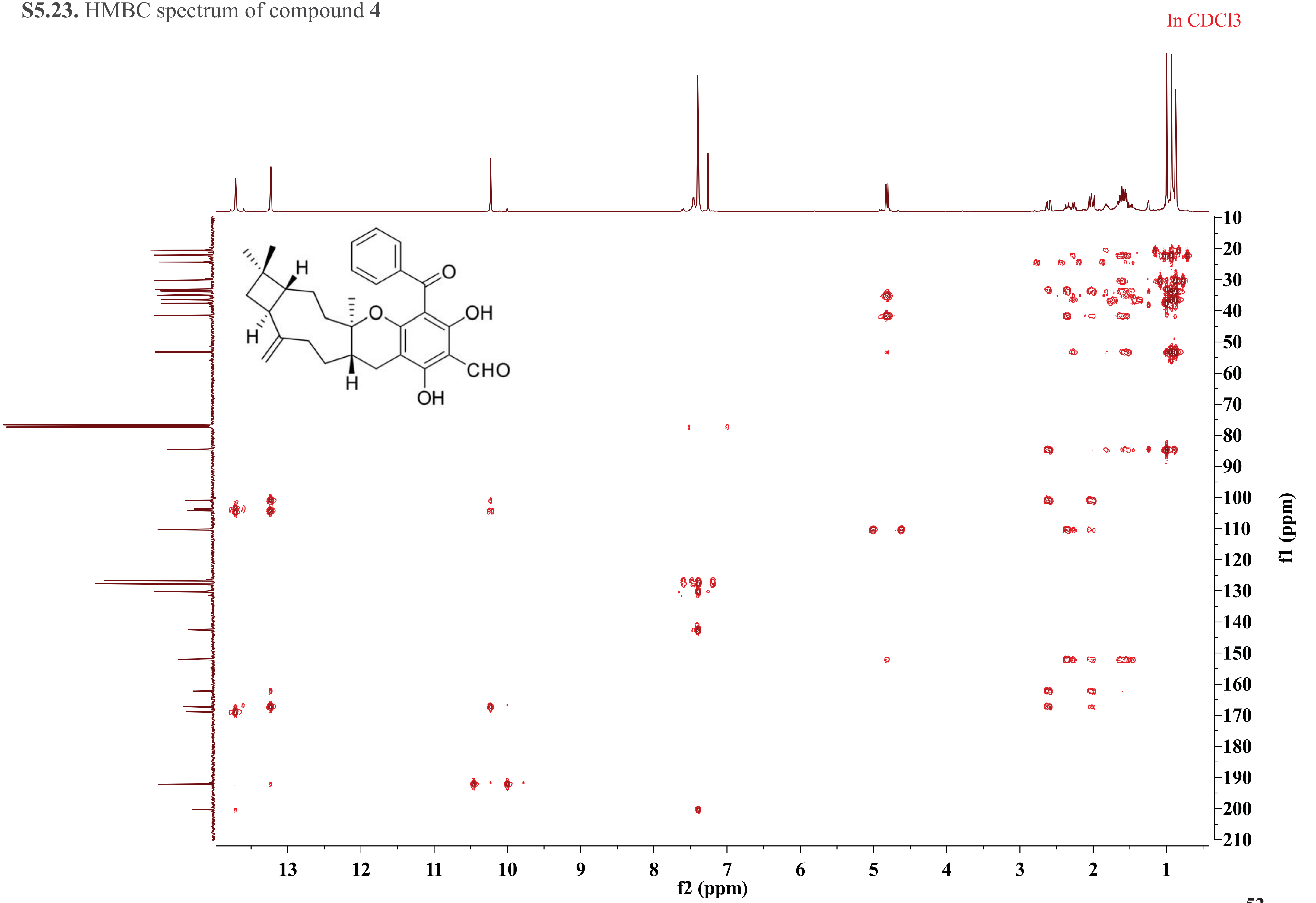

S5.24. NOESY spectrum of compound **4**

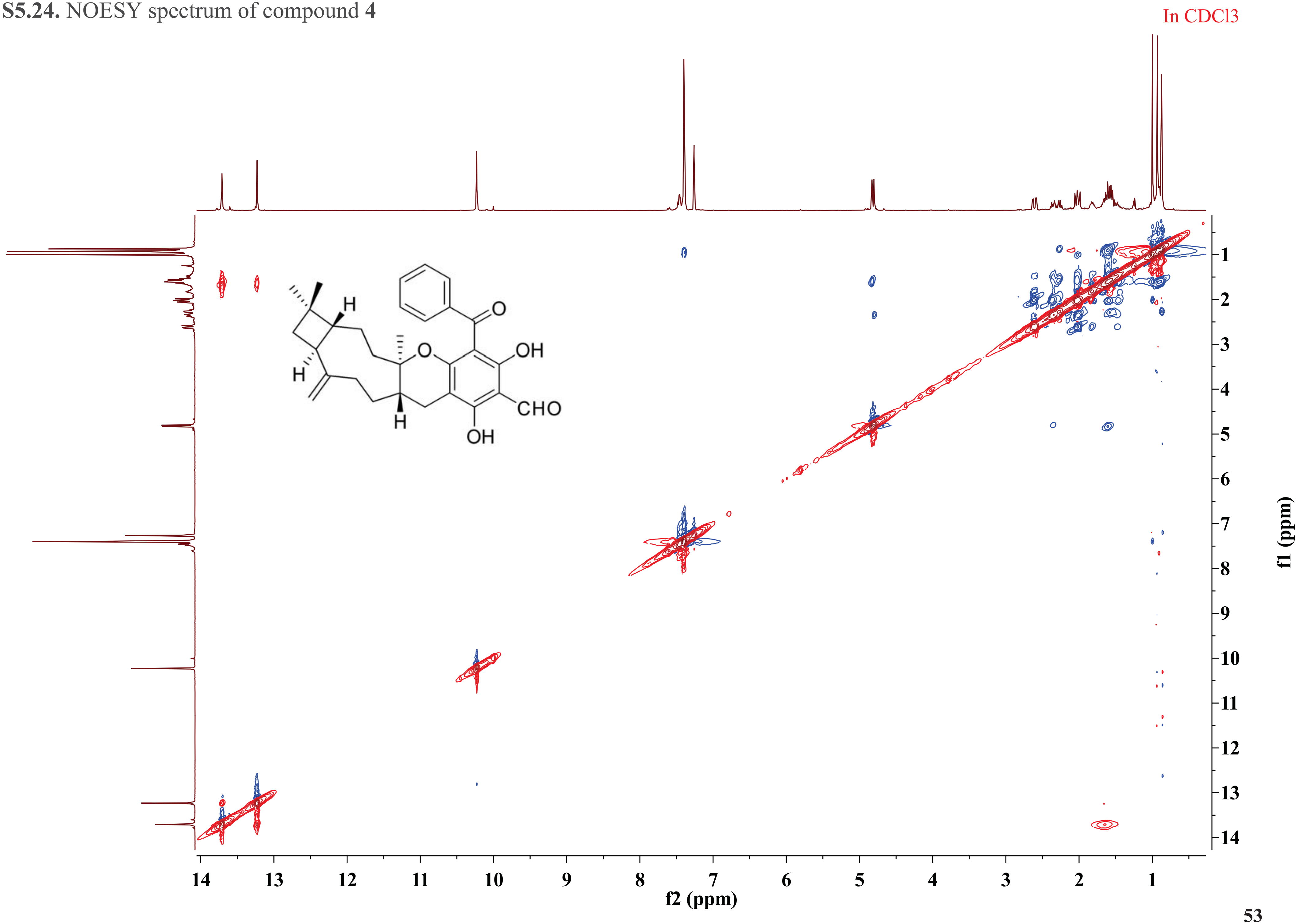

S5.25. ^{1}H NMR spectrum of compound **5**

In CDCl3

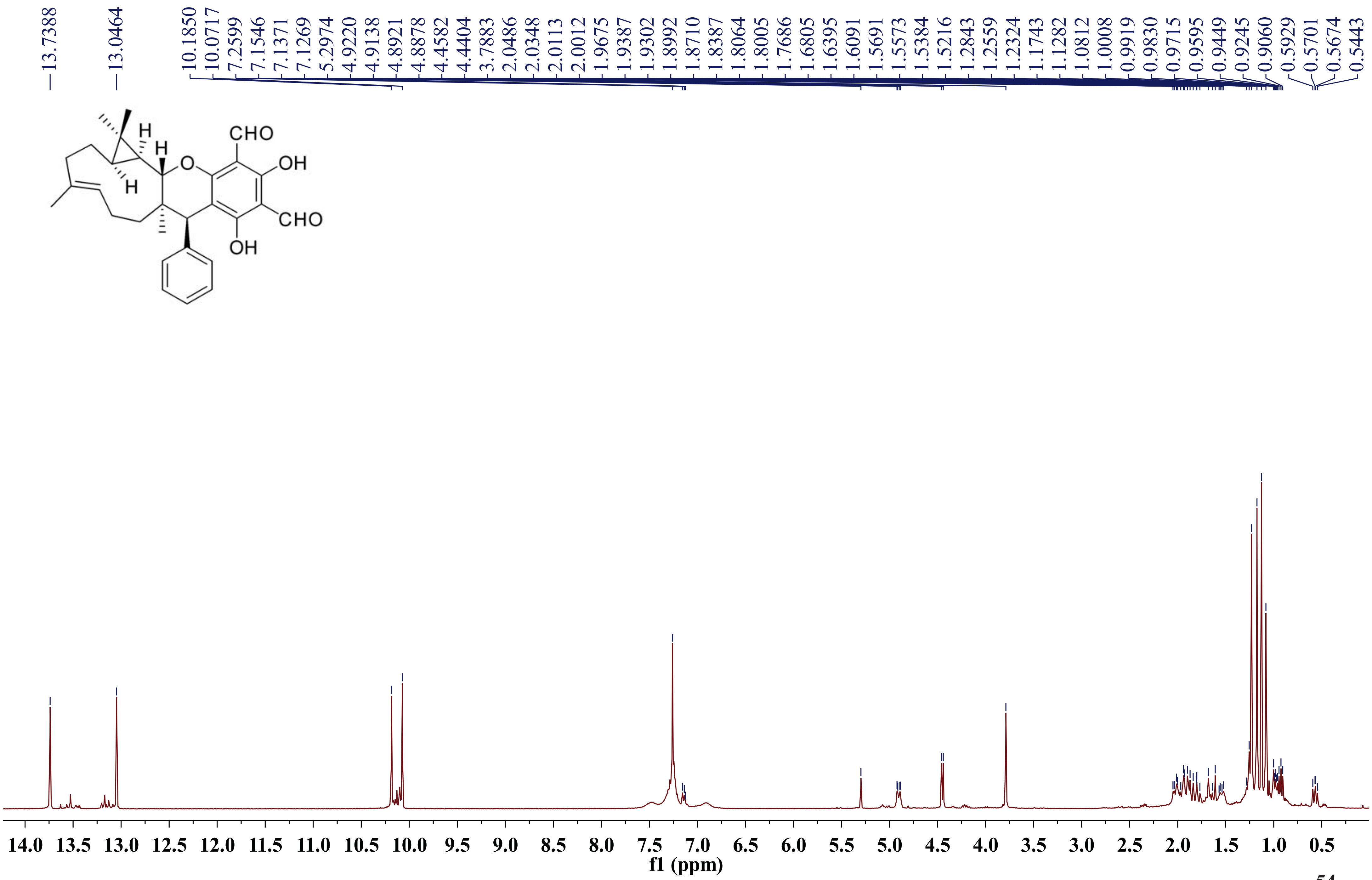

S5.26. DEPT spectra of compound **5**

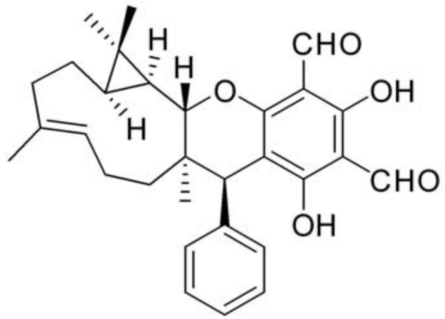

In CDCl3

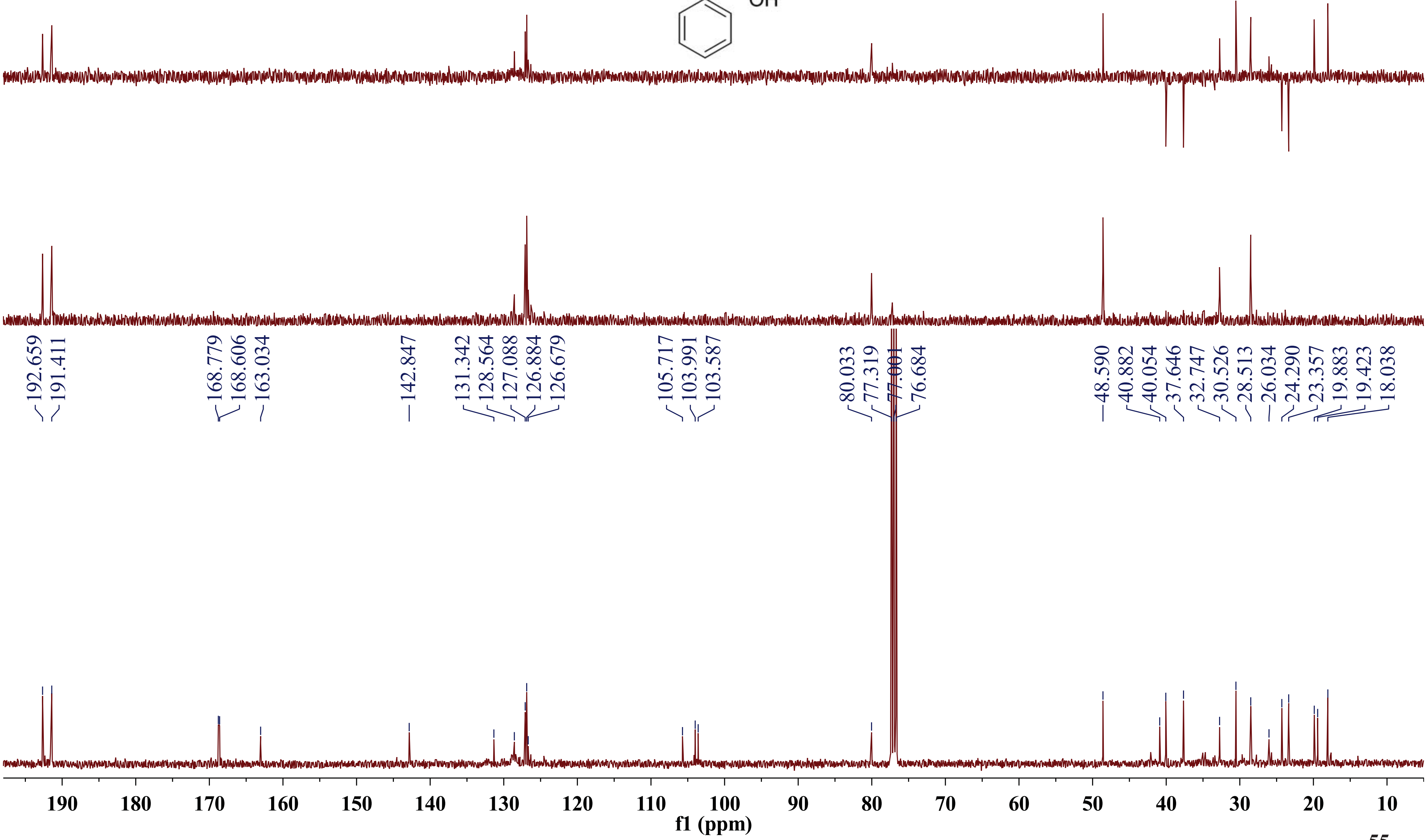

S5.27. HSQC spectrum of compound **5**

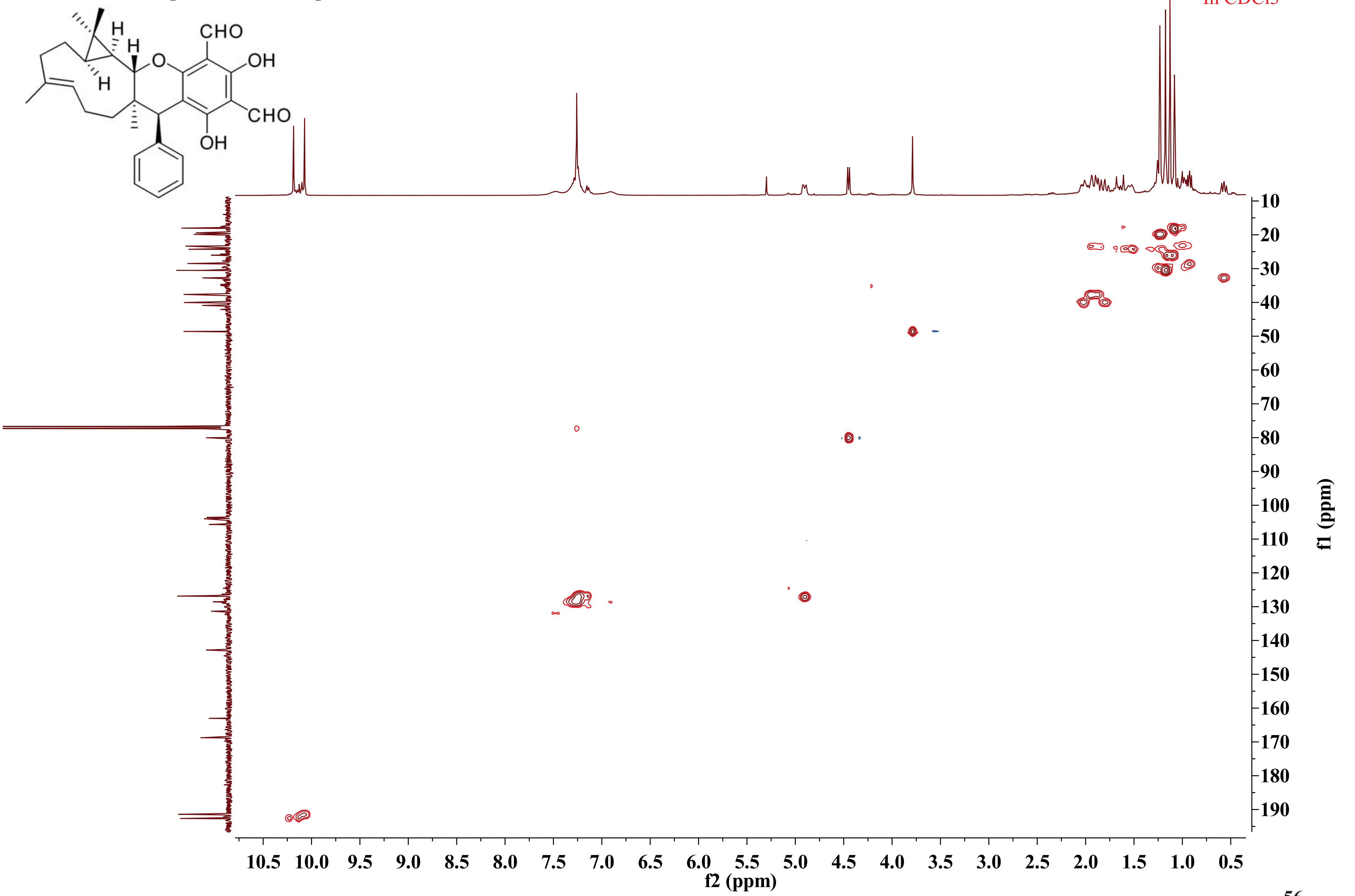

S5.28. ^1H-^1H COSY spectrum of compound **5**

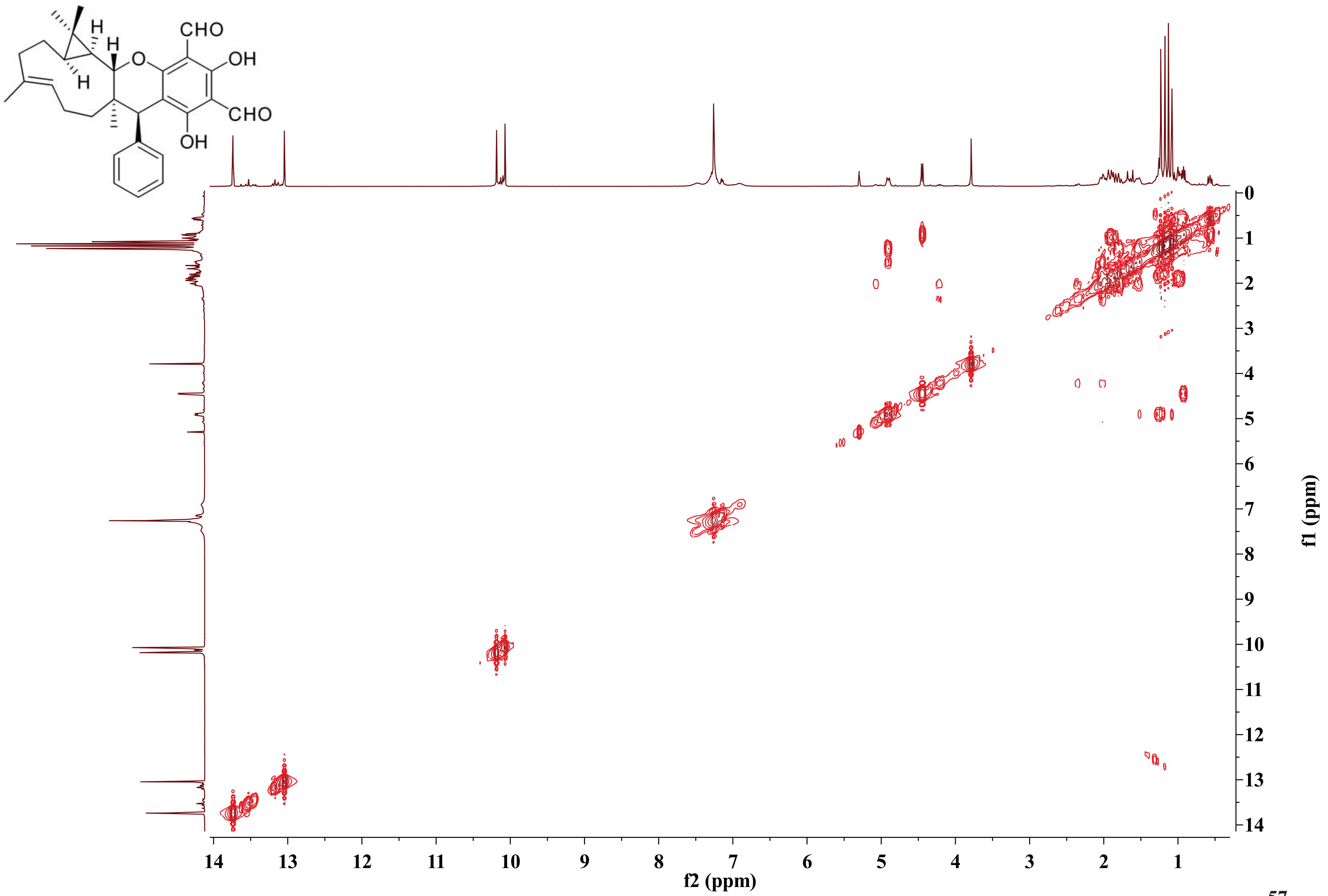

S5.29. HMBC spectrum of compound **5**

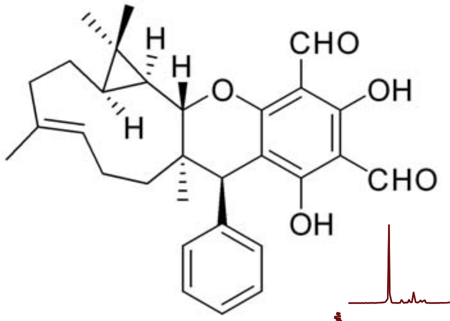

In CDCl3

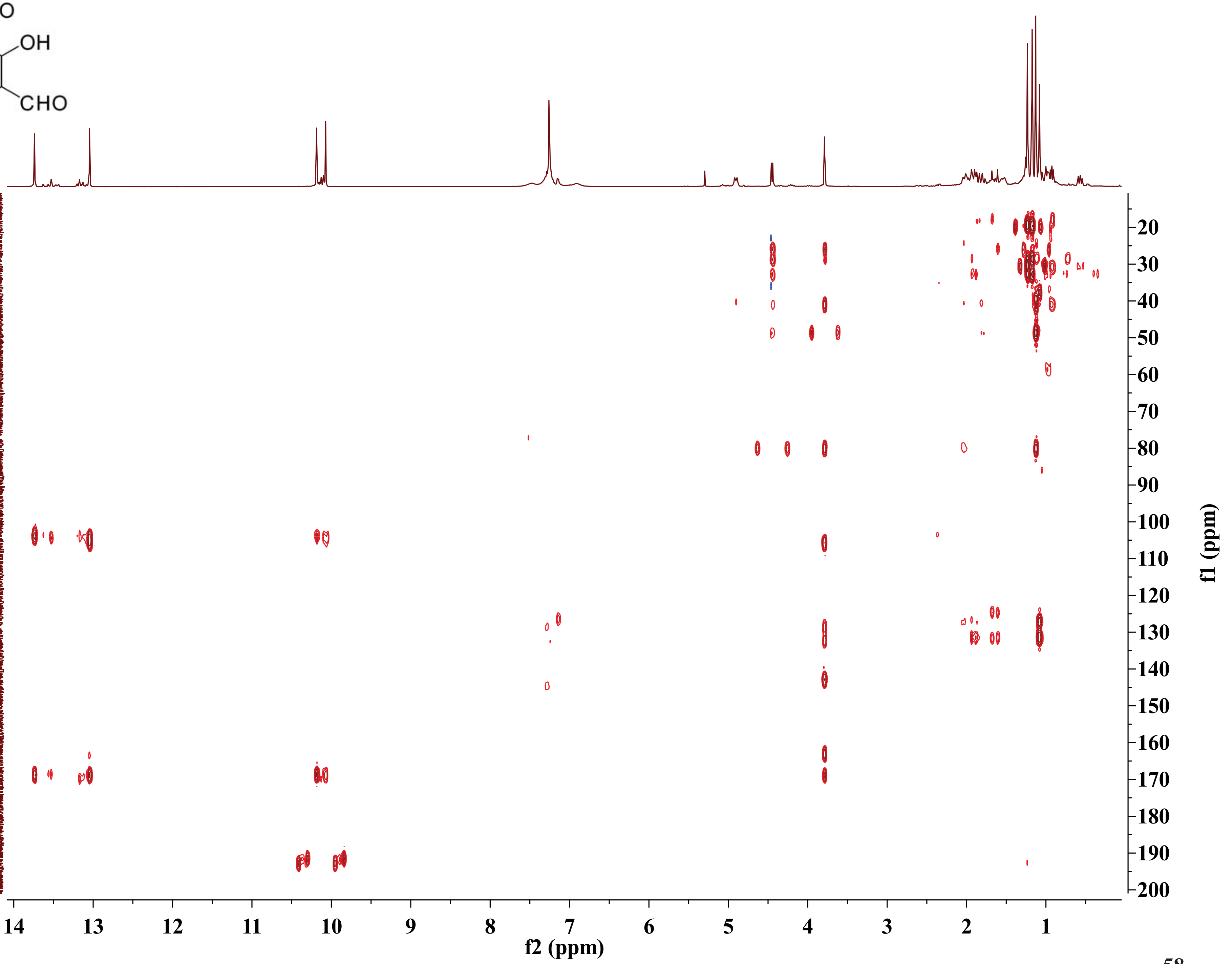

S5.30. NOESY spectrum of compound **5**

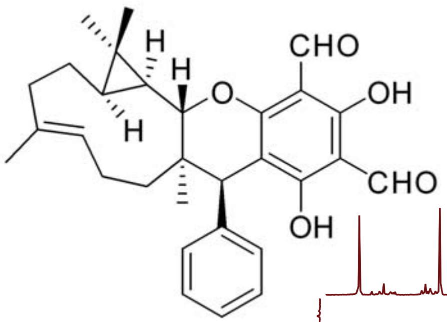

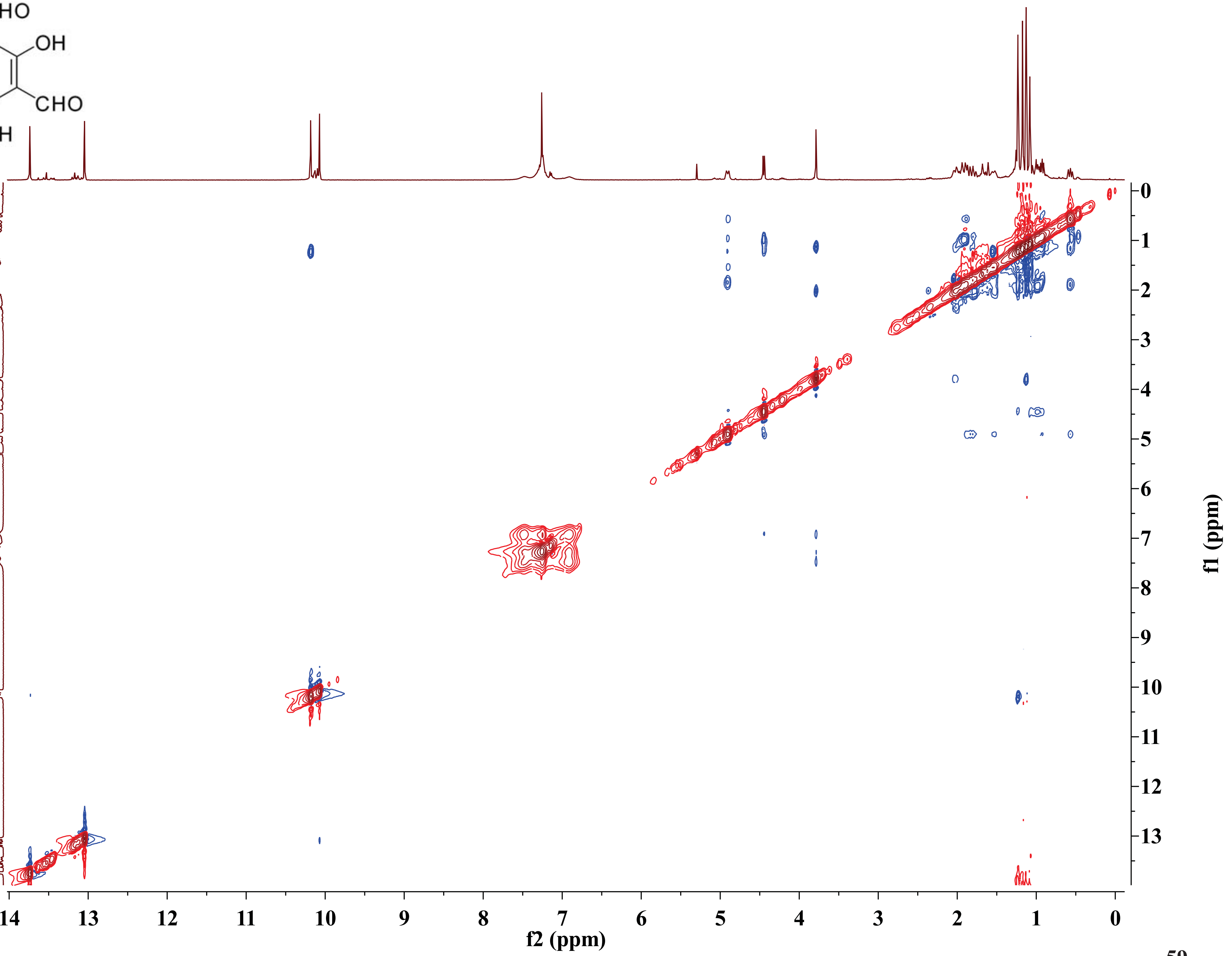

S5.31. ¹H NMR spectrum of compound **6**

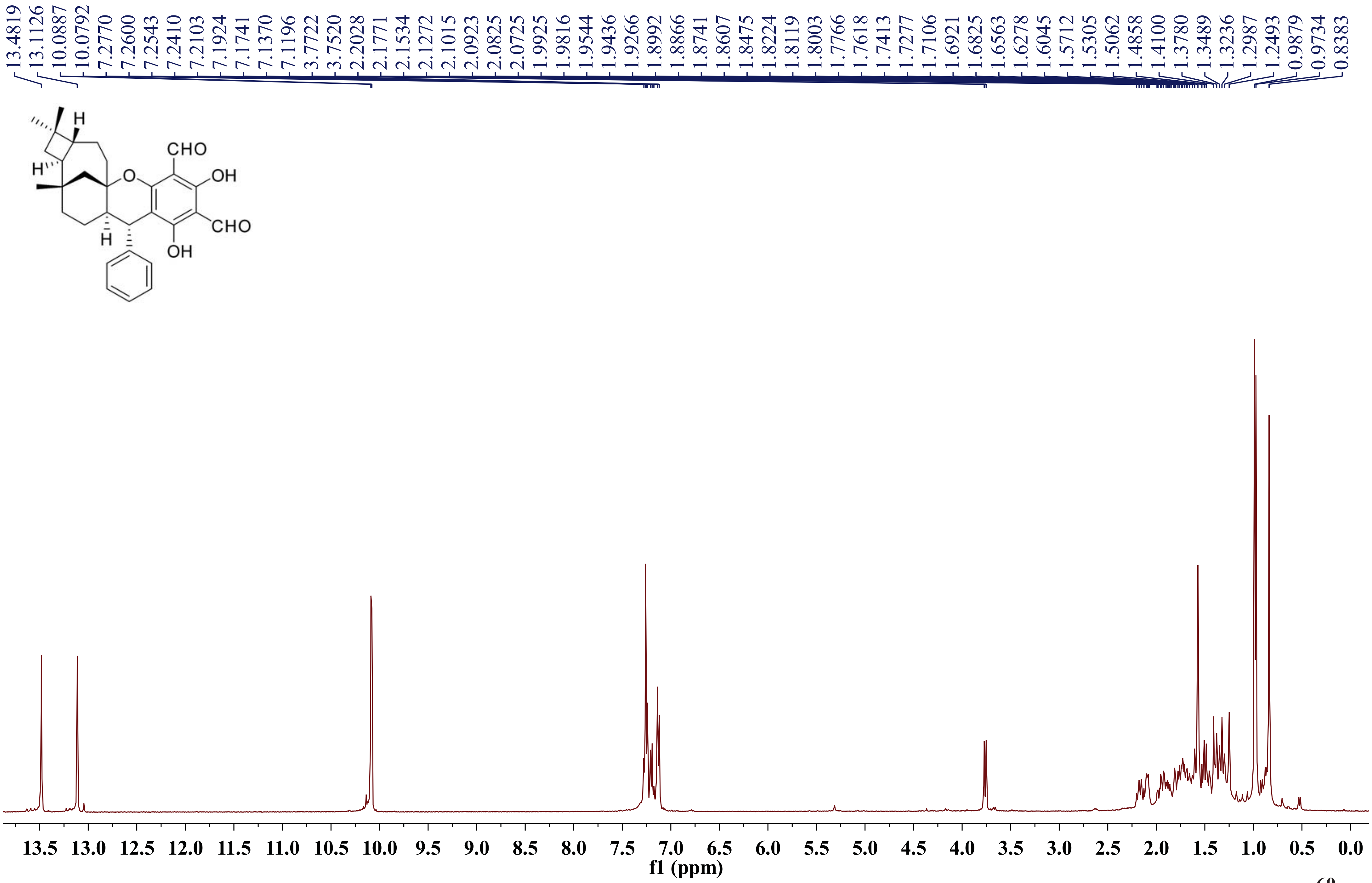

S5.32. DEPT spectra of compound **6**

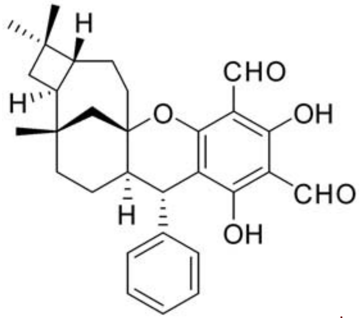

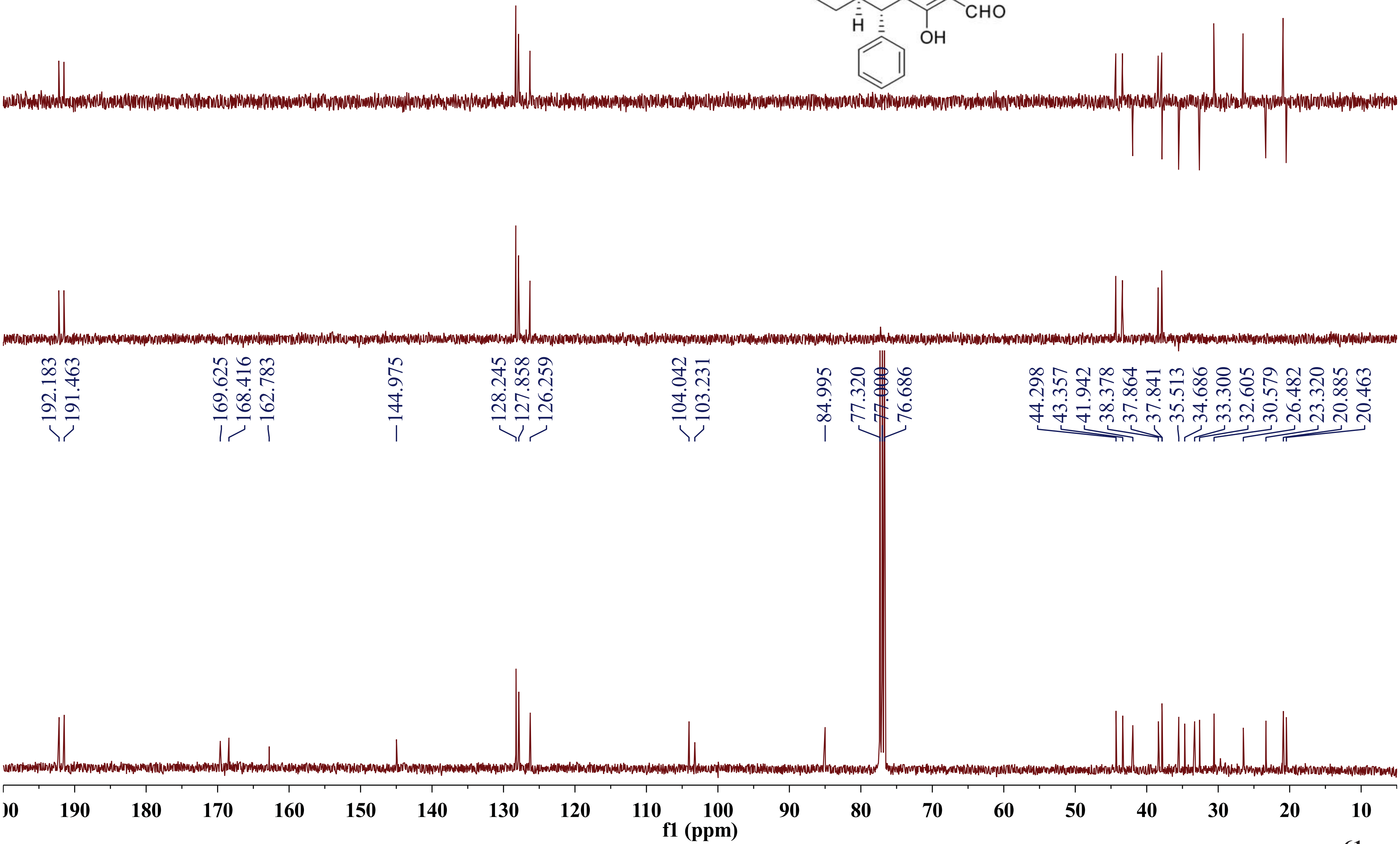

S5.33. HSQC spectrum of compound **6**

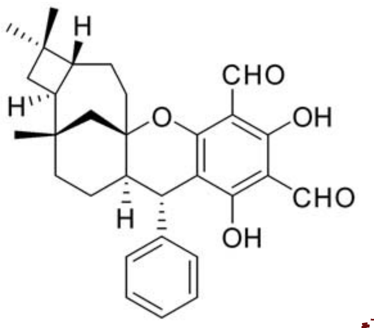

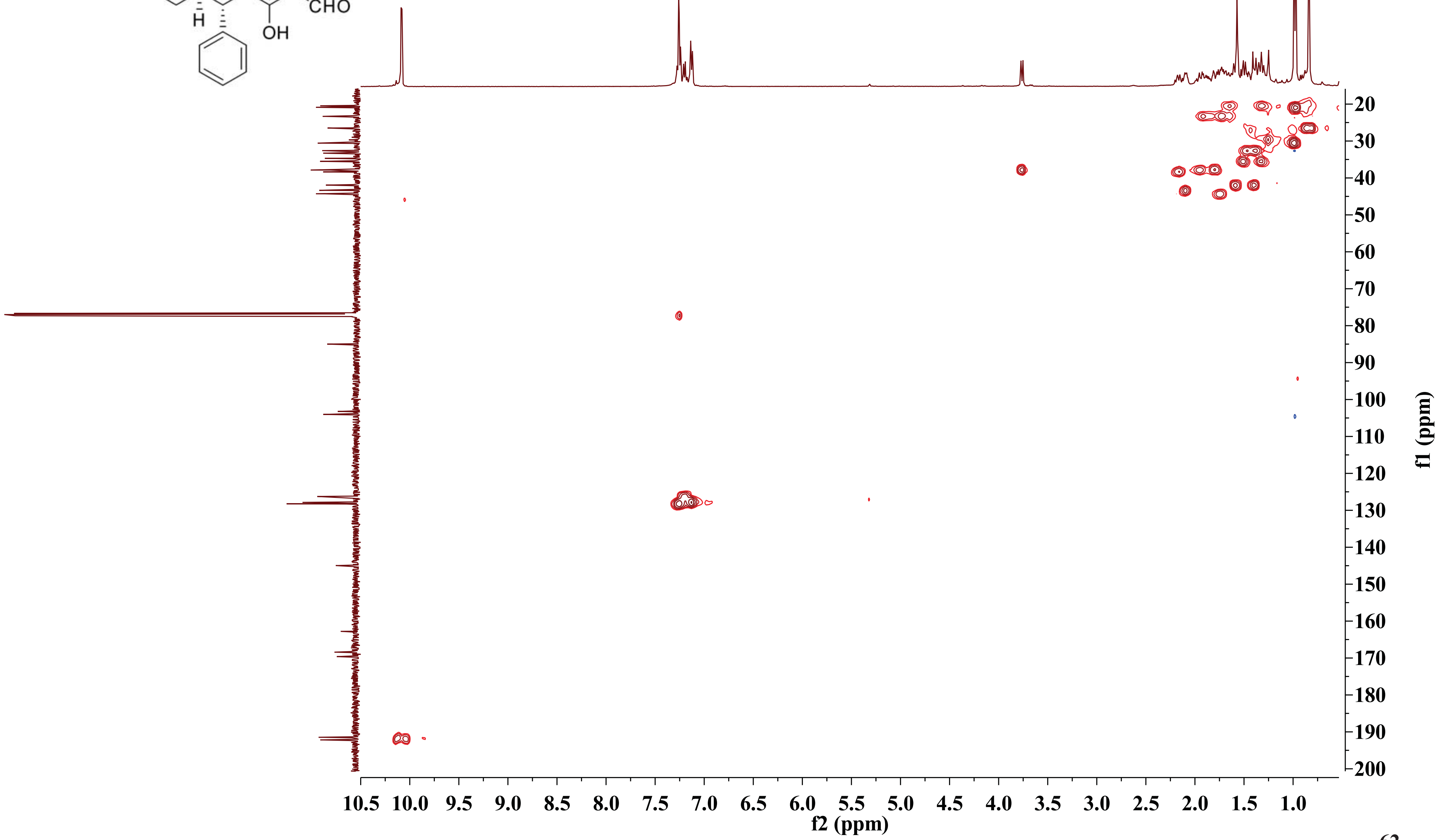

S5.34. ^{1}H-^{1}H COSY spectrum of compound **6**

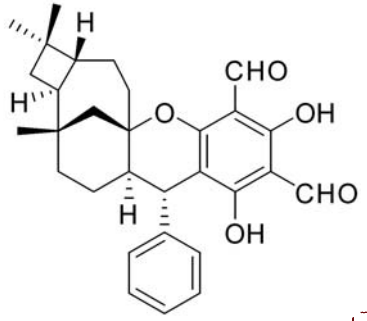

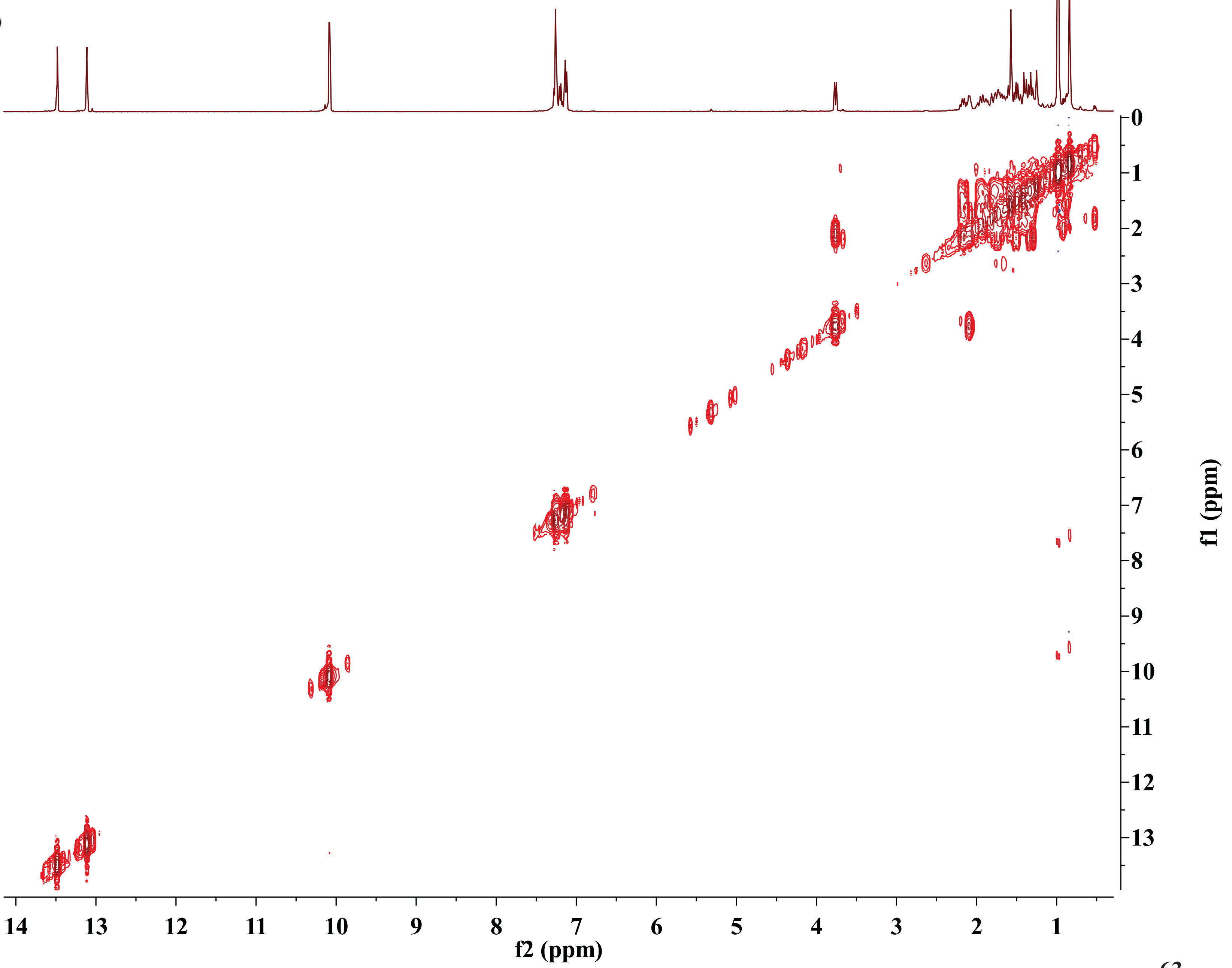

S5.35. HMBC spectrum of compound **6**

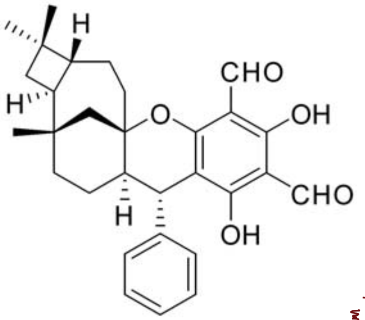

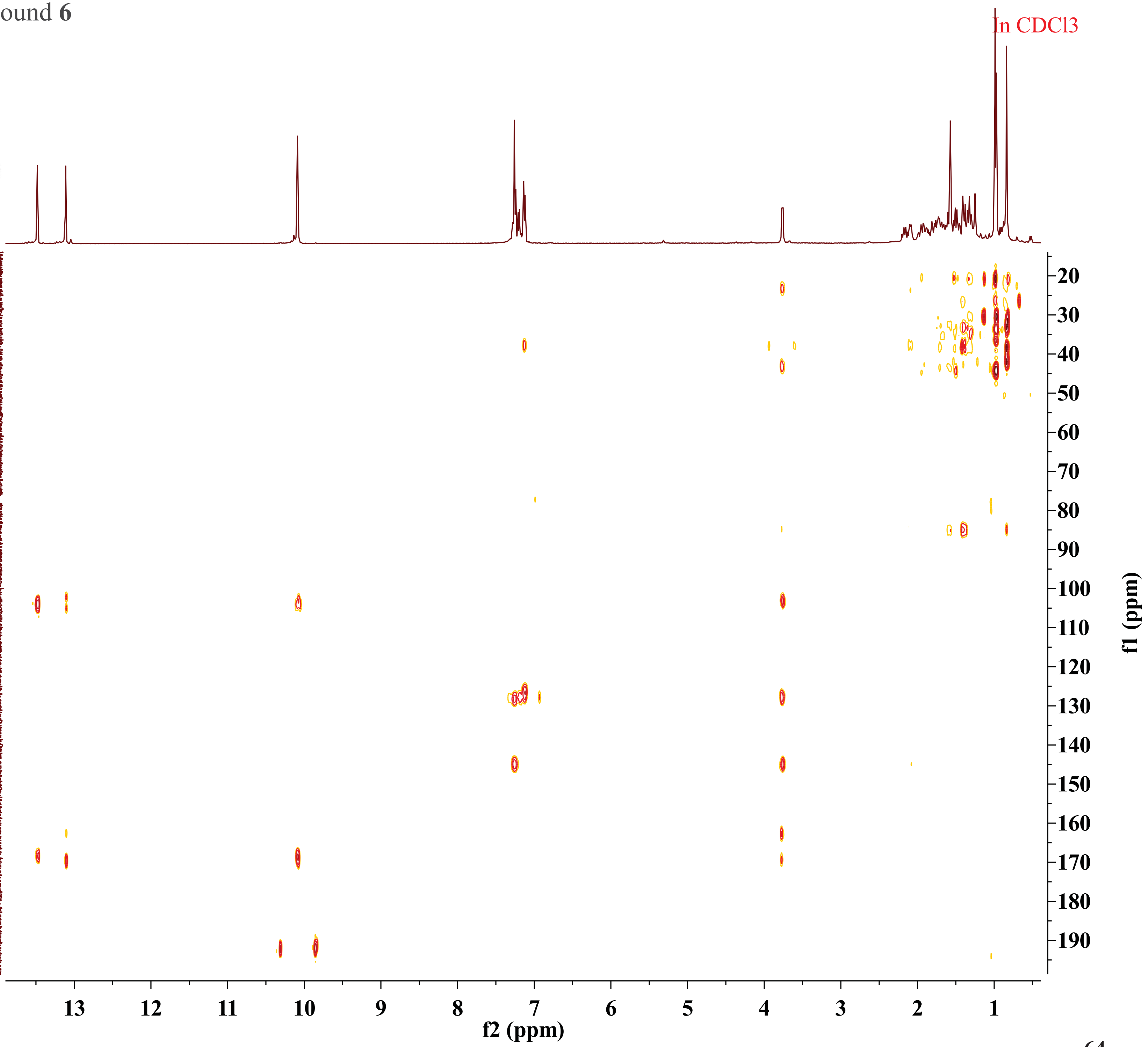

S5.36. NOESY spectrum of compound **6**

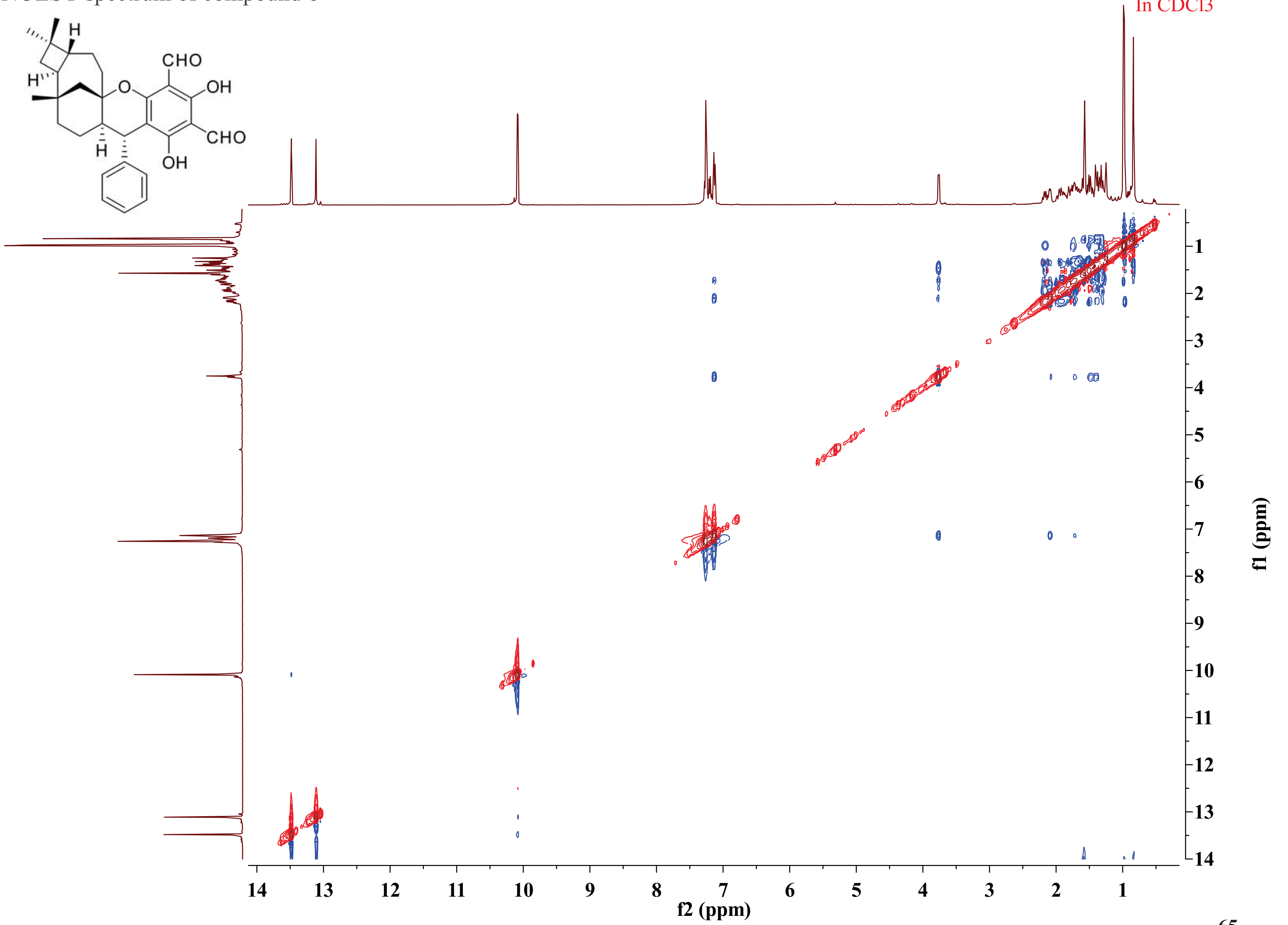

S5.37. ^{1}H NMR spectrum of compound **7**

In CDCl3

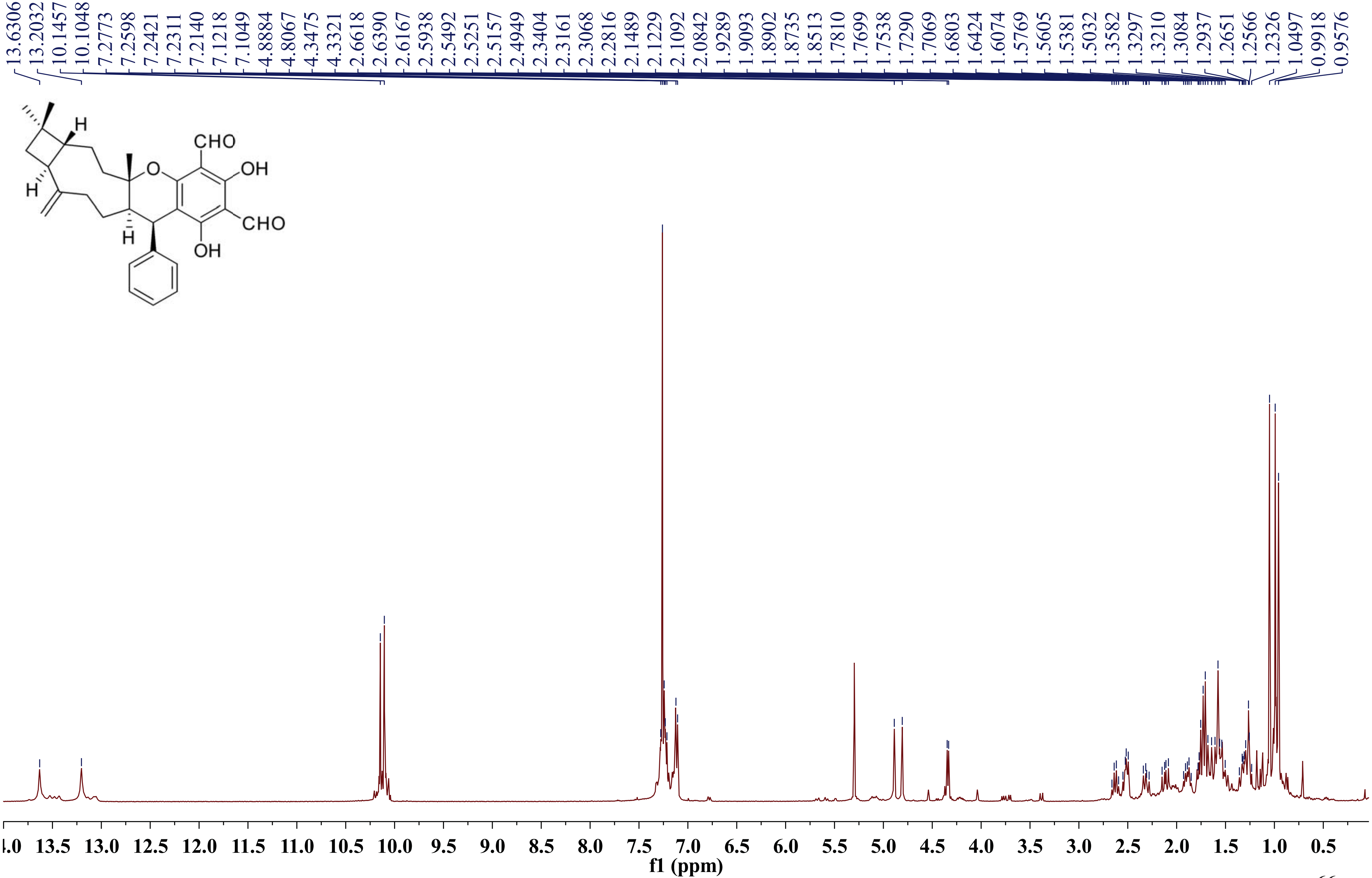

S5.38. DEPT spectra of compound **7**

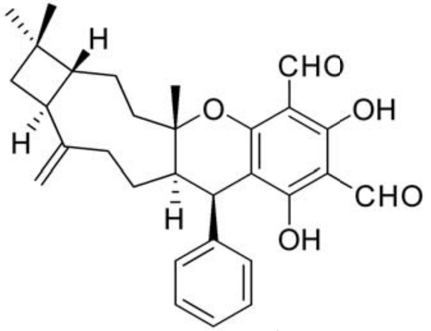

In CDCl3

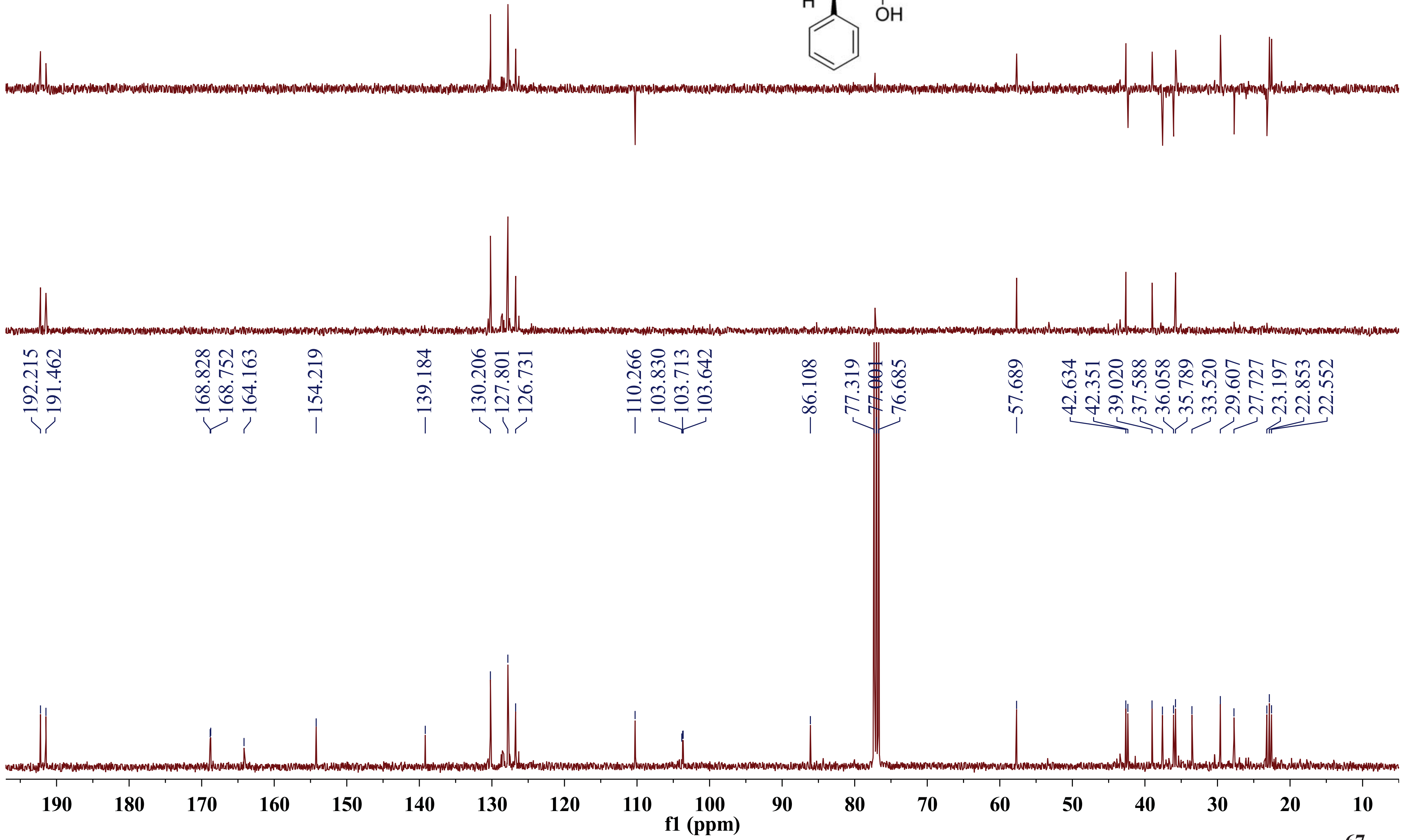

S5.39. HSQC spectrum of compound **7**

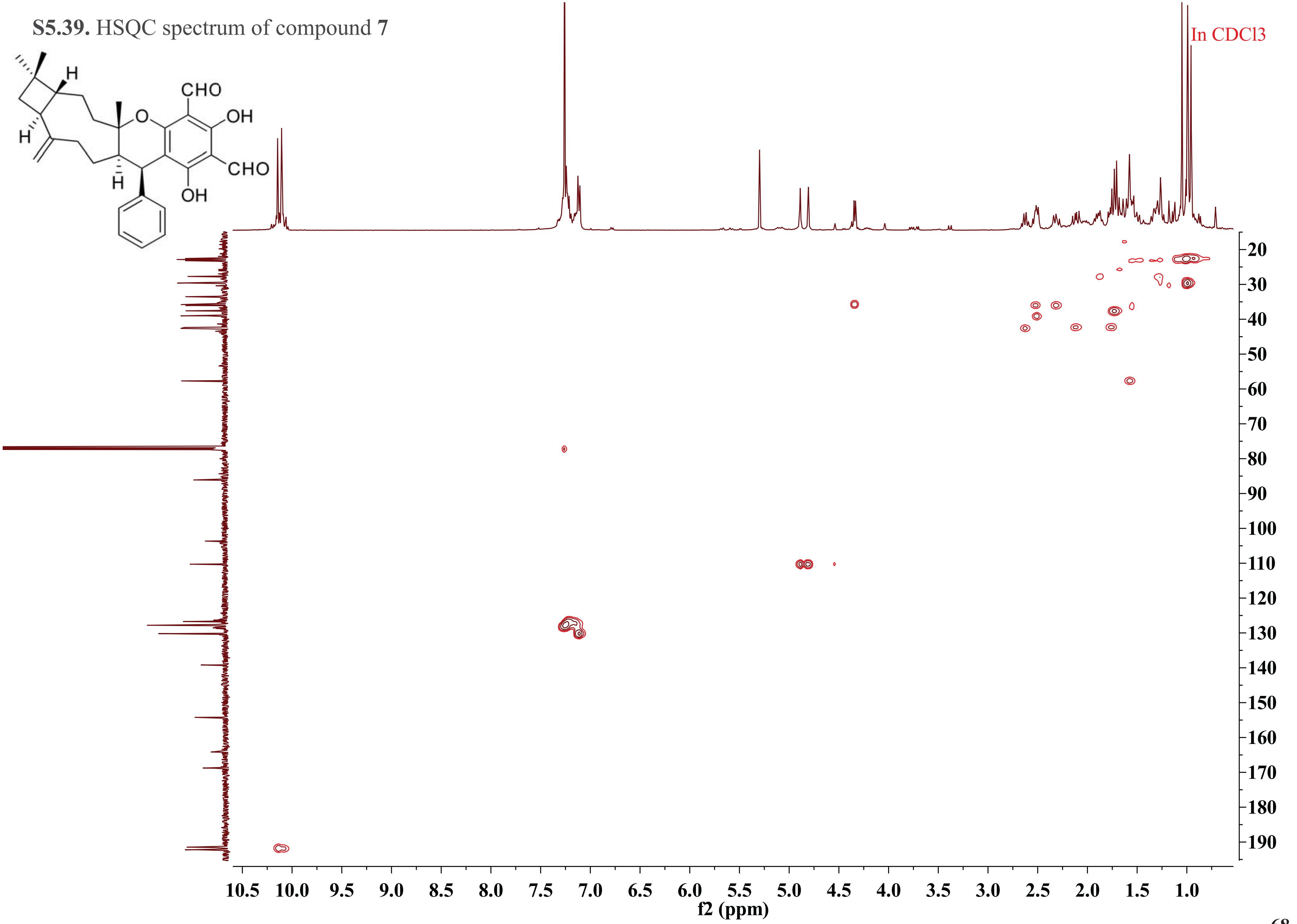

S5.40 ^{1}H-^{1}H COSY spectrum of compound **7**

In CDCl3

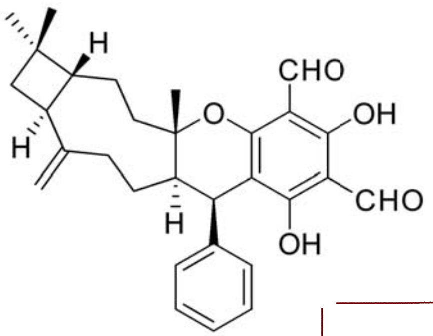

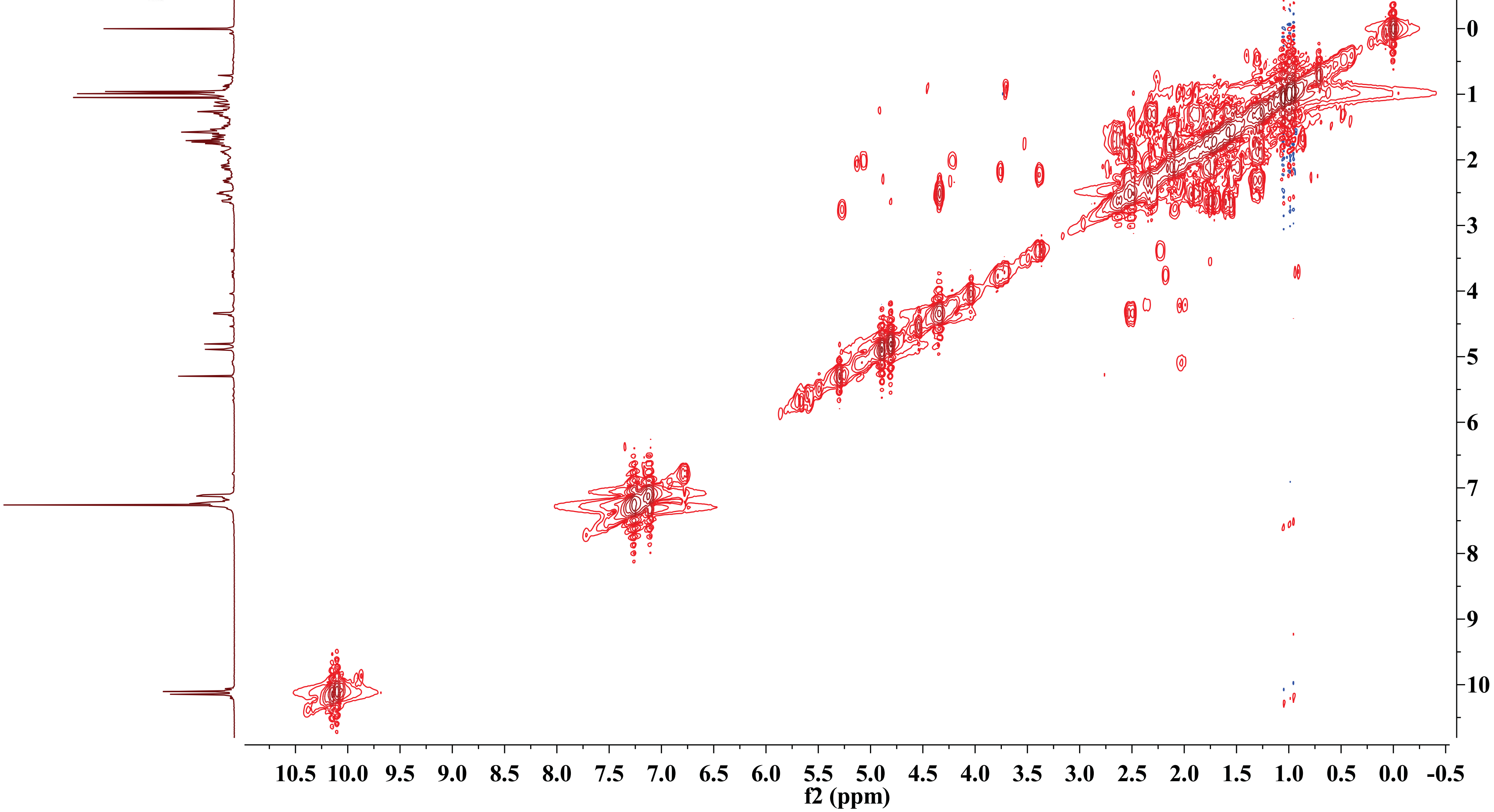

S5.41. HMBC spectrum of compound **7**

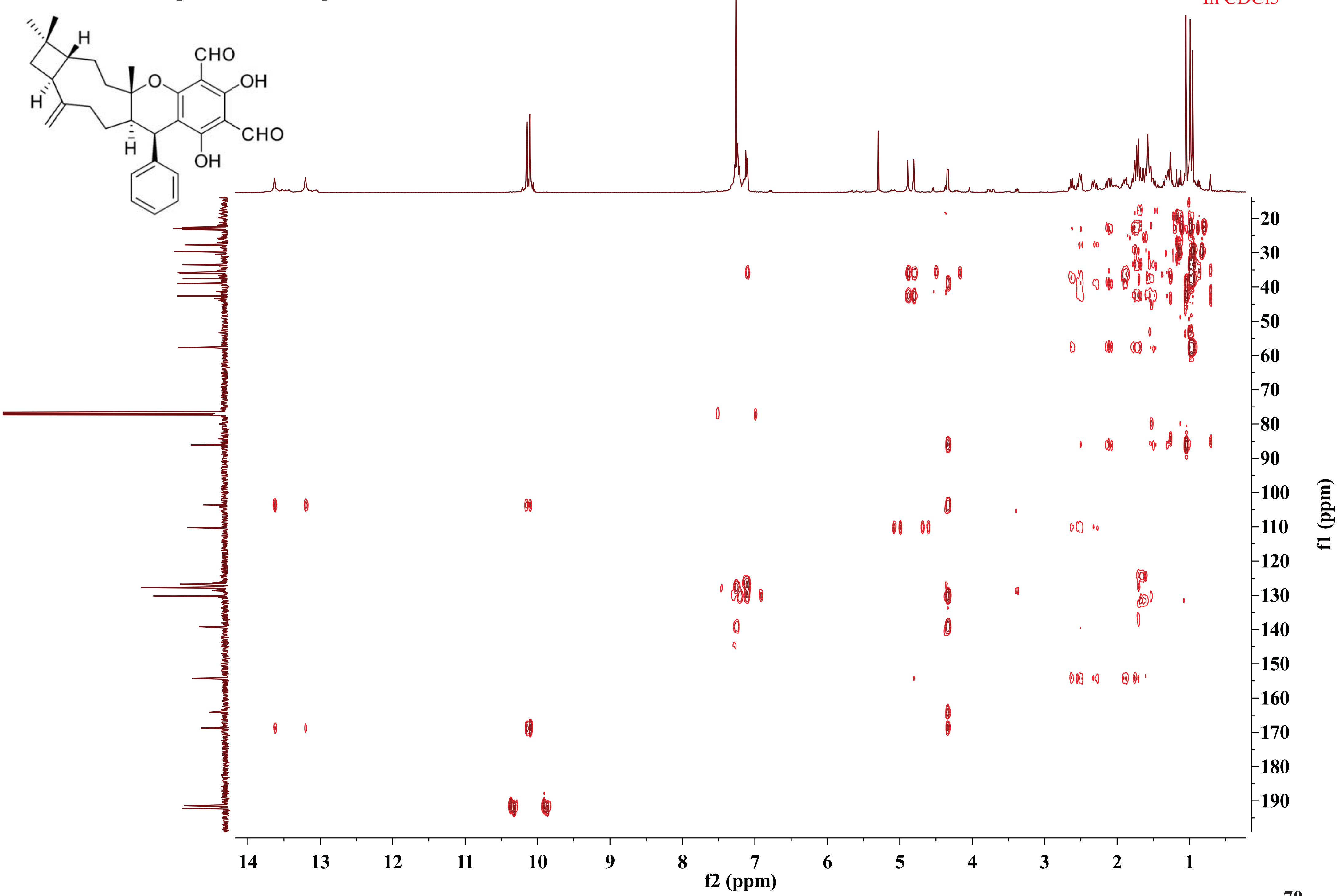

S5.42. NOESY spectrum of compound **7**

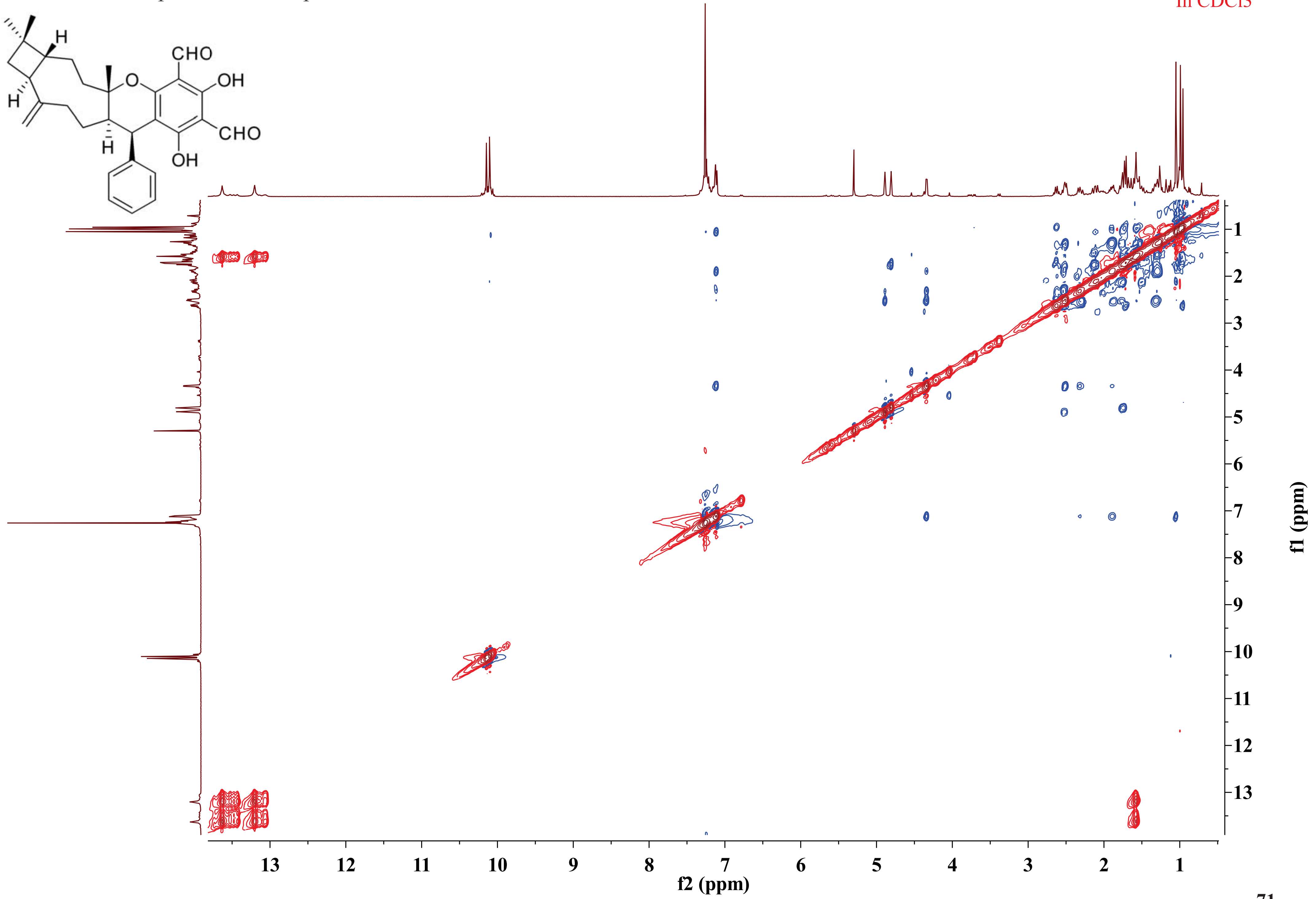

S5.43. ^{1}H NMR spectrum of compound **8**

In CDCl3

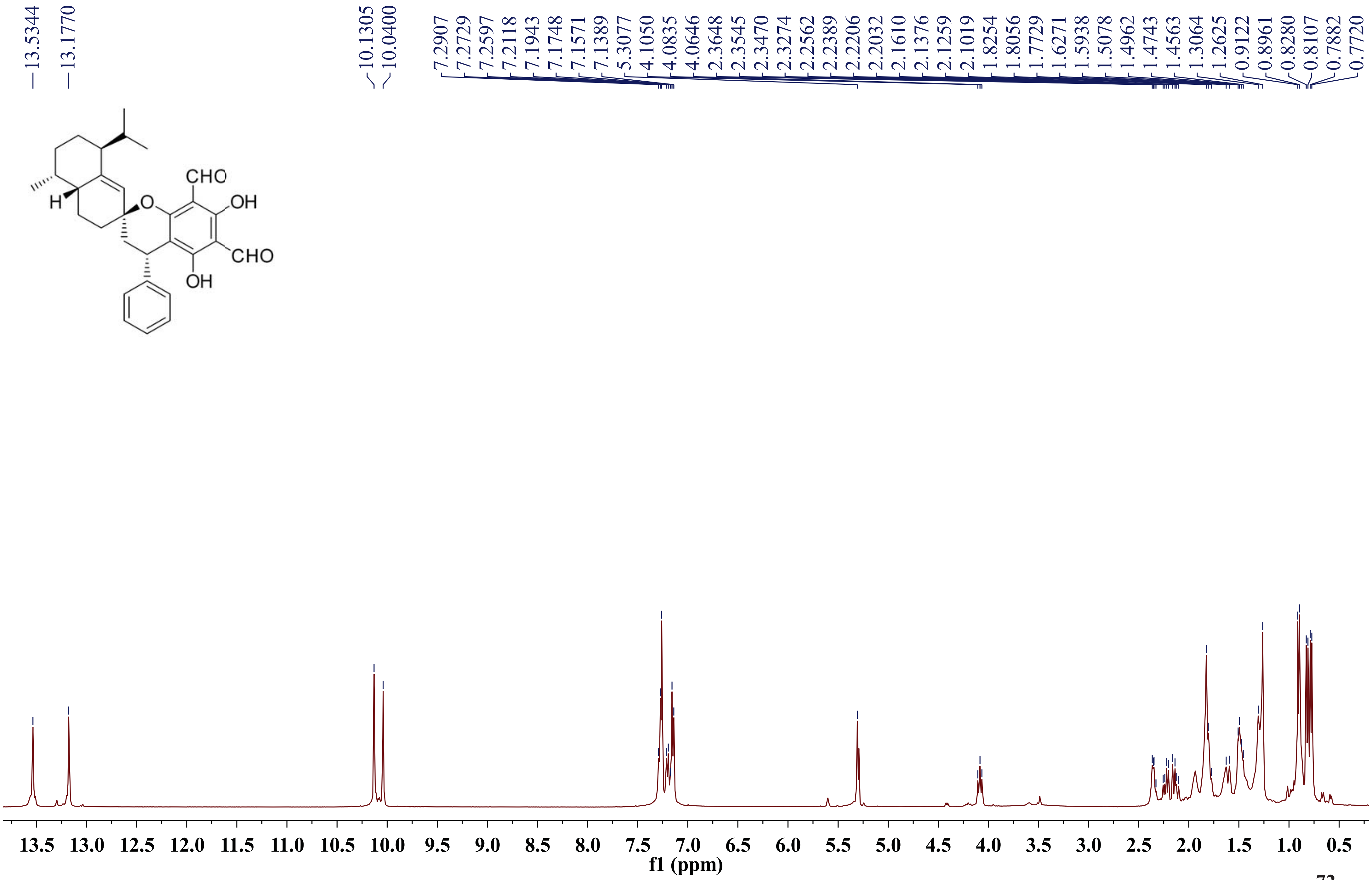

S5.44. DEPT spectra of compound **8**

In CDCl3

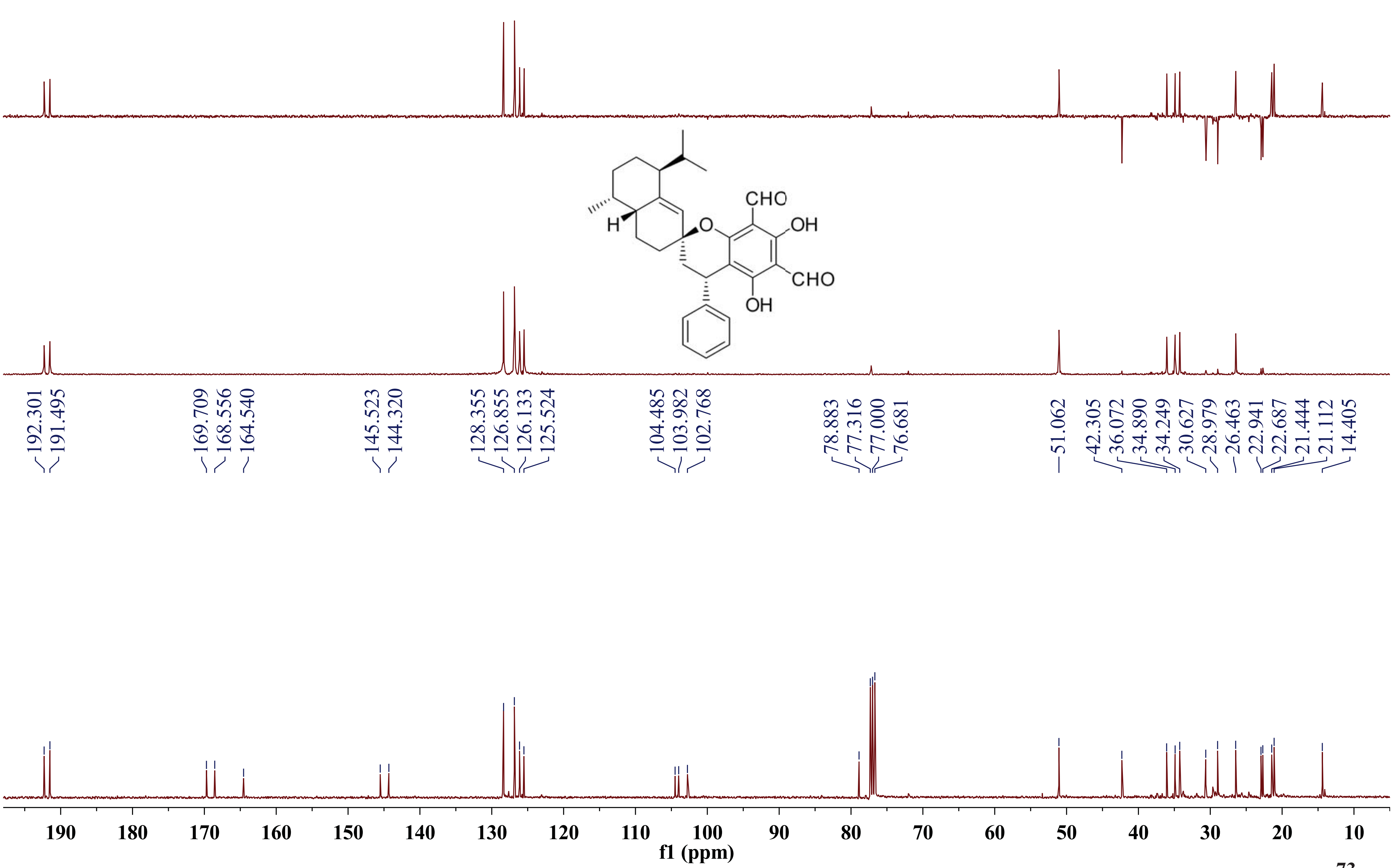

S5.45. HSQC spectrum of compound **8**

In CDCl3

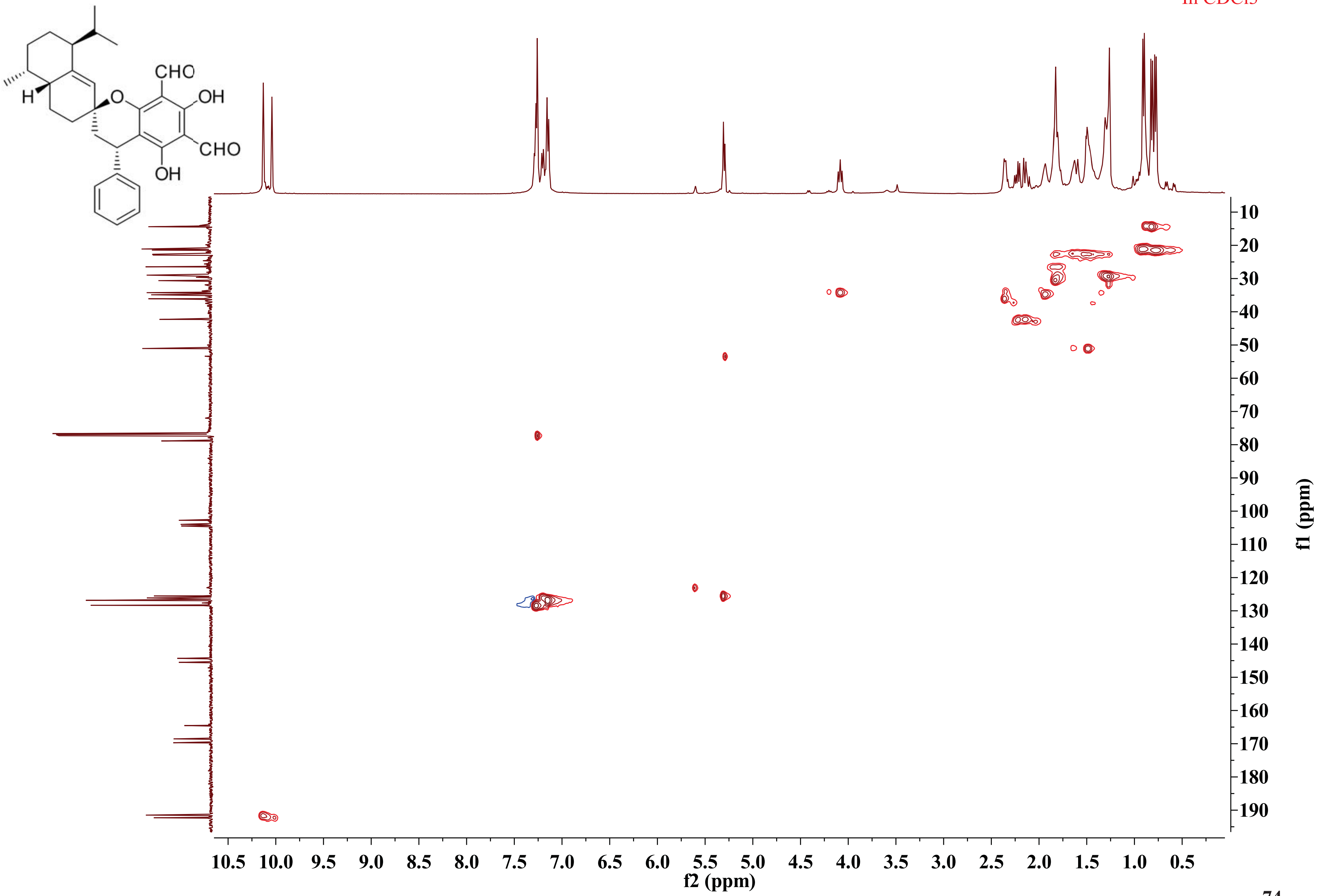

S5.46. ^{1}H-^{1}H COSY spectrum of compound **8**

In CDCl3

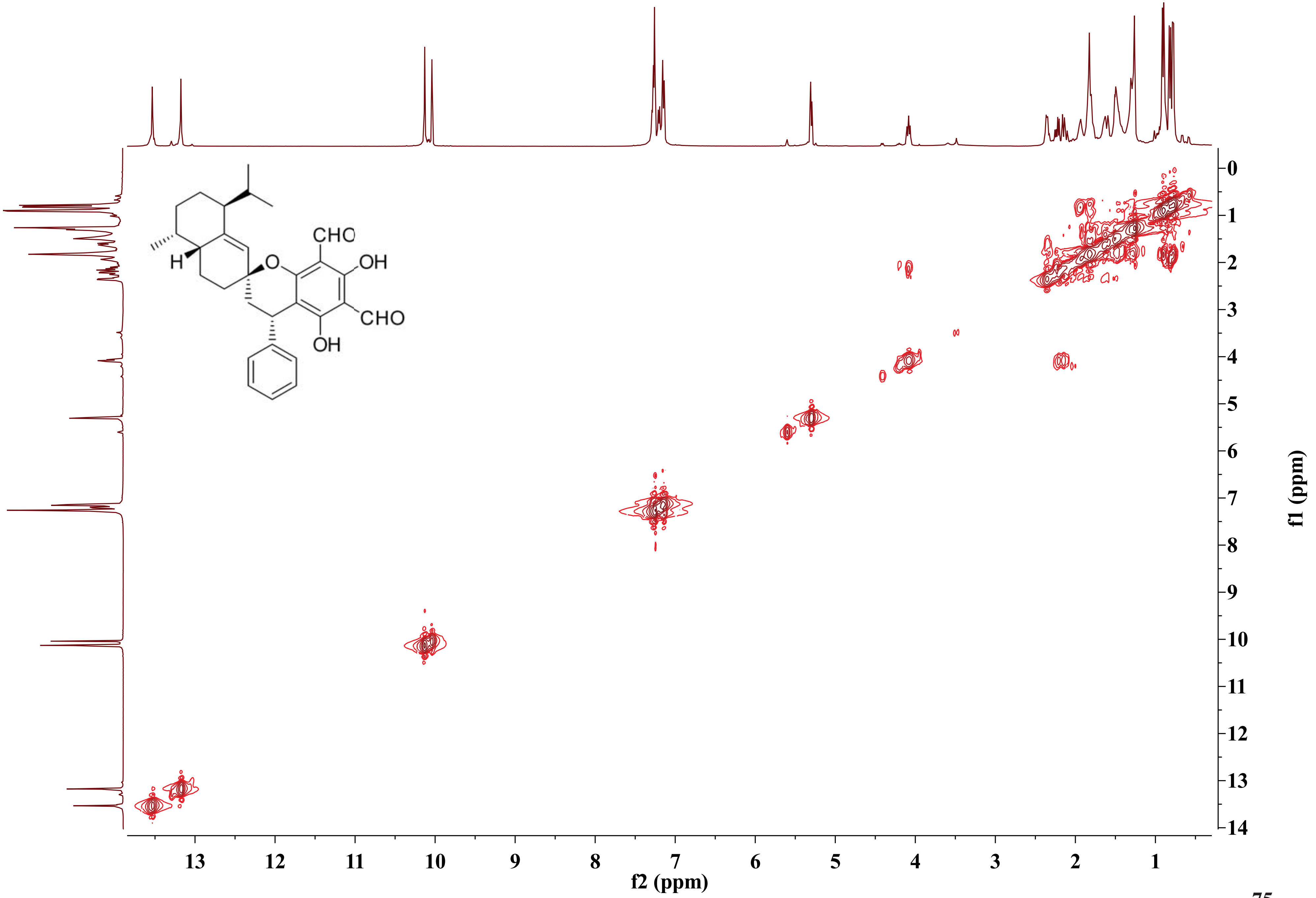

S5.47. HMBC spectrum of compound **8**

In CDCl3

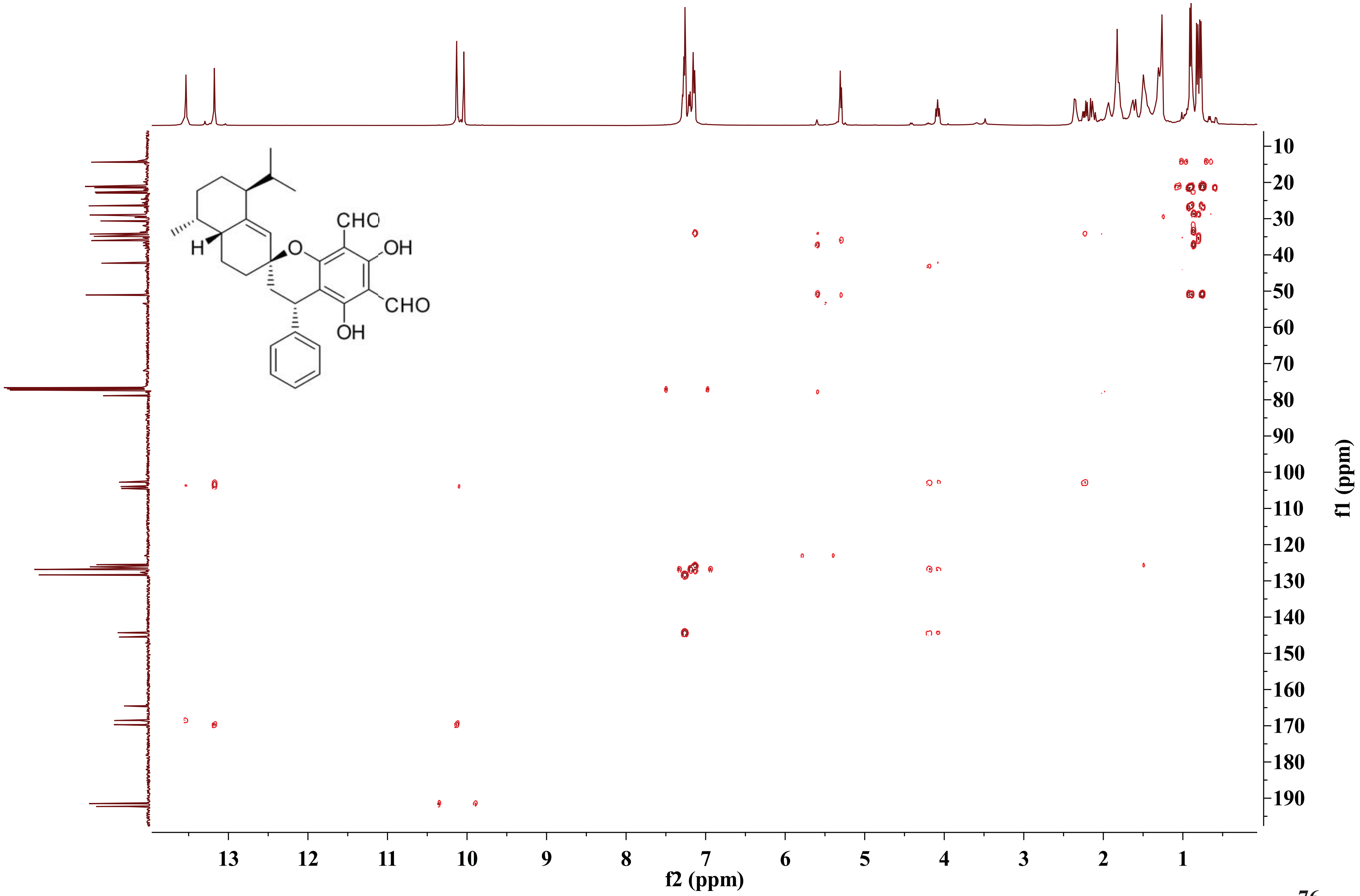

S5.48. NOESY spectrum of compound 8

In CDCl3

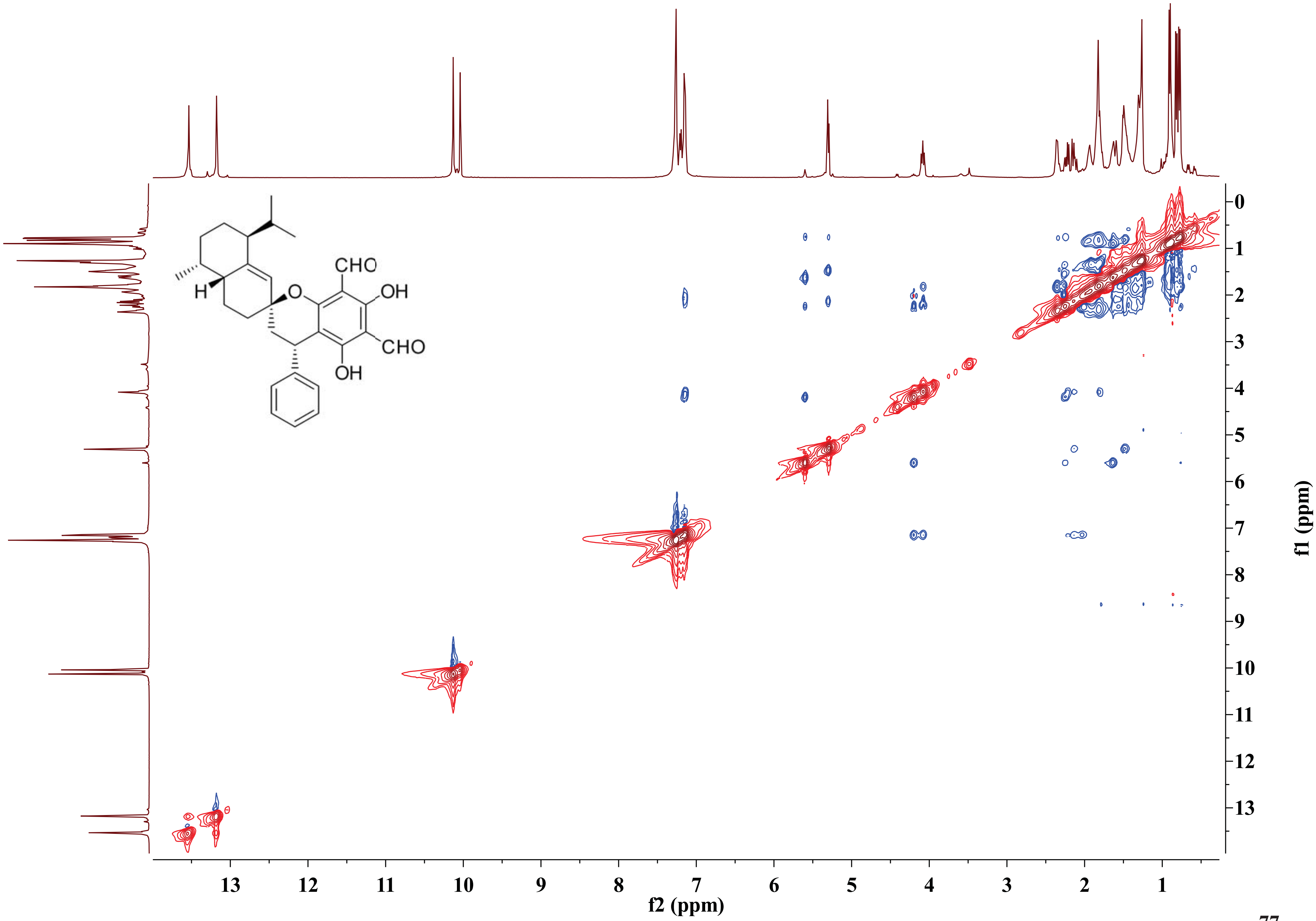

S5.49. ^{1}H NMR spectrum of compound **9**

In CDCl3

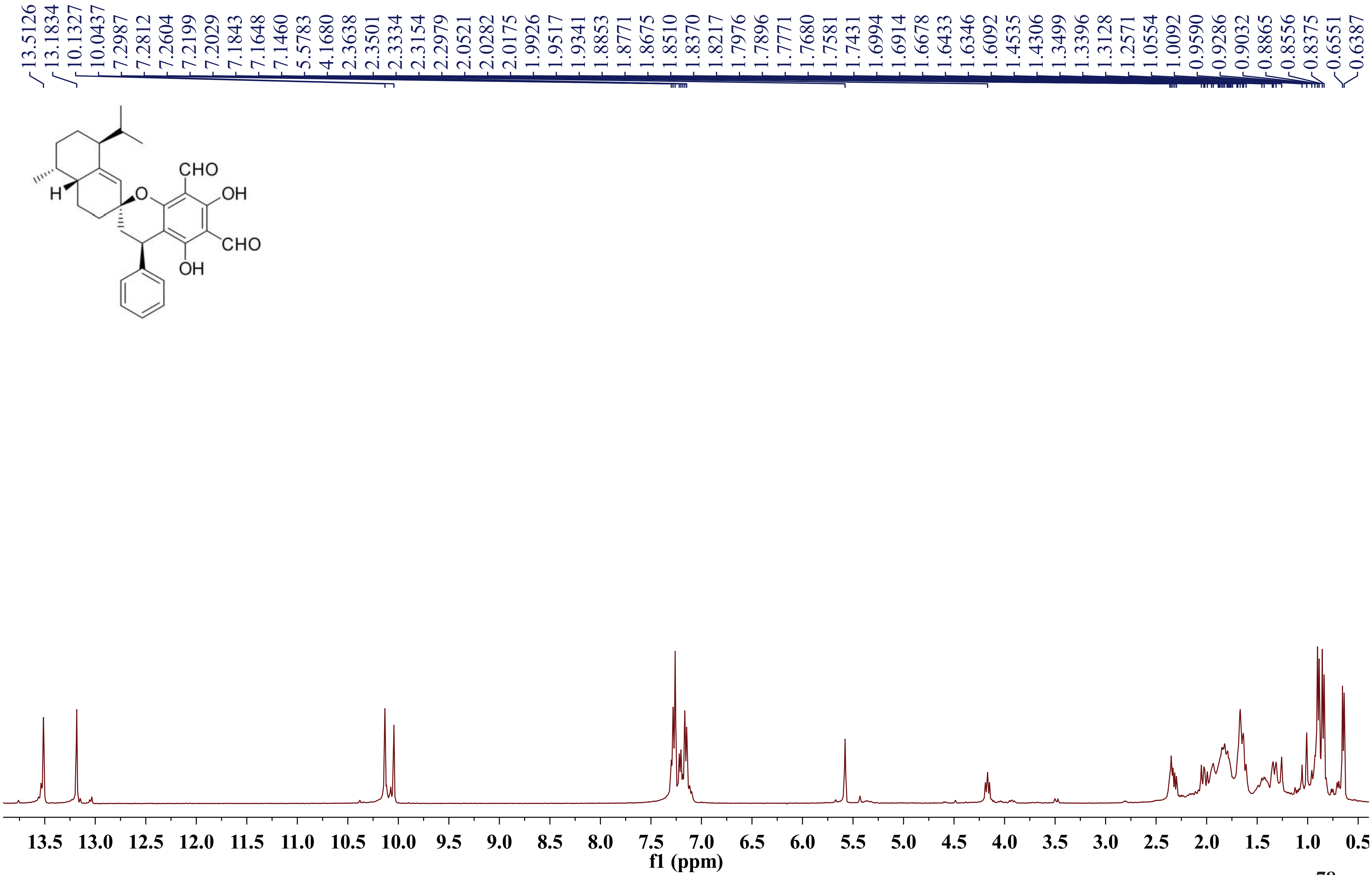

S5.50. DEPT spectra of compound **9**

In CDCl3

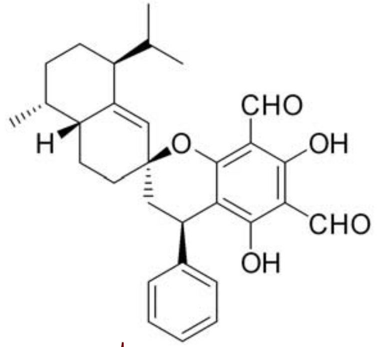

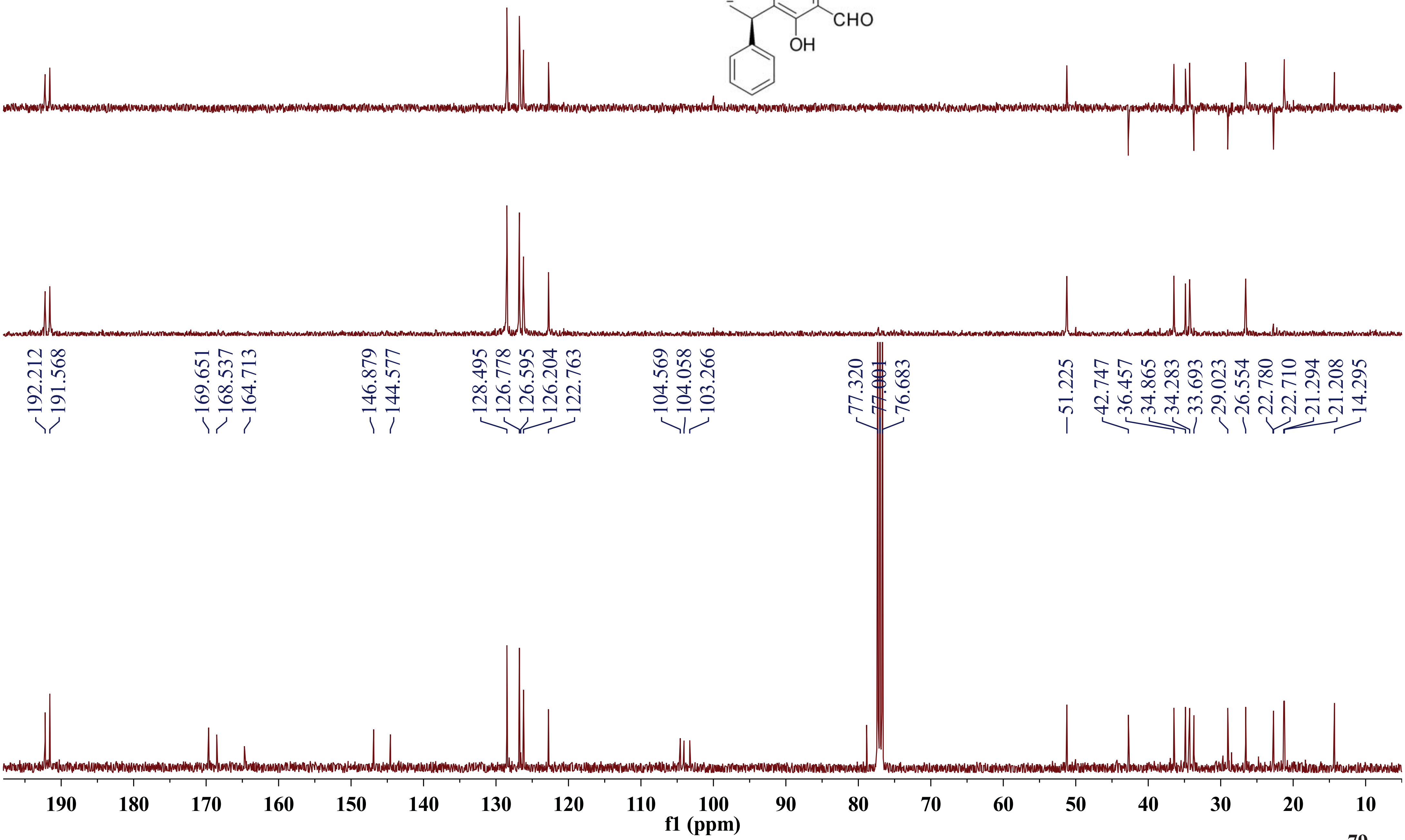

S5.51. HSQC spectrum of compound **9**

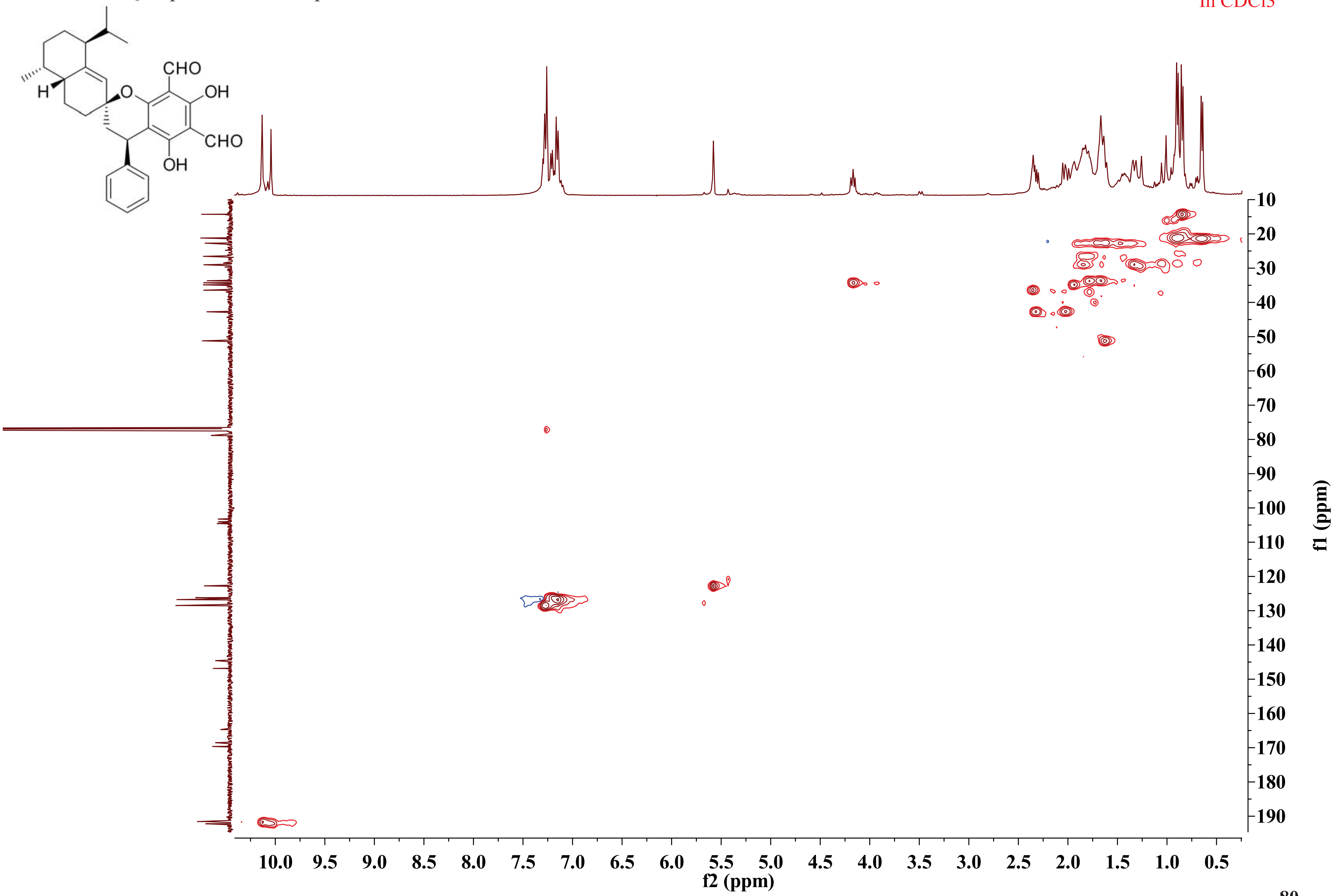

S5.52. ^{1}H-^{1}H COSY spectrum of compound **9**

In CDCl3

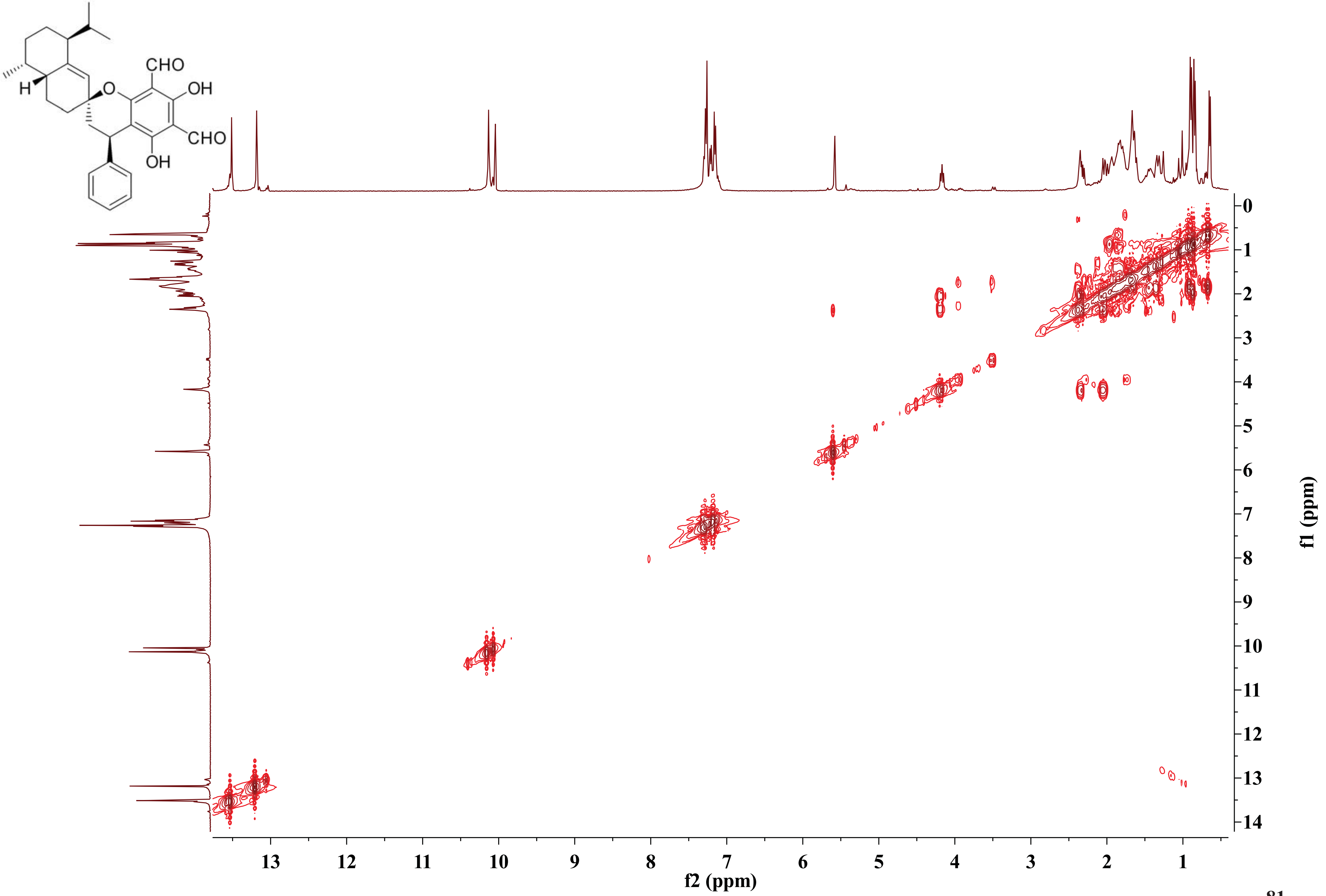

S5.53. HMBC spectrum of compound **9**

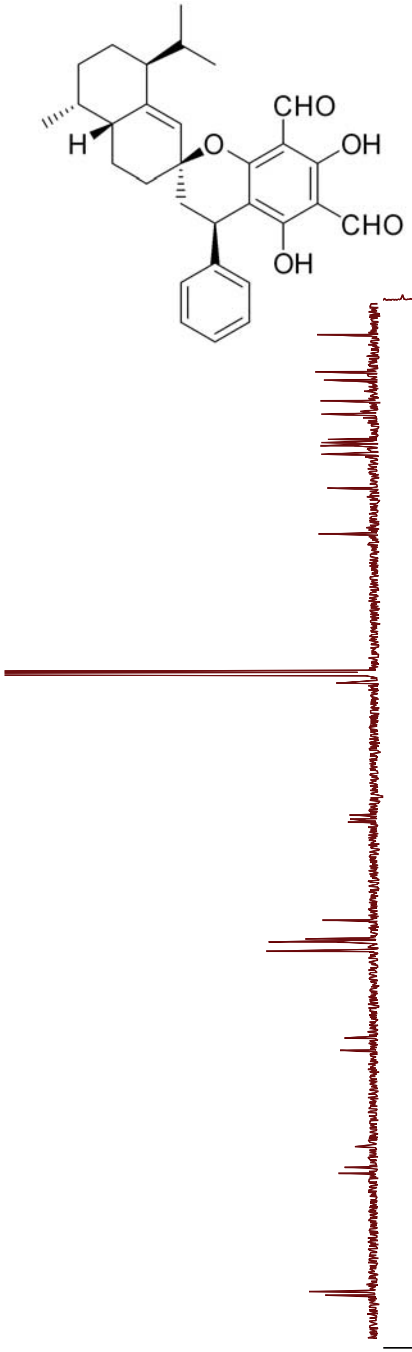

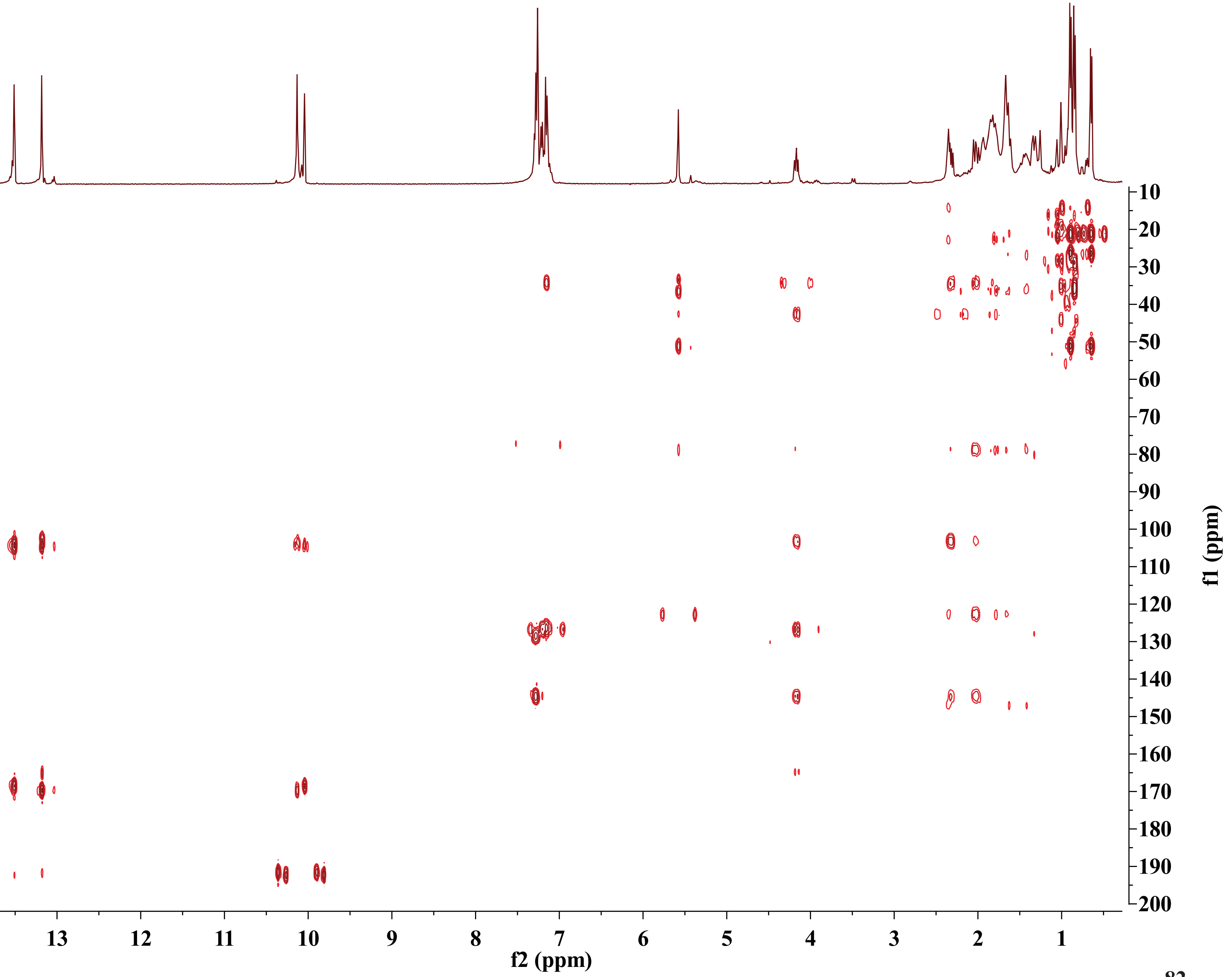

S5.54. NOESY spectrum of compound **9**

In CDCl3

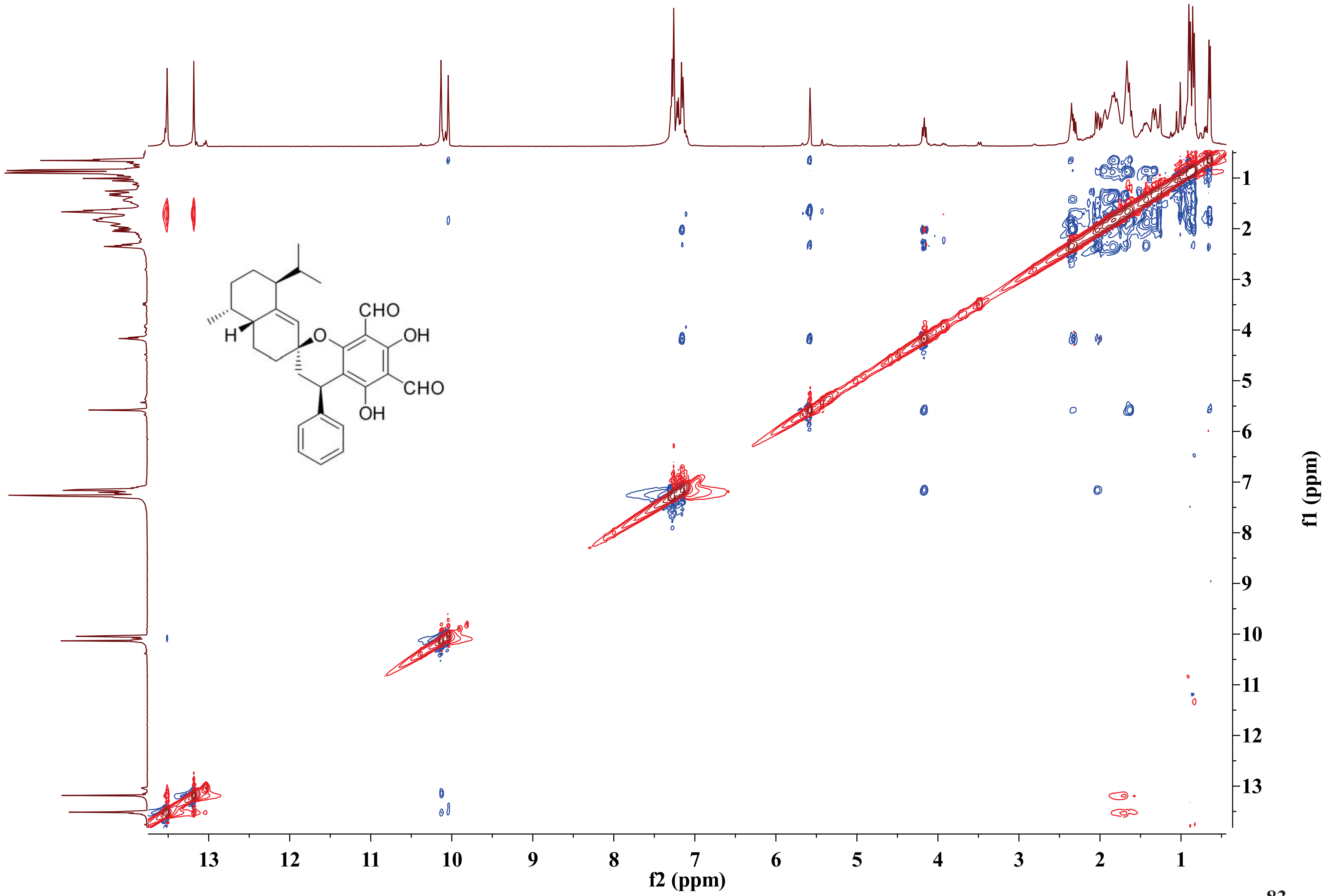

S5.55. ¹H NMR spectrum of compound **10**

In CDCl3

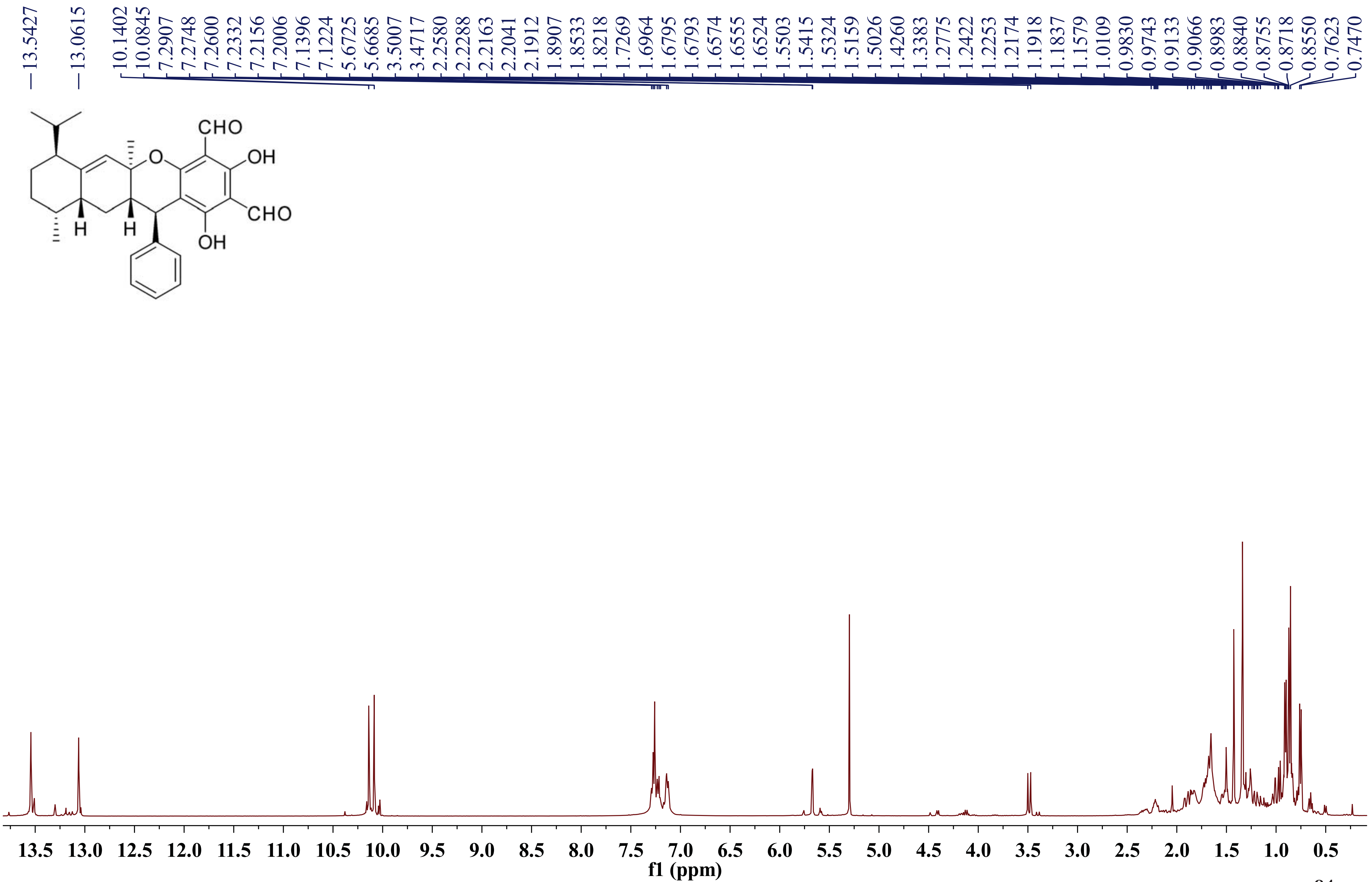

S5.56. DEPT spectra of compound **10**

In CDCl3

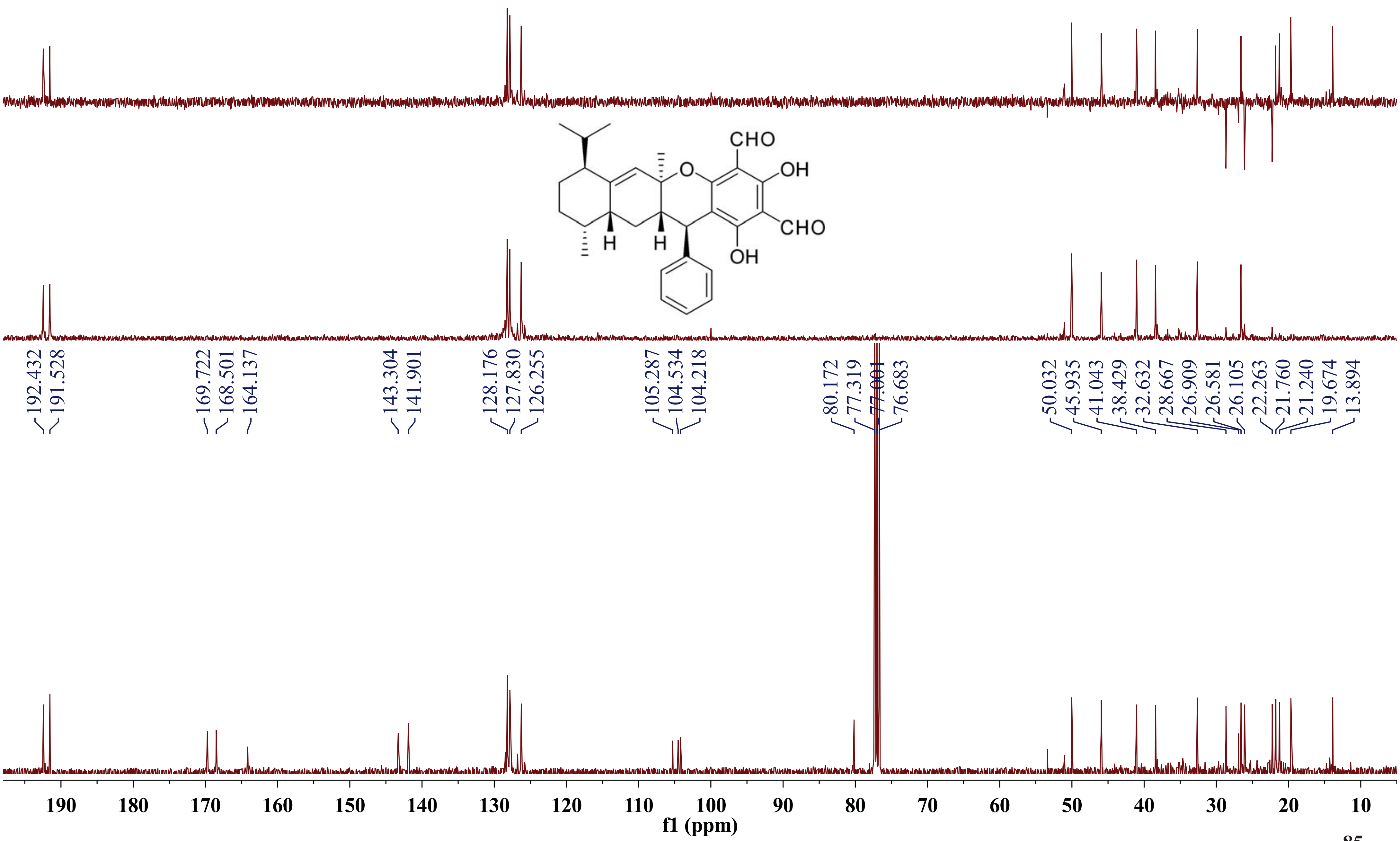

S5.57. HSQC spectrum of compound **10**

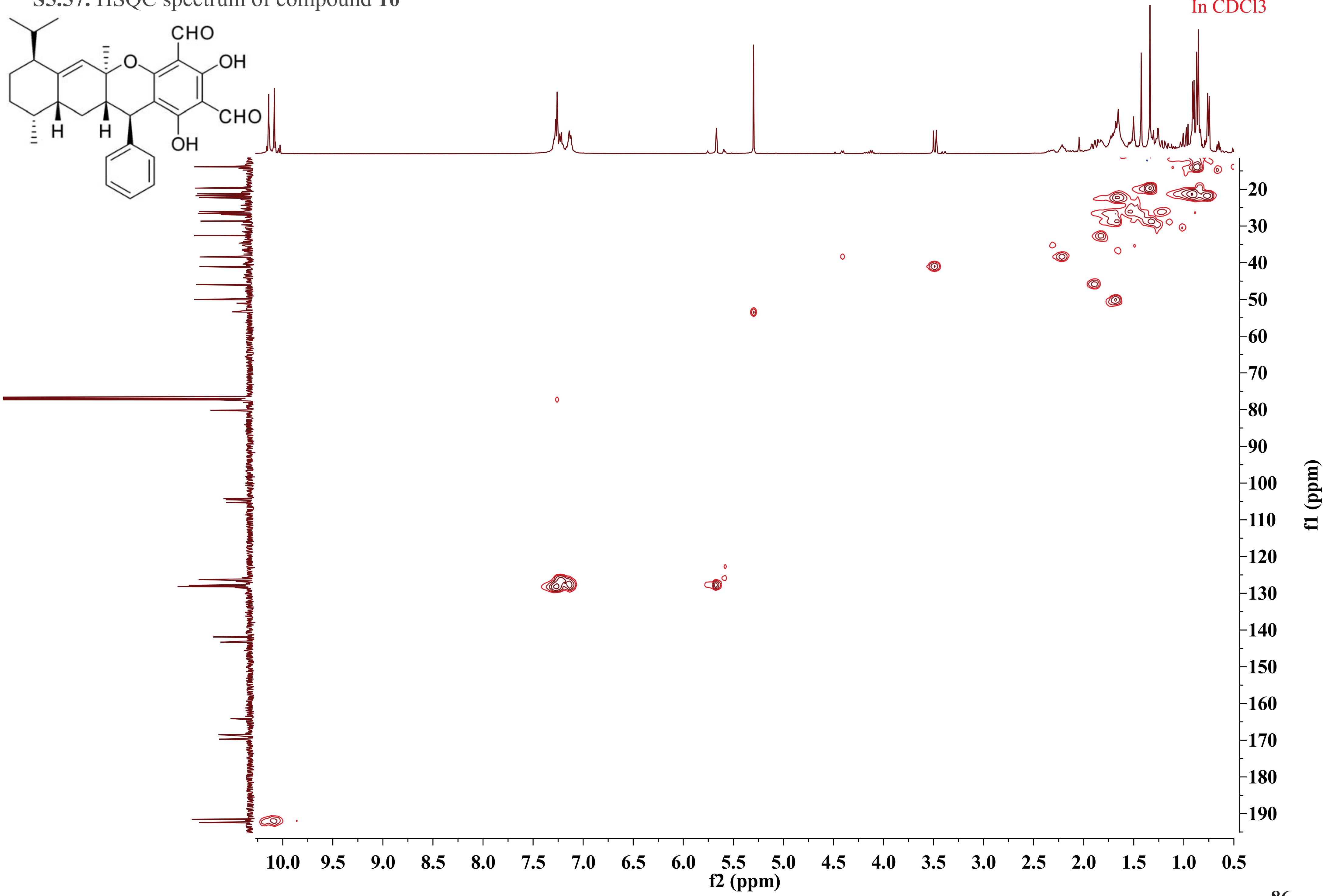

S5.58. ^1H-^1H COSY spectrum of compound **10**

In CDCl3

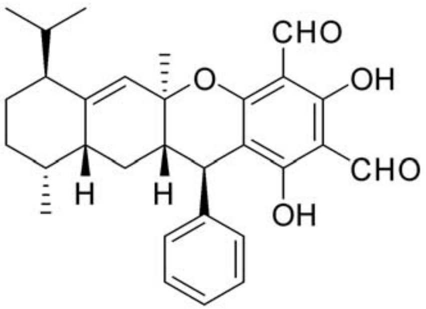

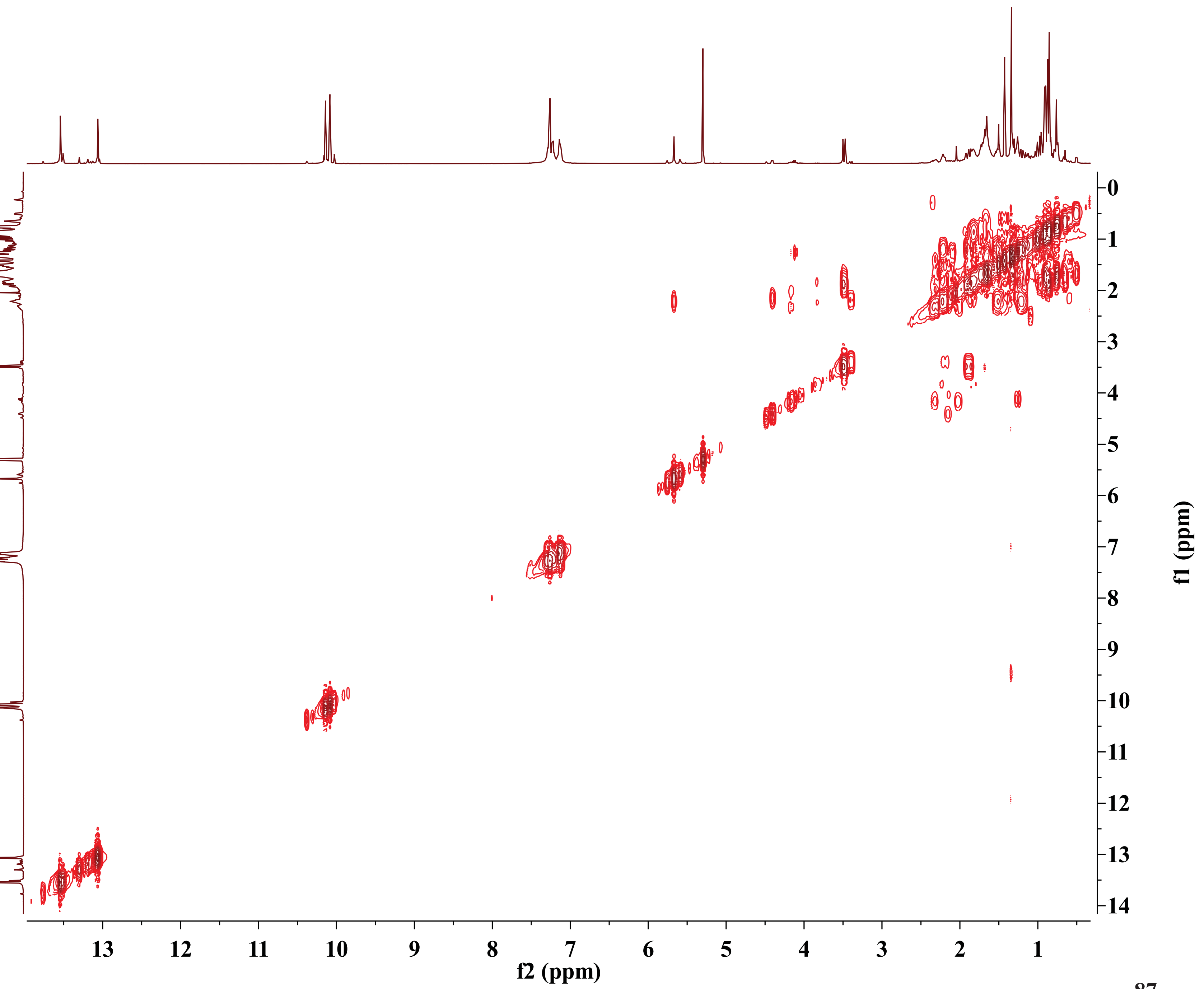

S5.59. HMBC spectrum of compound **10**

In CDCl3

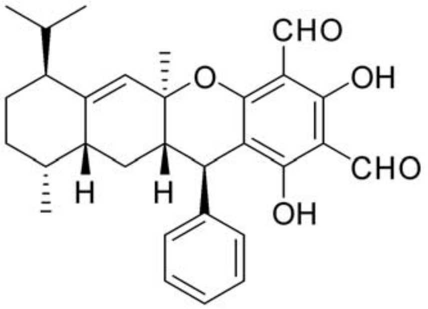

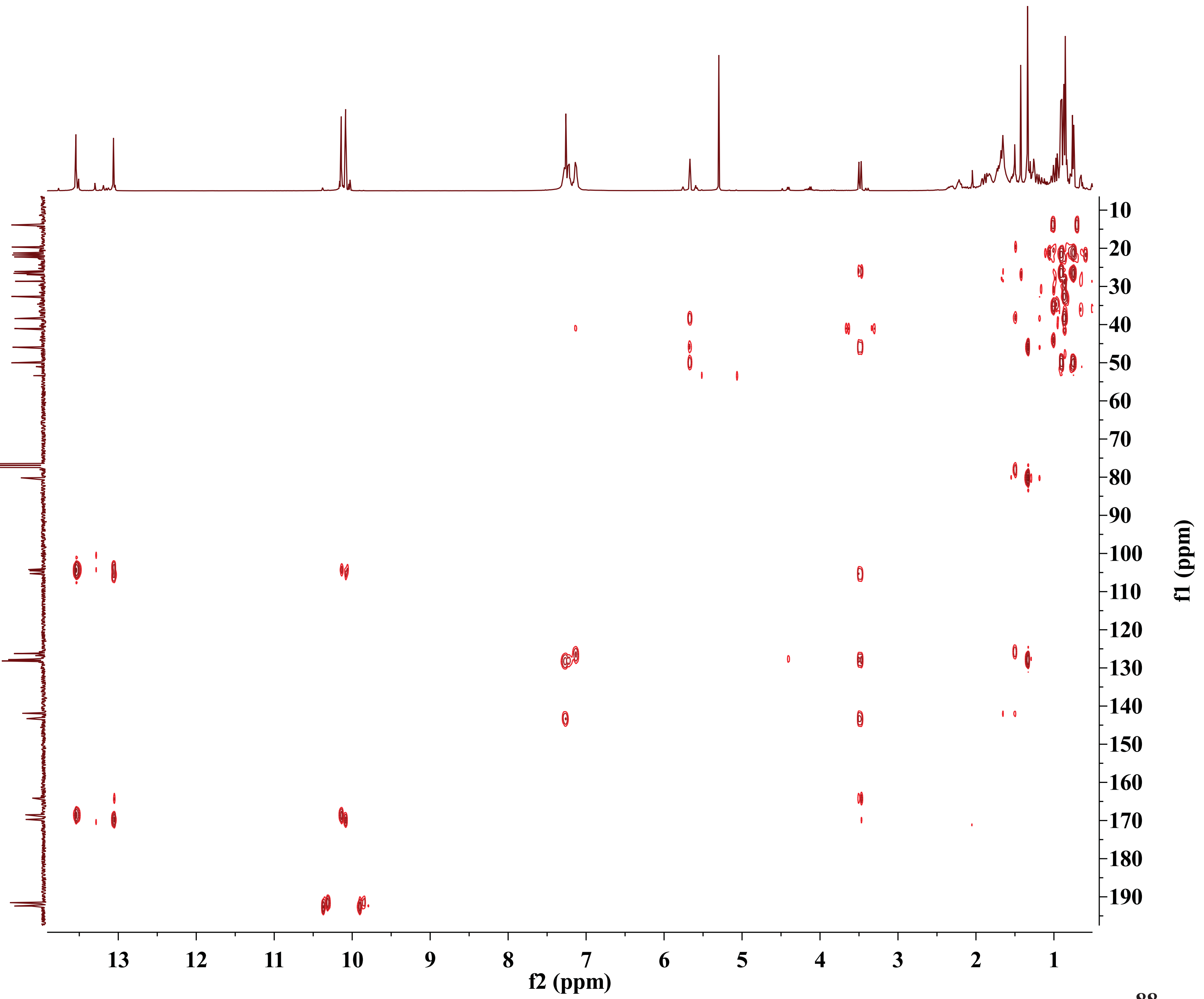

S5.60. NOESY spectrum of compound **10**

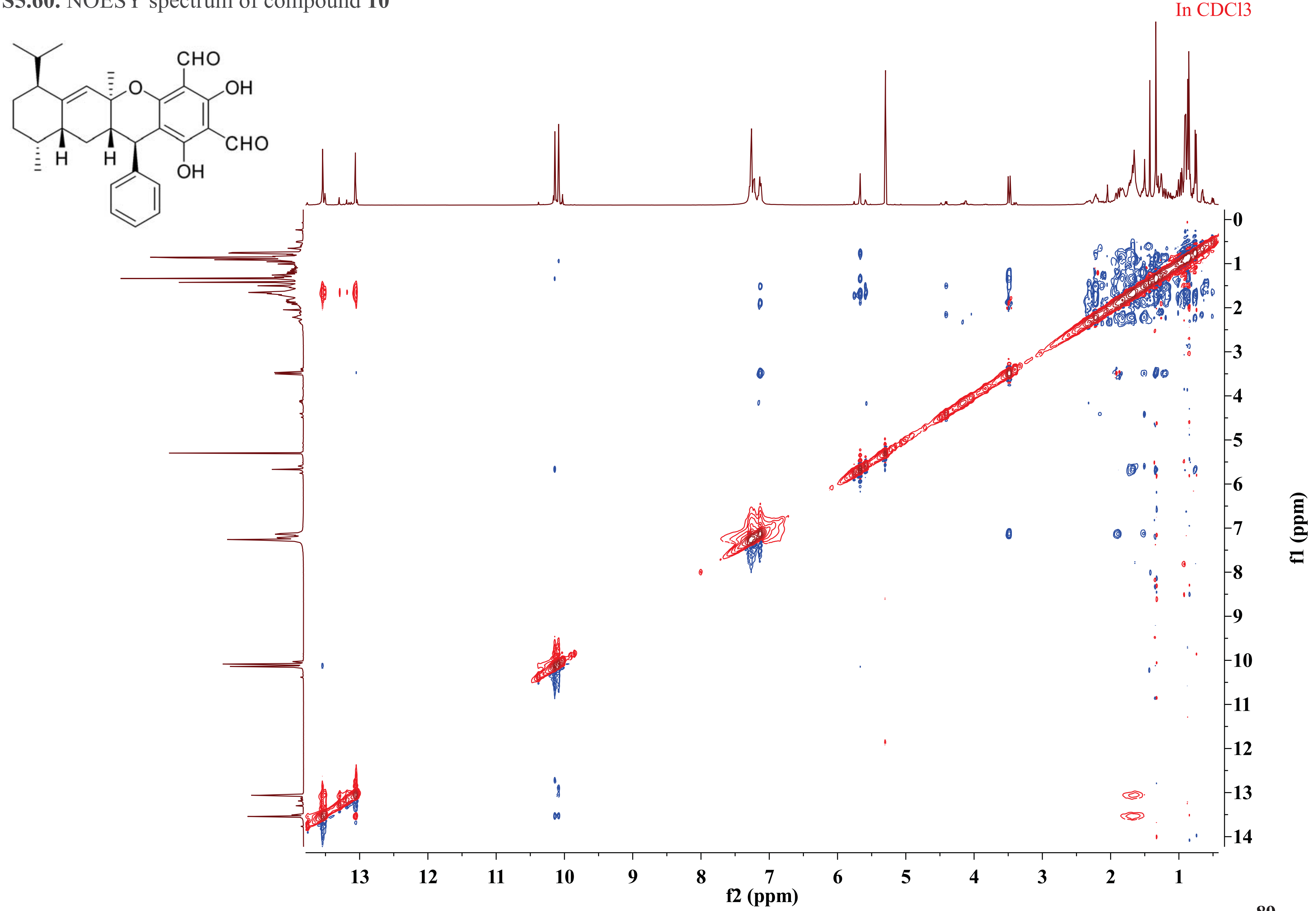

S5.61. ^{1}H NMR spectrum of compound **11**

In CDCl3

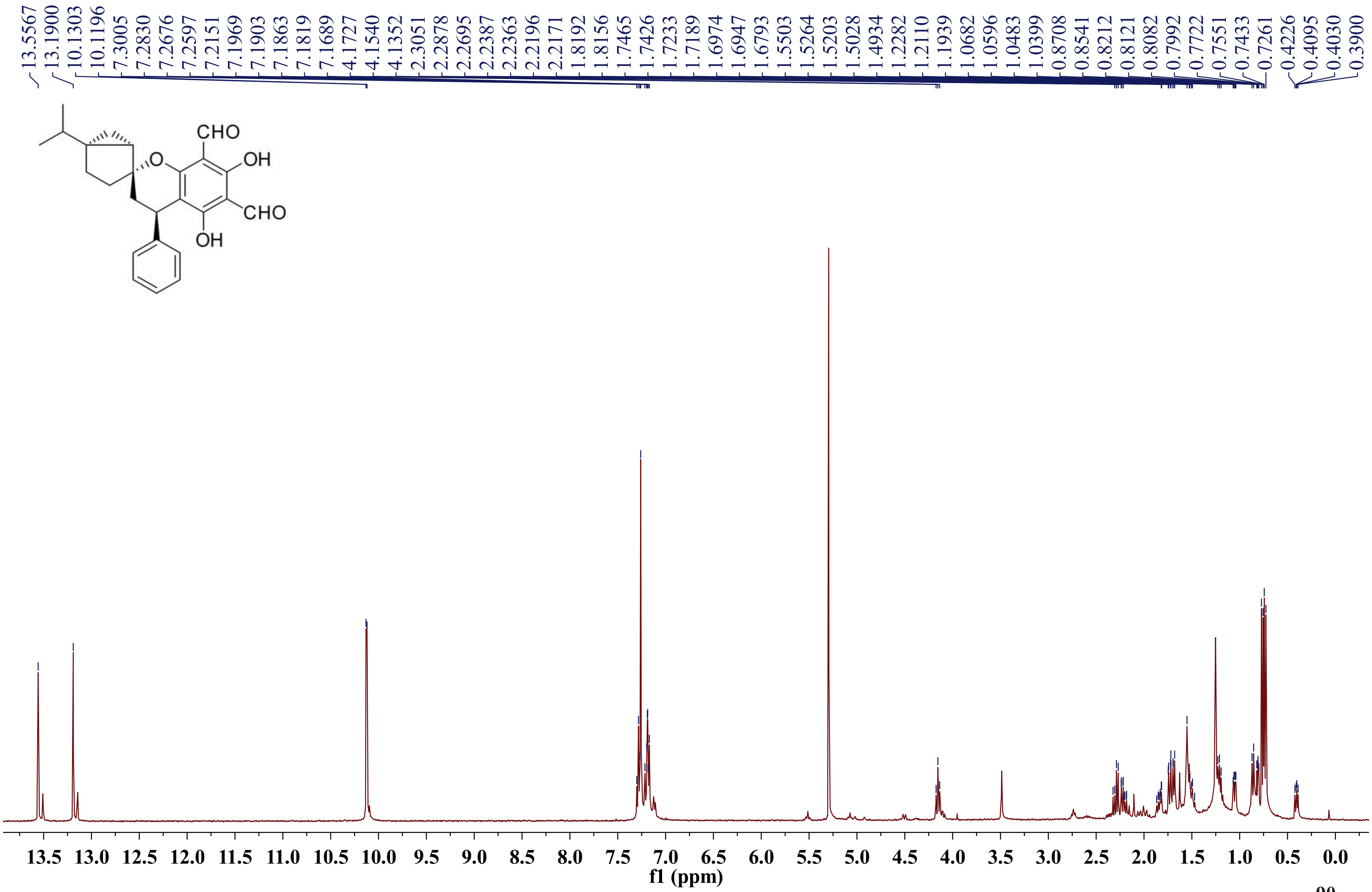

S5.62. DEPT spectra of compound **11**

In CDCl3

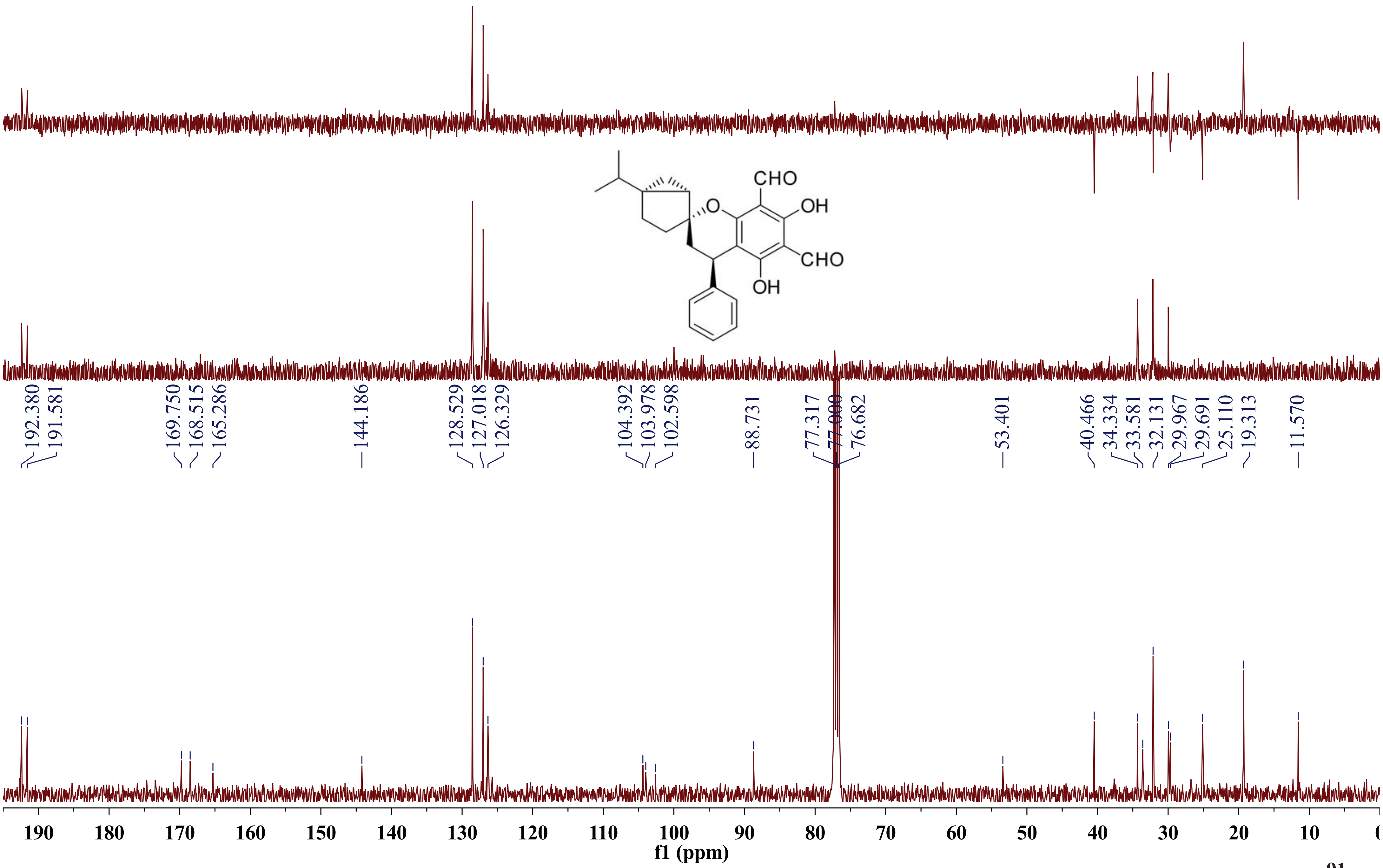

S5.63. HSQC spectrum of compound **11**

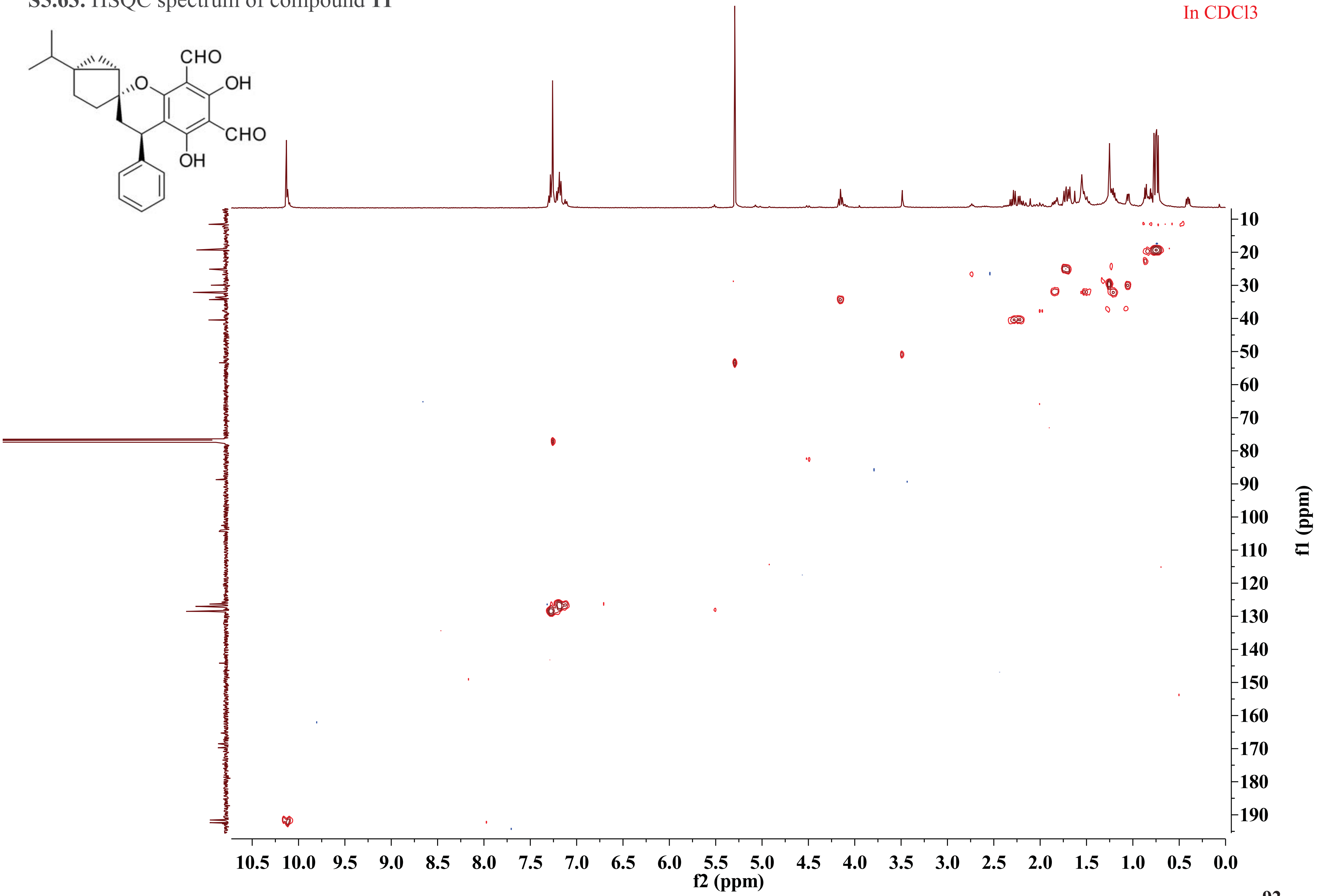

S5.64. ${}^{1}H$-${}^{1}H$ COSY spectrum of compound **11**

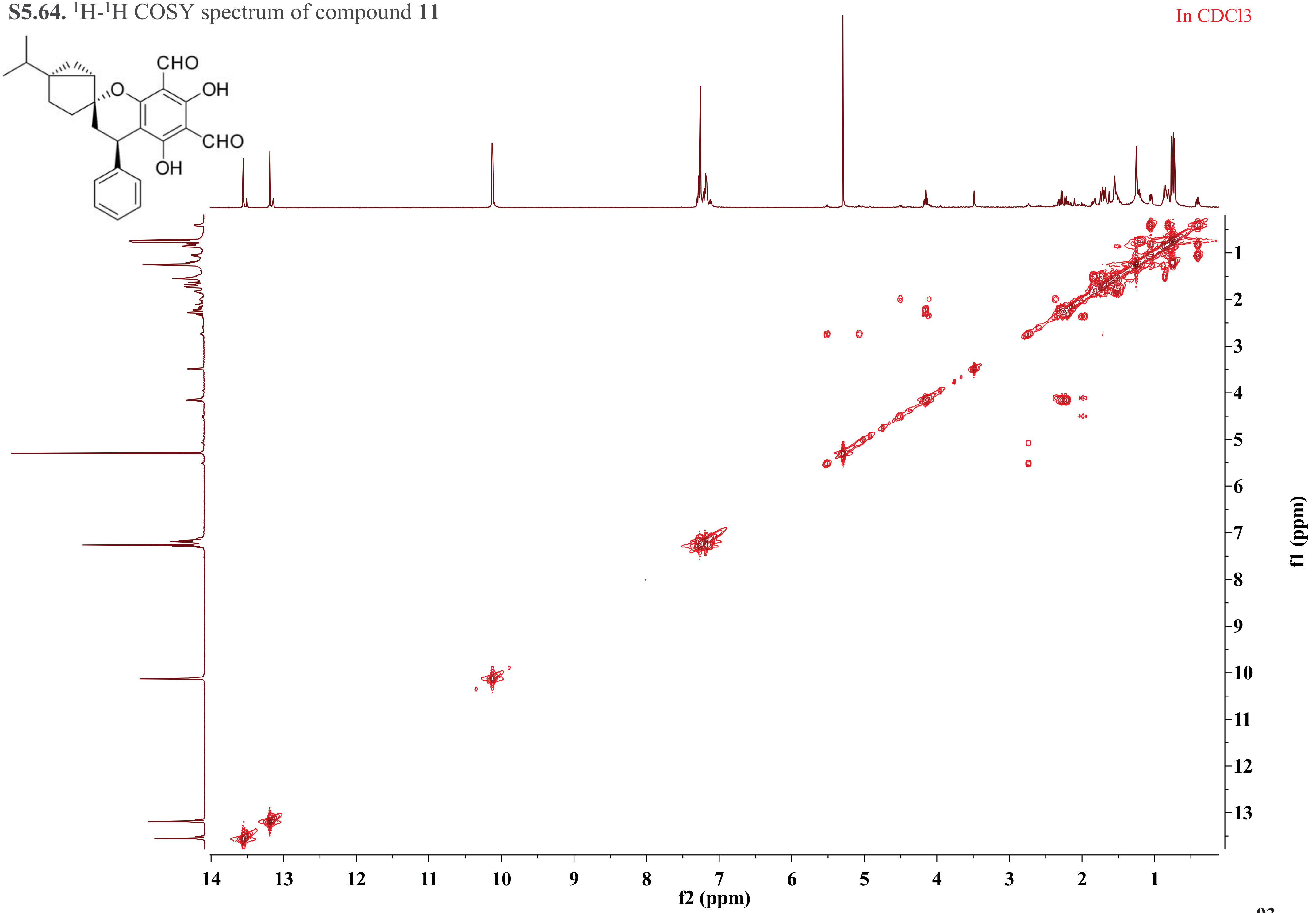

S5.65. HMBC spectrum of compound **11**

In CDCl3

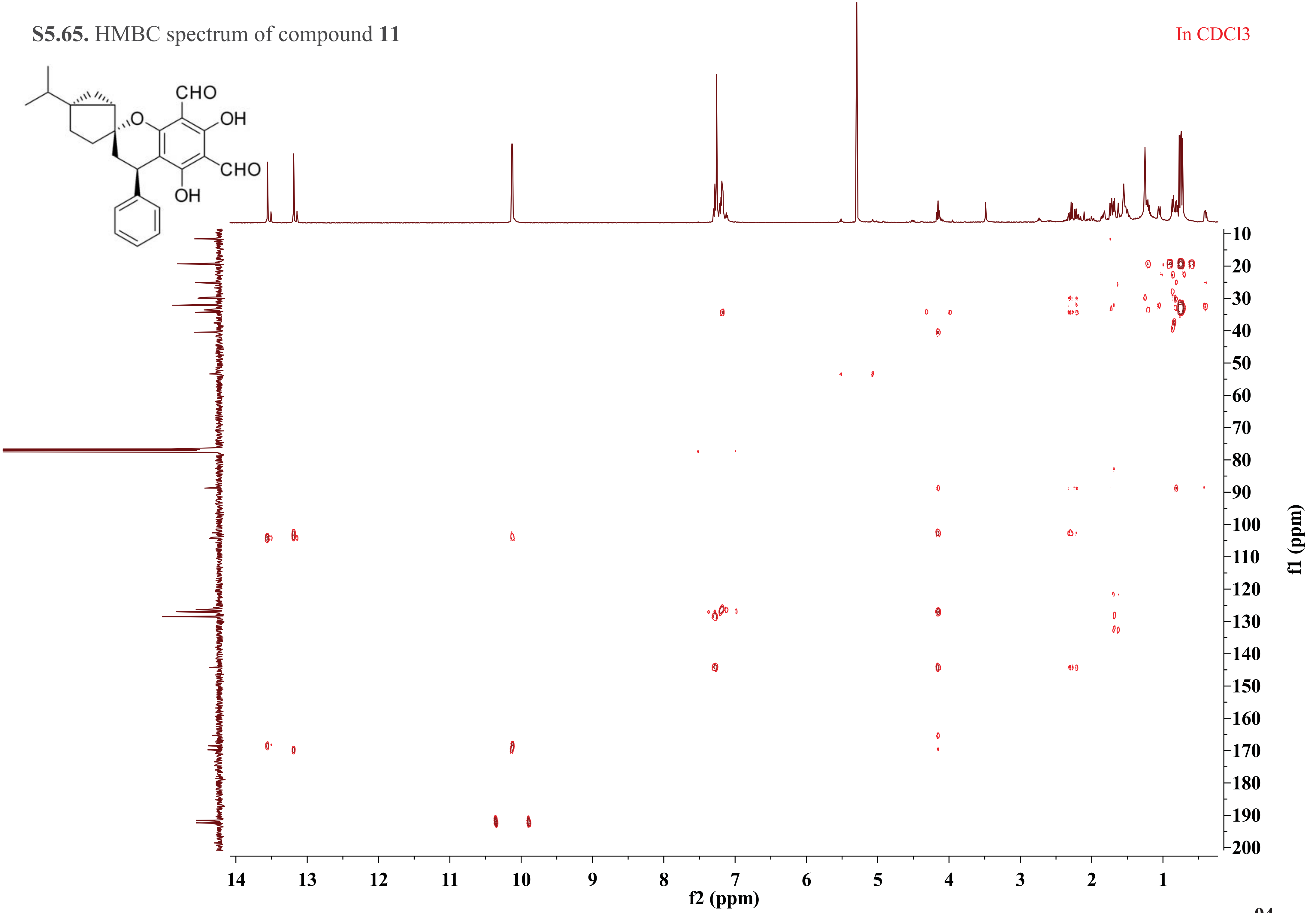

S5.66. NOESY spectrum of compound **11**

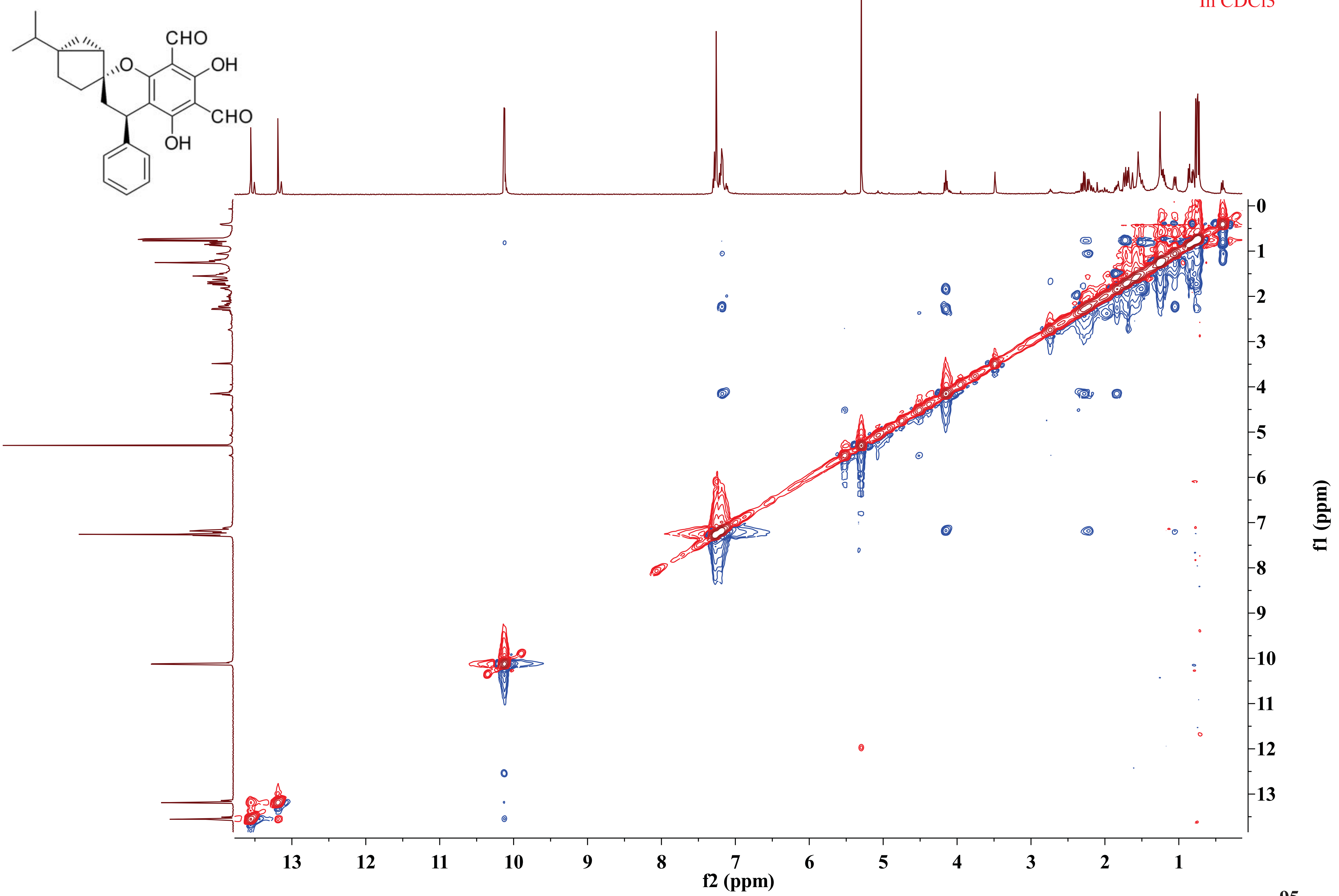

S6.1. ^{1}H NMR spectrum of compound **1**

In pyridine-d5

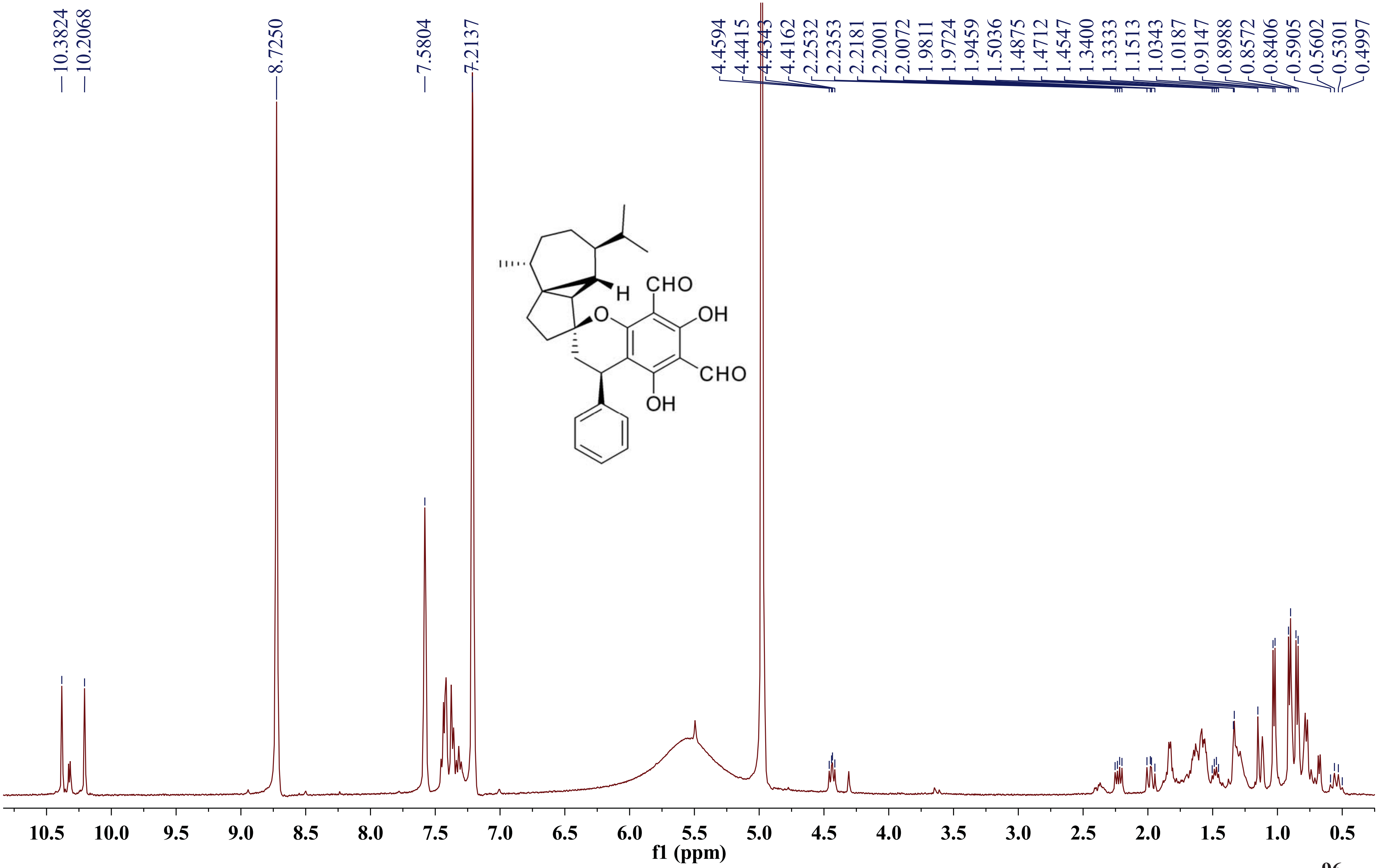

S6.2. DEPT spectra of compound **1**

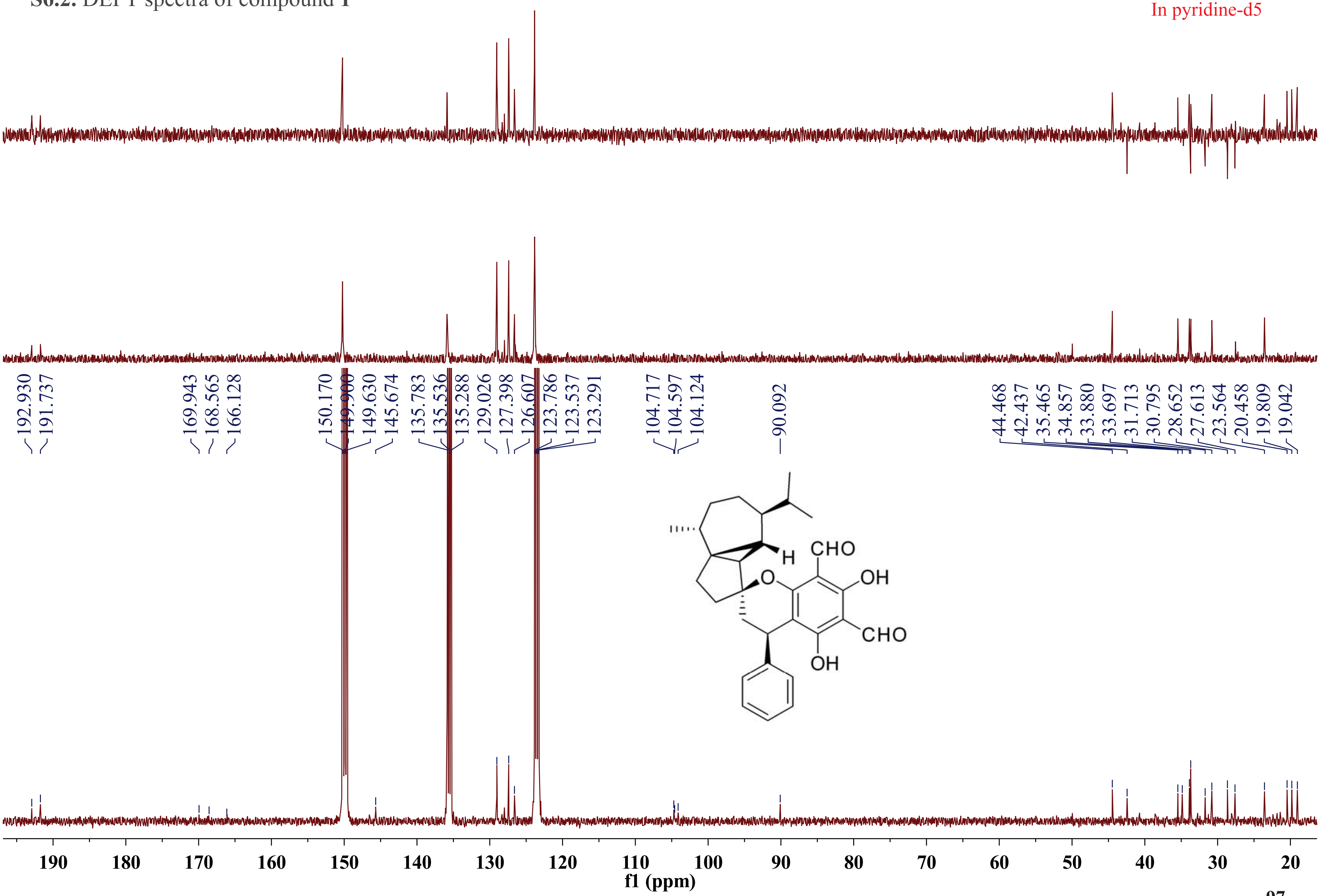

S6.3. HSQC spectrum of compound **1**

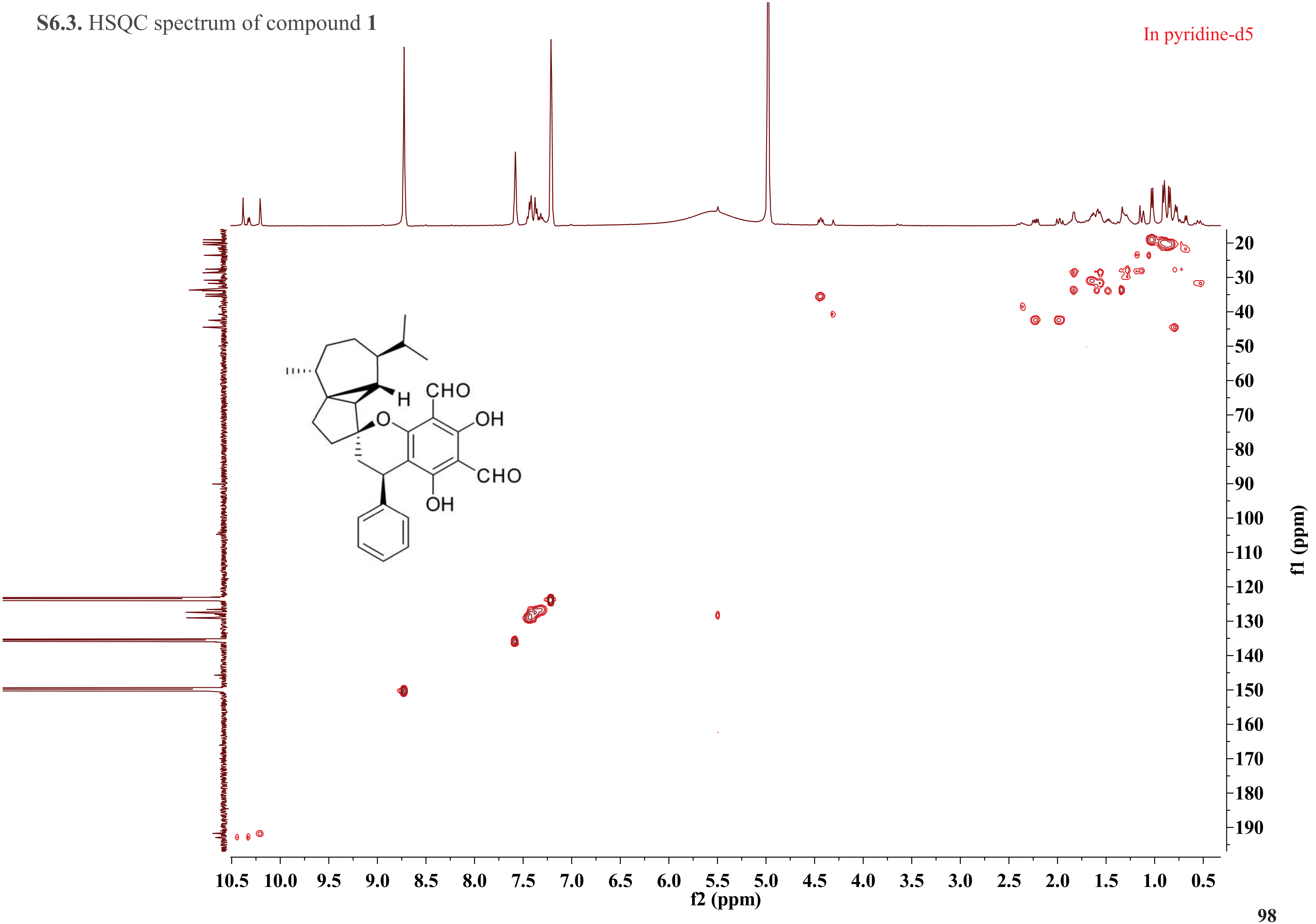

S6.4. ^1H-^1H COSY spectrum of compound **1**

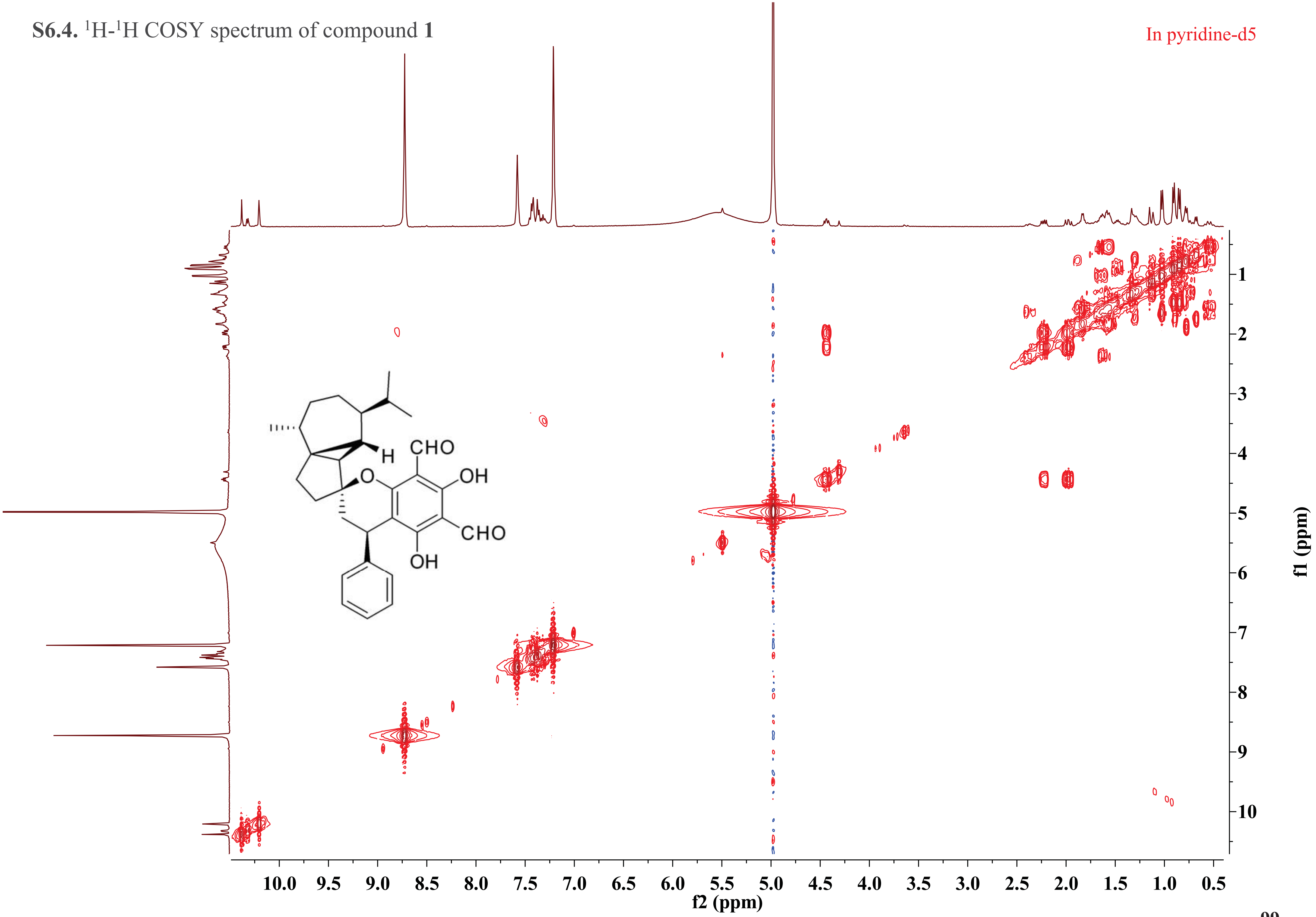

S6.5. HMBC spectrum of compound **1**

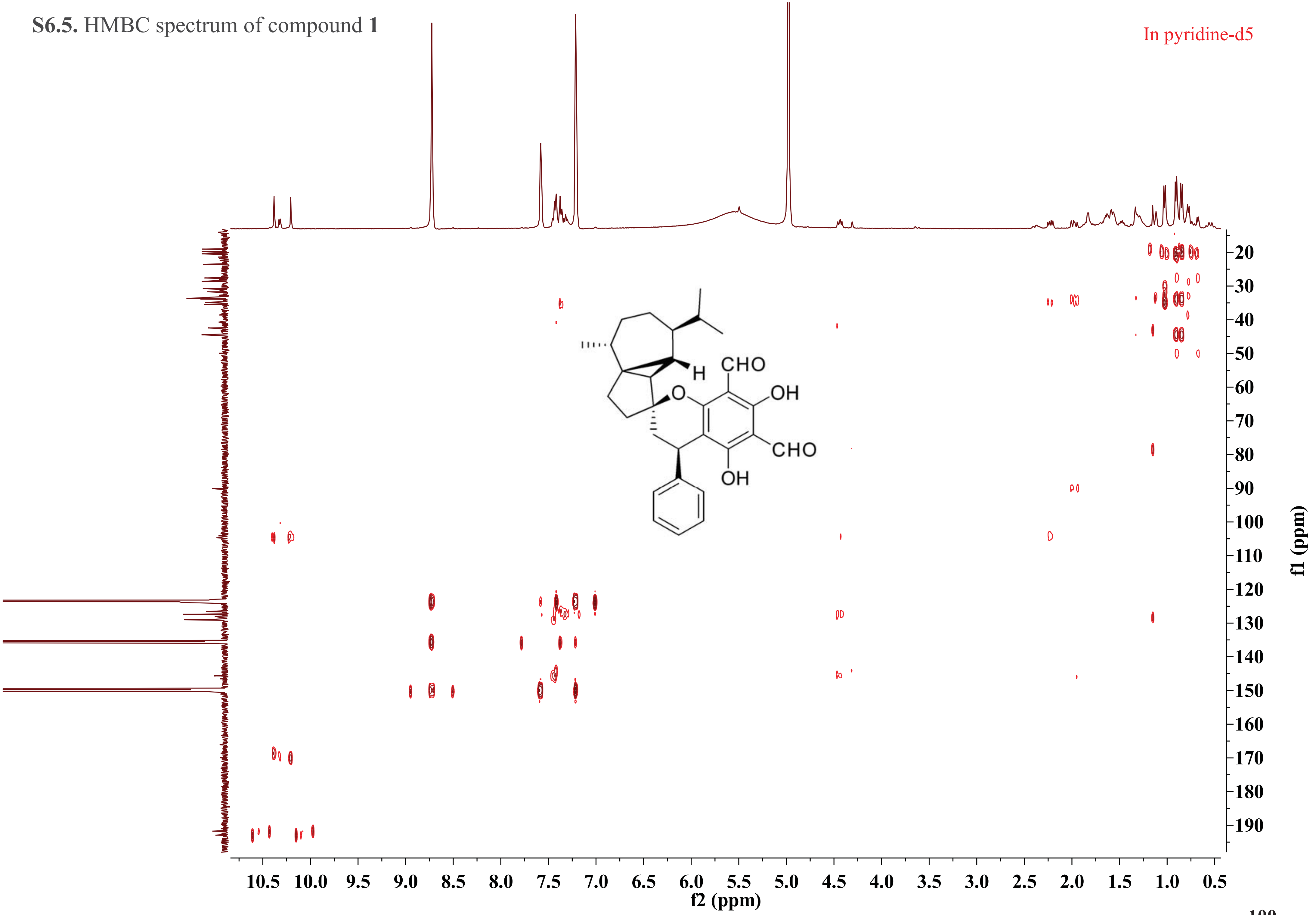

S6.6. NOESY spectrum of compound **1**

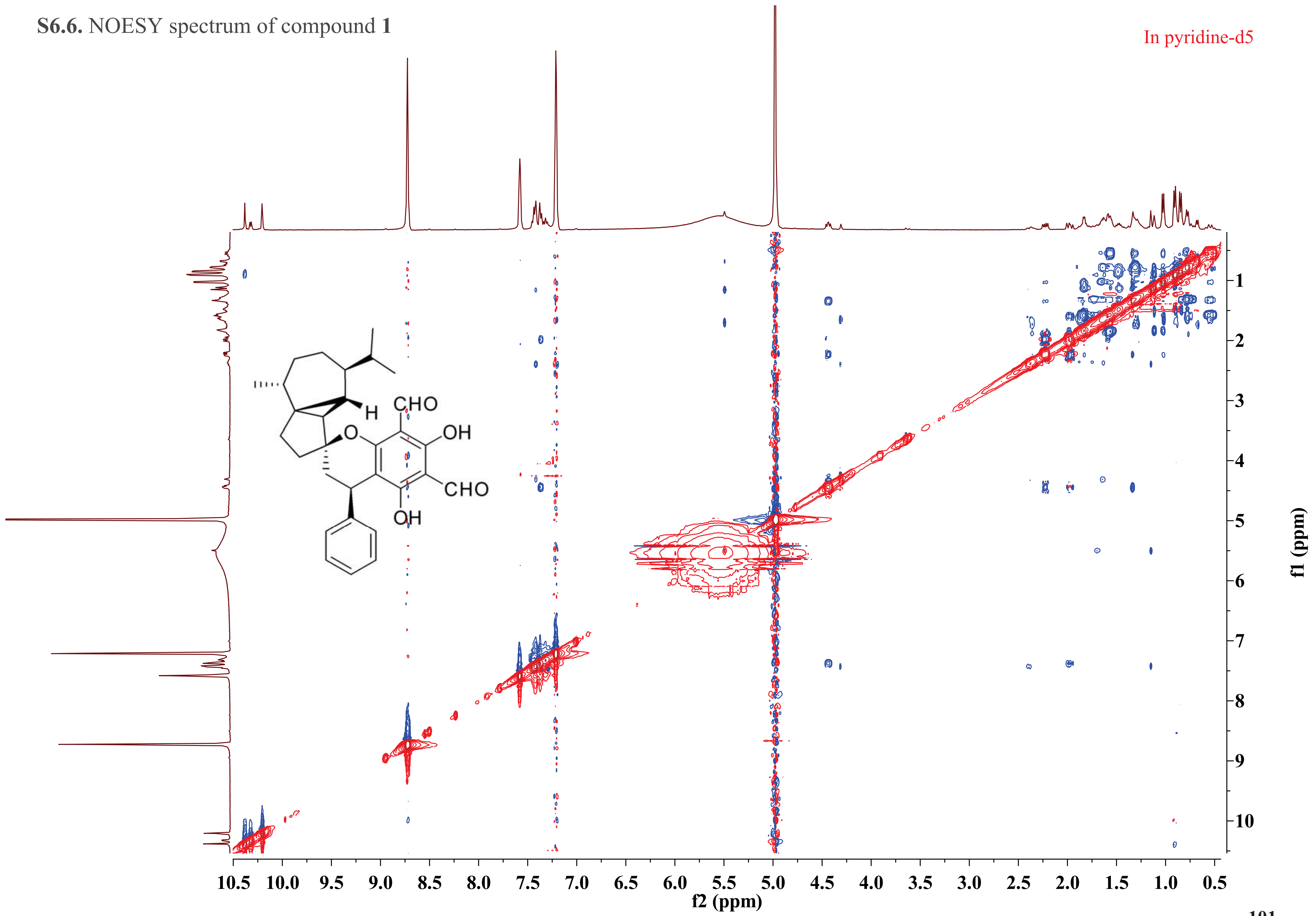

S6.7. ^{1}H NMR spectrum of compound **2**

In pyridine-d5

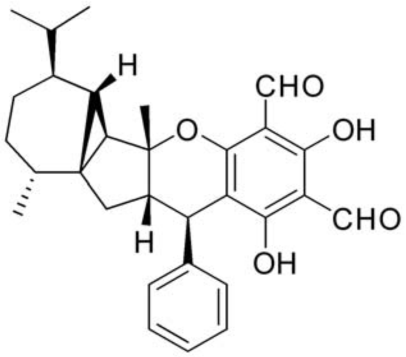

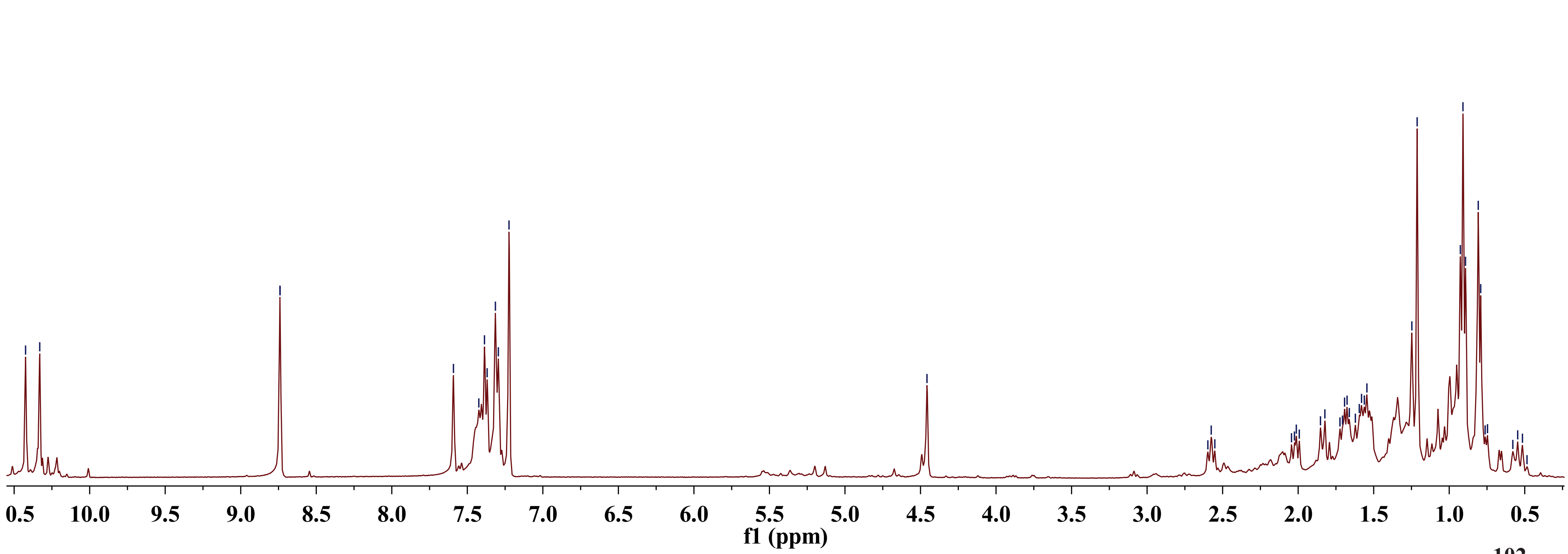

S6.8. DEPT spectra of compound **2**

In pyridine-d5

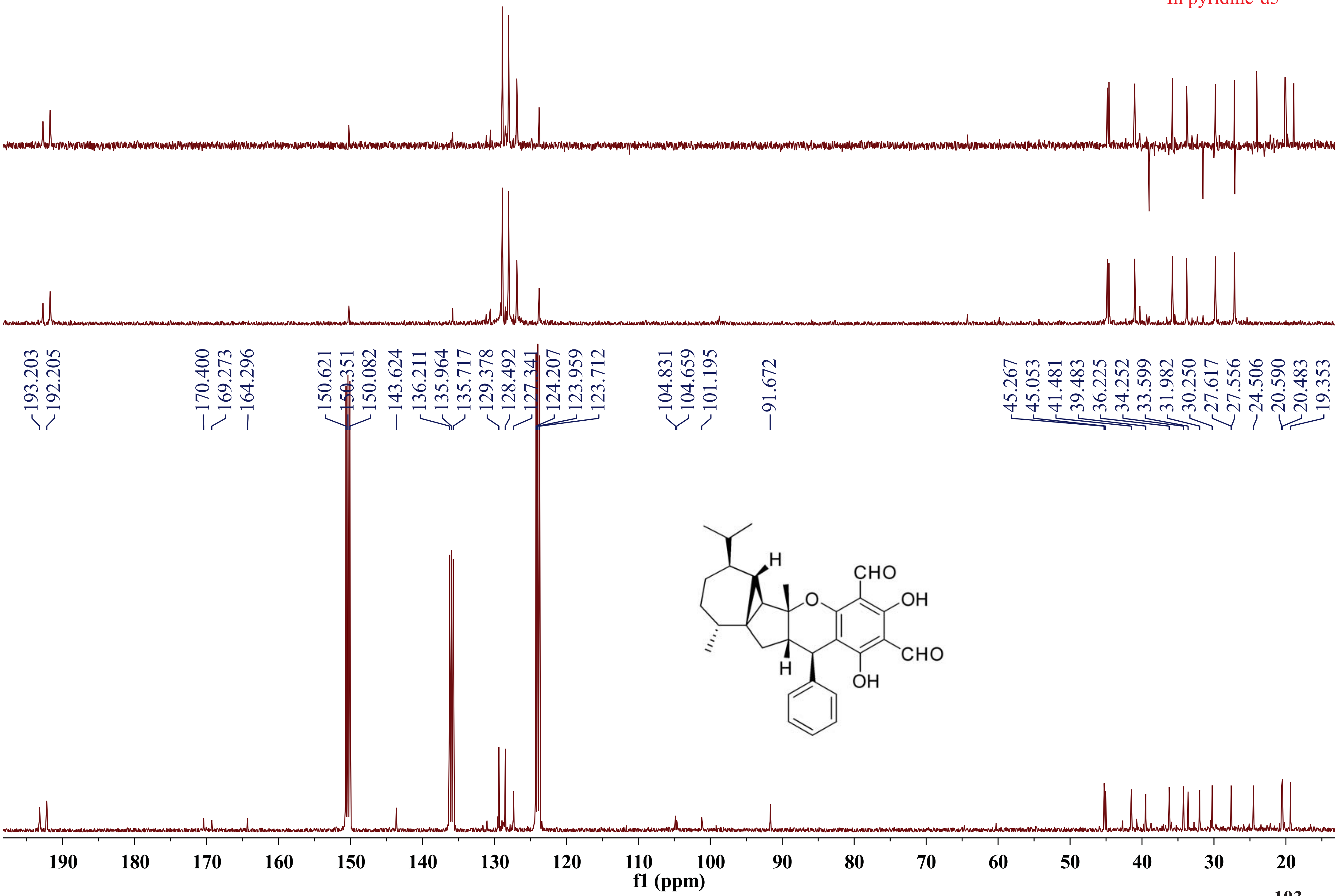

S6.9. HSQC spectrum of compound **2**

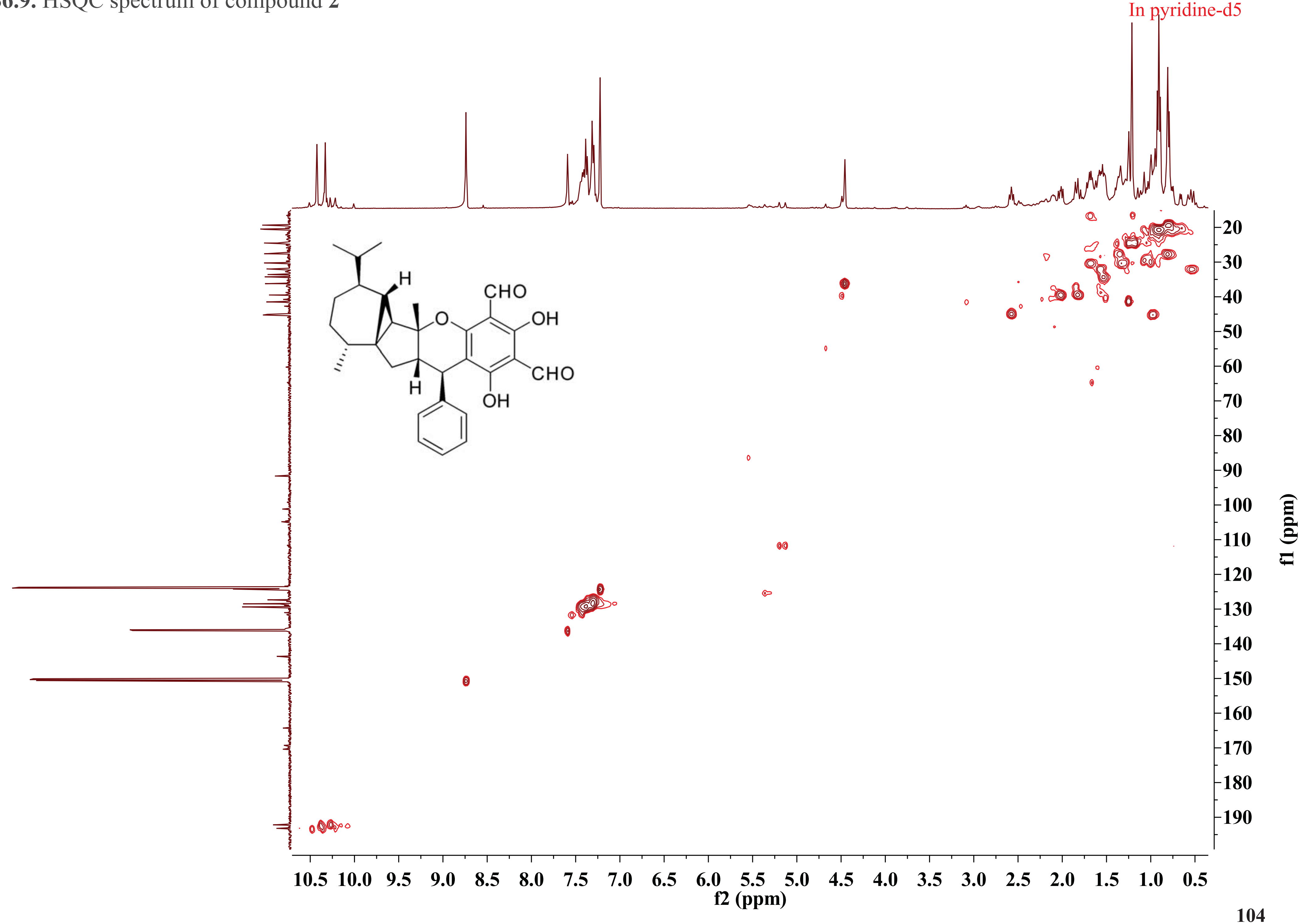

S6.10. ^{1}H-^{1}H COSY spectrum of compound **2**

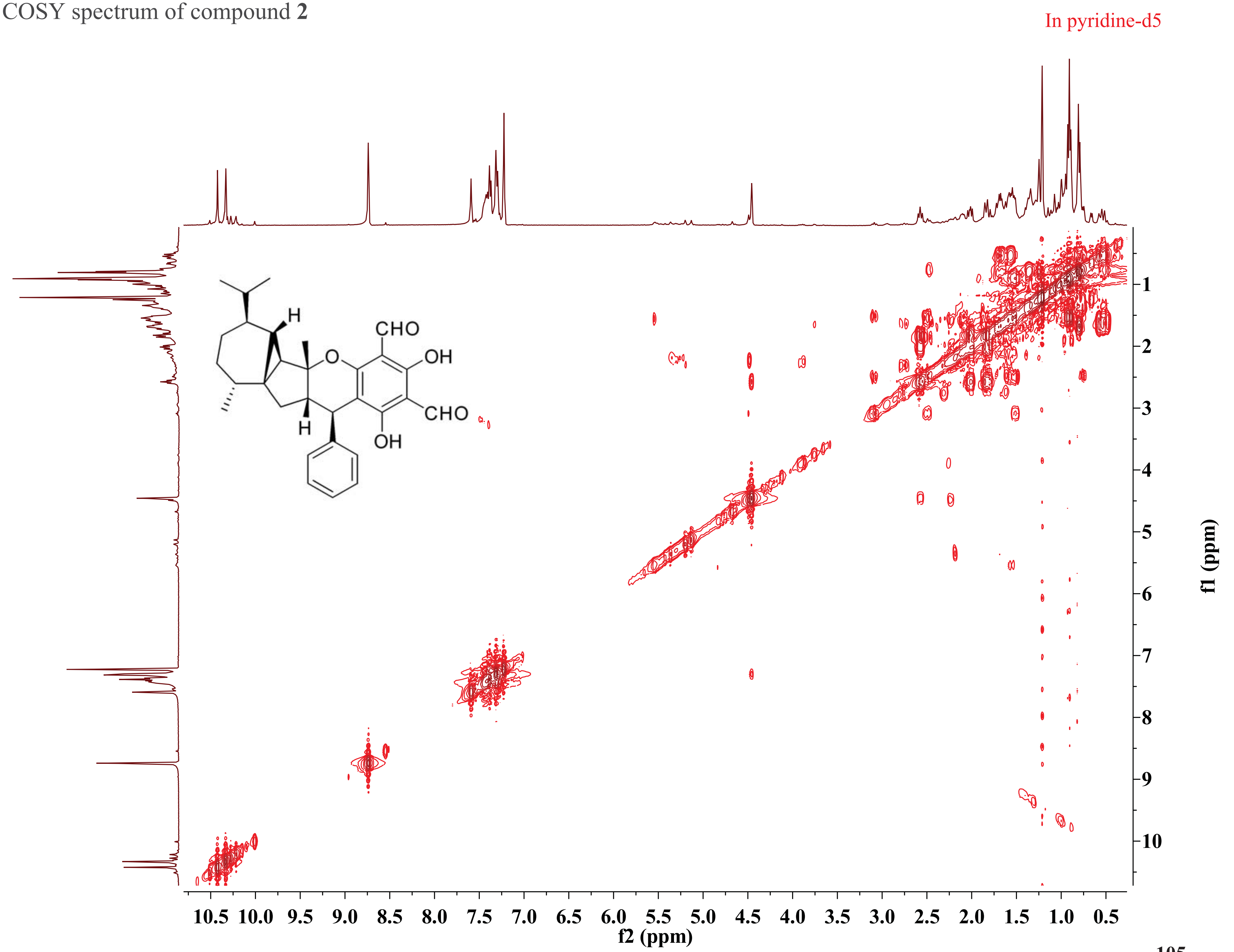

S6.10. ^{1}H-^{1}H COSY spectrum of compound **2**

In pyridine-d5

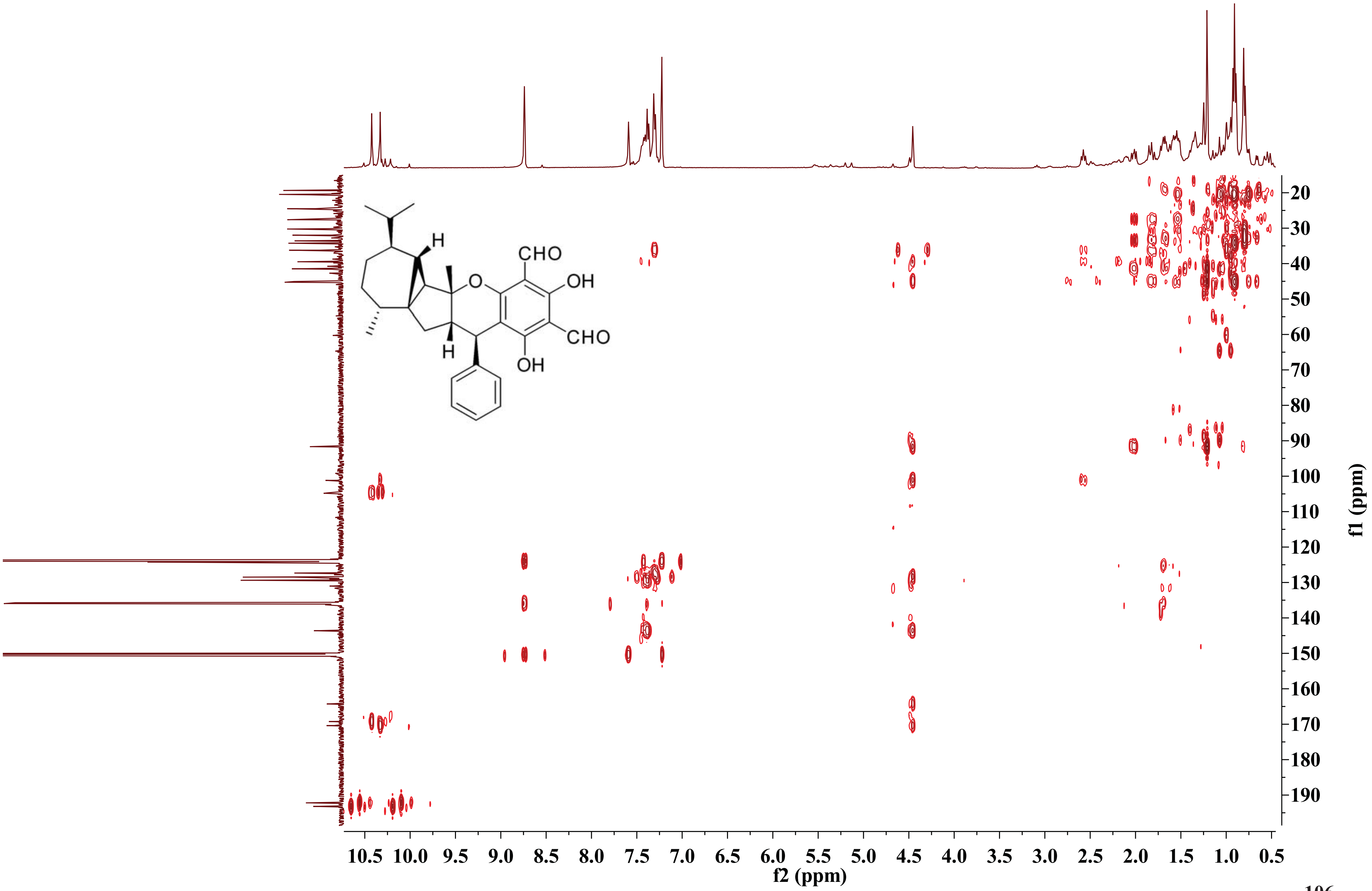

S6.12. NOESY spectrum of compound **2**

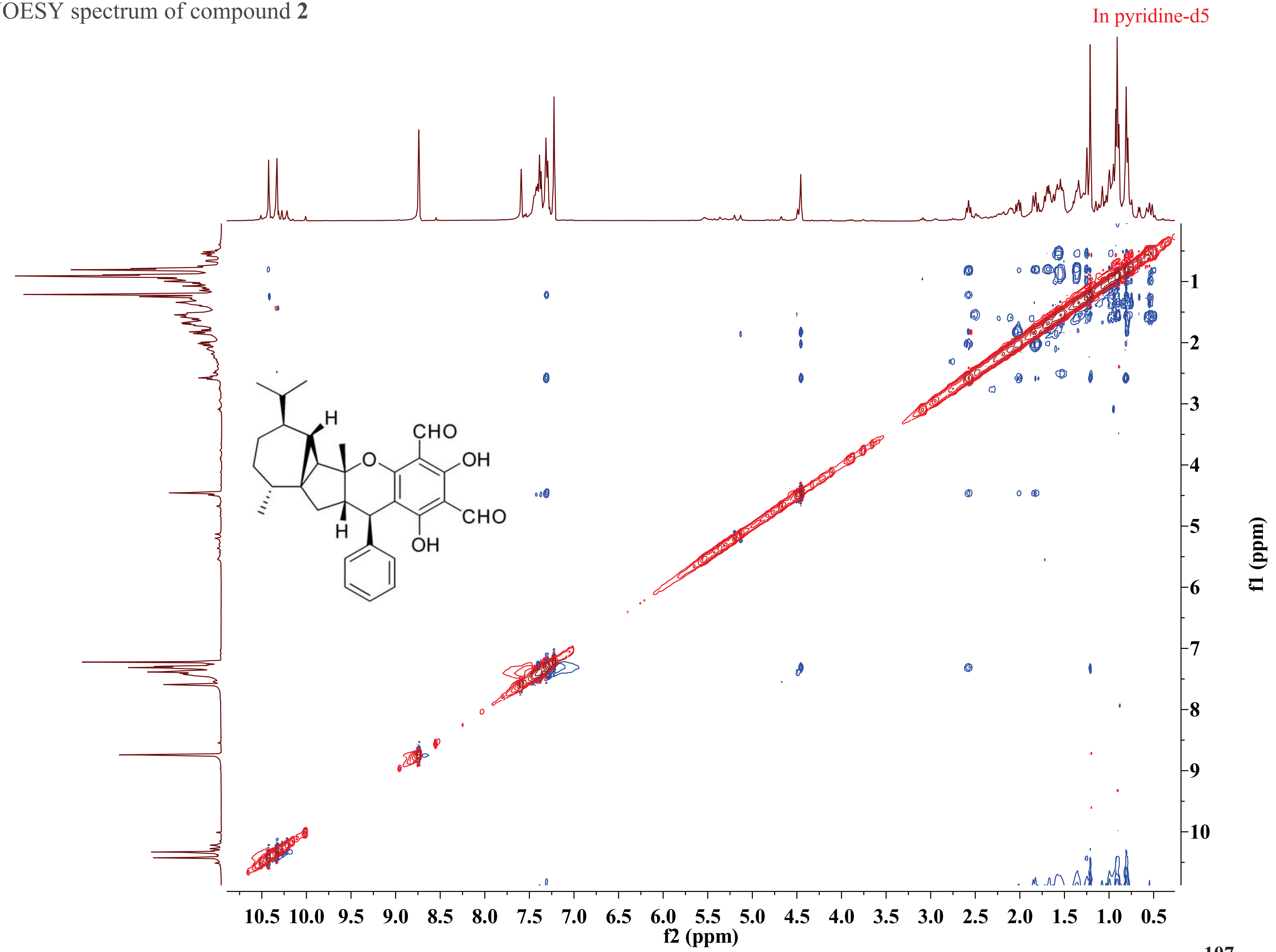

S6.13. ^{1}H NMR spectrum of compound **4**

In pyridine-d5

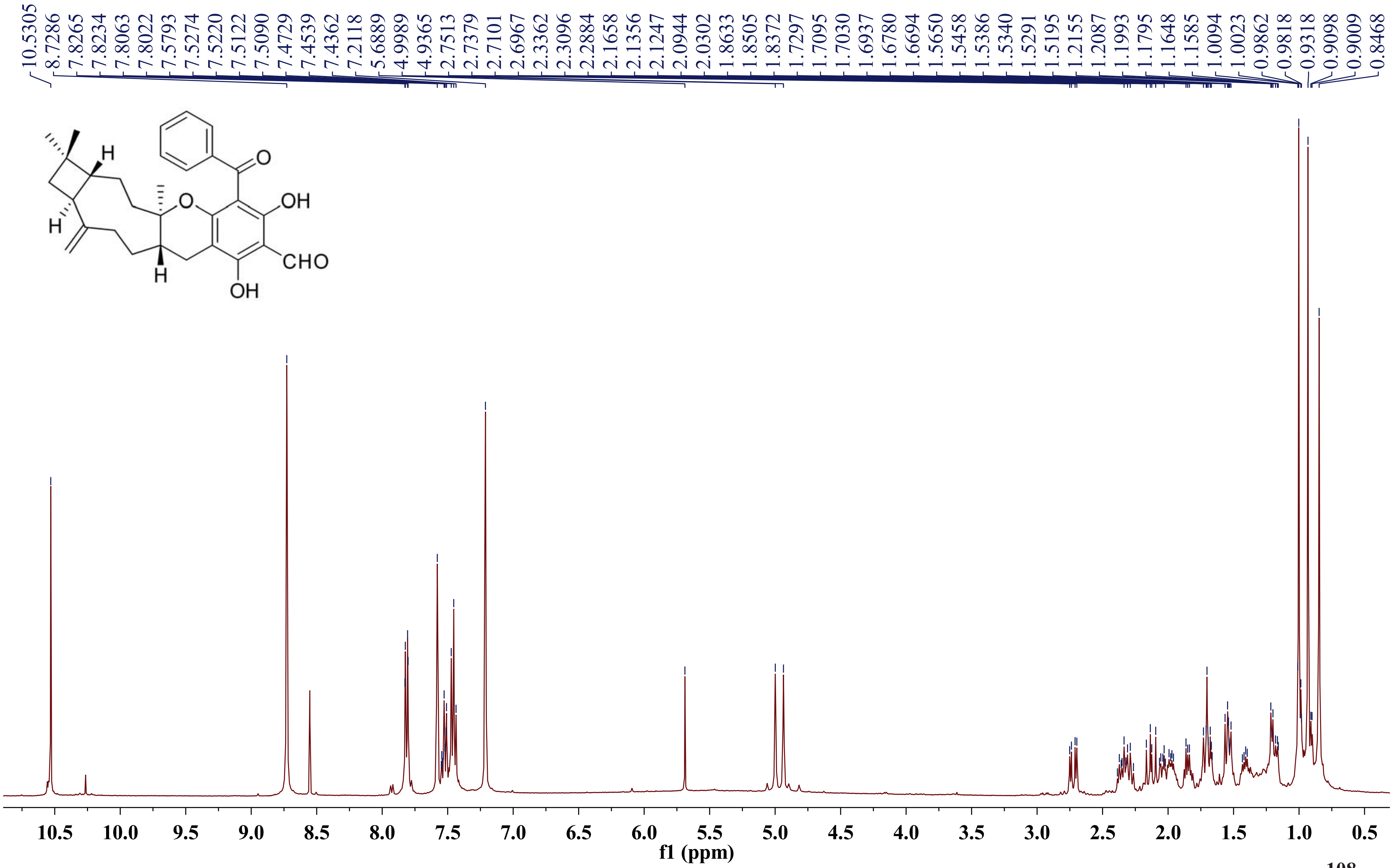

S6.14. DEPT spectra of compound **4**

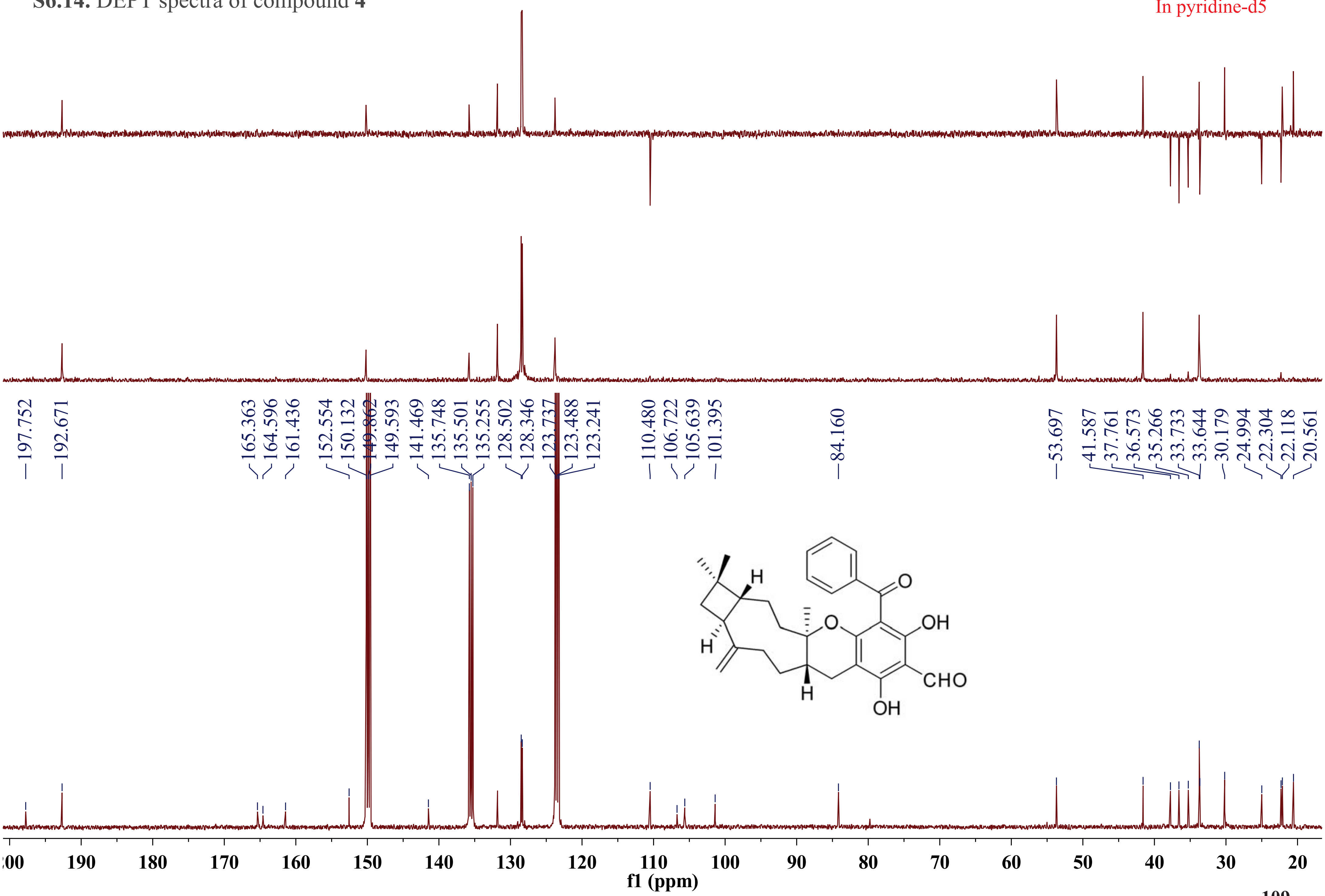

S6.15. HSQC spectrum of compound **4**

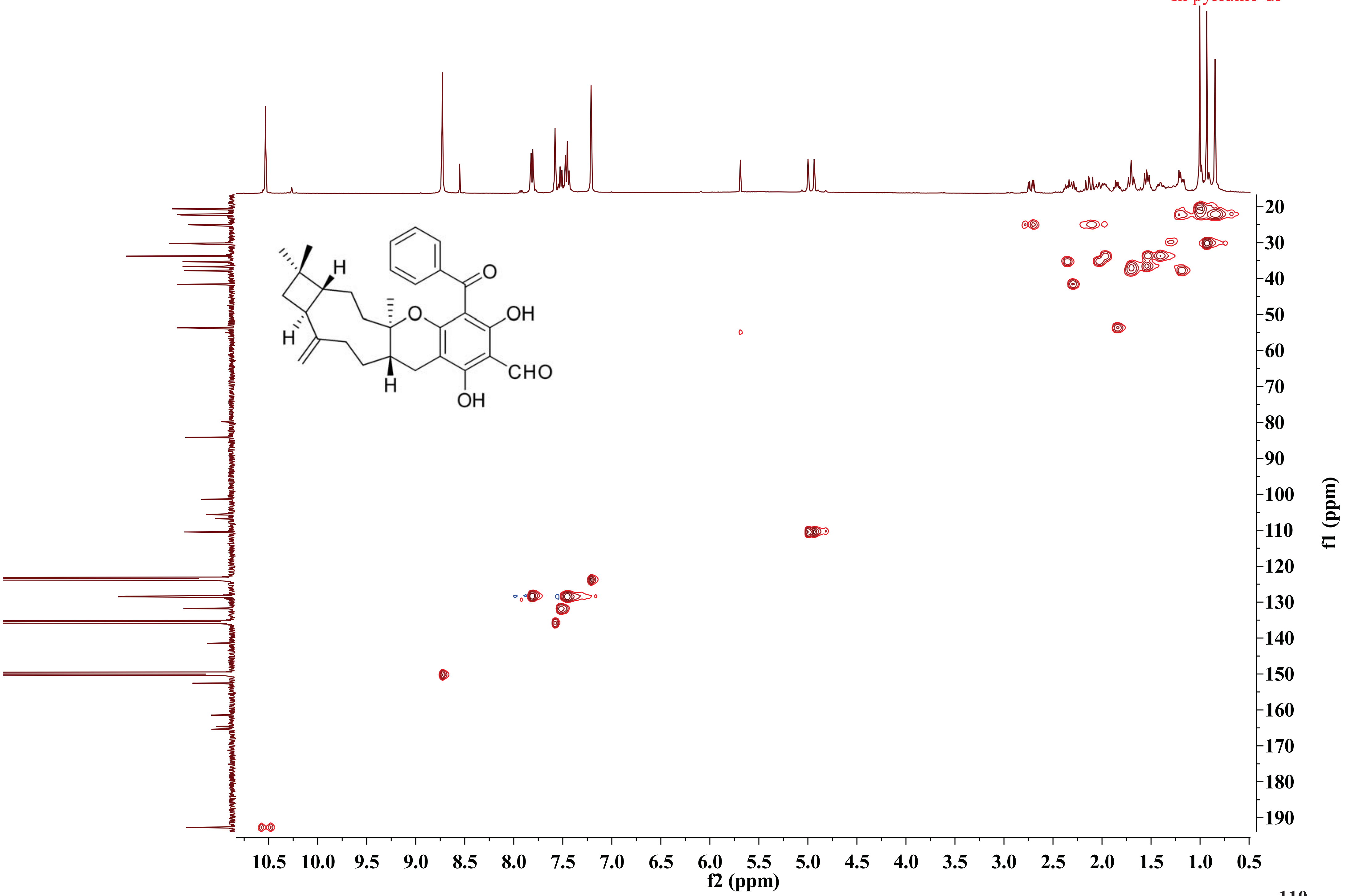

S6.16. ^{1}H-^{1}H COSY spectrum of compound **4**

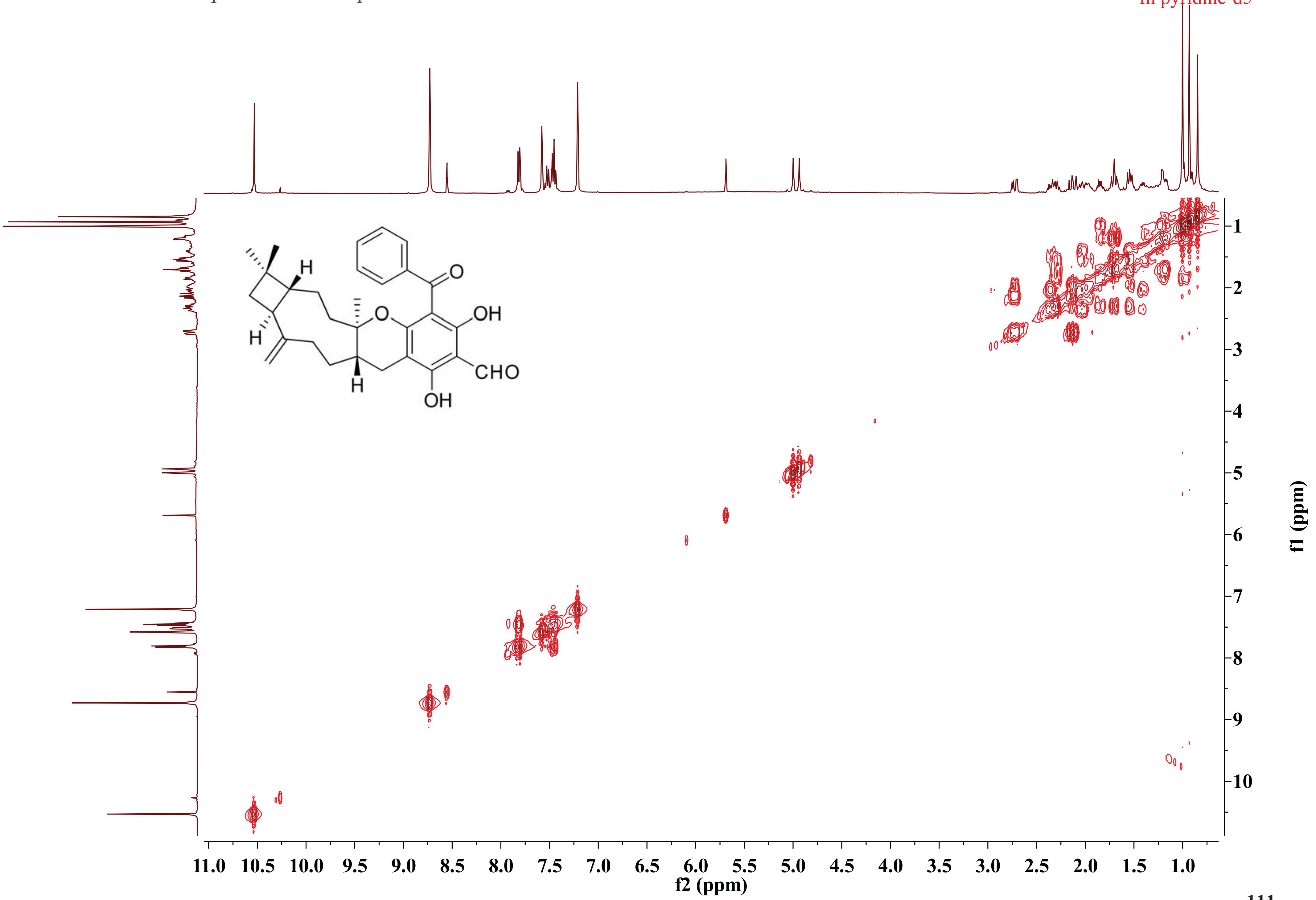

S6.17 HMBC spectrum of compound **4**

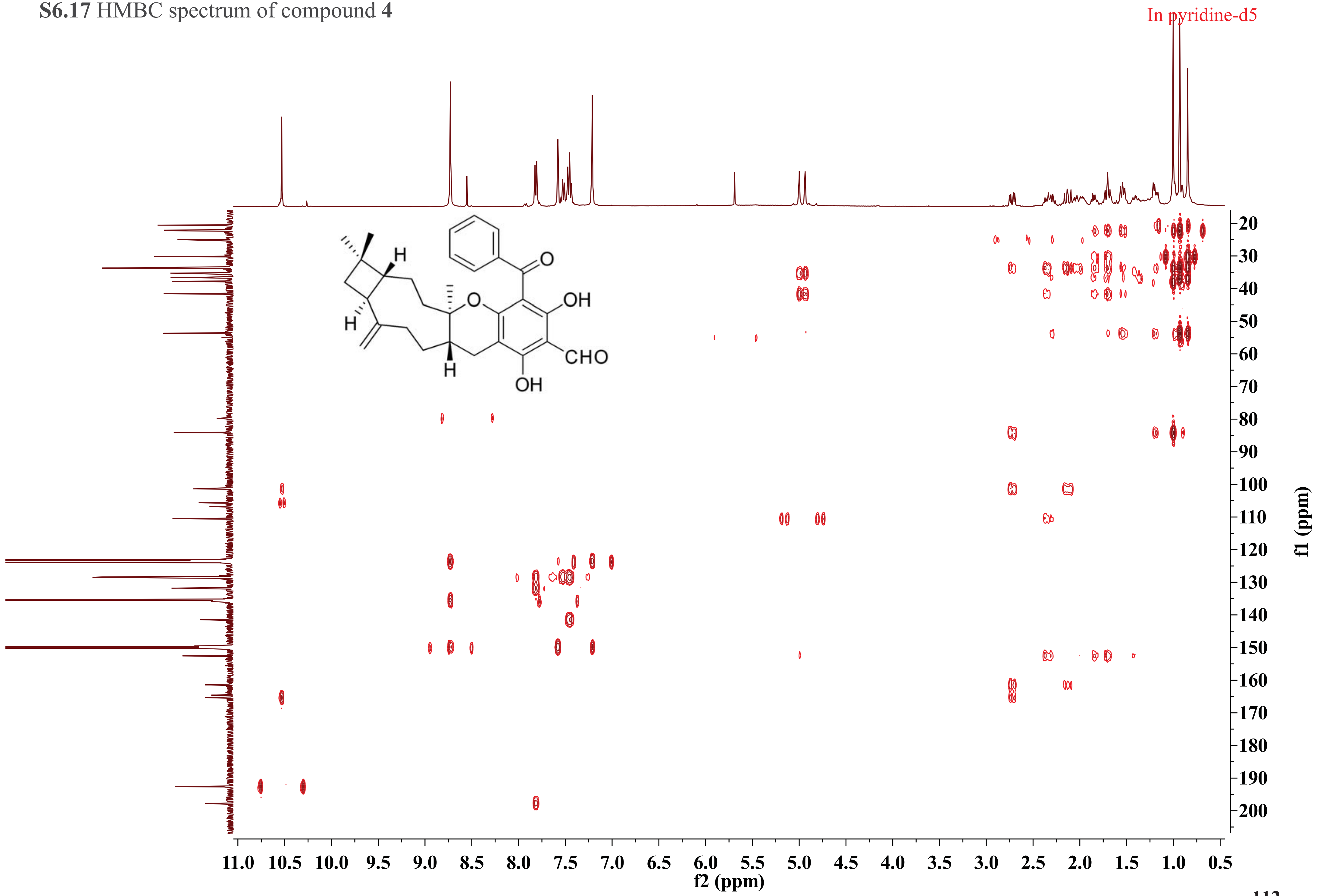

S6.18. NOESY spectrum of compound **4**

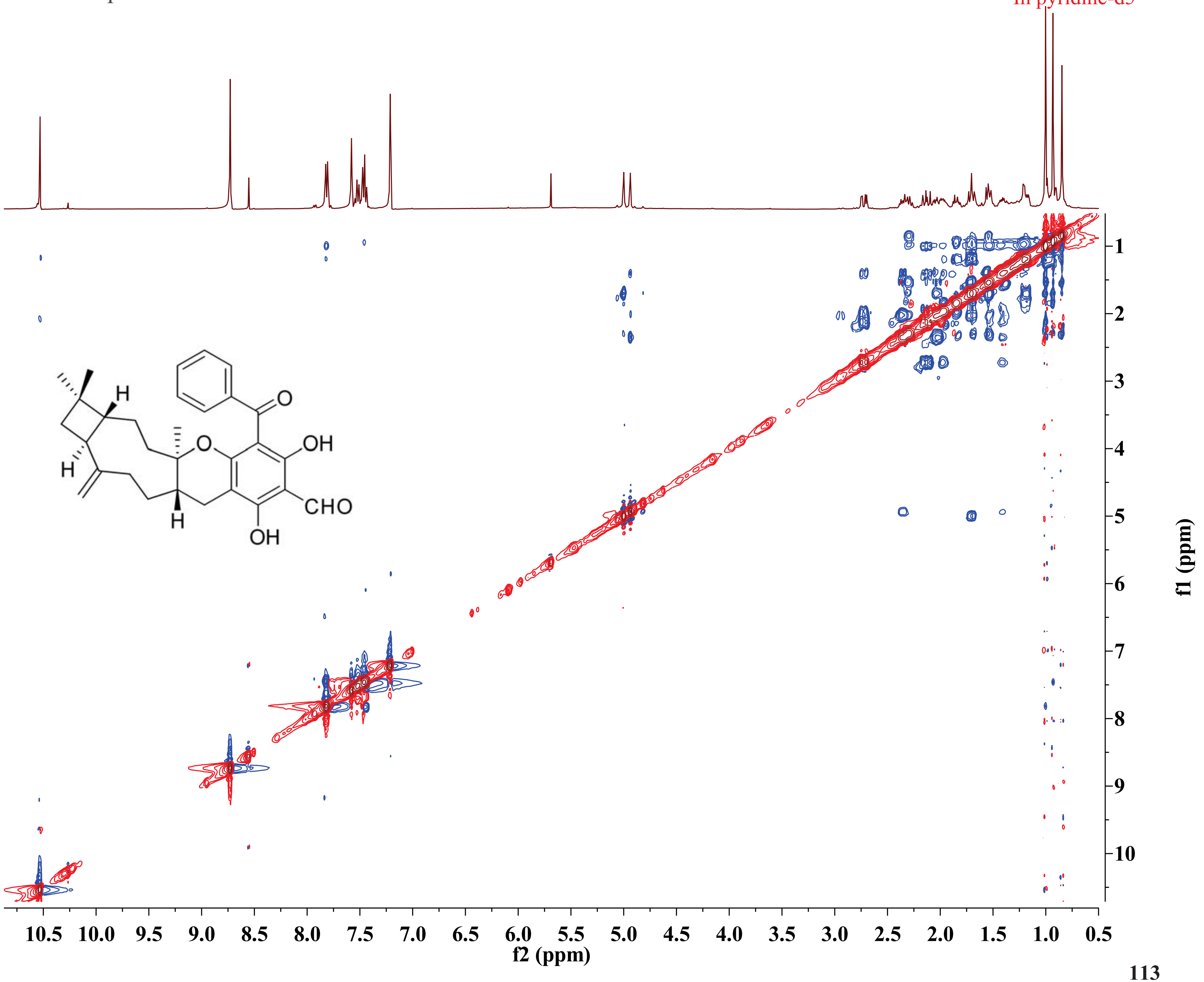

S7.1. ESIMS spectrum of compound **1**

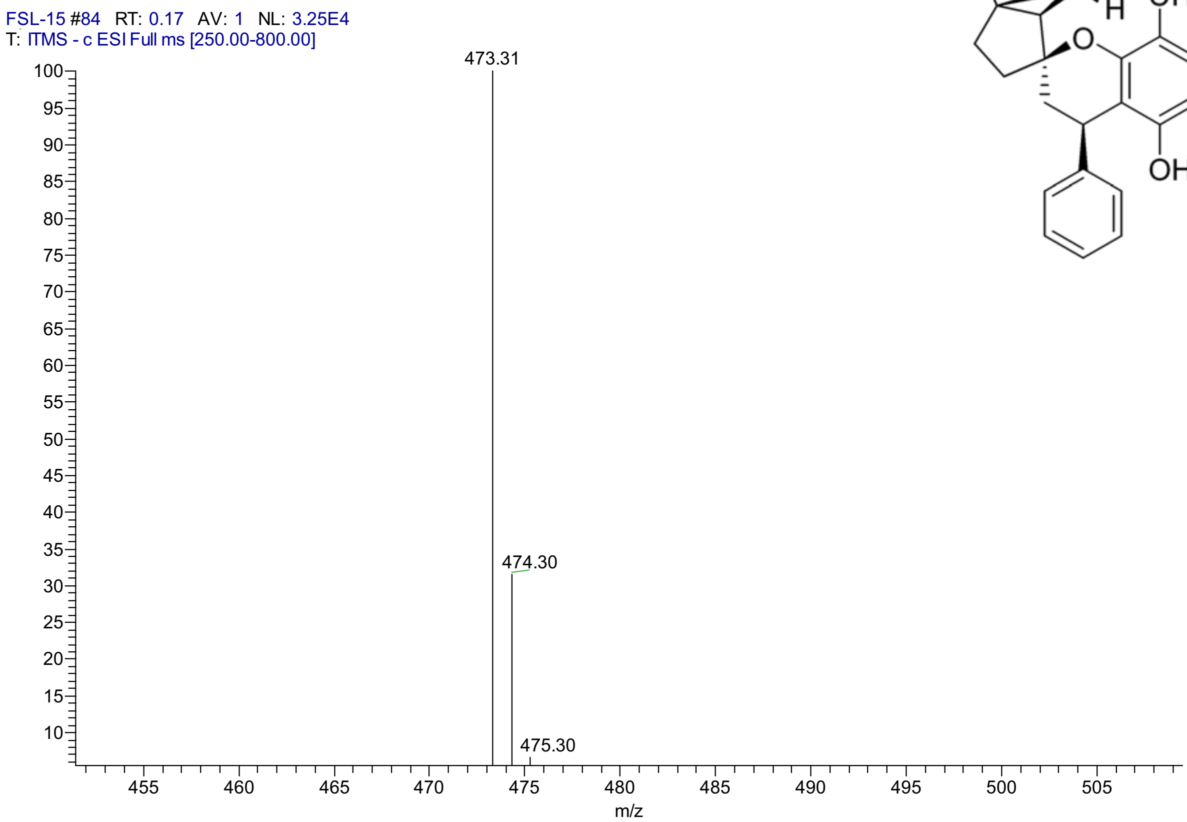

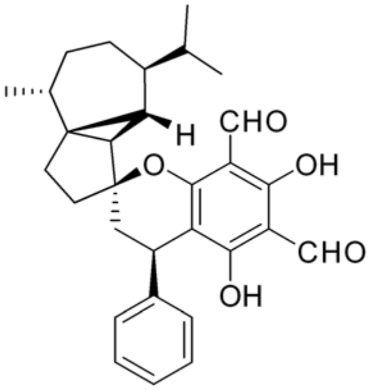

S7.2. HRESIMS spectrum of compound **1**

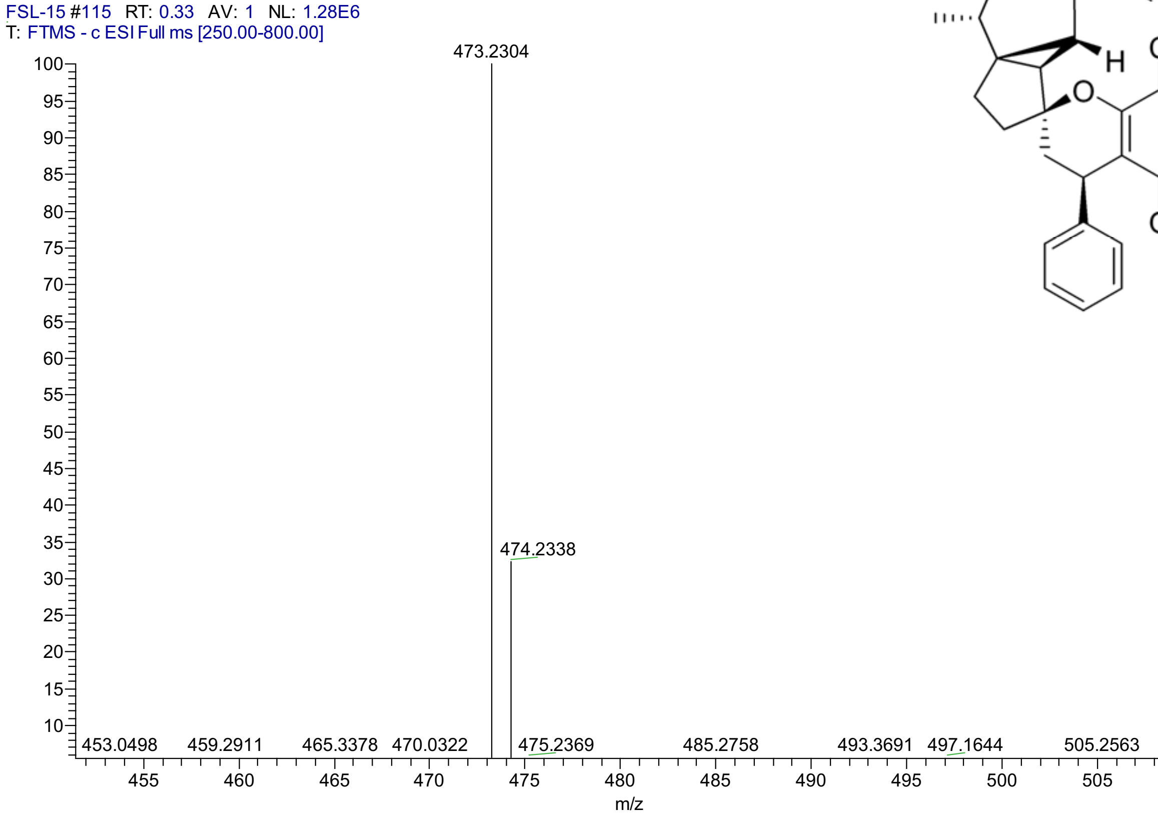

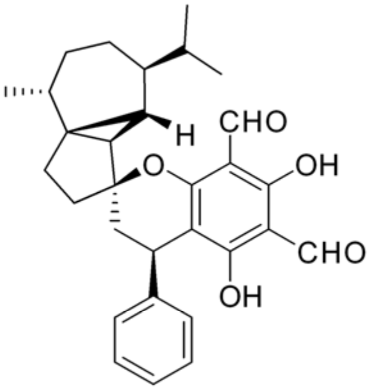

S7.3. ESIMS spectrum of compound **2**

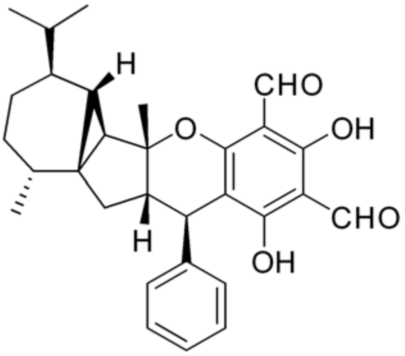

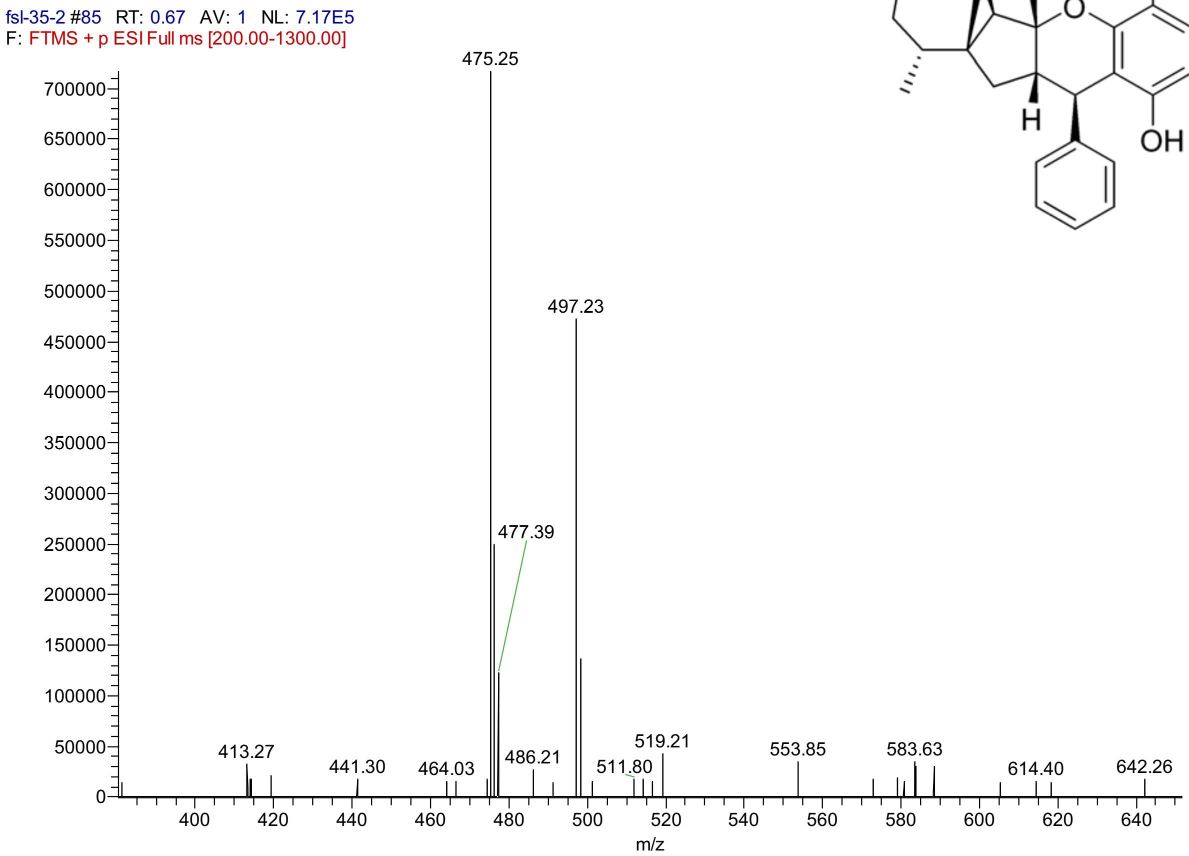

S7.4. HRESIMS spectrum of compound **2**

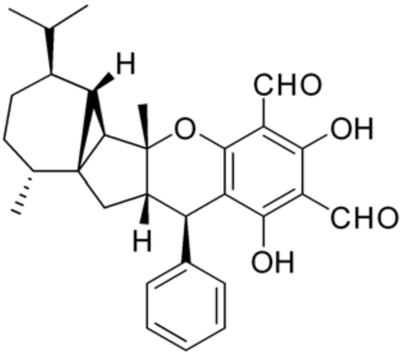

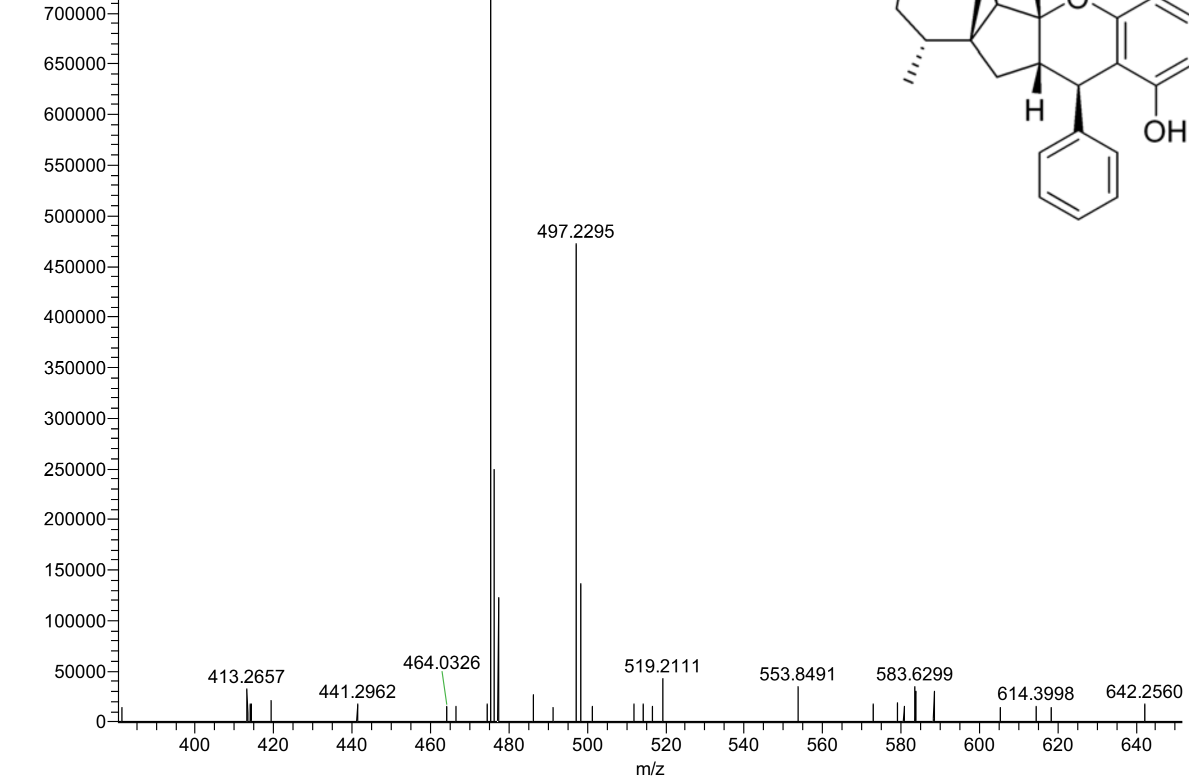

S7.5. ESIMS spectrum of compound **3**

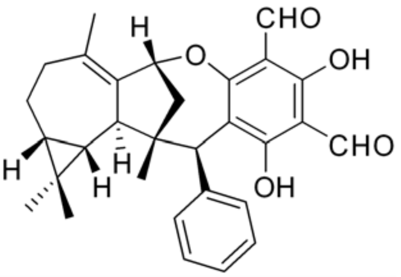

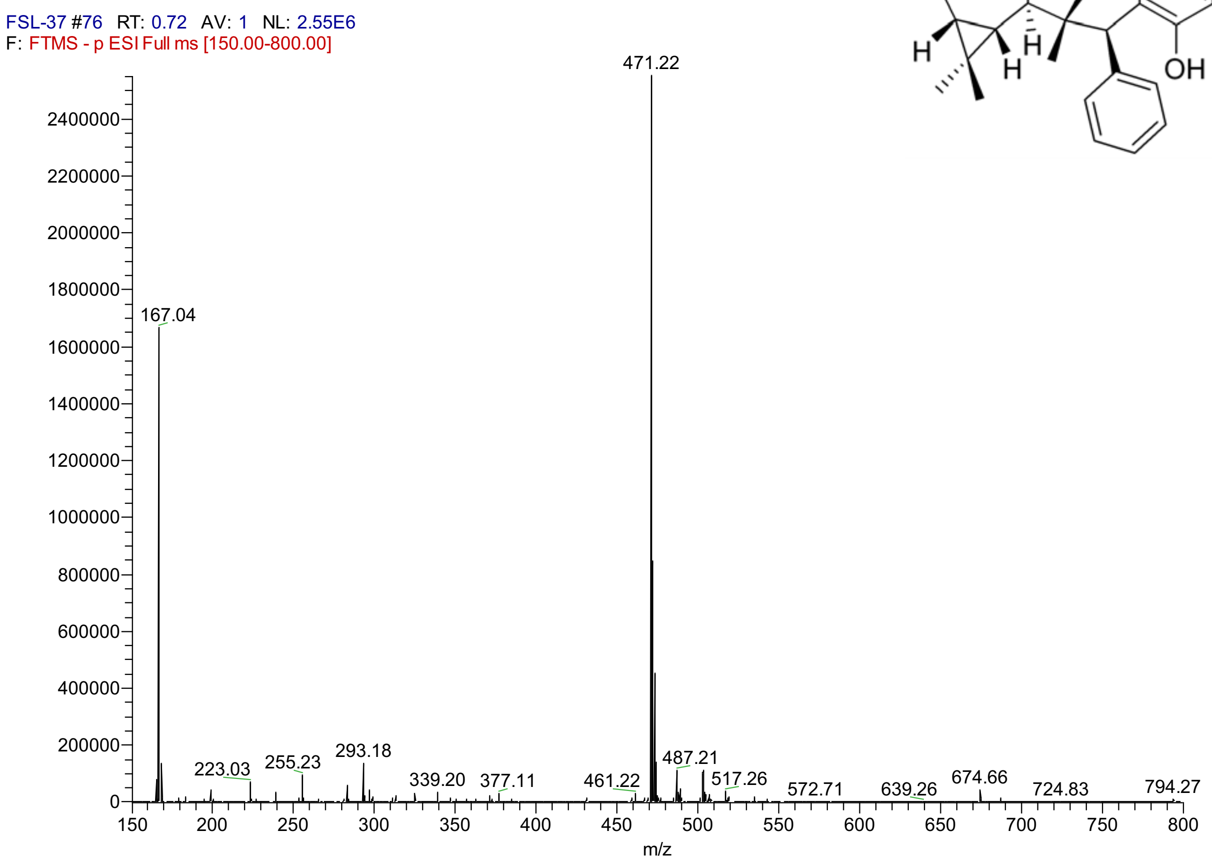

S7.6. HRESIMS spectrum of compound **3**

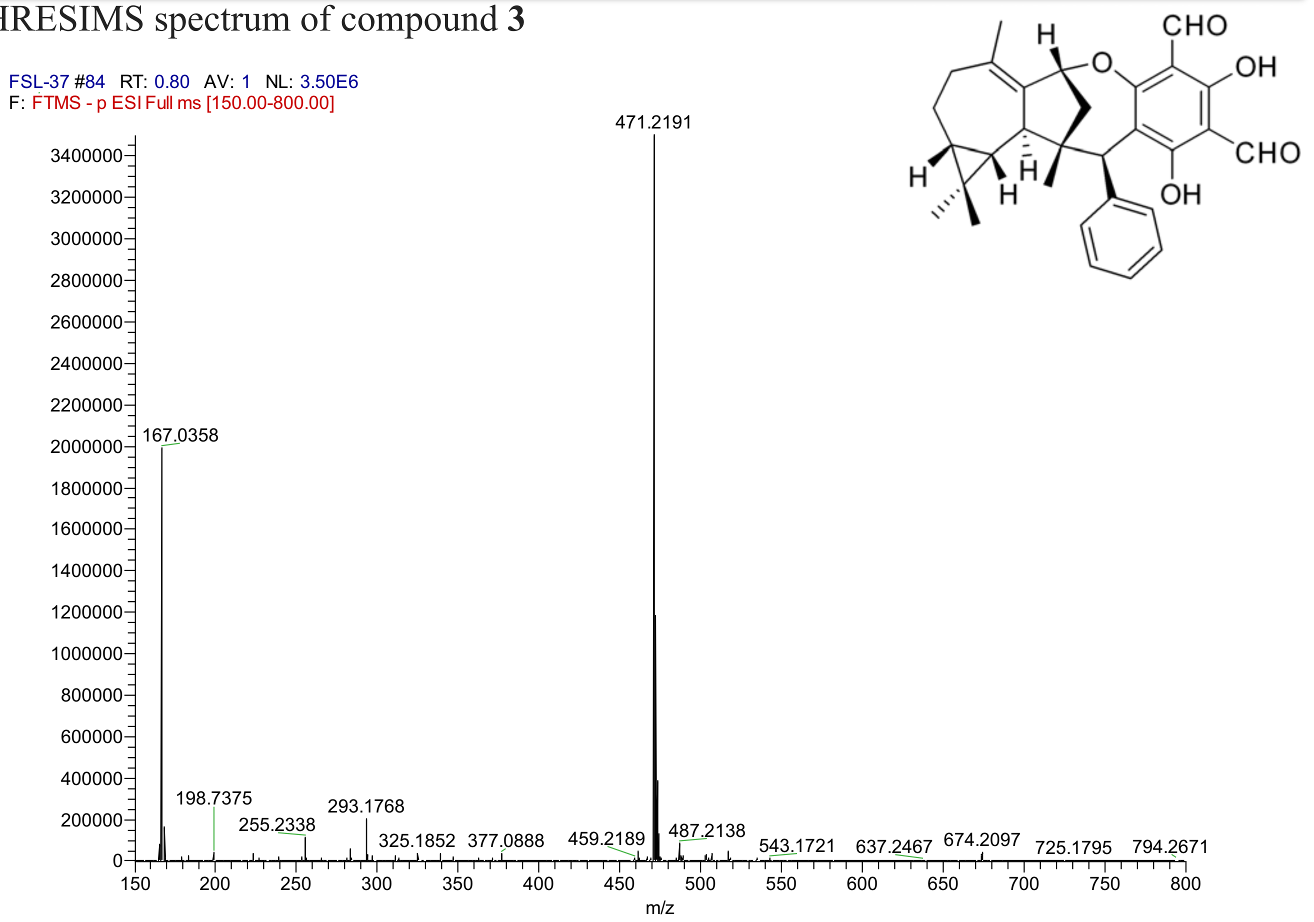

S7.7. ESIMS spectrum of compound **4**

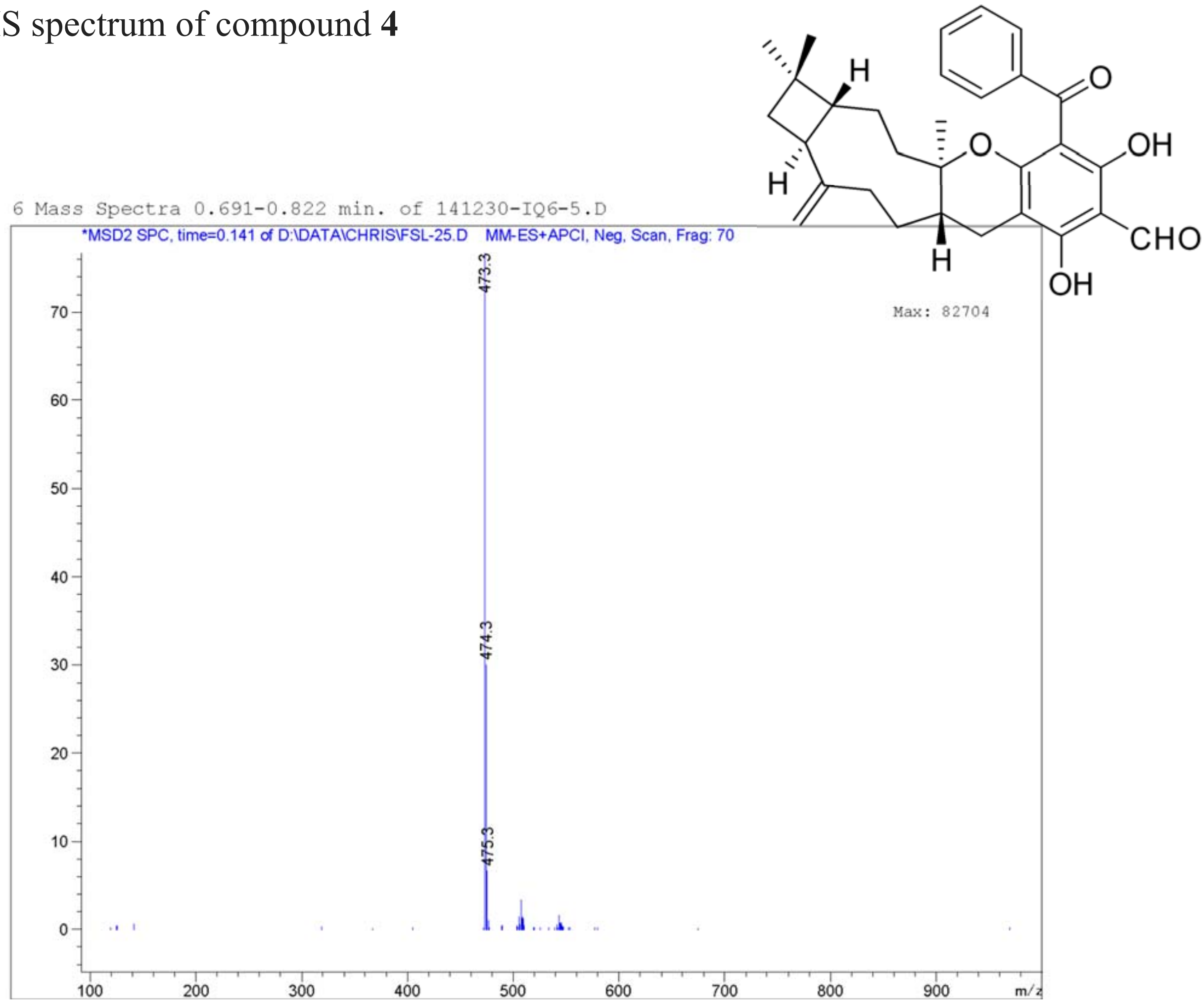

S7.8. HRESIMS spectrum of compound **4**

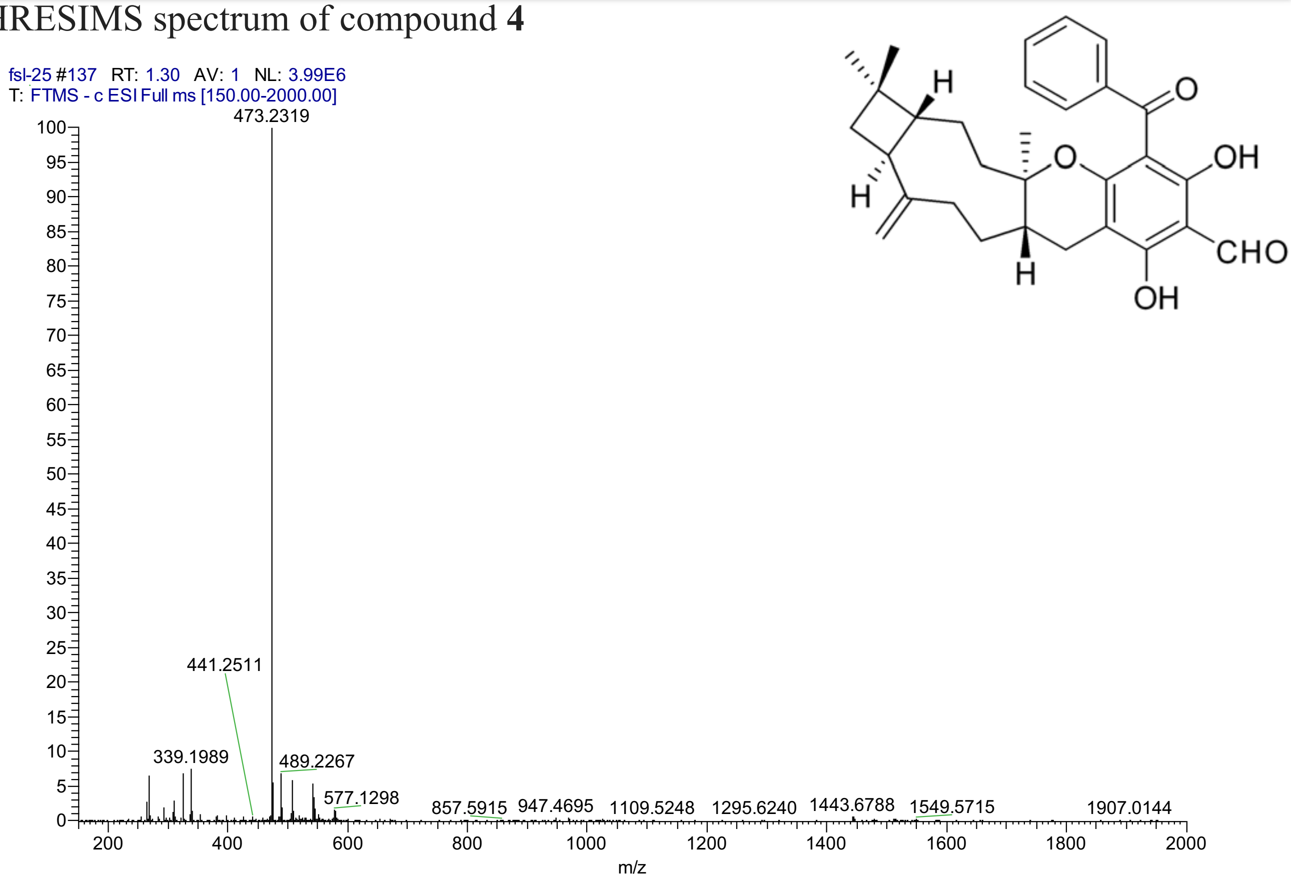

S7.9. ESIMS spectrum of compound **5**

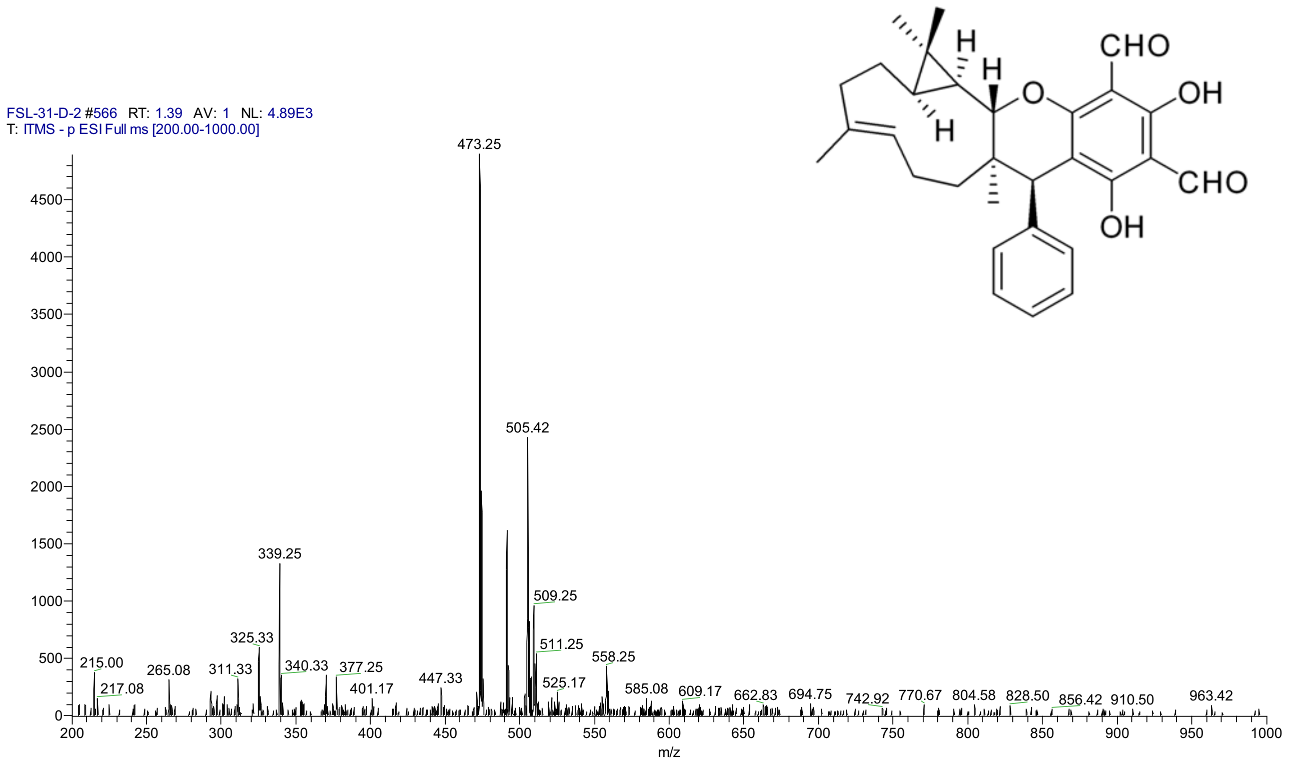

S7.10. HRESIMS spectrum of compound **5**

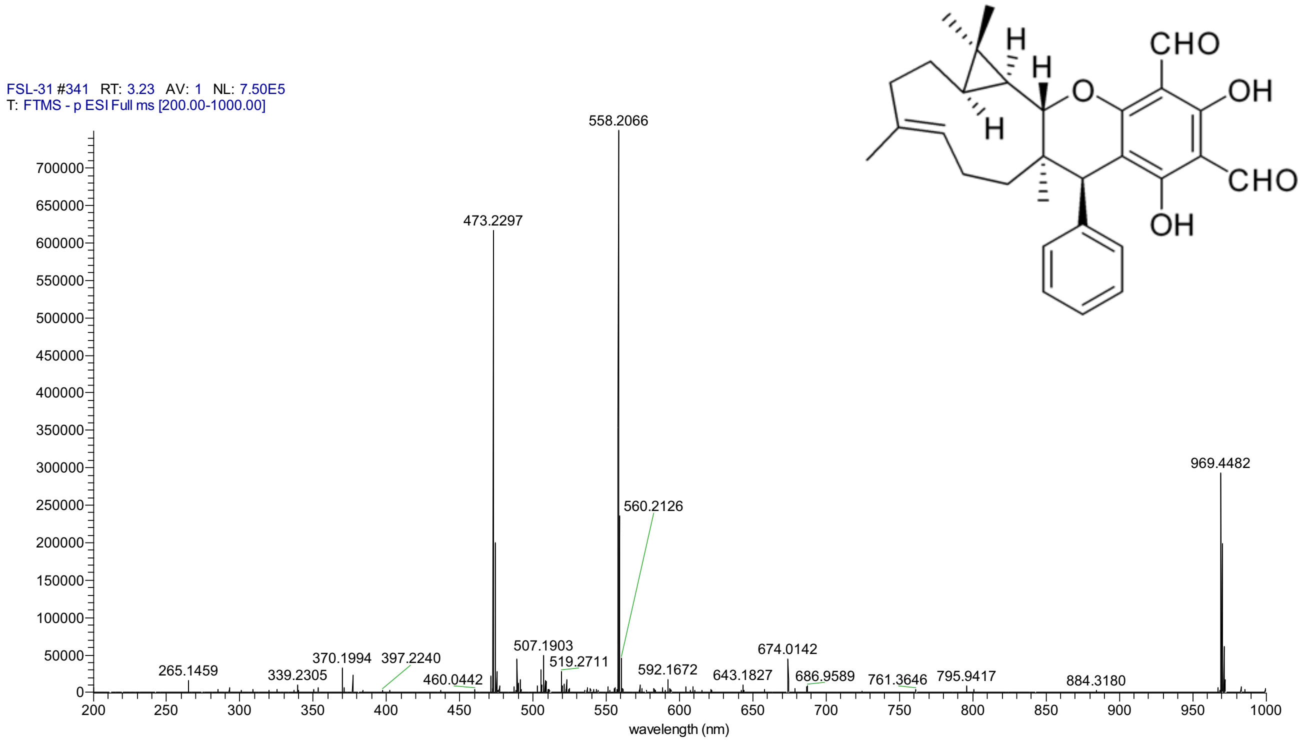

S7.11. ESIMS spectrum of compound **6**

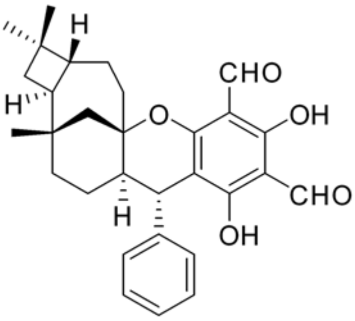

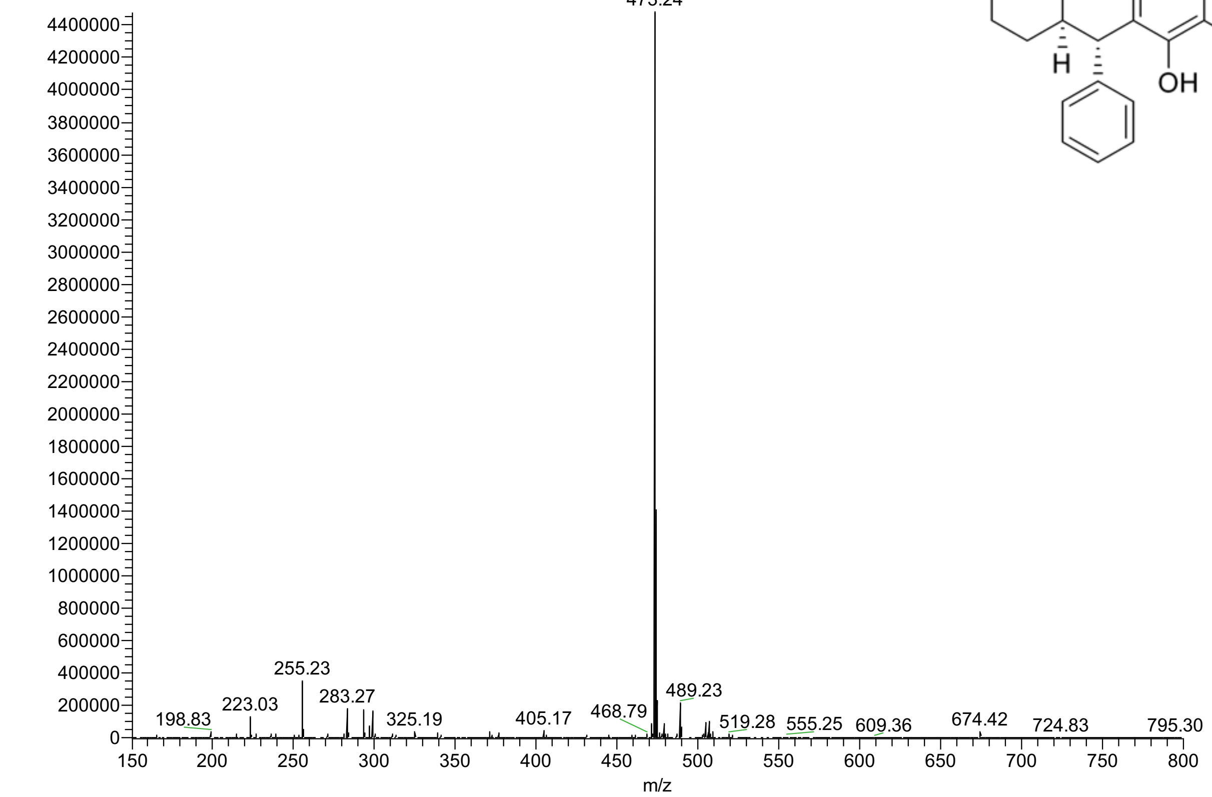

S7.12. HRESIMS spectrum of compound **6**

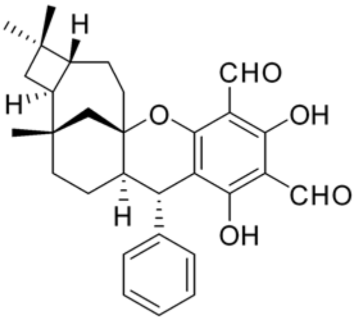

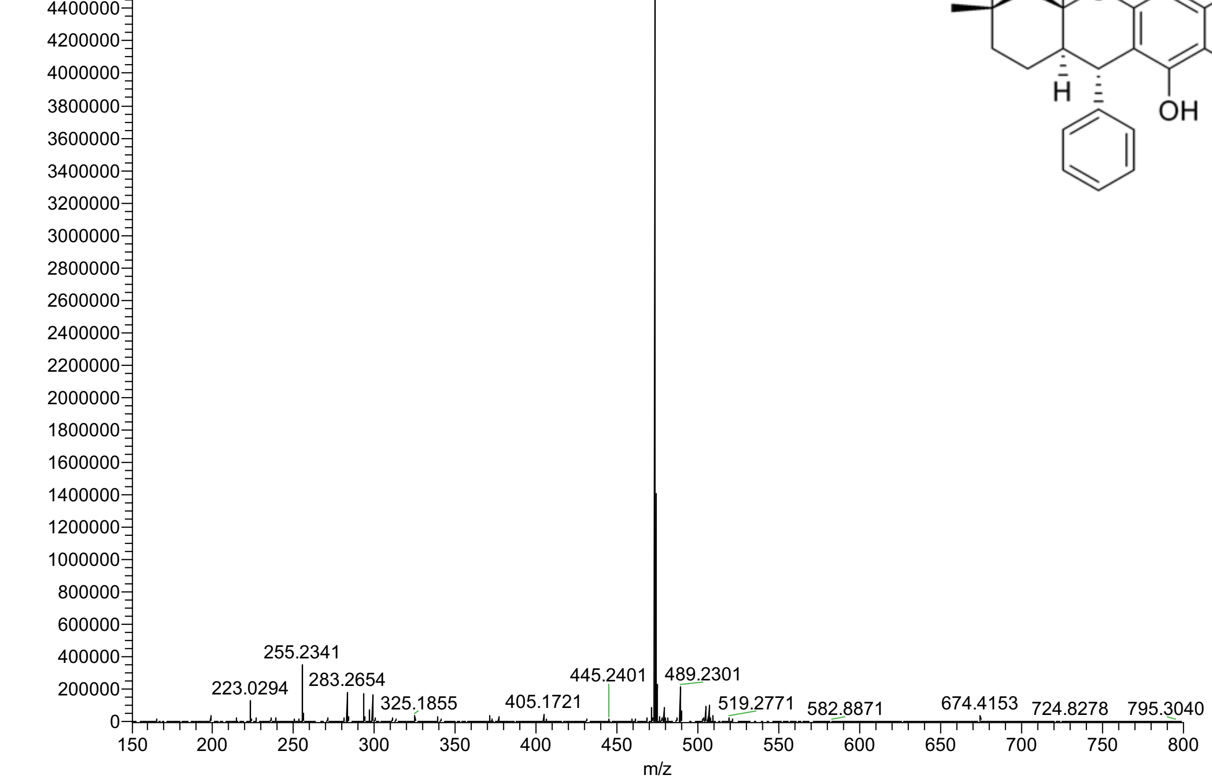

S7.13. ESIMS spectrum of compound **7**

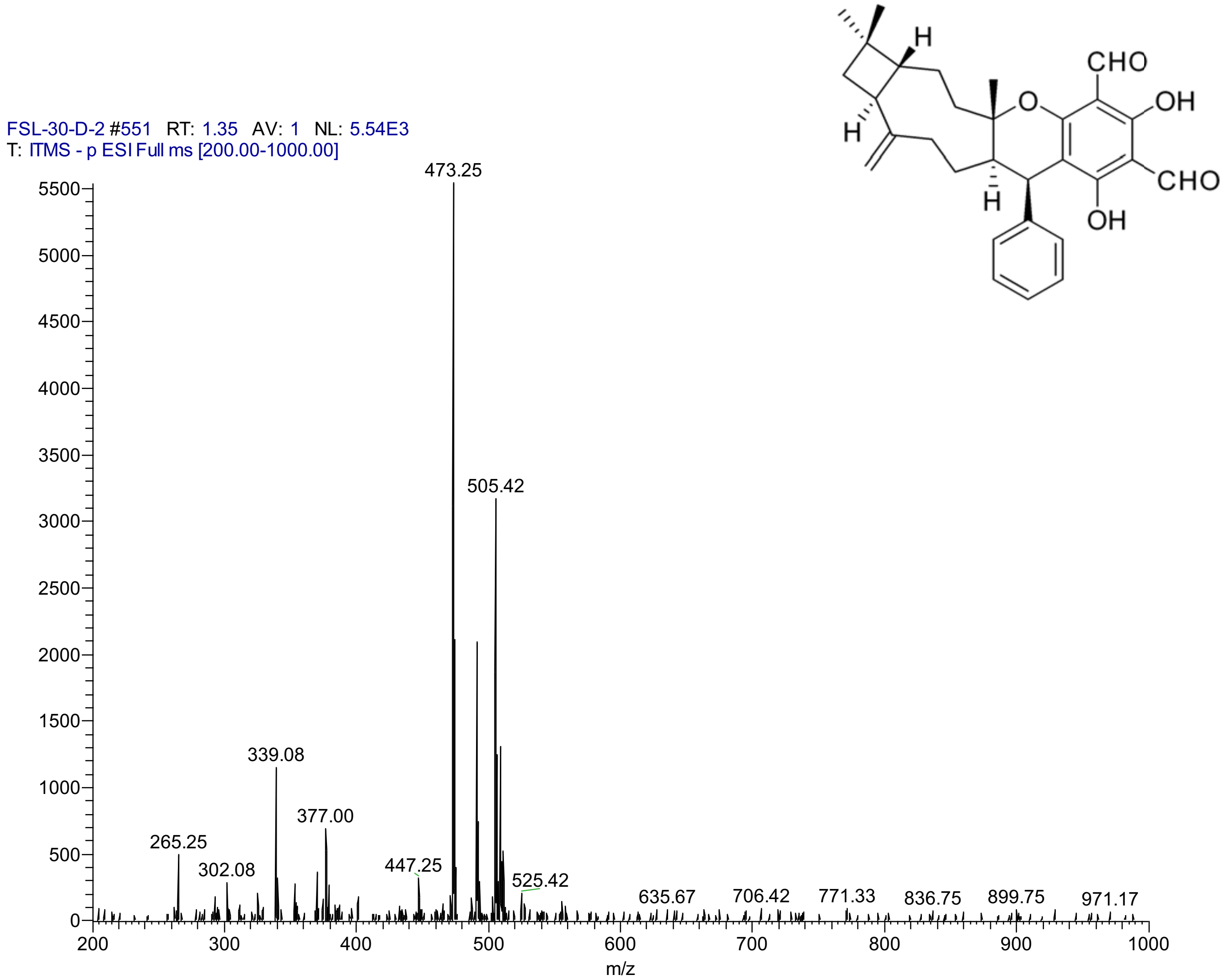

S7.14. HRESIMS spectrum of compound **7**

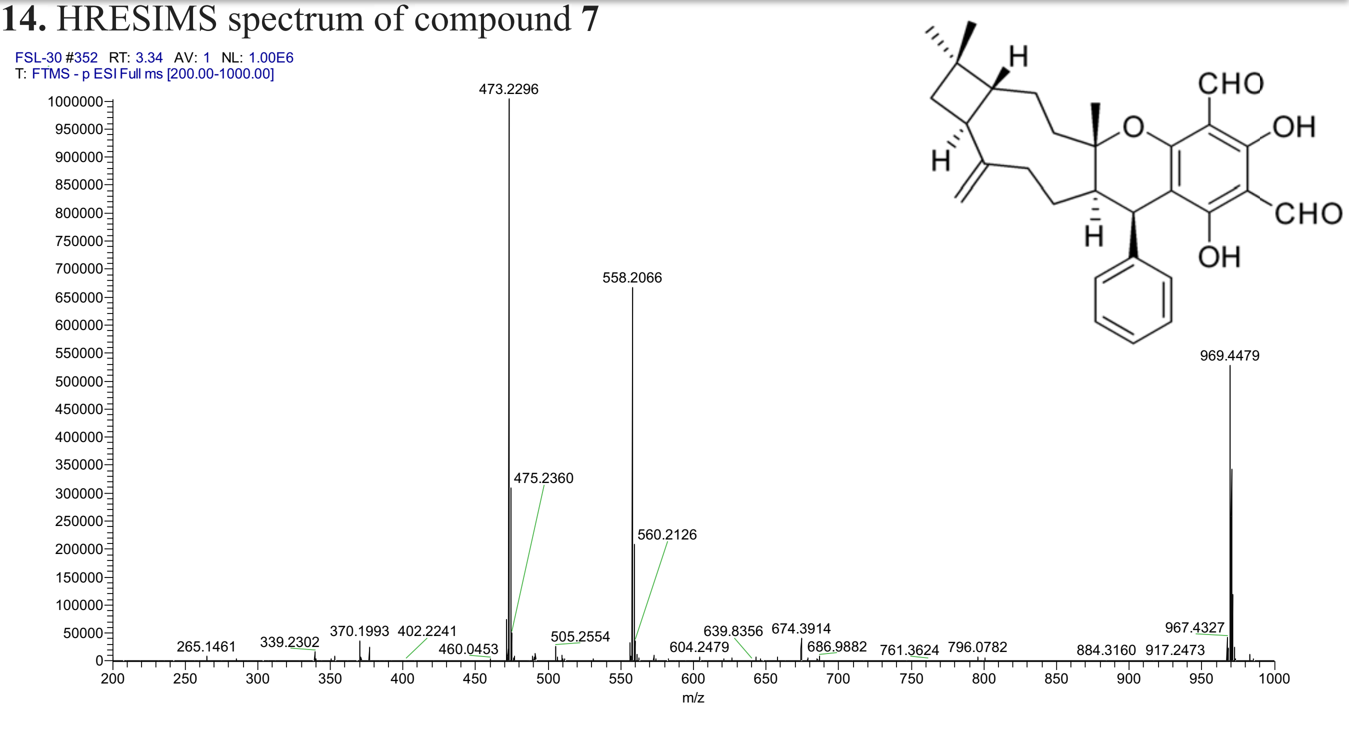

S7.15. ESIMS spectrum of compound **8**

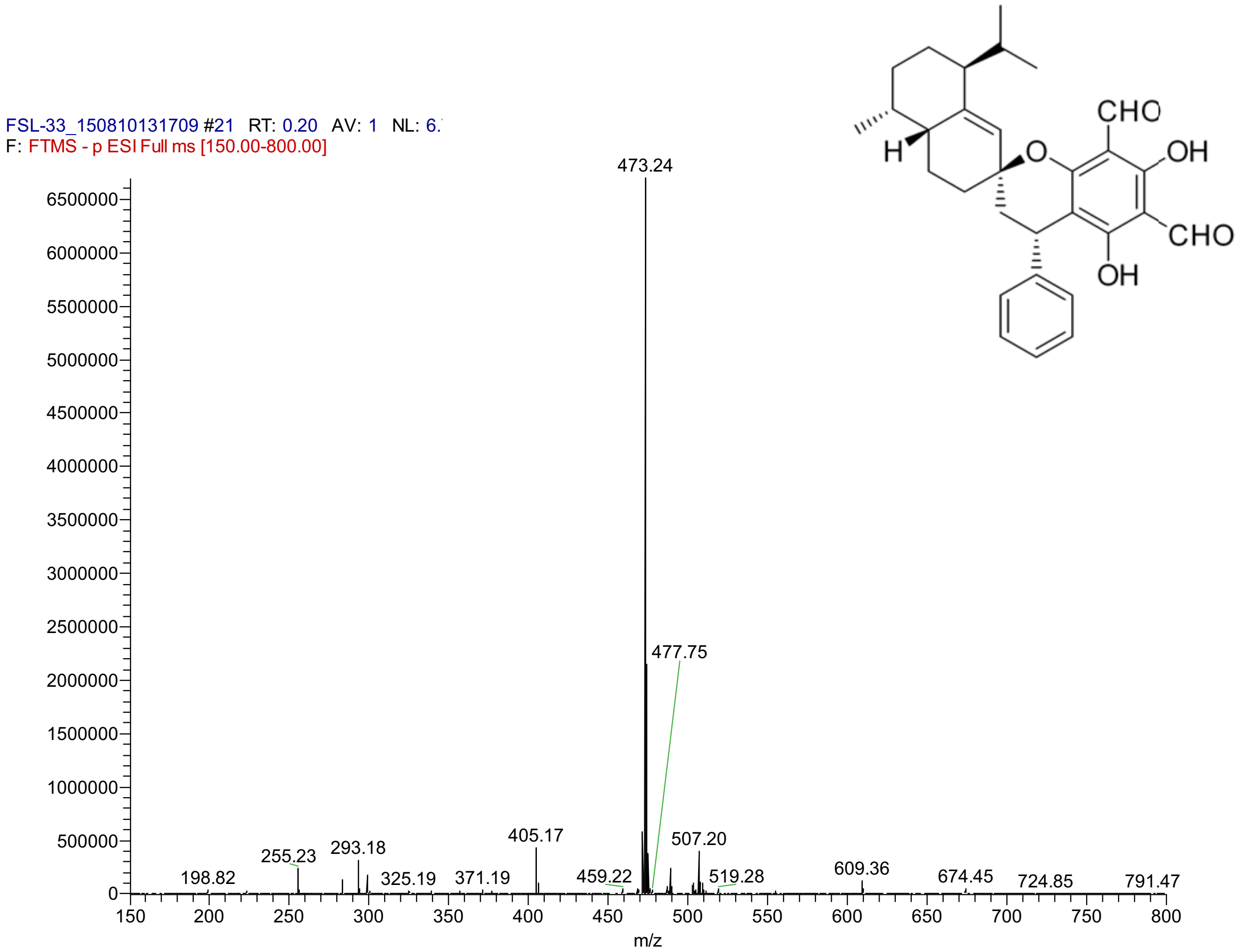

S7.16. HRESIMS spectrum of compound **8**

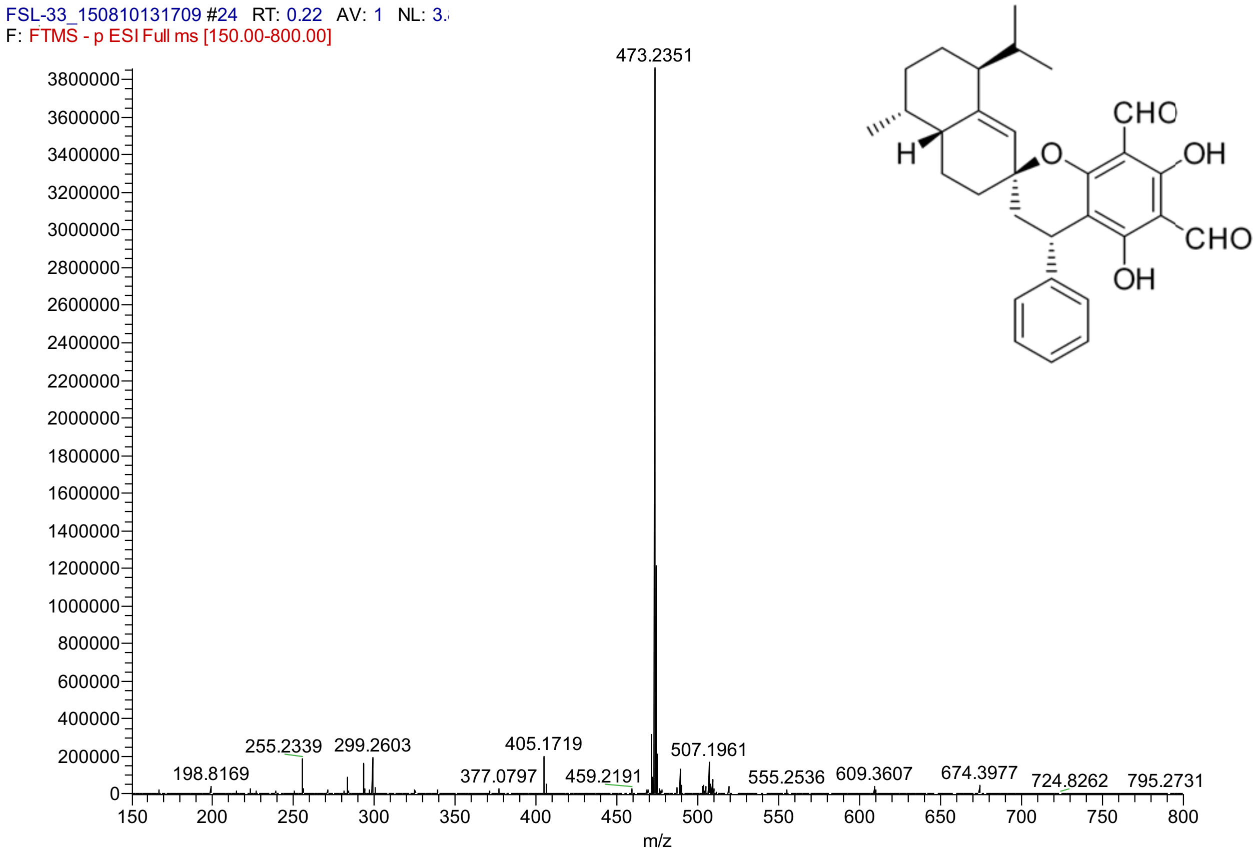

S7.17. ESIMS spectrum of compound **9**

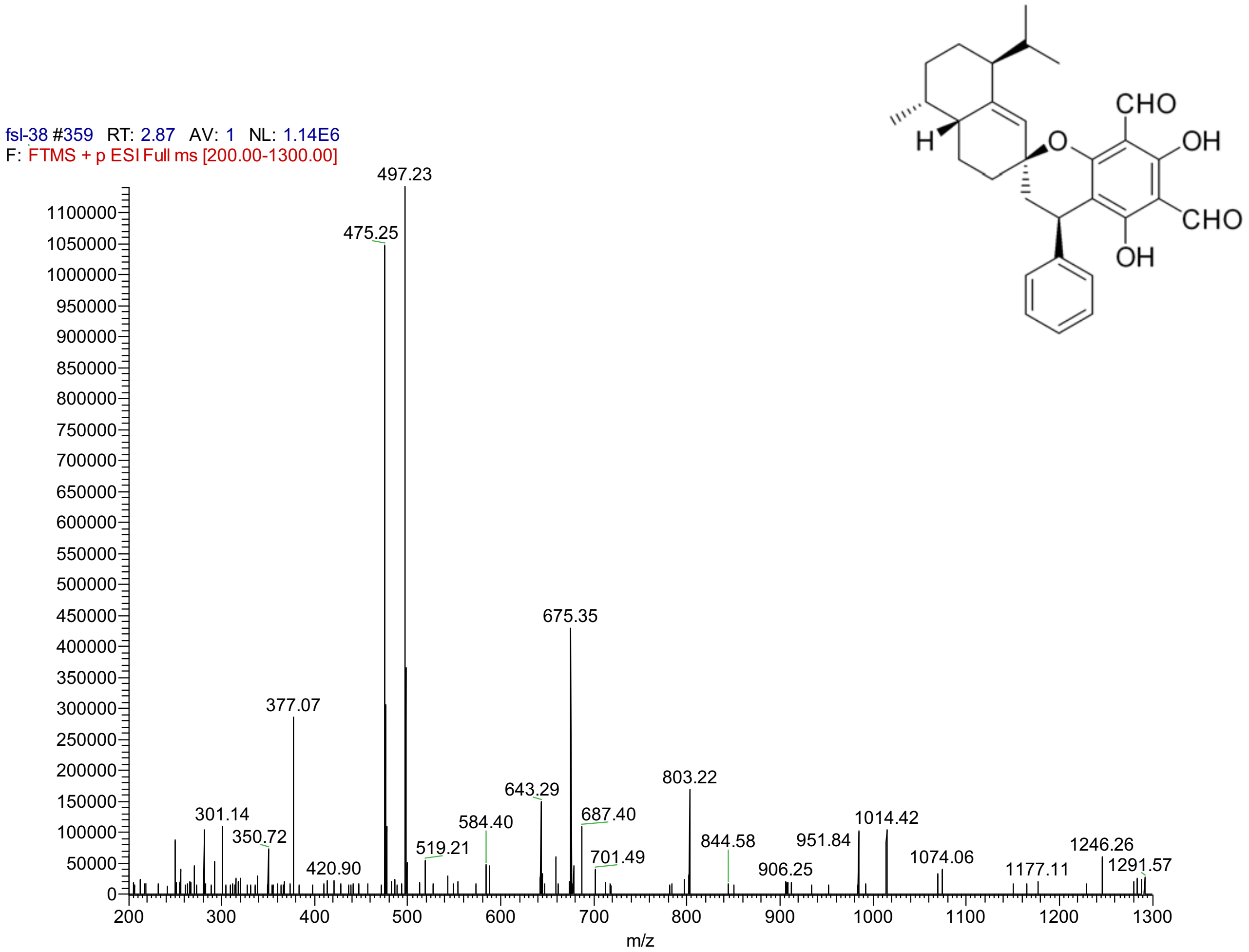

S7.18. HRESIMS spectrum of compound **9**

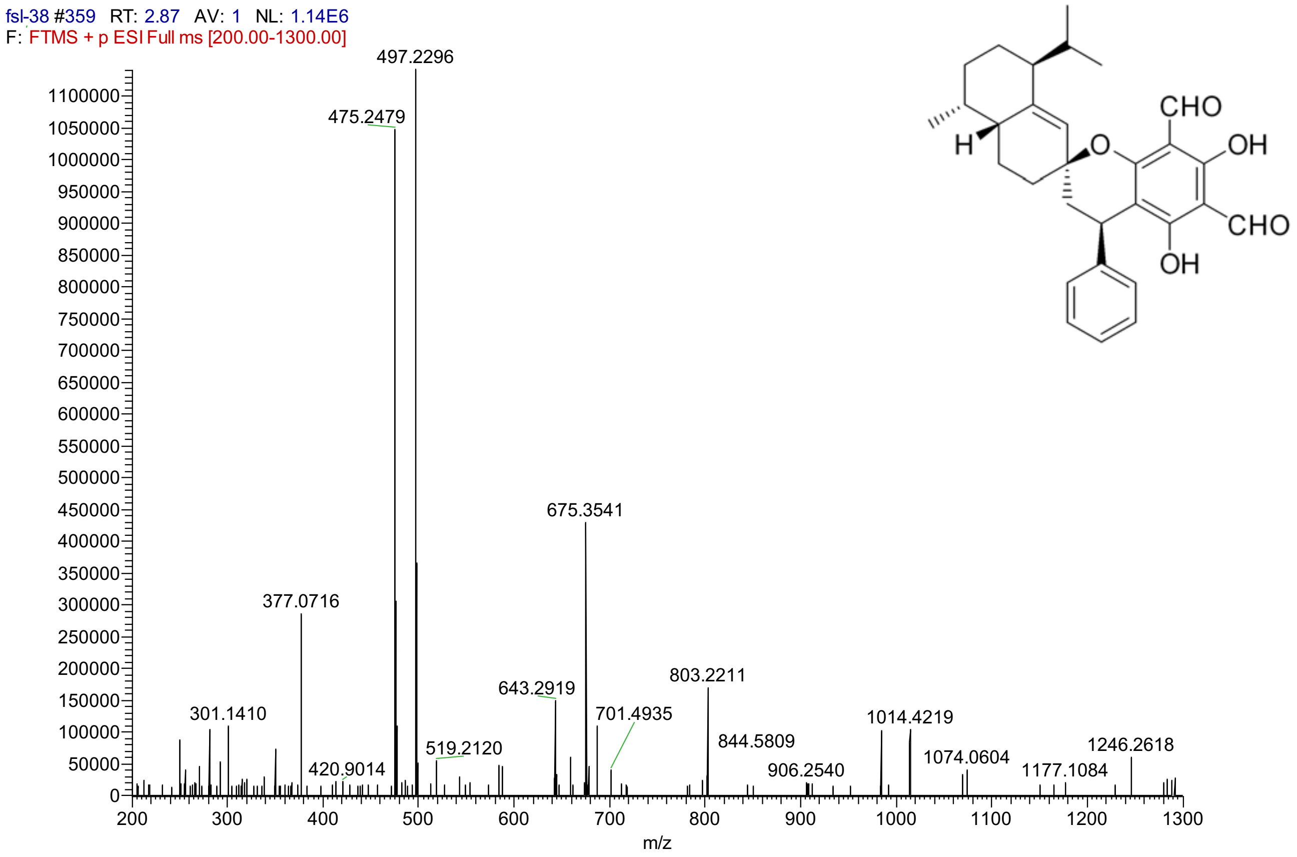

S7.19. ESIMS spectrum of compound **10**

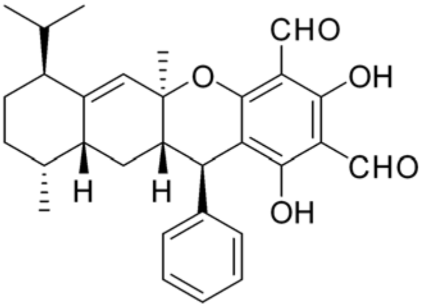

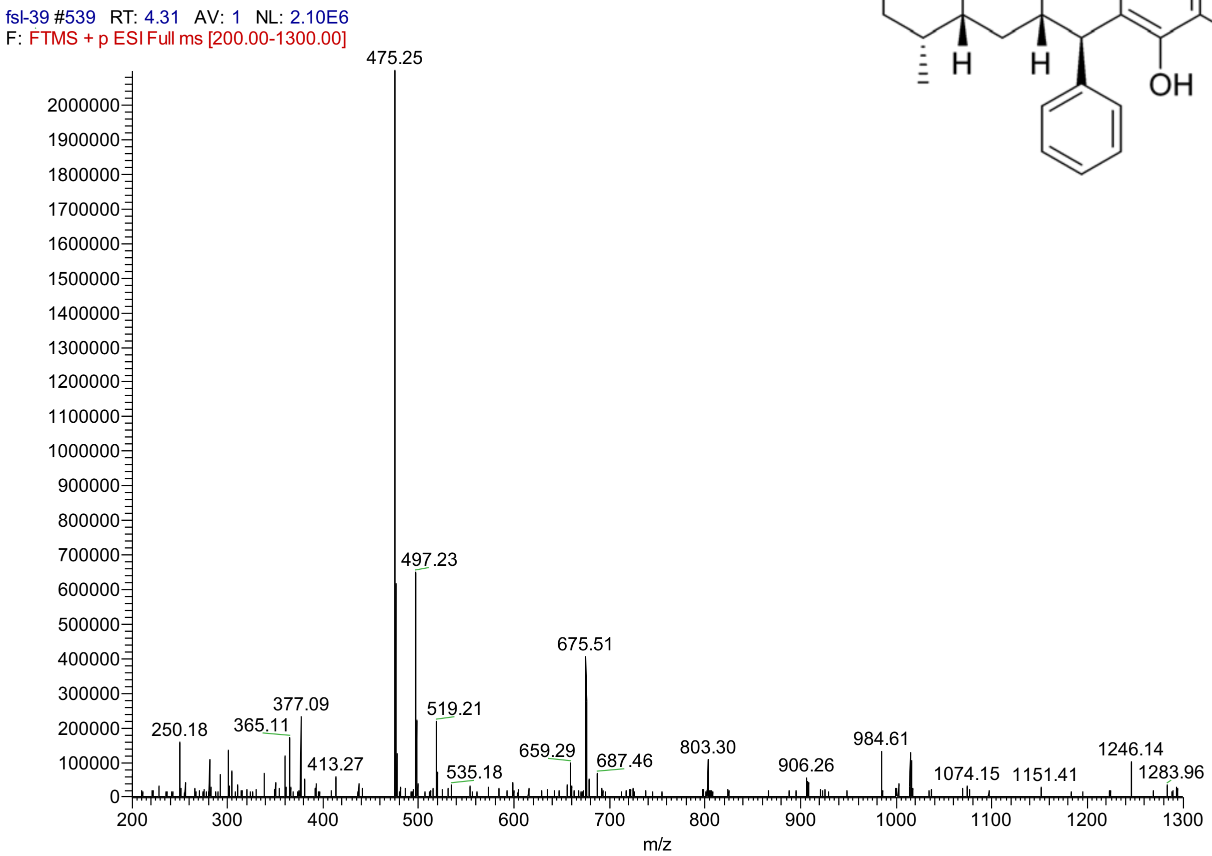

S7.20. HRESIMS spectrum of compound **10**

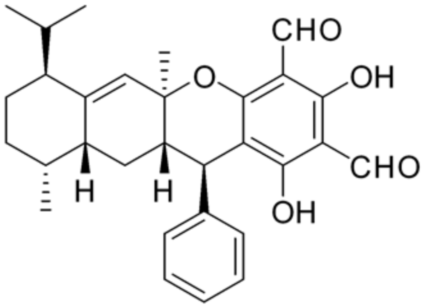

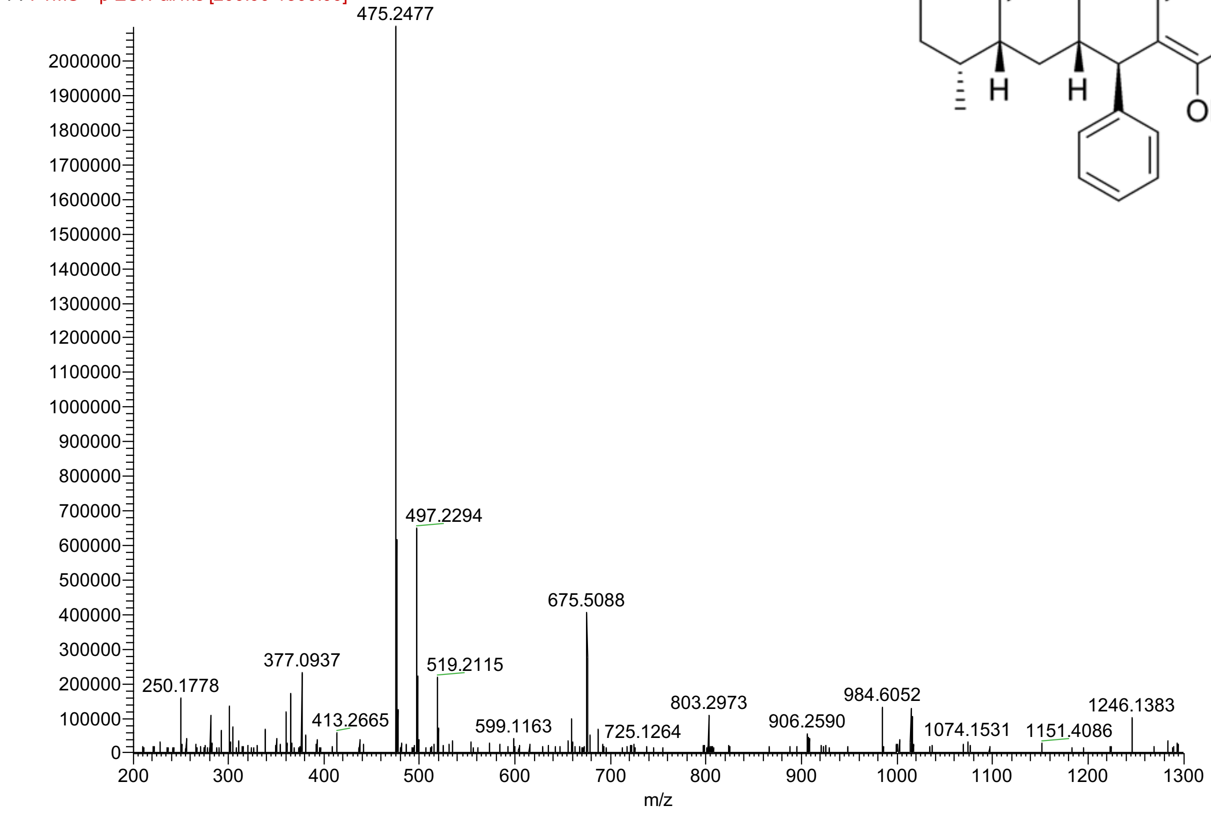

S7.21. ESIMS spectrum of compound **11**

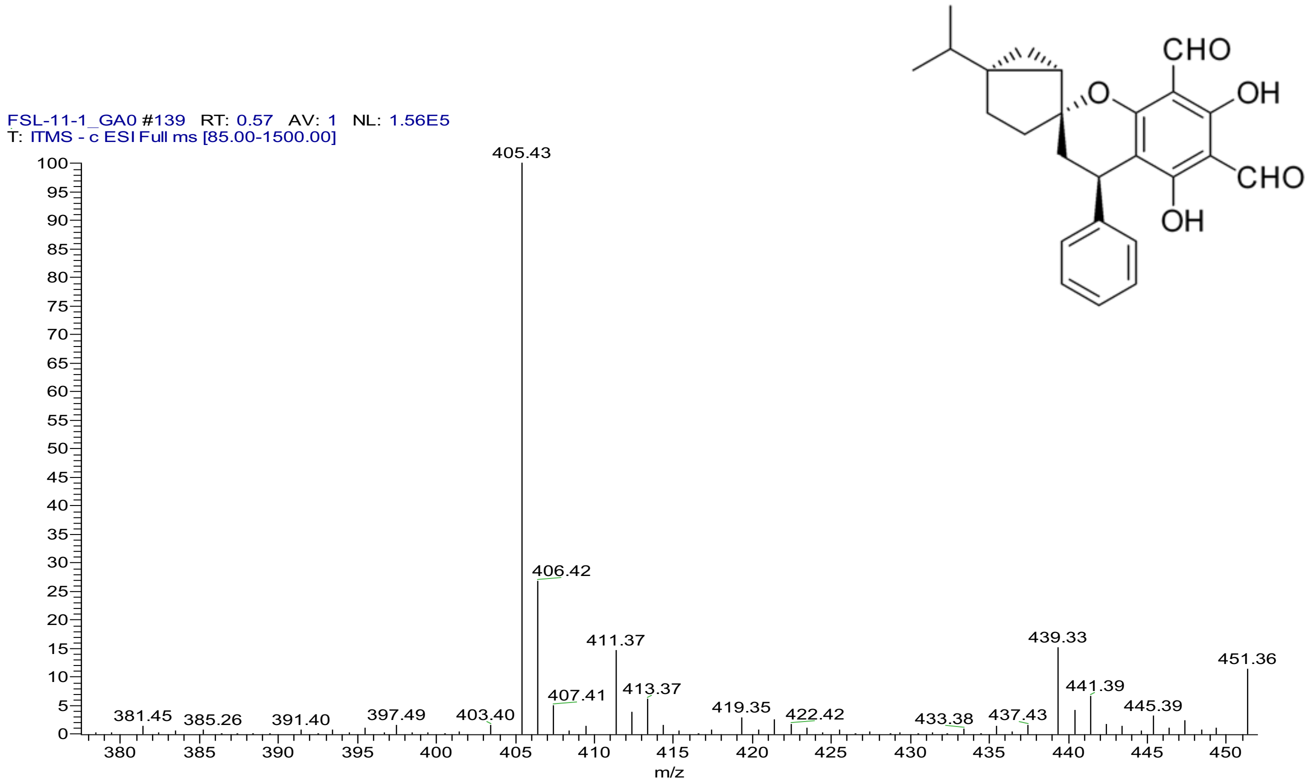

S7.22. HRESIMS spectrum of compound **11**

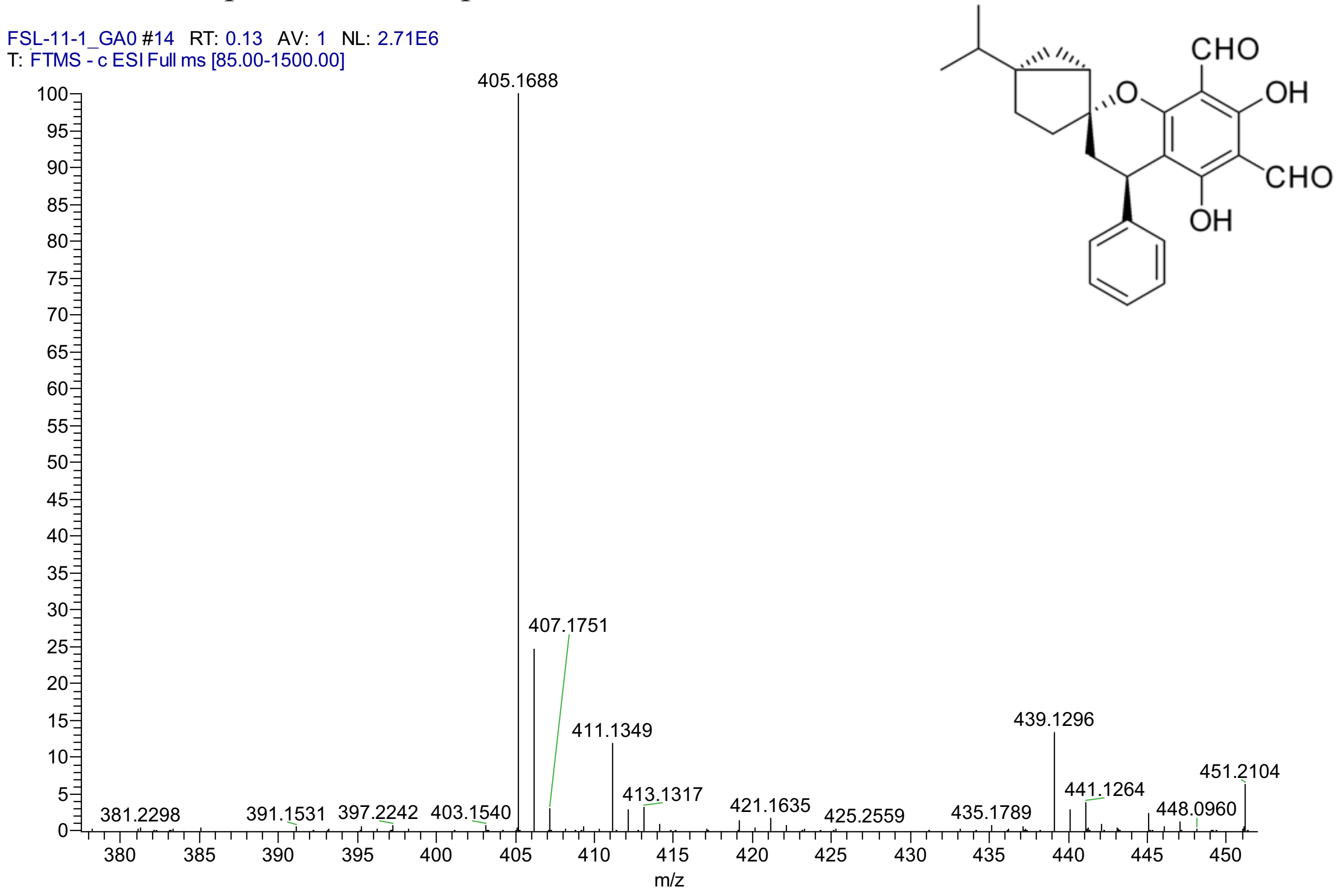

S7.23. ESIMS spectrum of compound **12**

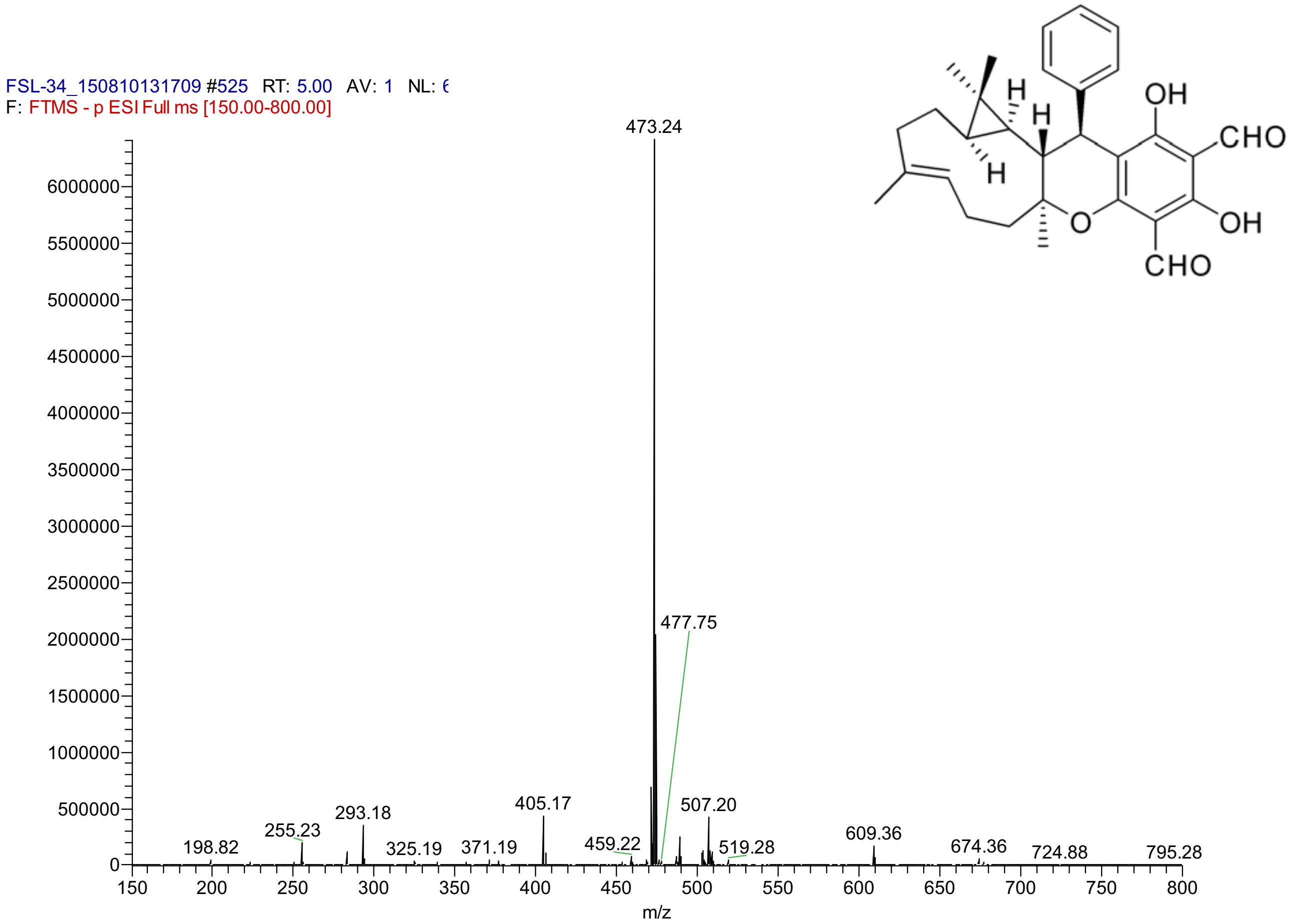

S7.24. HRESIMS spectrum of compound **12**

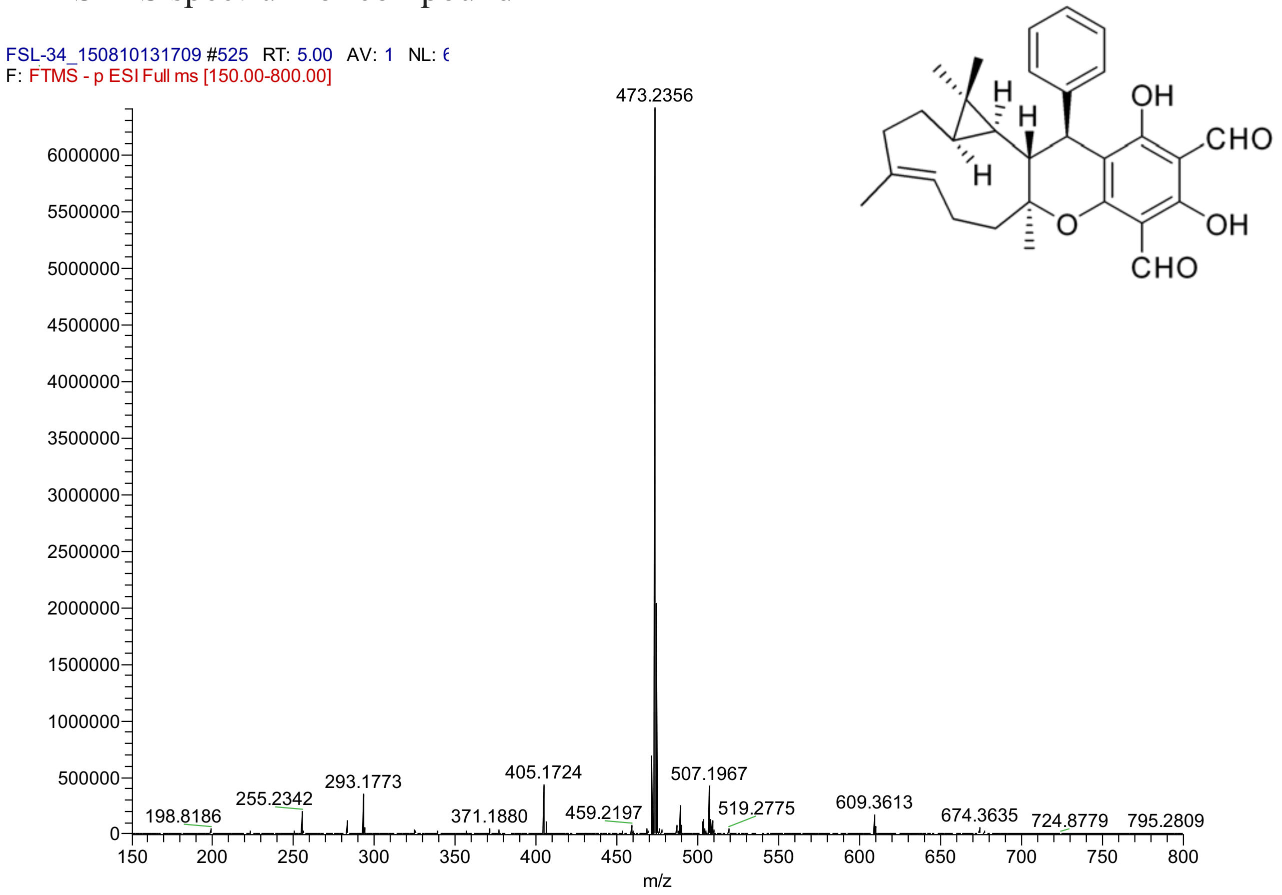

S7.25. ESIMS spectrum of compound **25**

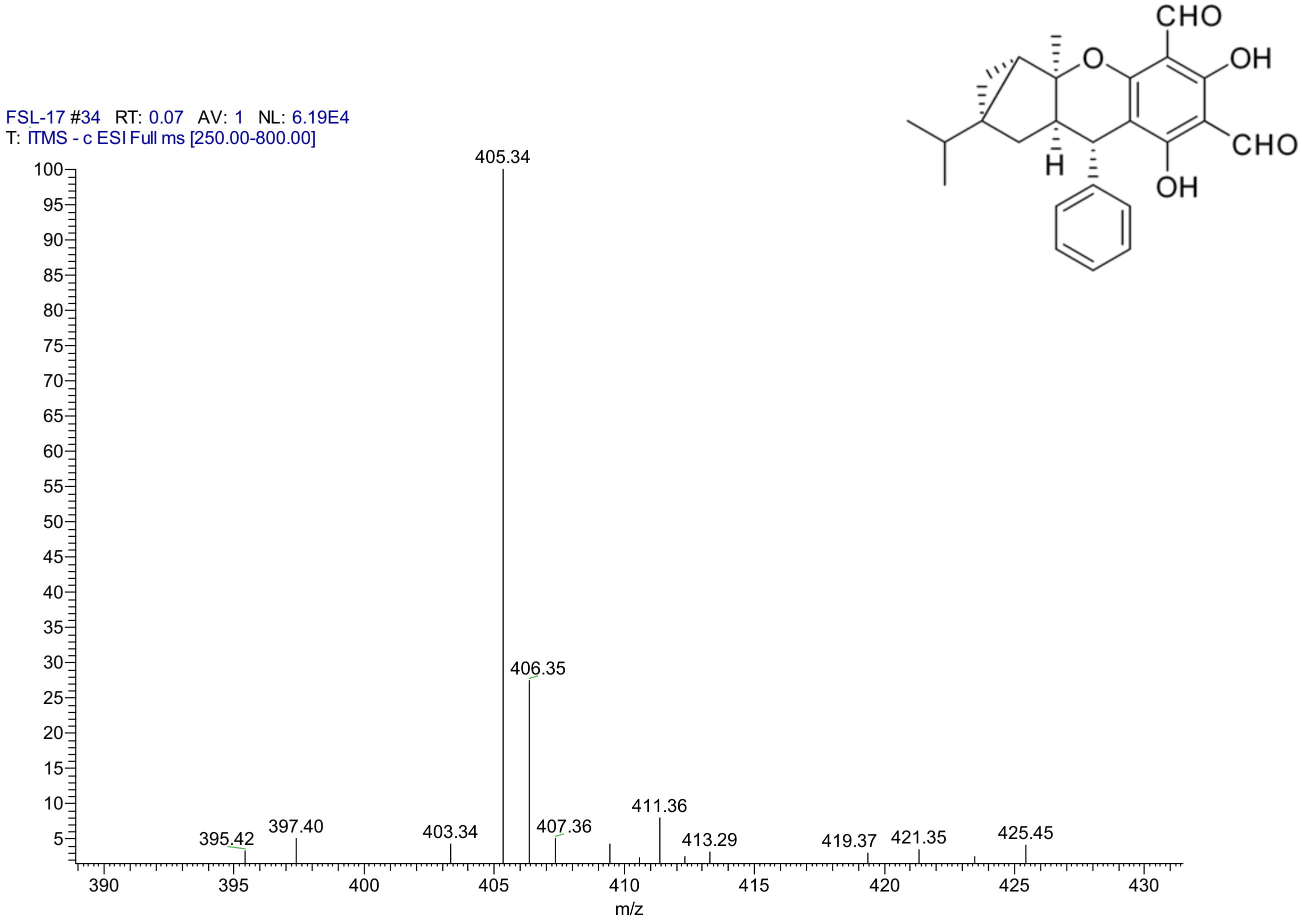

S7.26. HRESIMS spectrum of compound **25**

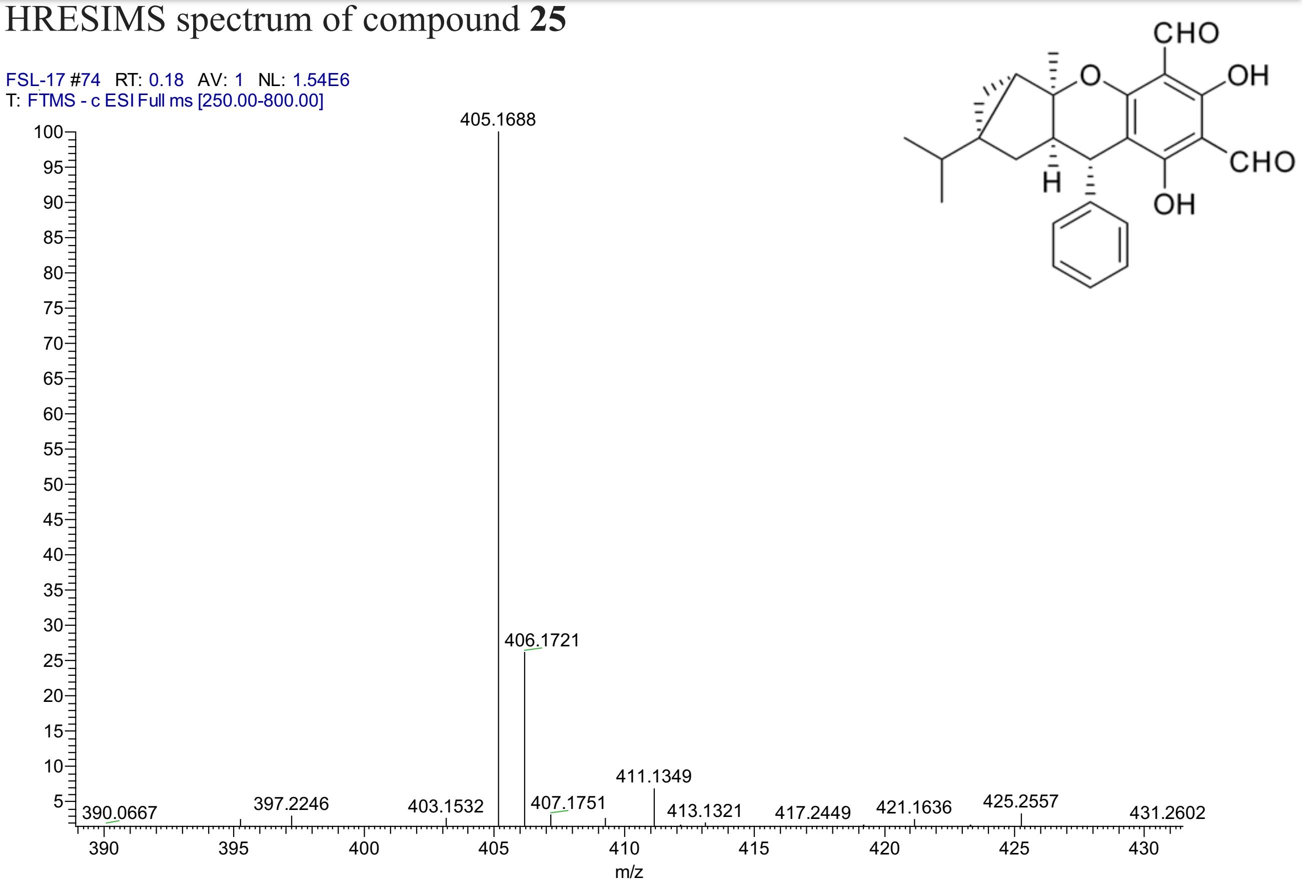

S7.27. IR (KBr disc) spectrum of compound **1**

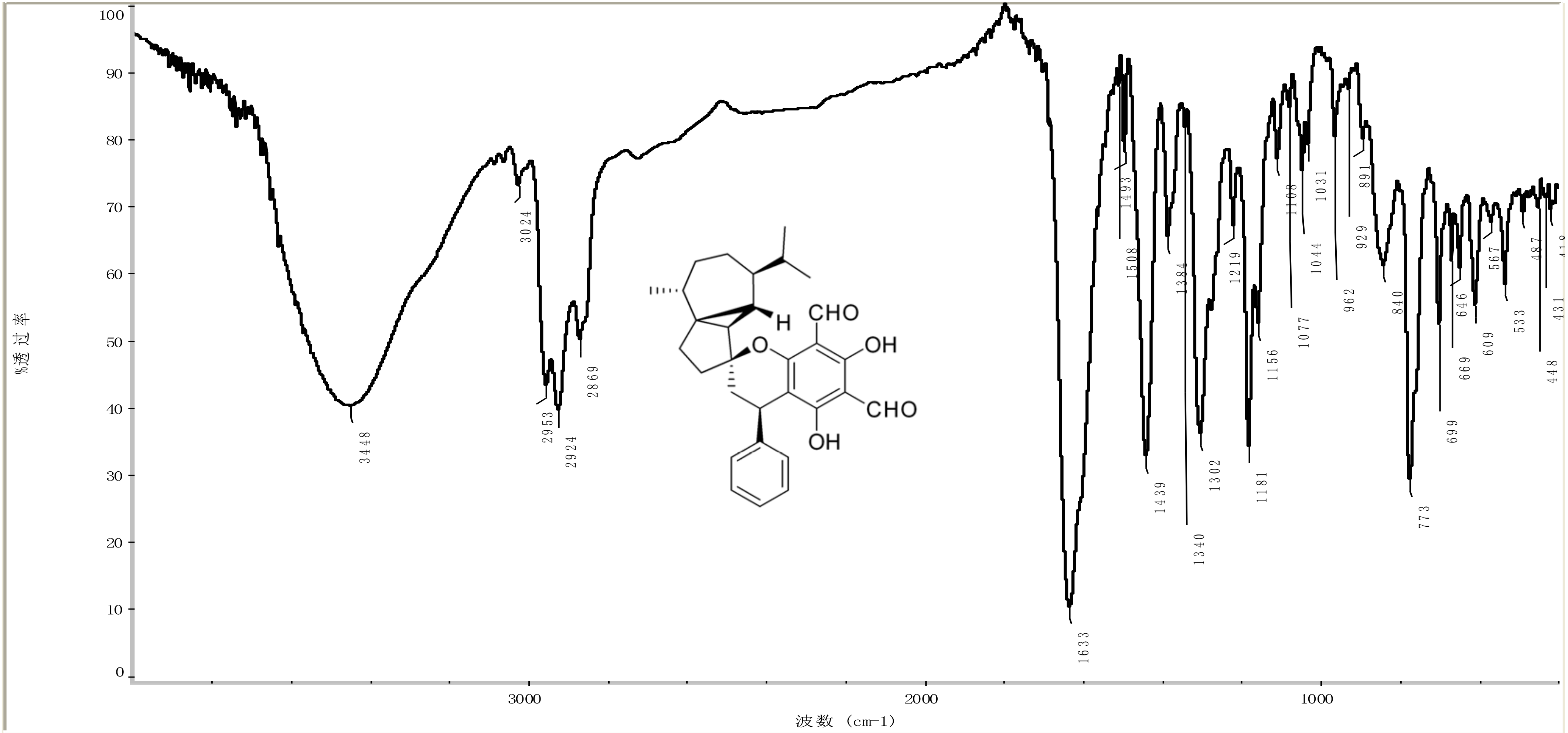

S7.28. IR (KBr disc) spectrum of compound **2**

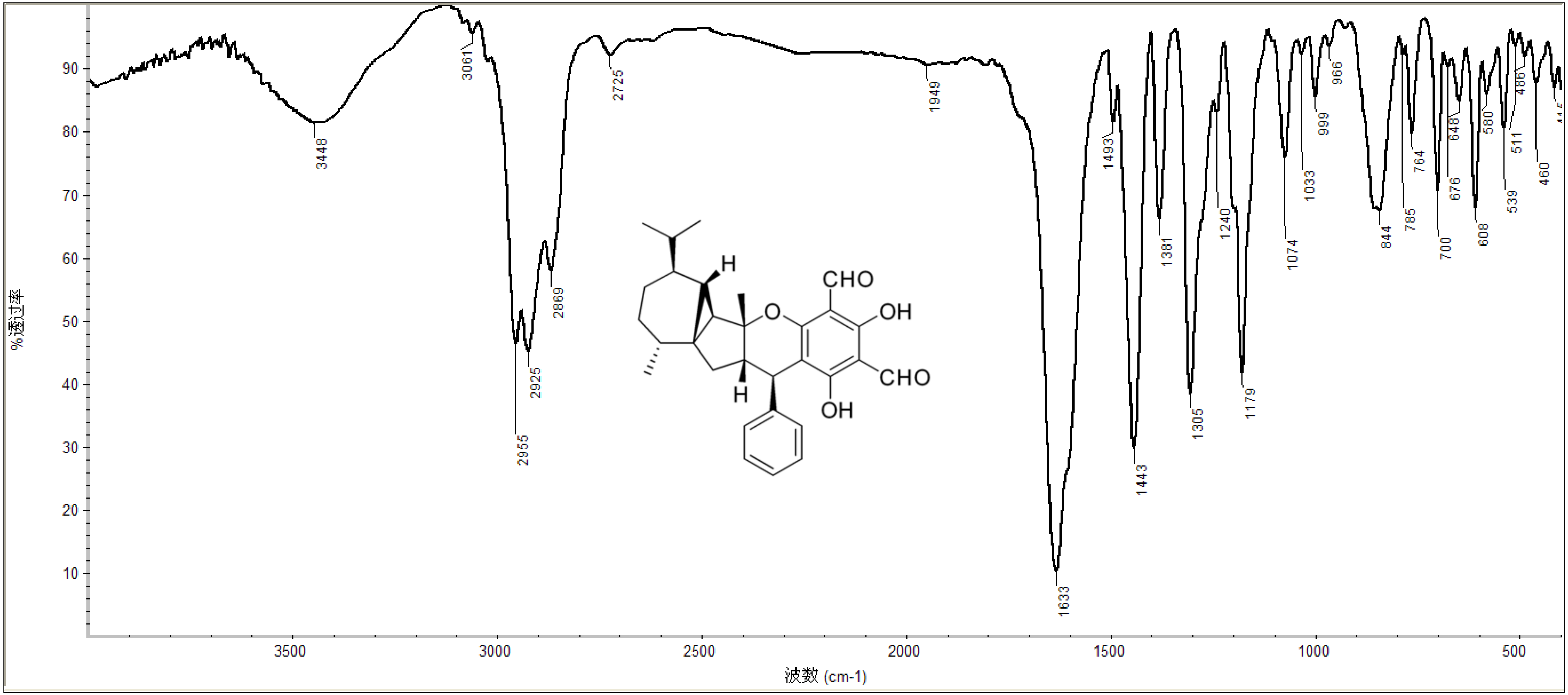

S7.29. IR (KBr disc) spectrum of compound **3**

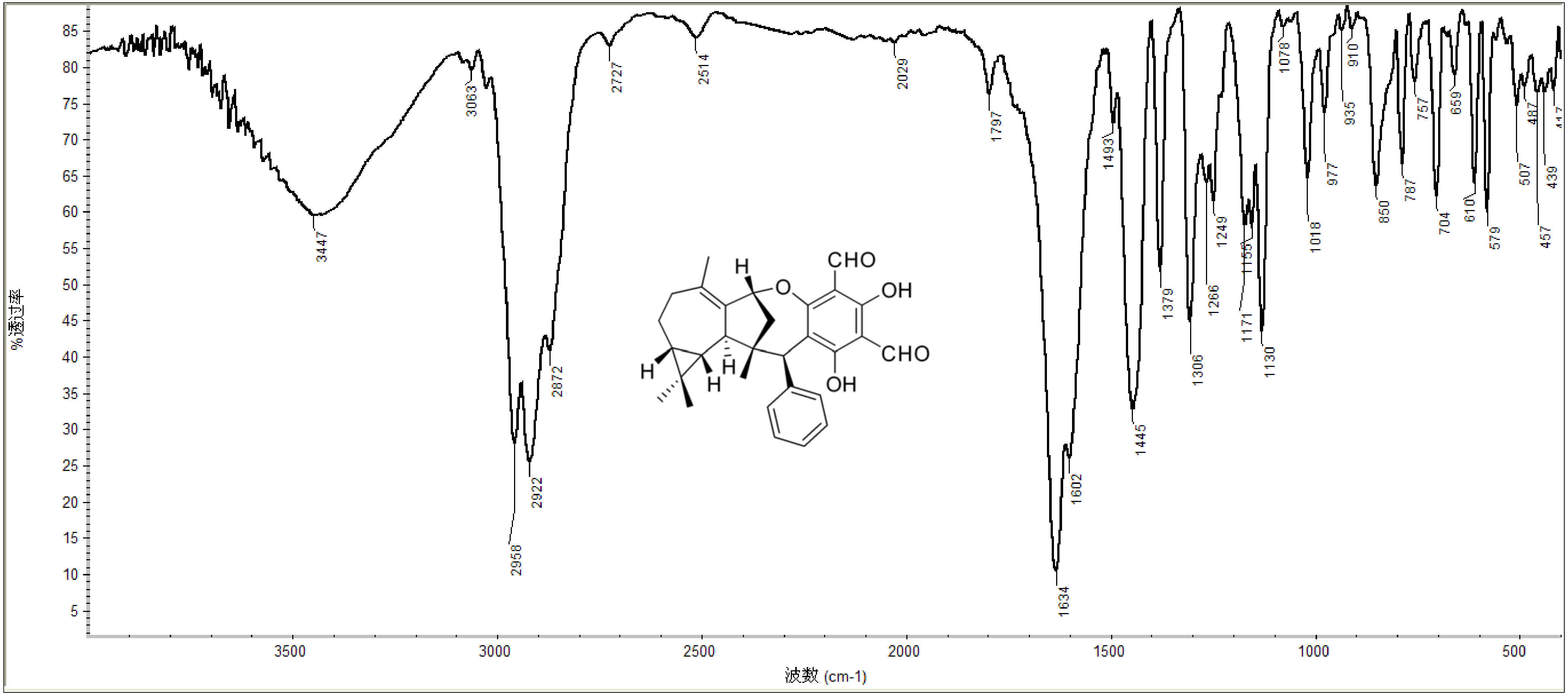

S7.30. IR (KBr disc) spectrum of compound **4**

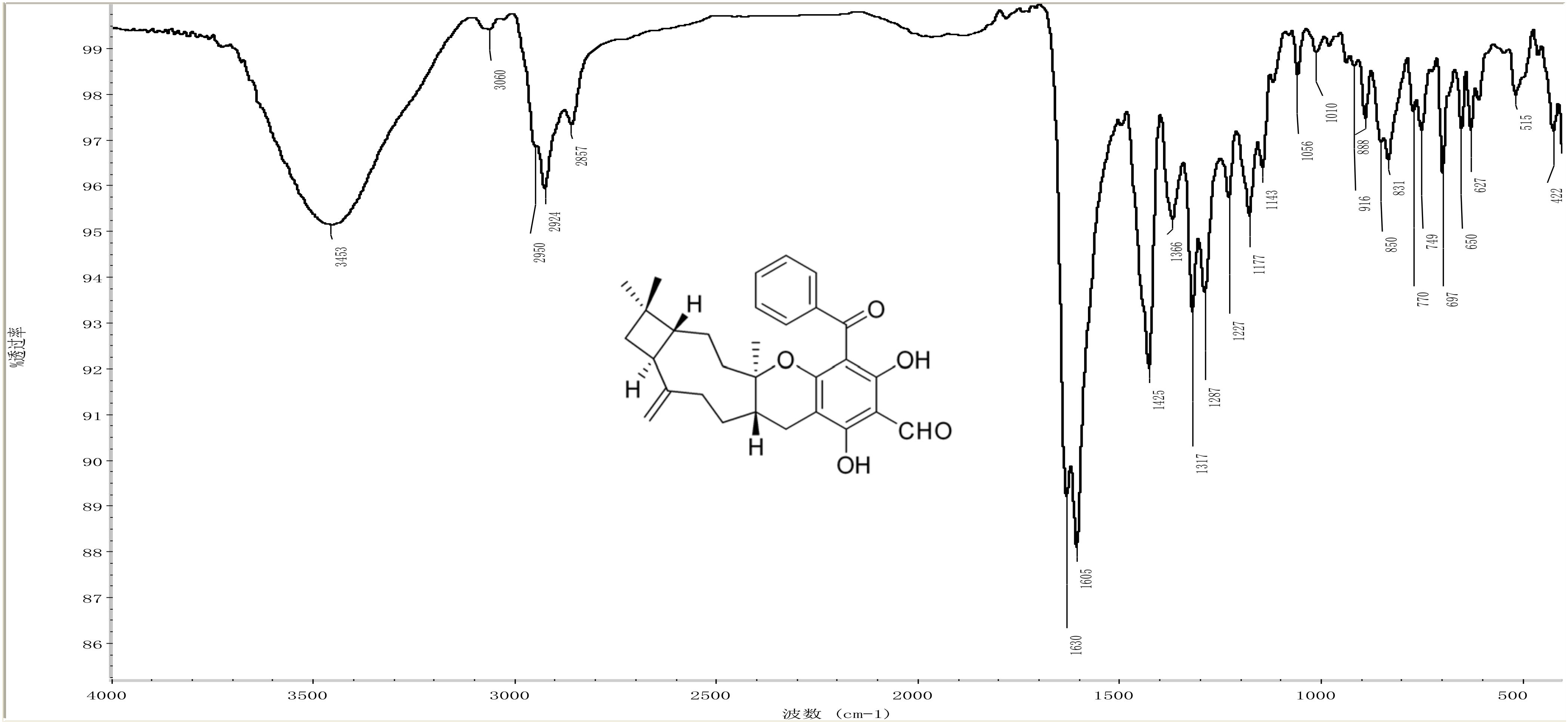

S7.31. IR (KBr disc) spectrum of compound **5**

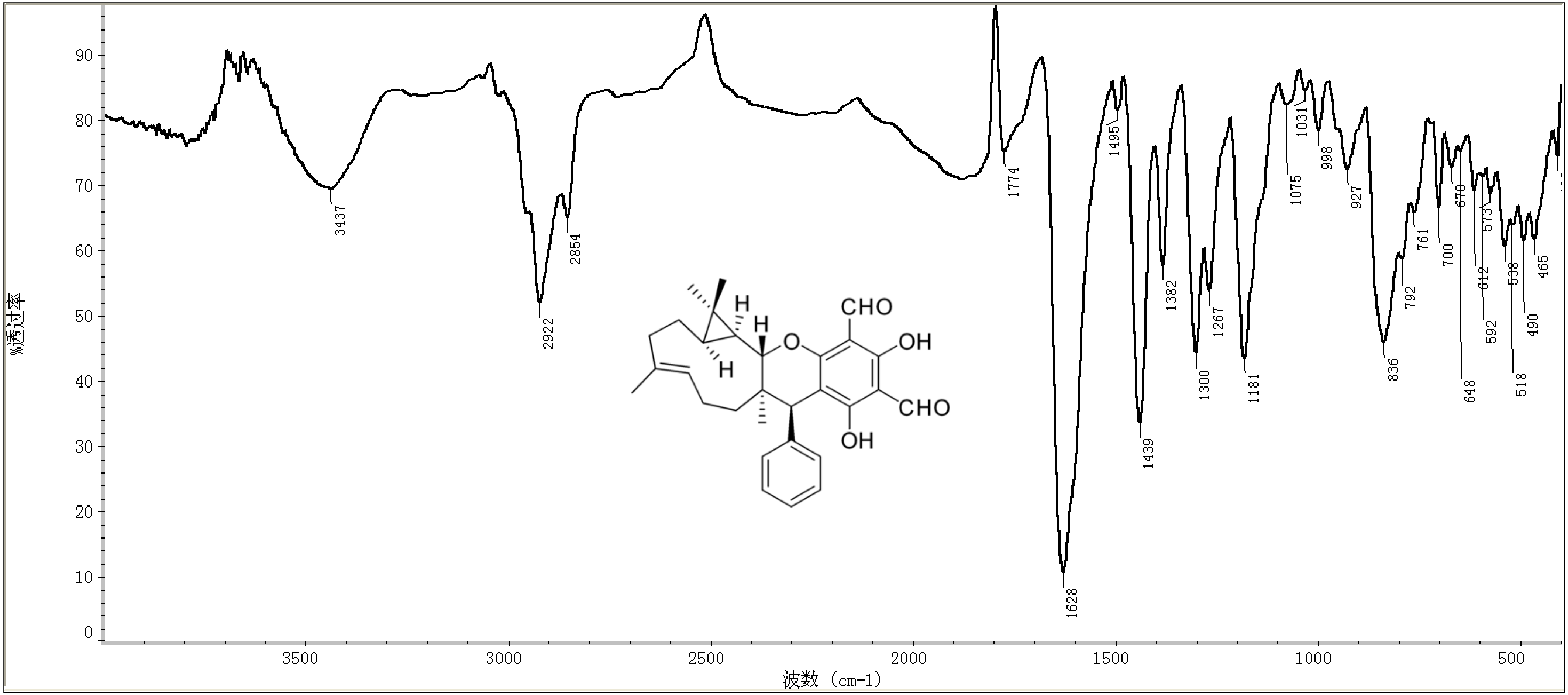

S7.32. IR (KBr disc) spectrum of compound **6**

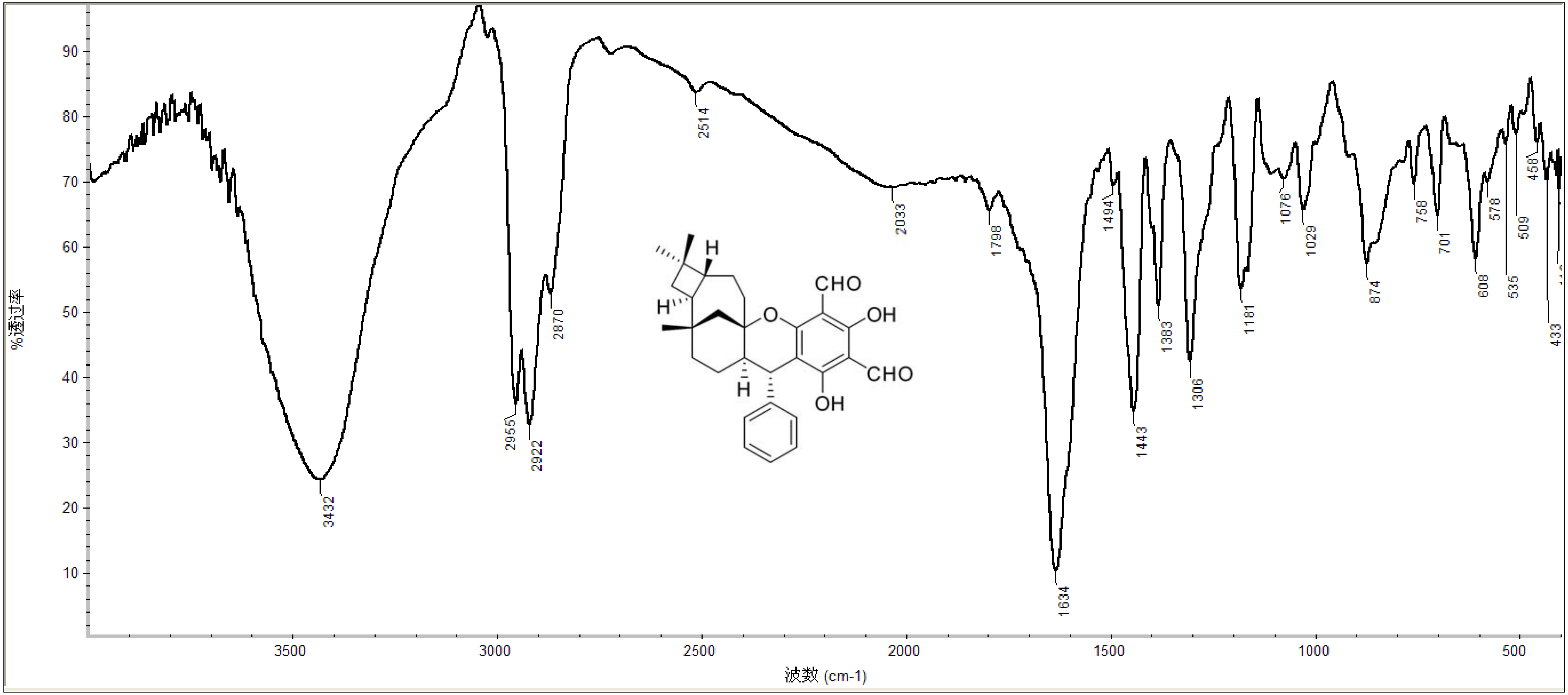

S7.33. IR (KBr disc) spectrum of compound **7**

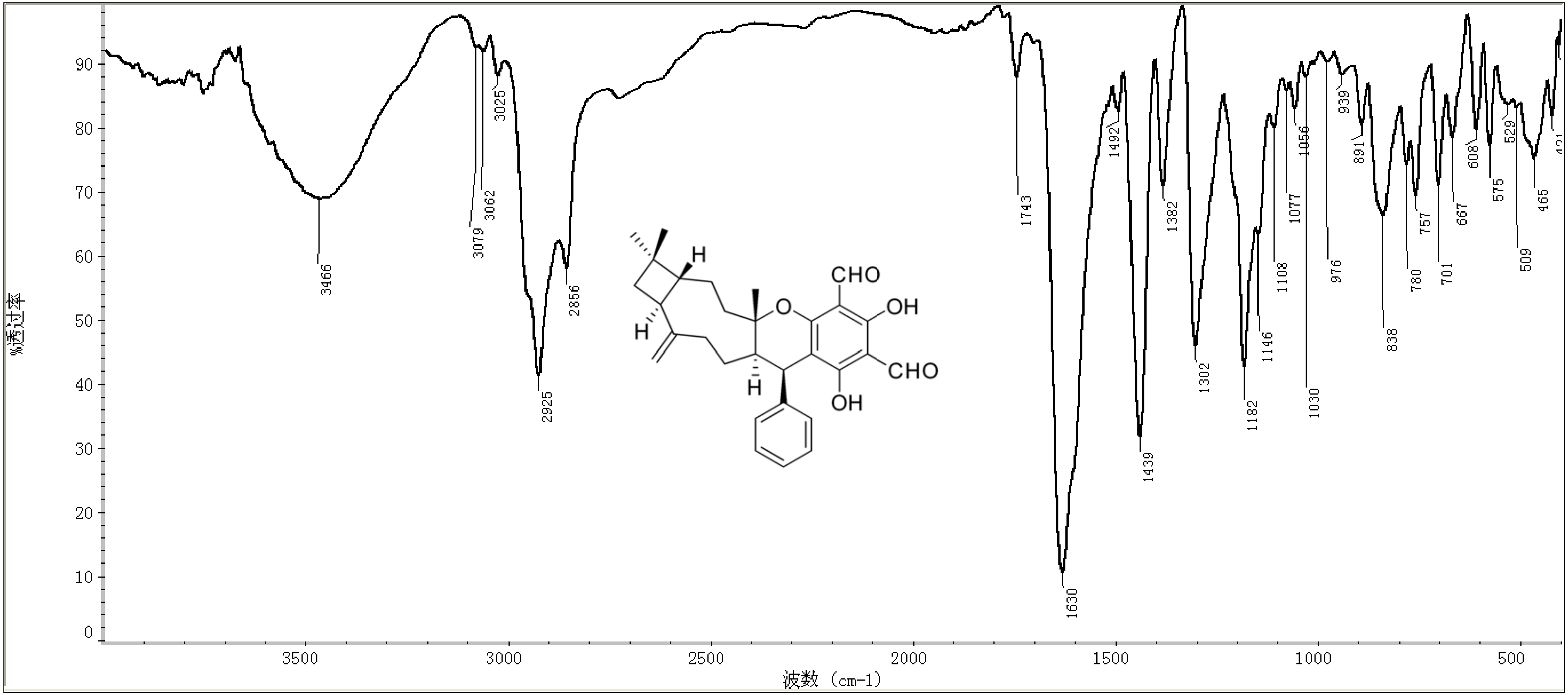

S7.34. IR (KBr disc) spectrum of compound **8**

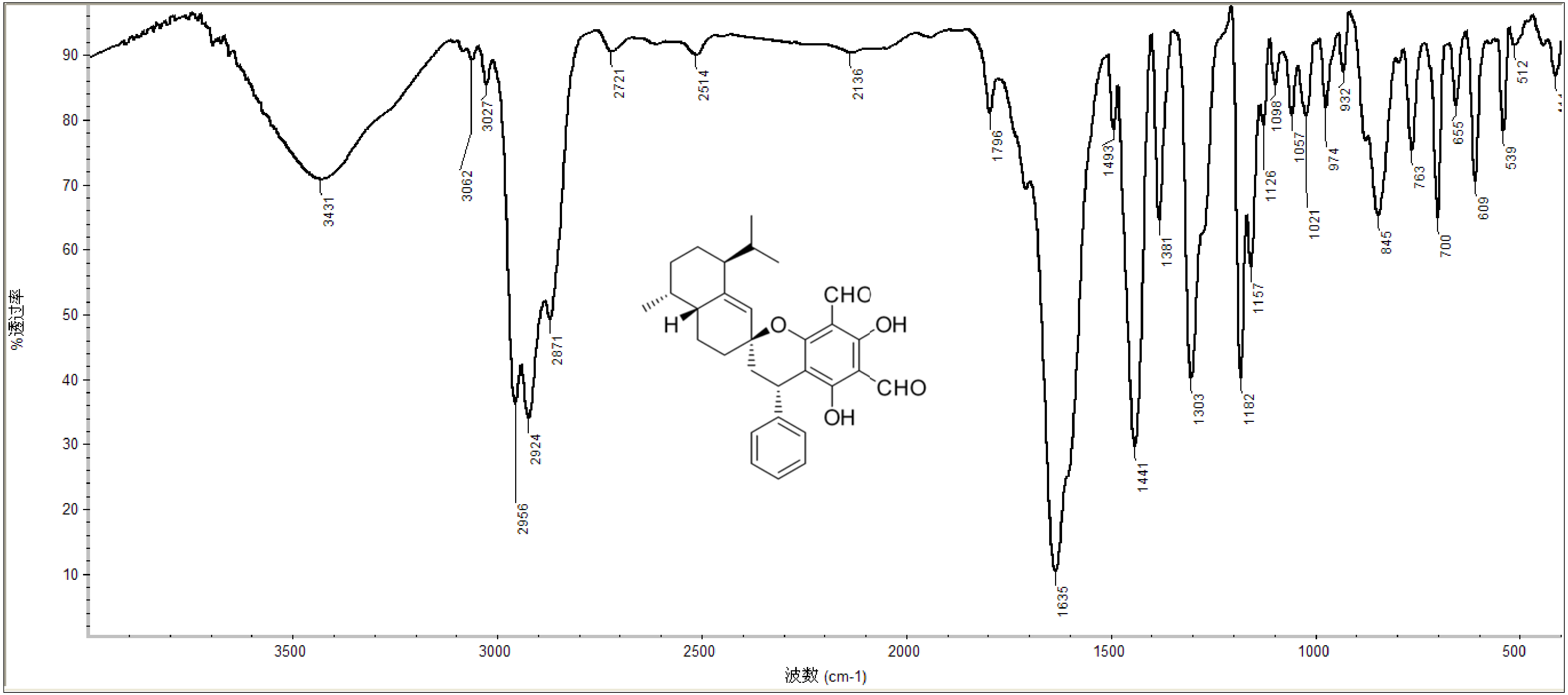

S7.35. IR (KBr disc) spectrum of compound **9**

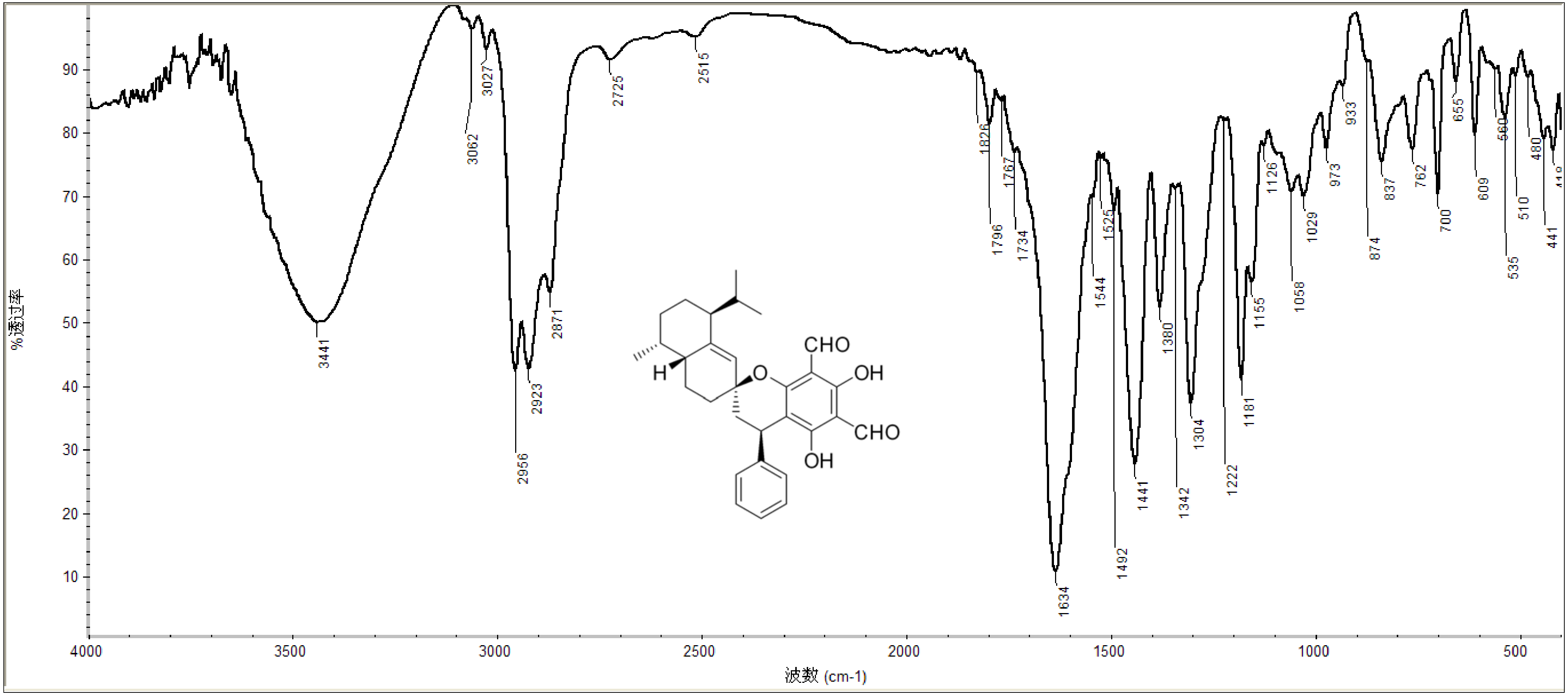

S7.36. IR (KBr disc) spectrum of compound **10**

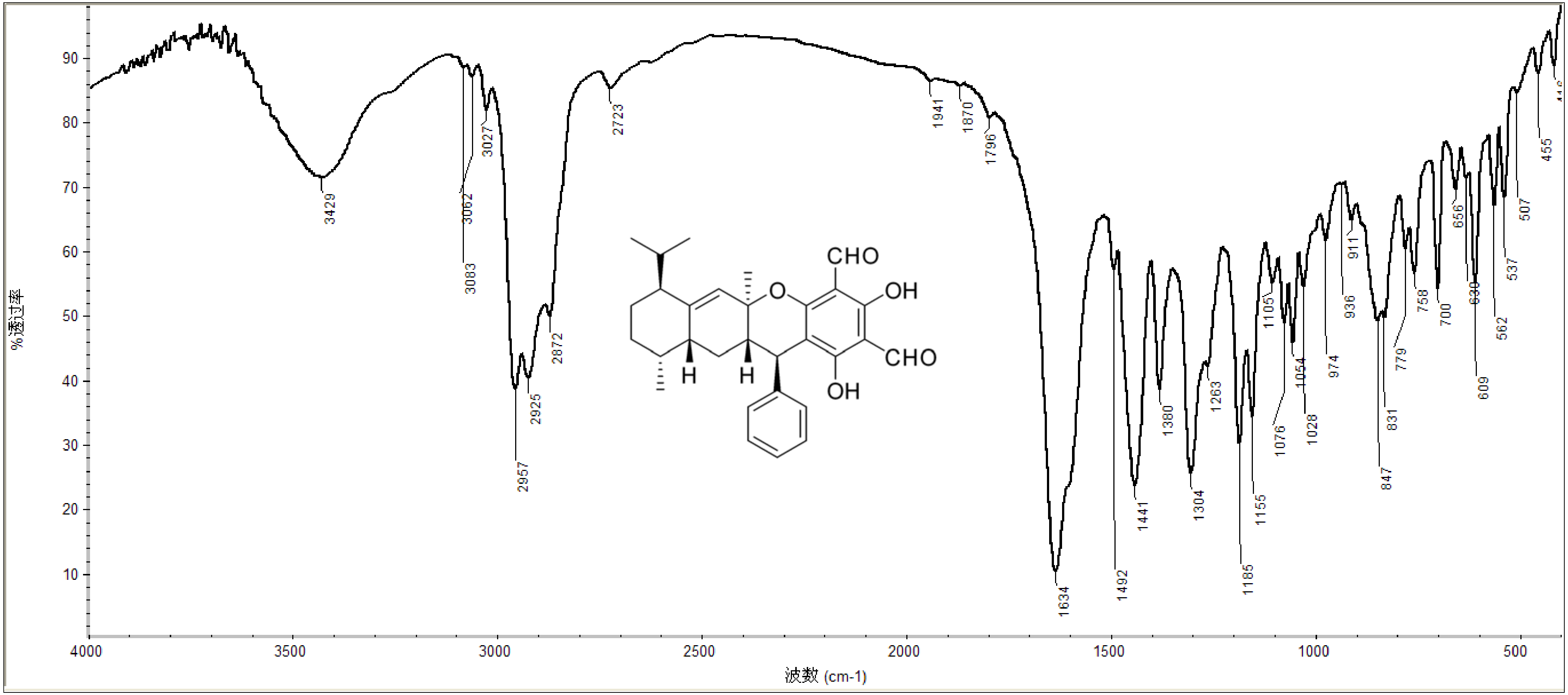

S7.37. IR (KBr disc) spectrum of compound **11**

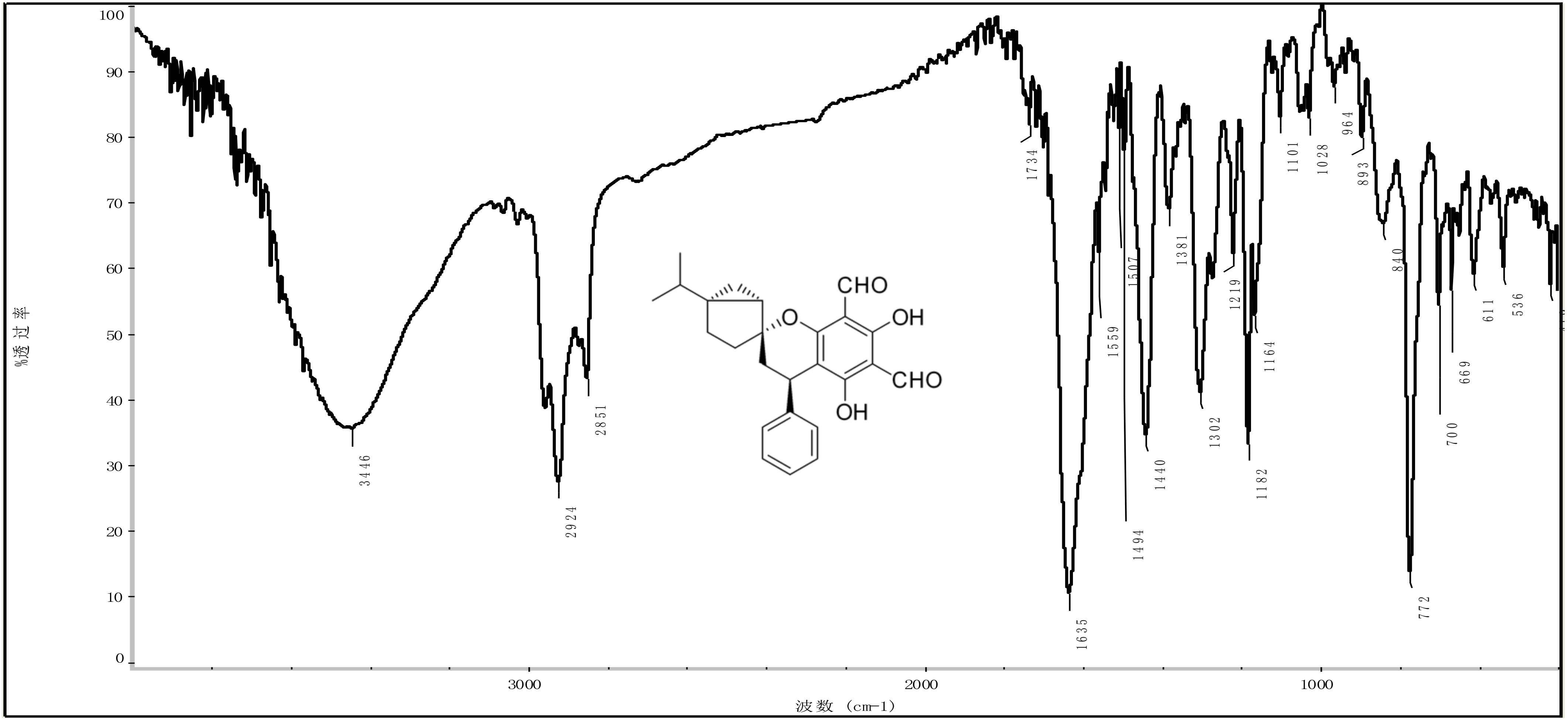

S7.38. IR (KBr disc) spectrum of compound **12**

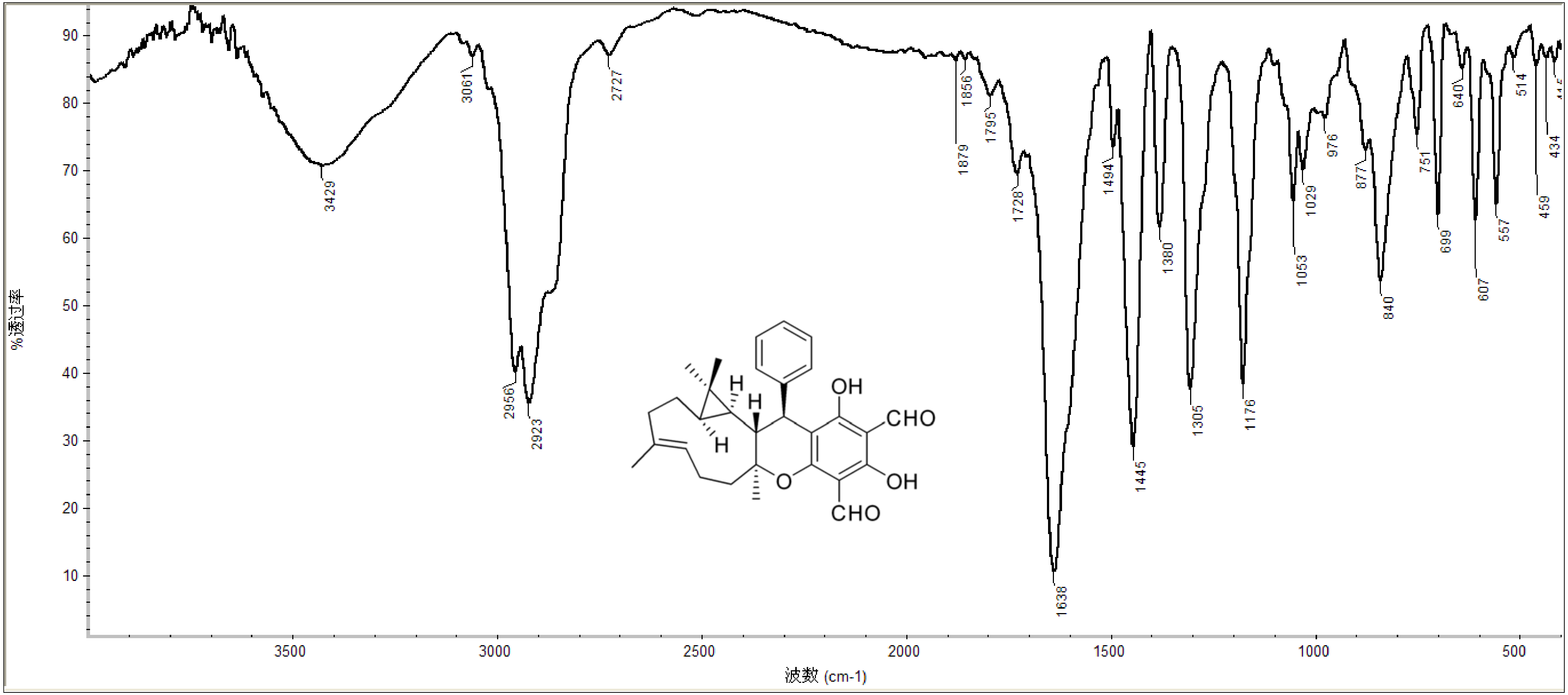

S7.39. IR (KBr disc) spectrum of compound **25**

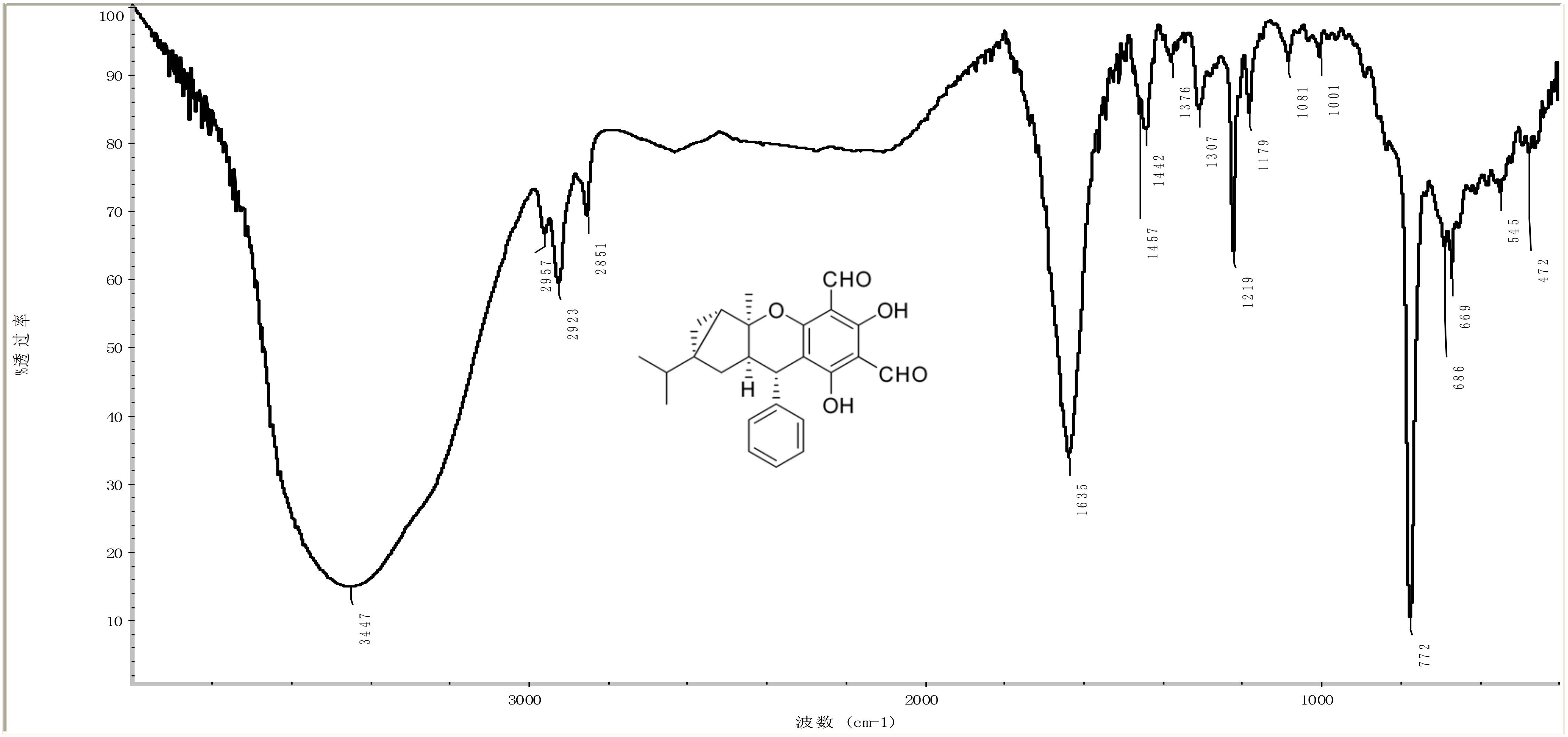

S7.40. UV spectrum of compound **1**

Spectrum Peak Pick Report

2014-11-26

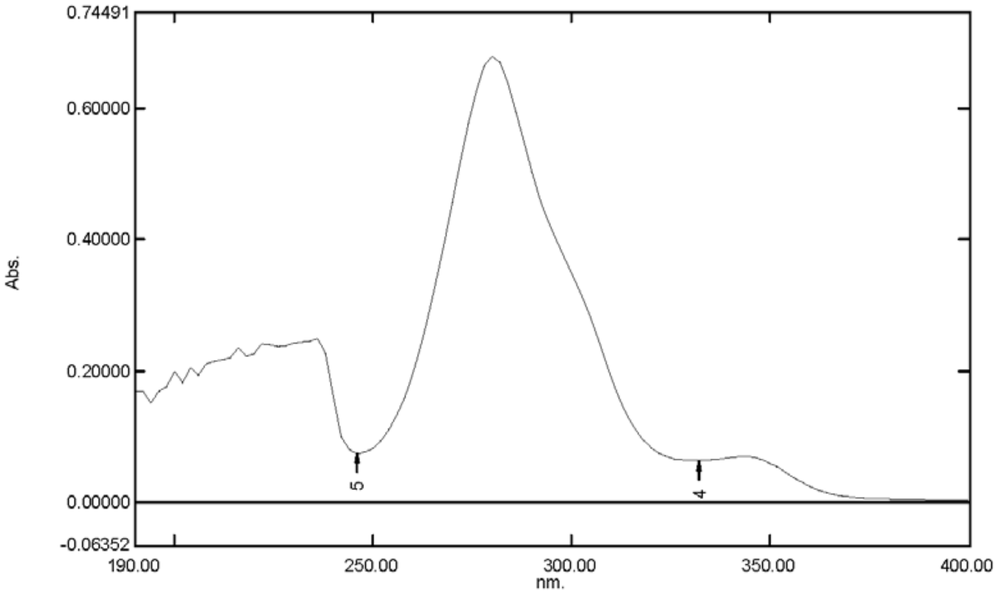

Measurement Properties
Wavelength Range (nm.): 190.00 to 400.00
Scan Speed: Medium
Sampling Interval: 2.0
Auto Sampling Interval: Disabled
Scan Mode: Auto

No.	P/V	Wavelength	Abs.
1	↑	344.00	0.07030
2	↑	280.00	0.67754
3	↑	236.00	0.24948
4	↓	332.00	0.06401
5	↓	246.00	0.07440

Sample Preparation Properties
Weight:
Volume:
Dilution:
Path Length:
Additional Information:

Instrument Properties
Instrument Type: UV-2400PC Series
Measuring Mode: Absorbance
Slit Width: 2.0 nm
Light Source Change Wavelength: 360.0 nm
S/R Exchange: Normal

Attachment Properties
Attachment: None

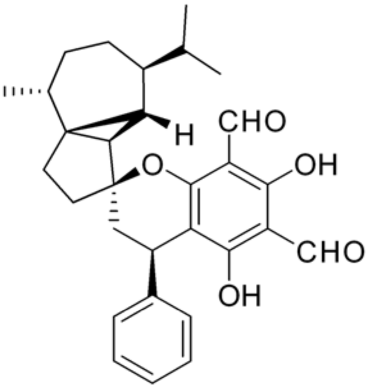

S7.41. UV spectrum of compound **2**

Spectrum Peak Pick Report 2015-08-01

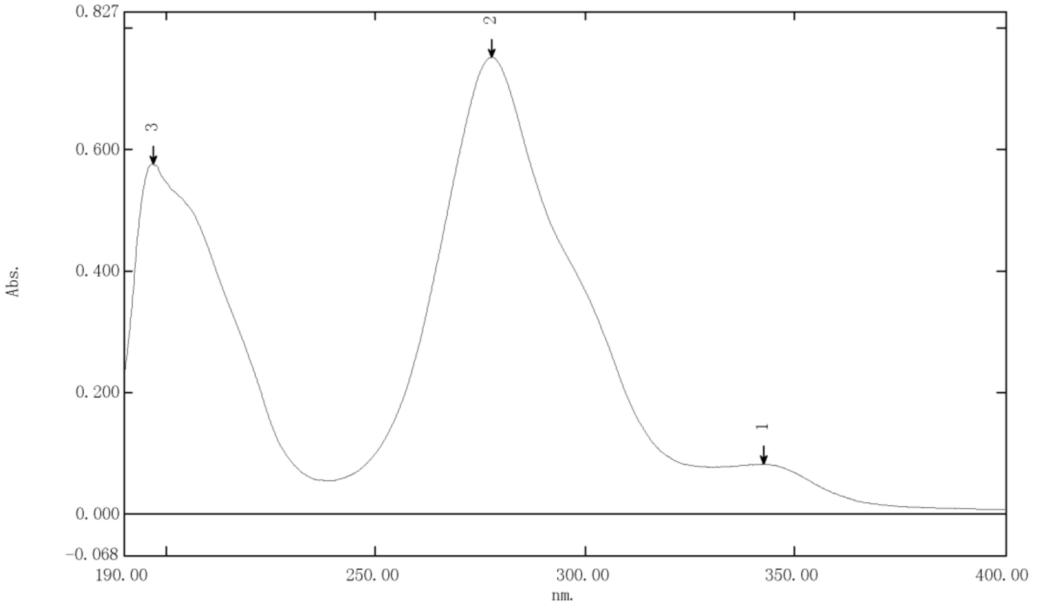

Measurement Properties
Wavelength Range (nm.): 190.00 to 400.00
Scan Speed: Medium
Sampling Interval: 2.0
Auto Sampling Interval: Disabled
Scan Mode: Auto

Sample Preparation Properties
Weight:
Volume:
Dilution:
Path Length:
Additional Information:

Instrument Properties
Instrument Type: UV-2400PC Series
Measuring Mode: Absorbance
Slit Width: 2.0 nm
Light Source Change Wavelength: 360.0 nm
S/R Exchange: Normal

No.	P/V	波长(nm)	吸收值
1	↑	342. 60	0. 082
2	↑	277. 70	0. 753
3	↑	196. 90	0. 577
4	↓	239. 00	0. 054

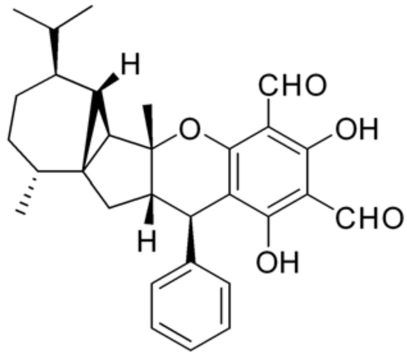

S7.42. UV spectrum of compound **3**

Spectrum Peak Pick Report 2015-08-01

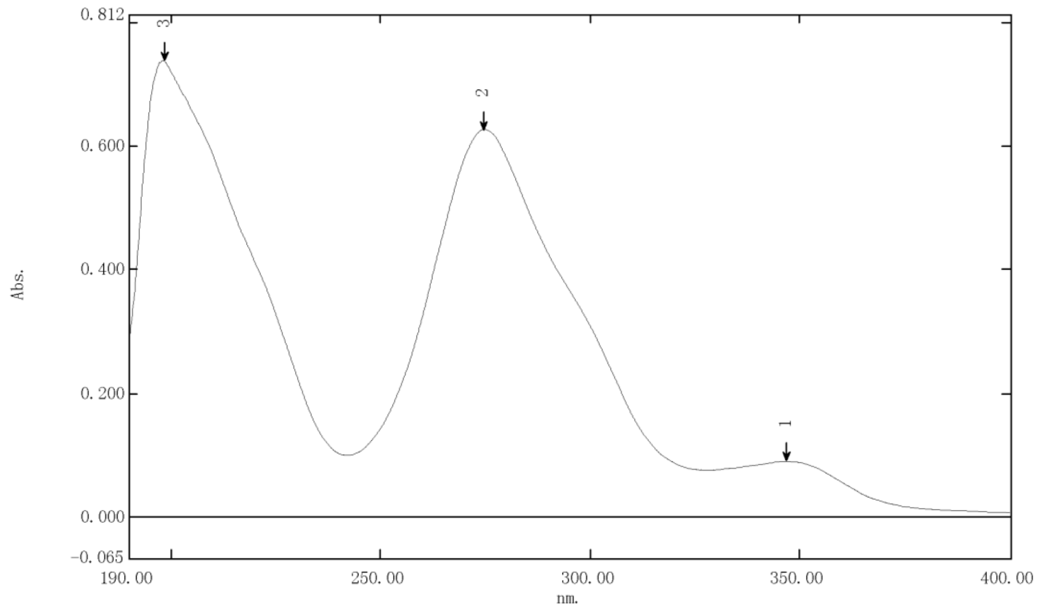

Measurement Properties
Wavelength Range (nm.): 190.00 to 400.00
Scan Speed: Medium
Sampling Interval: 2.0
Auto Sampling Interval: Disabled
Scan Mode: Auto

Sample Preparation Properties
Weight:
Volume:
Dilution:
Path Length:
Additional Information:

Instrument Properties
Instrument Type: UV-2400PC Series
Measuring Mode: Absorbance
Slit Width: 2.0 nm
Light Source Change Wavelength: 360.0 nm
S/R Exchange: Normal

No.	P/V	波长(nm)	吸收值
1	↑	346. 70	0. 091
2	↑	274. 50	0. 627
3	↑	198. 30	0. 739
4	↓	241. 80	0. 100

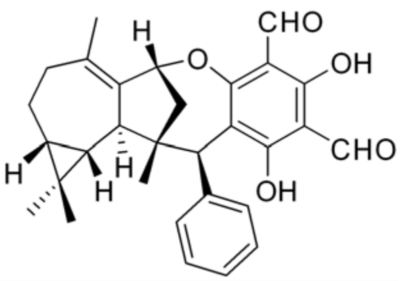

S7.43. UV spectrum of compound **4**

Spectrum Peak Pick Report

2015-01-05 02:50:57

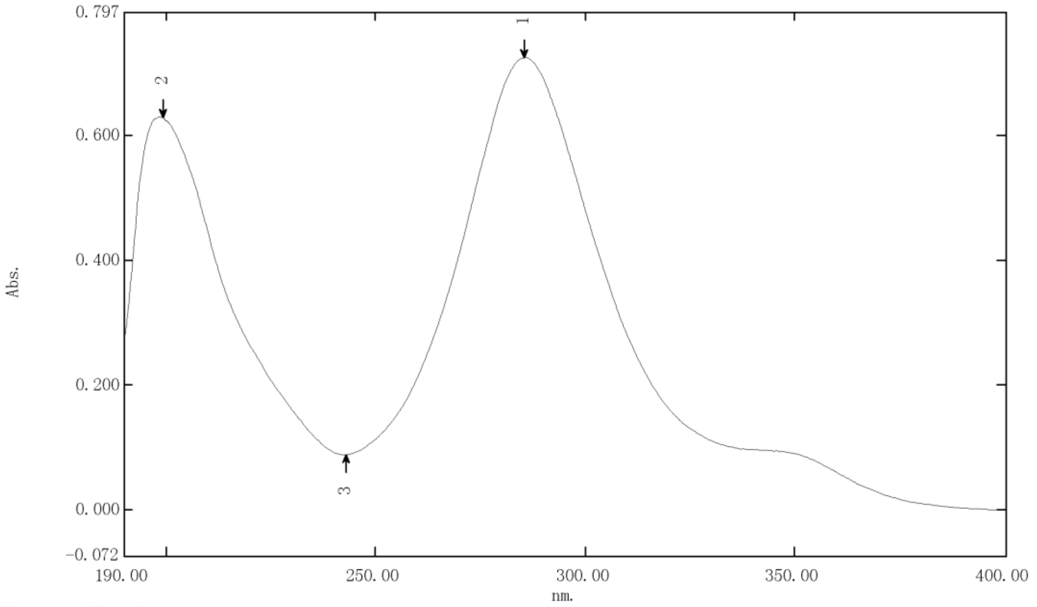

Measurement Properties	
Wavelength Range (nm.):	190.00 to 400.00
Scan Speed:	Medium
Sampling Interval:	2.0
Auto Sampling Interval:	Disabled
Scan Mode:	Auto

No.	P/V	波长(nm)	吸收值
1	↑	285. 40	0. 725
2	↑	199. 20	0. 630
3	↓	242. 80	0. 089

Sample Preparation Properties
Weight:
Volume:
Dilution:
Path Length:
Additional Information:

Instrument Properties	
Instrument Type:	UV-2400PC Series
Measuring Mode:	Absorbance
Slit Width:	2.0 nm
Light Source Change Wavelength:	360.0 nm
S/R Exchange:	Normal

Attachment Properties	
Attachment:	None

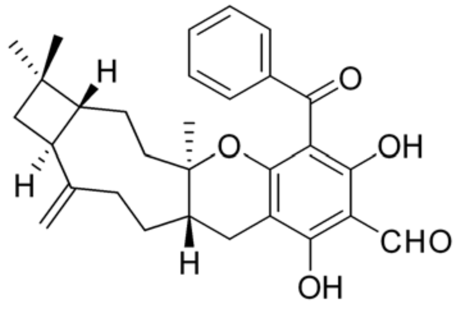

S7.44. UV spectrum of compound **5**

Spectrum Peak Pick Report 2015-04-08

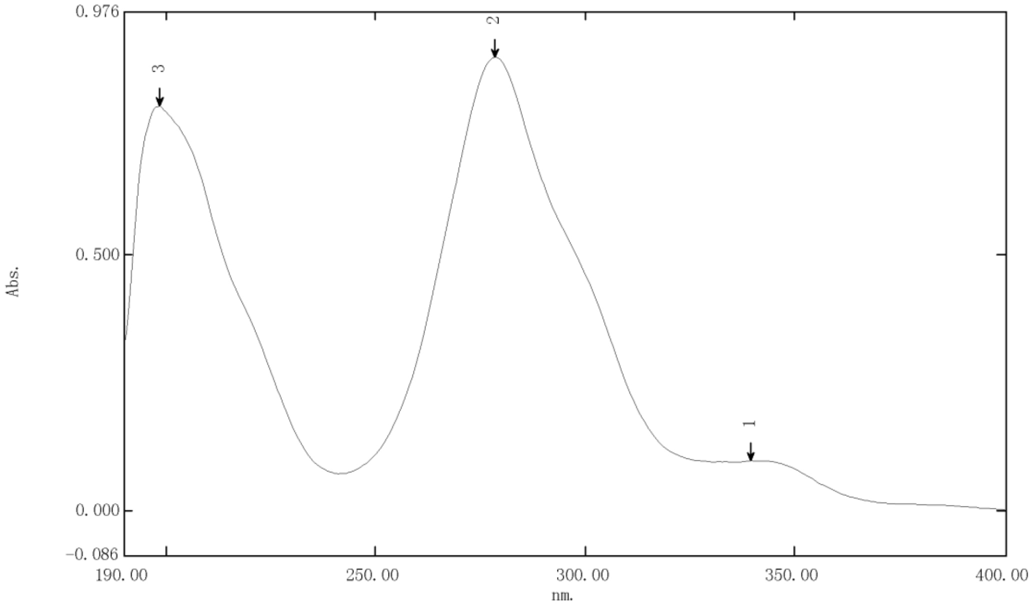

Measurement Properties
Wavelength Range (nm.): 190.00 to 400.00
Scan Speed: Medium
Sampling Interval: 2.0
Auto Sampling Interval: Disabled
Scan Mode: Auto

Sample Preparation Properties
Weight:
Volume:
Dilution:
Path Length:
Additional Information:

Instrument Properties
Instrument Type: UV-2400PC Series
Measuring Mode: Absorbance
Slit Width: 2.0 nm
Light Source Change Wavelength: 360.0 nm
S/R Exchange: Normal

No.	P/V	波长(nm)	吸收值
1	↑	339.50	0.098
2	↑	278.50	0.888
3	↑	198.50	0.793
4	↓	335.00	0.095
5	↓	241.00	0.073

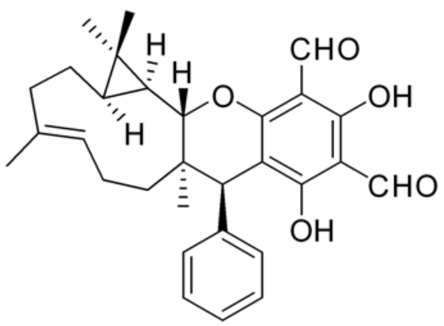

S7.45. UV spectrum of compound **6**

Spectrum Peak Pick Report 2015-08-01

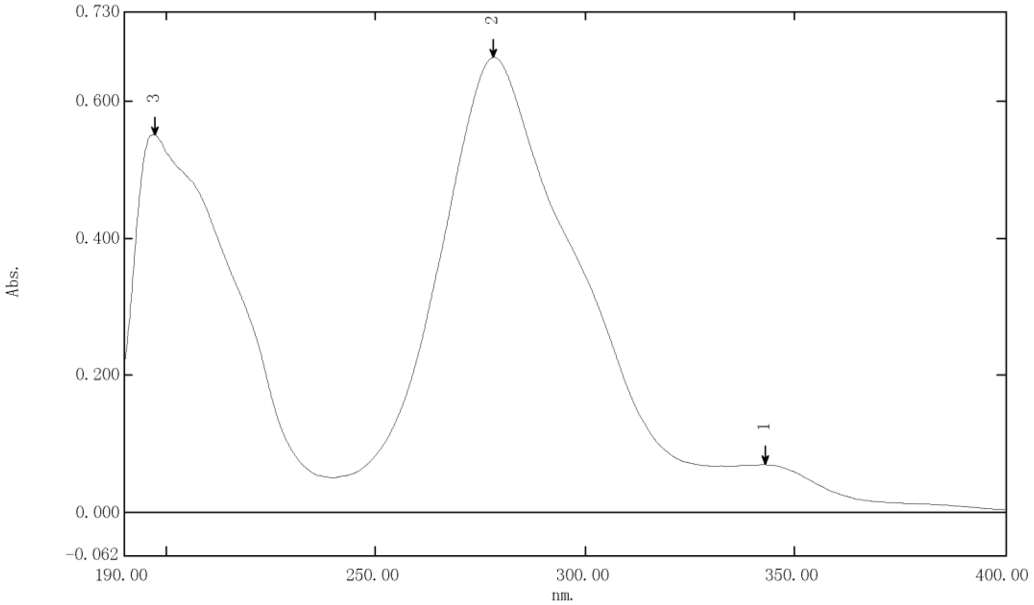

Measurement Properties
Wavelength Range (nm.): 190.00 to 400.00
Scan Speed: Medium
Sampling Interval: 2.0
Auto Sampling Interval: Disabled
Scan Mode: Auto

Sample Preparation Properties
Weight:
Volume:
Dilution:
Path Length:
Additional Information:

Instrument Properties
Instrument Type: UV-2400PC Series
Measuring Mode: Absorbance
Slit Width: 2.0 nm
Light Source Change Wavelength: 360.0 nm
S/R Exchange: Normal

No.	P/V	波长(nm)	吸收值
1	↑	342. 70	0. 070
2	↑	278. 00	0. 664
3	↑	197. 50	0. 552
4	↓	239. 80	0. 051

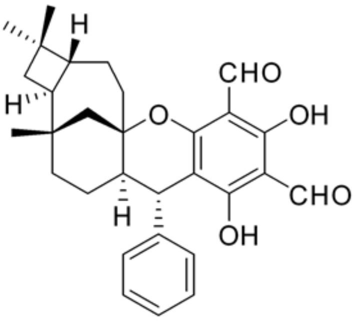

S7.46. UV spectrum of compound **7**

Spectrum Peak Pick Report 2015-04-08

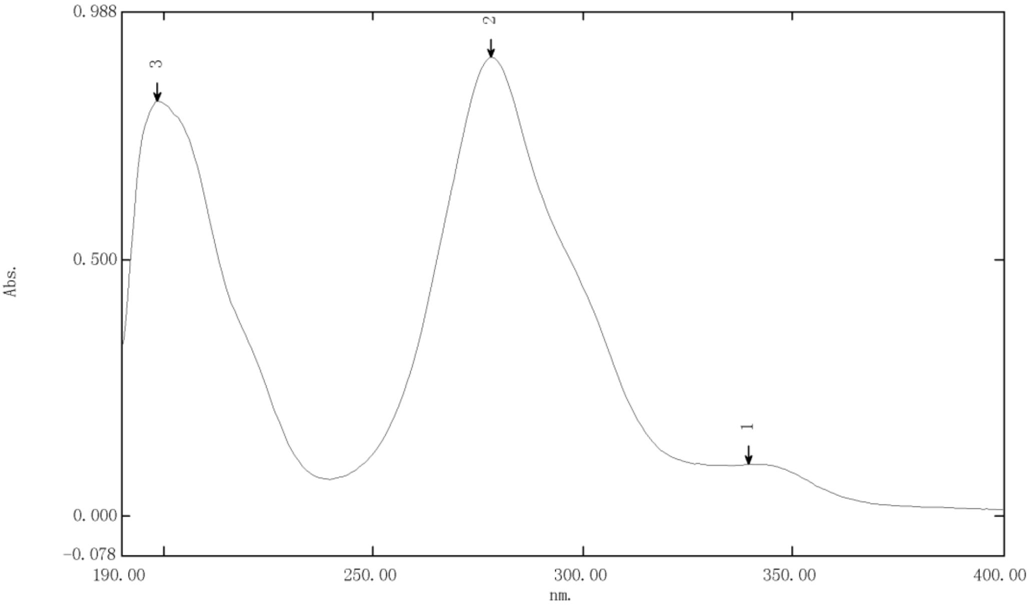

Measurement Properties
Wavelength Range (nm.): 190.00 to 400.00
Scan Speed: Medium
Sampling Interval: 2.0
Auto Sampling Interval: Disabled
Scan Mode: Auto

Sample Preparation Properties
Weight:
Volume:
Dilution:
Path Length:
Additional Information:

Instrument Properties
Instrument Type: UV-2400PC Series
Measuring Mode: Absorbance
Slit Width: 2.0 nm
Light Source Change Wavelength: 360.0 nm
S/R Exchange: Normal

No.	P/V	波长(nm)	吸收值
1	↑	339.50	0.100
2	↑	278.00	0.899
3	↑	198.50	0.814
4	↓	335.00	0.097
5	↓	239.50	0.070

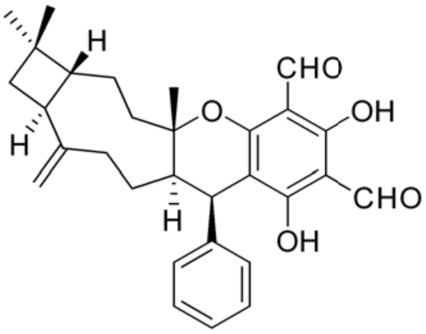

S7.47. UV spectrum of compound **9**

Spectrum Peak Pick Report 2015-08-01

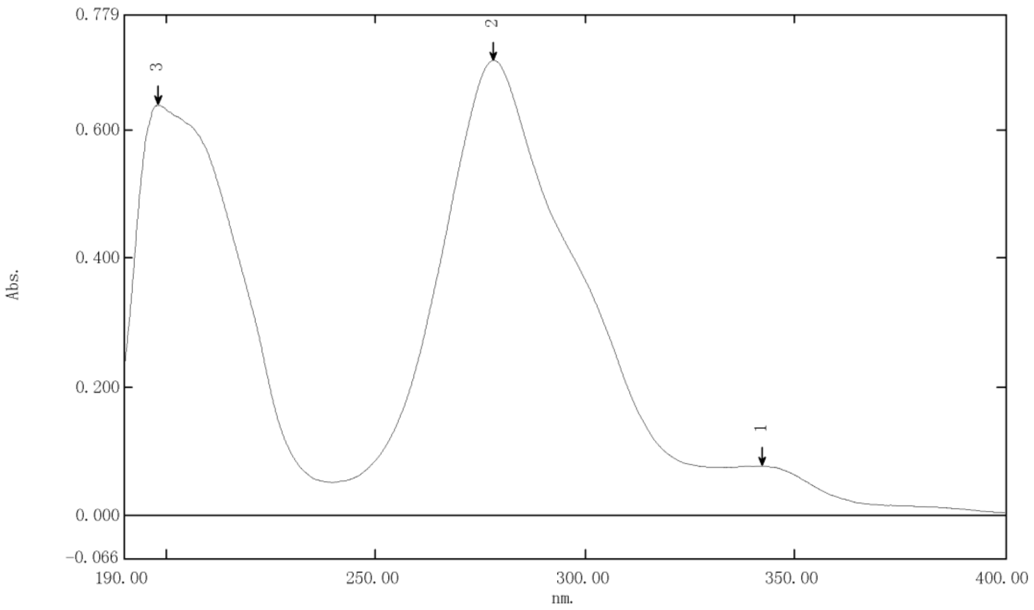

Measurement Properties
Wavelength Range (nm.): 190.00 to 400.00
Scan Speed: Medium
Sampling Interval: 2.0
Auto Sampling Interval: Disabled
Scan Mode: Auto

Sample Preparation Properties
Weight:
Volume:
Dilution:
Path Length:
Additional Information:

Instrument Properties
Instrument Type: UV-2400PC Series
Measuring Mode: Absorbance
Slit Width: 2.0 nm
Light Source Change Wavelength: 360.0 nm
S/R Exchange: Normal

No.	P/V	波长(nm)	吸收值
1	↑	342.20	0.077
2	↑	278.10	0.708
3	↑	198.00	0.639
4	↓	239.60	0.051

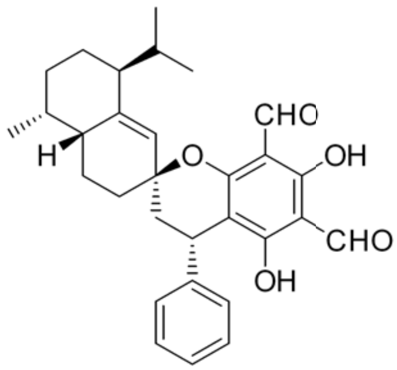

S7.48. UV spectrum of compound **9**

Spectrum Peak Pick Report 2015-08-01

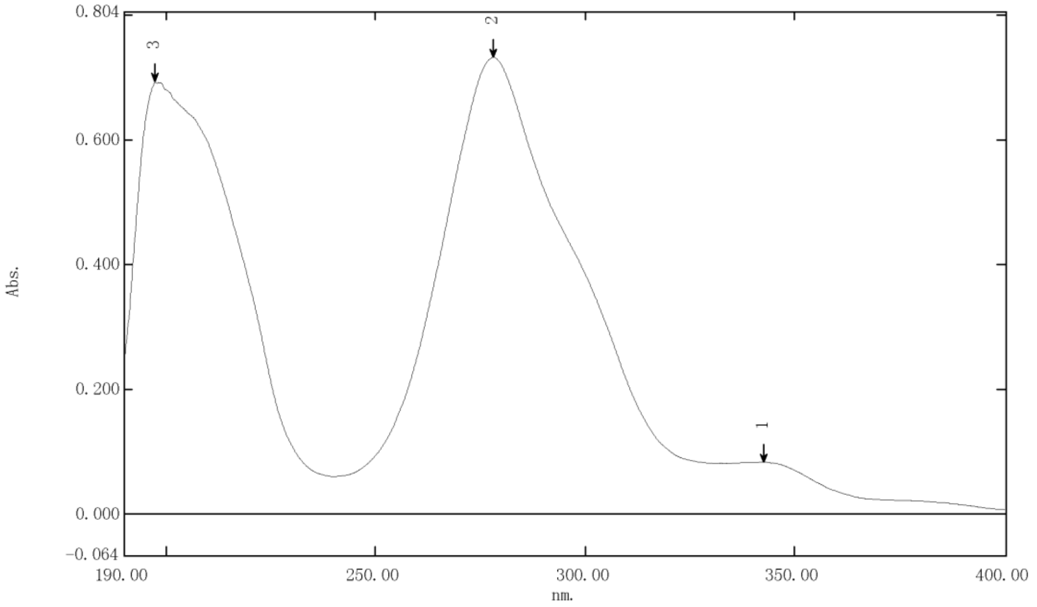

Measurement Properties
Wavelength Range (nm.): 190.00 to 400.00
Scan Speed: Medium
Sampling Interval: 2.0
Auto Sampling Interval: Disabled
Scan Mode: Auto

Sample Preparation Properties
Weight:
Volume:
Dilution:
Path Length:
Additional Information:

Instrument Properties
Instrument Type: UV-2400PC Series
Measuring Mode: Absorbance
Slit Width: 2.0 nm
Light Source Change Wavelength: 360.0 nm
S/R Exchange: Normal

No.	P/V	波长(nm)	吸收值
1	↑	342.40	0.085
2	↑	278.00	0.732
3	↑	197.30	0.692
4	↓	334.30	0.082
5	↓	239.90	0.061

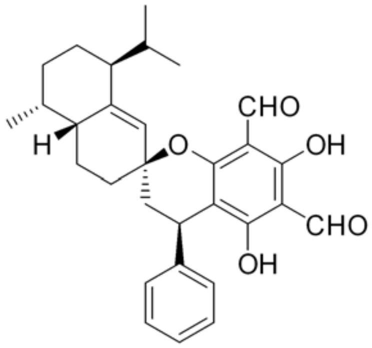

S7.49. UV spectrum of compound **10**

Spectrum Peak Pick Report

2015-08-01

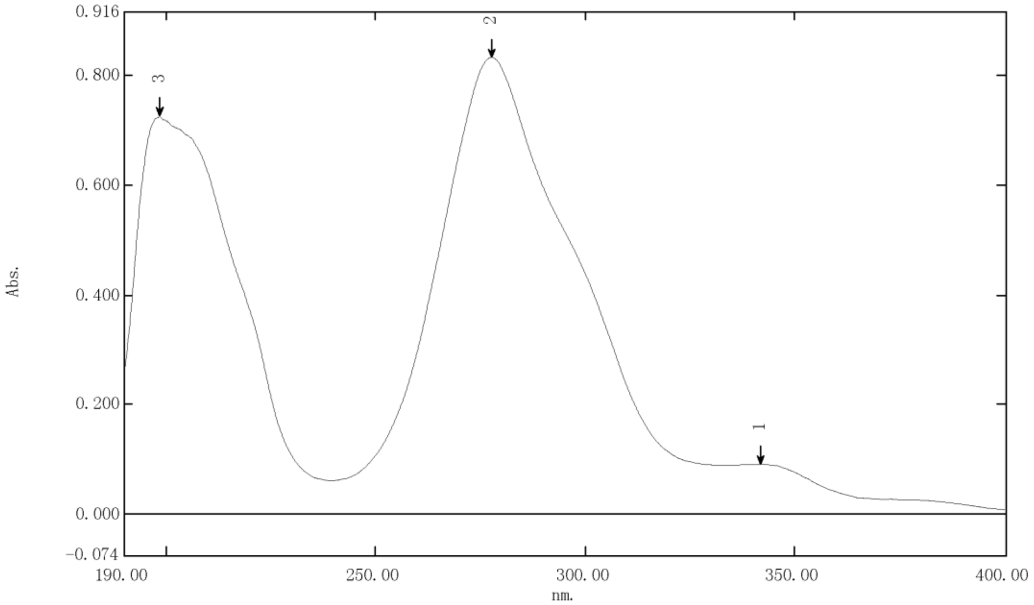

Measurement Properties	
Wavelength Range (nm.):	190.00 to 400.00
Scan Speed:	Medium
Sampling Interval:	2.0
Auto Sampling Interval:	Disabled
Scan Mode:	Auto
Sample Preparation Properties	
Weight:	
Volume:	
Dilution:	
Path Length:	
Additional Information:	
Instrument Properties	
Instrument Type:	UV-2400PC Series
Measuring Mode:	Absorbance
Slit Width:	2.0 nm
Light Source Change Wavelength:	360.0 nm
S/R Exchange:	Normal

No.	P/V	波长(nm)	吸收值
1	↑	341. 60	0. 091
2	↑	277. 60	0. 833
3	↑	198. 40	0. 725
4	↓	239. 70	0. 061

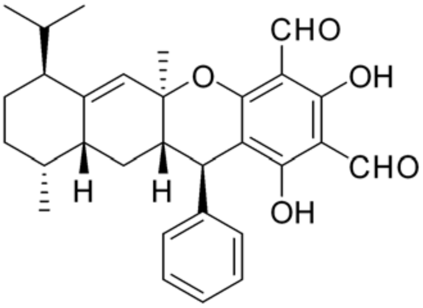

S7.50. UV spectrum of compound **11**

Spectrum Peak Pick Report

2014-11-27

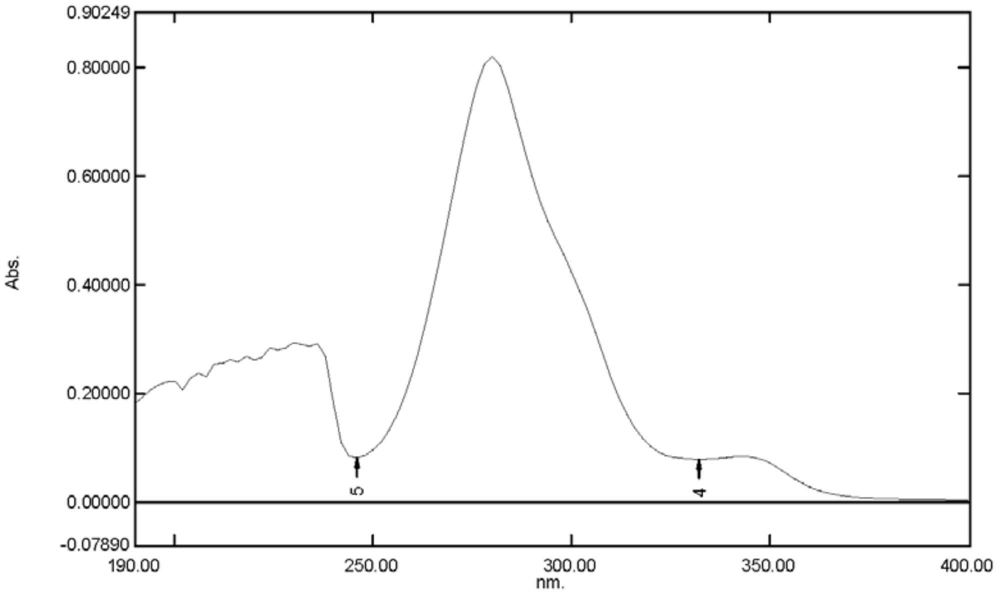

Measurement Properties
Wavelength Range (nm.): 190.00 to 400.00
Scan Speed: Medium
Sampling Interval: 2.0
Auto Sampling Interval: Disabled
Scan Mode: Auto

No.	P/V	Wavelength	Abs.
1	↑	342.00	0.08449
2	↑	280.00	0.82071
3	↑	230.00	0.29338
4	↓	332.00	0.07806
5	↓	246.00	0.08171

Sample Preparation Properties
Weight:
Volume:
Dilution:
Path Length:
Additional Information:

Instrument Properties
Instrument Type: UV-2400PC Series
Measuring Mode: Absorbance
Slit Width: 2.0 nm
Light Source Change Wavelength: 360.0 nm
S/R Exchange: Normal

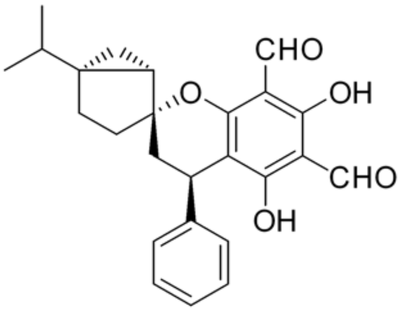

S7.51. UV spectrum of compound **12**

Spectrum Peak Pick Report 2015-08-01

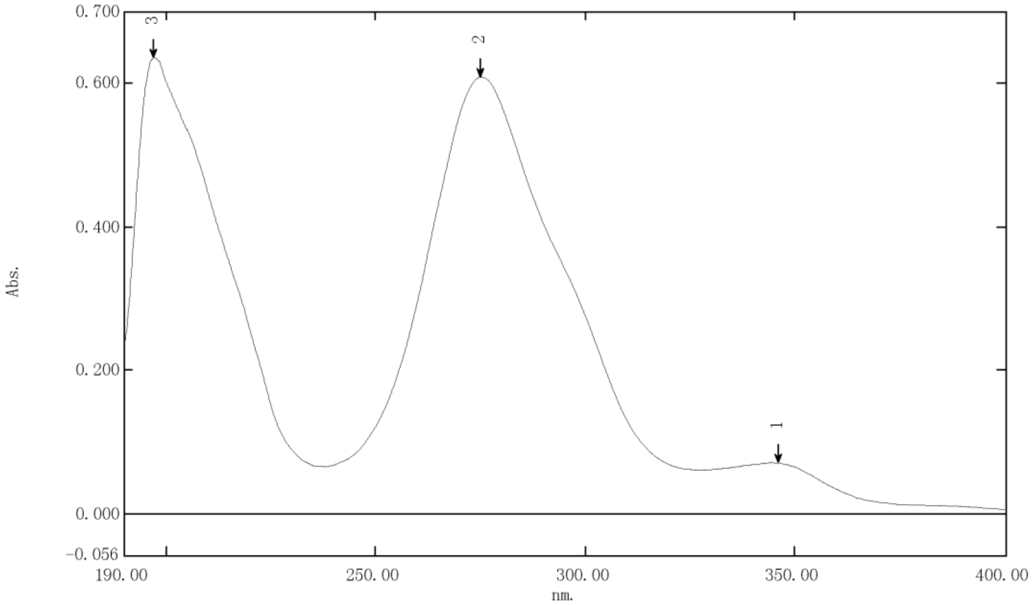

Measurement Properties	
Wavelength Range (nm.):	190.00 to 400.00
Scan Speed:	Medium
Sampling Interval:	2.0
Auto Sampling Interval:	Disabled
Scan Mode:	Auto
Sample Preparation Properties	
Weight:	
Volume:	
Dilution:	
Path Length:	
Additional Information:	
Instrument Properties	
Instrument Type:	UV-2400PC Series
Measuring Mode:	Absorbance
Slit Width:	2.0 nm
Light Source Change Wavelength:	360.0 nm
S/R Exchange:	Normal

No.	P/V	波长(nm)	吸收值
1	↑	346. 00	0. 072
2	↑	275. 00	0. 609
3	↑	197. 10	0. 637
4	↓	327. 80	0. 061
5	↓	238. 00	0. 067

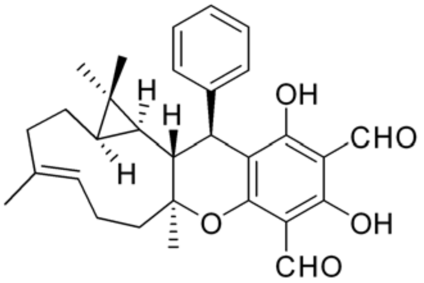

S7.52. UV spectrum of compound **25**

Spectrum Peak Pick Report

2014-11-26

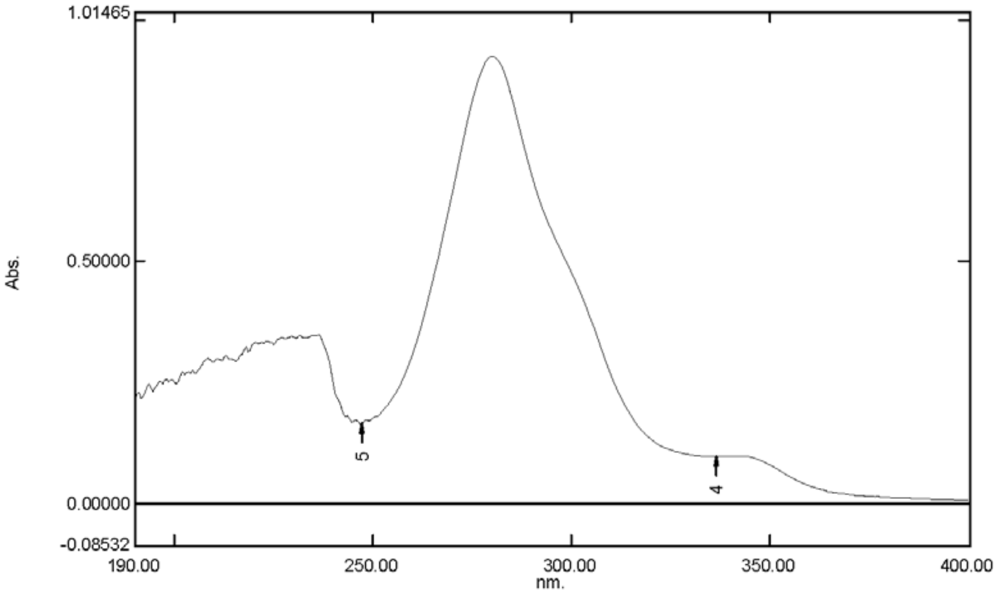

Measurement Properties
Wavelength Range (nm.): 190.00 to 400.00
Scan Speed: Fast
Sampling Interval: 0.5
Auto Sampling Interval: Disabled
Scan Mode: Auto

No.	P/V	Wavelength	Abs.
1	↑	342.00	0.09793
2	↑	280.00	0.92299
3	↑	236.50	0.34818
4	↓	336.50	0.09654
5	↓	247.00	0.16396

Sample Preparation Properties
Weight:
Volume:
Dilution:
Path Length:
Additional Information:

Instrument Properties
Instrument Type: UV-2400PC Series
Measuring Mode: Absorbance
Slit Width: 2.0 nm
Light Source Change Wavelength: 360.0 nm
S/R Exchange: Normal

Attachment Properties
Attachment: None

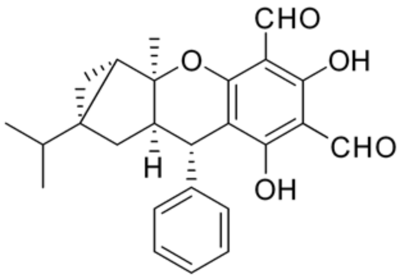

S7.53. ECD spectrum of compound **1**

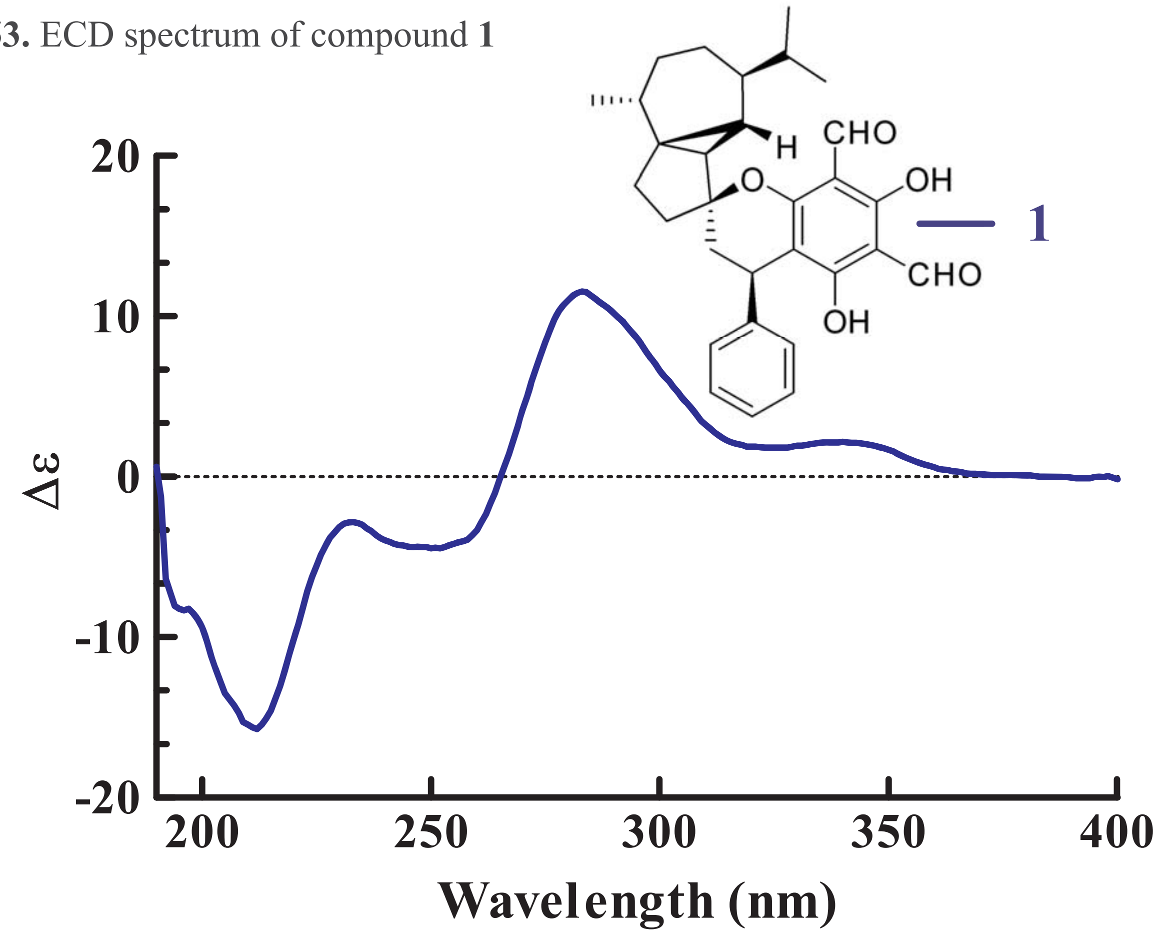

S7.54. ECD spectrum of compound **2**

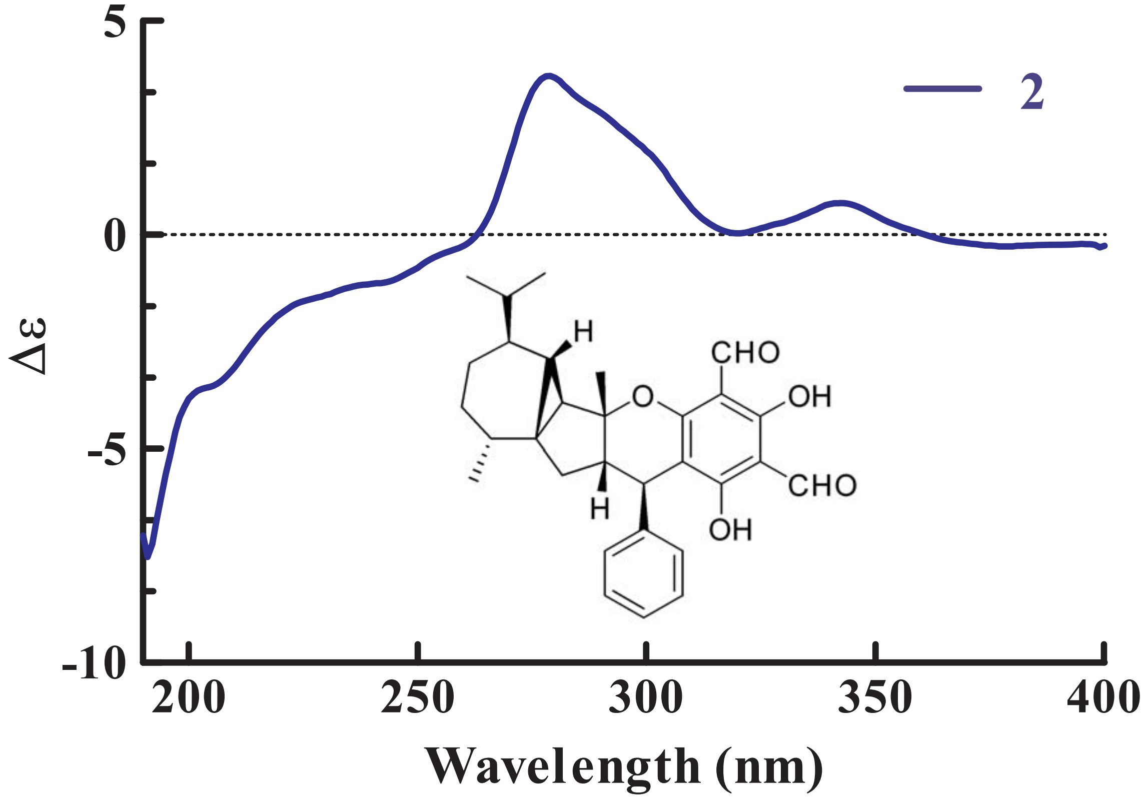

S7.55. ECD spectrum of compound **3**

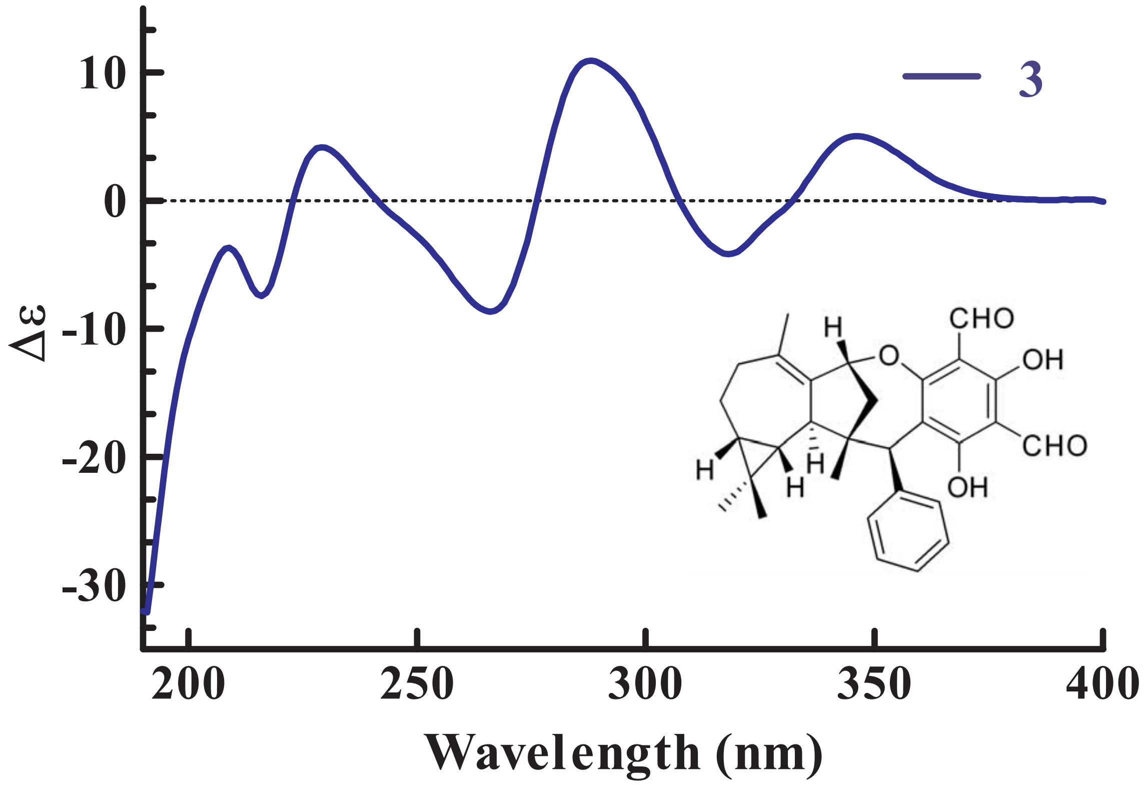

S7.56. ECD spectrum of compound **4**

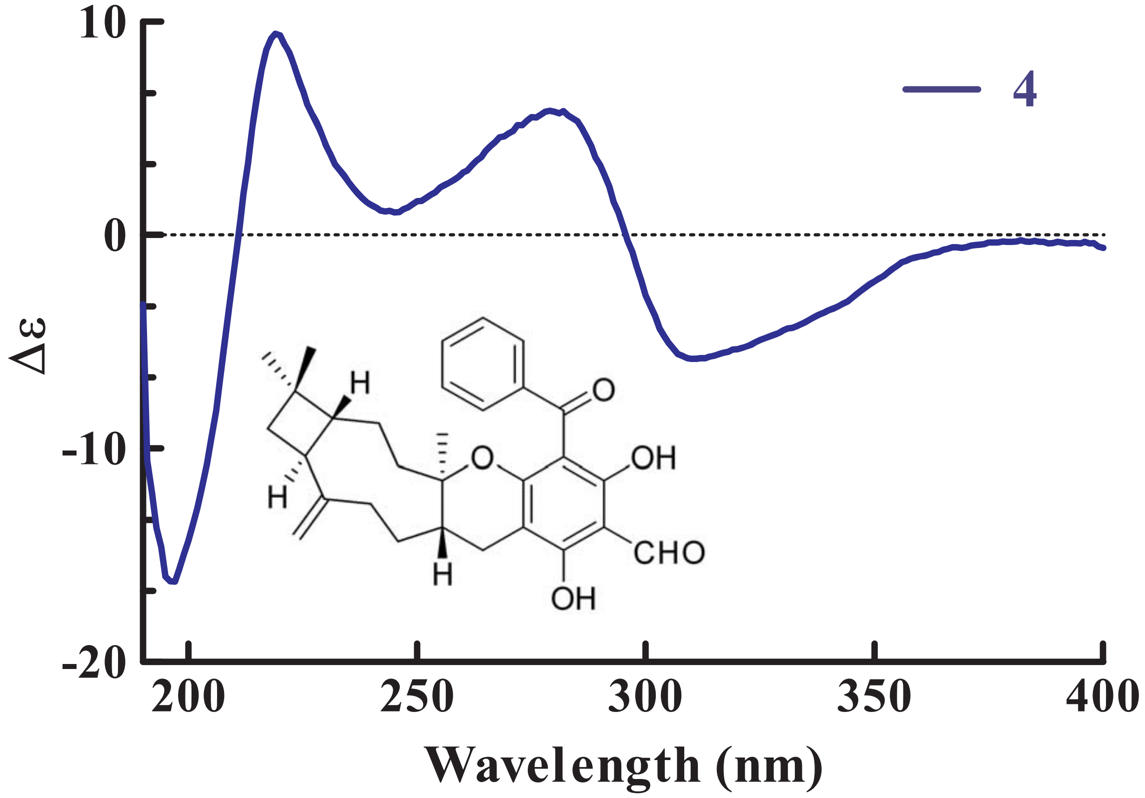

S7.57. ECD spectrum of compound **5**

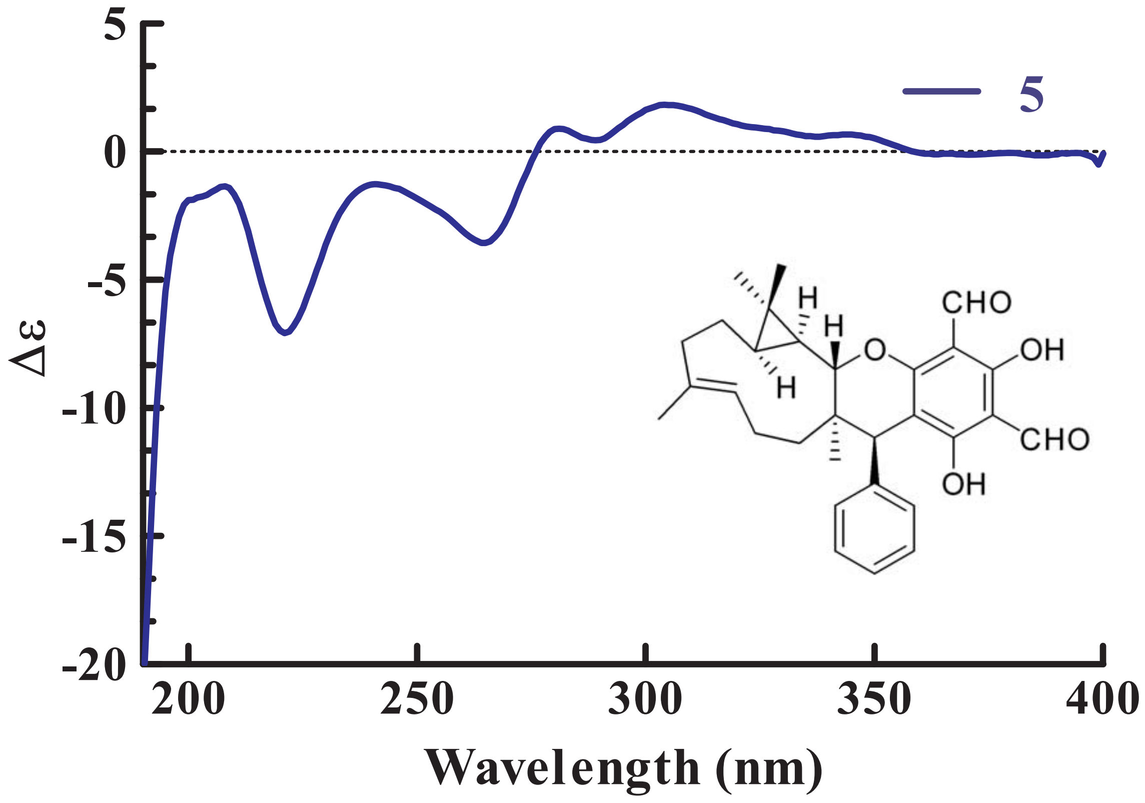

S7.58. ECD spectrum of compound **6**

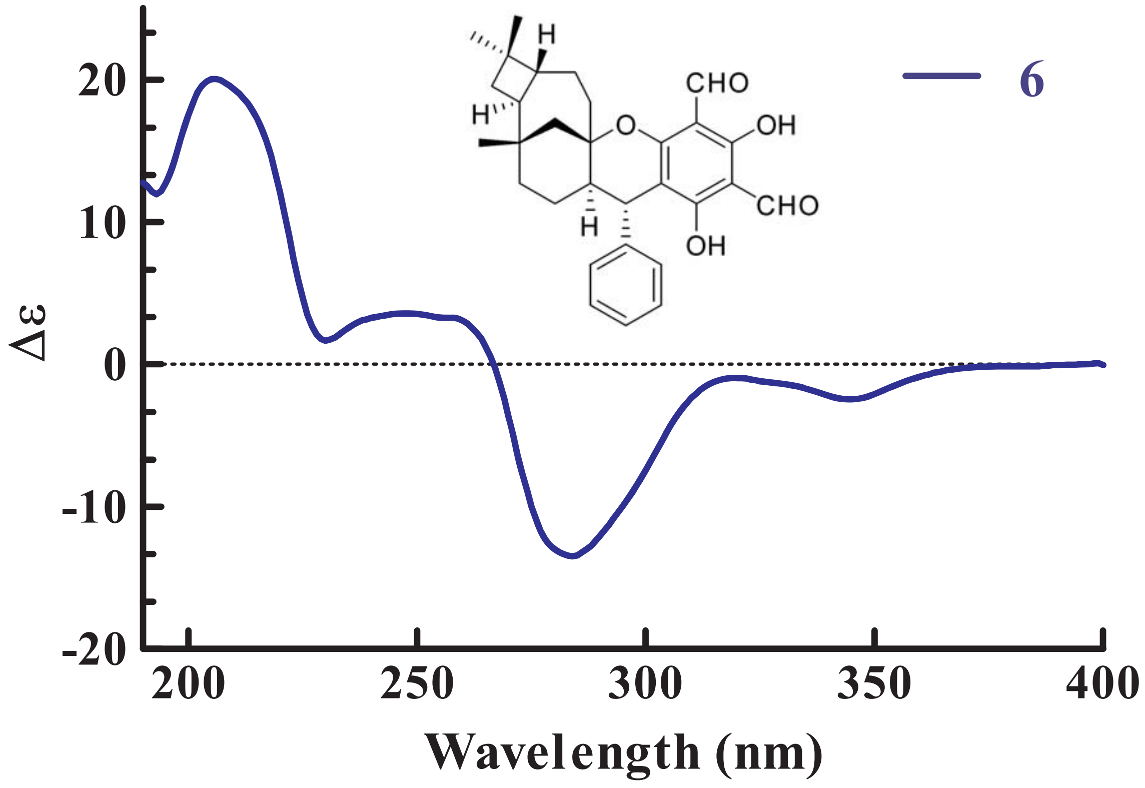

S7.59. ECD spectrum of compound **7**

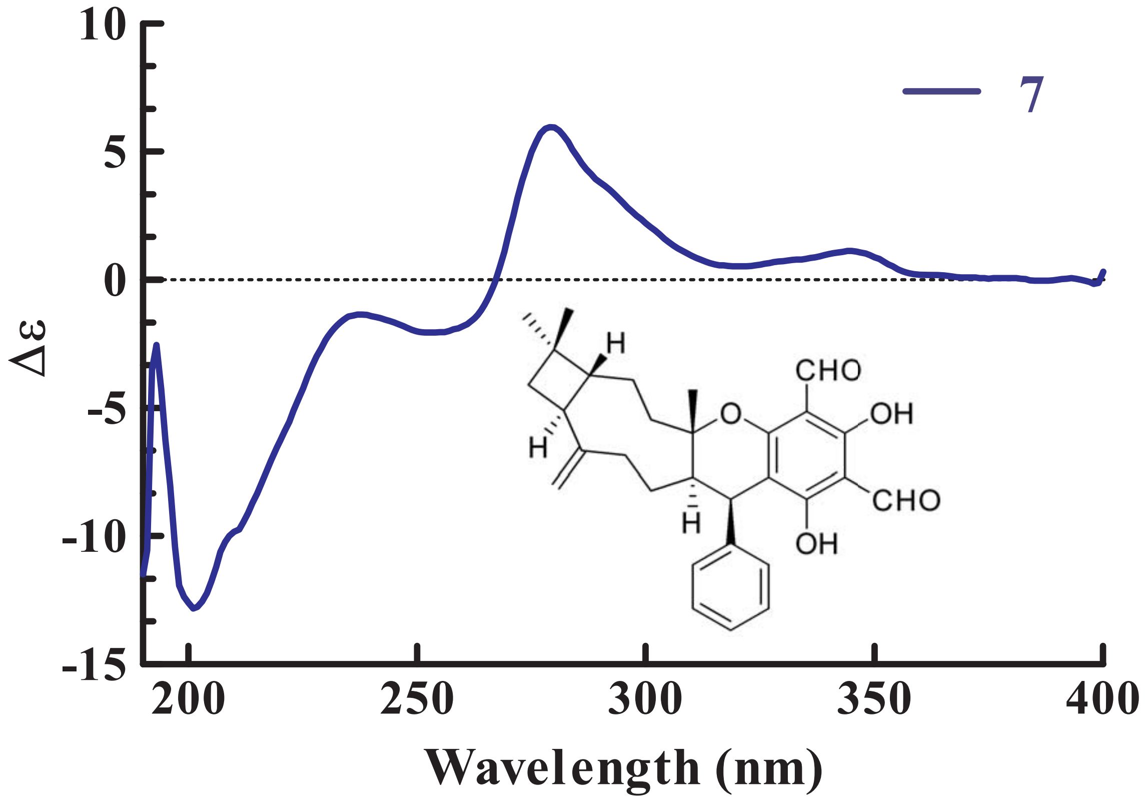

S7.60. ECD spectrum of compound **8**

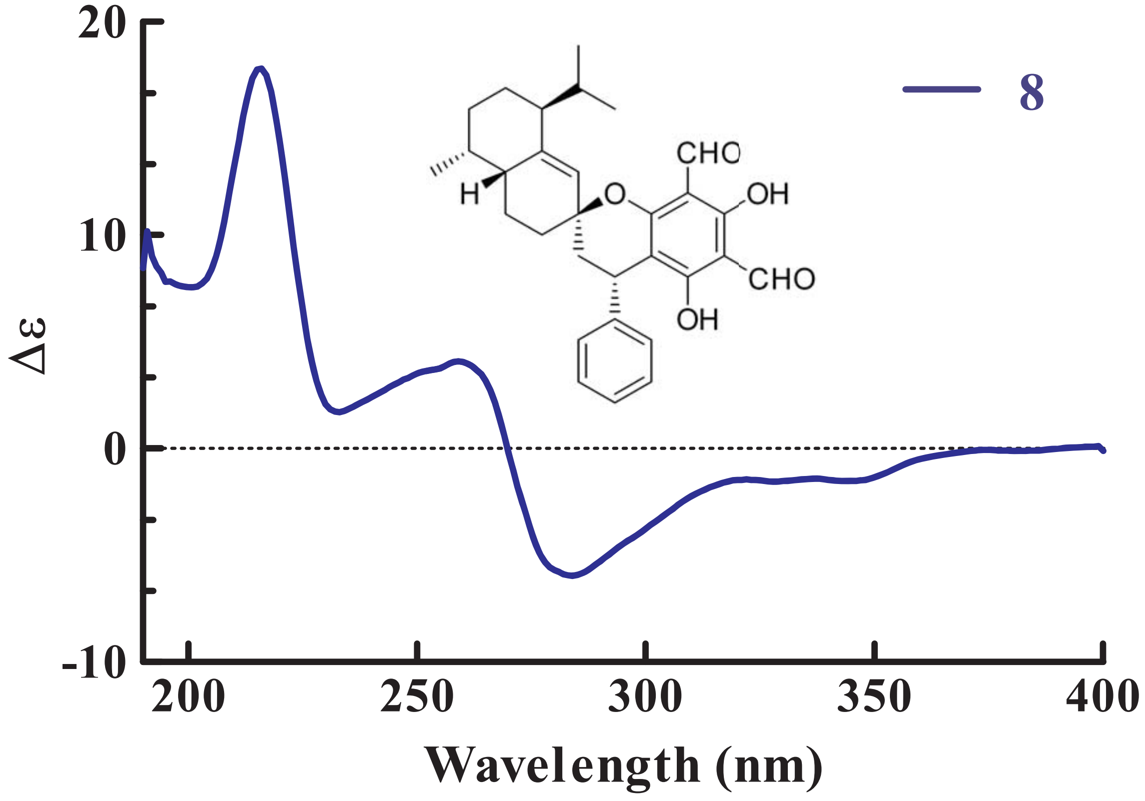

S7.61. ECD spectrum of compound **9**

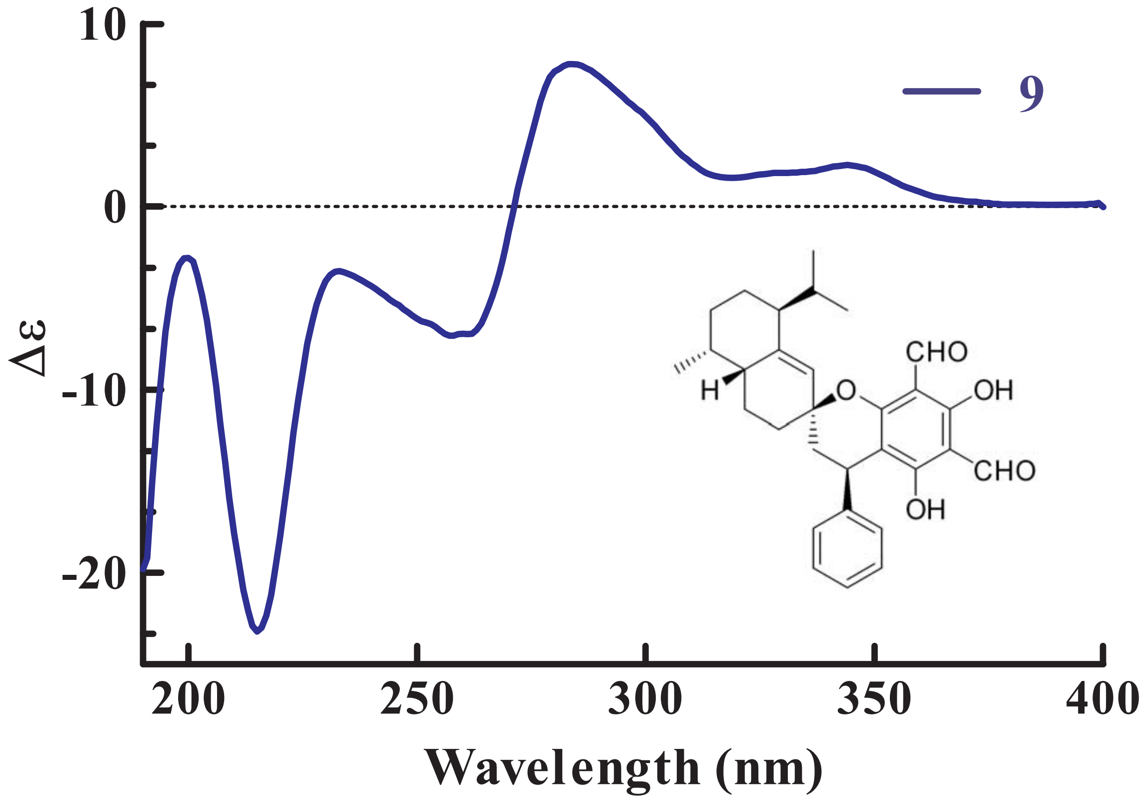

S7.62. ECD spectrum of compound **10**

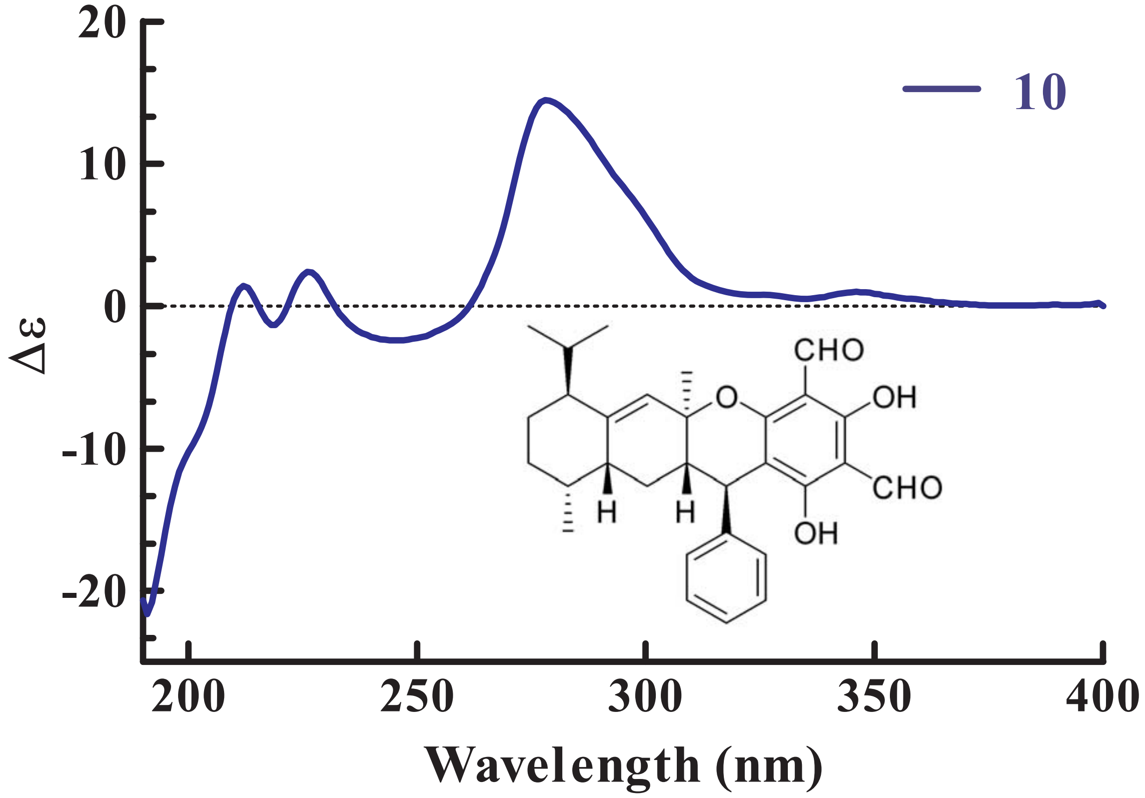

S7.63 ECD spectrum of compound **11**

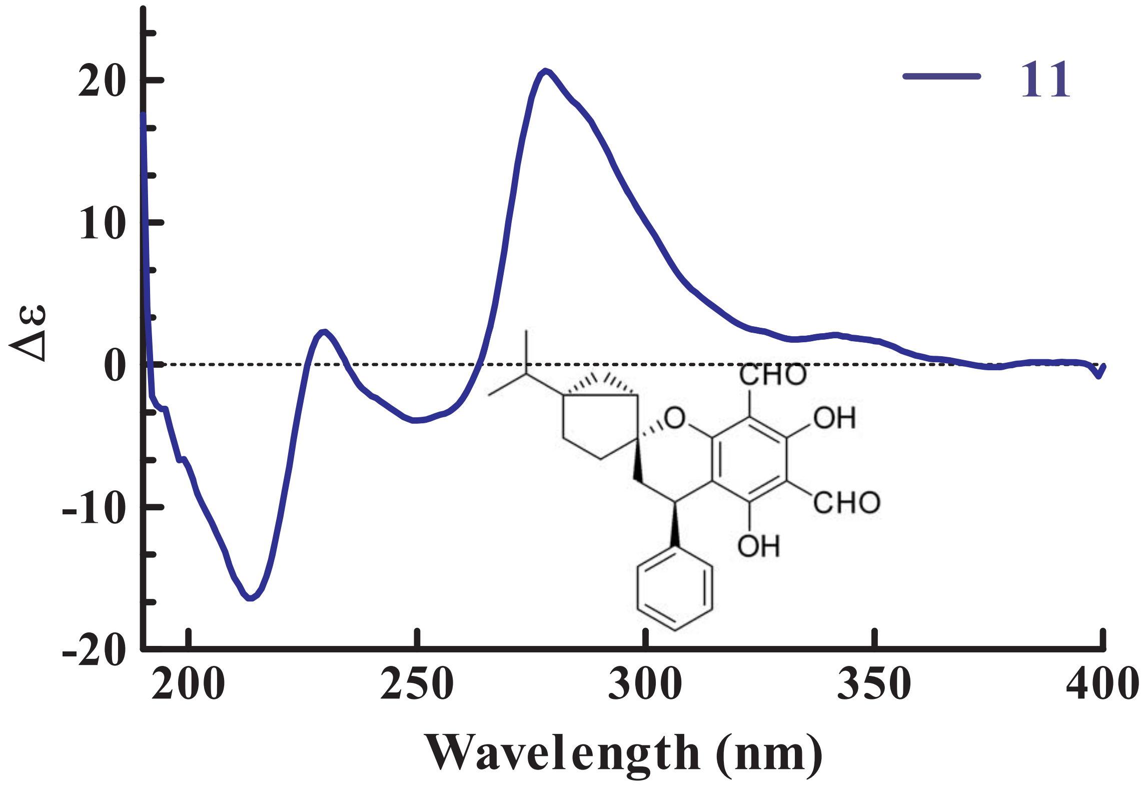

S7.64 ECD spectrum of compound **12**

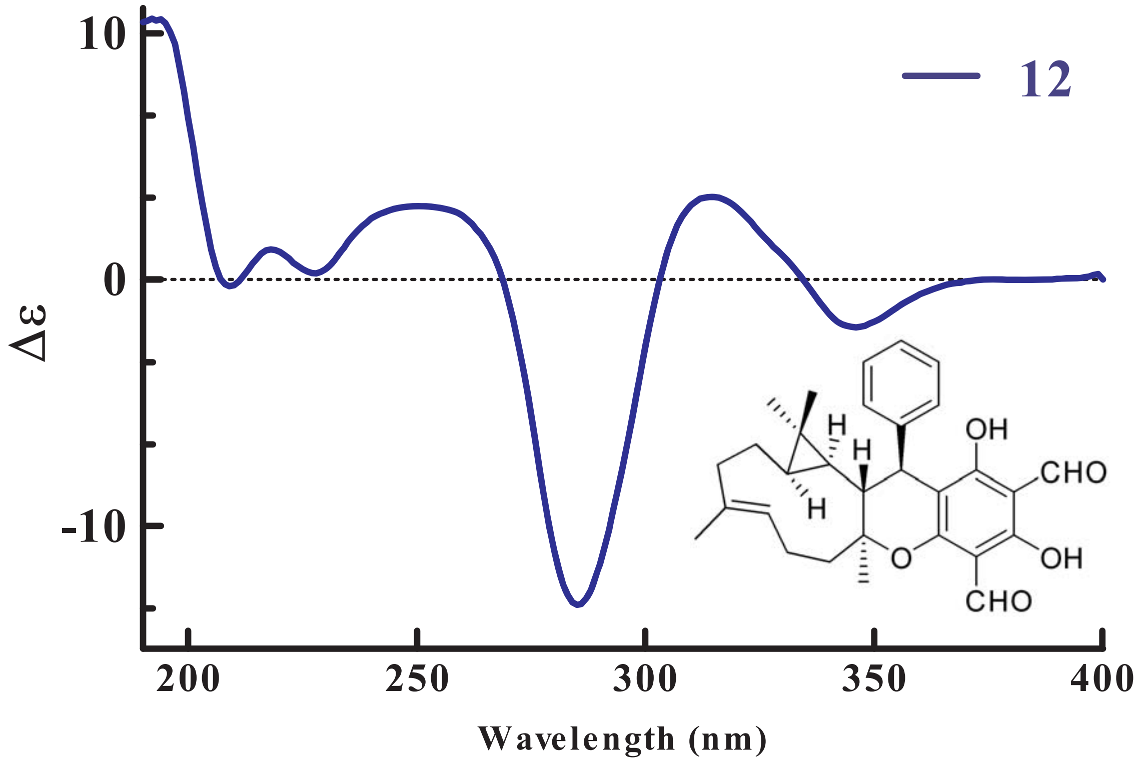

S7.65. ECD spectrum of compound **14**

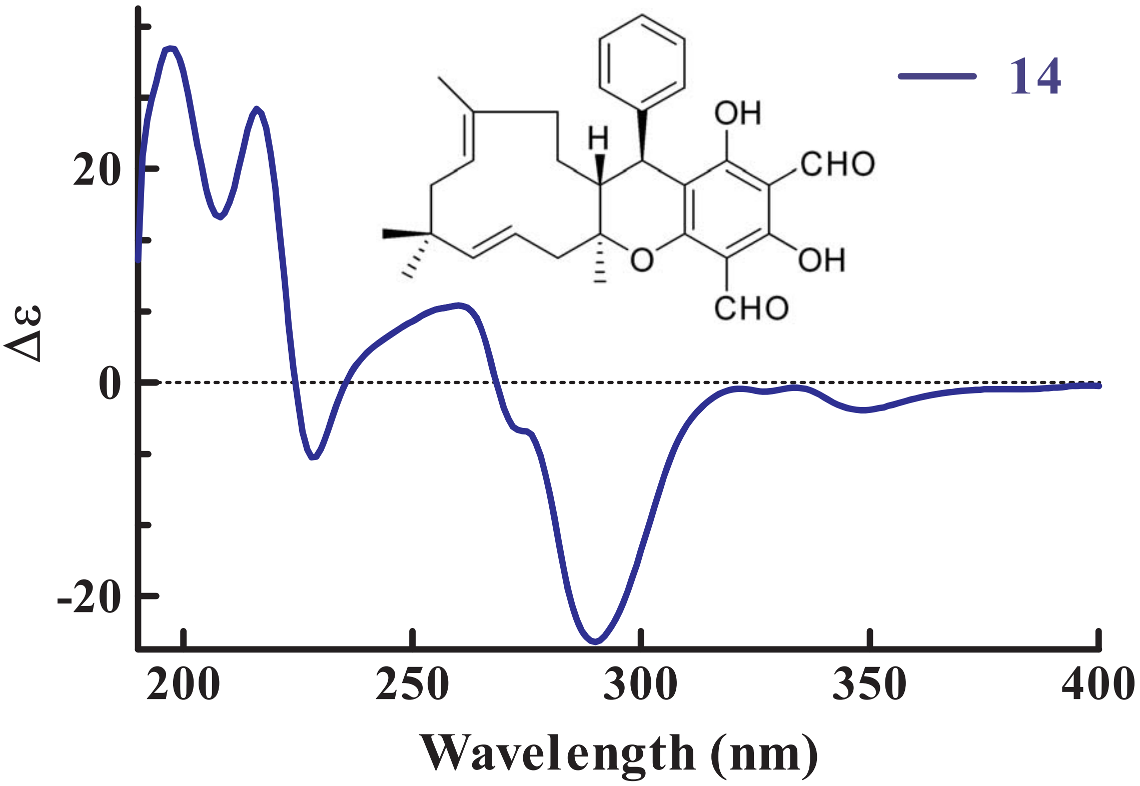

S7.66. ECD spectrum of compound **15**

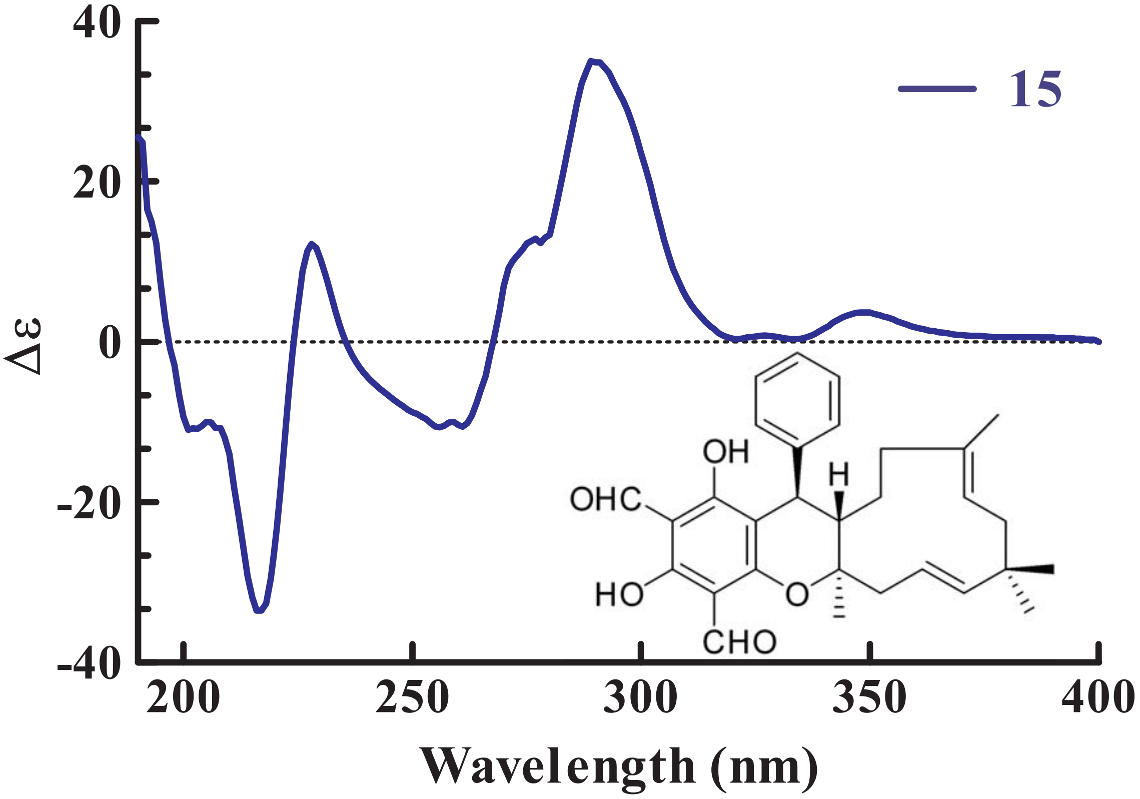

S7.67. ECD spectrum of compound **20**

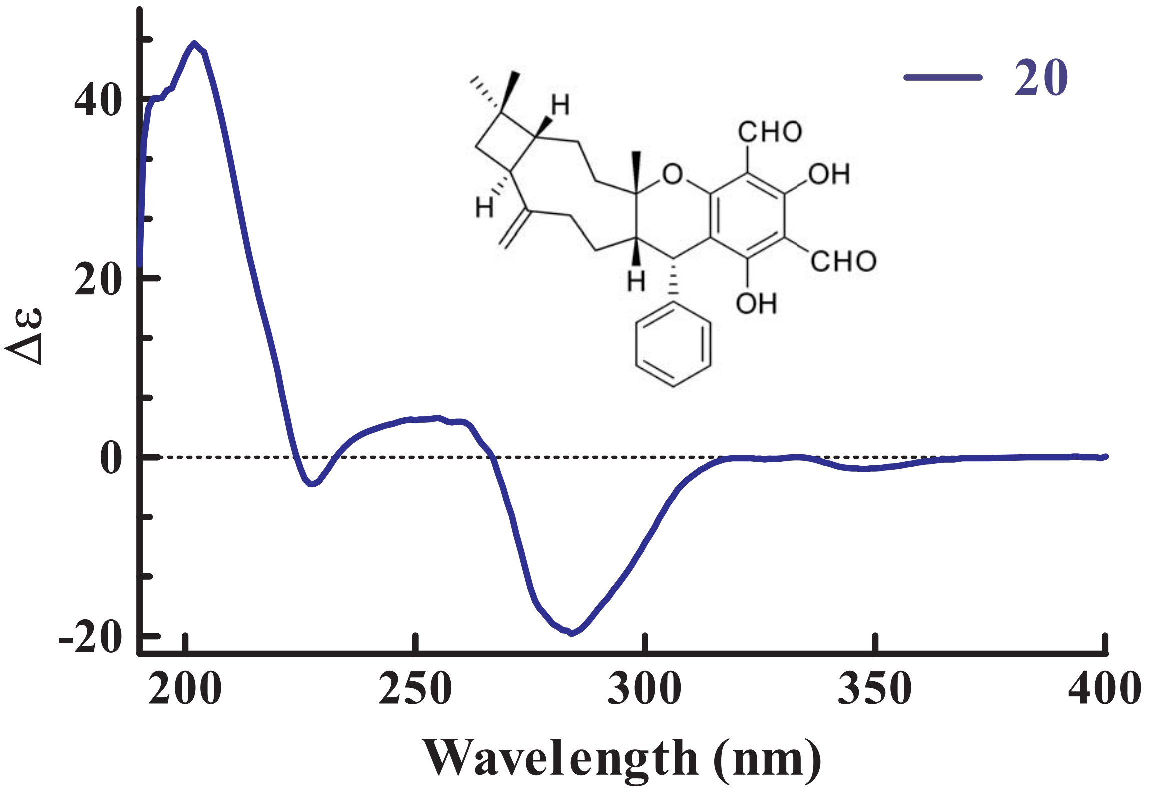

S7.68. ECD spectrum of compound **21**

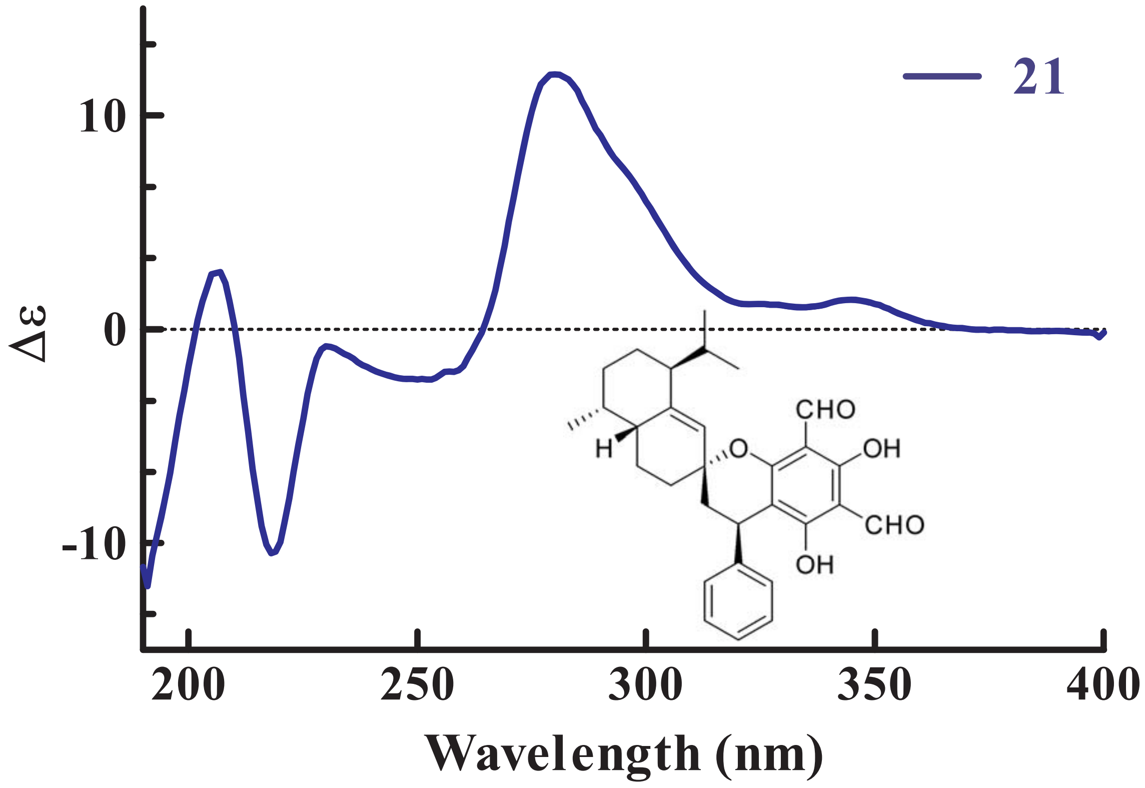

S7.69. ECD spectrum of compound **22**

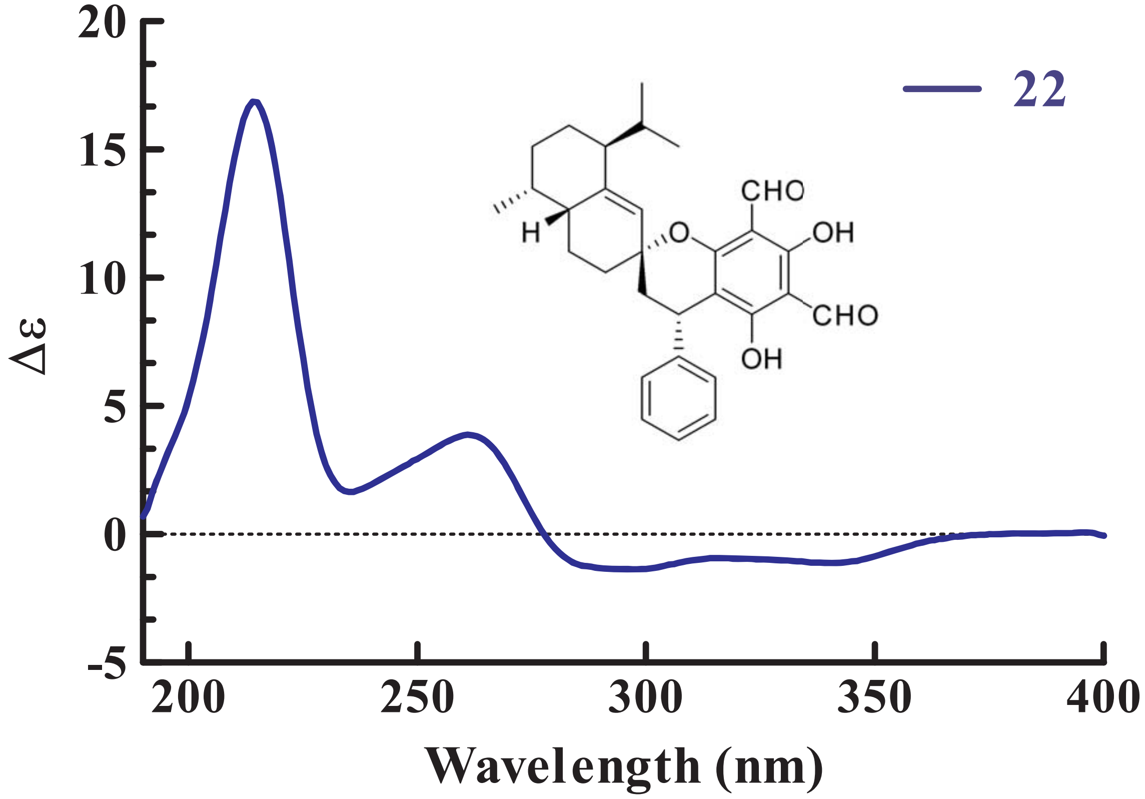

S7.70. ECD spectrum of compound **23**

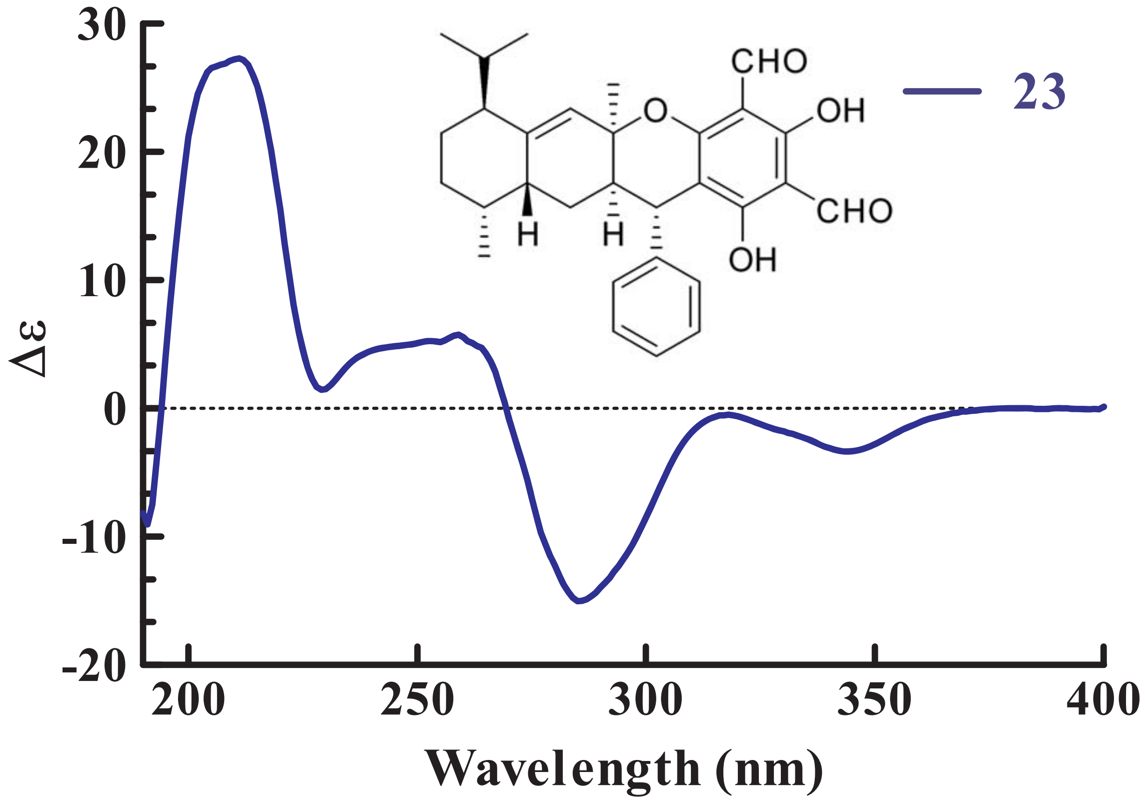

S7.71. ECD spectrum of compound **24**

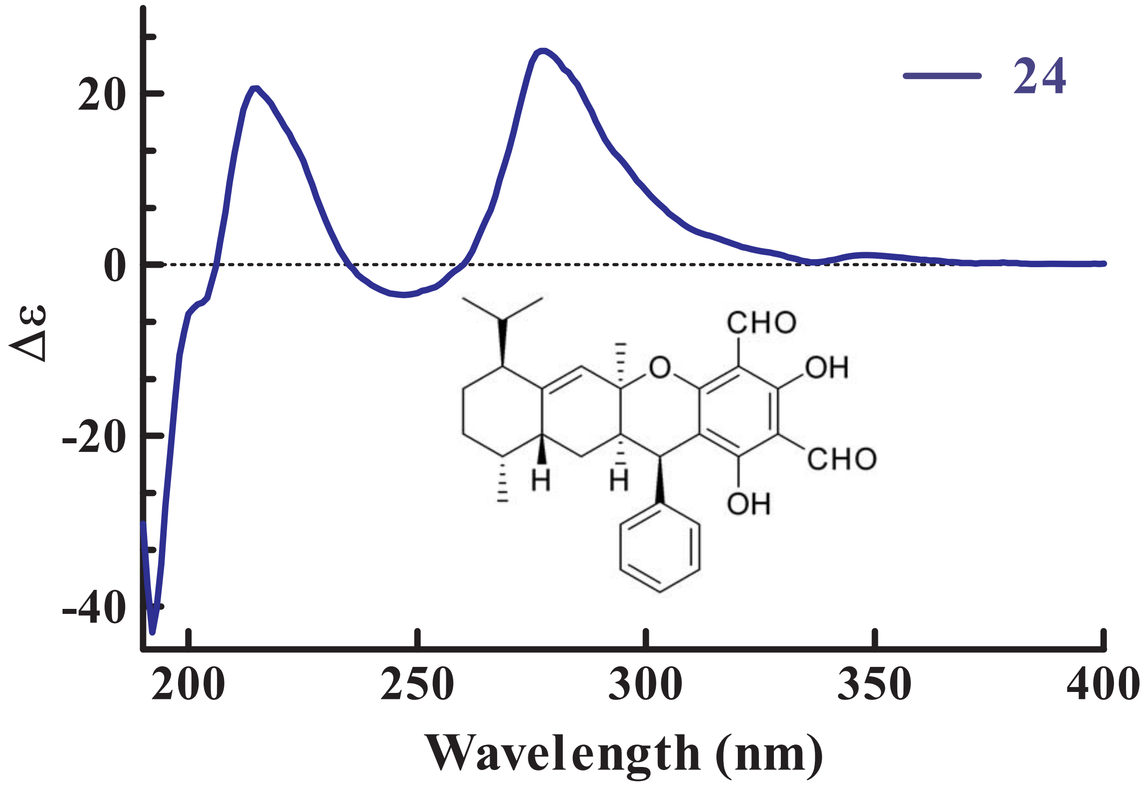

S7.72. ECD spectrum of compound **25**

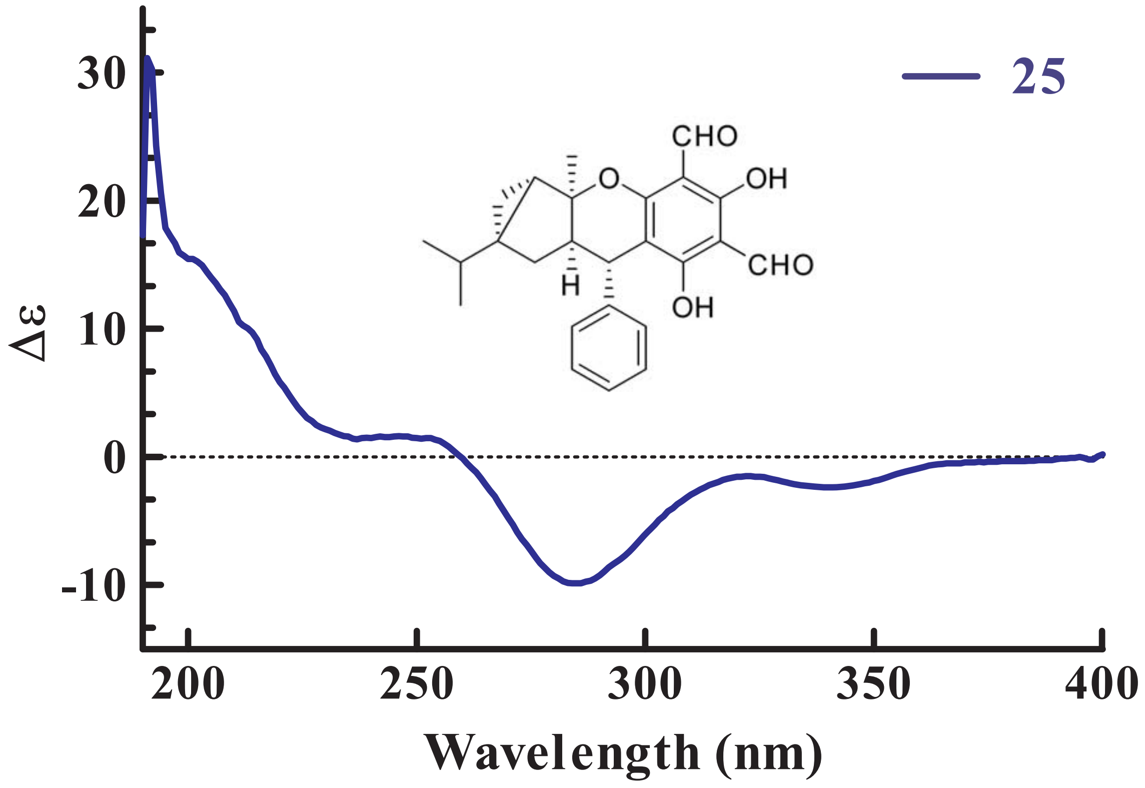

S8.1. ^{1}H NMR spectrum of compound **12**

In CDCl3

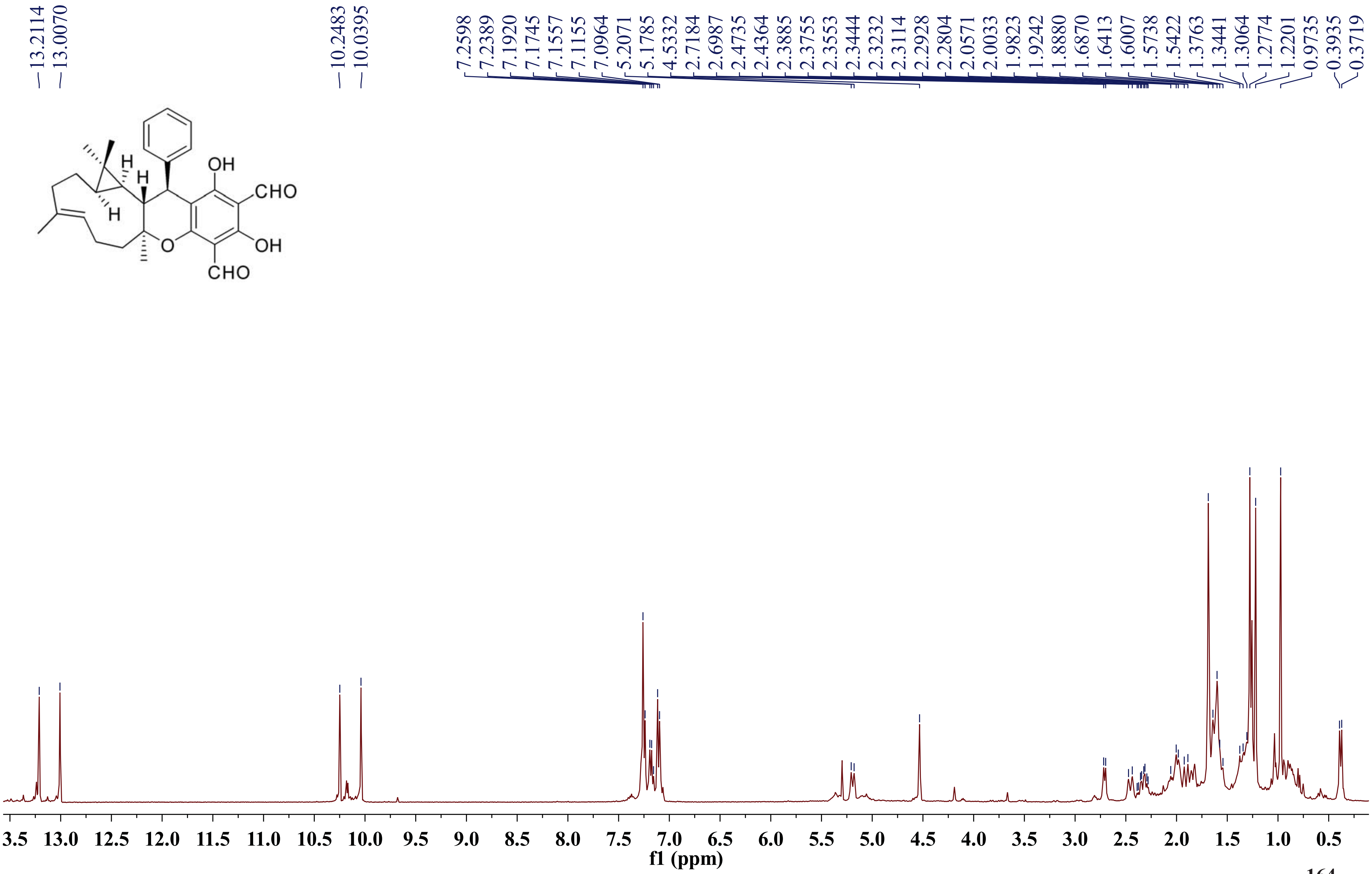

S8.2. DEPT spectra of compound **12**

In CDCl3

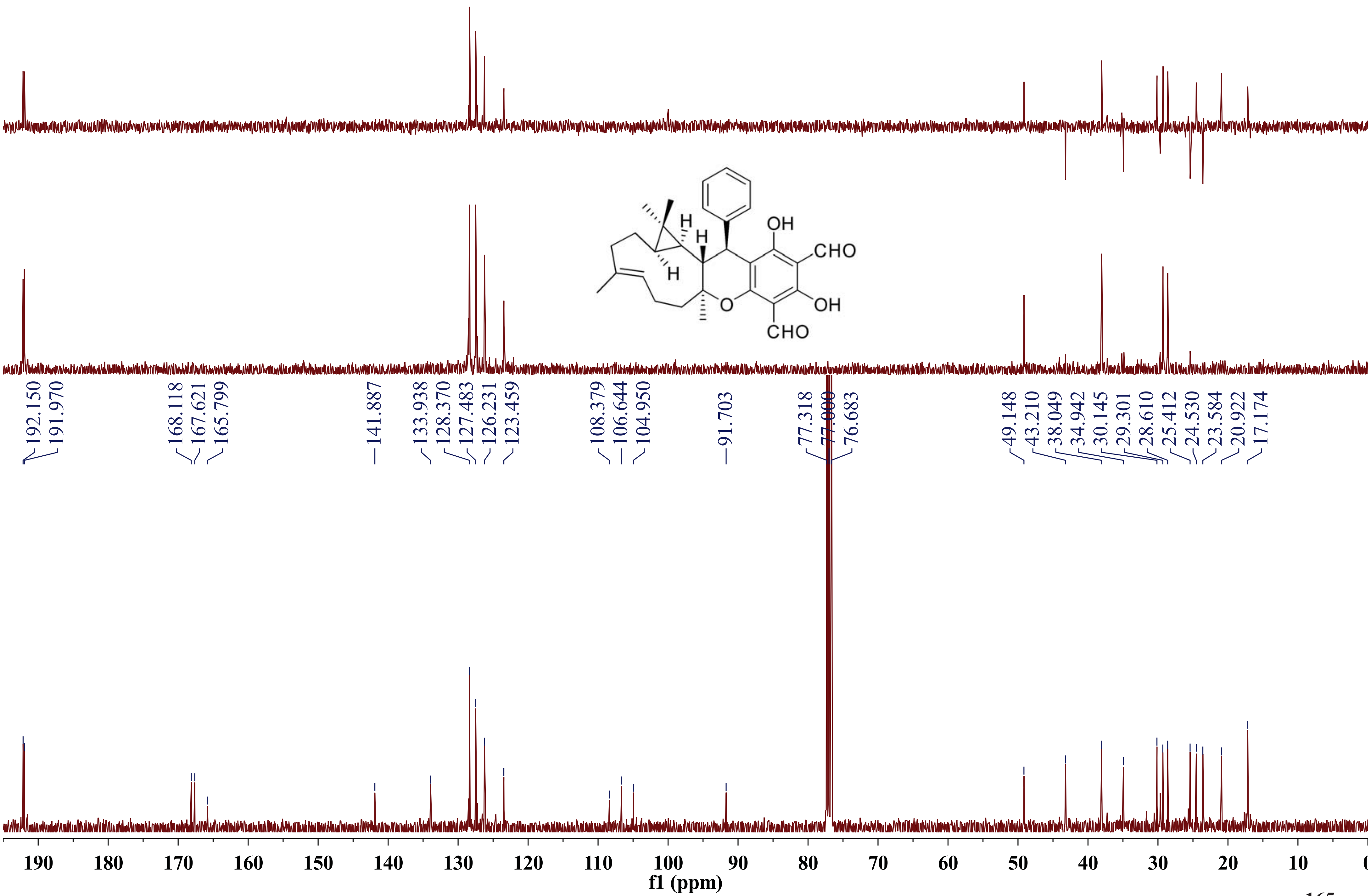

S8.3. HSQC spectrum of compound **12**

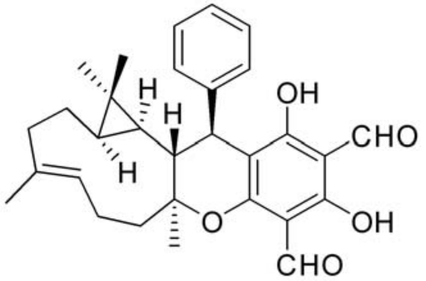

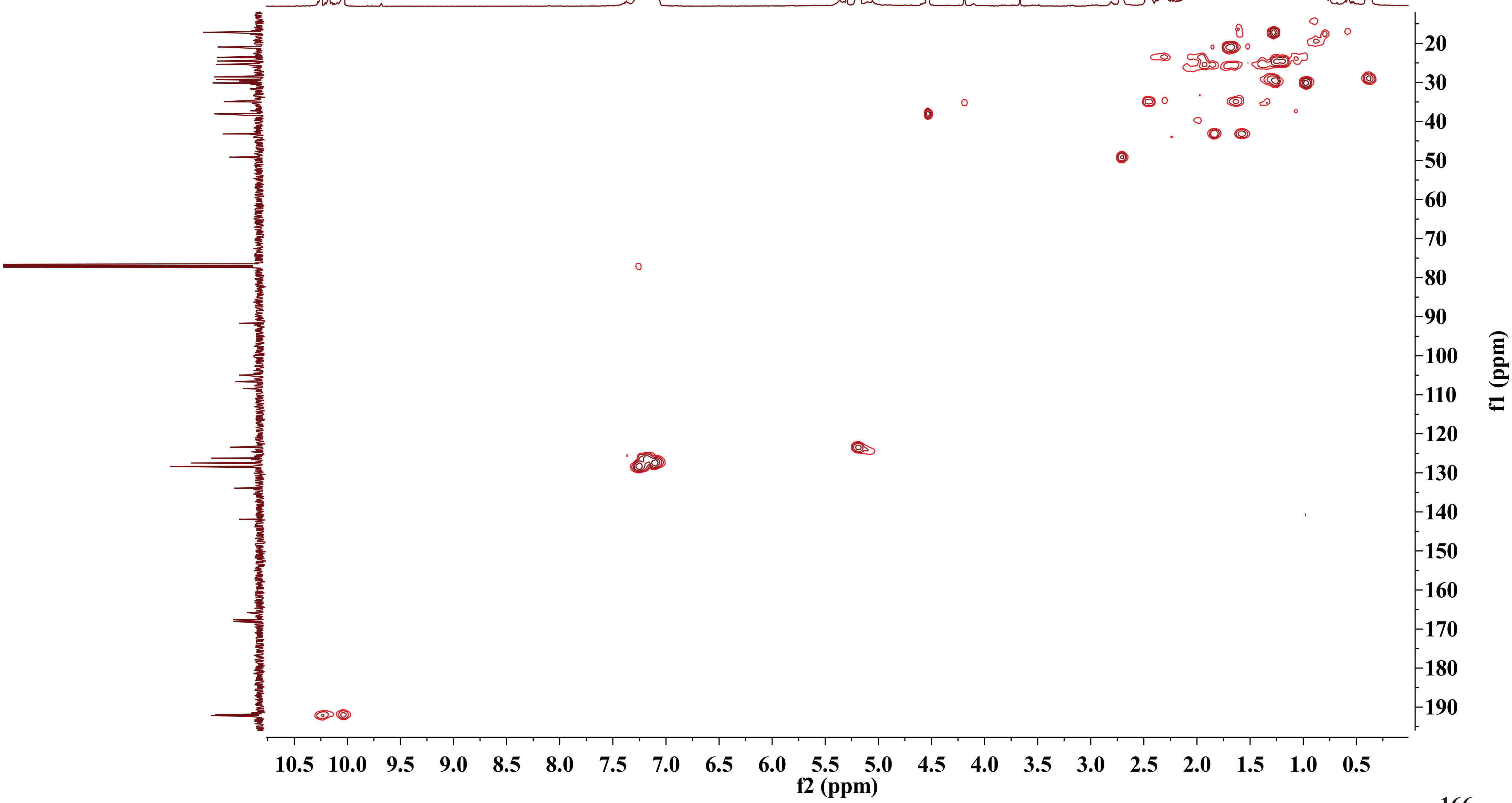

S8.4. ^{1}H-^{1}H COSY spectrum of compound **12**

In CDCl3

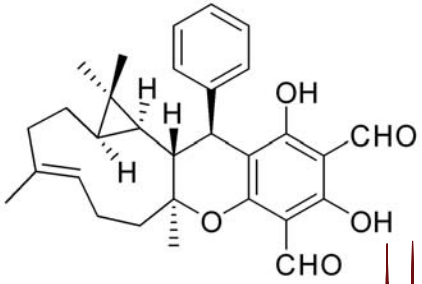

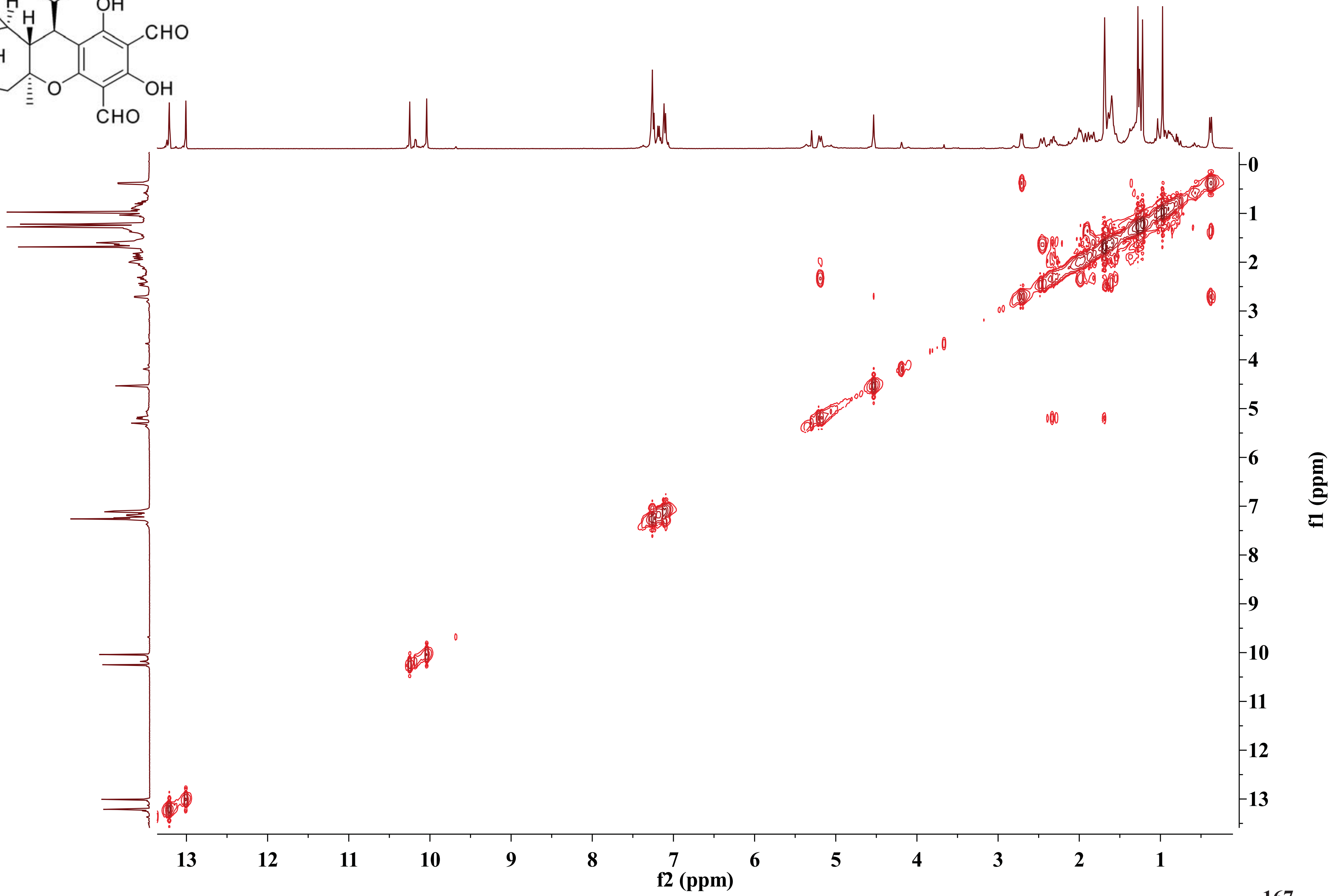

S8.5. HMBC spectrum of compound **12**

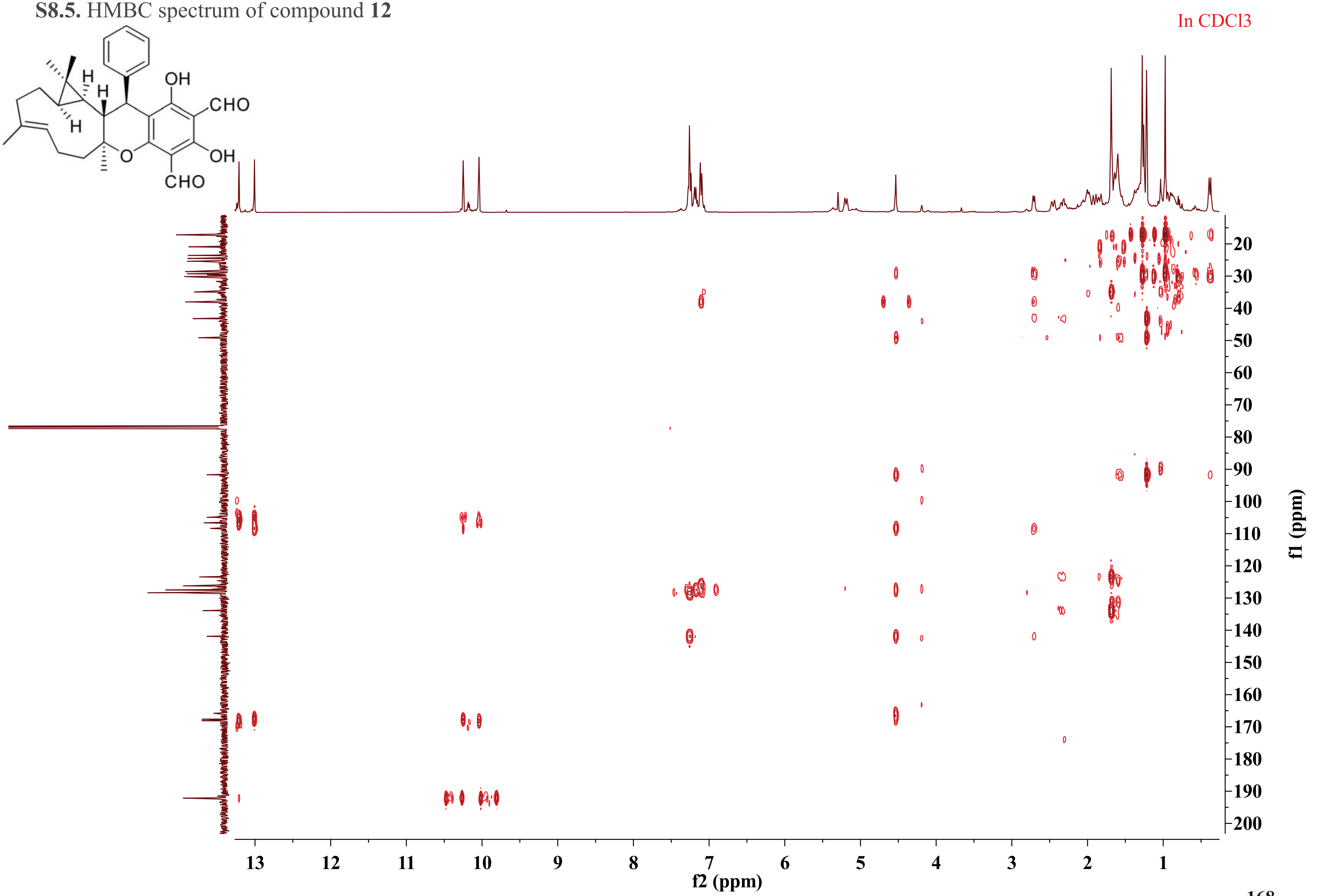

S8.6. NOESY spectrum of compound **12**

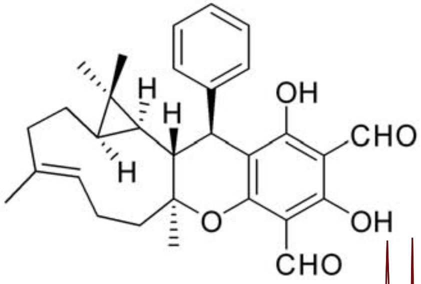

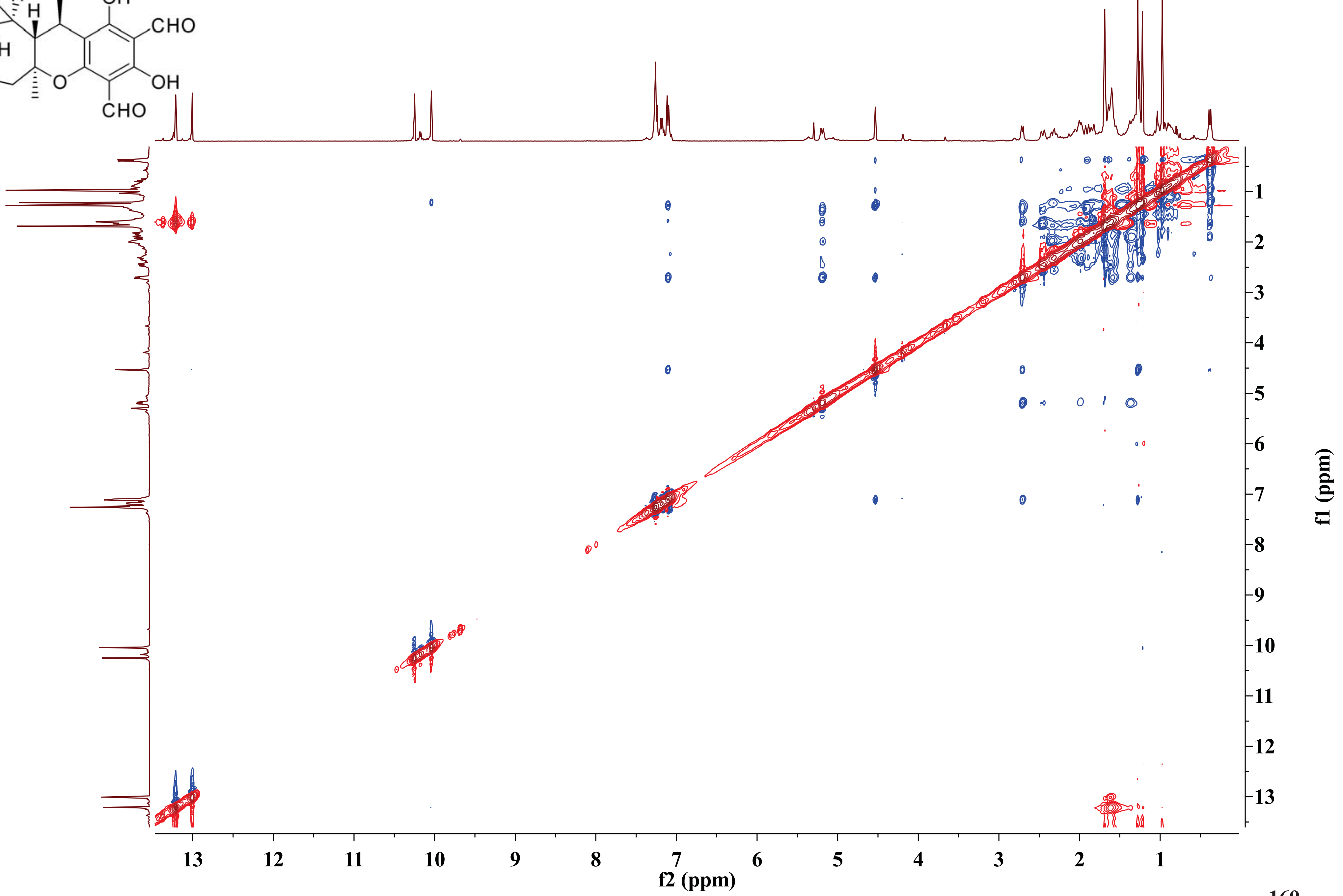

S8.7. ¹H NMR spectrum of compound **12**

In pyridine-d5

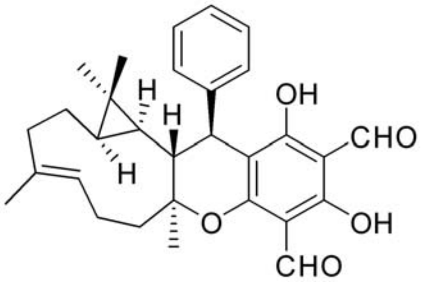

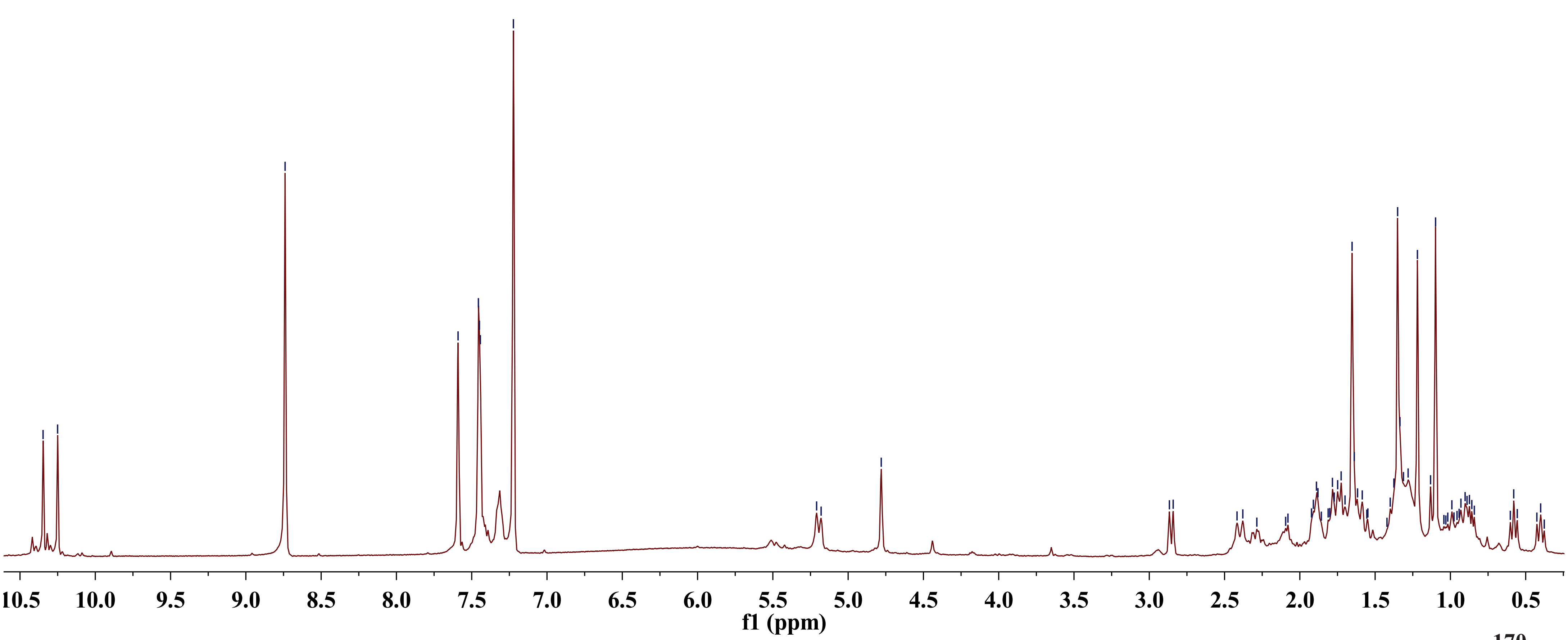

S8.8. DEPT spectra of compound **12**

In pyridine-d5

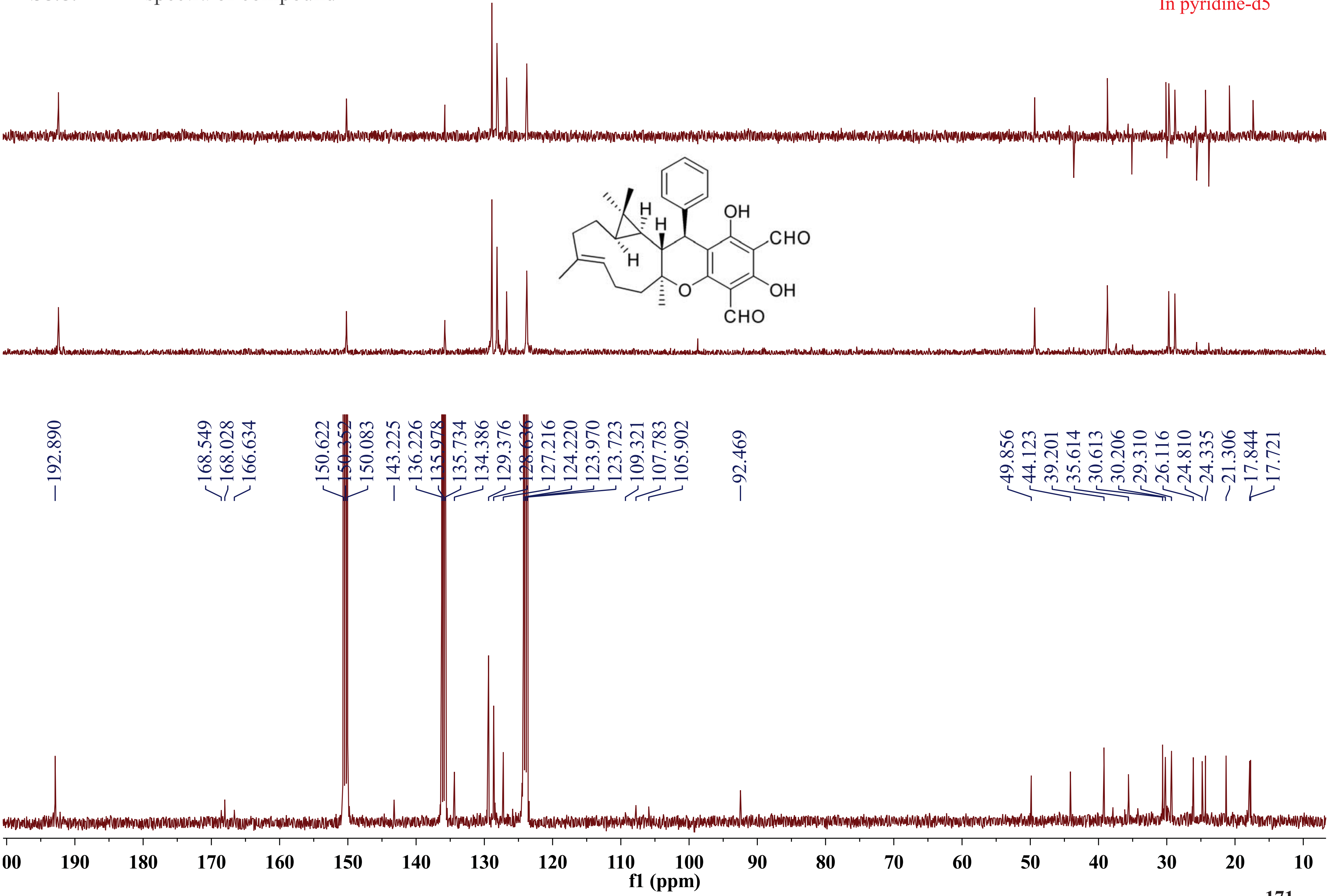

S8.9 HSQC spectrum of compound **12**

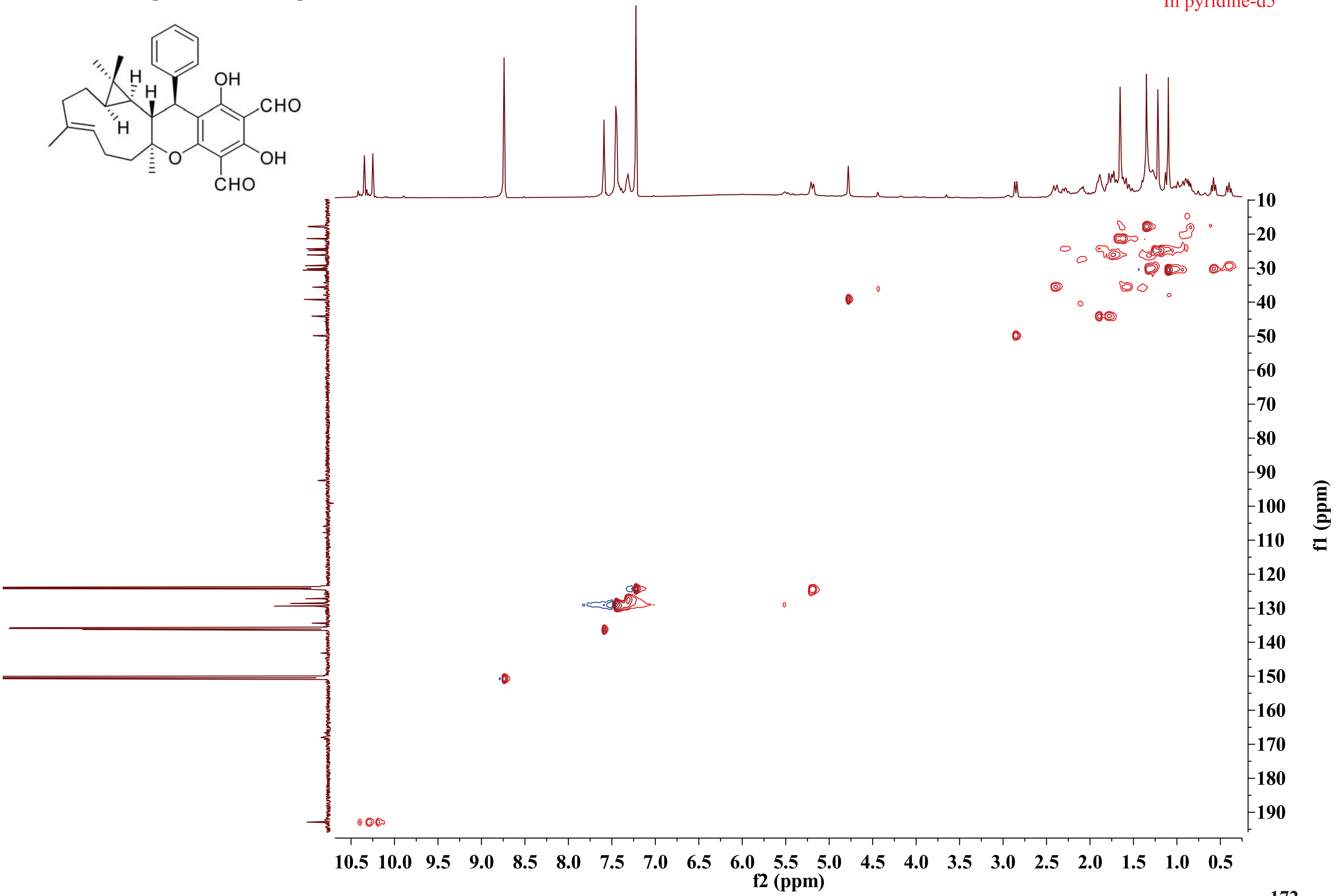

S8.10. ^{1}H-^{1}H COSY spectrum of compound **12**

In pyridine-d5

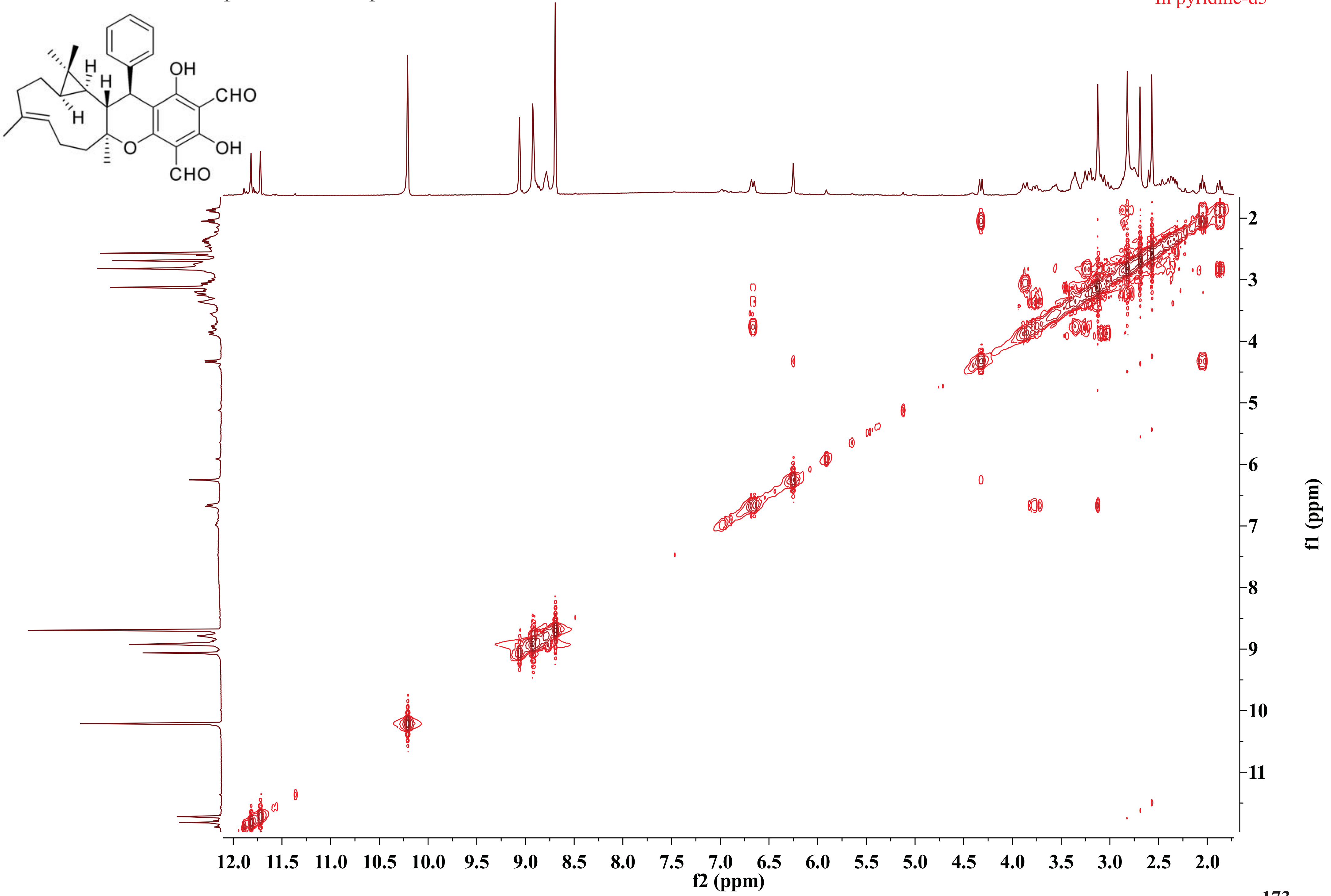

S8.11. HMBC spectrum of compound **12**

In pyridine-d5

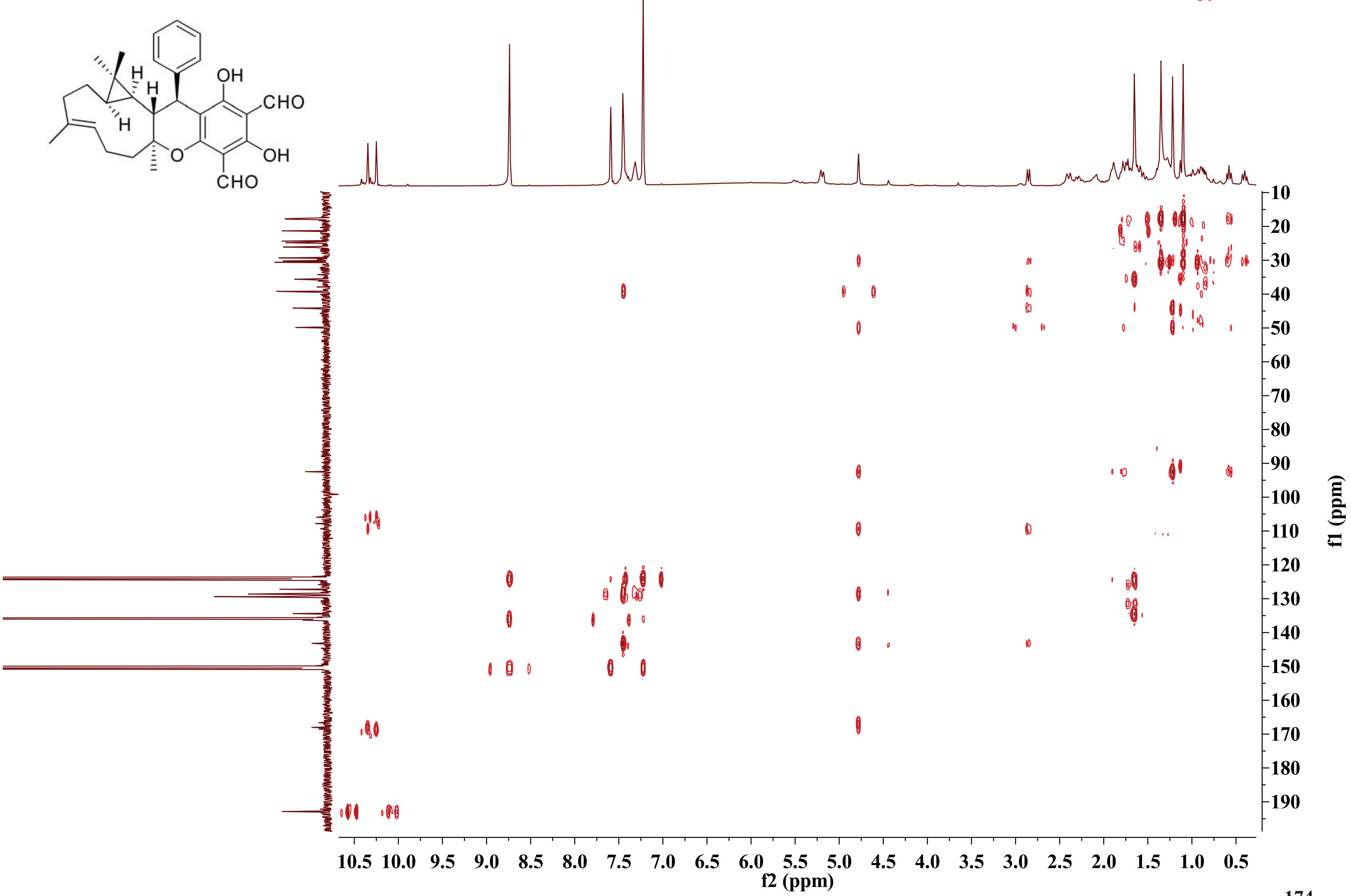

S8.12. NOESY spectrum of compound **12**

In pyridine-d5

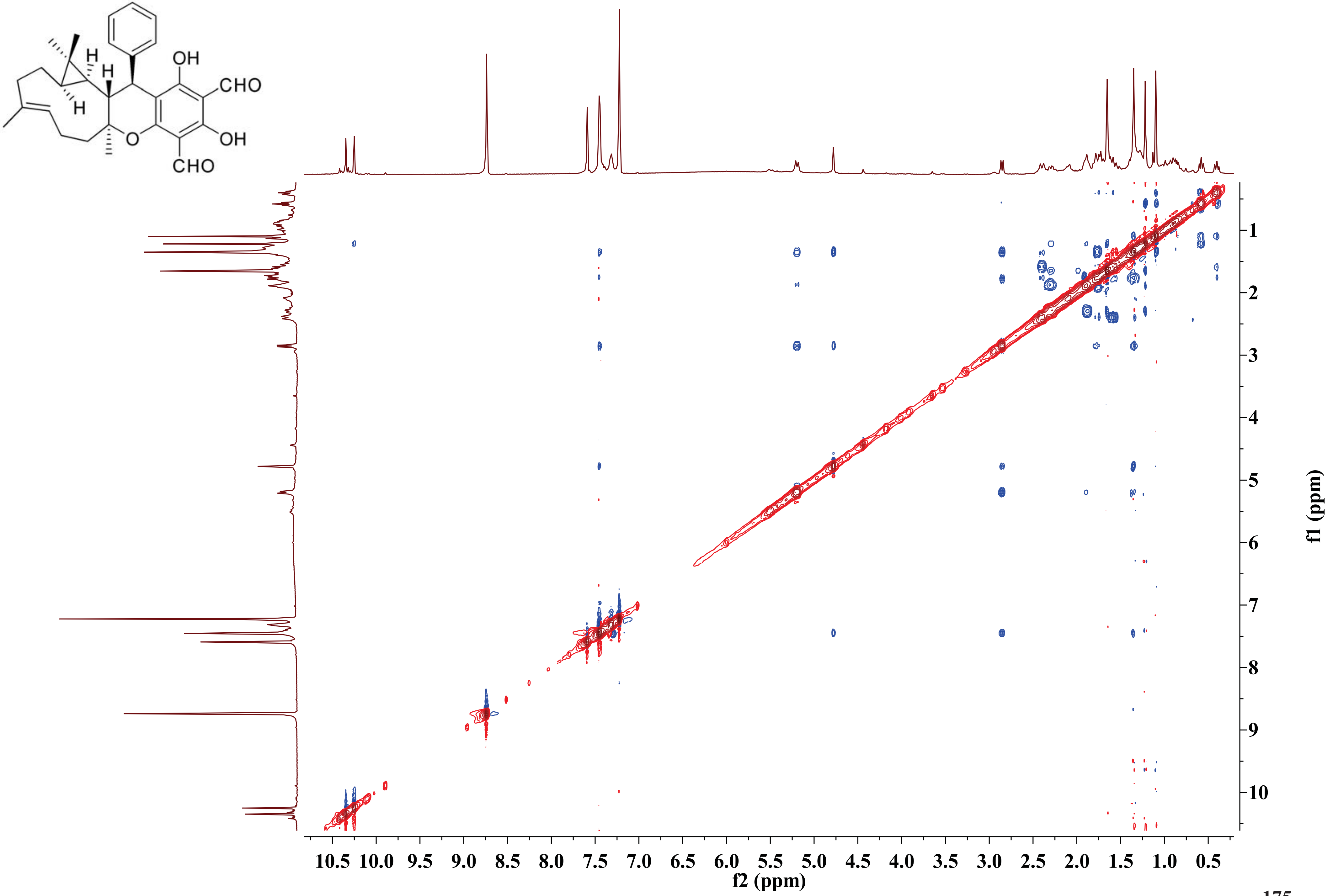

S8.13. ^{1}H NMR spectrum of compound **13**

In CDCl3

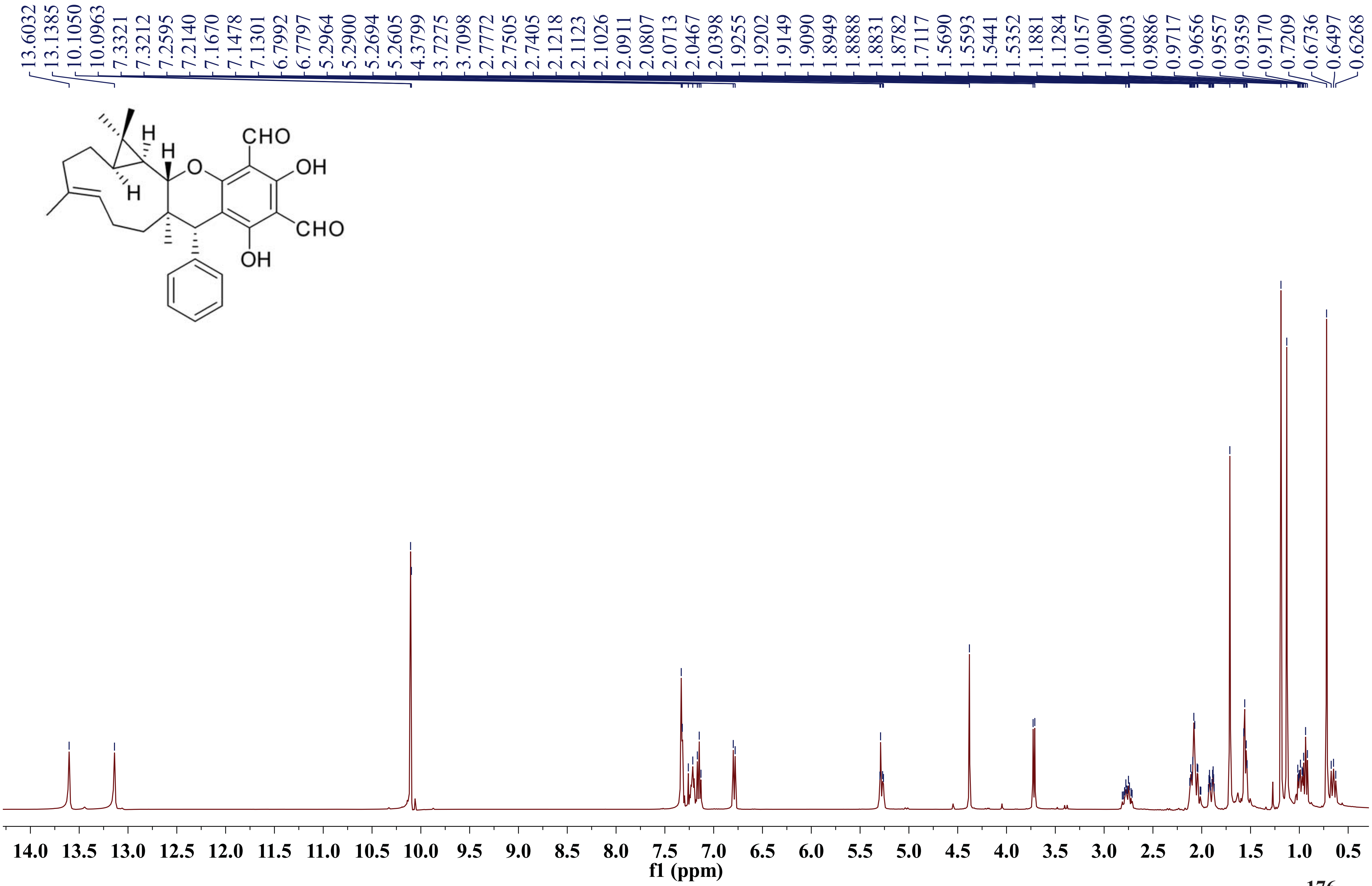

S8.14. DEPT spectra of compound **13**

In CDCl3

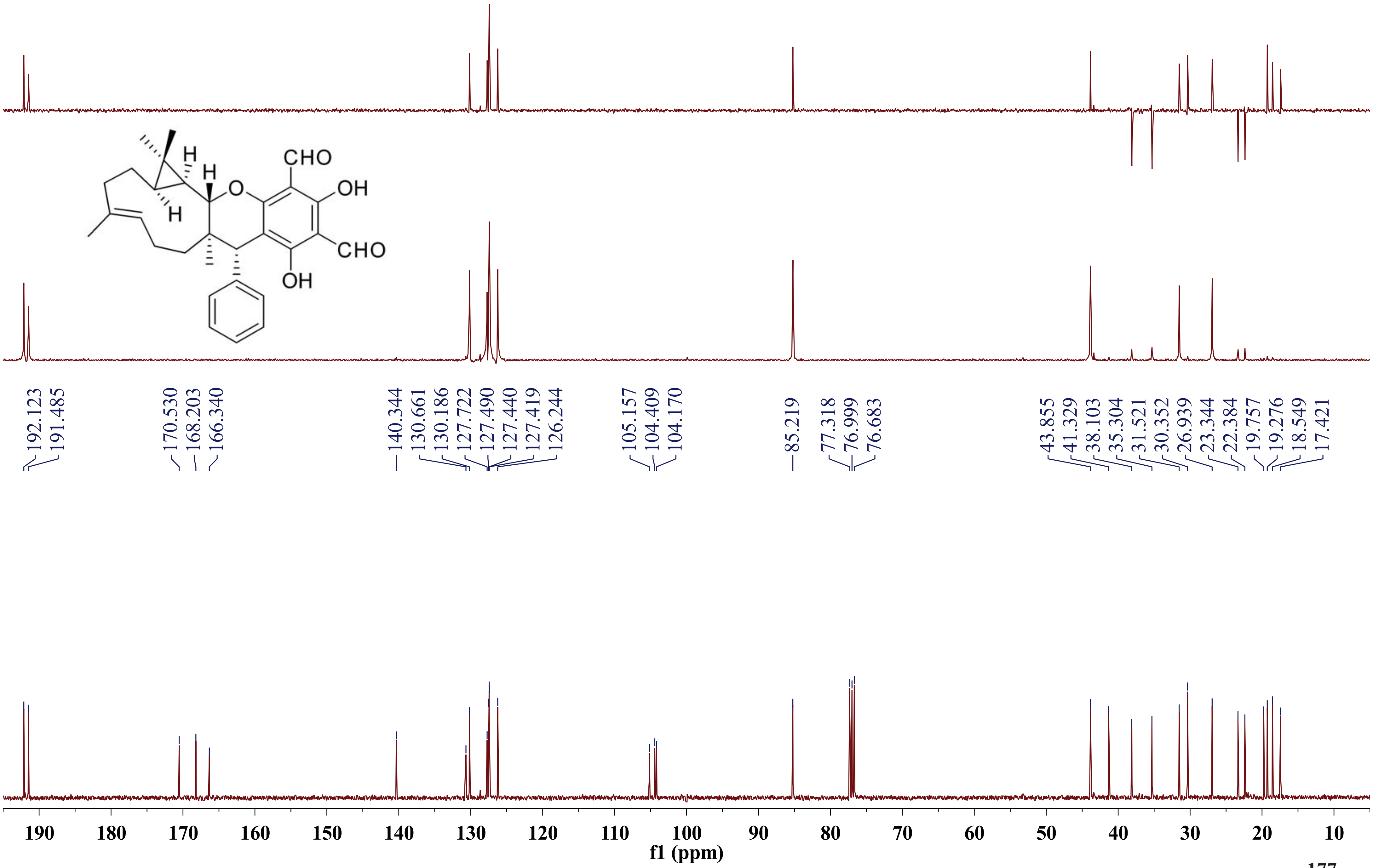

S8.15. ^{1}H NMR spectrum of compound **14** and **15**

In CDCl3

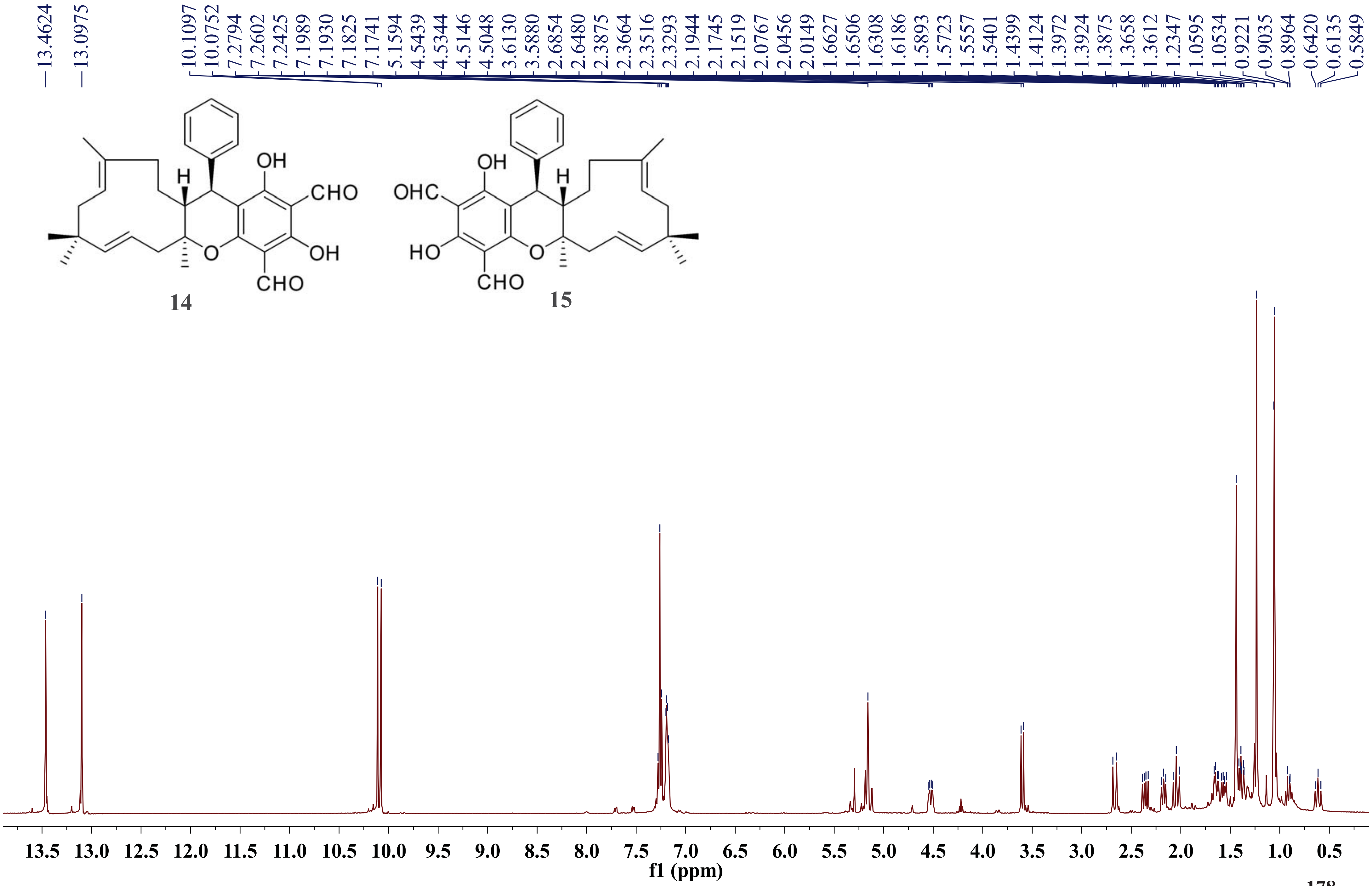

S8.16. ^{13}C NMR spectrum of compound **14** and **15**

In CDCl3

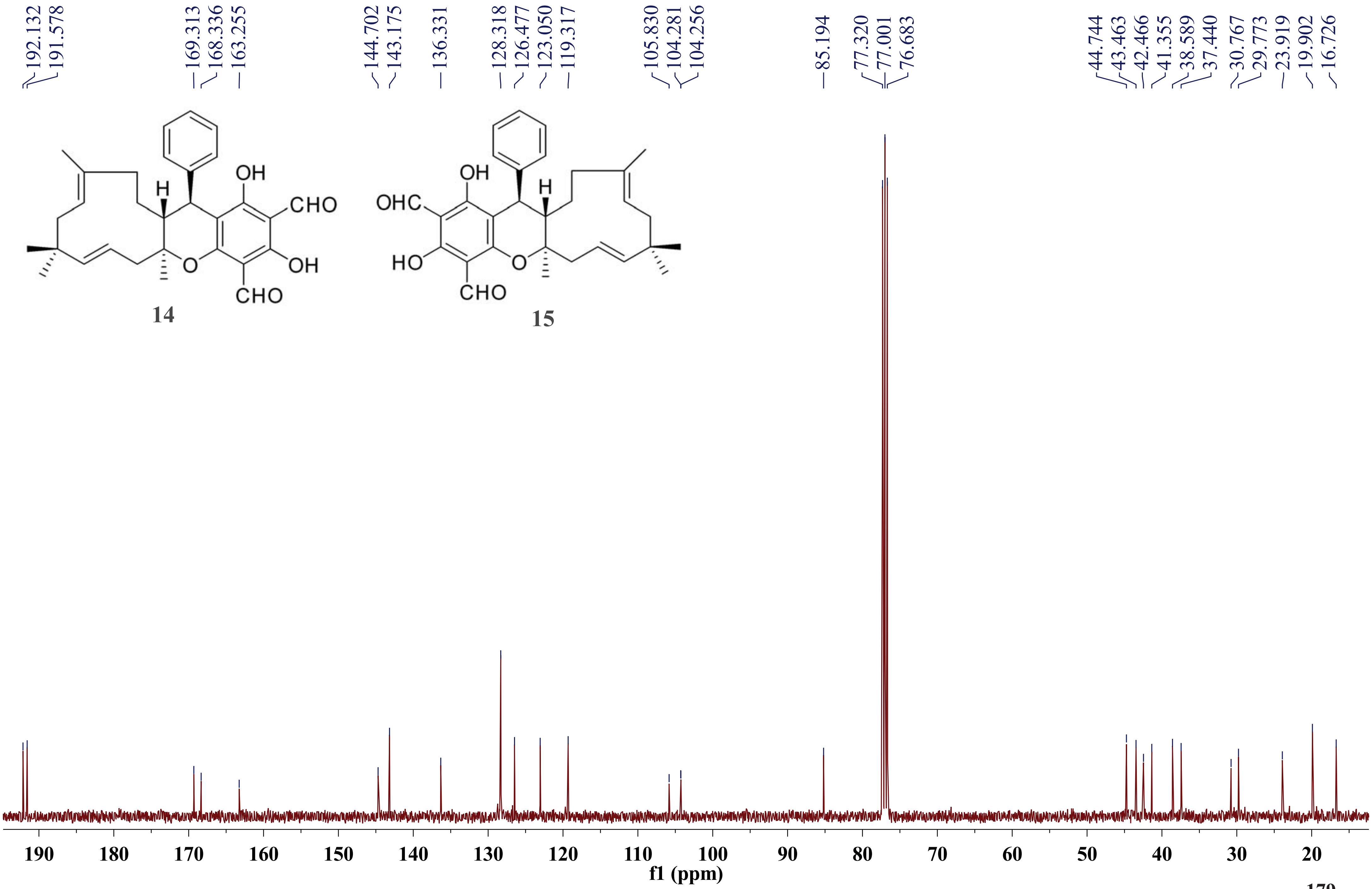

S8.17. ^{1}H NMR spectrum of compound **16**

In CDCl3

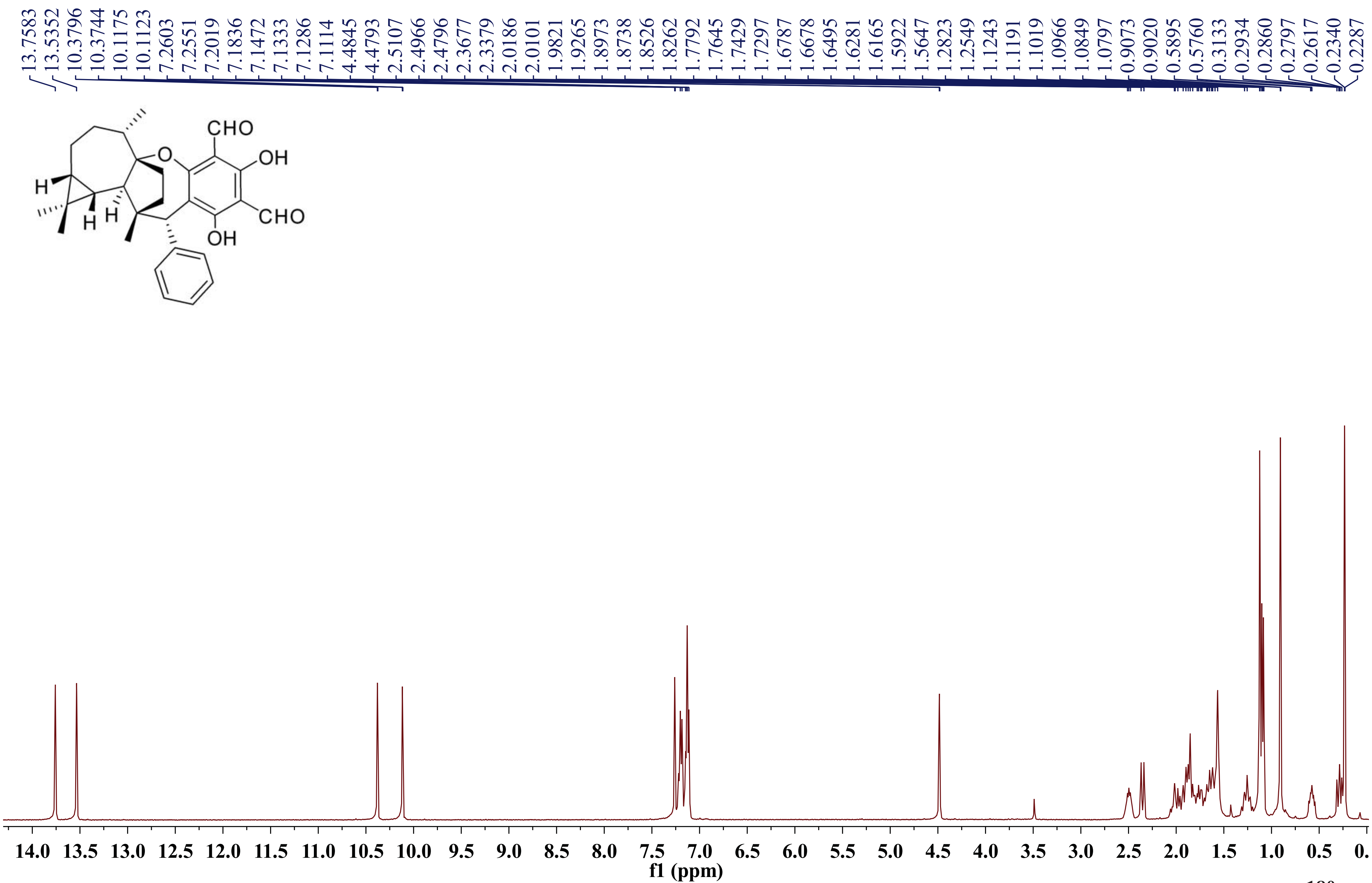

S8.18. DEPT spectra of compound **16**

In CDCl3

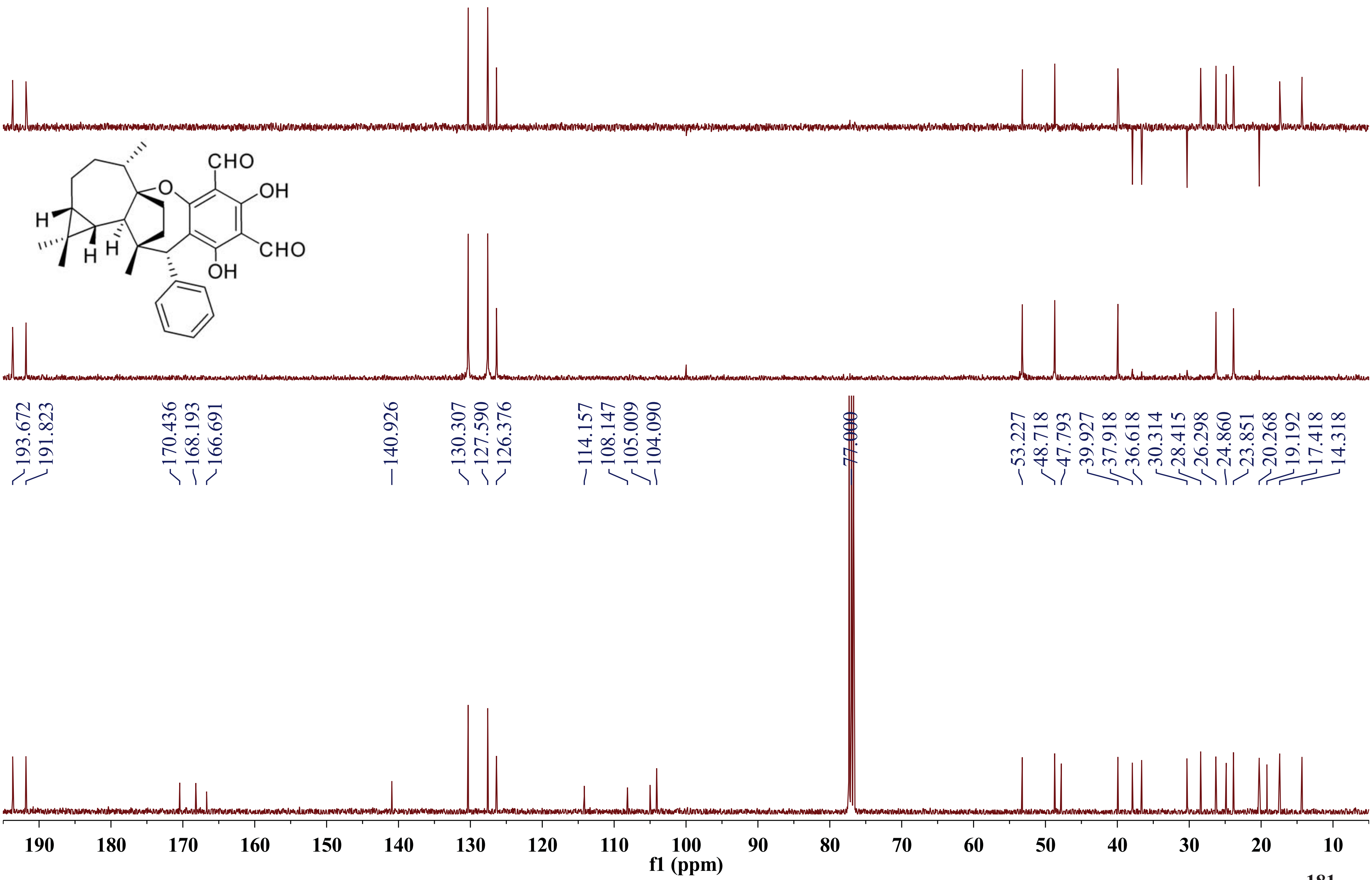

S8.19. ^{1}H NMR spectrum of compound **17**

In CDCl3

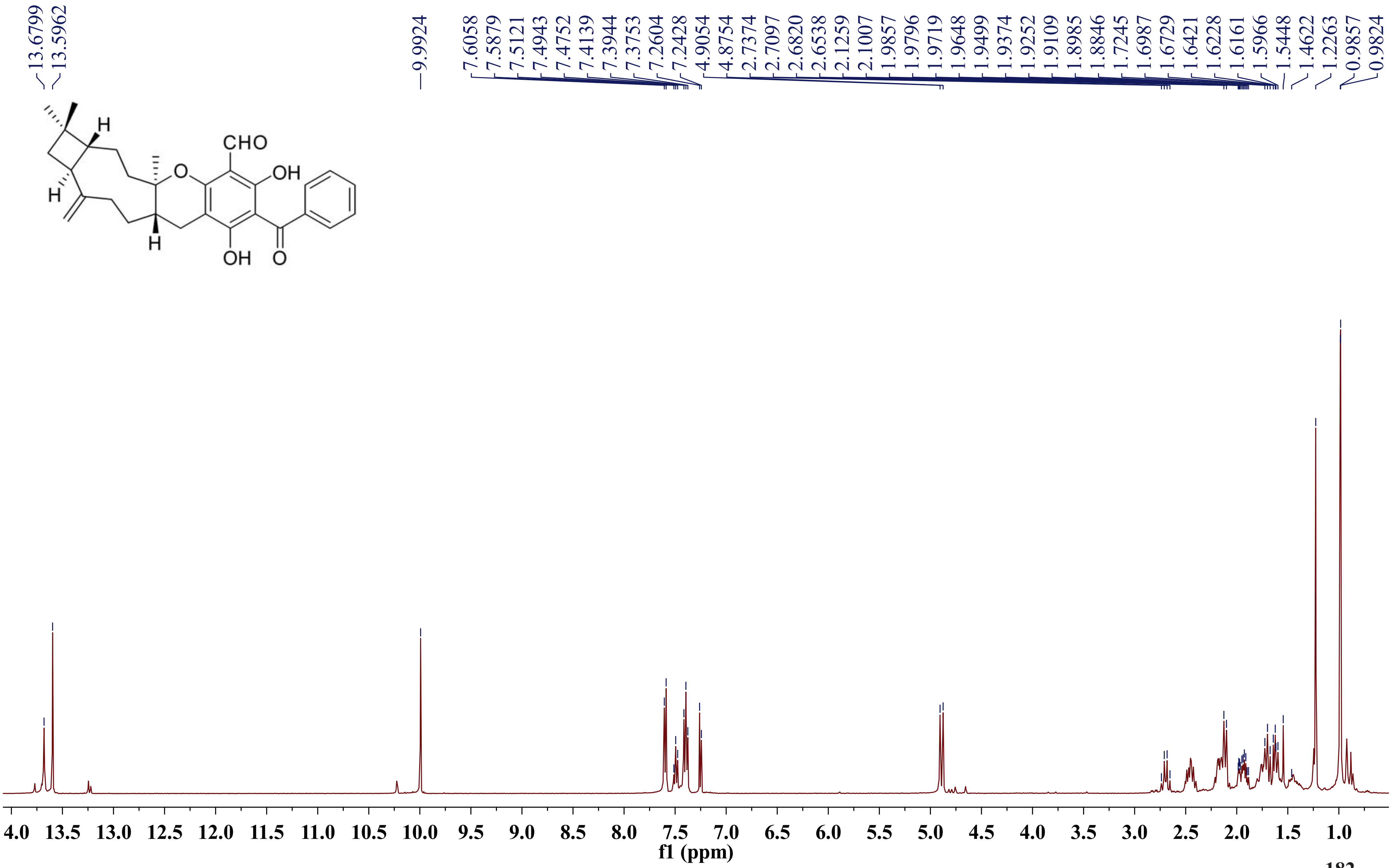

S8.20. DEPT spectra of compound **17**

In CDCl3

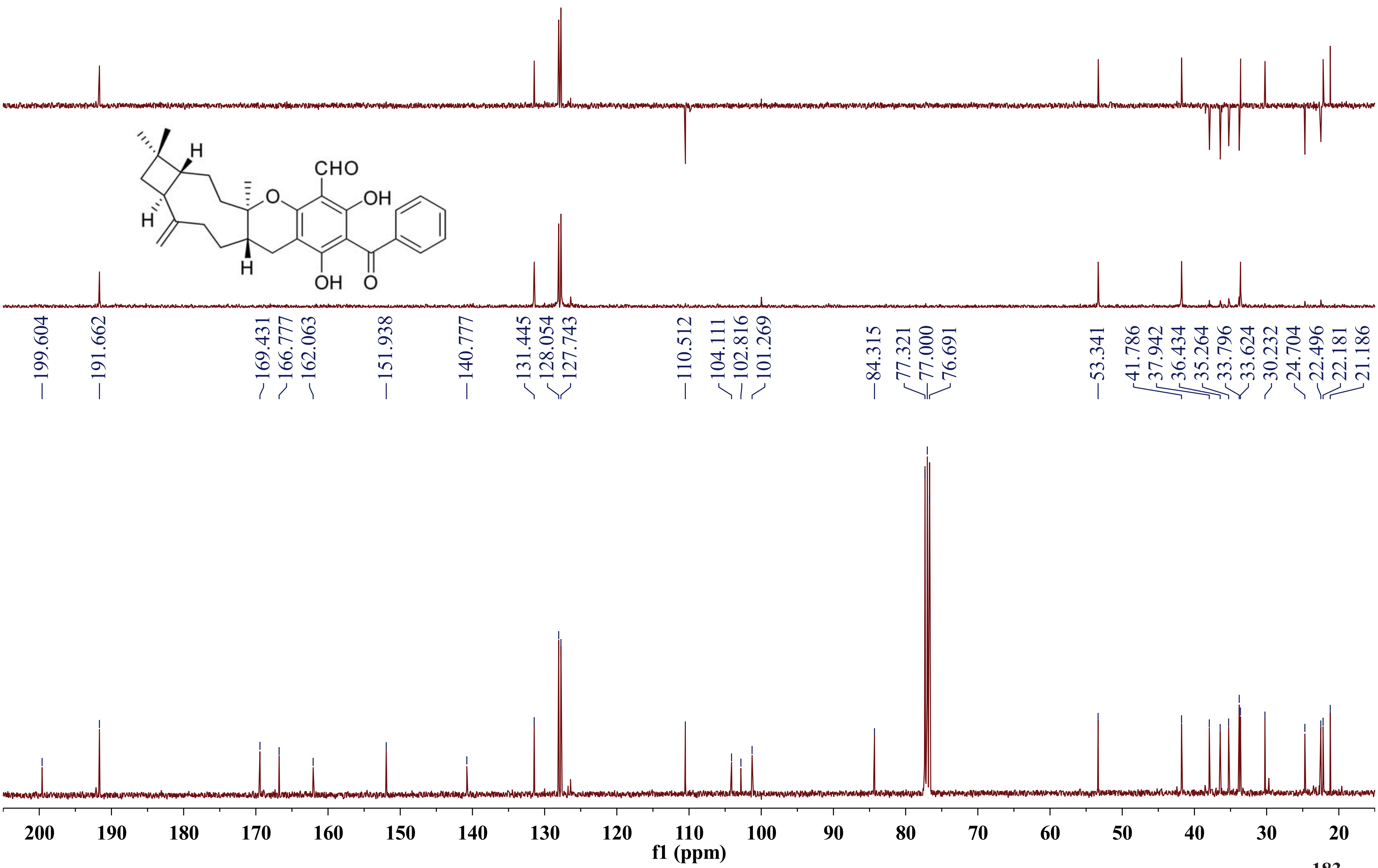

S8.21. ^{1}H NMR spectrum of compound **18**

In CDCl3

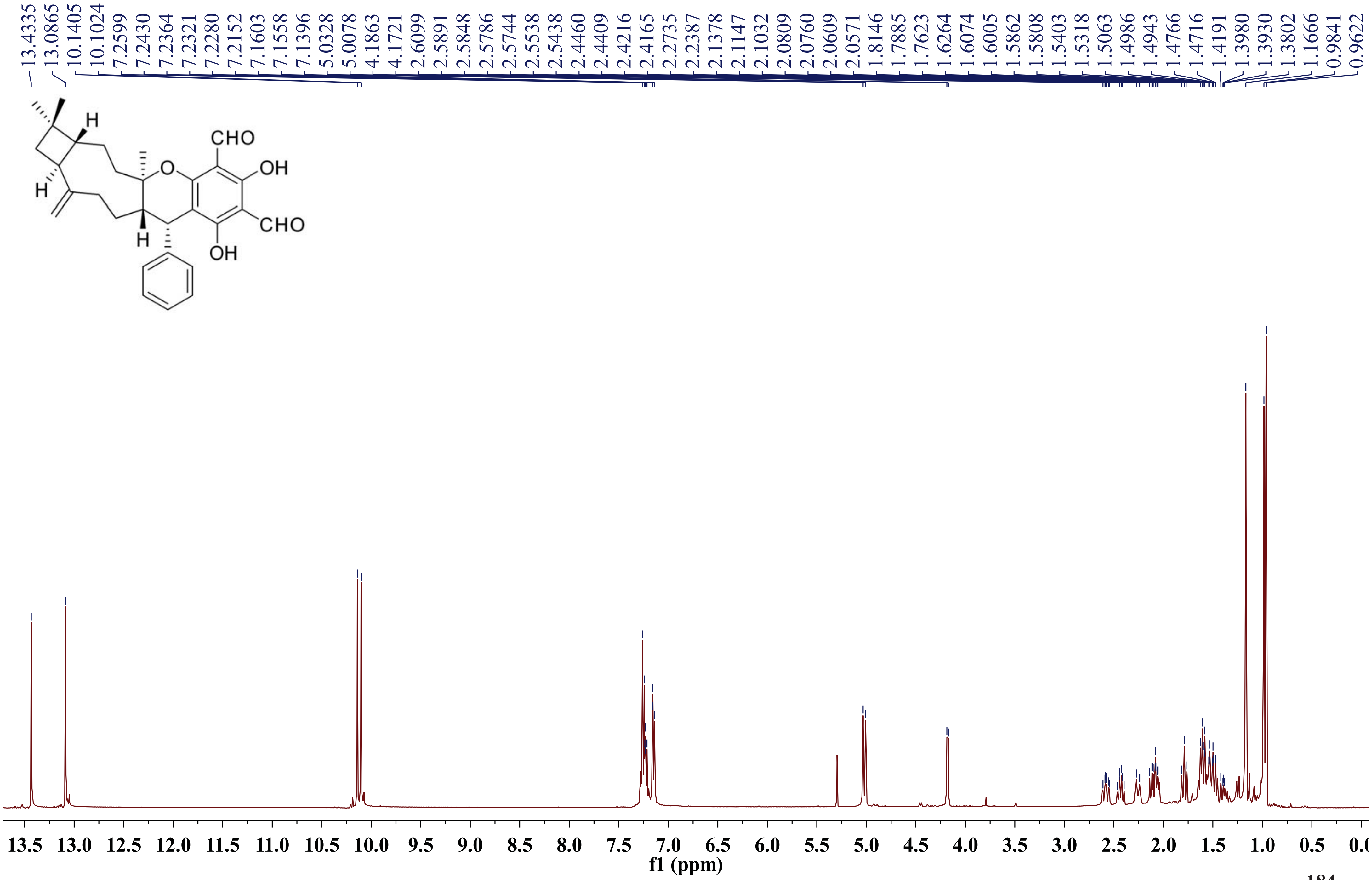

S8.22. ^{13}C NMR spectrum of compound **18**

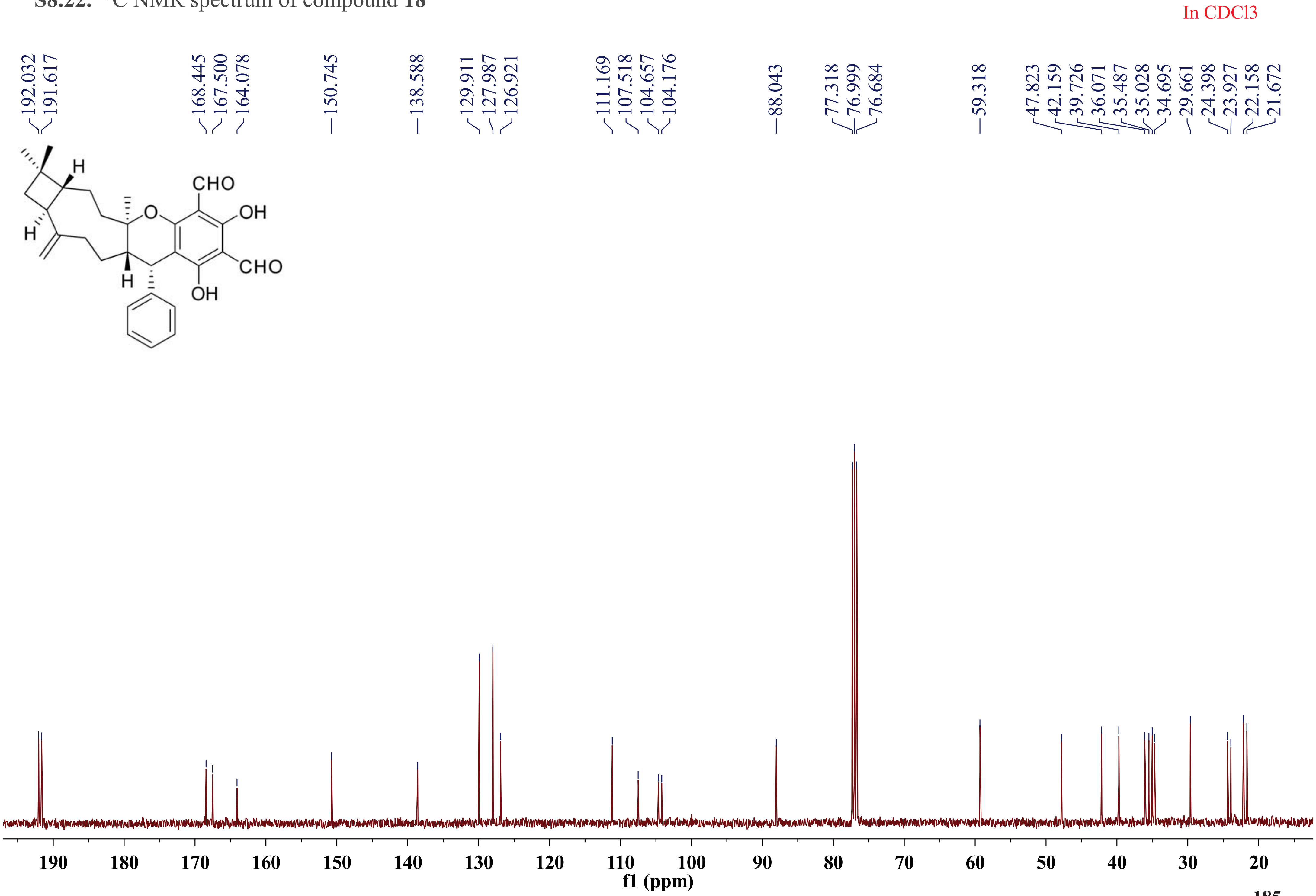

S8.23. ¹H NMR spectrum of compound **19**

In CDCl3

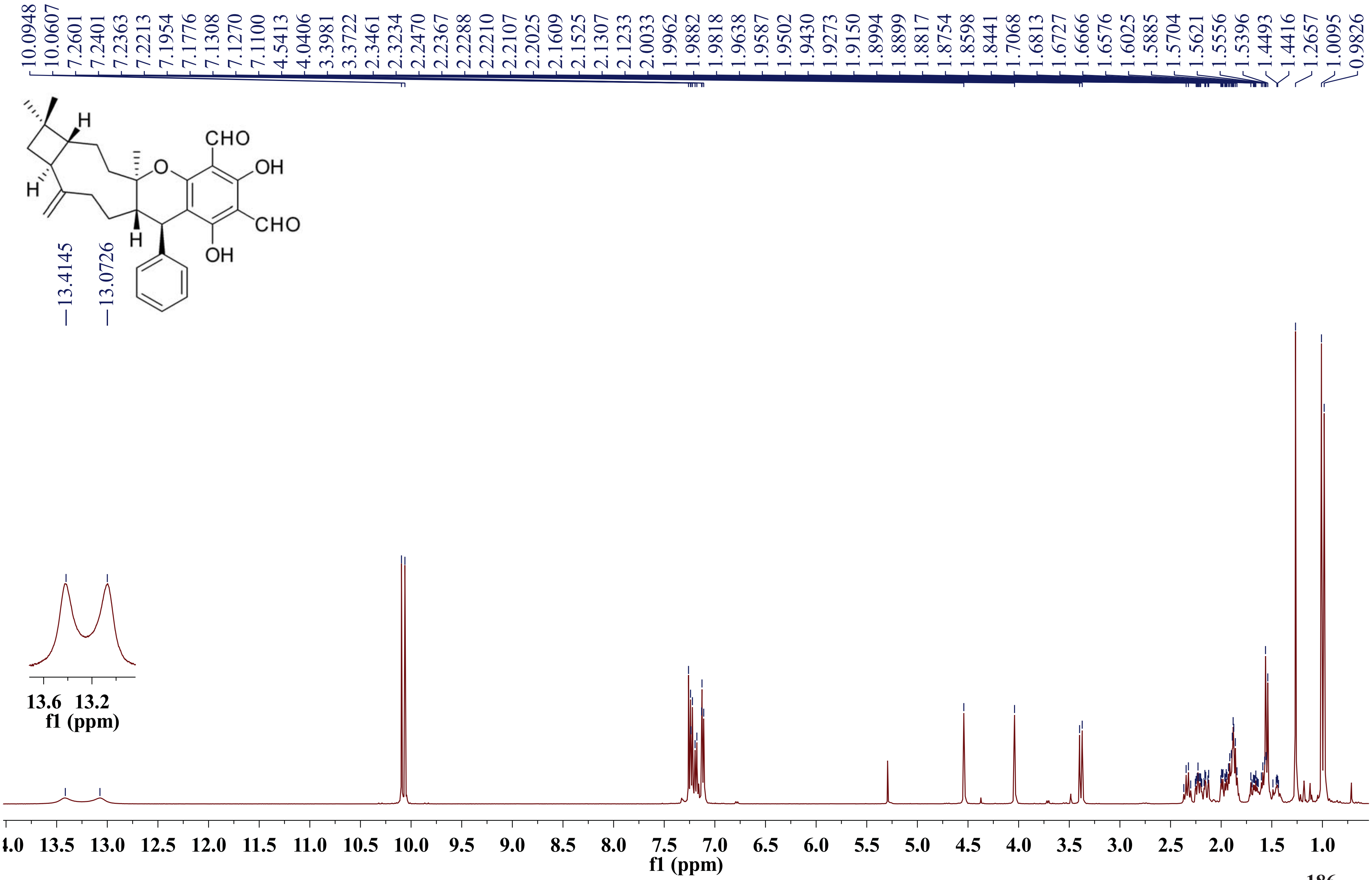

S8.24. ^{13}C NMR spectrum of compound **19**

In CDCl3

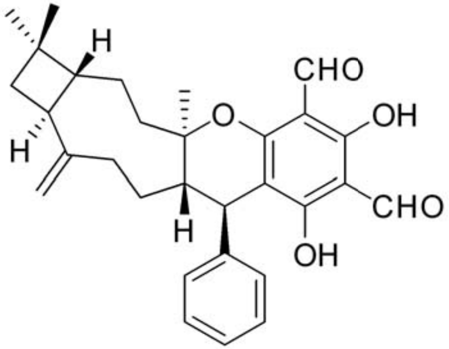

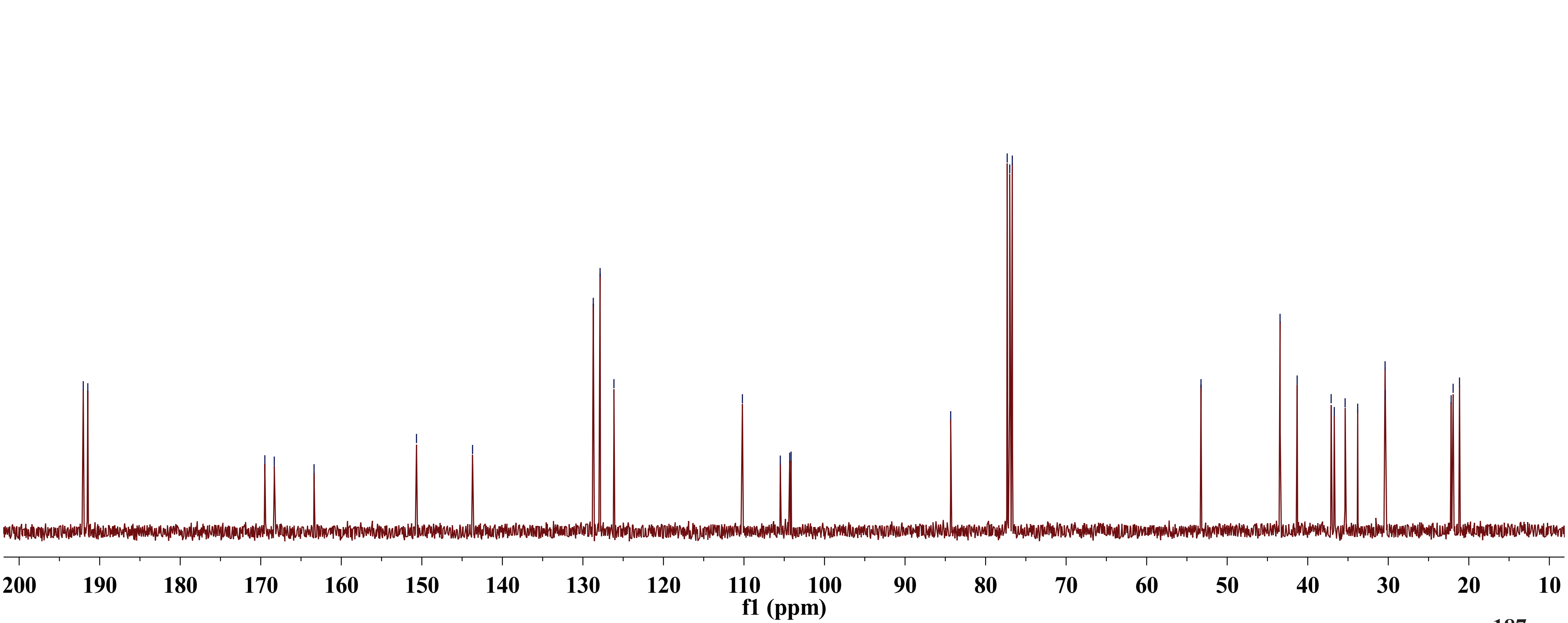

S8.25. ^{13}C NMR spectrum of compound **20**

In CDCl3

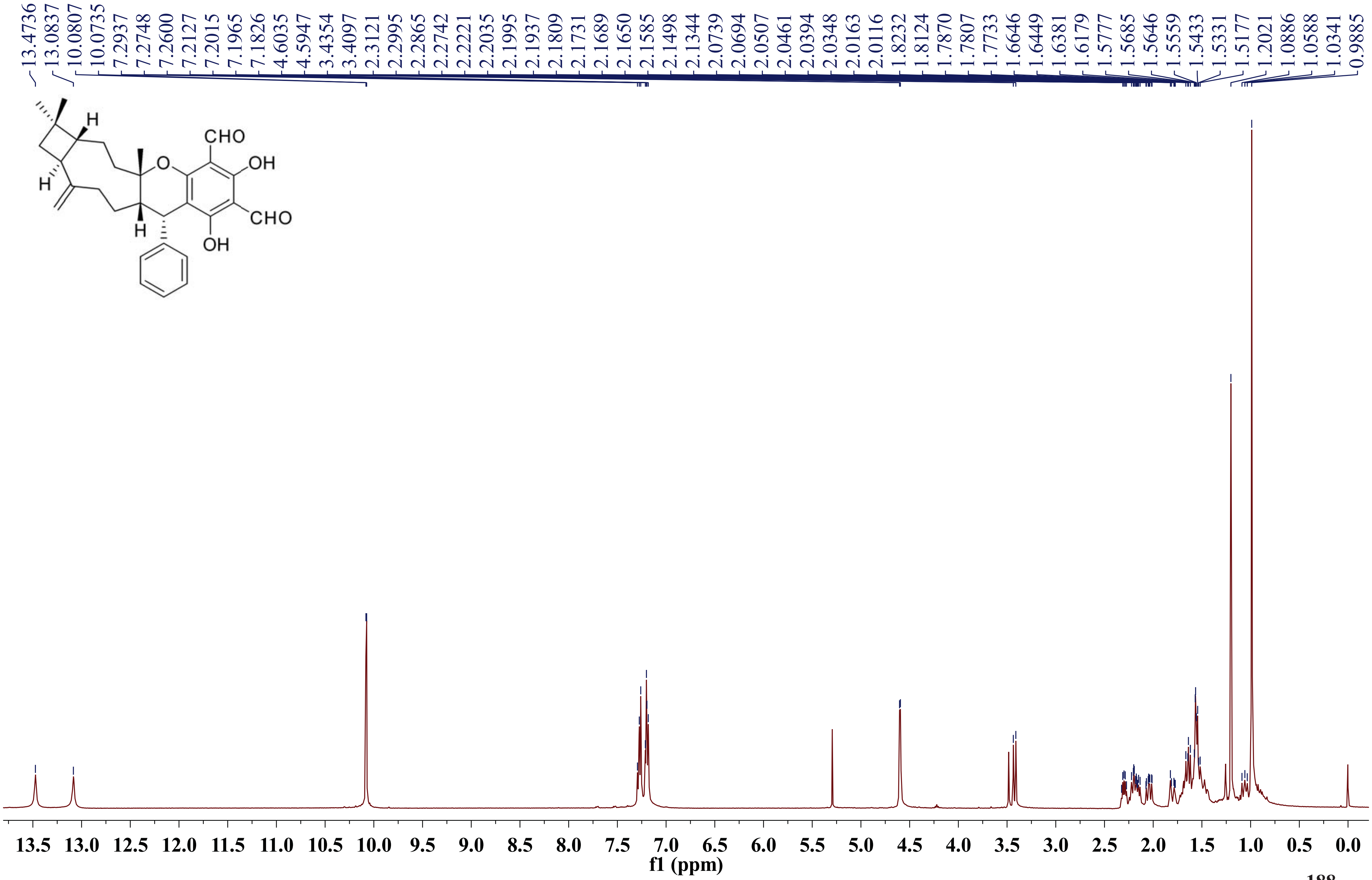

S8.26. DEPT spectra of compound **20**

In CDCl3

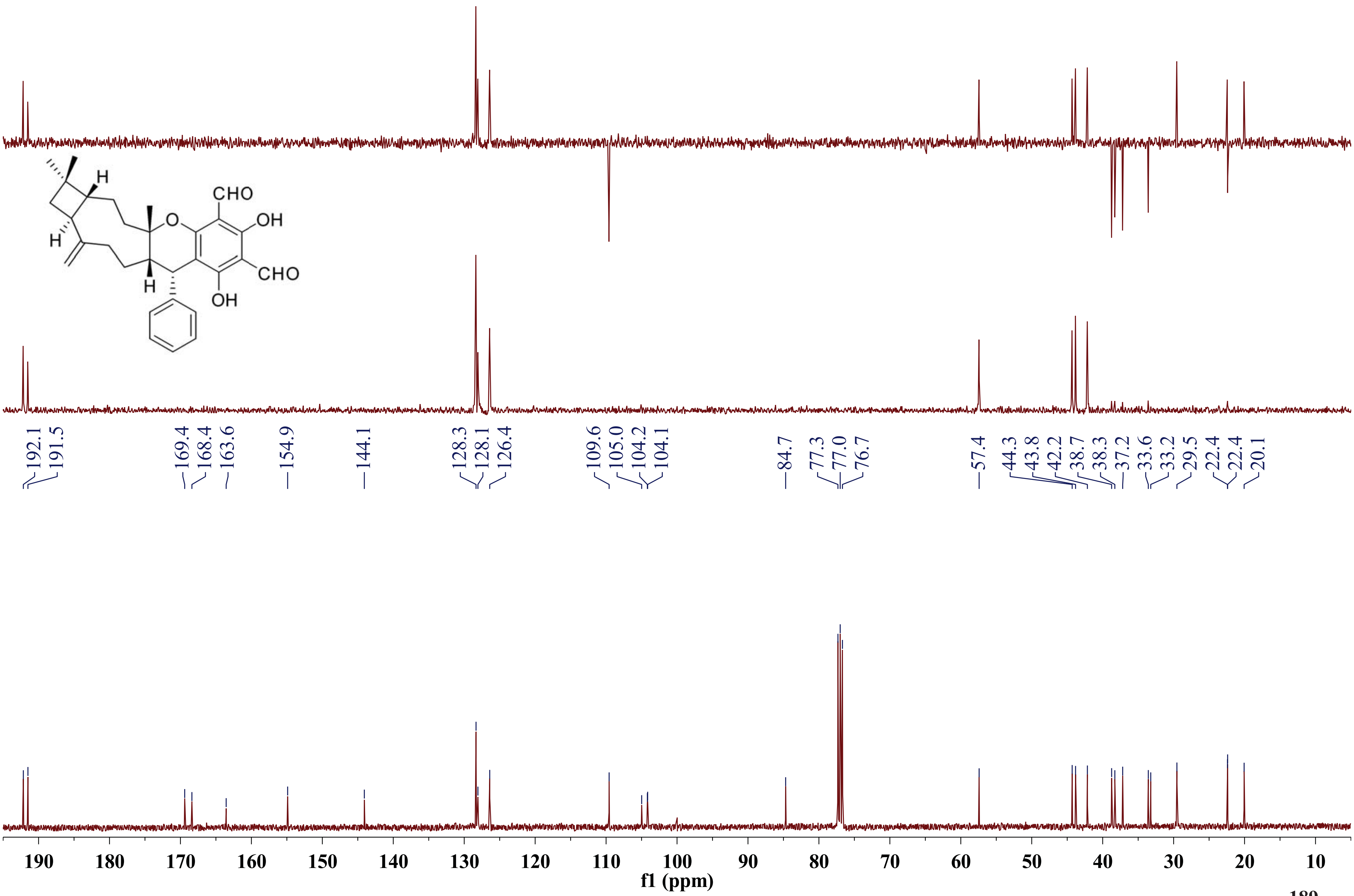

S8.27. ^{1}H NMR spectrum of compound **21**

In CDCl3

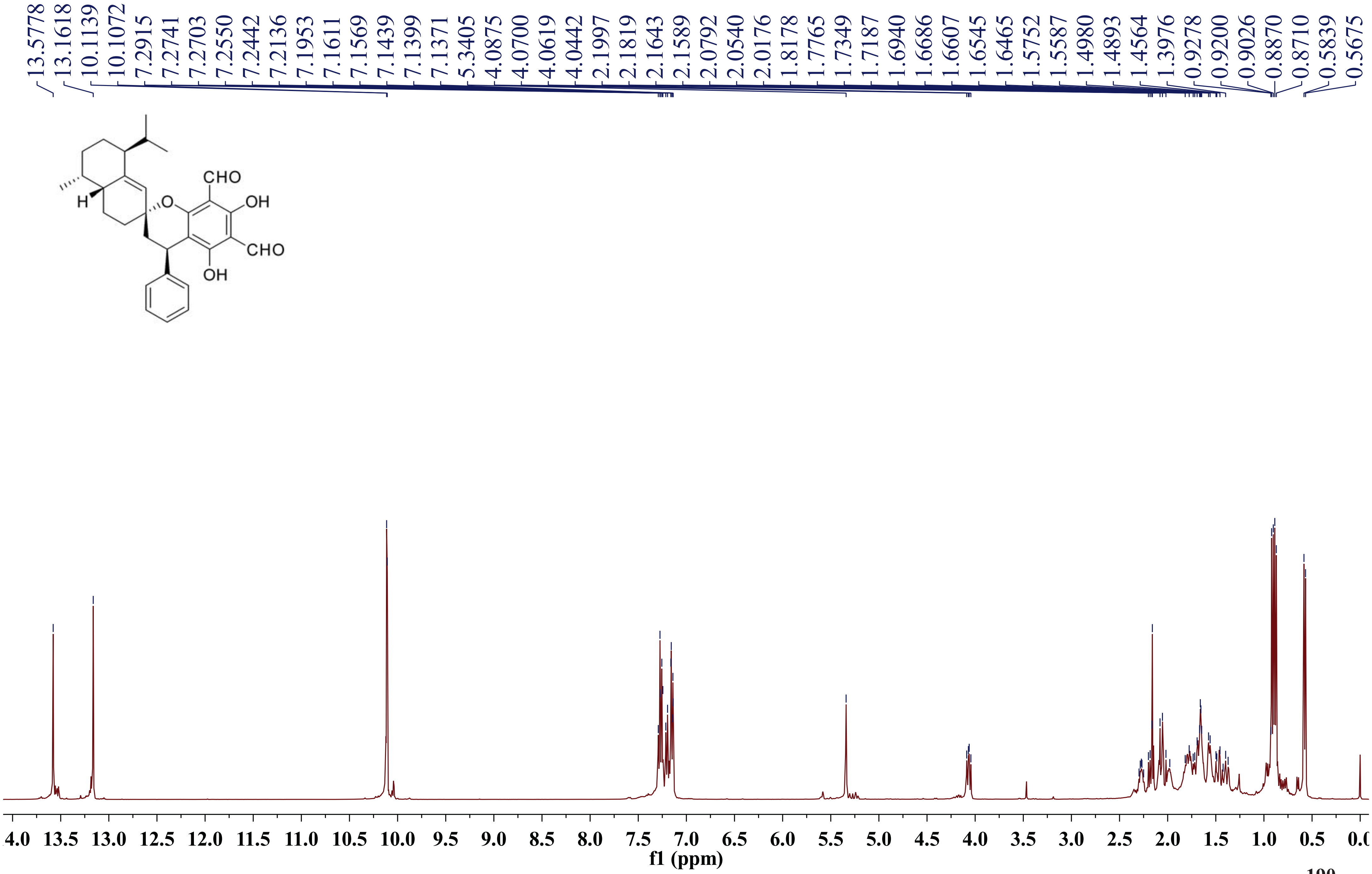

S8.28. DEPT spectra of compound **21**

In CDCl3

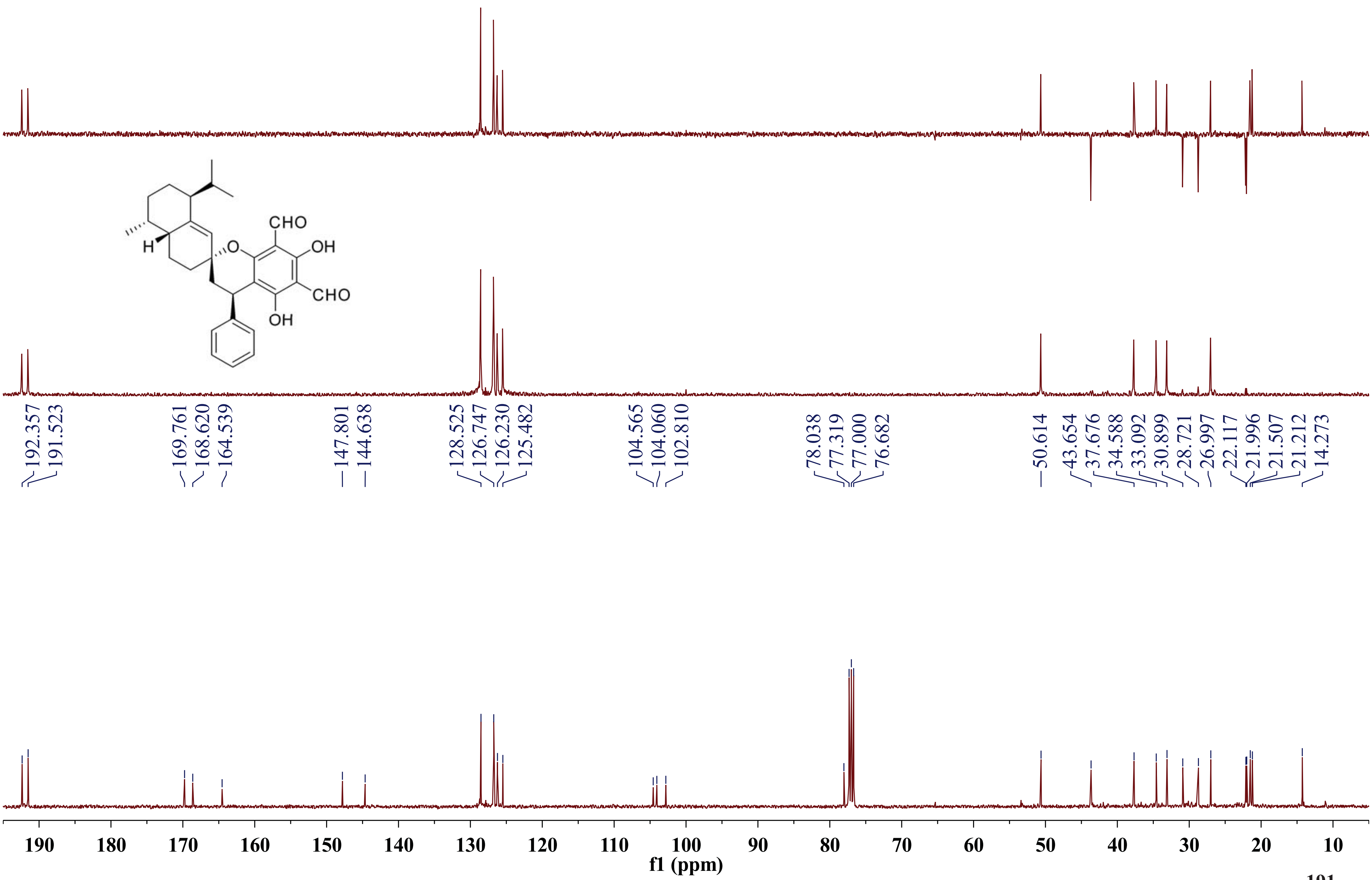

S8.29. ^{1}H NMR spectrum of compound **22**

In CDCl3

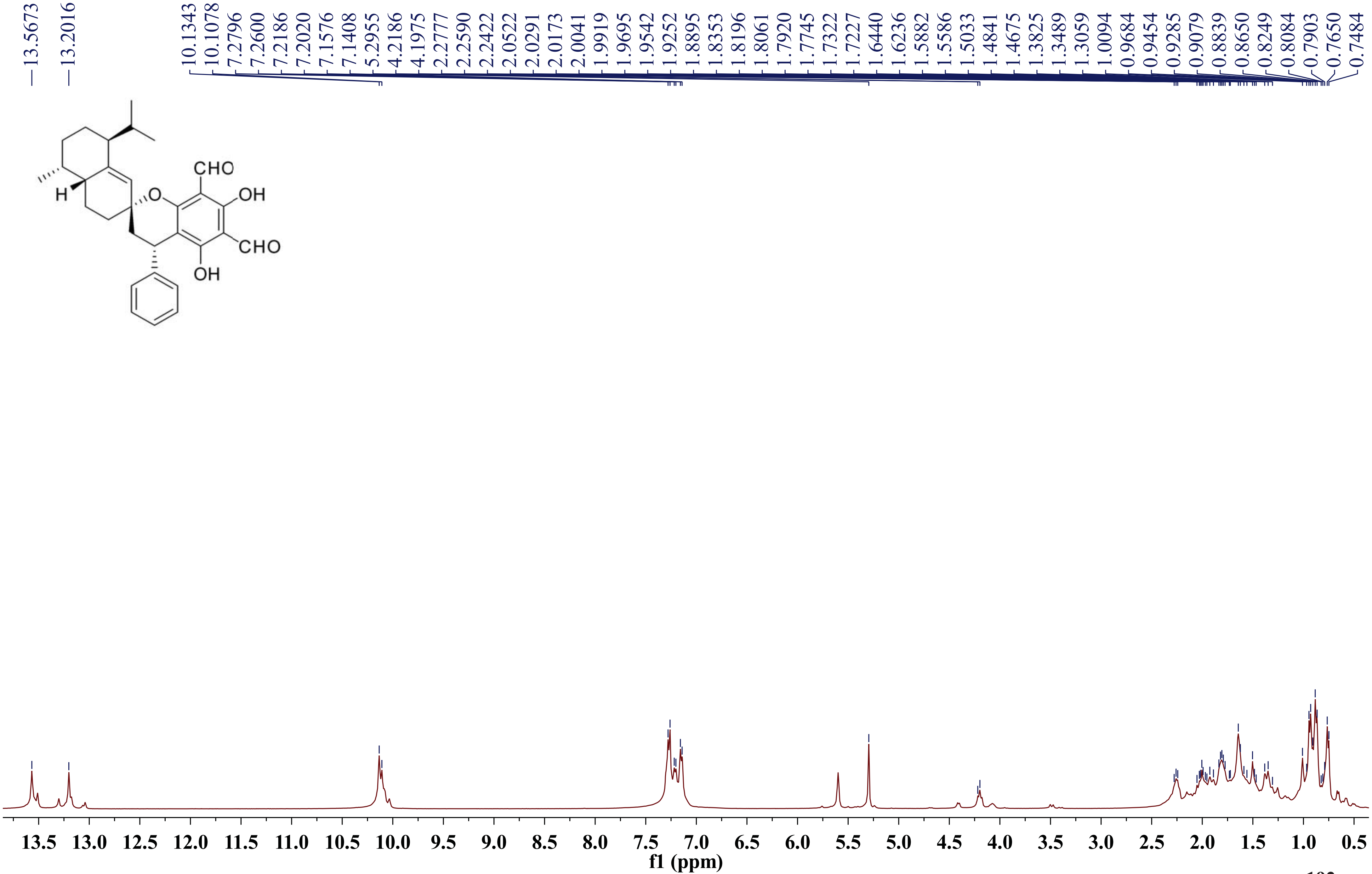

S8.30. DEPT spectra of compound **22**

In CDCl3

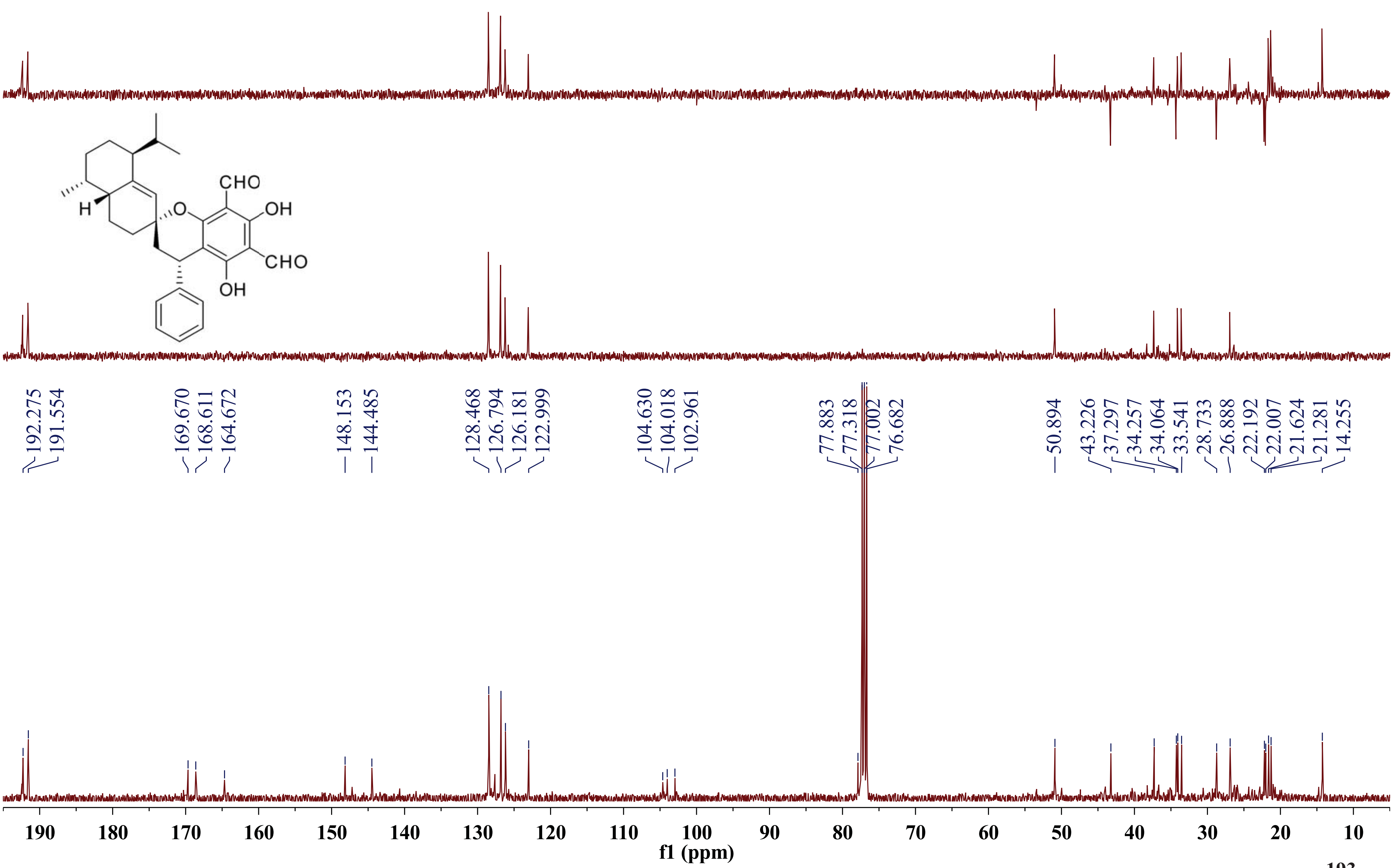

S8.31. ^{1}H NMR spectrum of compound **23**

In CDCl3

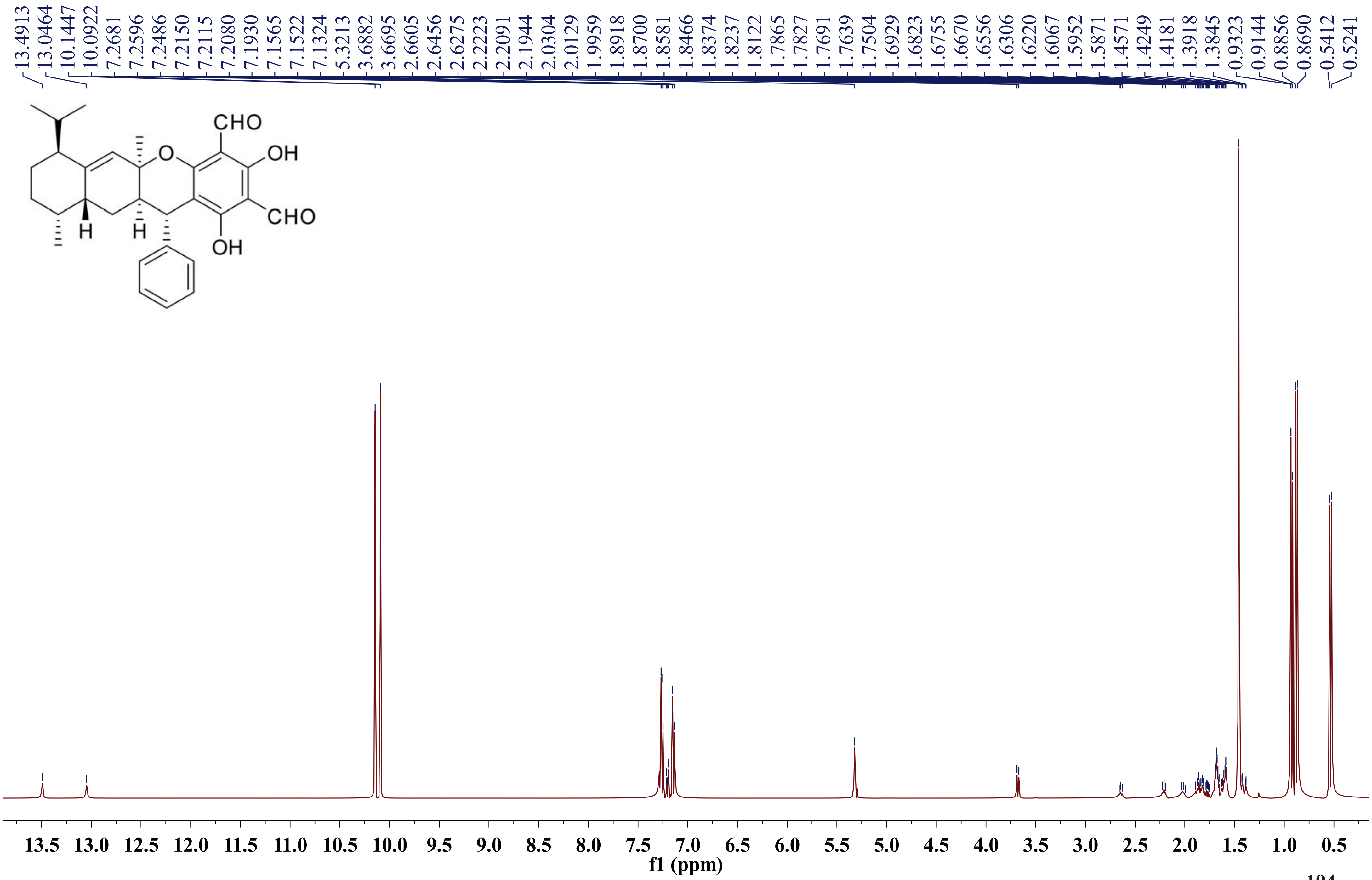

S8.32. DEPT spectra of compound **23**

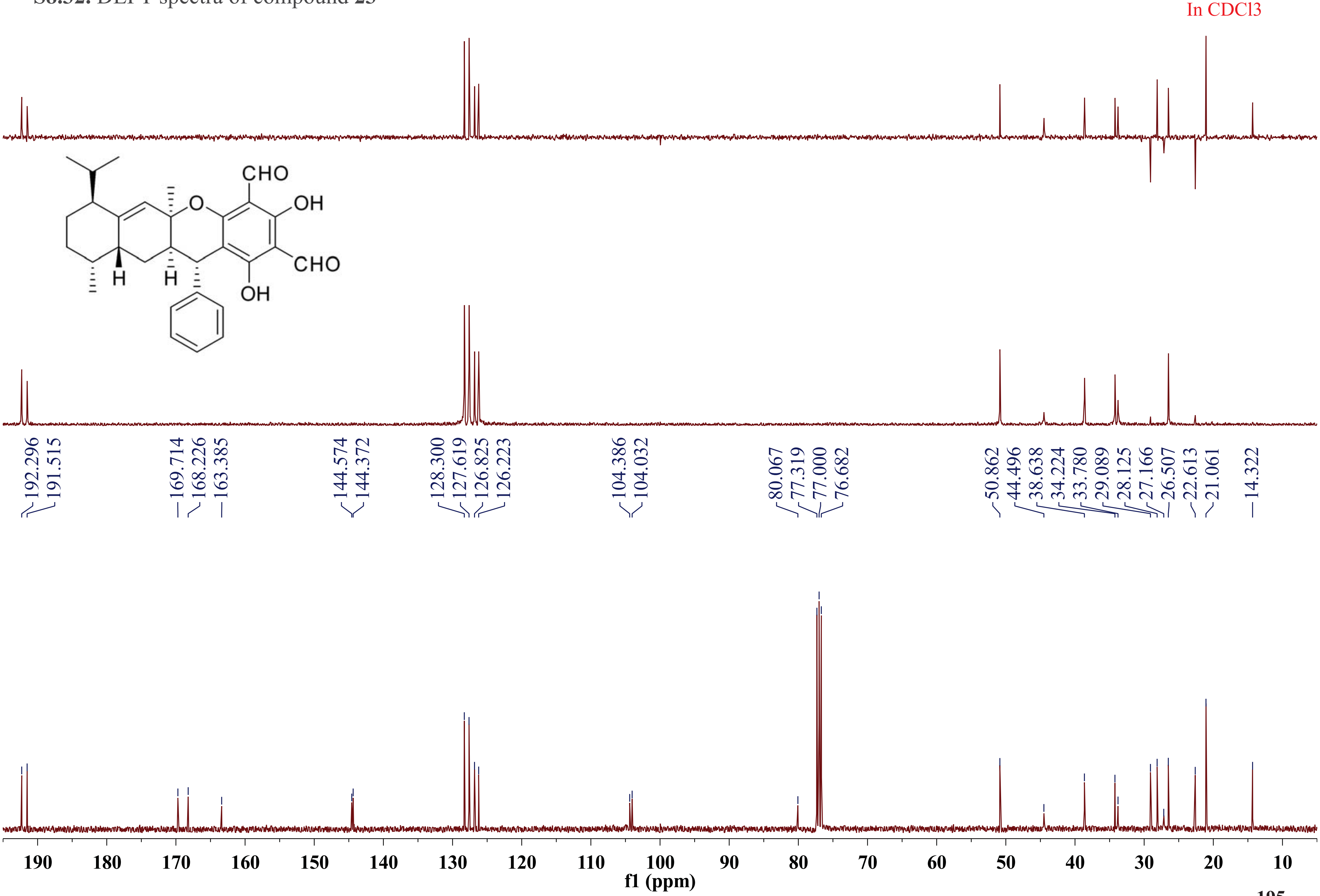

S8.33. ^{1}H NMR spectrum of compound **24**

In CDCl3

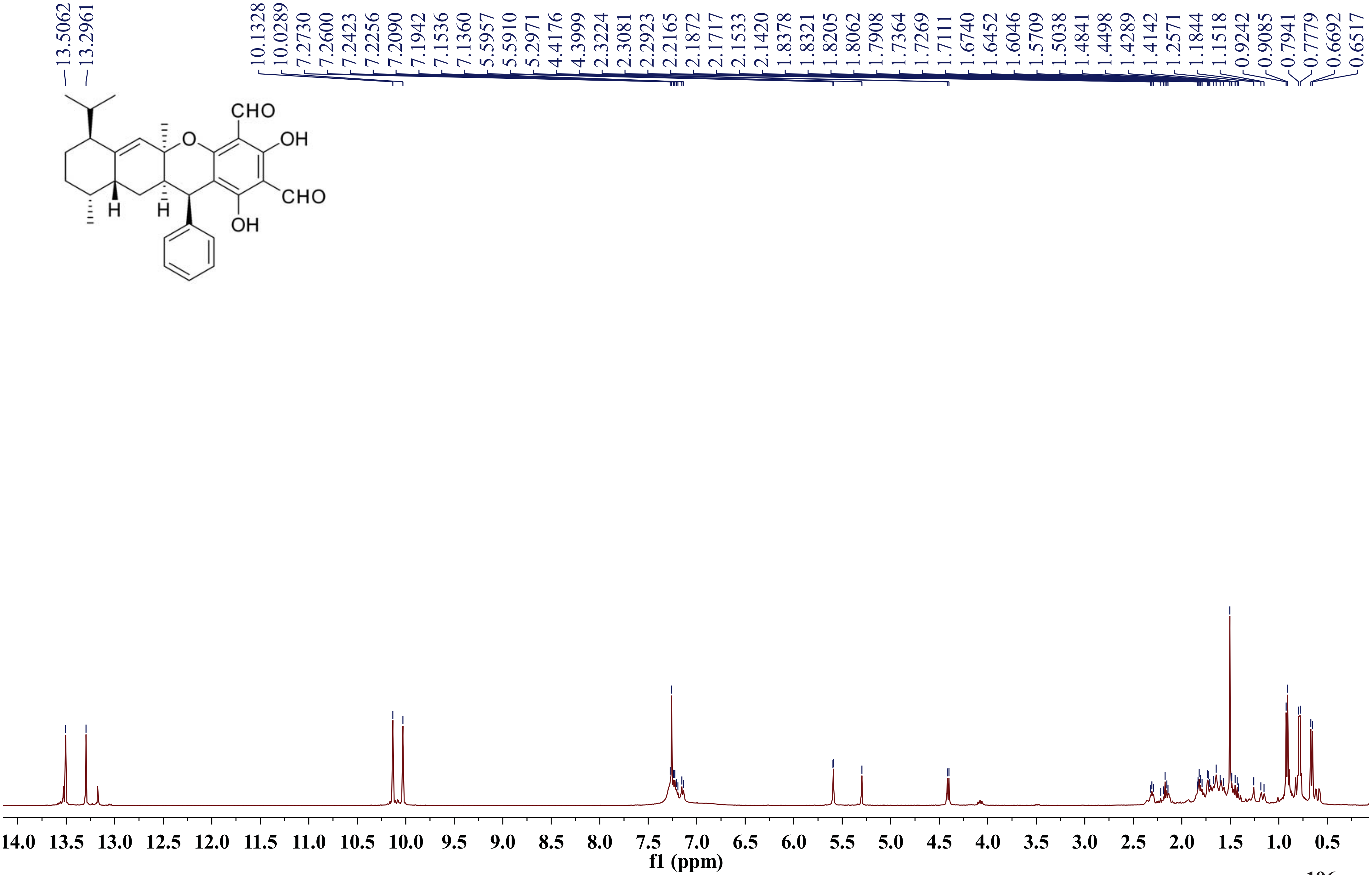

S8.34. ^{13}C NMR spectrum of compound **24**

In CDCl3

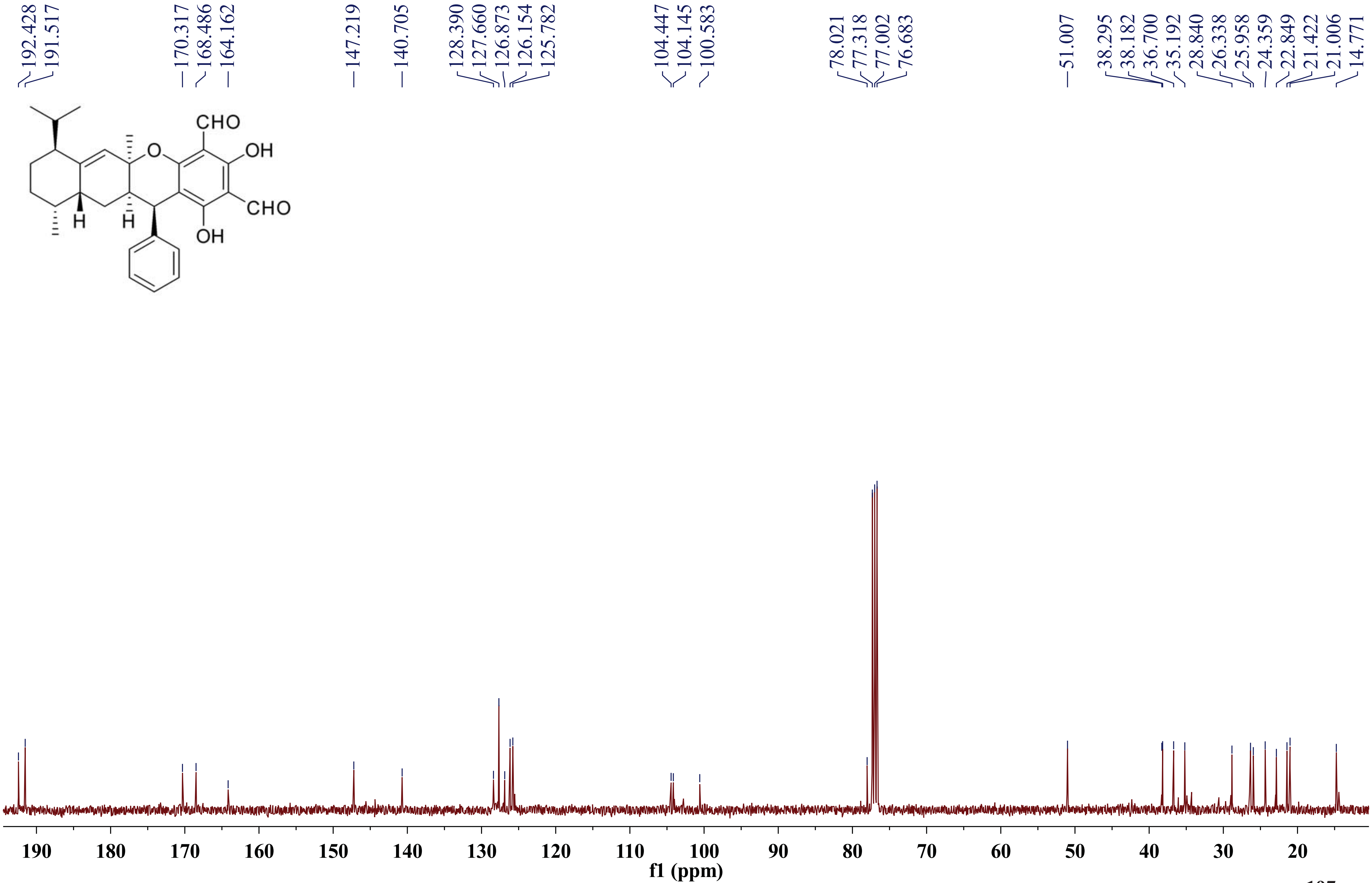

S8.35. ^{1}H NMR spectrum of compound **25**

In CDCl3

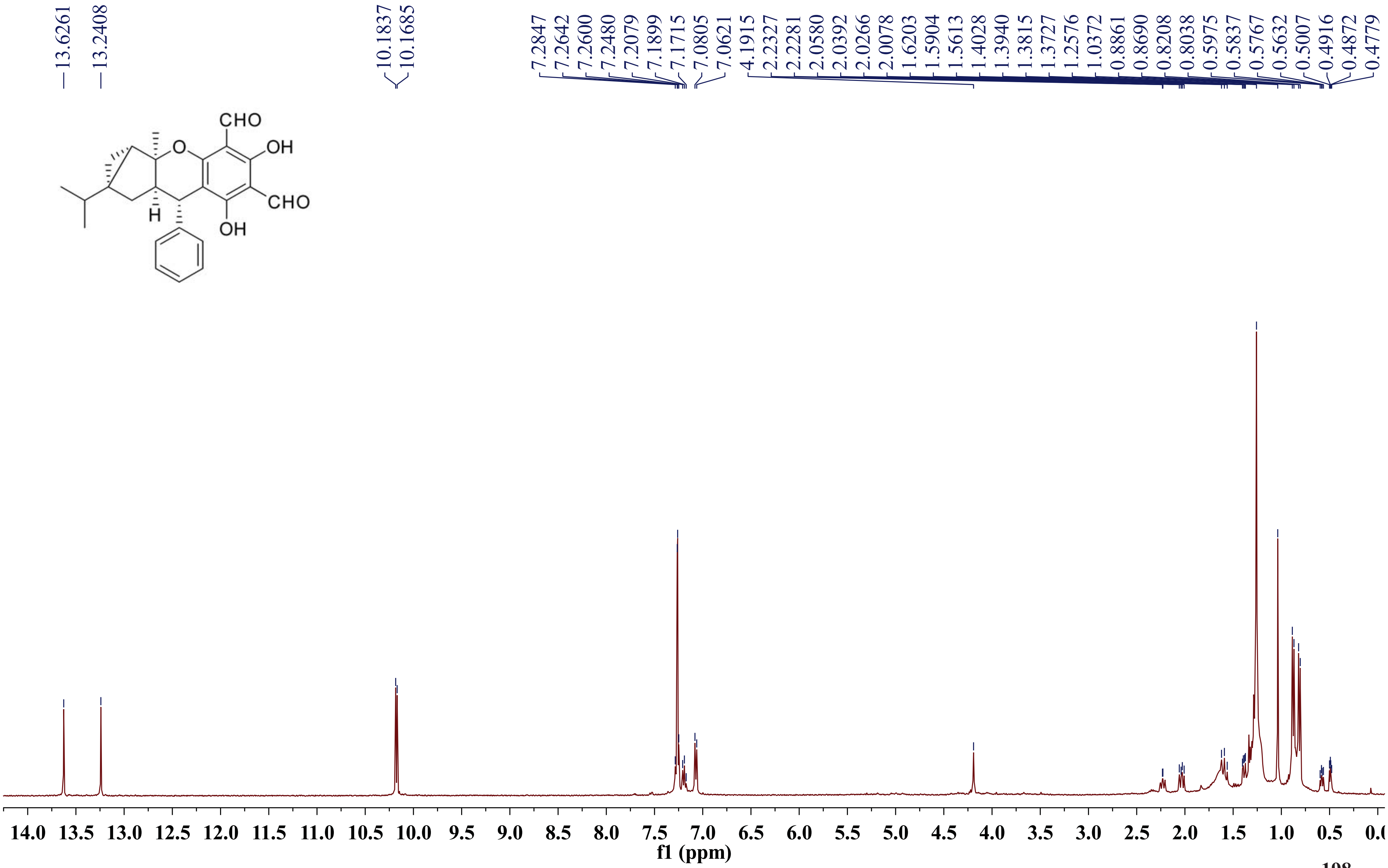

S8.36. DEPT spectra of compound **25**

In CDCl3

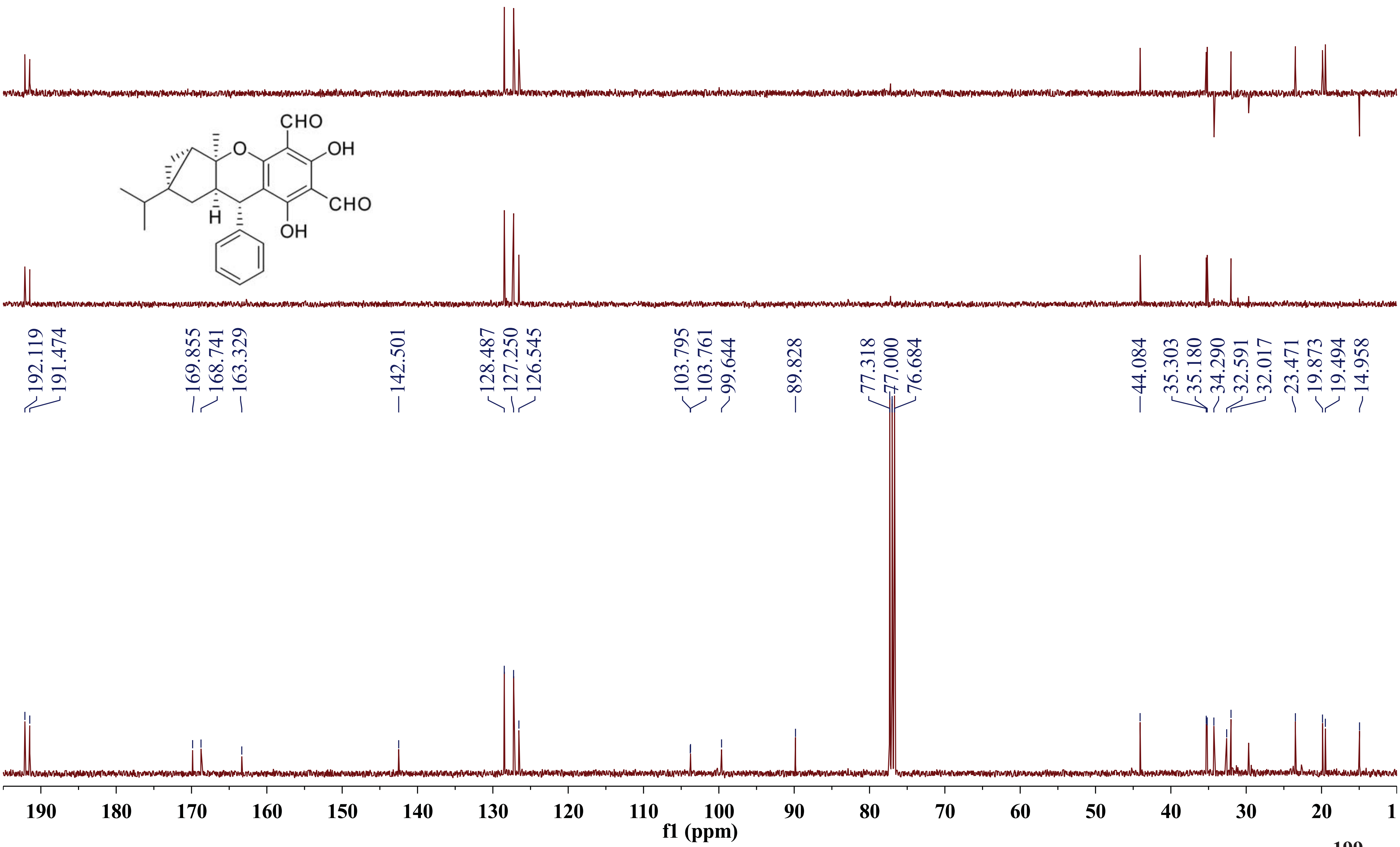

S8.37. HSQC spectrum of compound **25**

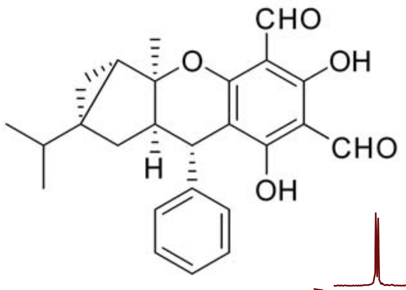

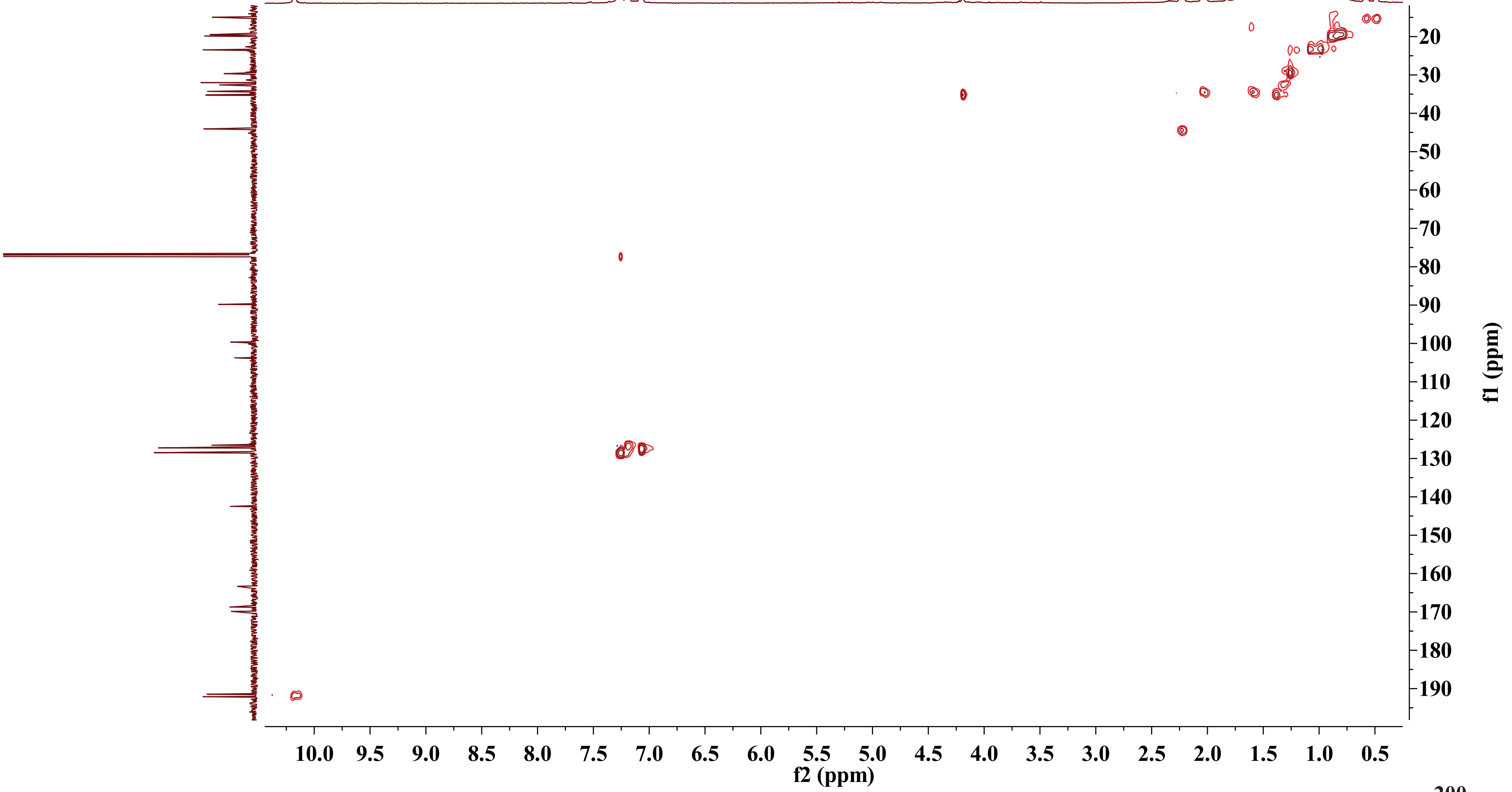

S8.38. ^1H-^1H COSY spectrum of compound **25**

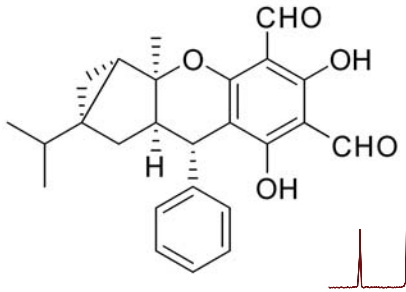

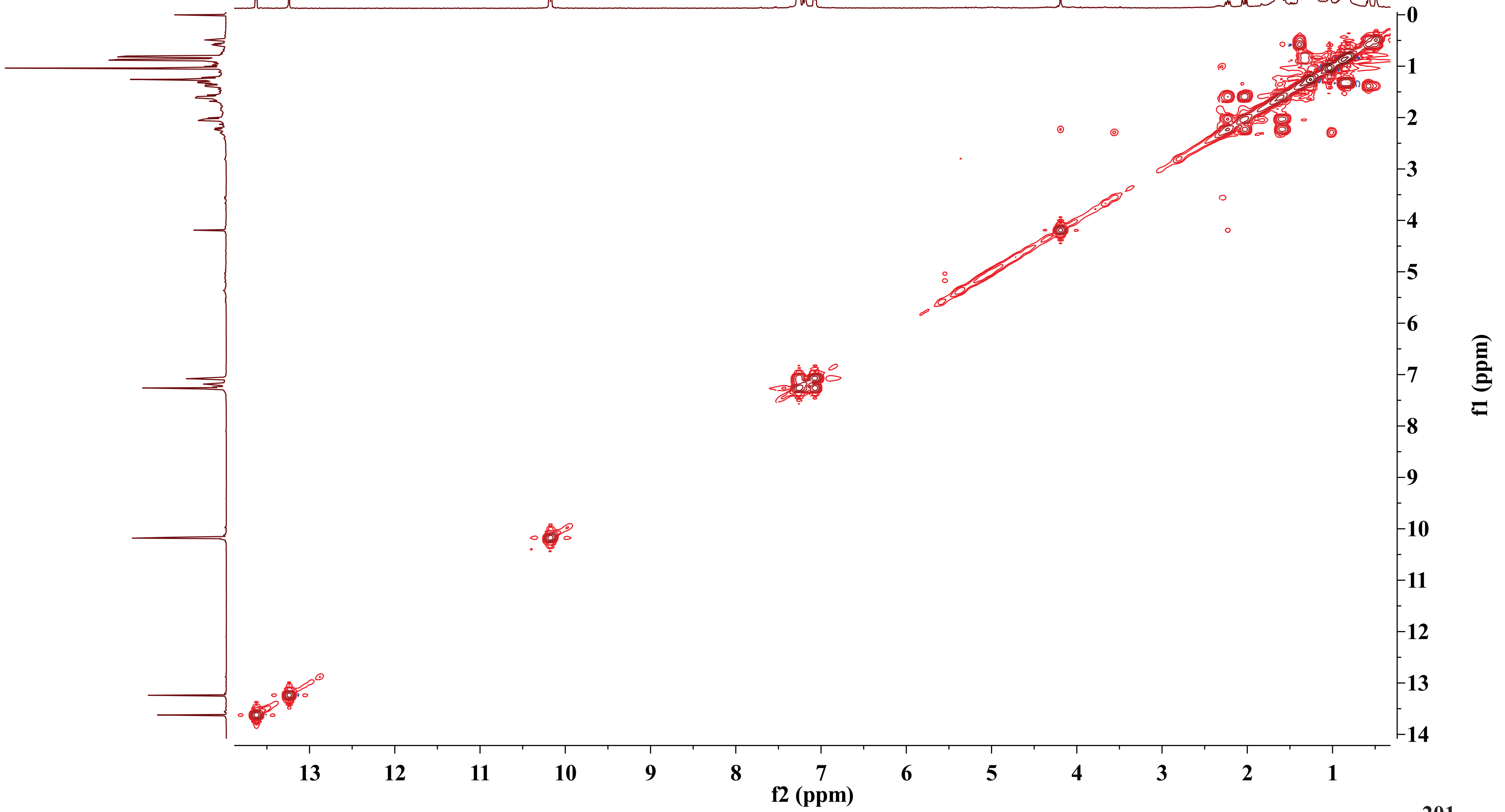

S8.39. HMBC spectrum of compound **25**

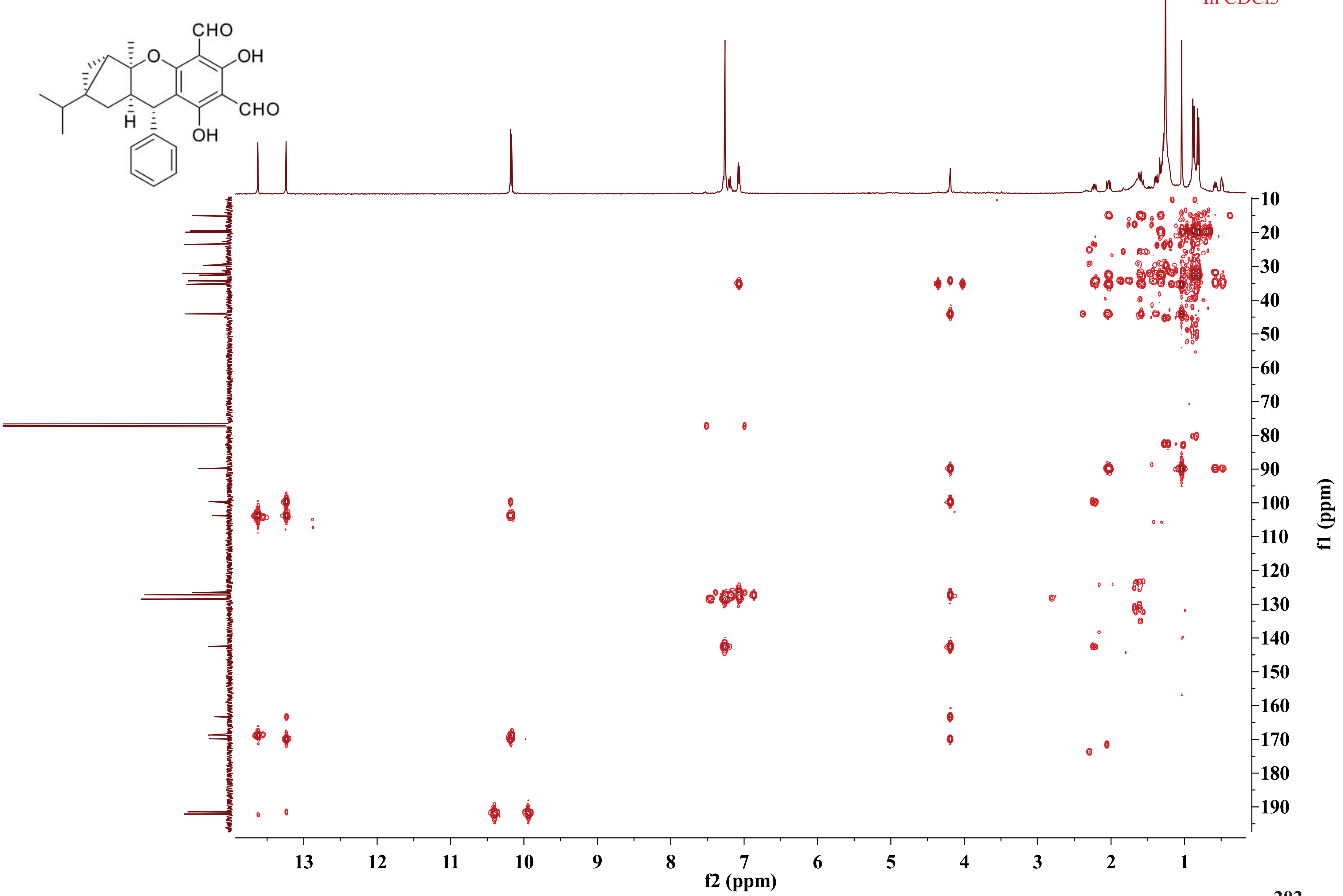

S8.40. NOESY spectrum of compound **25**

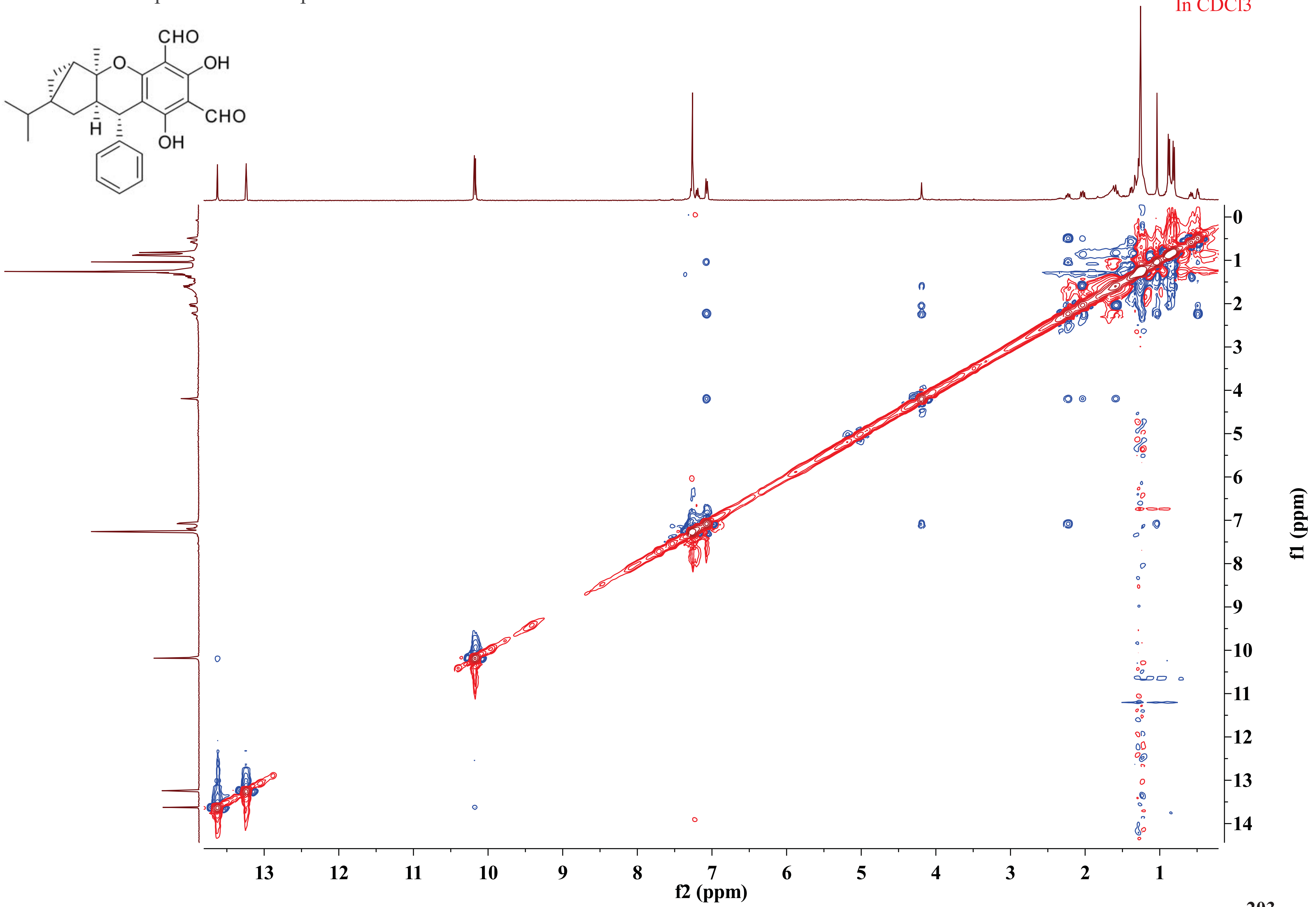

S8.41. ^{1}H NMR spectrum of compound **26**

In CDCl3

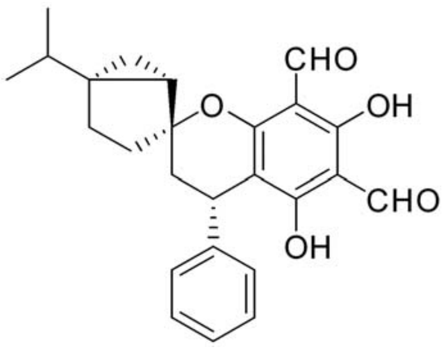

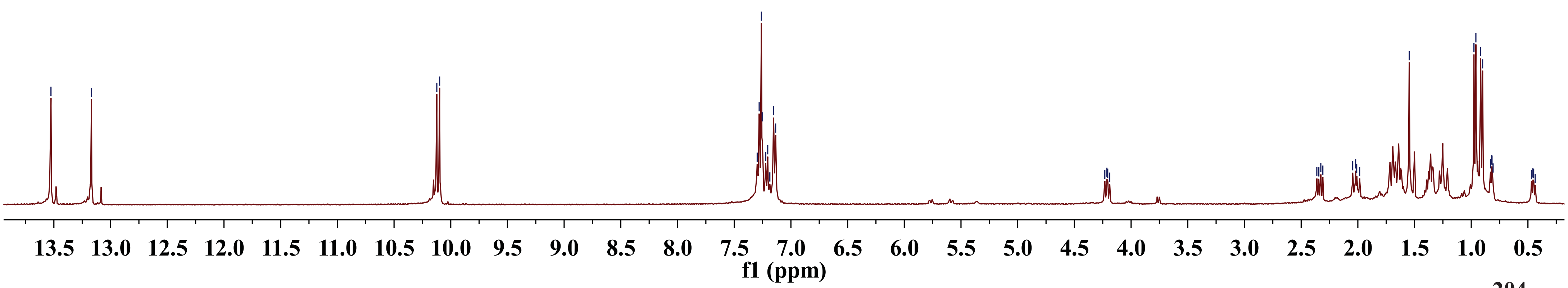

S8.42. DEPT spectra of compound **26**

In CDCl3

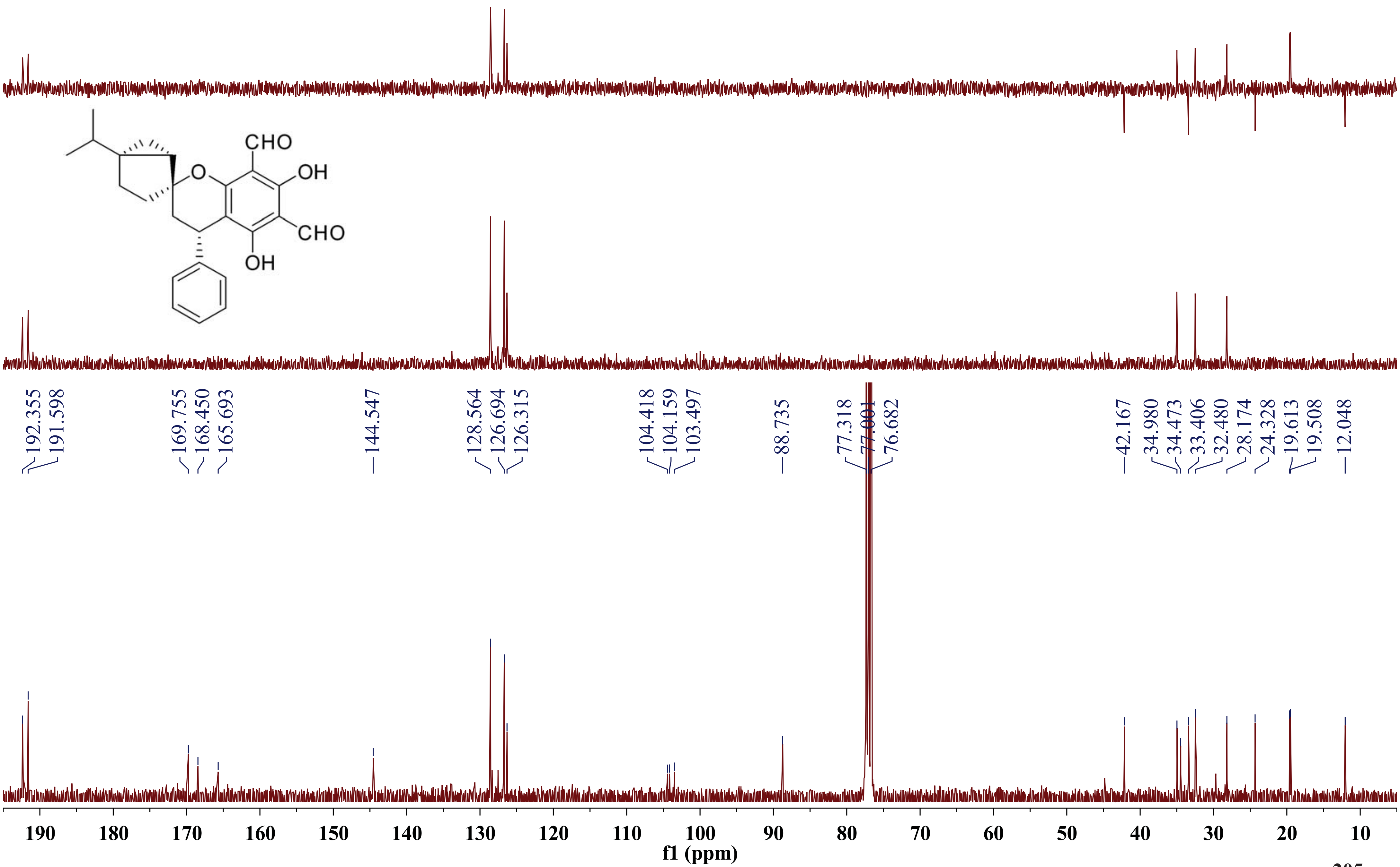

S8.43. DEPT spectra of compound **27**

In CDCl3

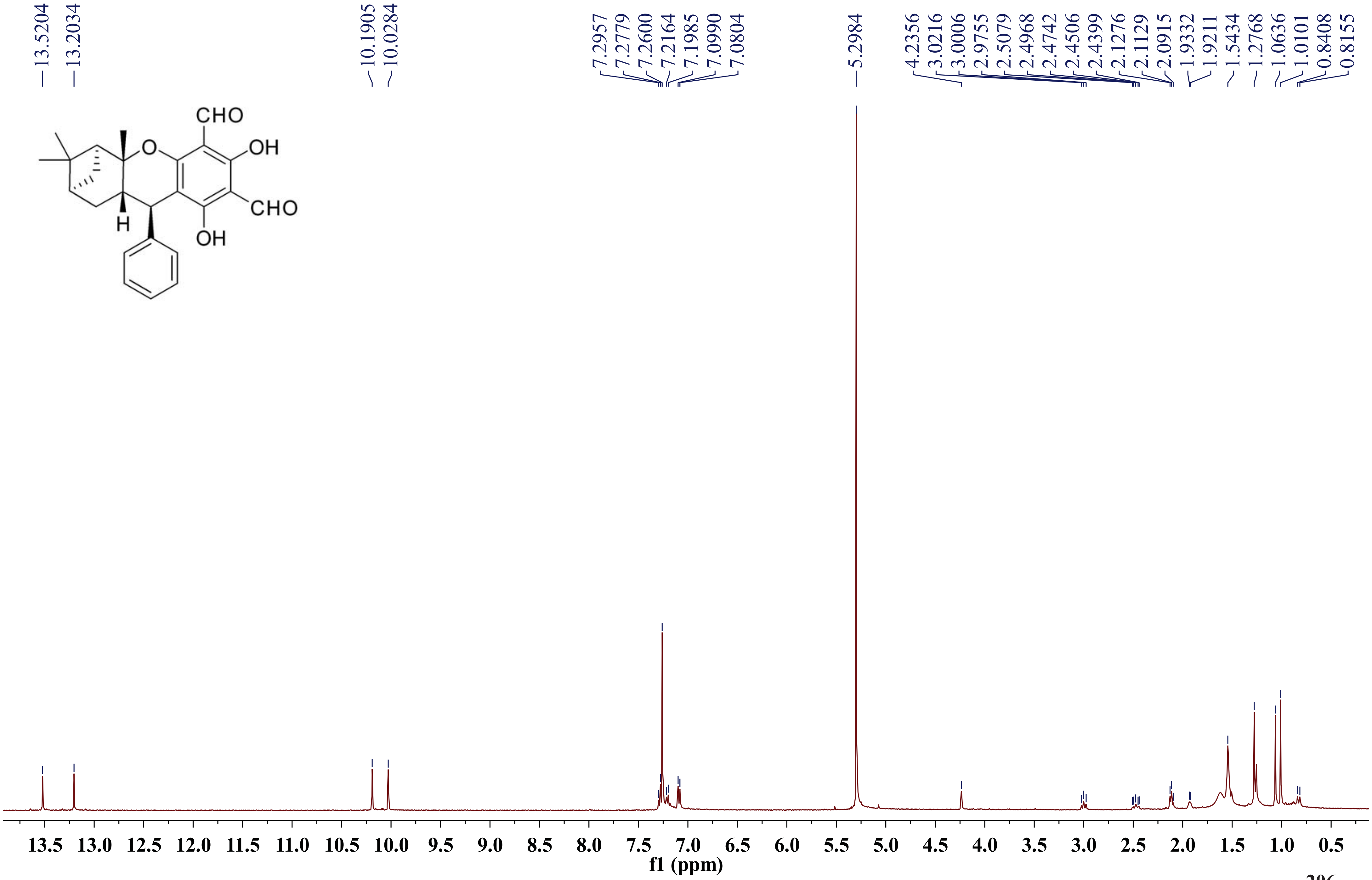

S8.44. DEPT spectra of compound **27**

In CDCl3

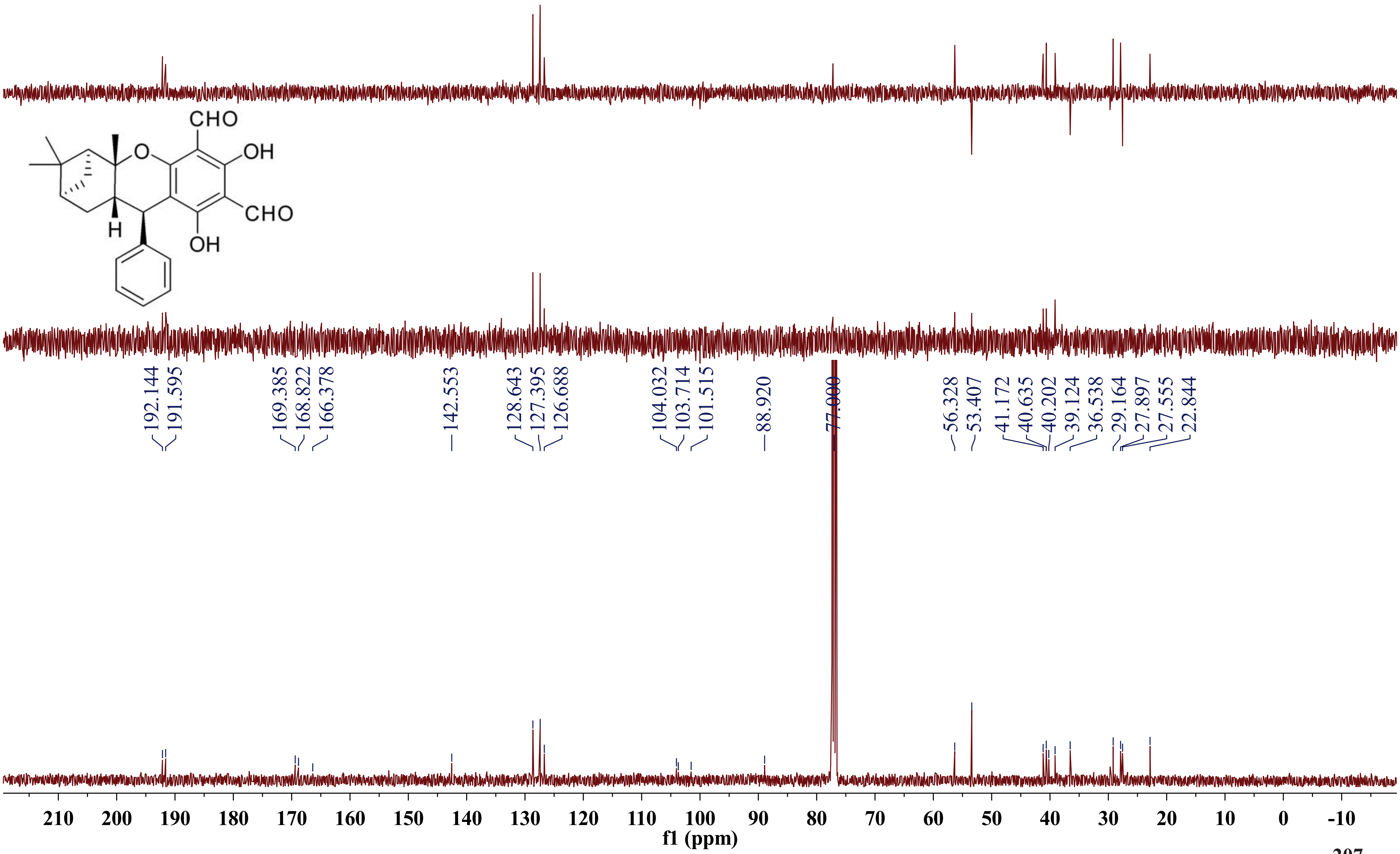

S8.45. ^{1}H NMR spectrum of compound **28**

In CDCl3

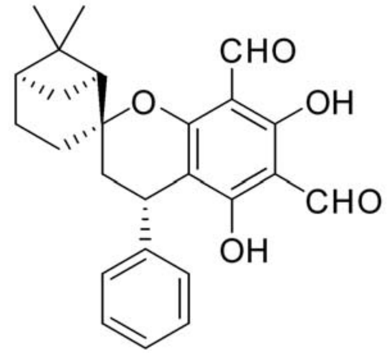

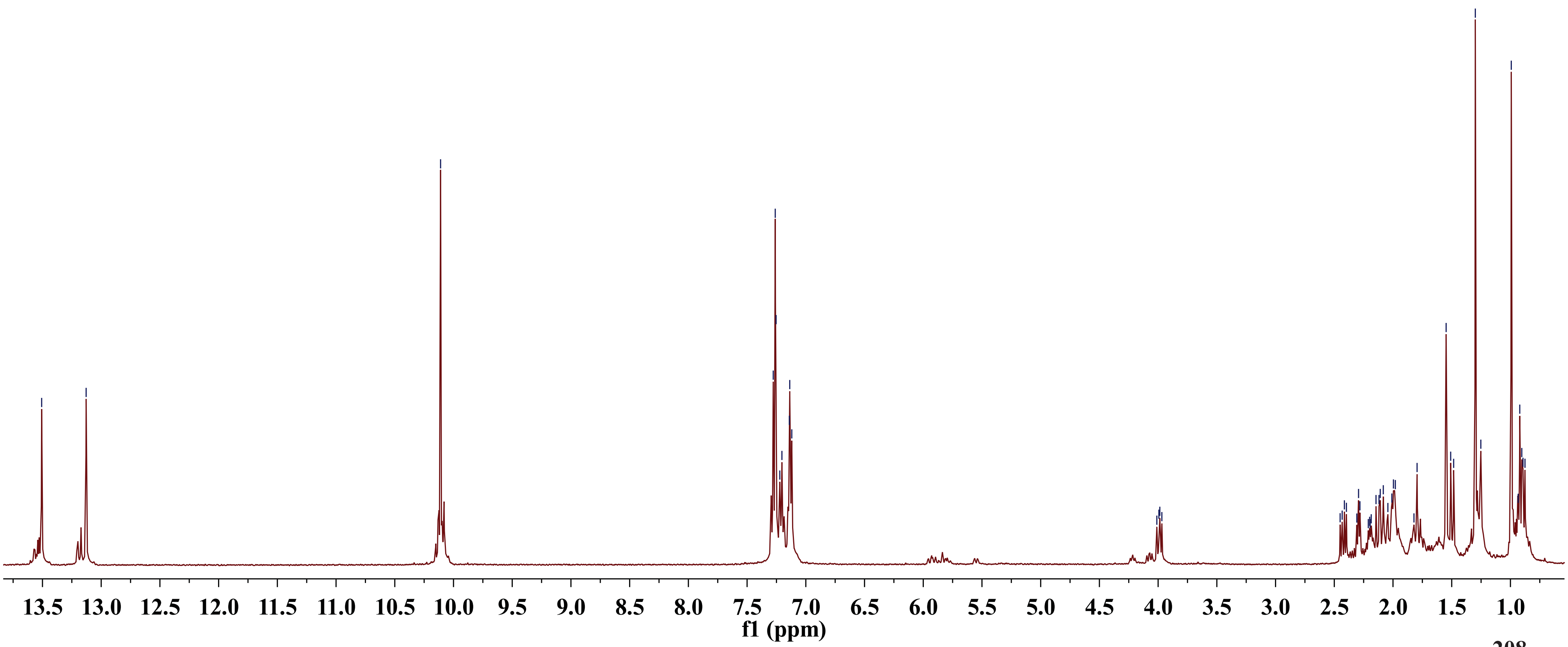

S8.46. DEPT spectra of compound **28**

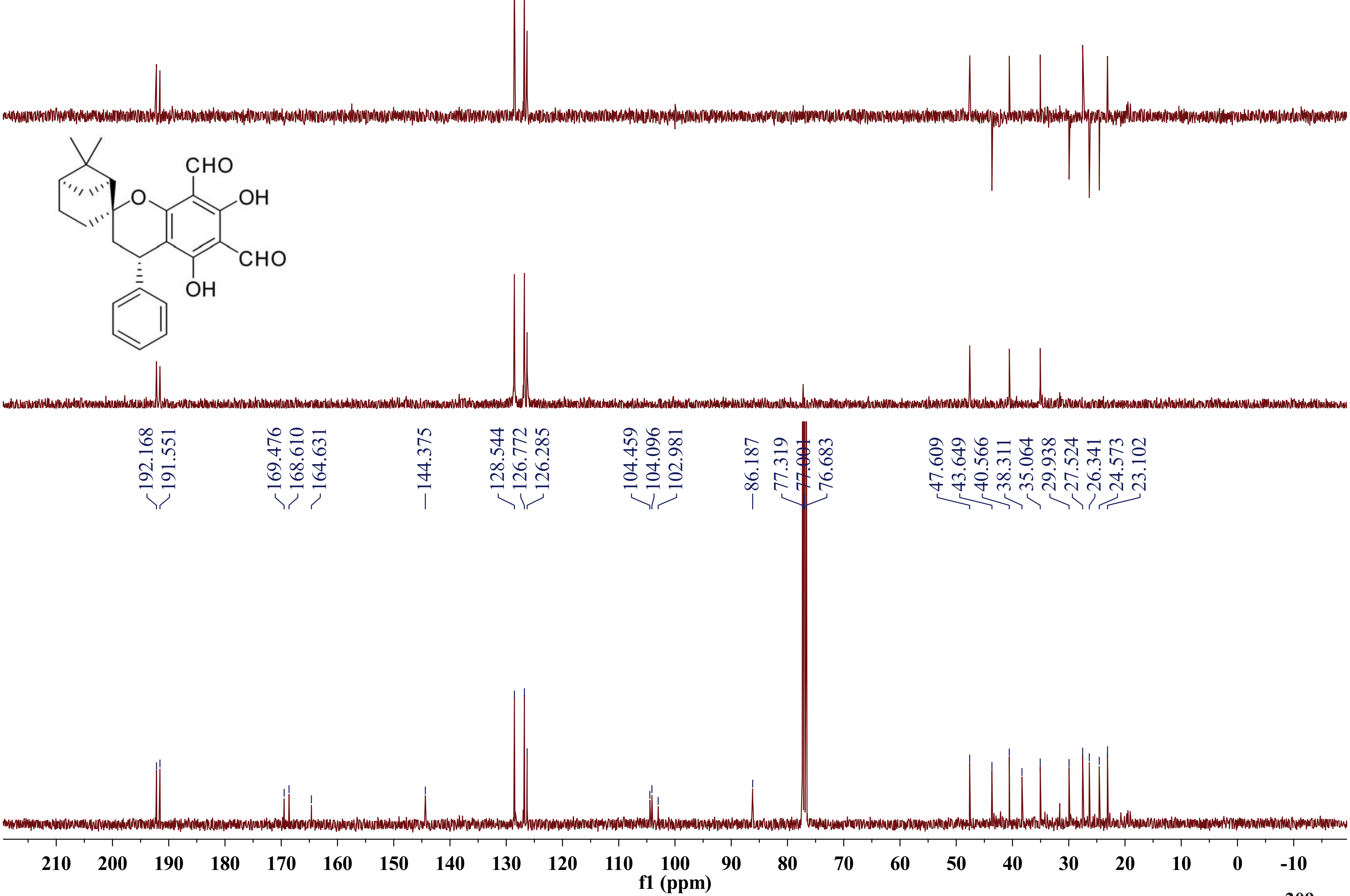

Supplement: Supplementary file 1 — Supplementary Information [file 41598_2017_1028_MOESM1_ESM.pdf]
